# Supplementary material for: The helicase domain of human Dicer prevents RNAi-independent activation of antiviral and inflammatory pathways
Source: EMBO J. 2024 Jan 29;43(5):7. doi: 10.1038/s44318-024-00035-2 (PMC10907635; doi:10.1038/s44318-024-00035-2)
Supplement: Supplementary file 1 — Appendix [file 44318_2024_35_MOESM1_ESM.pdf]

Appendix

The helicase domain of human Dicer prevents RNAi-independent activation of antiviral and inflammatory pathways

Morgane Baldaccini, Léa Gaucherand, Béatrice Chane-Woon-Ming, Mélanie Messmer, Floriane Gucciardi, Sébastien Pfeffer*

Table of Contents

	Page
**Appendix Figure S1**	2
**Appendix Figure S2**	3
**Appendix Figure S3**	4
**Appendix Figure S4**	5
**Appendix Table S1**	6

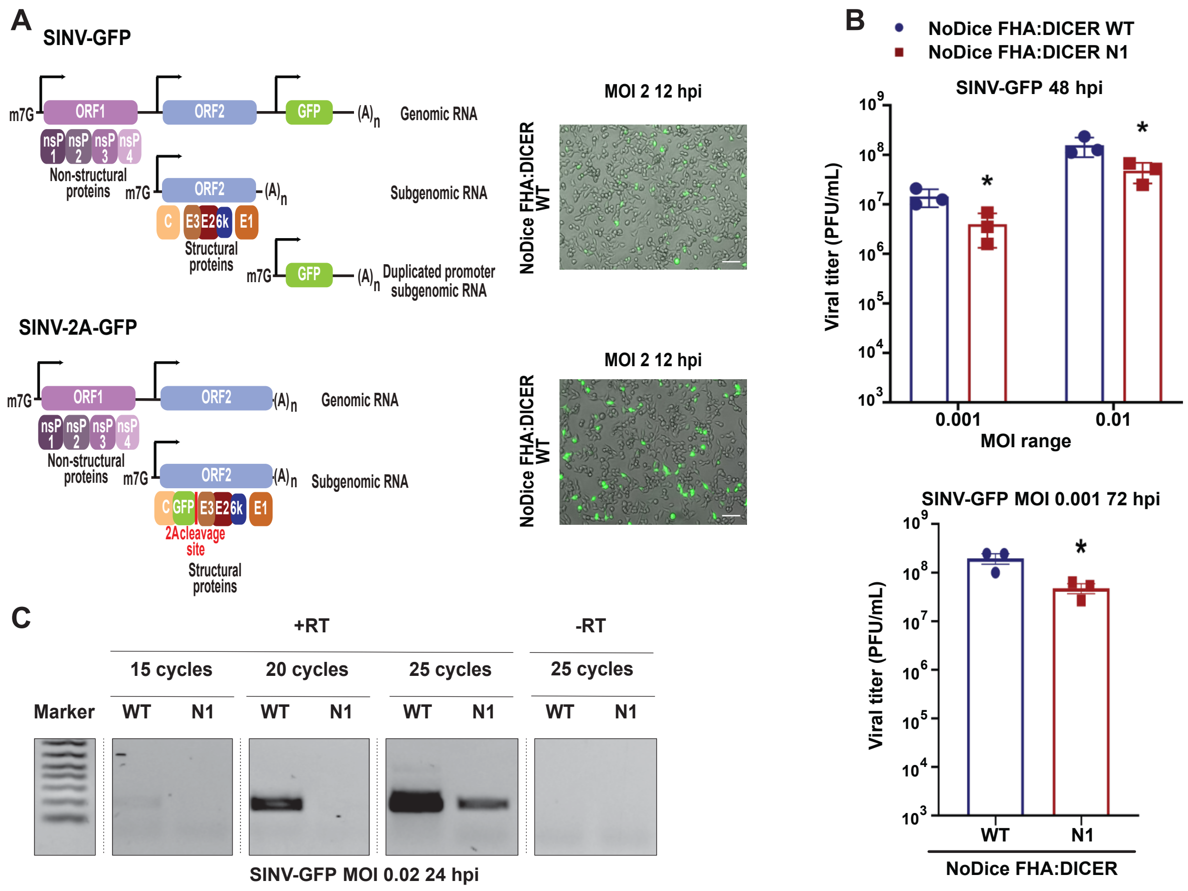

Appendix Figure S1. Dicer N1 reduces SINV replication at longer infection times and decreases antigenome production.

A. Schematic representation of SINV-GFP and SINV-2A-GFP genome organization and representative fluorescent pictures of GFP expressed by the two viruses upon infection of NoDice FHA:DICER WT cells at an MOI of 2 for 12h. Scale bar = 50µm, (n = 3 biological replicates). ORF: Open reading frame, nsP: non-structural proteins, C: capsid, E1 to E3: envelope proteins.

B. Mean (+/- SEM) SINV-GFP viral titer in NoDice FHA:DICER WT and N1 infected at an MOI 0.001 and 0.01 for 48h (up) and 0.001 for 72h (bottom) from plaque assay quantification (n = 3 biological replicates). Upper panel: ordinary two-way ANOVA test with Sidak's correction. *: $p < 0.05$. Bottom panel: Unpaired t-test. *: $p<0.05$.

C. Agarose gel of RT-PCR on SINV antigenome in SINV-GFP infected NoDice FHA:DICER WT and N1 cells at an MOI of 0.02 for 24h (n = 3 biological replicates).

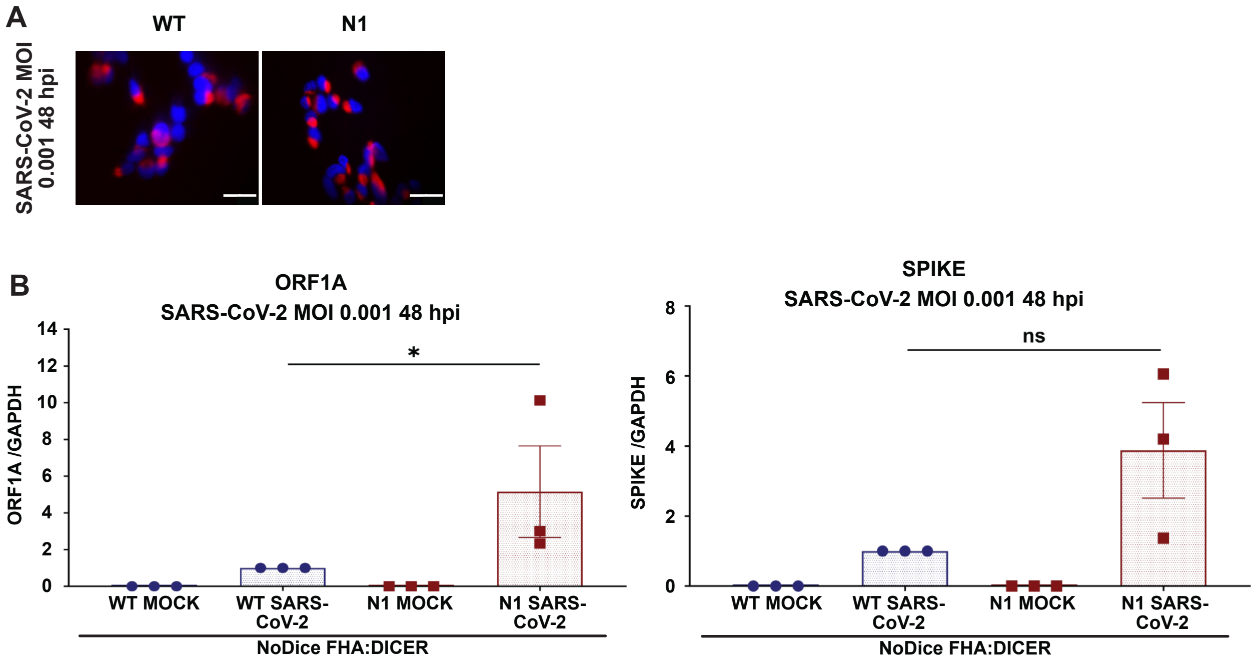

Appendix Figure S2. Dicer N1 enhances SARS-CoV-2 replication.
A. Immunofluorescence analysis on NoDice FHA:DICER WT and N1 cells in SARS-CoV-2 infected cells at an MOI of 0.001 for 48h. J2 antibody (red) was used to detect dsRNA upon infection. DAPI was used to stain the nuclei (blue). Scale bar = 100 µm, (n = 3 biological replicates).
B. RT-qPCR on SARS-CoV-2 genome (ORF1A and SPIKE) in NoDice FHA:DICER WT and N1 cells infected with SARS-CoV-2 at an MOI of 0.001 for 48h. Mean (+/-SEM); n = 3 biological replicates. Unpaired t-test. *: $p<0.05$; ns: non-significant.

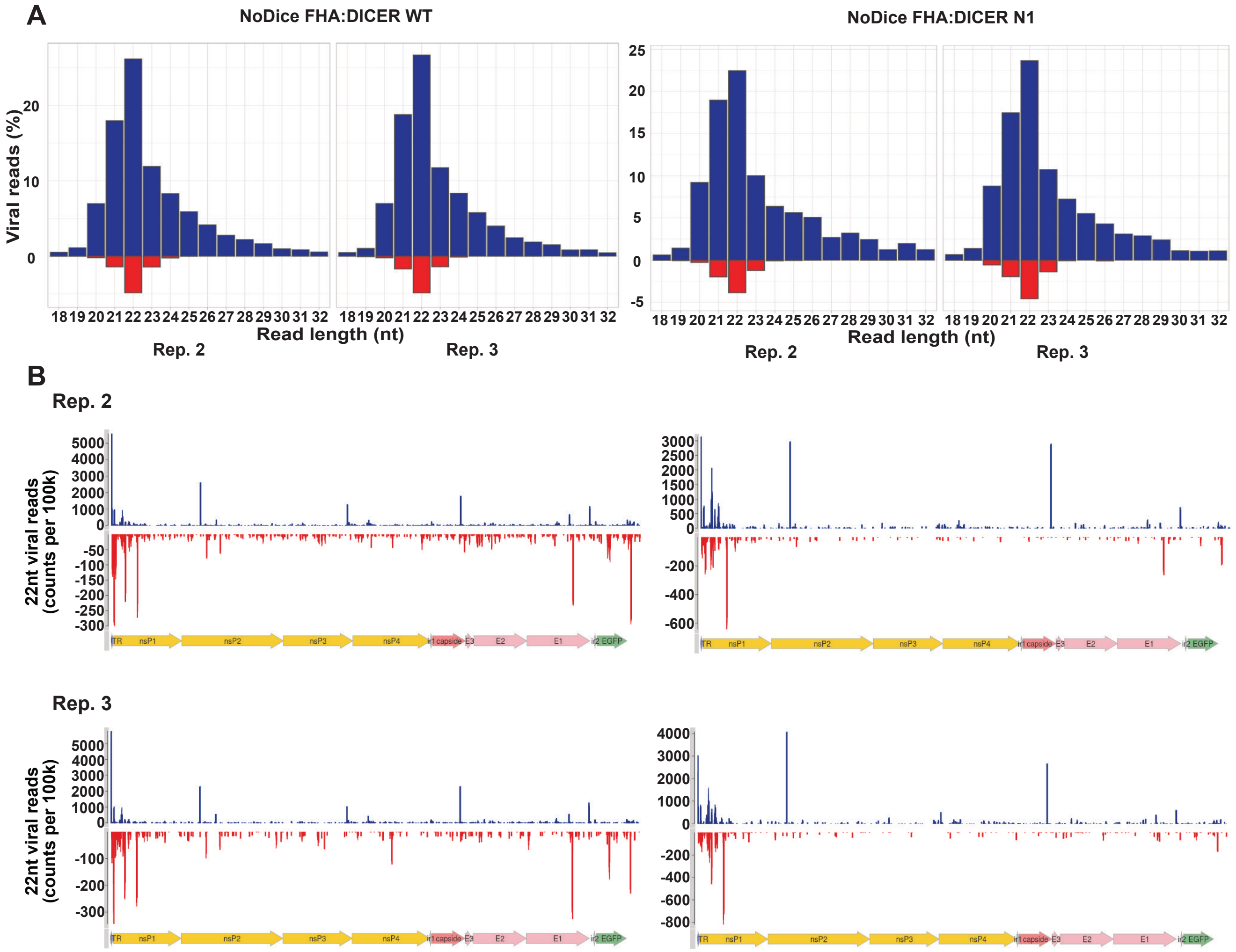

Appendix Figure S3. Dicer N1 cells do not accumulate more virus-derived small RNAs than Dicer WT cells.

A. Representative histograms of the distribution of viral reads (in percent) per RNA length upon small RNA sequencing for replicates 2 and 3 in NoDice FHA:DICER WT (left) and N1 (right) cells infected with SINV-GFP at an MOI of 0.02 for 24h. Blue: positive strand; red: negative strand.

B. Representative graphs of the mapping of 22-nt-reads on SINV-GFP genome for replicates 2 and 3 in NoDice FHA:DICER WT (left) and N1 (right) cells.

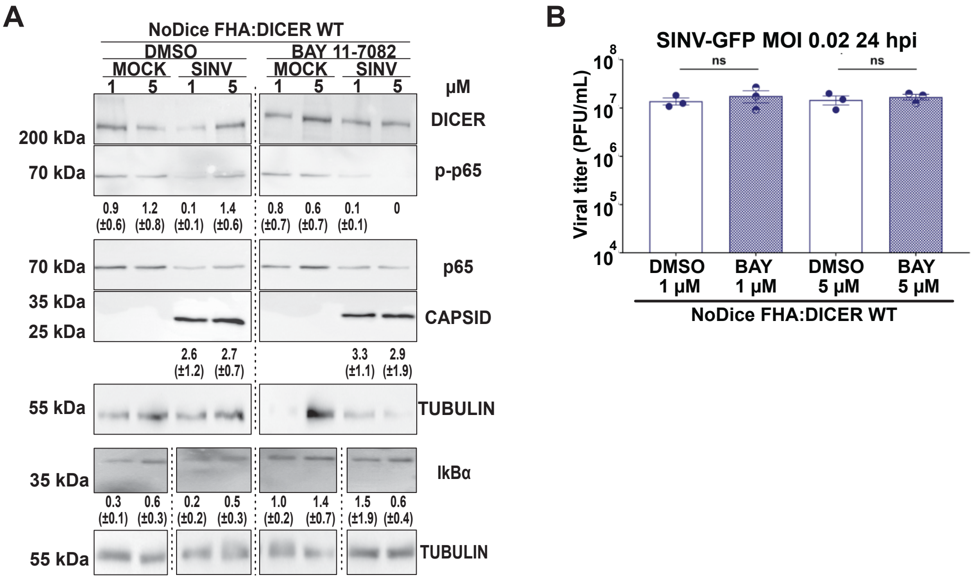

Appendix Figure S4. NF-kB/p65 is not involved in the infection outcome in Dicer WT cells.

A. Western blot analysis of DICER, p-p65, p65, CAPSID and IkBα expression in SINV-GFP infected NoDice FHA:DICER WT cells at an MOI of 0.02 for 24h. Before infection, cells were treated with the NF-kB/p65 inhibitor, BAY 11-7082 or the vehicle (DMSO) at the indicated concentrations for 1 h. Alpha-Tubulin was used as loading control. Mean (+/- SD) of bands quantification of three independent replicates normalized to Tubulin are represented under the corresponding lane; p-p65 = p-p65/p65 quantification.

B. Mean (+/- SEM) of SINV-GFP viral titers in the same samples as in 1, infected at an MOI of 0.02 for 24h (n = 3 biological replicates) from plaque assay quantification. Ordinary one-way ANOVA test with Sidak's correction. ns: non-significant.

Appendix Table S1. List of oligonucleotides used in this study.

Type	Target	Fw	Rev
**PCR primers for mutagenesis**	PKR K296R	CGTTATTAGACGTGTTAAATATAATAACGAGAAGG	ACACGTCTAATAACGTAAGTCTTTCCG
	PKR T451A	TAAGGGAGCATTGCGATACATGAGCCCAGAAC	CGCAATGCTCCCTTACTCCTTGTTCGCTTTCC
	MYC EMPTY	AGGATCTGACCCAGCTTTCTTGTACAAAGTGG	GCTGGGTCAGATCCTCTTCTGAGATGAGTTT
	N1-CM	ACCAATTCAGTCGACATGGAAGCAGAATTC	GAATTCTGCTTCCATGTCGACTGAATTGGT
	ΔHEL1	CGCCAAGAAAATATCAGGTTAGTAATGCTGAAACTGC	GTTGCAGTTTCAGCATTACTAACCTGATATTTTCTTG
	ΔHEL2i	TTAGACAGAAATTTTCCTTCTCCTTTTACCAAC	AAAATTTCTGTCTAAGACCACCAGGTCAG
	ΔHEL2	CAGAGACACCTGTCATGGATGATGATCACG	TGACAGGTGTCTCTGGCTTCTCTTTTTCTTC
**RT-PCR primers**	RT specific SINV	ATTGACGGCGTAGTACACACTATTGAATCAAACAGCC GACCA	/
	PCR SINV antigenome	TAGACGTAGACCCCCAGAGTC	CATTCTACGAGCCGGTGCGC
**qPCR primers**	SINV genome	CCACTACGCAAGCAGAGACG	AGTGCCCAGGGCCTGTGTCCG
	GAPDH	CTTTGGTATCGTGGAAGGACT	CCAGTGAGCTTCCCGTTCAG
	OAS3	TGCTGCCAGCCTTTGACGCC	TCGCCCGCATTGCTGTAGCTG
	PTGS2	ACCCACTCCAAACACAGTGC	GCTTCCCAGCTTTTGTAGCC
	APOBEC3B	GACCCTTTGGTCCTTCGAC	GCACAGCCCCAGGAGAAG
	MX1	AAGCTGATCCGCCTCCACTT	TGCAATGCACCCCTGTATACC
	IFN ß	AAGGCCAAGGAGTACAGTC	ATCTTCAGTTTVGGAGGTAA
	IFIT3	ATGAGTGAGGTCACCAAGAATTCCC	GGCTGCCTCGTTGTTACCAT
**Northern blot probes**	miR-16	CGCCAATATTTACGTGCTGCTA	
	U6	GCAGGGGCCATGCTAATCTTCTCTGTATCG	
