# Supplementary figures and images for: The helicase domain of human Dicer prevents RNAi-independent activation of antiviral and inflammatory pathways (part 1 of 5)
Source: EMBO J. 2024 Jan 29;43(5):7. doi: 10.1038/s44318-024-00035-2 (PMC10907635; doi:10.1038/s44318-024-00035-2)

**B**

**Replicate 1**

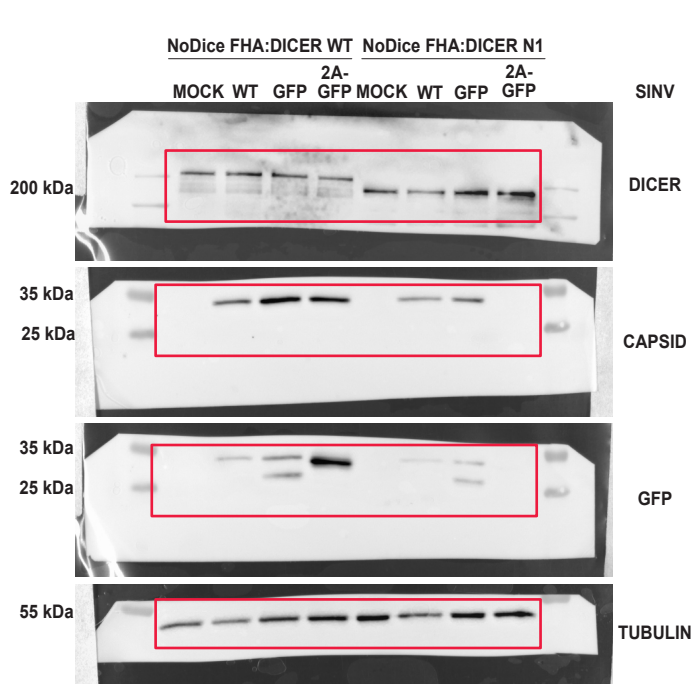

**Replicate 2**

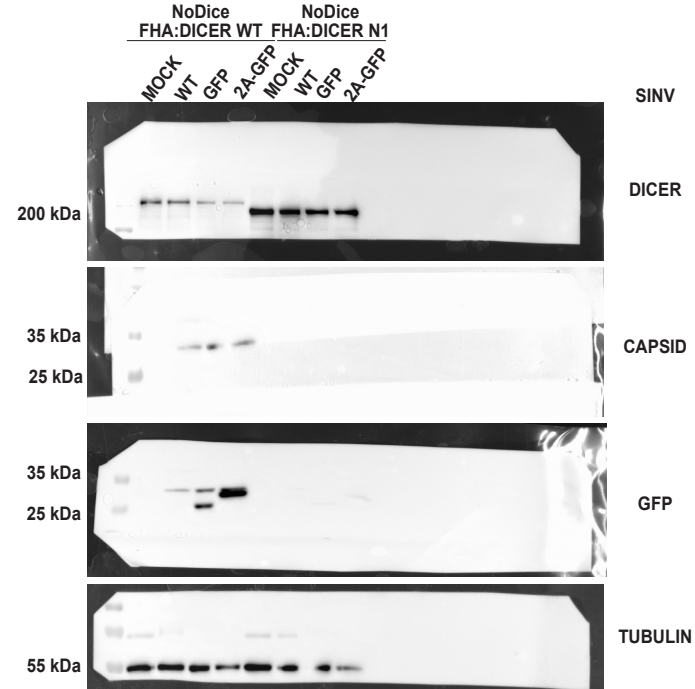

**Replicate 3**

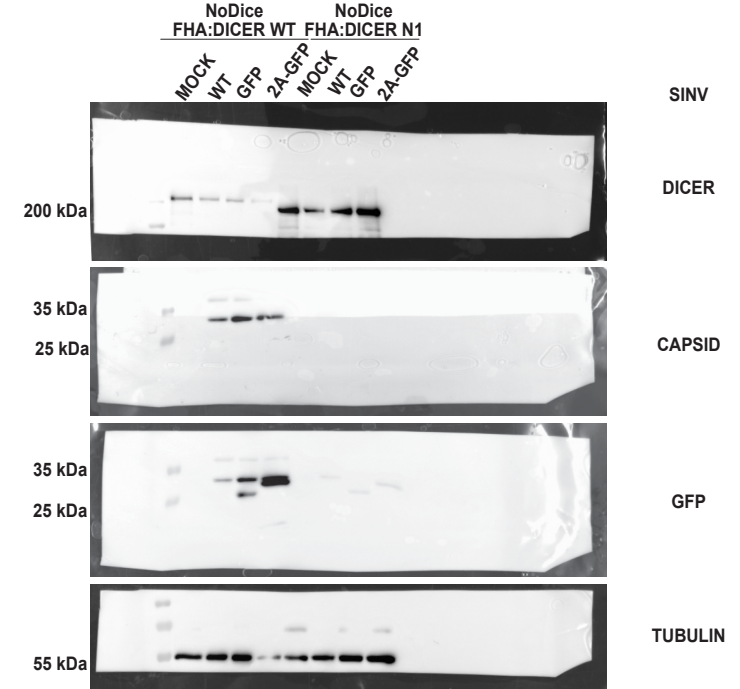

C

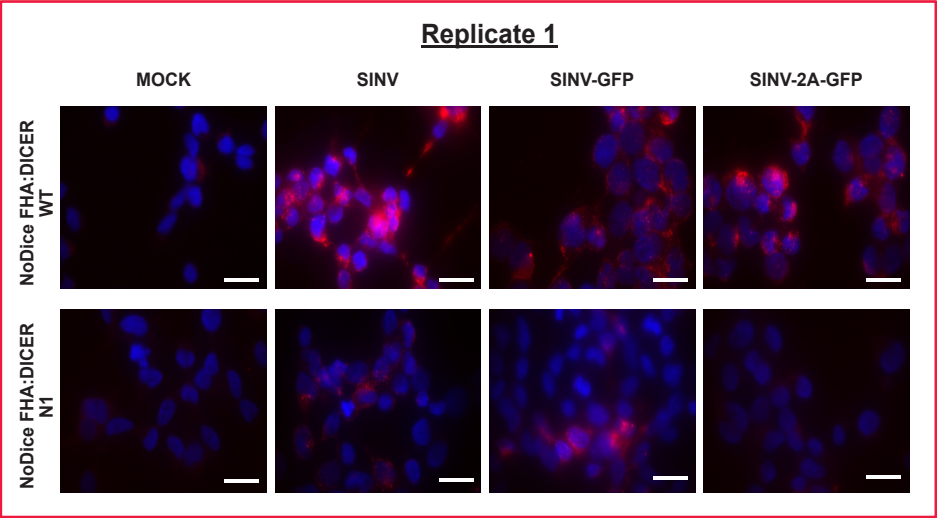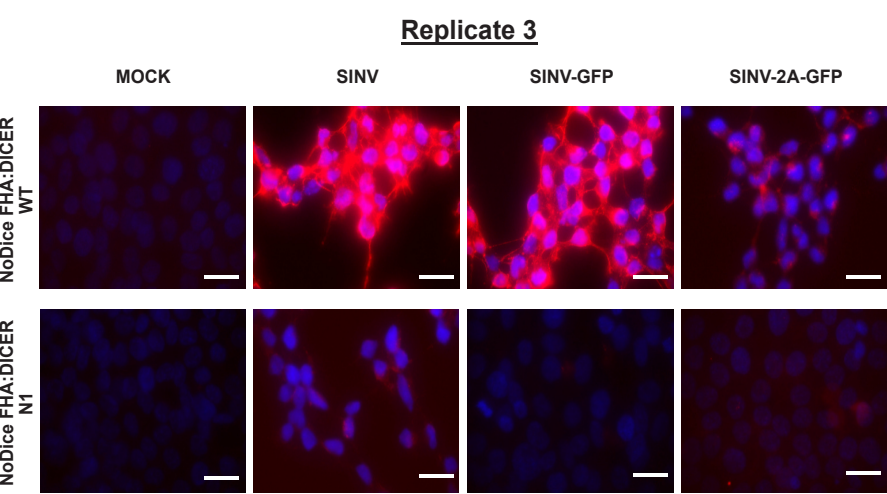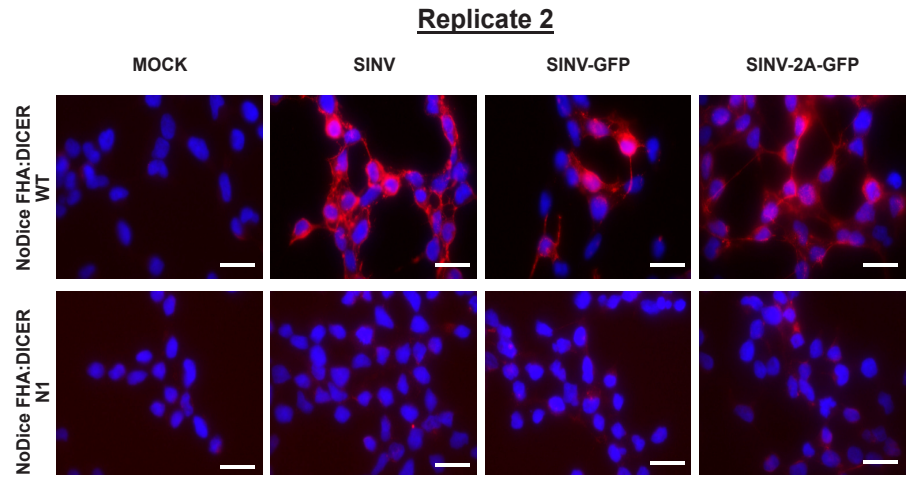

Supplement: Supplementary file 2 — Source Data Fig. 1 [file 44318_2024_35_MOESM2_ESM.zip › EMBOJ-2023-115792R2_SourceData_Fig1/Figure1.pdf]

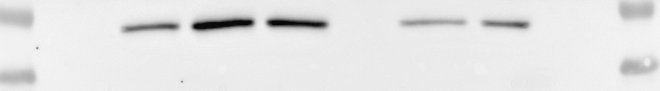

Supplement: Supplementary file 2 — Source Data Fig. 1 [file 44318_2024_35_MOESM2_ESM.zip › EMBOJ-2023-115792R2_SourceData_Fig1/Fig1B/R1/western capsid.tiff]

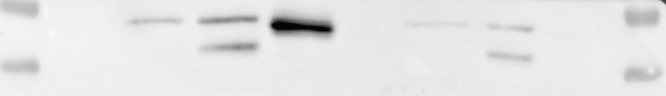

Supplement: Supplementary file 2 — Source Data Fig. 1 [file 44318_2024_35_MOESM2_ESM.zip › EMBOJ-2023-115792R2_SourceData_Fig1/Fig1B/R1/western gfp.tiff]

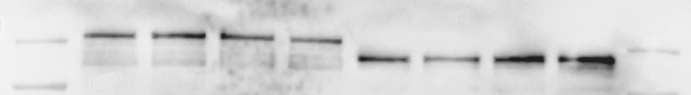

Supplement: Supplementary file 2 — Source Data Fig. 1 [file 44318_2024_35_MOESM2_ESM.zip › EMBOJ-2023-115792R2_SourceData_Fig1/Fig1B/R1/western dicer.tiff]

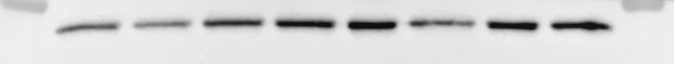

Supplement: Supplementary file 2 — Source Data Fig. 1 [file 44318_2024_35_MOESM2_ESM.zip › EMBOJ-2023-115792R2_SourceData_Fig1/Fig1B/R1/western tubulin.tiff]

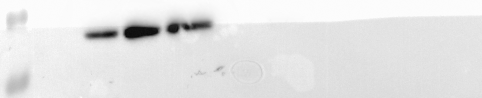

Supplement: Supplementary file 2 — Source Data Fig. 1 [file 44318_2024_35_MOESM2_ESM.zip › EMBOJ-2023-115792R2_SourceData_Fig1/Fig1B/R3/western capsid.tiff]

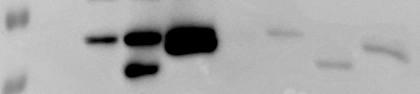

Supplement: Supplementary file 2 — Source Data Fig. 1 [file 44318_2024_35_MOESM2_ESM.zip › EMBOJ-2023-115792R2_SourceData_Fig1/Fig1B/R3/western gfp.tiff]

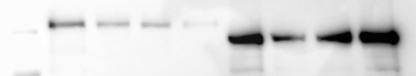

Supplement: Supplementary file 2 — Source Data Fig. 1 [file 44318_2024_35_MOESM2_ESM.zip › EMBOJ-2023-115792R2_SourceData_Fig1/Fig1B/R3/western dicer.tiff]

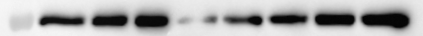

Supplement: Supplementary file 2 — Source Data Fig. 1 [file 44318_2024_35_MOESM2_ESM.zip › EMBOJ-2023-115792R2_SourceData_Fig1/Fig1B/R3/western tubulin.tiff]

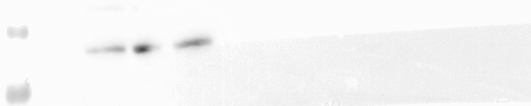

Supplement: Supplementary file 2 — Source Data Fig. 1 [file 44318_2024_35_MOESM2_ESM.zip › EMBOJ-2023-115792R2_SourceData_Fig1/Fig1B/R2/western capsid.tiff]

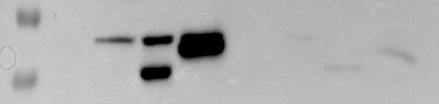

Supplement: Supplementary file 2 — Source Data Fig. 1 [file 44318_2024_35_MOESM2_ESM.zip › EMBOJ-2023-115792R2_SourceData_Fig1/Fig1B/R2/western gfp.tiff]

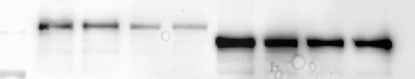

Supplement: Supplementary file 2 — Source Data Fig. 1 [file 44318_2024_35_MOESM2_ESM.zip › EMBOJ-2023-115792R2_SourceData_Fig1/Fig1B/R2/western dicer.tiff]

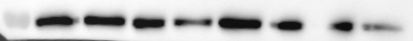

Supplement: Supplementary file 2 — Source Data Fig. 1 [file 44318_2024_35_MOESM2_ESM.zip › EMBOJ-2023-115792R2_SourceData_Fig1/Fig1B/R2/western tubulin.tiff]

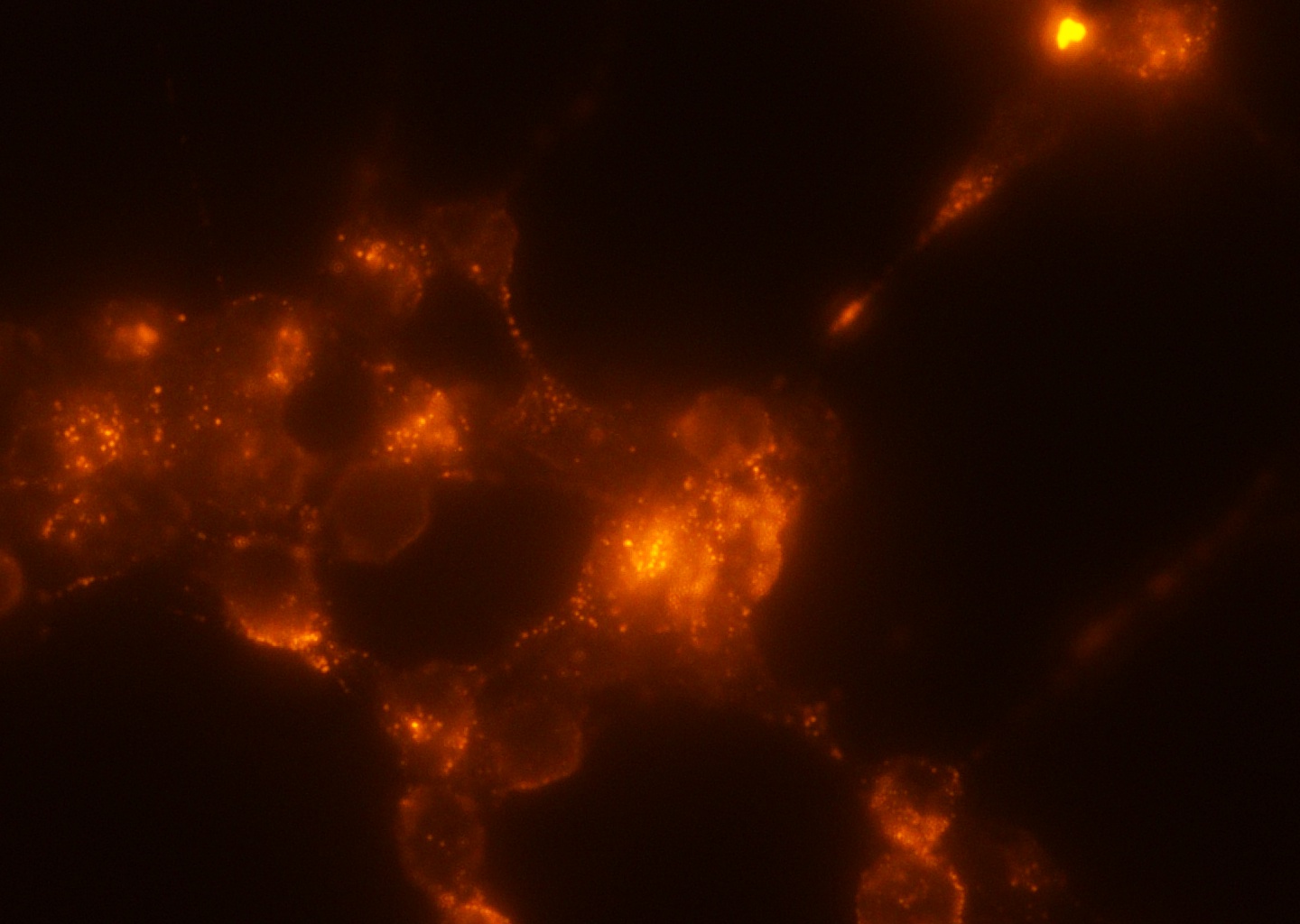

Supplement: Supplementary file 2 — Source Data Fig. 1 [file 44318_2024_35_MOESM2_ESM.zip › EMBOJ-2023-115792R2_SourceData_Fig1/Fig1C/R1/WT SINV WT/J2.jpg]

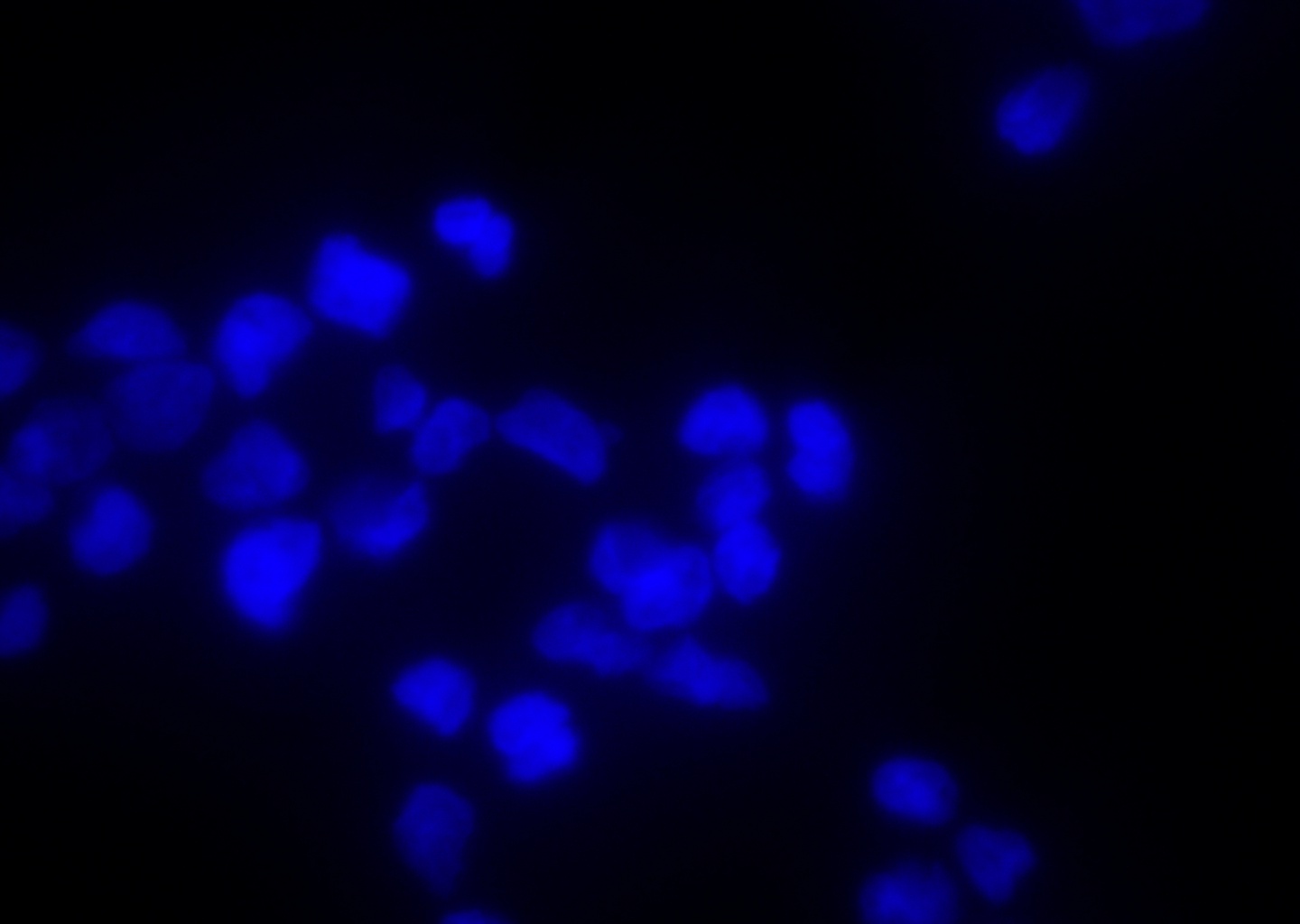

Supplement: Supplementary file 2 — Source Data Fig. 1 [file 44318_2024_35_MOESM2_ESM.zip › EMBOJ-2023-115792R2_SourceData_Fig1/Fig1C/R1/WT SINV WT/DAPI.jpg]

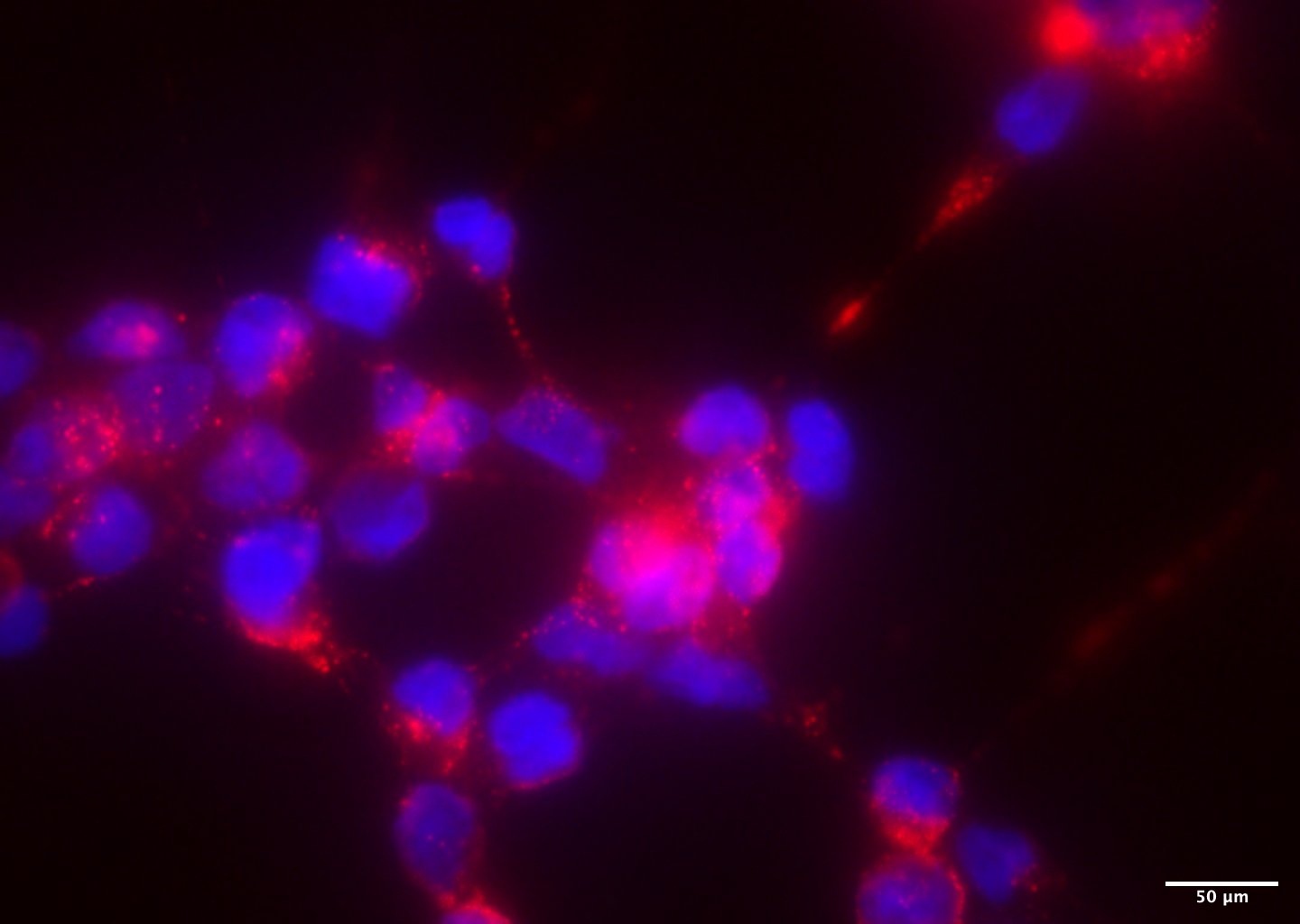

Supplement: Supplementary file 2 — Source Data Fig. 1 [file 44318_2024_35_MOESM2_ESM.zip › EMBOJ-2023-115792R2_SourceData_Fig1/Fig1C/R1/WT SINV WT/WT SINV WT merge.jpg]

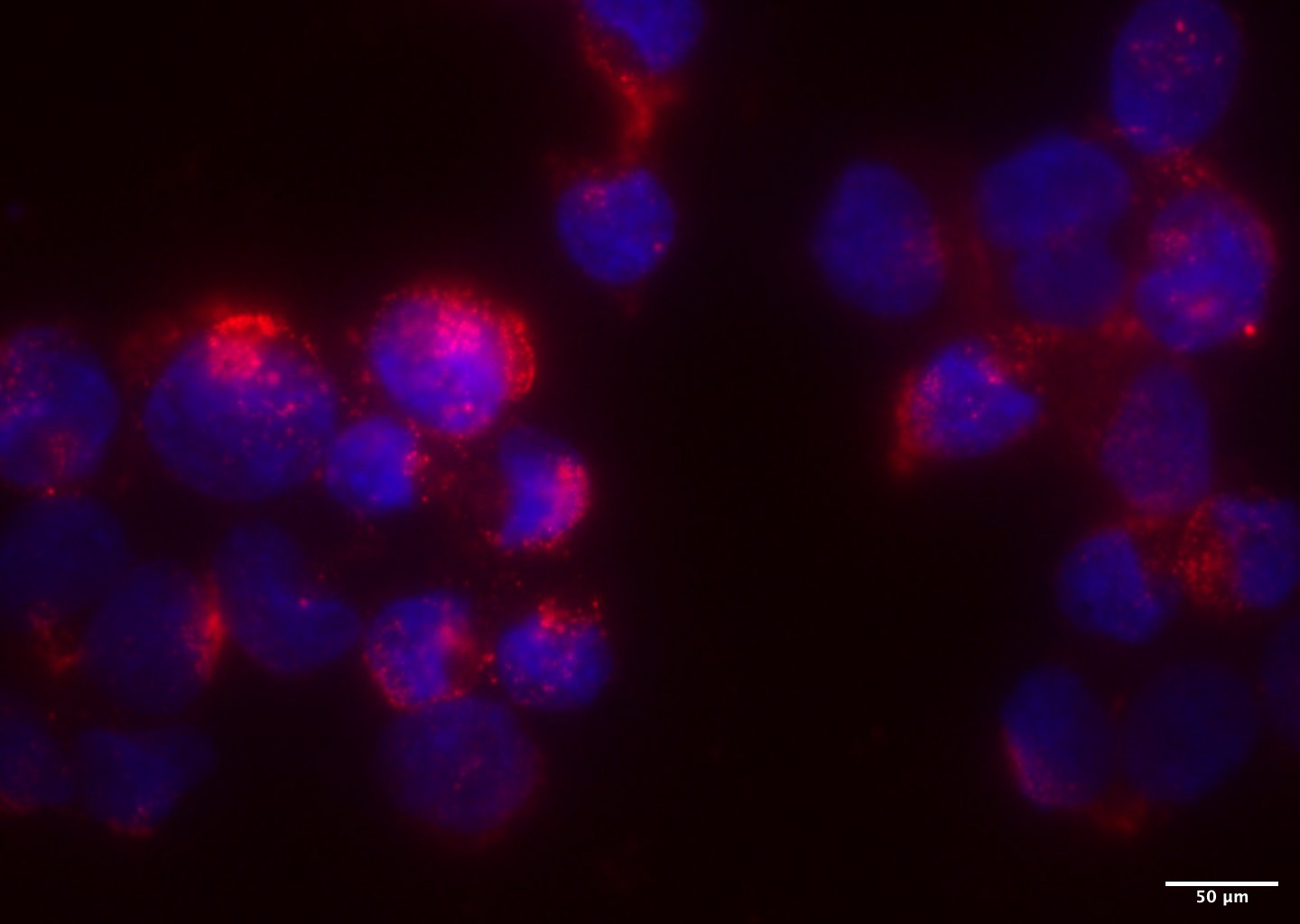

Supplement: Supplementary file 2 — Source Data Fig. 1 [file 44318_2024_35_MOESM2_ESM.zip › EMBOJ-2023-115792R2_SourceData_Fig1/Fig1C/R1/WT SINV 2A-GFP/WT 2A-GFP merge.jpg]

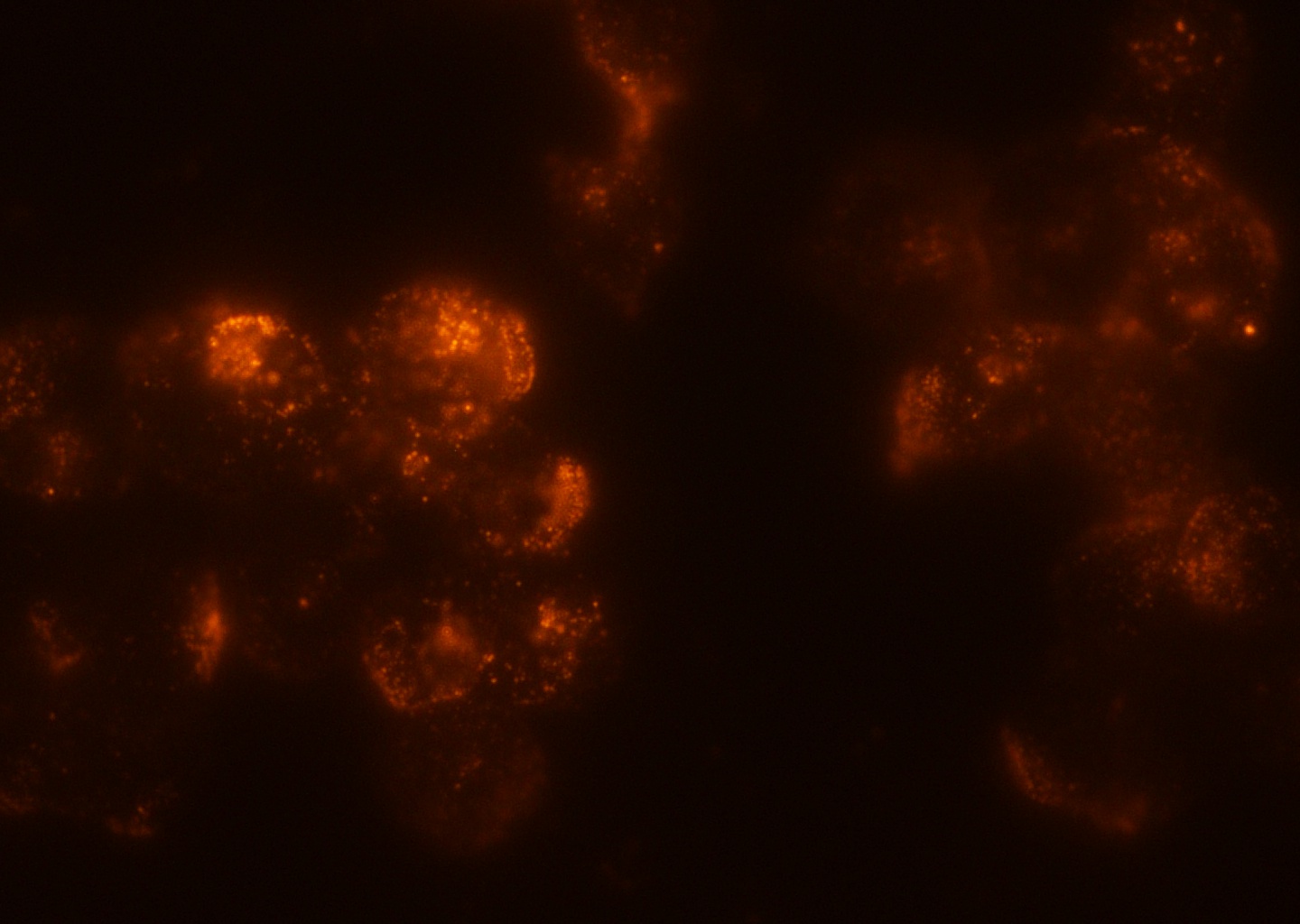

Supplement: Supplementary file 2 — Source Data Fig. 1 [file 44318_2024_35_MOESM2_ESM.zip › EMBOJ-2023-115792R2_SourceData_Fig1/Fig1C/R1/WT SINV 2A-GFP/J2.jpg]

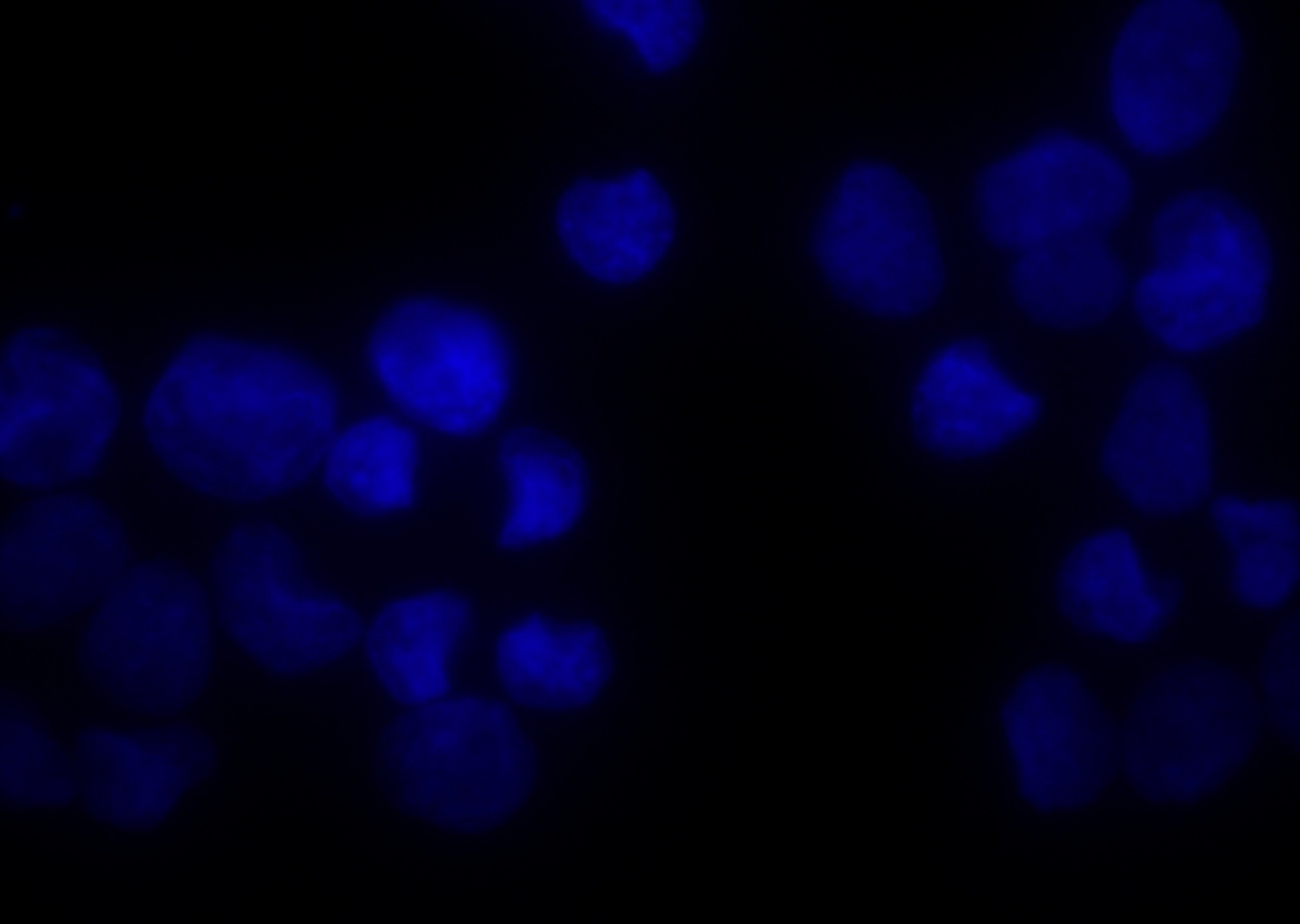

Supplement: Supplementary file 2 — Source Data Fig. 1 [file 44318_2024_35_MOESM2_ESM.zip › EMBOJ-2023-115792R2_SourceData_Fig1/Fig1C/R1/WT SINV 2A-GFP/DAPI.jpg]

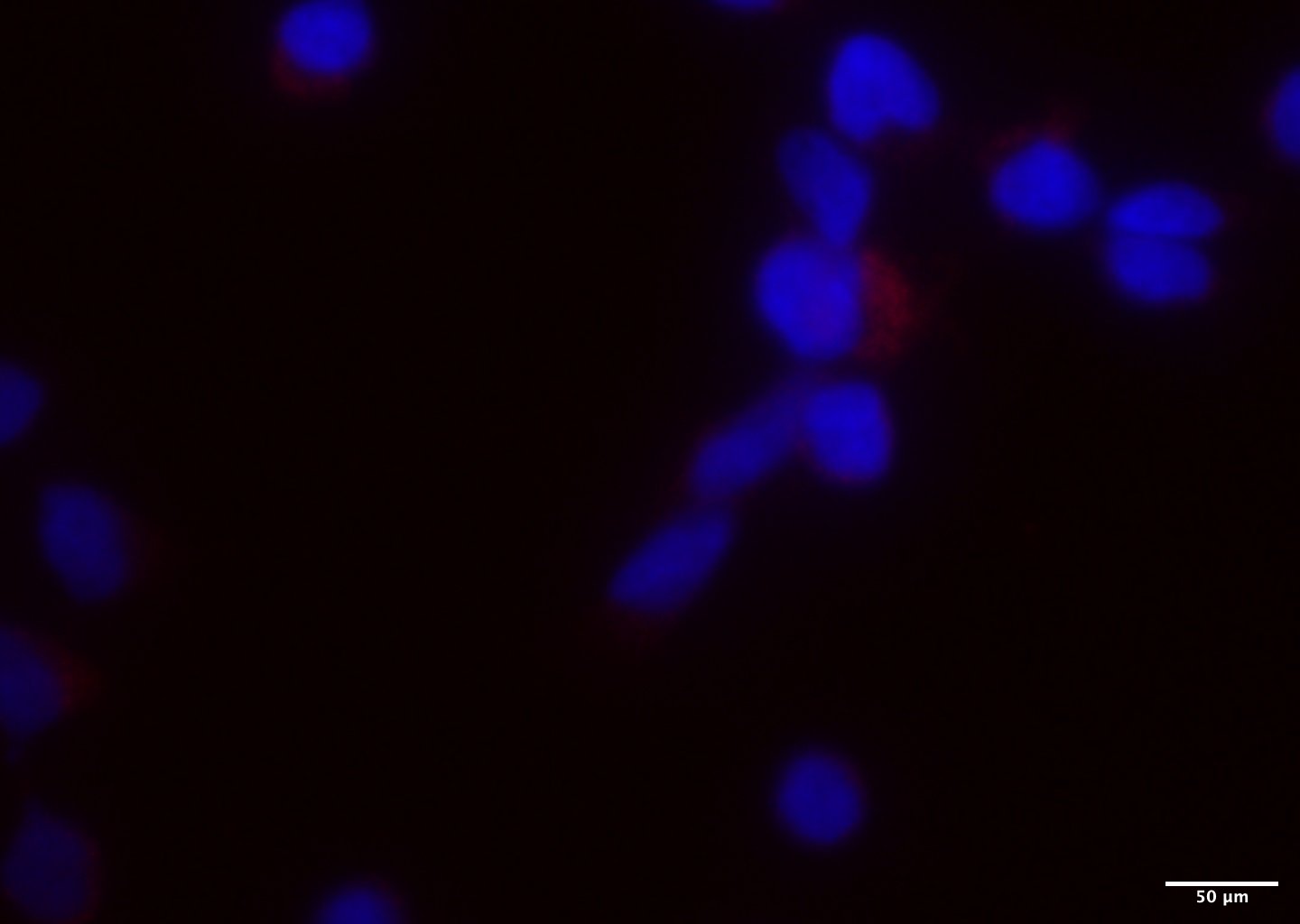

Supplement: Supplementary file 2 — Source Data Fig. 1 [file 44318_2024_35_MOESM2_ESM.zip › EMBOJ-2023-115792R2_SourceData_Fig1/Fig1C/R1/WT Mock/WT MOCK merge.jpg]

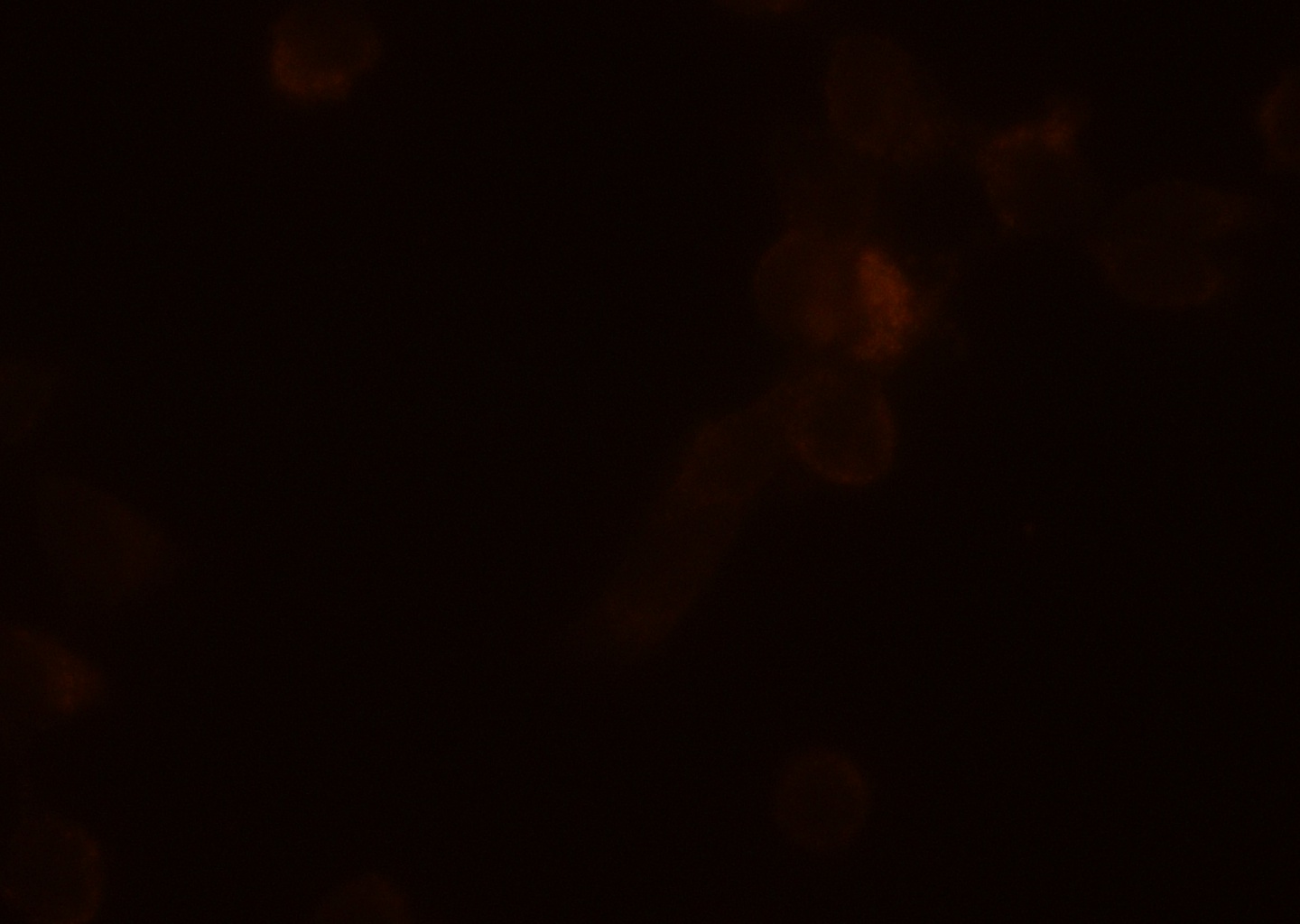

Supplement: Supplementary file 2 — Source Data Fig. 1 [file 44318_2024_35_MOESM2_ESM.zip › EMBOJ-2023-115792R2_SourceData_Fig1/Fig1C/R1/WT Mock/J2.jpg]

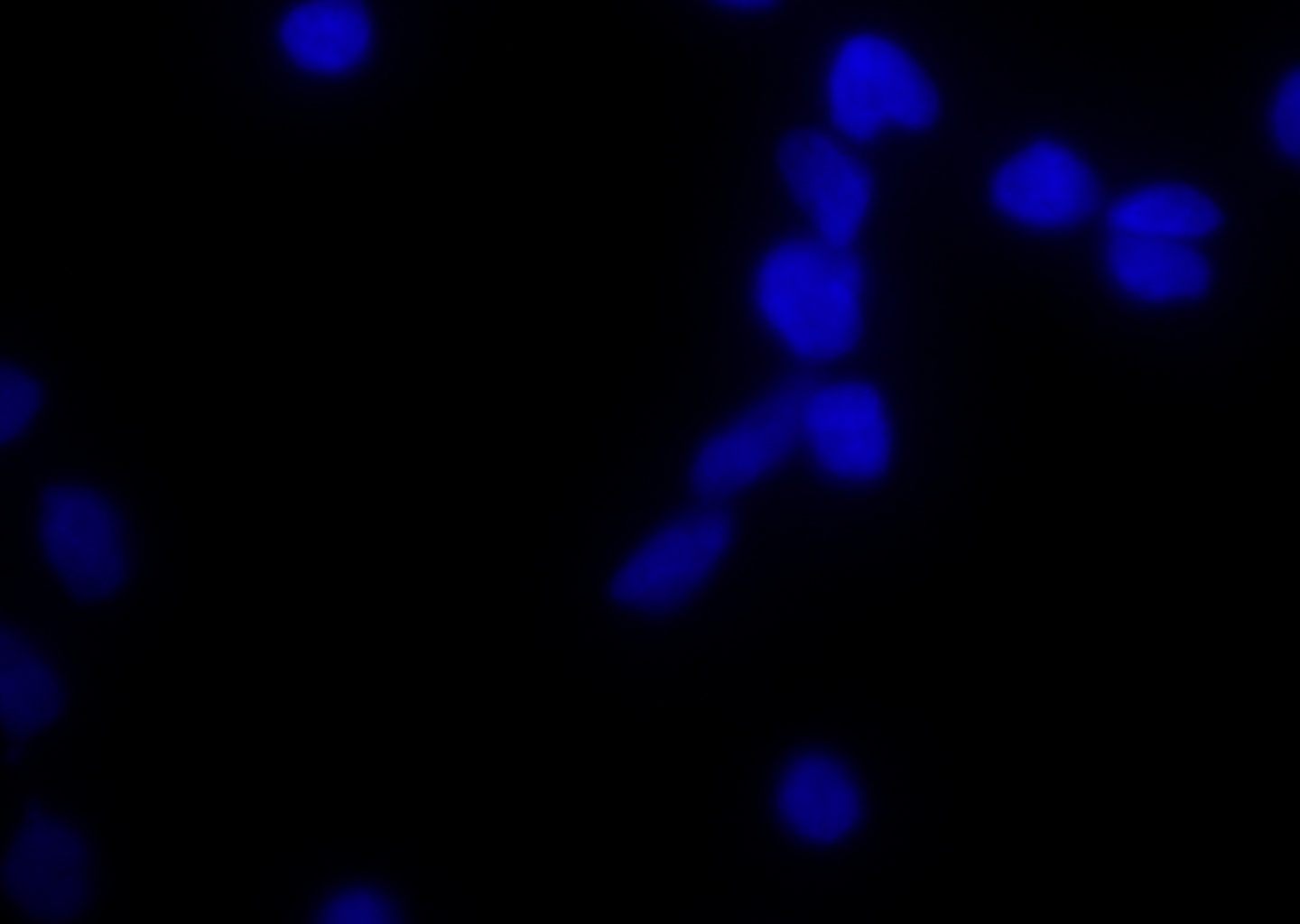

Supplement: Supplementary file 2 — Source Data Fig. 1 [file 44318_2024_35_MOESM2_ESM.zip › EMBOJ-2023-115792R2_SourceData_Fig1/Fig1C/R1/WT Mock/DAPI.jpg]

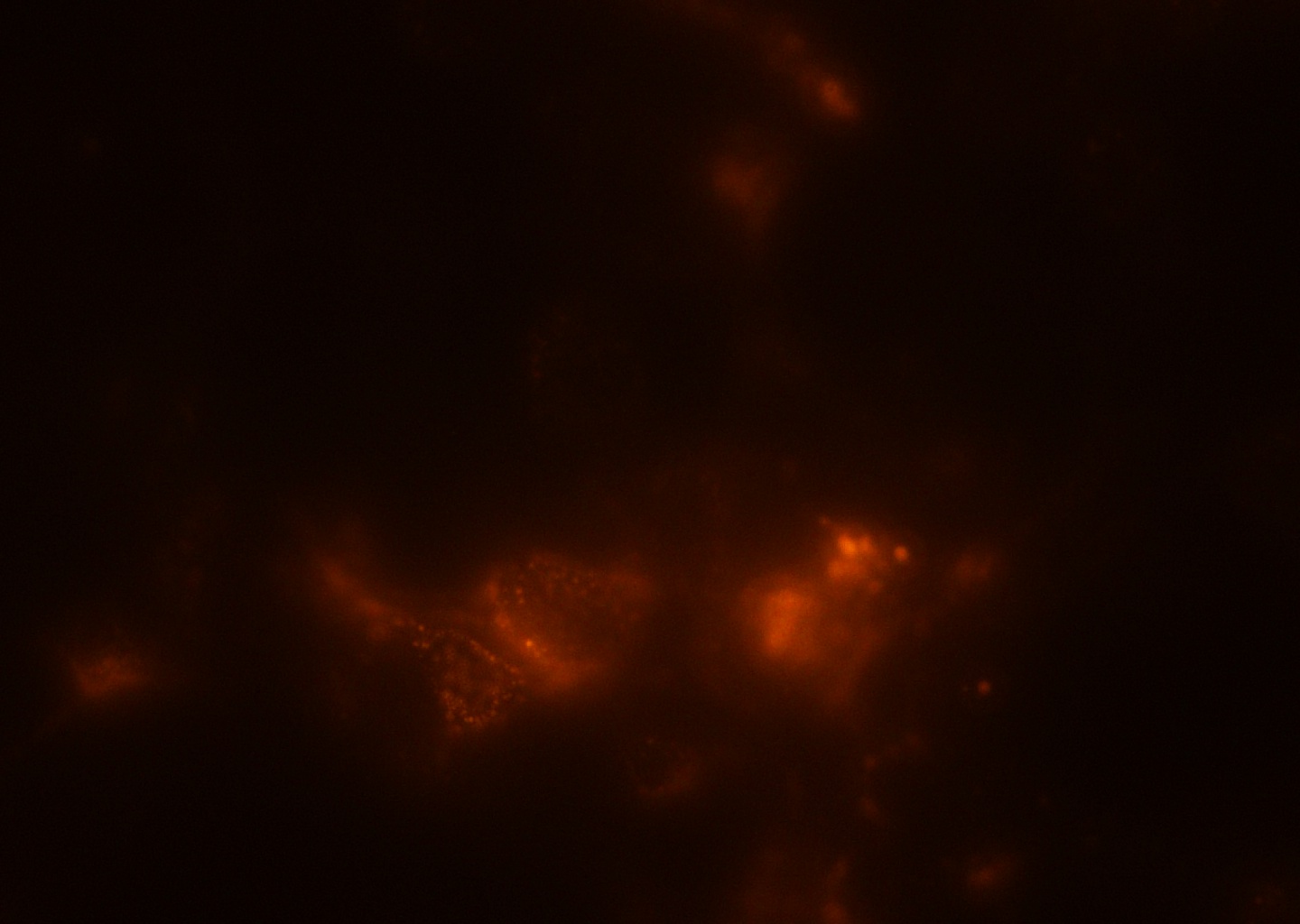

Supplement: Supplementary file 2 — Source Data Fig. 1 [file 44318_2024_35_MOESM2_ESM.zip › EMBOJ-2023-115792R2_SourceData_Fig1/Fig1C/R1/N1 SINV-GFP/J2.jpg]

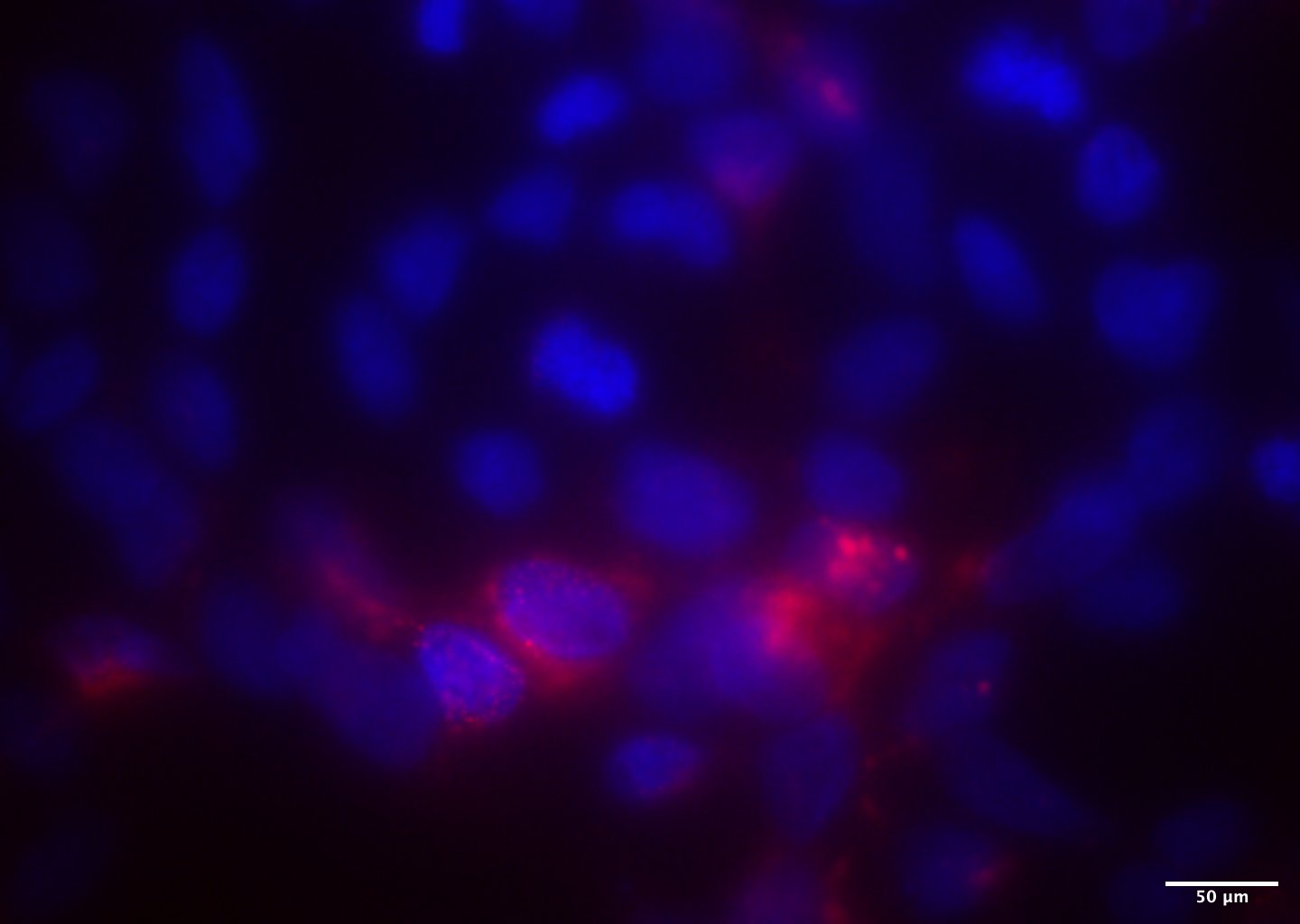

Supplement: Supplementary file 2 — Source Data Fig. 1 [file 44318_2024_35_MOESM2_ESM.zip › EMBOJ-2023-115792R2_SourceData_Fig1/Fig1C/R1/N1 SINV-GFP/N1 SINV GFP merge.jpg]

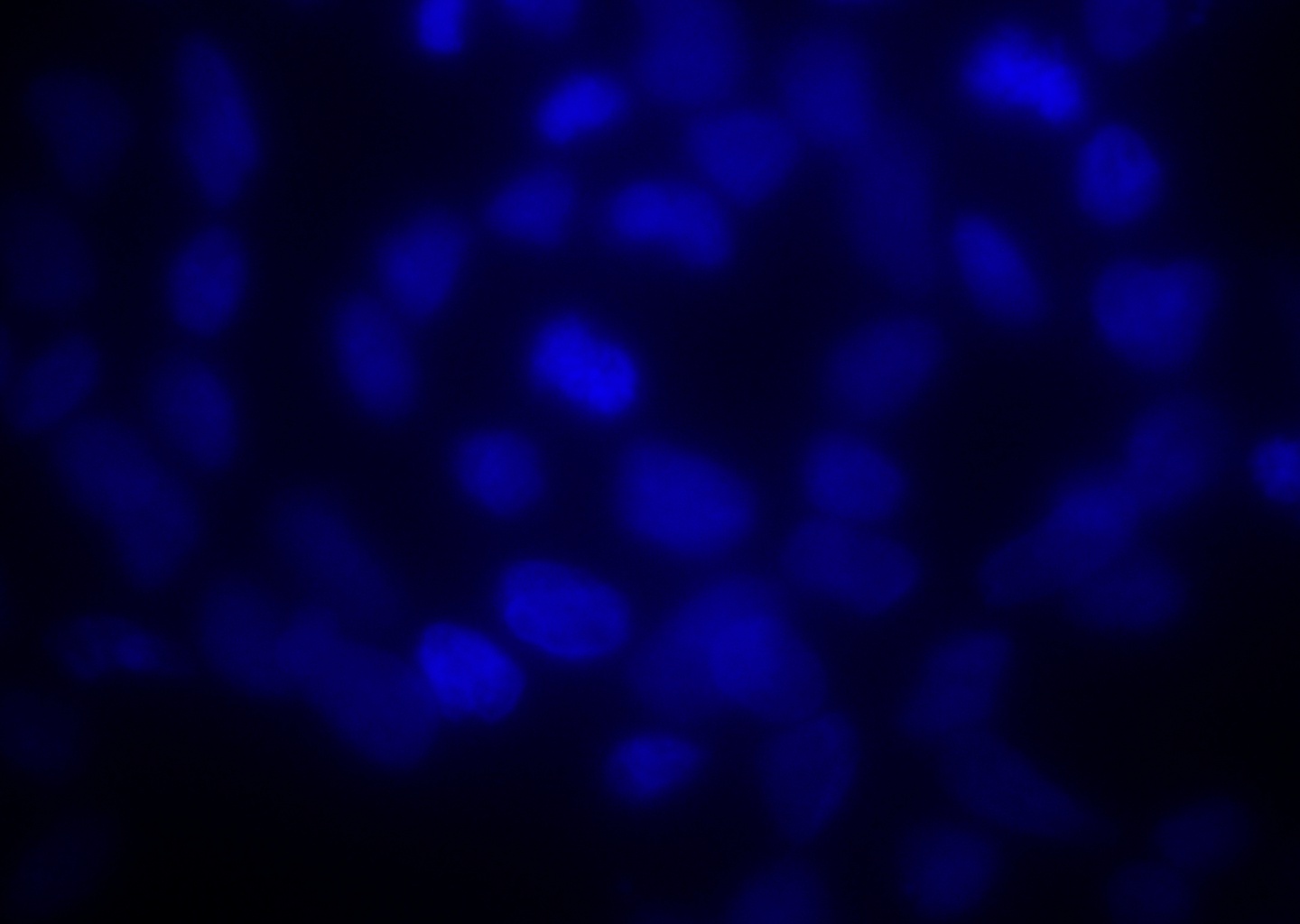

Supplement: Supplementary file 2 — Source Data Fig. 1 [file 44318_2024_35_MOESM2_ESM.zip › EMBOJ-2023-115792R2_SourceData_Fig1/Fig1C/R1/N1 SINV-GFP/DAPI.jpg]

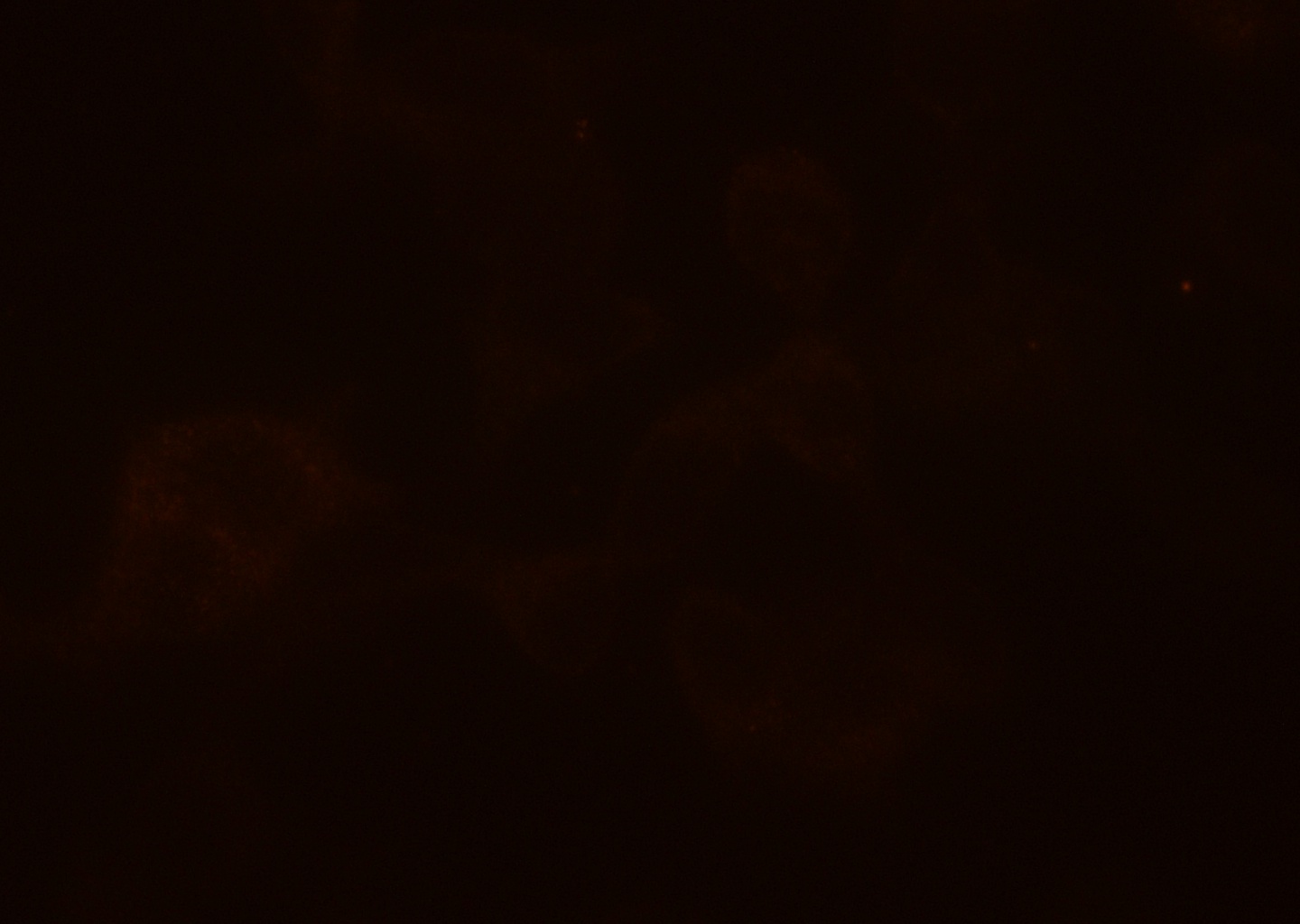

Supplement: Supplementary file 2 — Source Data Fig. 1 [file 44318_2024_35_MOESM2_ESM.zip › EMBOJ-2023-115792R2_SourceData_Fig1/Fig1C/R1/N1 Mock/J2.jpg]

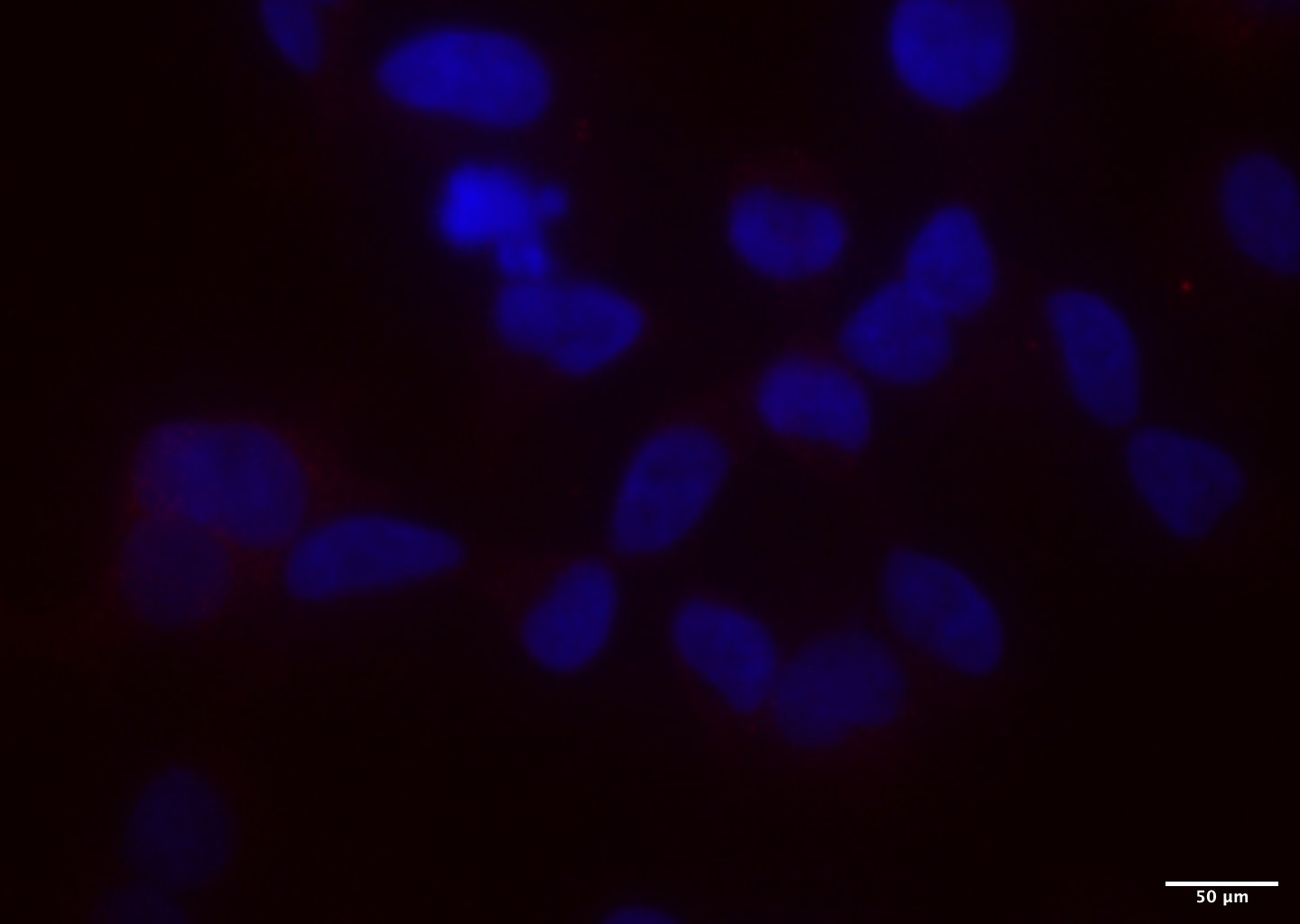

Supplement: Supplementary file 2 — Source Data Fig. 1 [file 44318_2024_35_MOESM2_ESM.zip › EMBOJ-2023-115792R2_SourceData_Fig1/Fig1C/R1/N1 Mock/N1 MOCK merge.jpg]

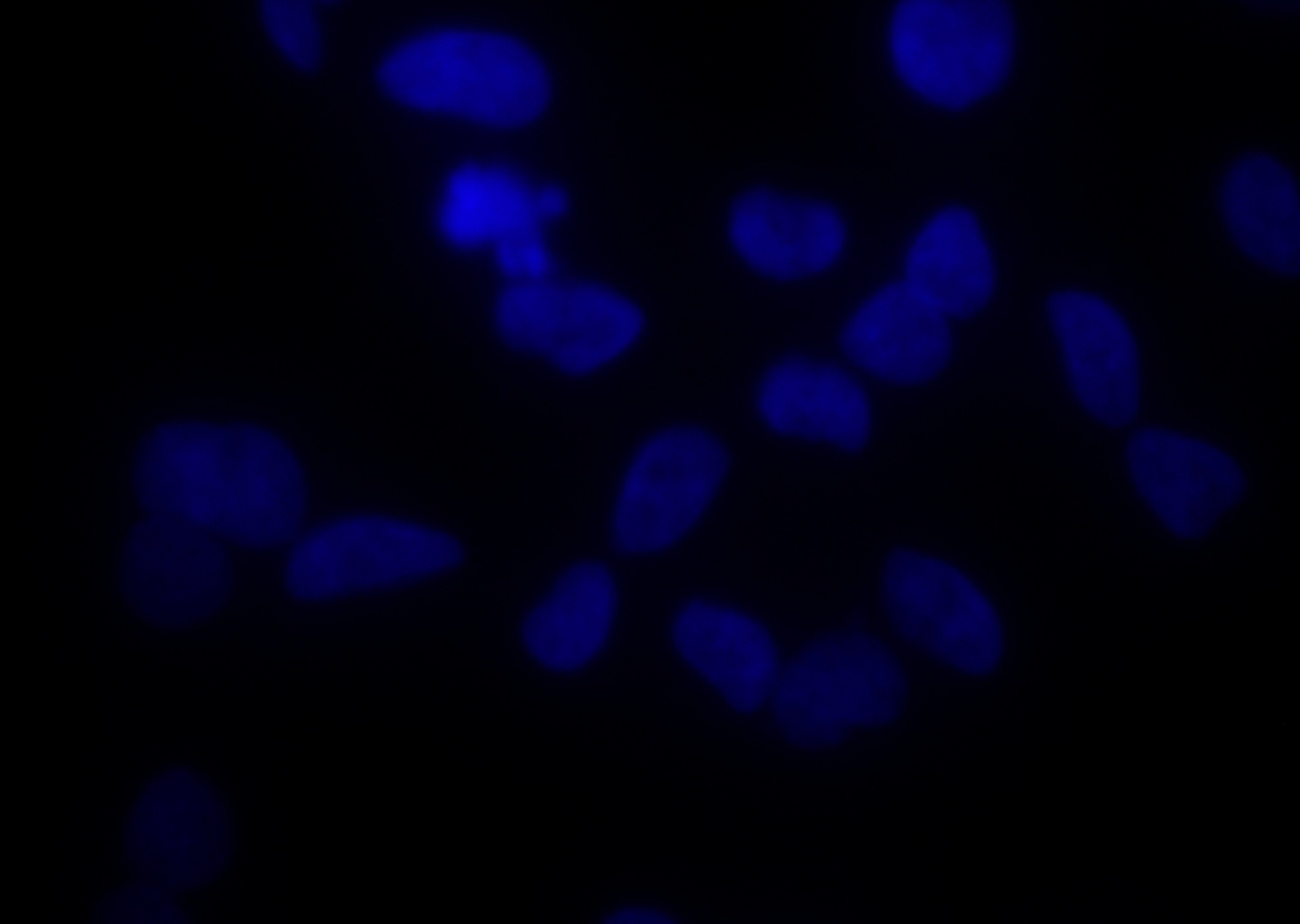

Supplement: Supplementary file 2 — Source Data Fig. 1 [file 44318_2024_35_MOESM2_ESM.zip › EMBOJ-2023-115792R2_SourceData_Fig1/Fig1C/R1/N1 Mock/DAPI.jpg]

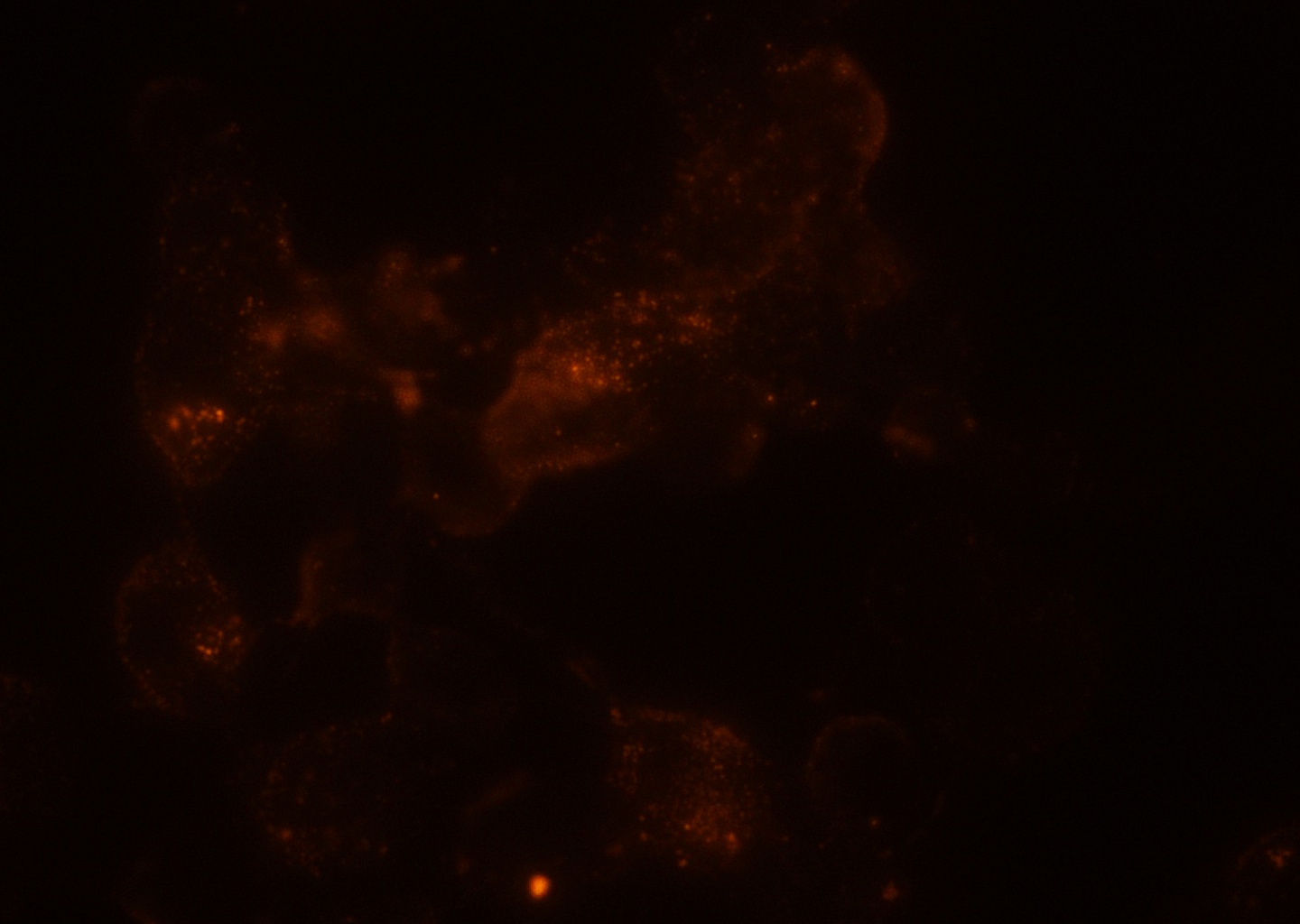

Supplement: Supplementary file 2 — Source Data Fig. 1 [file 44318_2024_35_MOESM2_ESM.zip › EMBOJ-2023-115792R2_SourceData_Fig1/Fig1C/R1/N1 SINV WT/J2.jpg]

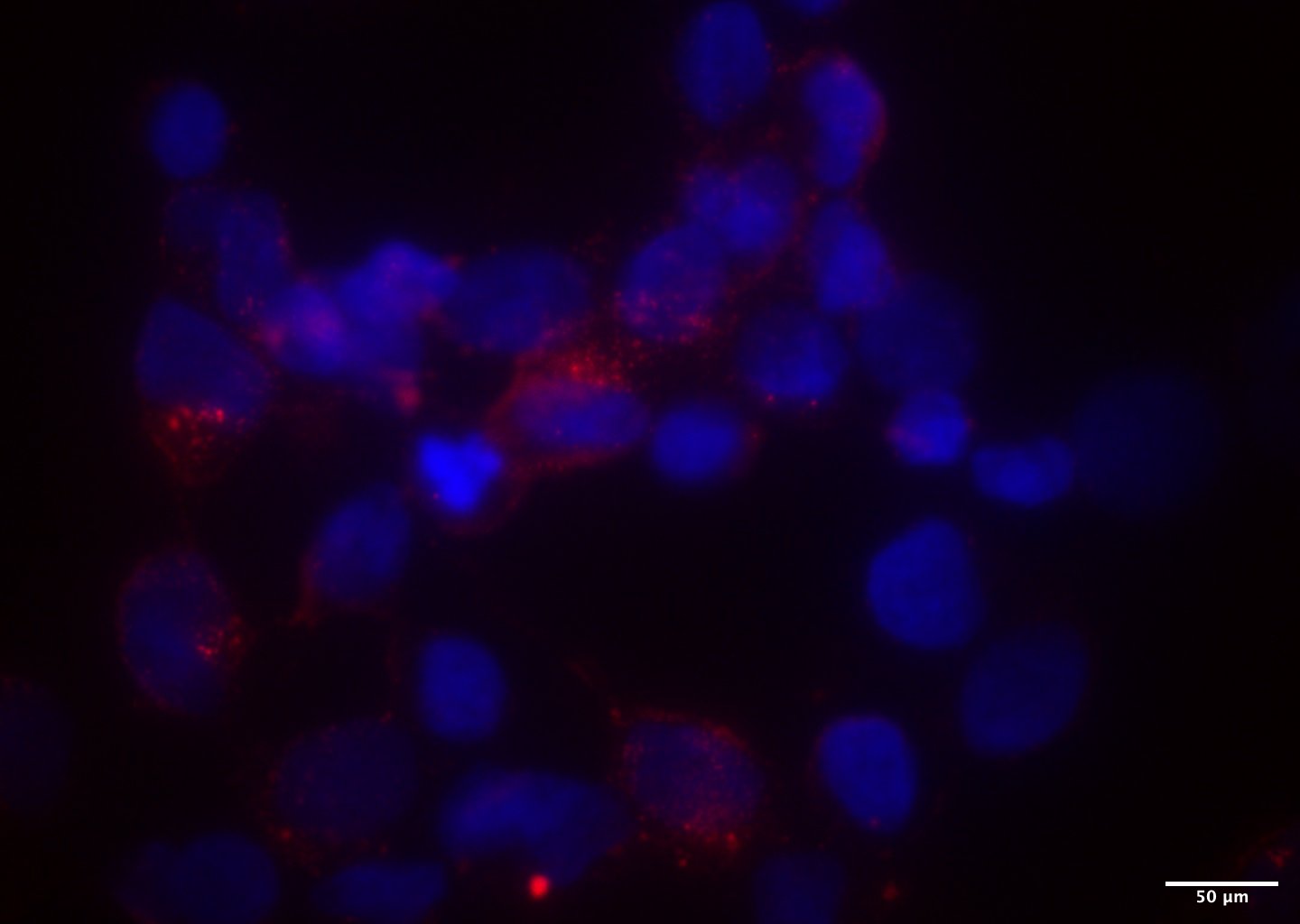

Supplement: Supplementary file 2 — Source Data Fig. 1 [file 44318_2024_35_MOESM2_ESM.zip › EMBOJ-2023-115792R2_SourceData_Fig1/Fig1C/R1/N1 SINV WT/N1 SINV WT merge.jpg]

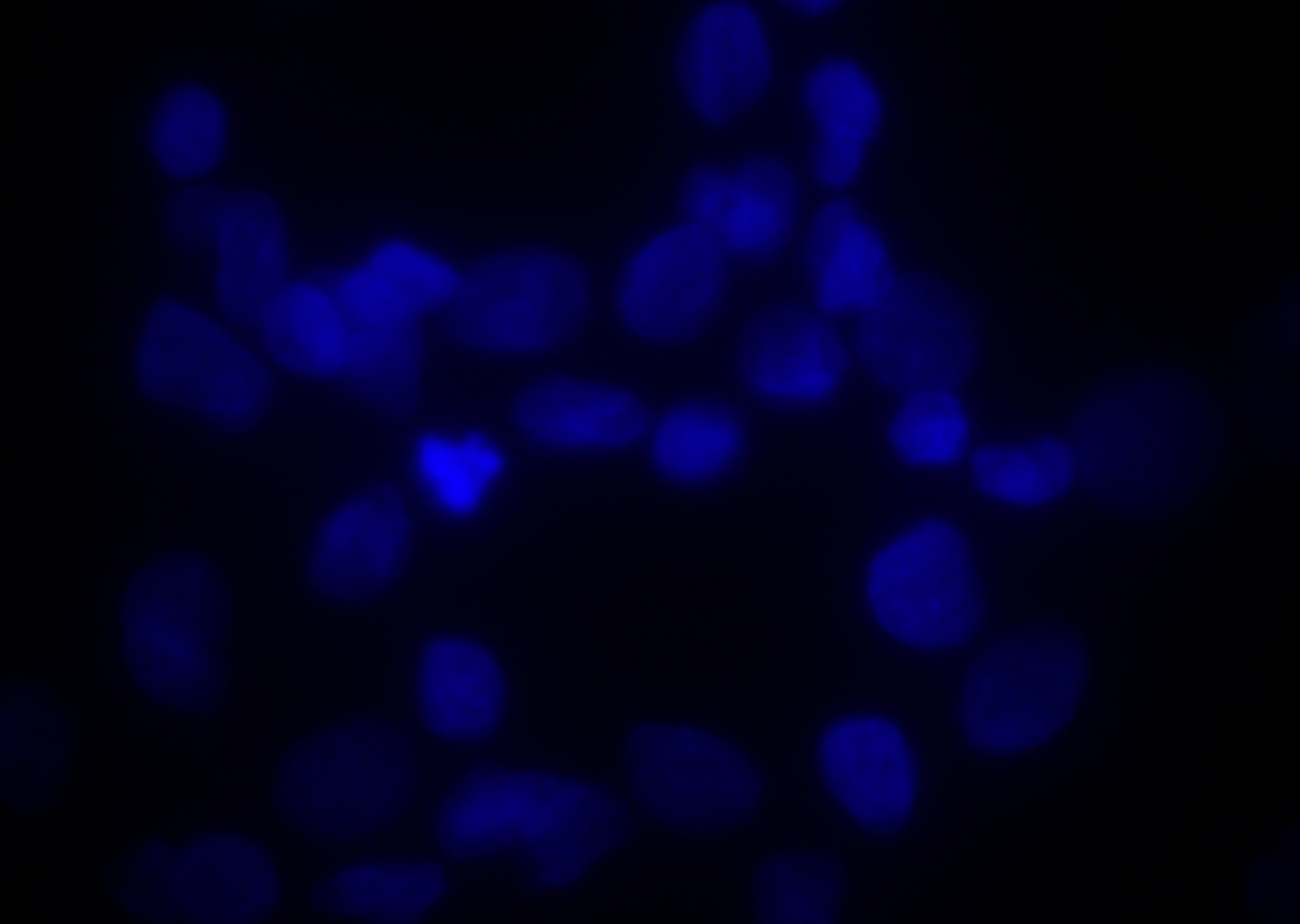

Supplement: Supplementary file 2 — Source Data Fig. 1 [file 44318_2024_35_MOESM2_ESM.zip › EMBOJ-2023-115792R2_SourceData_Fig1/Fig1C/R1/N1 SINV WT/DAPI.jpg]

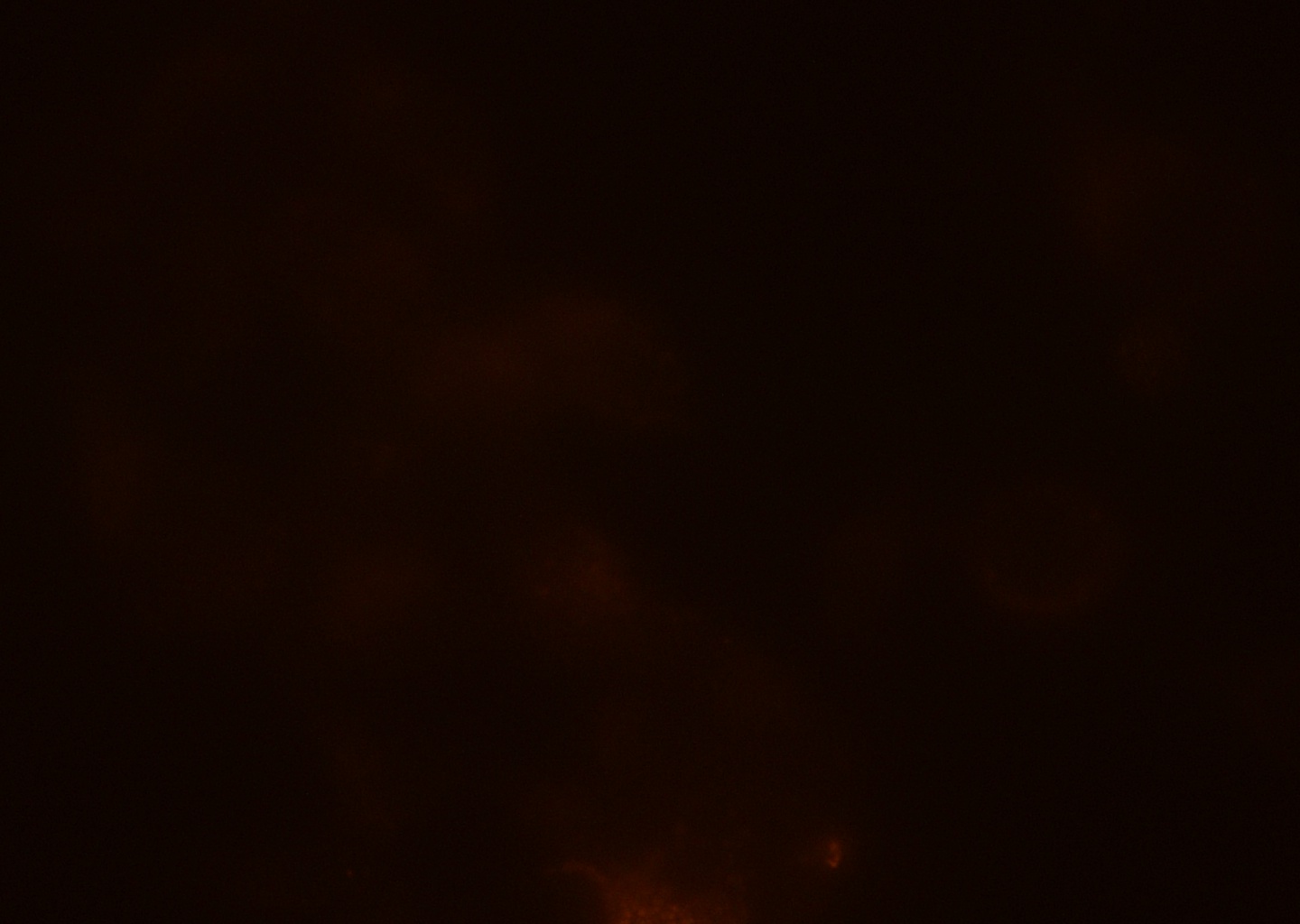

Supplement: Supplementary file 2 — Source Data Fig. 1 [file 44318_2024_35_MOESM2_ESM.zip › EMBOJ-2023-115792R2_SourceData_Fig1/Fig1C/R1/N1 SINV 2A-GFP/J2.jpg]

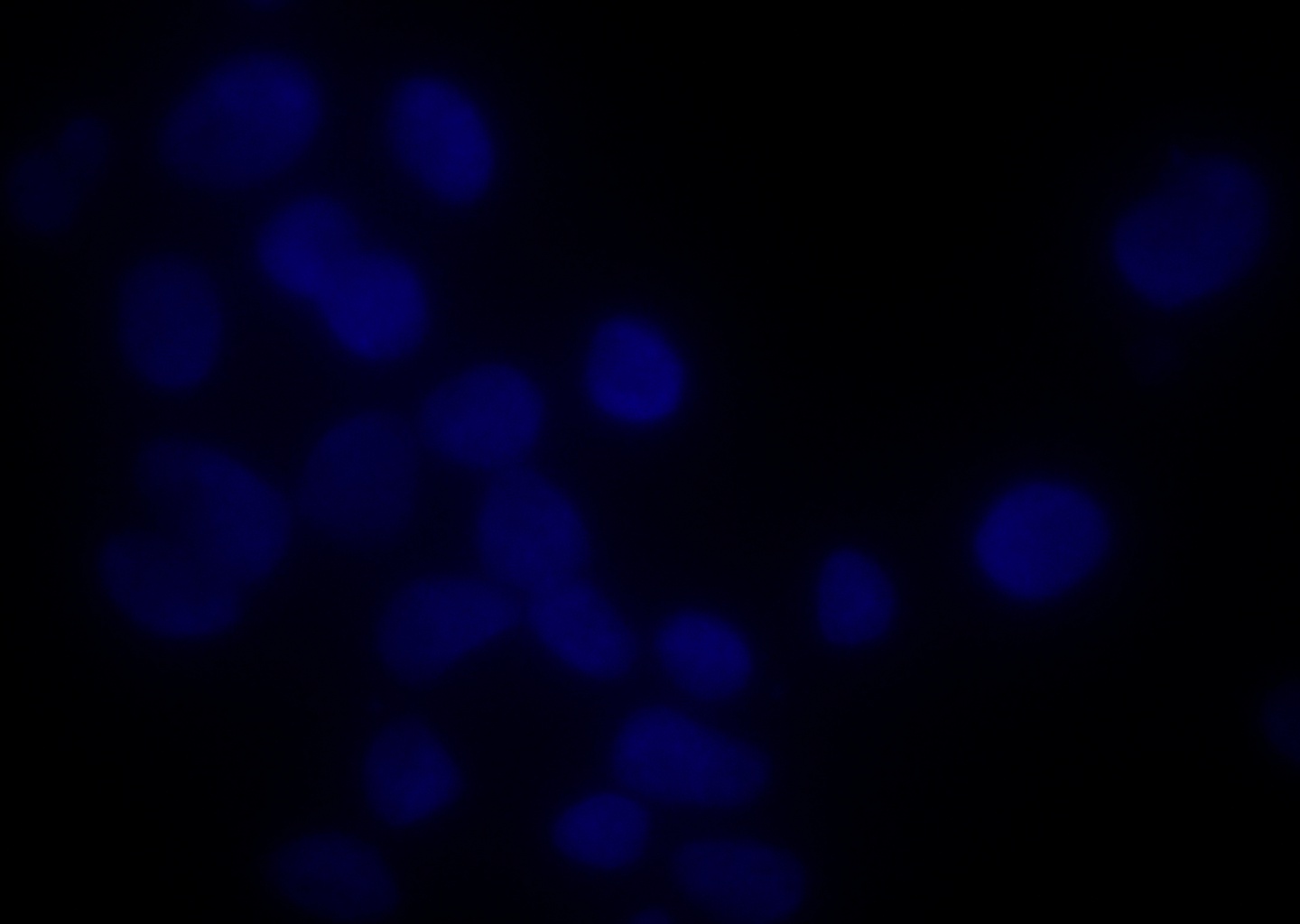

Supplement: Supplementary file 2 — Source Data Fig. 1 [file 44318_2024_35_MOESM2_ESM.zip › EMBOJ-2023-115792R2_SourceData_Fig1/Fig1C/R1/N1 SINV 2A-GFP/DAPI.jpg]

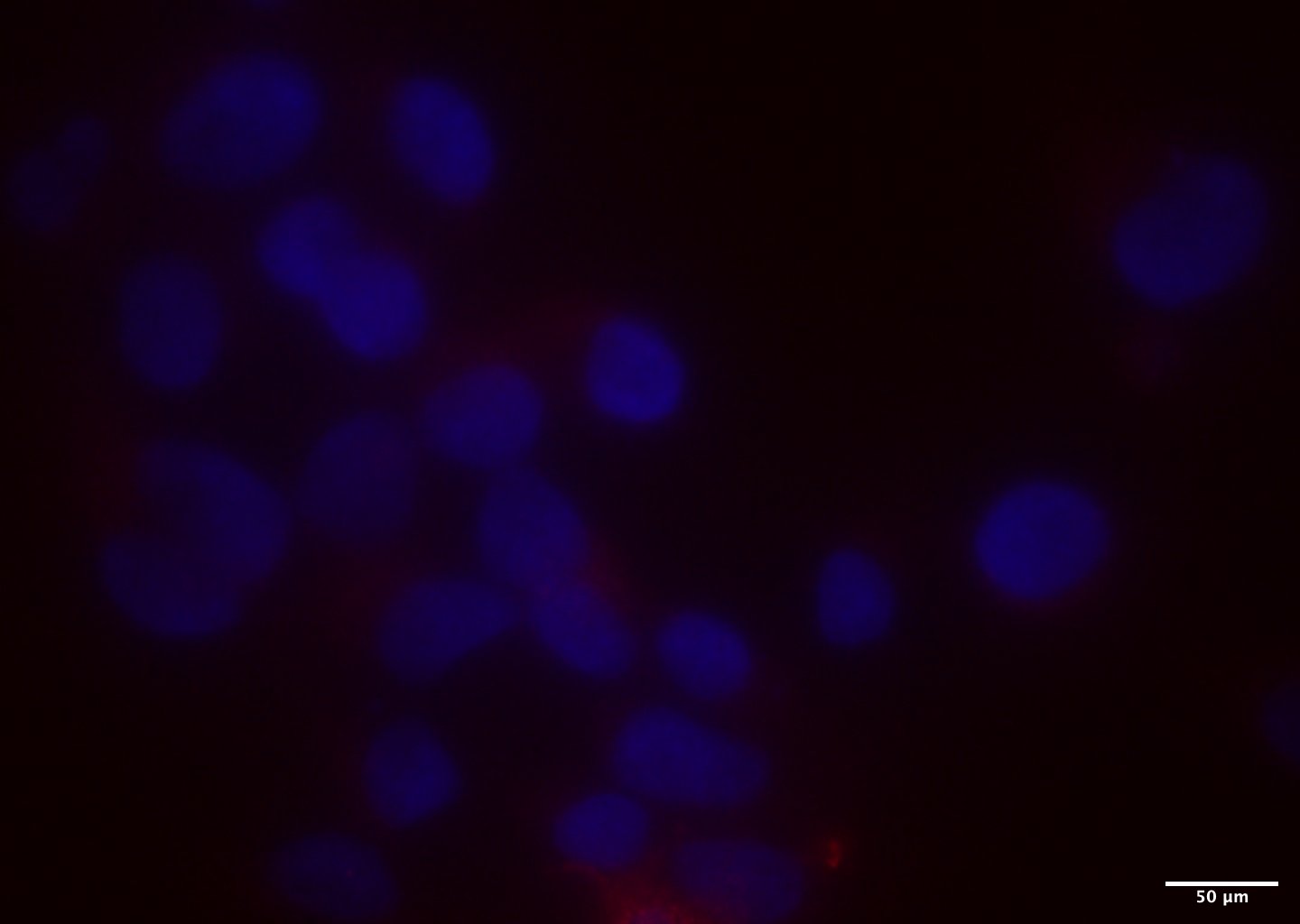

Supplement: Supplementary file 2 — Source Data Fig. 1 [file 44318_2024_35_MOESM2_ESM.zip › EMBOJ-2023-115792R2_SourceData_Fig1/Fig1C/R1/N1 SINV 2A-GFP/N1 2A-GFP merge.jpg]

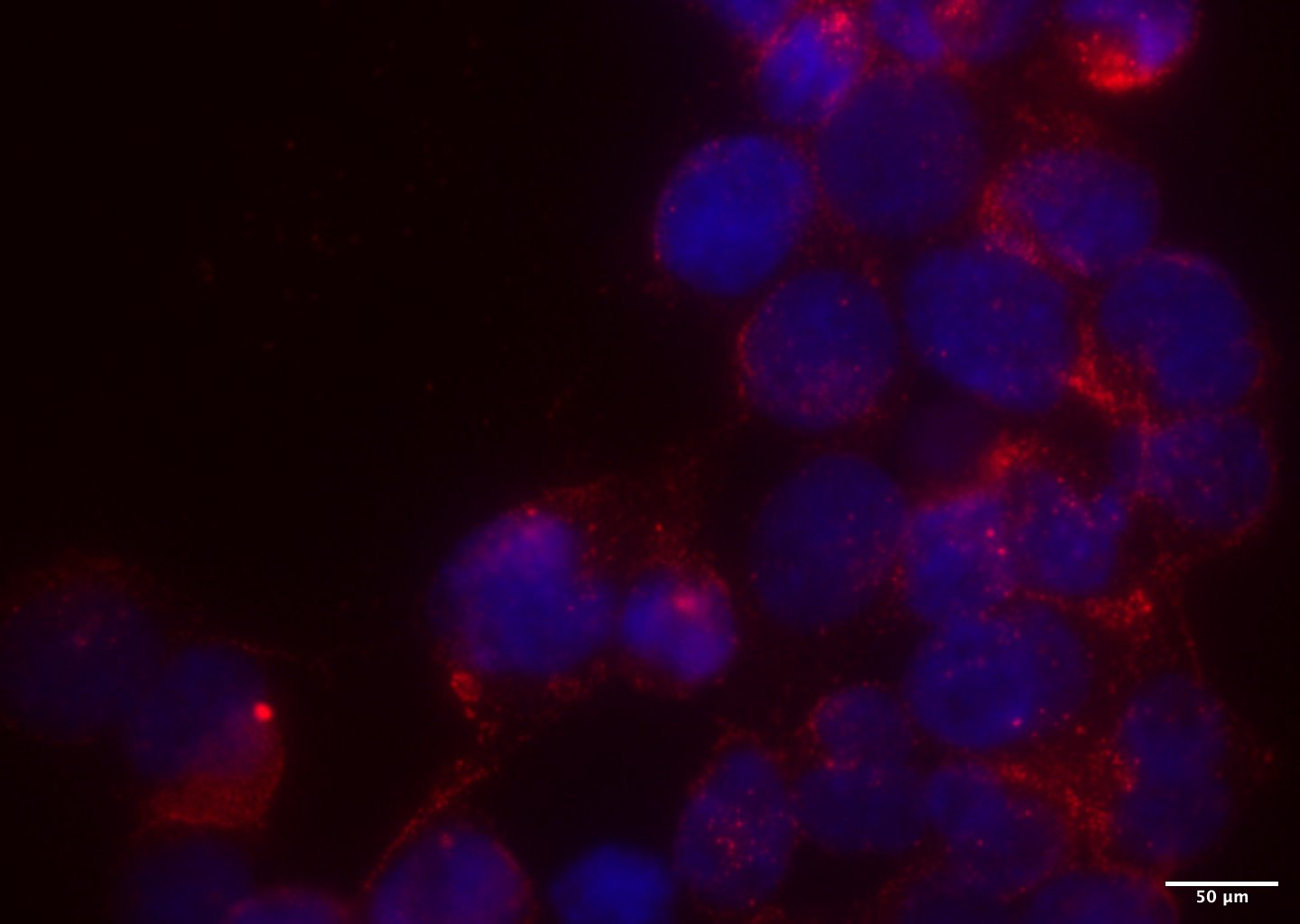

Supplement: Supplementary file 2 — Source Data Fig. 1 [file 44318_2024_35_MOESM2_ESM.zip › EMBOJ-2023-115792R2_SourceData_Fig1/Fig1C/R1/WT SINV-GFP/WT SINV-GFP merge.jpg]

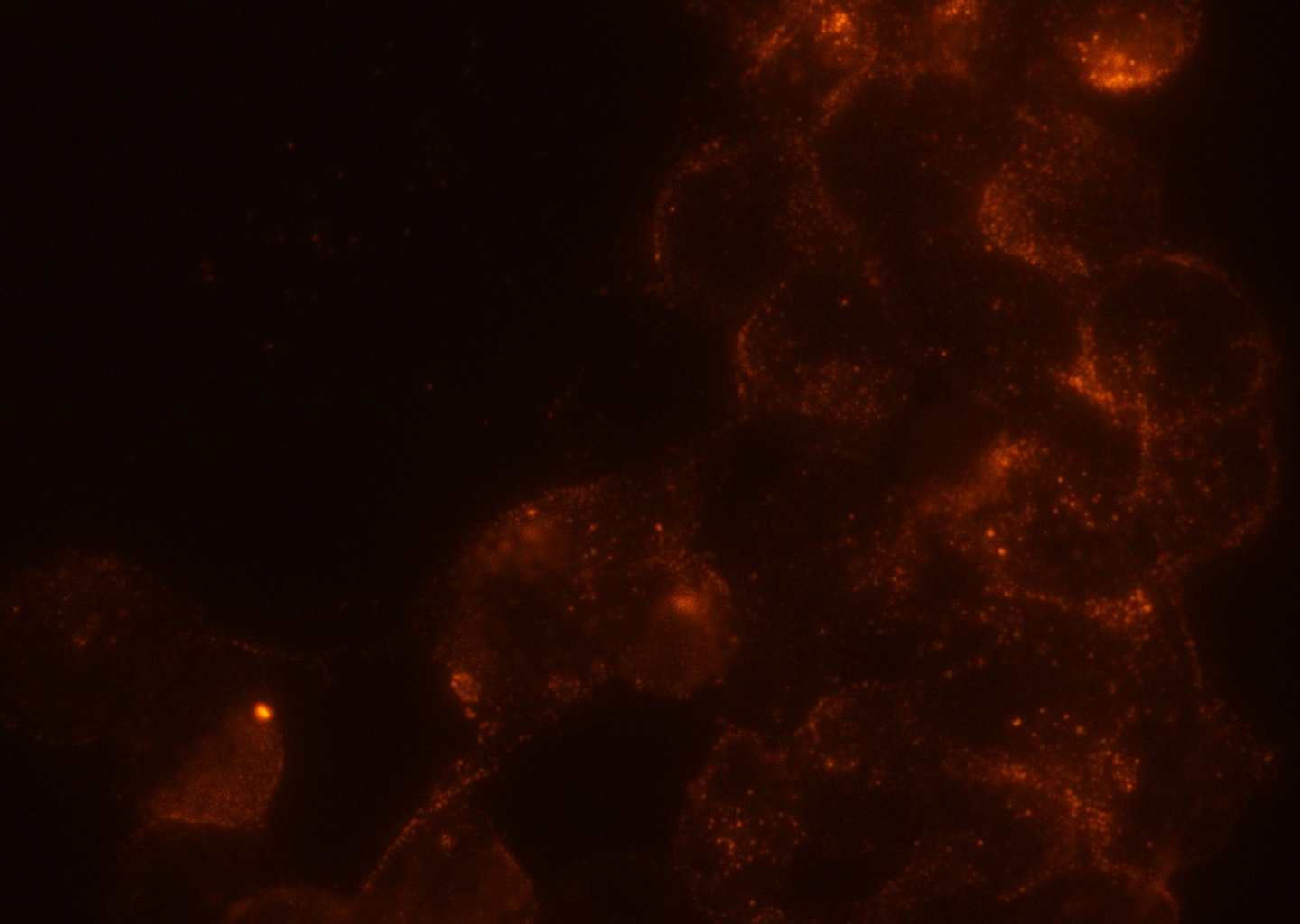

Supplement: Supplementary file 2 — Source Data Fig. 1 [file 44318_2024_35_MOESM2_ESM.zip › EMBOJ-2023-115792R2_SourceData_Fig1/Fig1C/R1/WT SINV-GFP/J2.jpg]

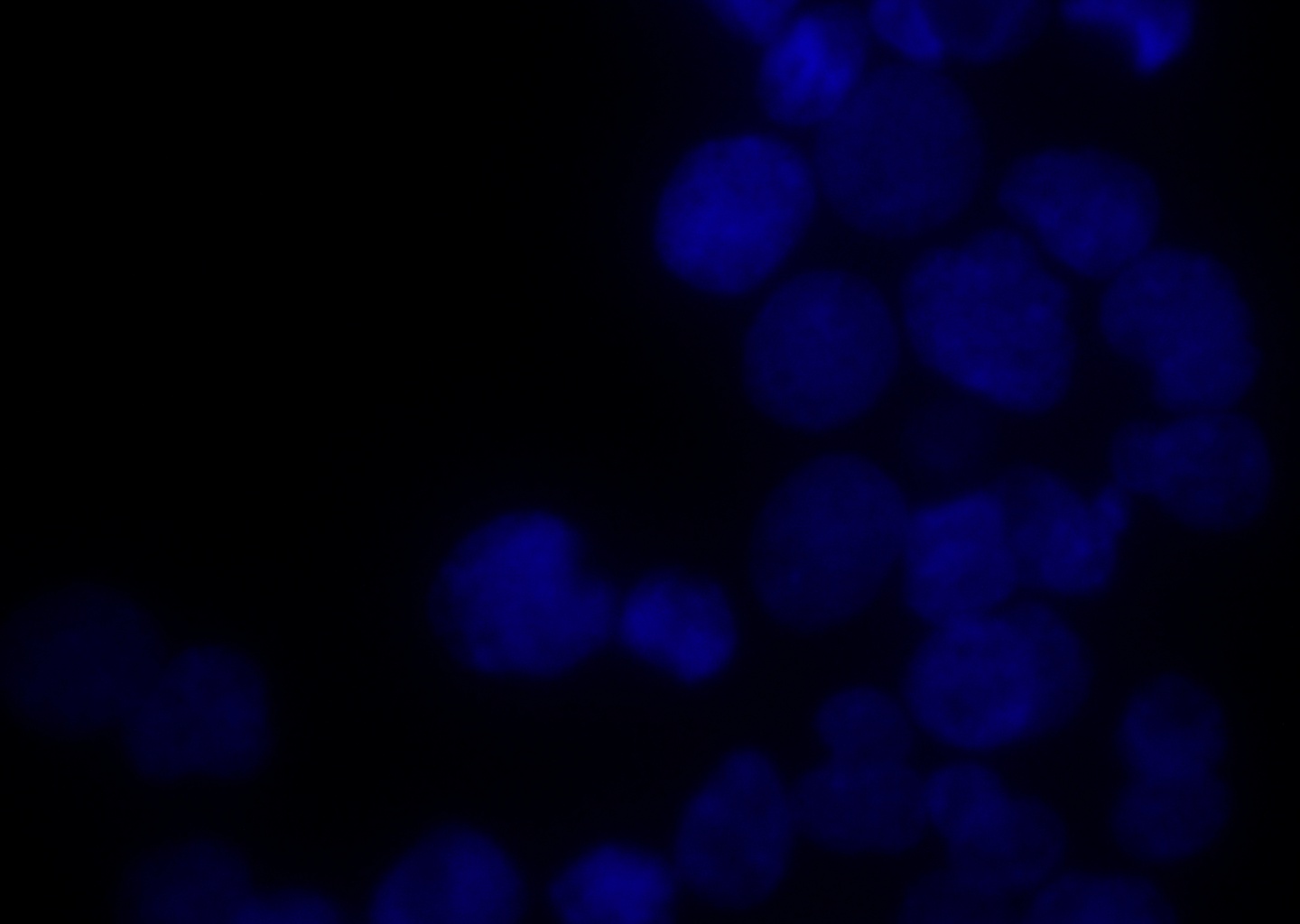

Supplement: Supplementary file 2 — Source Data Fig. 1 [file 44318_2024_35_MOESM2_ESM.zip › EMBOJ-2023-115792R2_SourceData_Fig1/Fig1C/R1/WT SINV-GFP/DAPI.jpg]

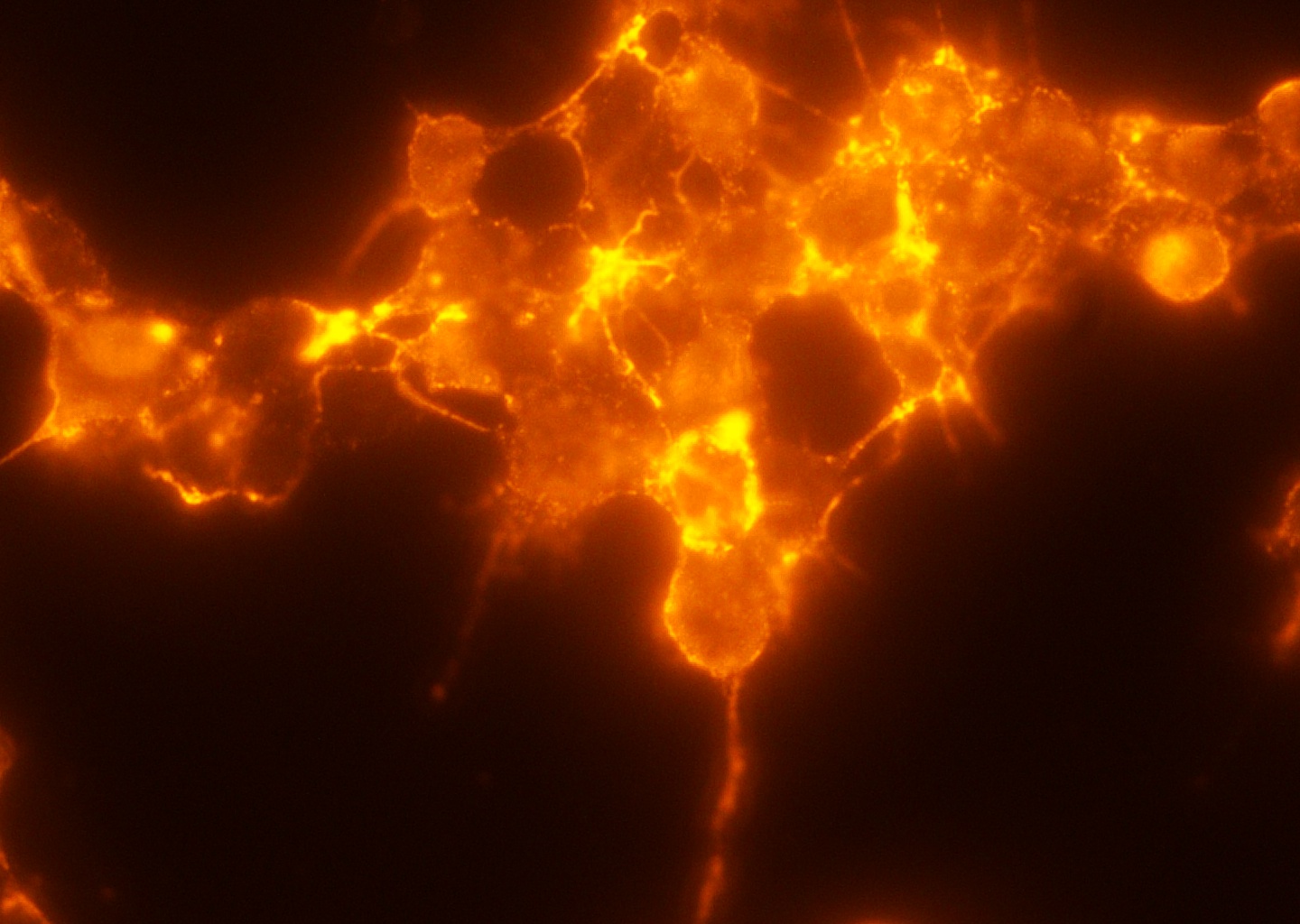

Supplement: Supplementary file 2 — Source Data Fig. 1 [file 44318_2024_35_MOESM2_ESM.zip › EMBOJ-2023-115792R2_SourceData_Fig1/Fig1C/R3/WT SINV WT/J2.jpg]

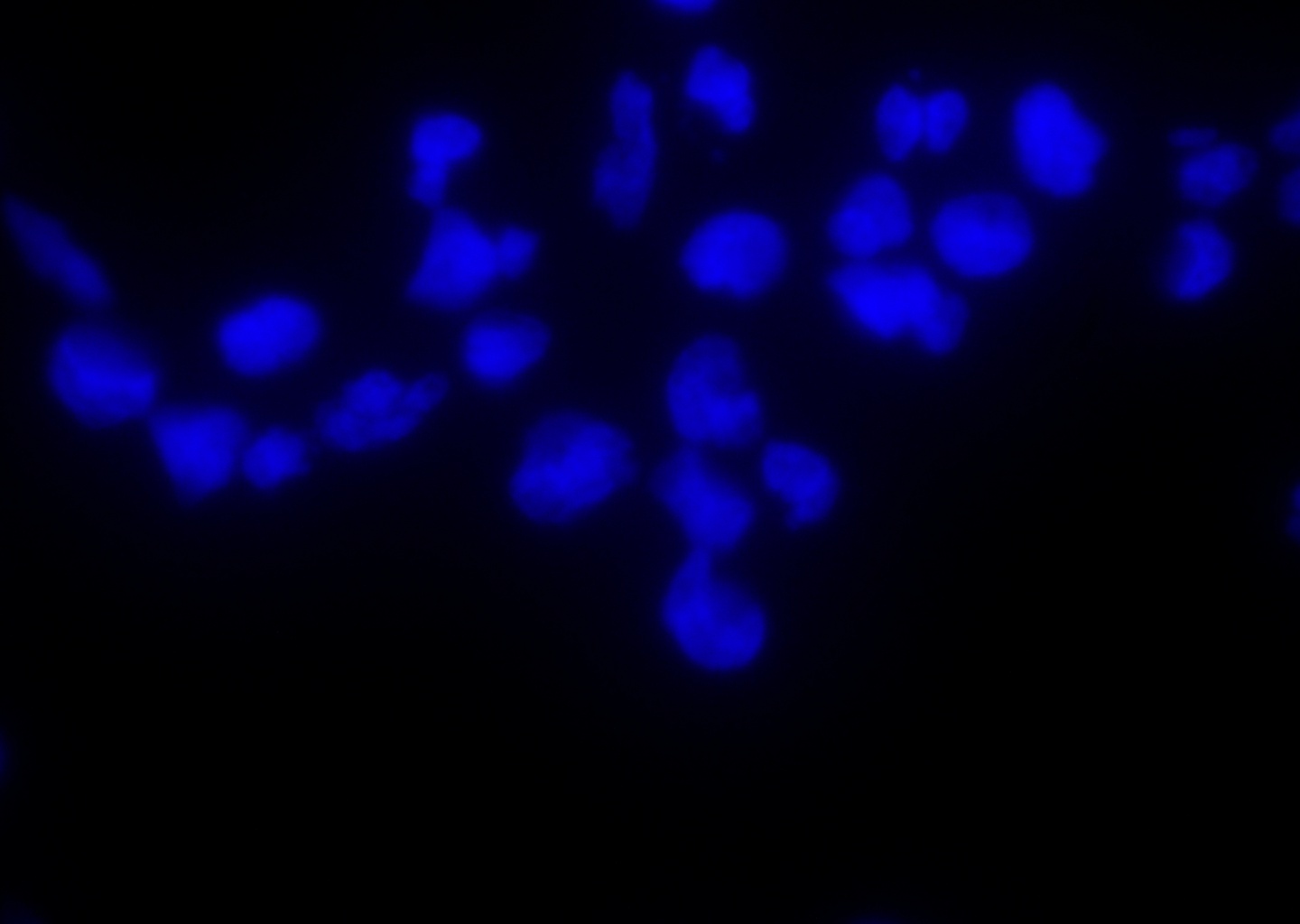

Supplement: Supplementary file 2 — Source Data Fig. 1 [file 44318_2024_35_MOESM2_ESM.zip › EMBOJ-2023-115792R2_SourceData_Fig1/Fig1C/R3/WT SINV WT/DAPI.jpg]

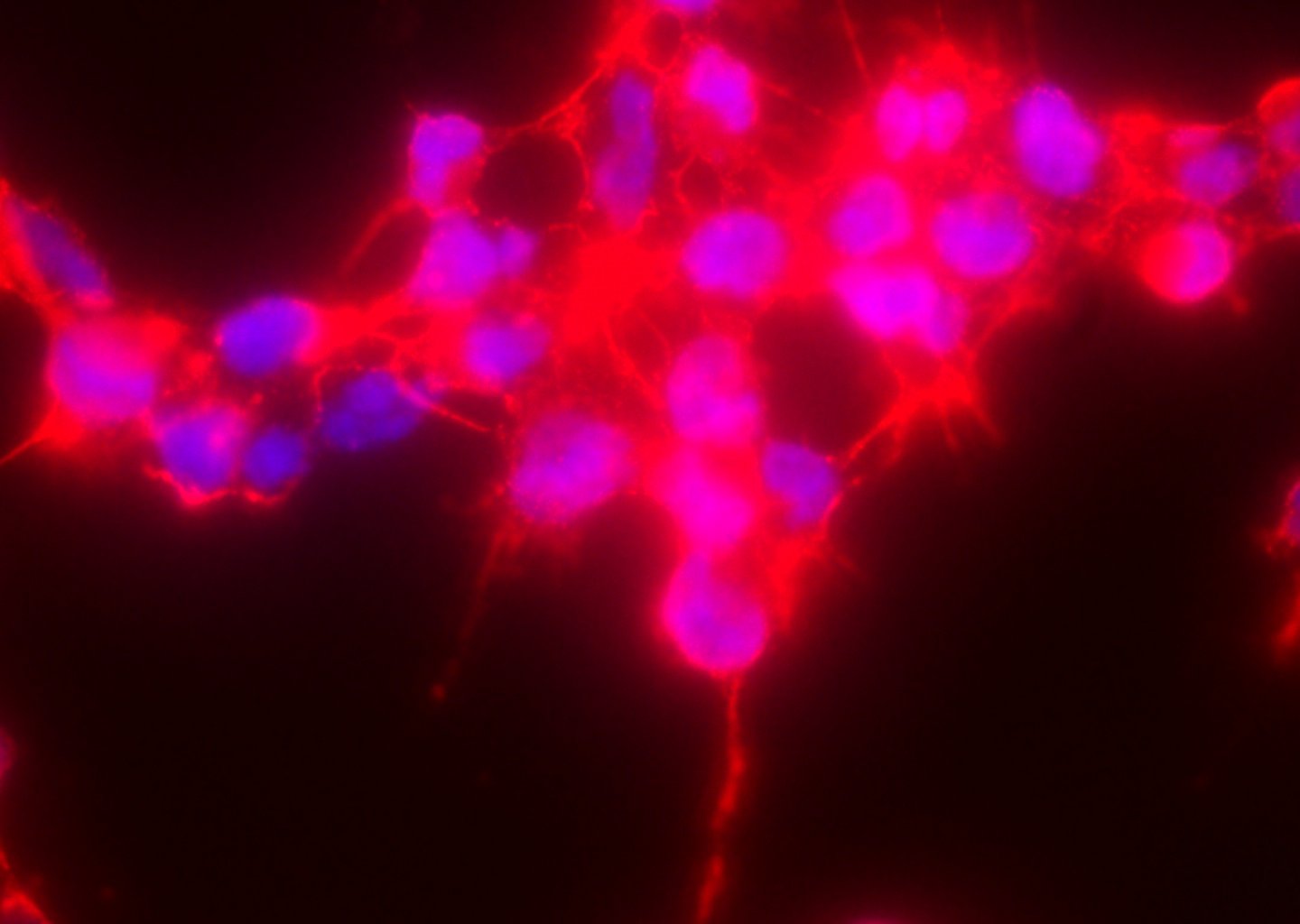

Supplement: Supplementary file 2 — Source Data Fig. 1 [file 44318_2024_35_MOESM2_ESM.zip › EMBOJ-2023-115792R2_SourceData_Fig1/Fig1C/R3/WT SINV WT/WT SINV WT merge.jpg]

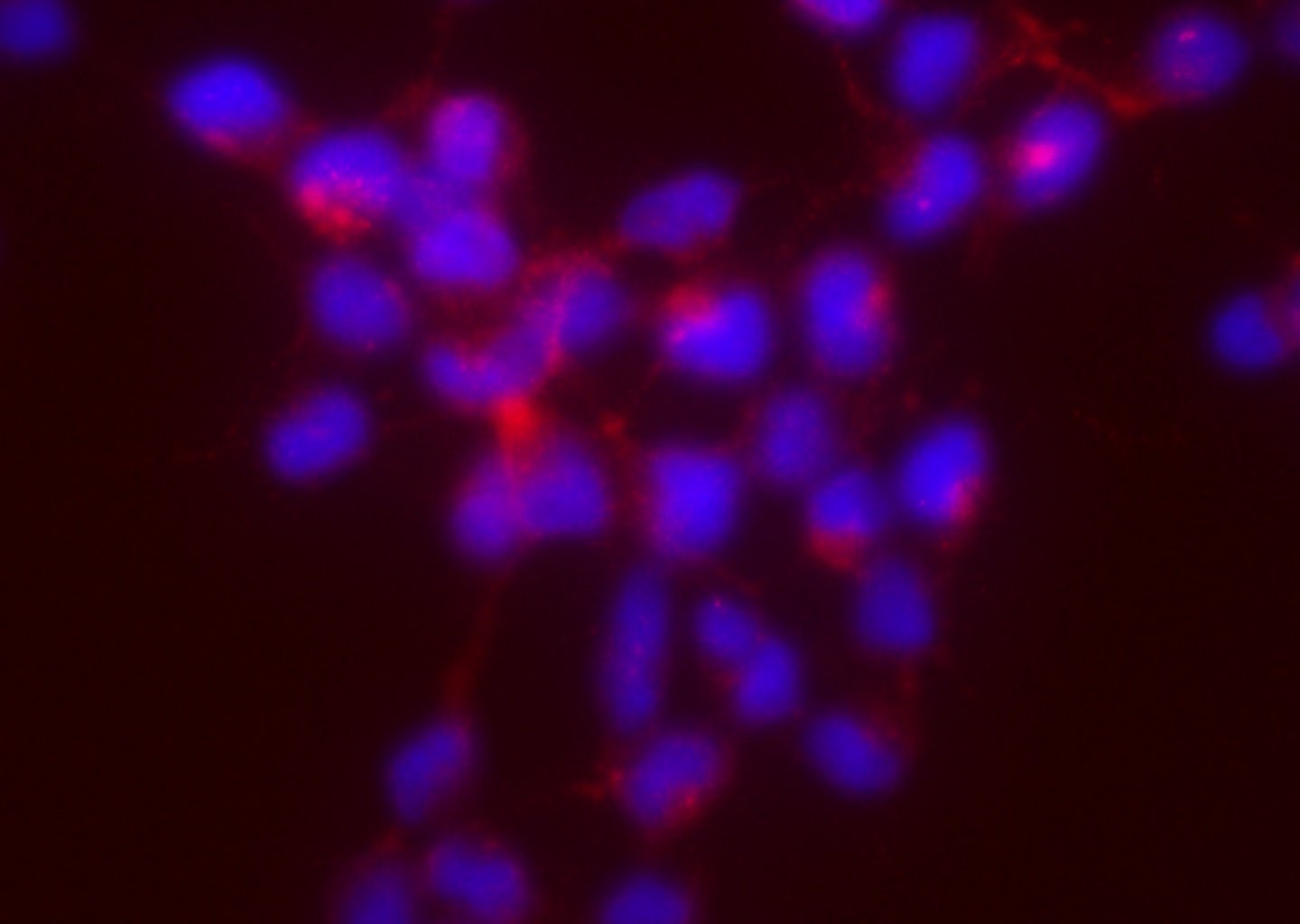

Supplement: Supplementary file 2 — Source Data Fig. 1 [file 44318_2024_35_MOESM2_ESM.zip › EMBOJ-2023-115792R2_SourceData_Fig1/Fig1C/R3/WT SINV 2A-GFP/WT 2A-GFP merge.jpg]

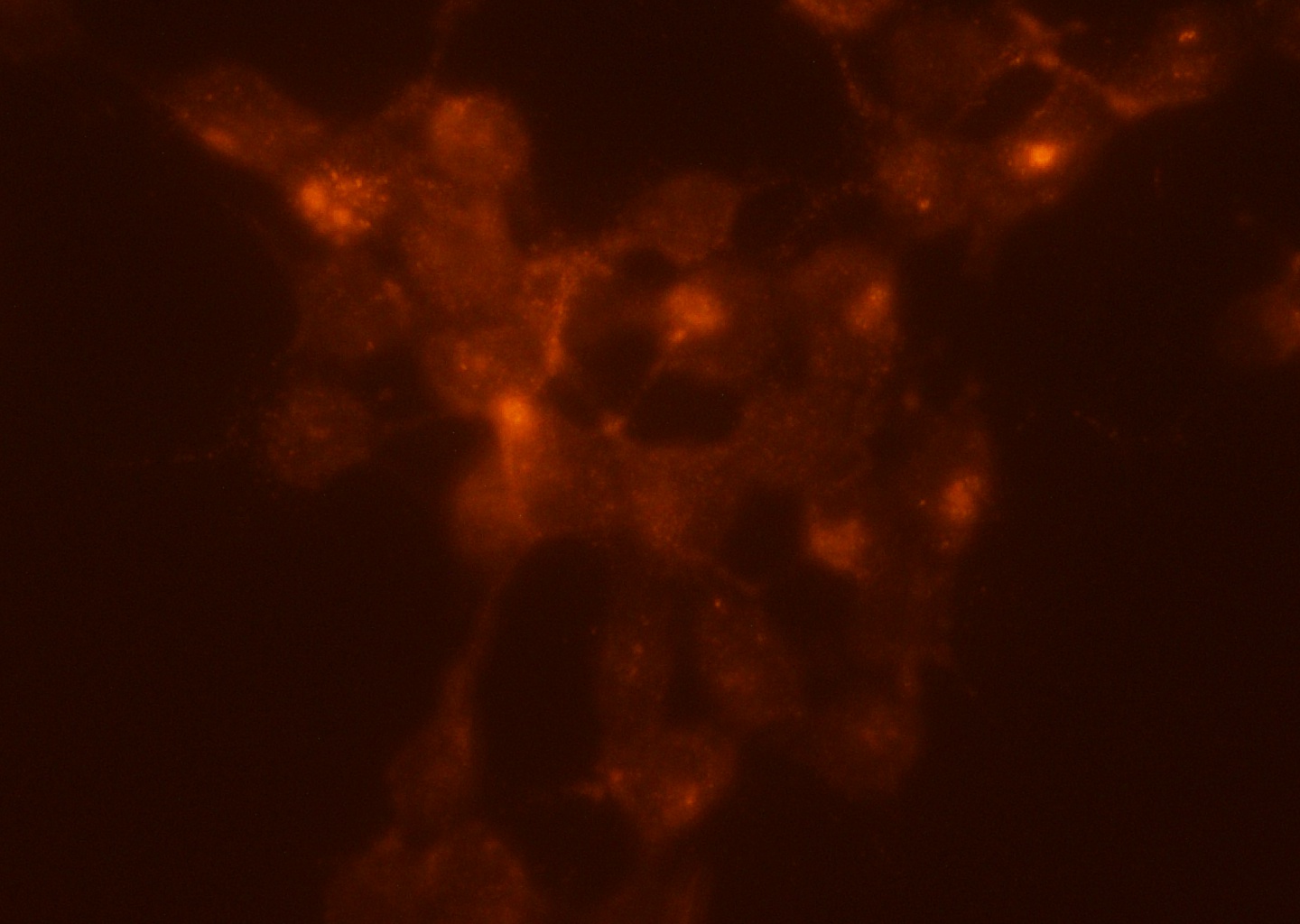

Supplement: Supplementary file 2 — Source Data Fig. 1 [file 44318_2024_35_MOESM2_ESM.zip › EMBOJ-2023-115792R2_SourceData_Fig1/Fig1C/R3/WT SINV 2A-GFP/J2.jpg]

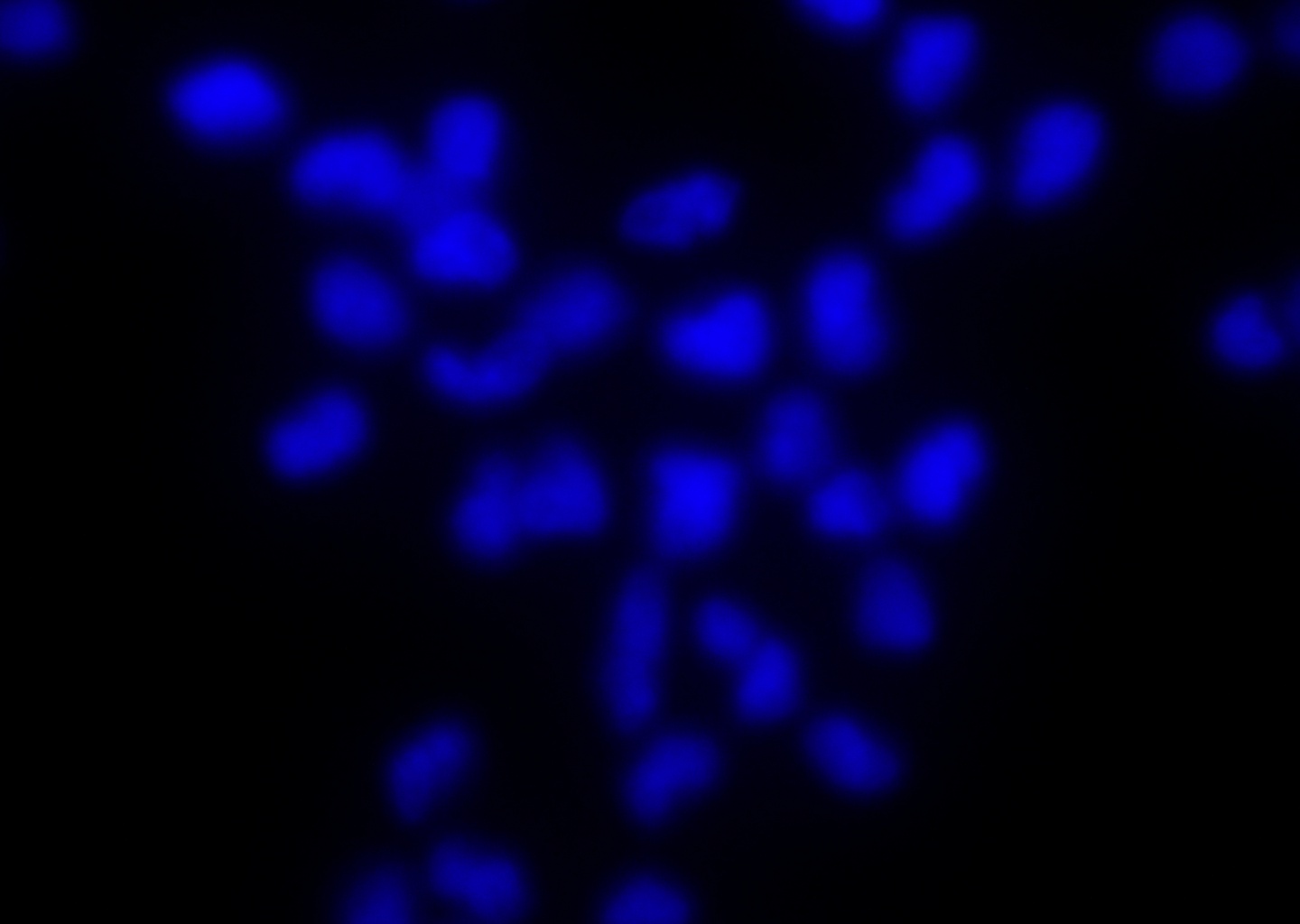

Supplement: Supplementary file 2 — Source Data Fig. 1 [file 44318_2024_35_MOESM2_ESM.zip › EMBOJ-2023-115792R2_SourceData_Fig1/Fig1C/R3/WT SINV 2A-GFP/DAPI.jpg]

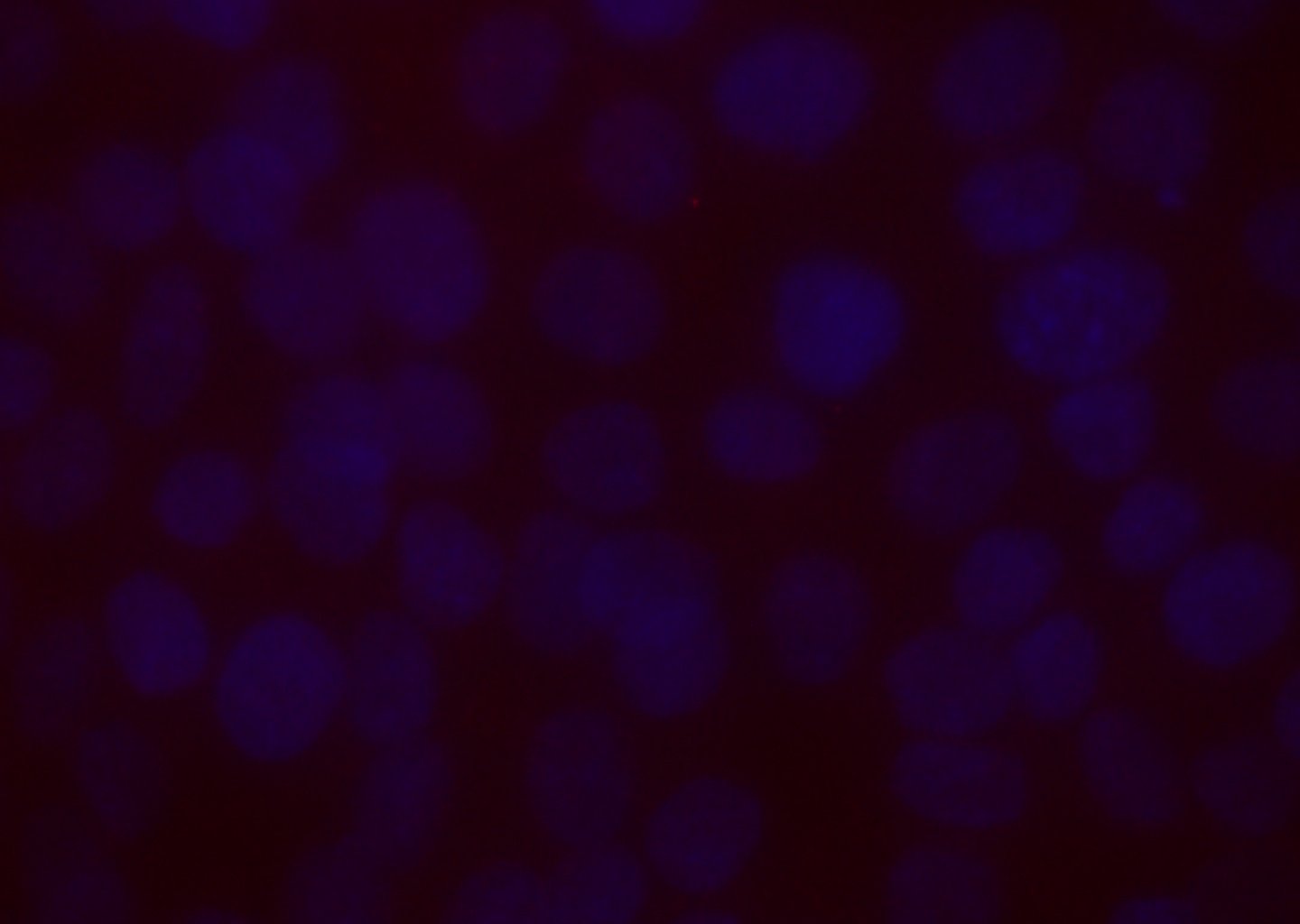

Supplement: Supplementary file 2 — Source Data Fig. 1 [file 44318_2024_35_MOESM2_ESM.zip › EMBOJ-2023-115792R2_SourceData_Fig1/Fig1C/R3/WT MOCK/WT MOCK merge.jpg]

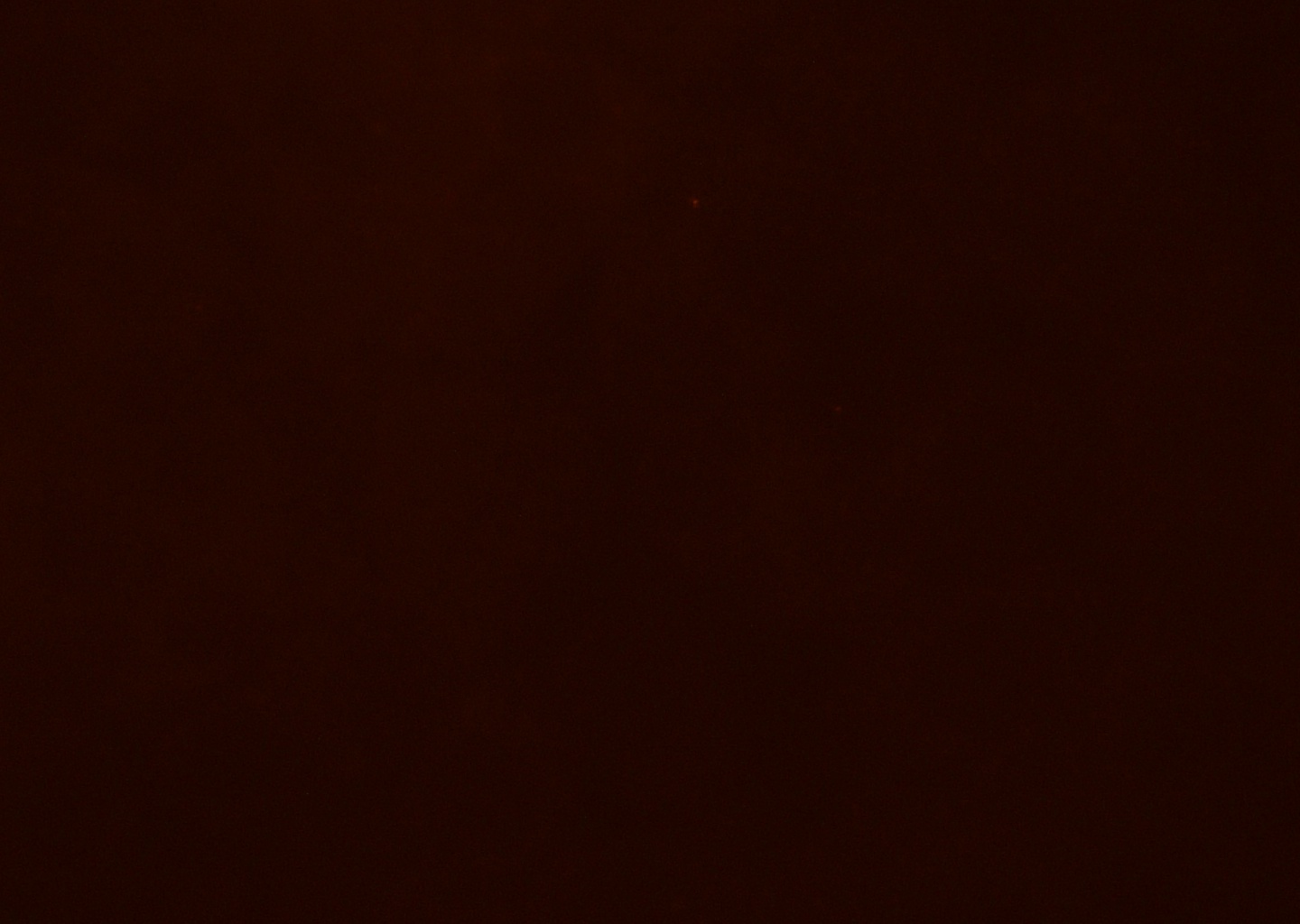

Supplement: Supplementary file 2 — Source Data Fig. 1 [file 44318_2024_35_MOESM2_ESM.zip › EMBOJ-2023-115792R2_SourceData_Fig1/Fig1C/R3/WT MOCK/J2.jpg]

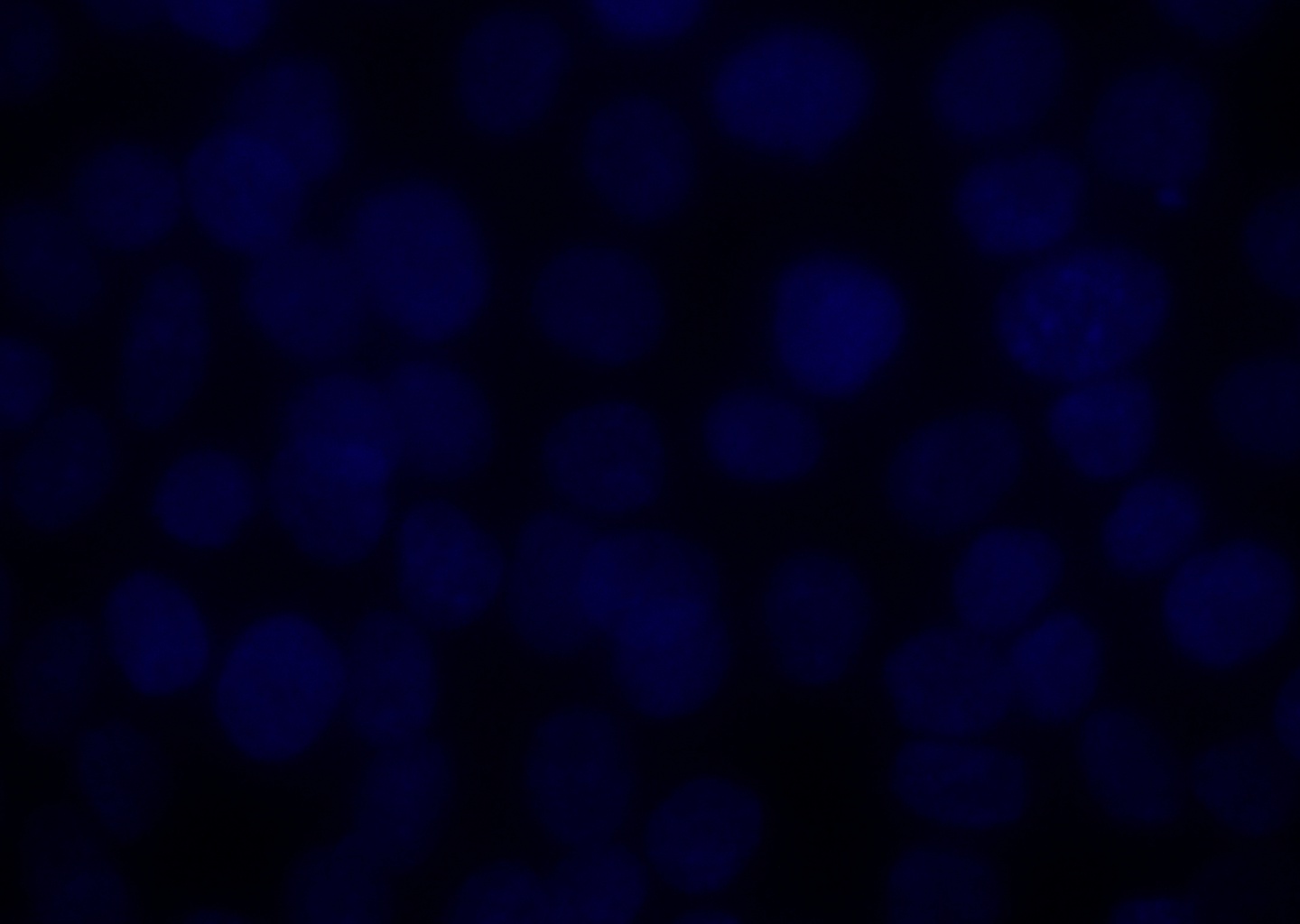

Supplement: Supplementary file 2 — Source Data Fig. 1 [file 44318_2024_35_MOESM2_ESM.zip › EMBOJ-2023-115792R2_SourceData_Fig1/Fig1C/R3/WT MOCK/DAPI.jpg]

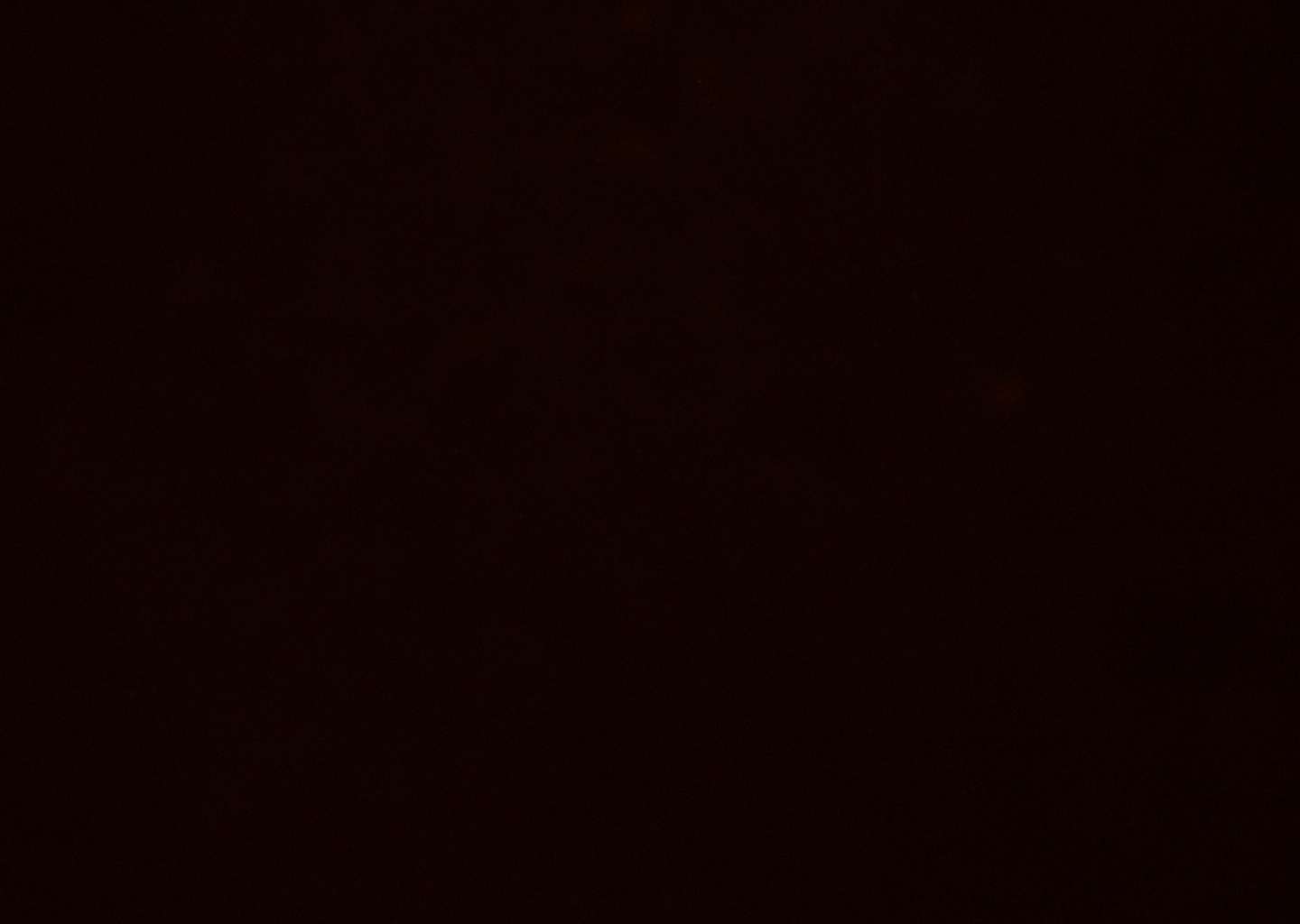

Supplement: Supplementary file 2 — Source Data Fig. 1 [file 44318_2024_35_MOESM2_ESM.zip › EMBOJ-2023-115792R2_SourceData_Fig1/Fig1C/R3/N1 MOCK/J2.jpg]

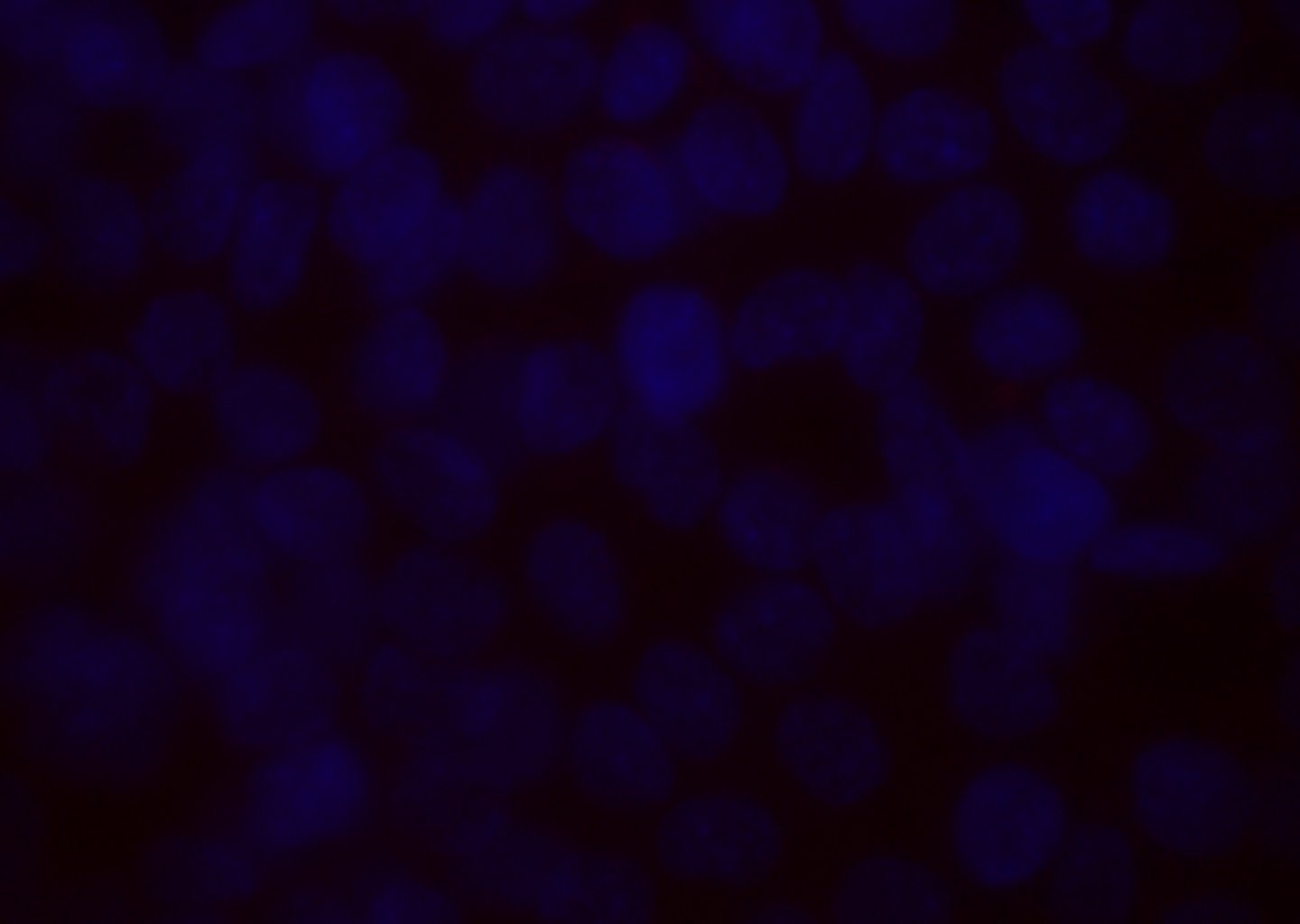

Supplement: Supplementary file 2 — Source Data Fig. 1 [file 44318_2024_35_MOESM2_ESM.zip › EMBOJ-2023-115792R2_SourceData_Fig1/Fig1C/R3/N1 MOCK/N1 MOCK merge.jpg]

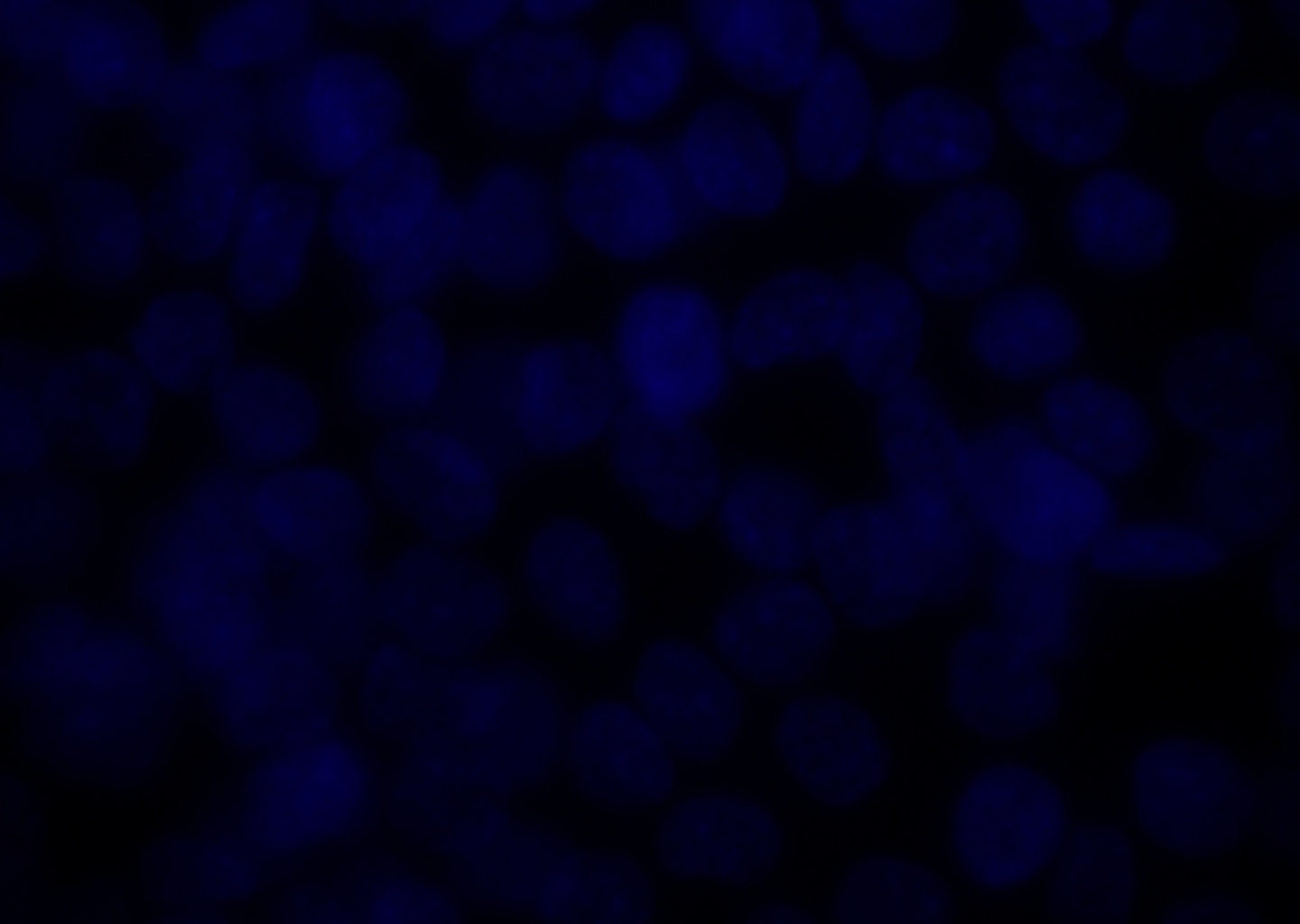

Supplement: Supplementary file 2 — Source Data Fig. 1 [file 44318_2024_35_MOESM2_ESM.zip › EMBOJ-2023-115792R2_SourceData_Fig1/Fig1C/R3/N1 MOCK/DAPI.jpg]

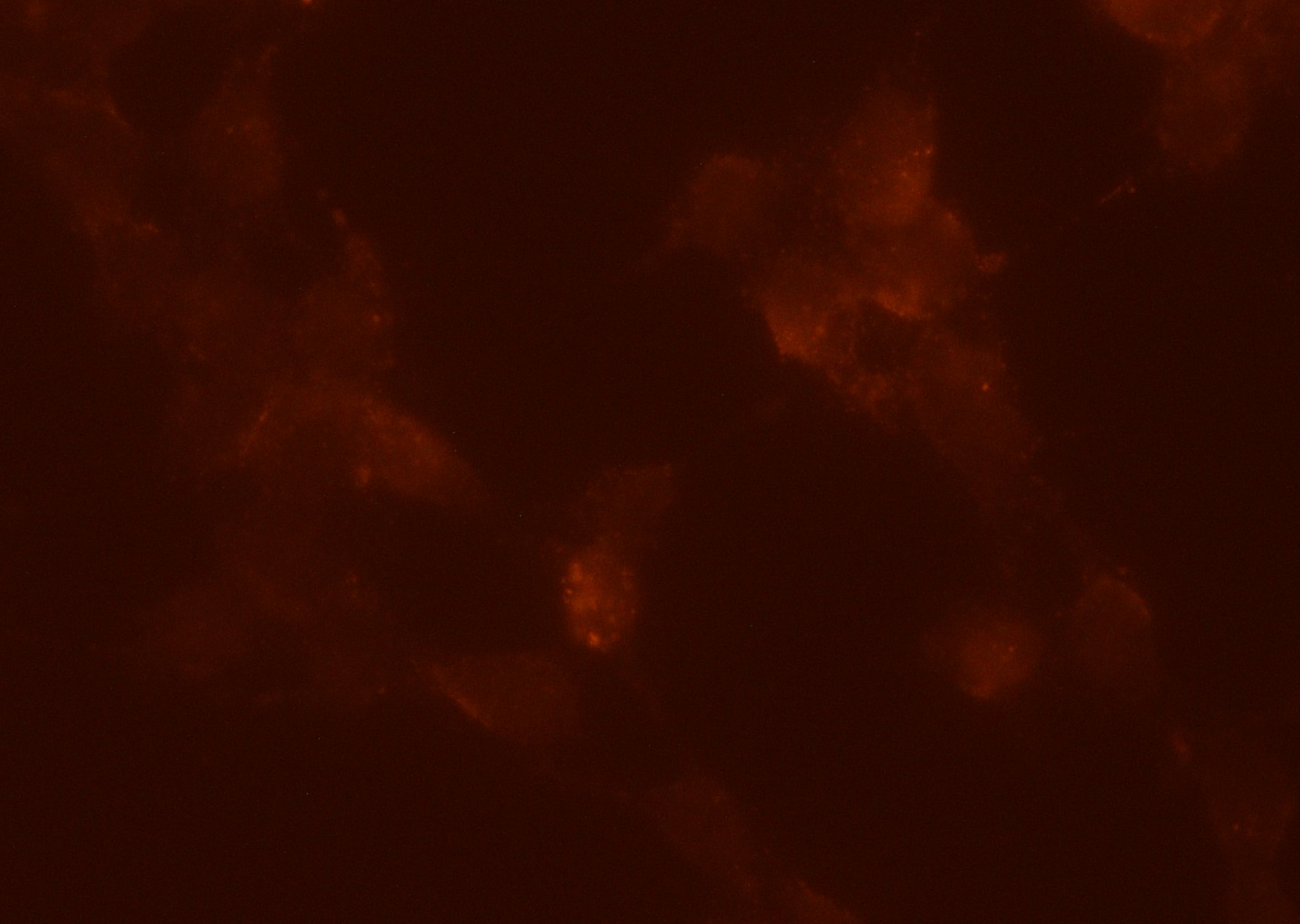

Supplement: Supplementary file 2 — Source Data Fig. 1 [file 44318_2024_35_MOESM2_ESM.zip › EMBOJ-2023-115792R2_SourceData_Fig1/Fig1C/R3/N1 SINV WT/J2.jpg]

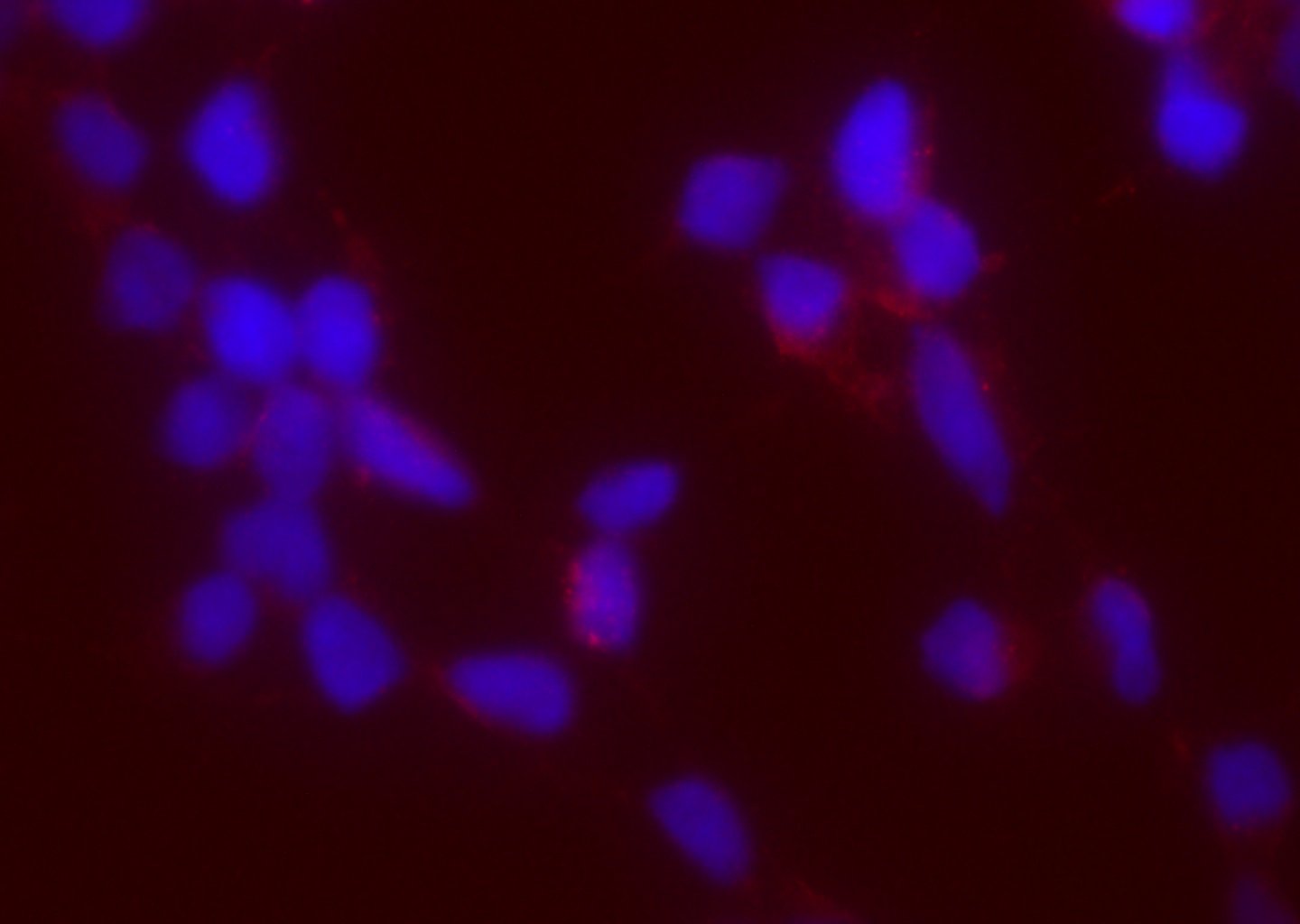

Supplement: Supplementary file 2 — Source Data Fig. 1 [file 44318_2024_35_MOESM2_ESM.zip › EMBOJ-2023-115792R2_SourceData_Fig1/Fig1C/R3/N1 SINV WT/N1 SINV WT merge.jpg]

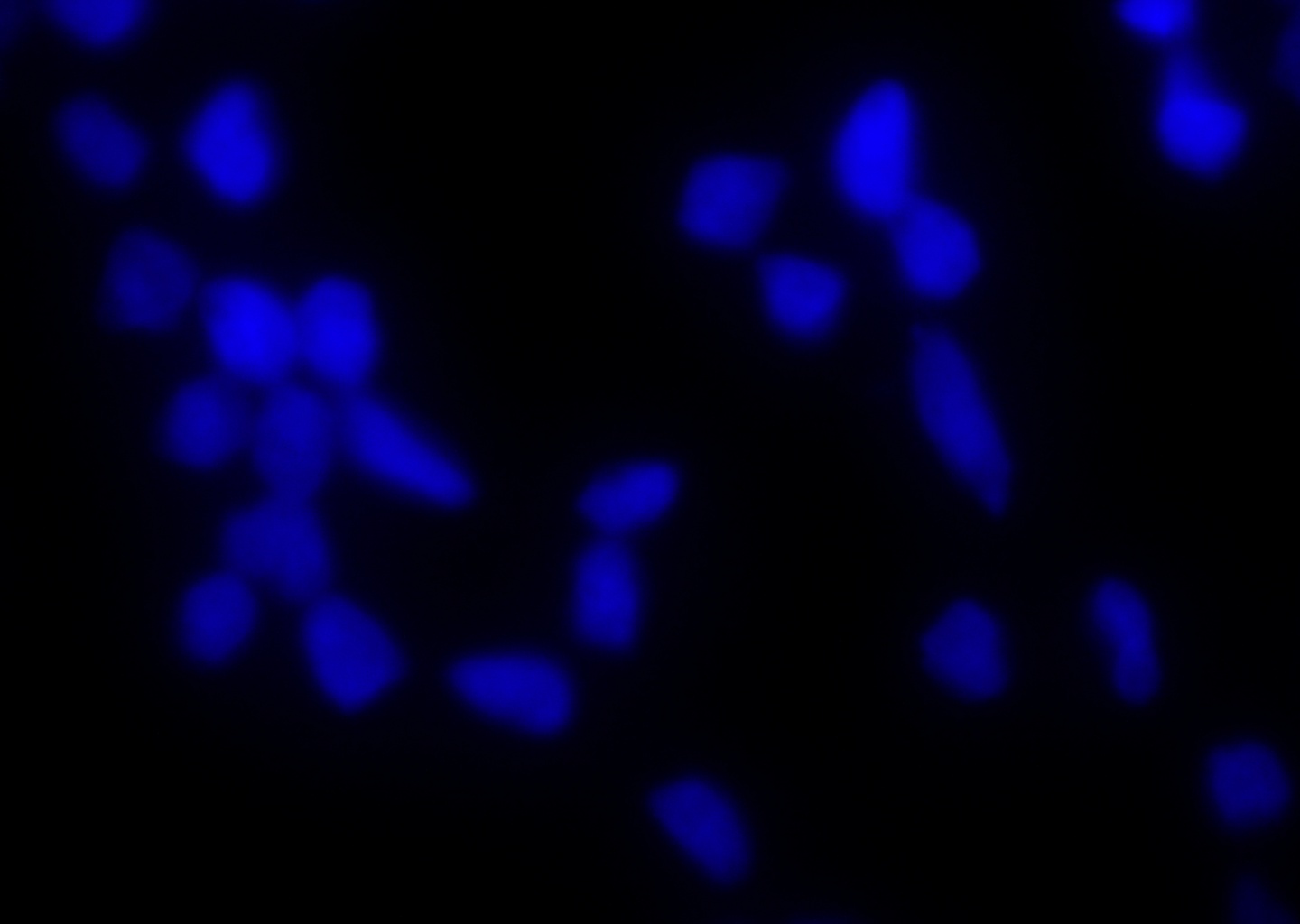

Supplement: Supplementary file 2 — Source Data Fig. 1 [file 44318_2024_35_MOESM2_ESM.zip › EMBOJ-2023-115792R2_SourceData_Fig1/Fig1C/R3/N1 SINV WT/DAPI.jpg]

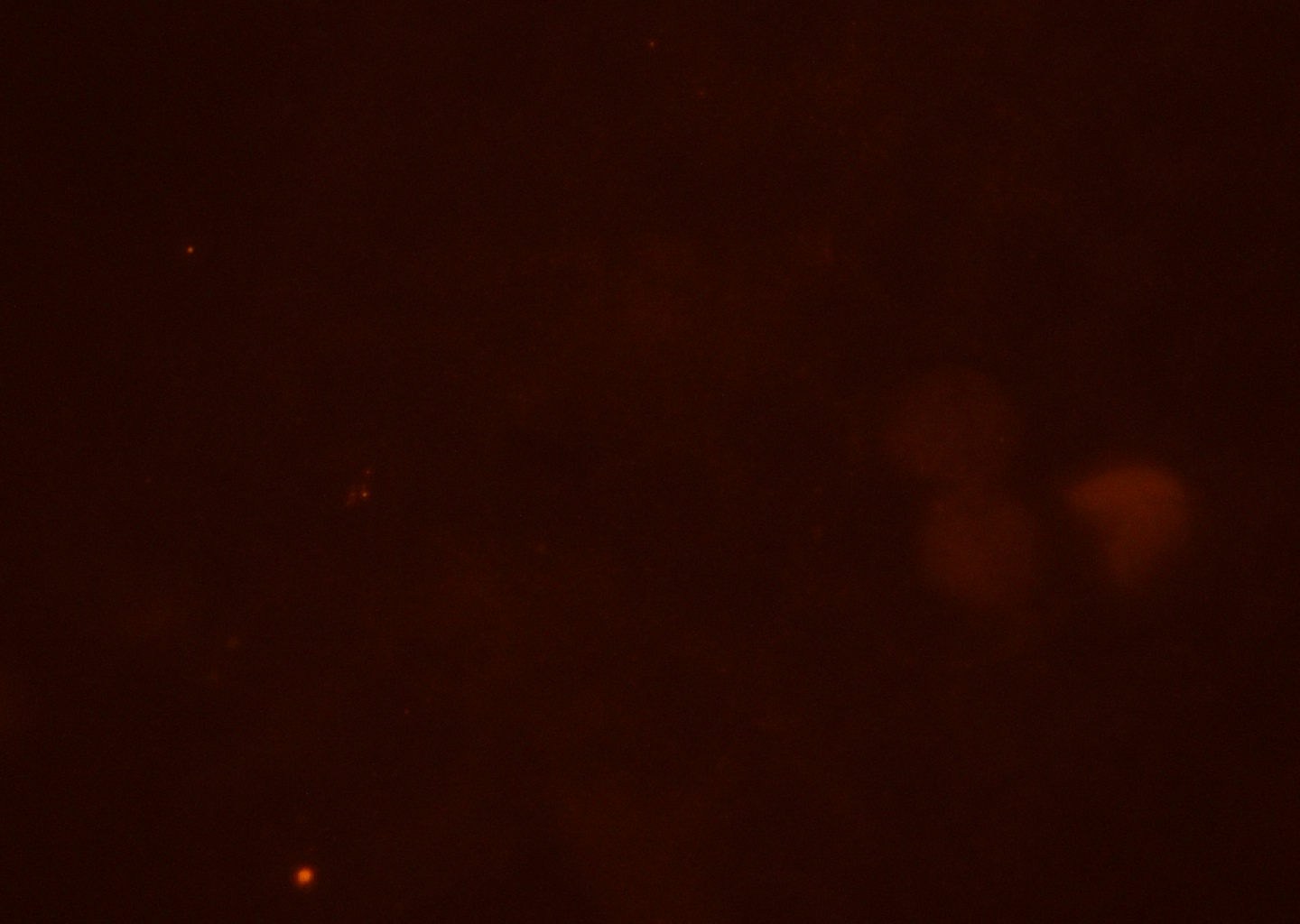

Supplement: Supplementary file 2 — Source Data Fig. 1 [file 44318_2024_35_MOESM2_ESM.zip › EMBOJ-2023-115792R2_SourceData_Fig1/Fig1C/R3/N1 SINV 2A-GFP/J2.jpg]

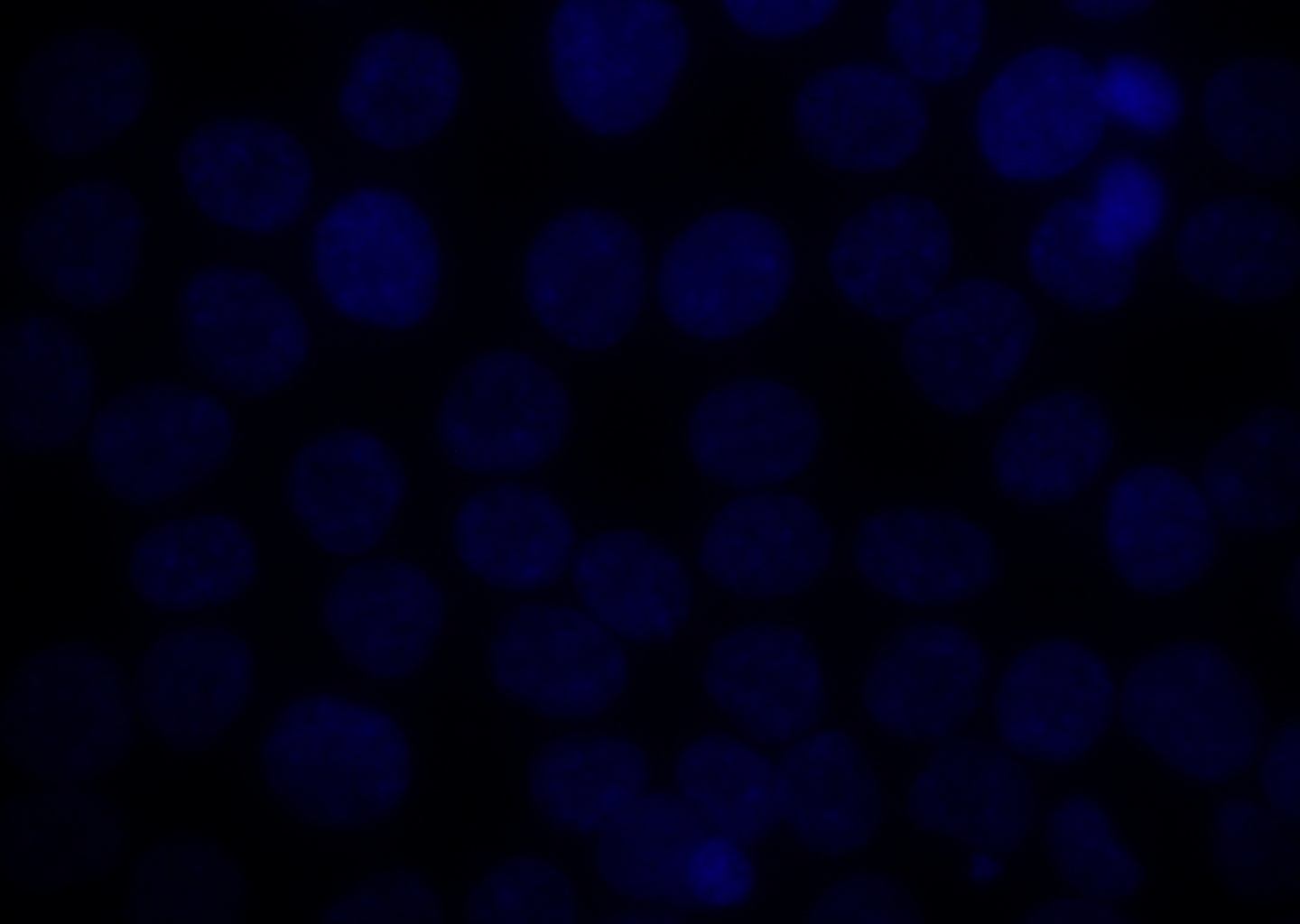

Supplement: Supplementary file 2 — Source Data Fig. 1 [file 44318_2024_35_MOESM2_ESM.zip › EMBOJ-2023-115792R2_SourceData_Fig1/Fig1C/R3/N1 SINV 2A-GFP/DAPI.jpg]

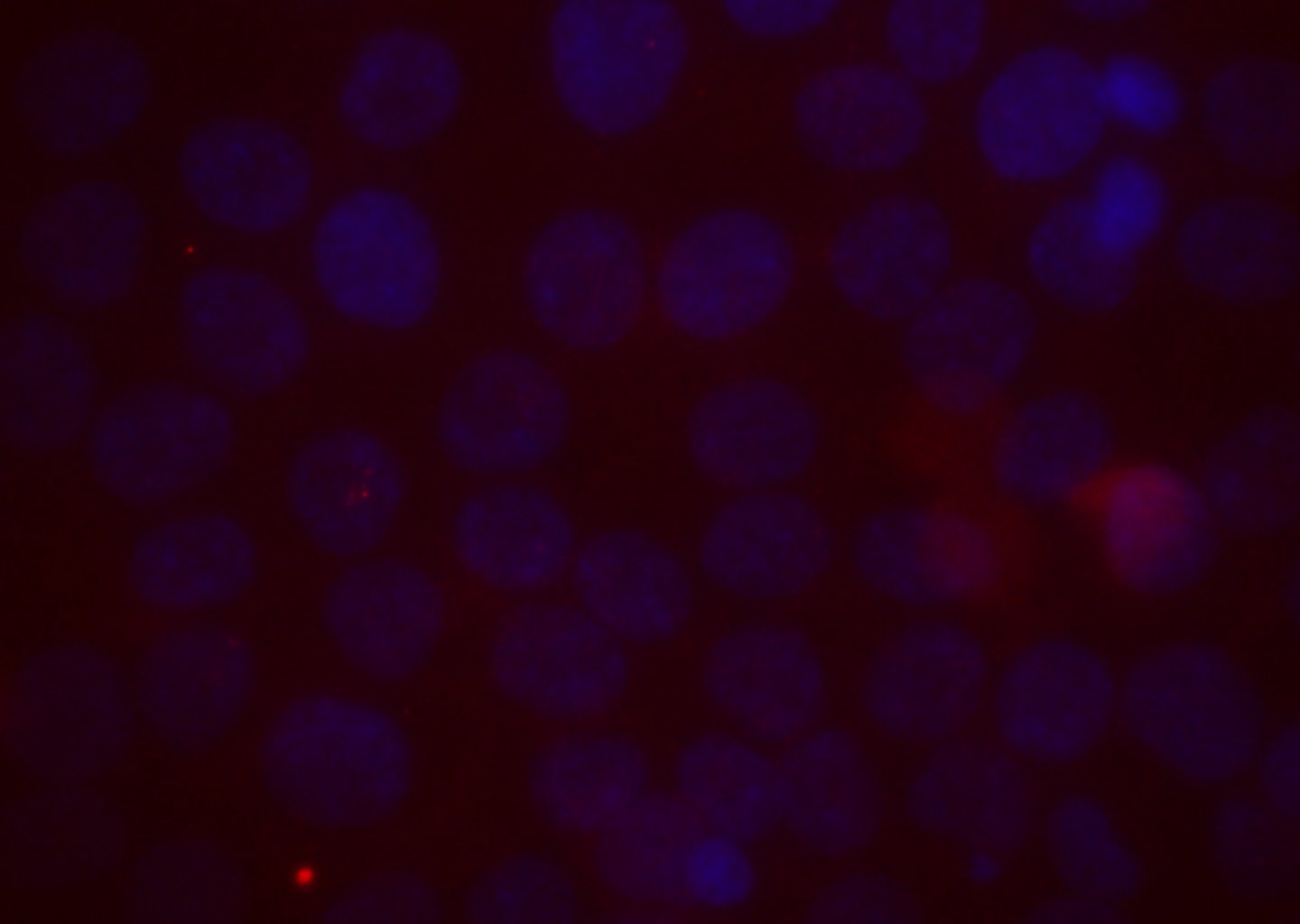

Supplement: Supplementary file 2 — Source Data Fig. 1 [file 44318_2024_35_MOESM2_ESM.zip › EMBOJ-2023-115792R2_SourceData_Fig1/Fig1C/R3/N1 SINV 2A-GFP/N1 2A-GFP merge.jpg]

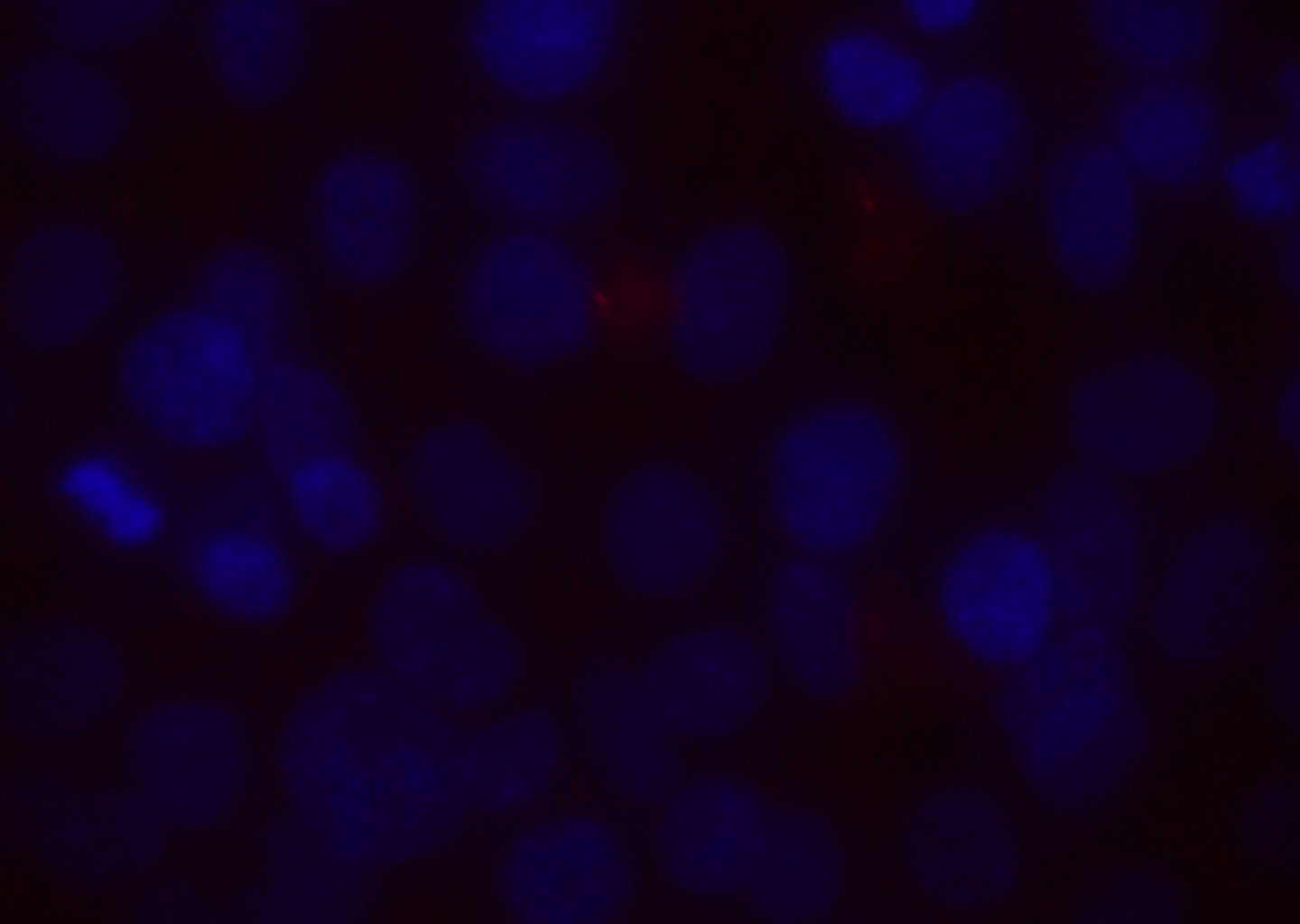

Supplement: Supplementary file 2 — Source Data Fig. 1 [file 44318_2024_35_MOESM2_ESM.zip › EMBOJ-2023-115792R2_SourceData_Fig1/Fig1C/R3/N1 SINV GFP/N1 SINV-GFP merge.jpg]

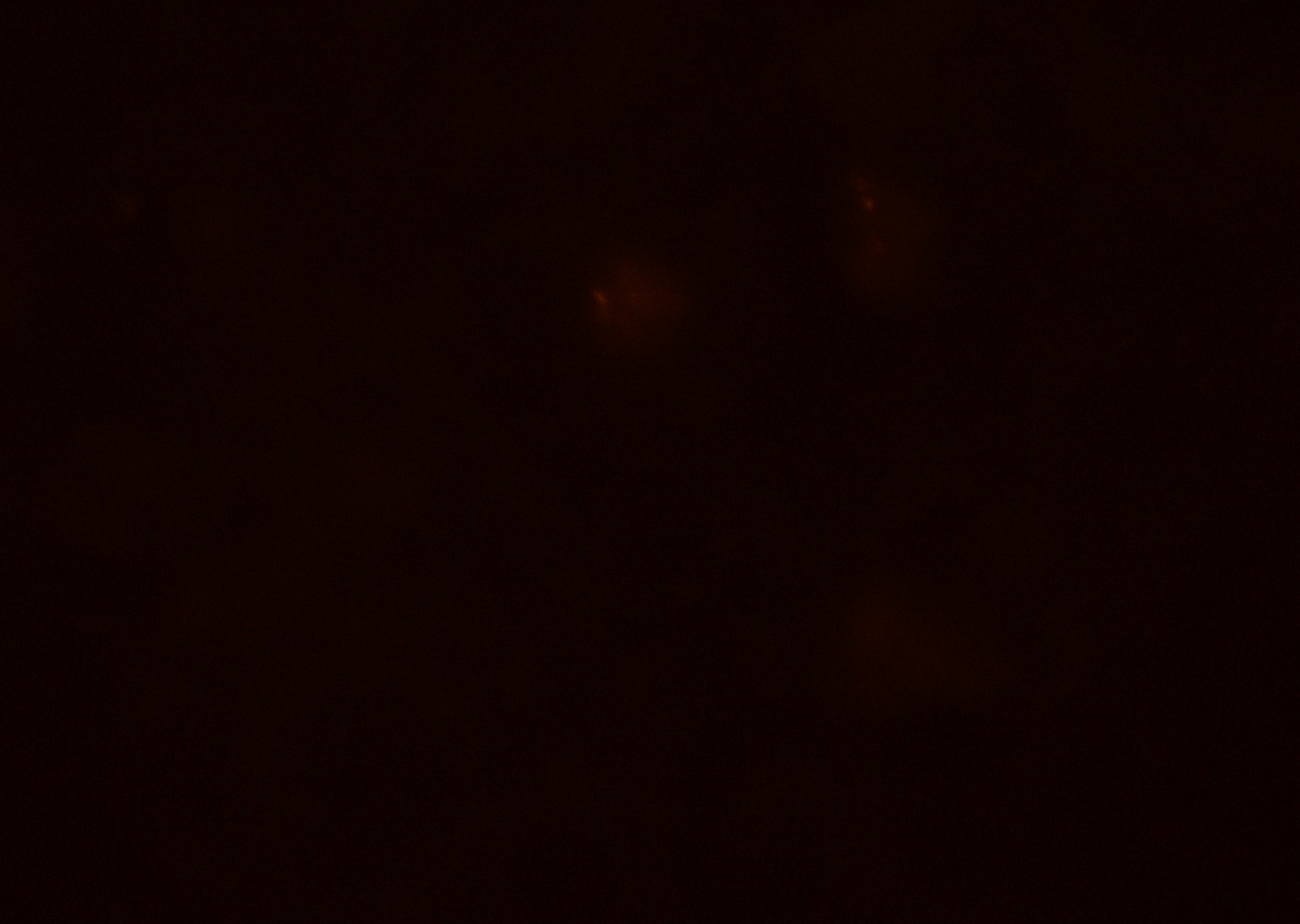

Supplement: Supplementary file 2 — Source Data Fig. 1 [file 44318_2024_35_MOESM2_ESM.zip › EMBOJ-2023-115792R2_SourceData_Fig1/Fig1C/R3/N1 SINV GFP/J2.jpg]

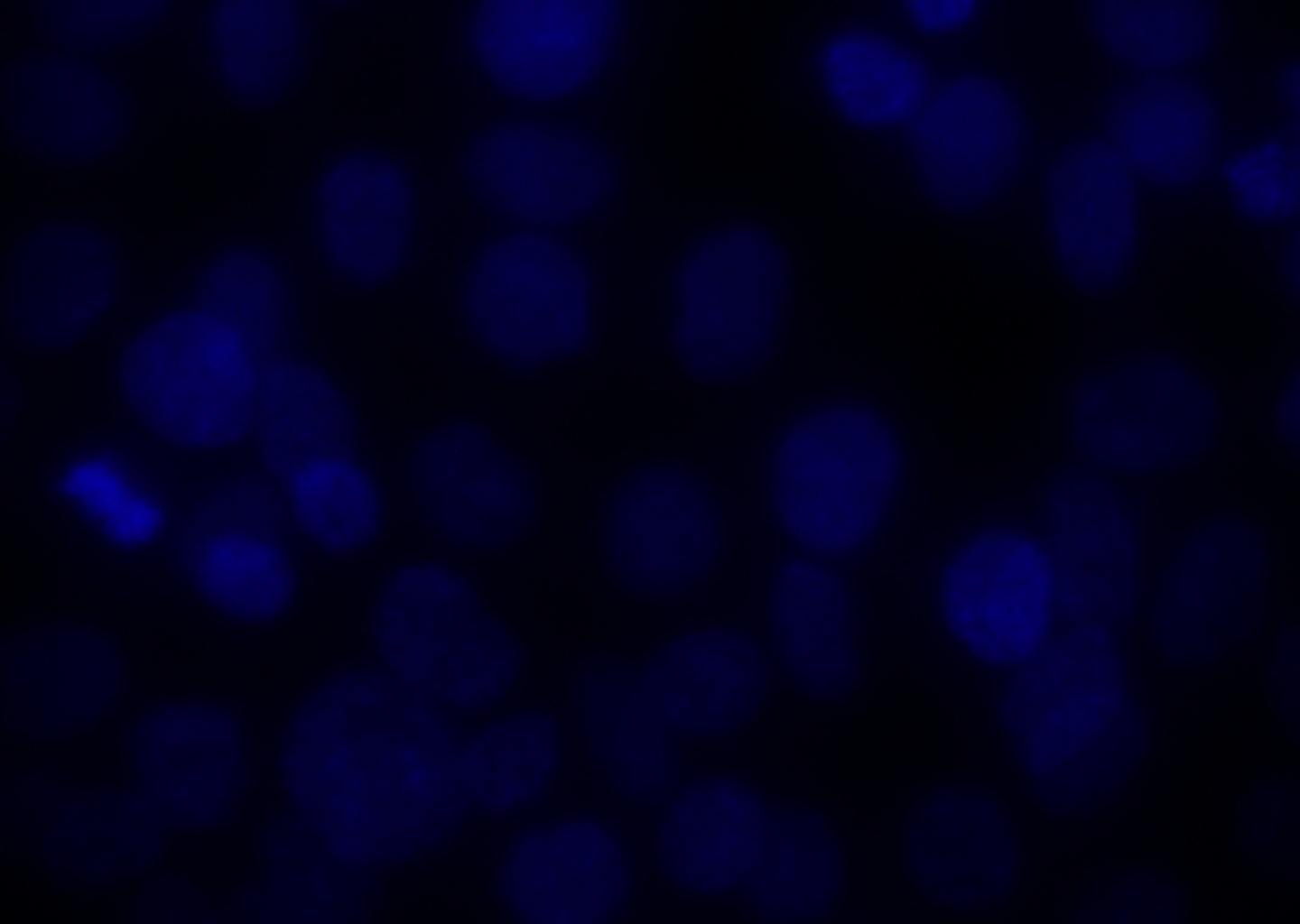

Supplement: Supplementary file 2 — Source Data Fig. 1 [file 44318_2024_35_MOESM2_ESM.zip › EMBOJ-2023-115792R2_SourceData_Fig1/Fig1C/R3/N1 SINV GFP/DAPI.jpg]

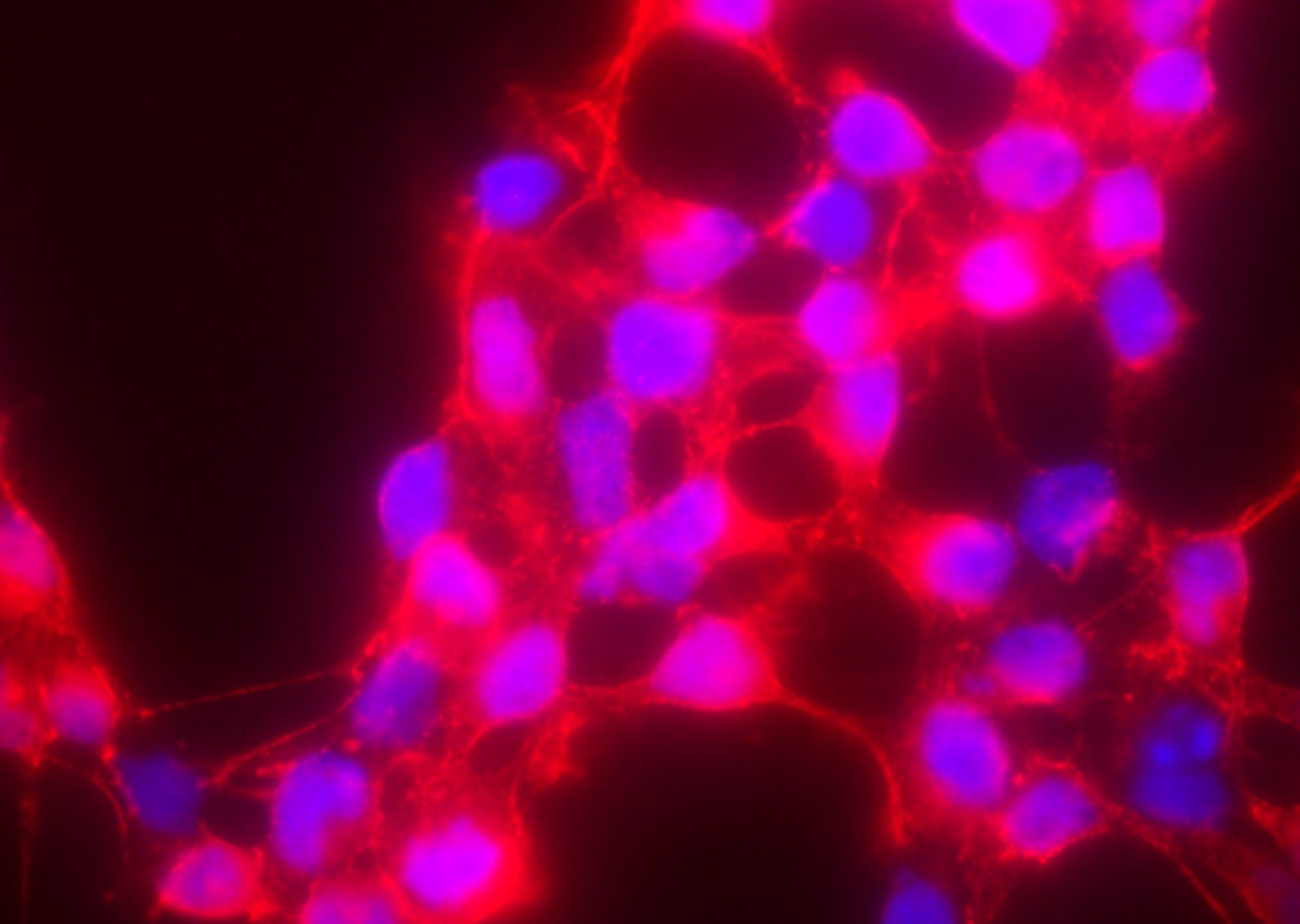

Supplement: Supplementary file 2 — Source Data Fig. 1 [file 44318_2024_35_MOESM2_ESM.zip › EMBOJ-2023-115792R2_SourceData_Fig1/Fig1C/R3/WT SINV-GFP/WT SINV-GFP merge.jpg]

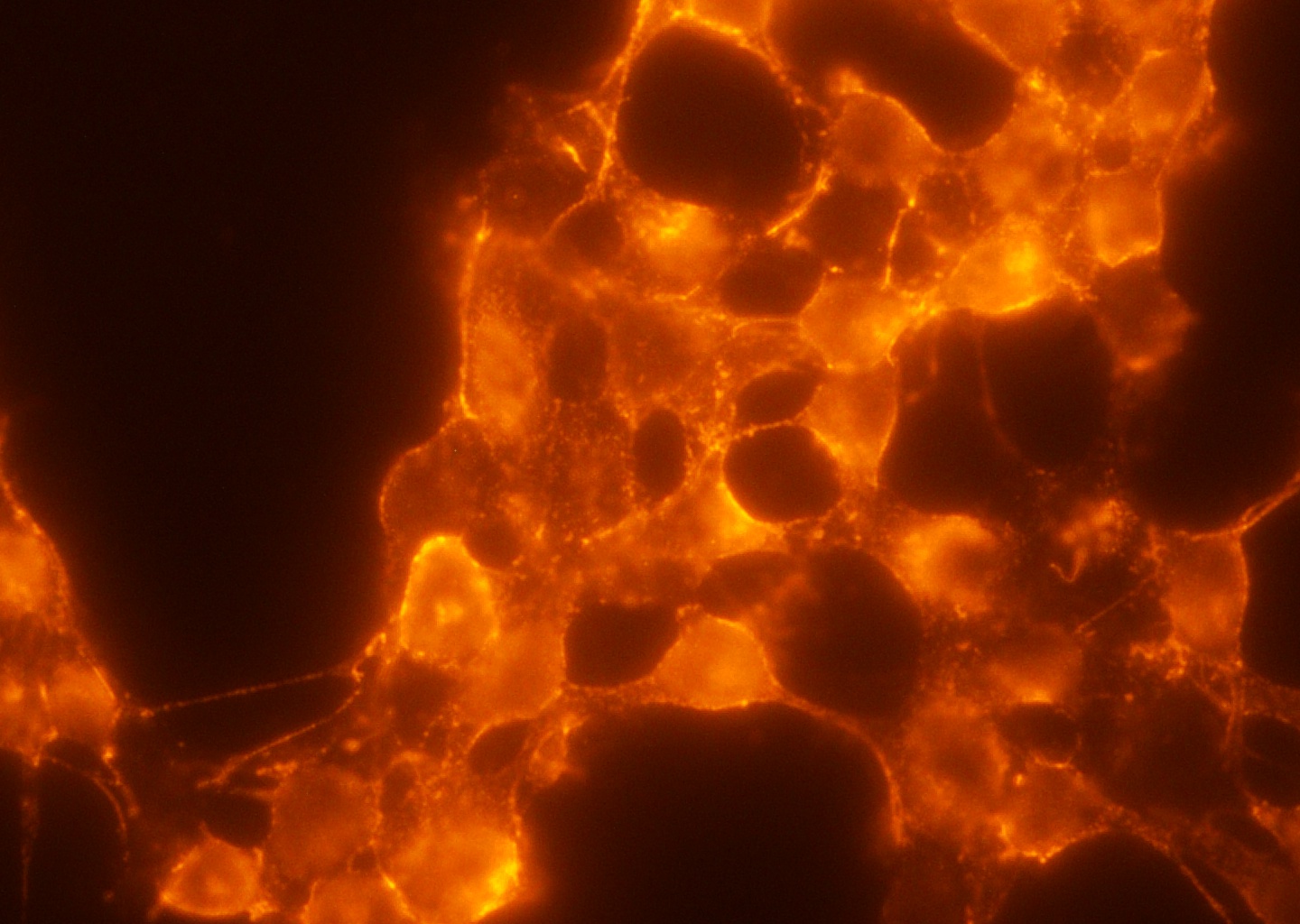

Supplement: Supplementary file 2 — Source Data Fig. 1 [file 44318_2024_35_MOESM2_ESM.zip › EMBOJ-2023-115792R2_SourceData_Fig1/Fig1C/R3/WT SINV-GFP/J2.jpg]

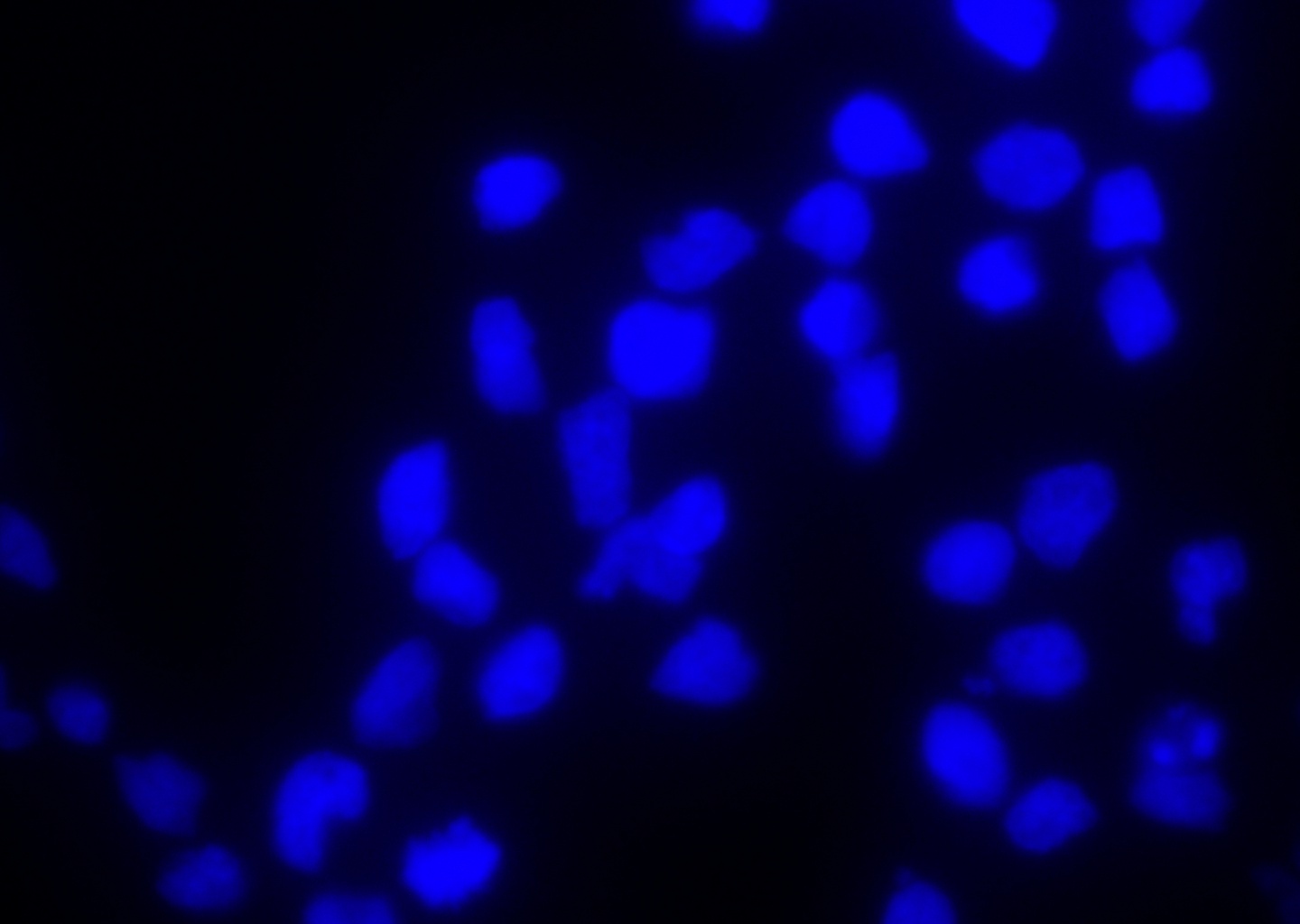

Supplement: Supplementary file 2 — Source Data Fig. 1 [file 44318_2024_35_MOESM2_ESM.zip › EMBOJ-2023-115792R2_SourceData_Fig1/Fig1C/R3/WT SINV-GFP/DAPI.jpg]

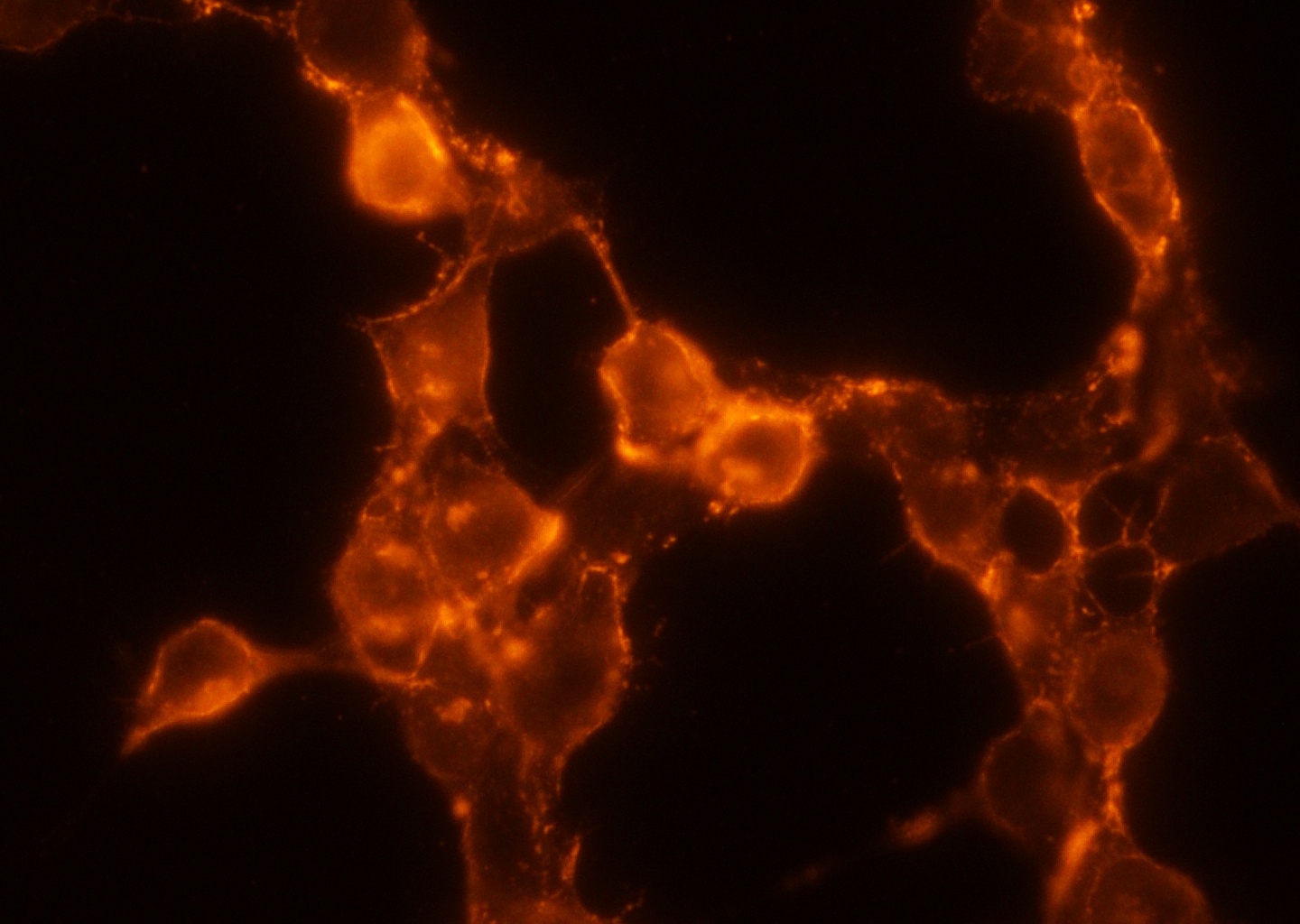

Supplement: Supplementary file 2 — Source Data Fig. 1 [file 44318_2024_35_MOESM2_ESM.zip › EMBOJ-2023-115792R2_SourceData_Fig1/Fig1C/R2/WT SINV WT/J2.jpg]

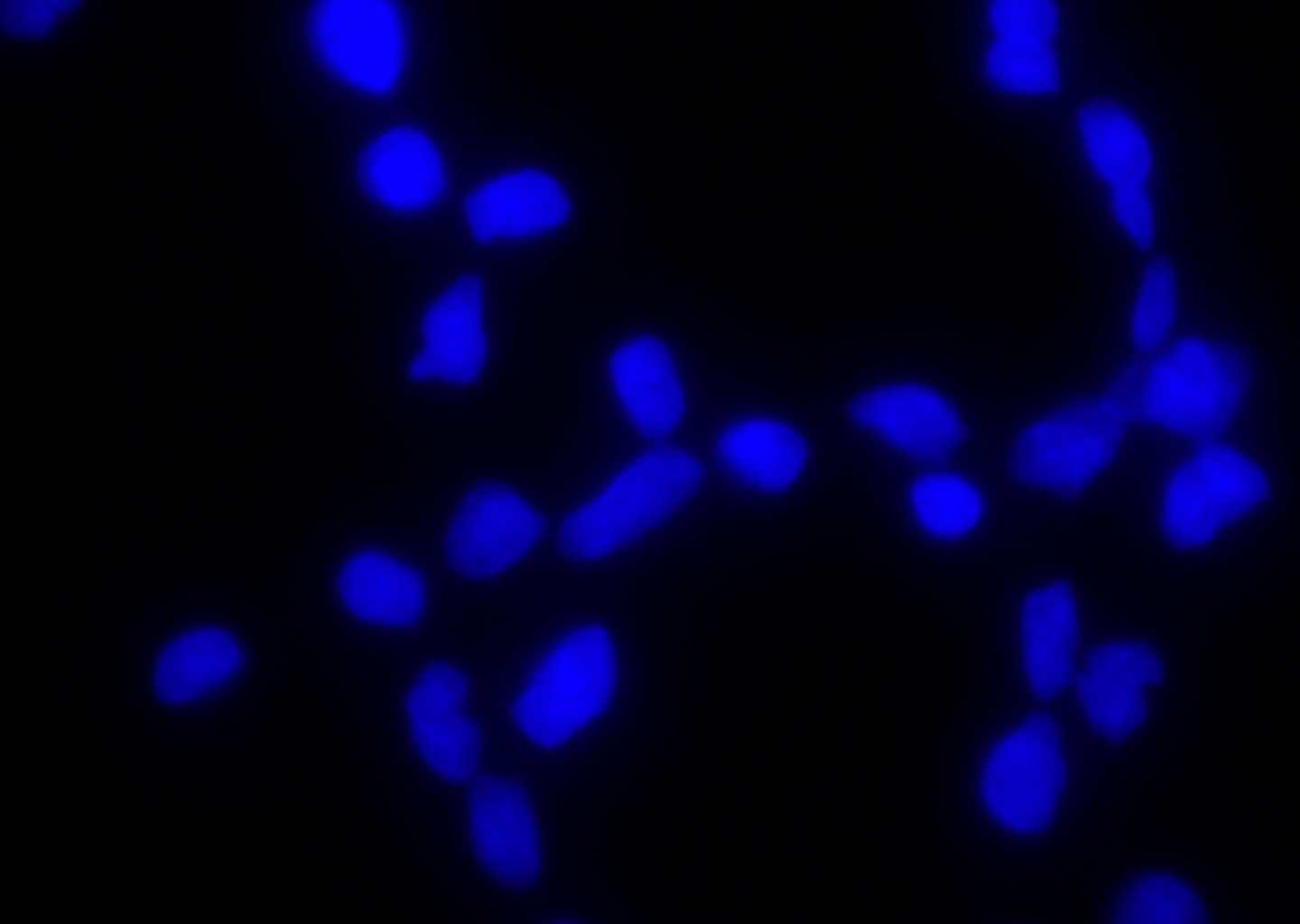

Supplement: Supplementary file 2 — Source Data Fig. 1 [file 44318_2024_35_MOESM2_ESM.zip › EMBOJ-2023-115792R2_SourceData_Fig1/Fig1C/R2/WT SINV WT/DAPI.jpg]

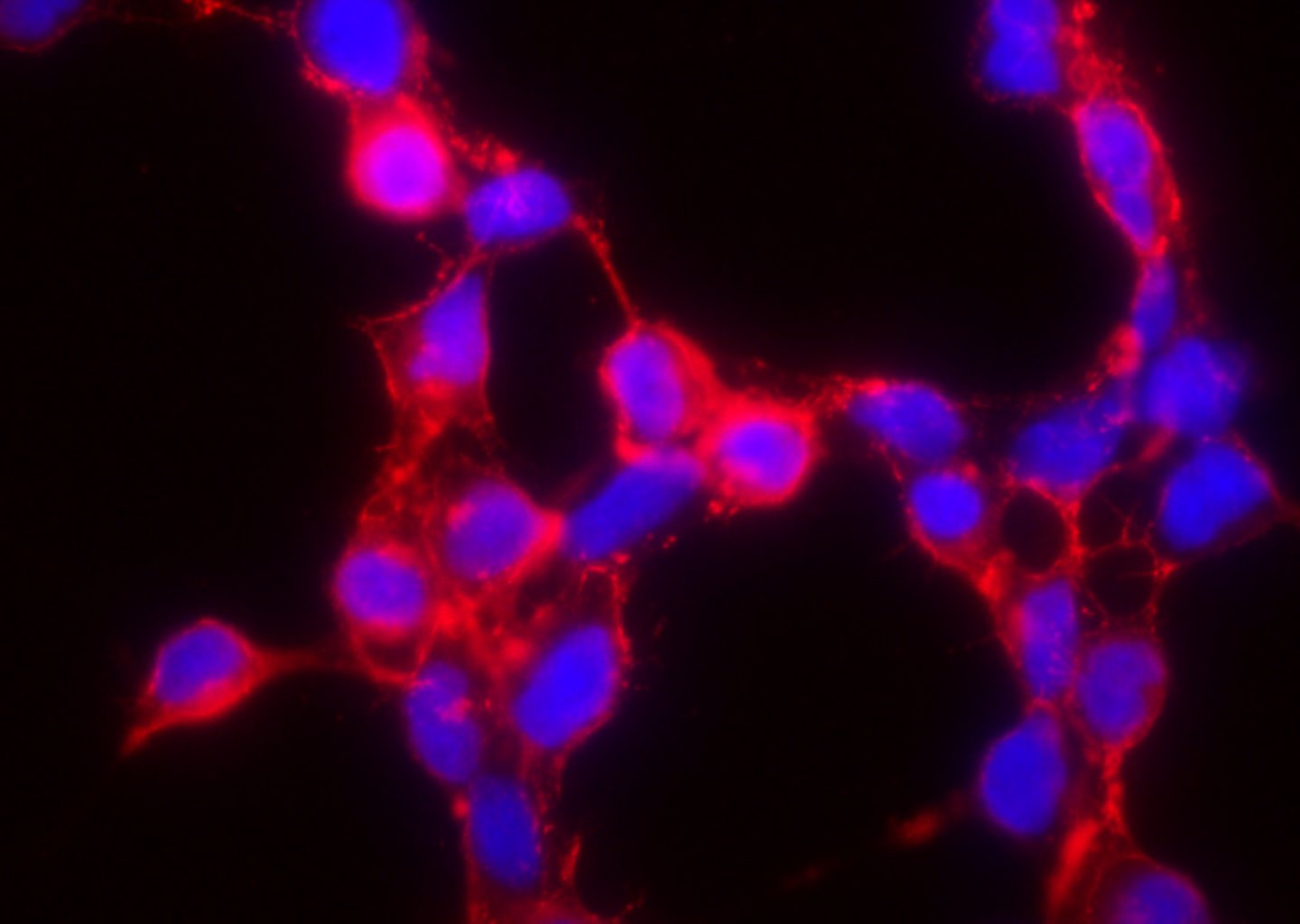

Supplement: Supplementary file 2 — Source Data Fig. 1 [file 44318_2024_35_MOESM2_ESM.zip › EMBOJ-2023-115792R2_SourceData_Fig1/Fig1C/R2/WT SINV WT/WT SINV WT merge.jpg]

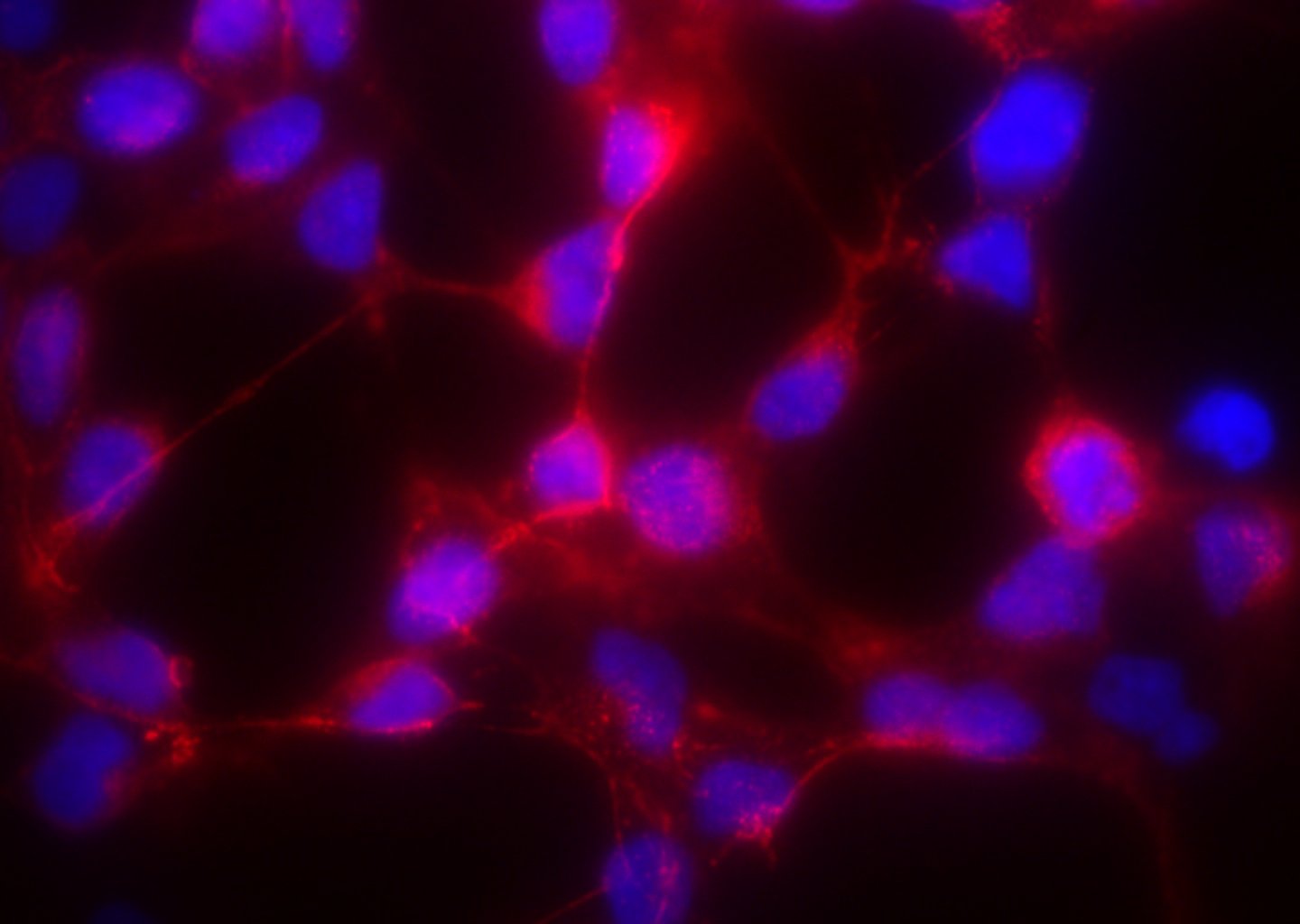

Supplement: Supplementary file 2 — Source Data Fig. 1 [file 44318_2024_35_MOESM2_ESM.zip › EMBOJ-2023-115792R2_SourceData_Fig1/Fig1C/R2/WT SINV 2A-GFP/WT 2A-GFP merge.jpg]

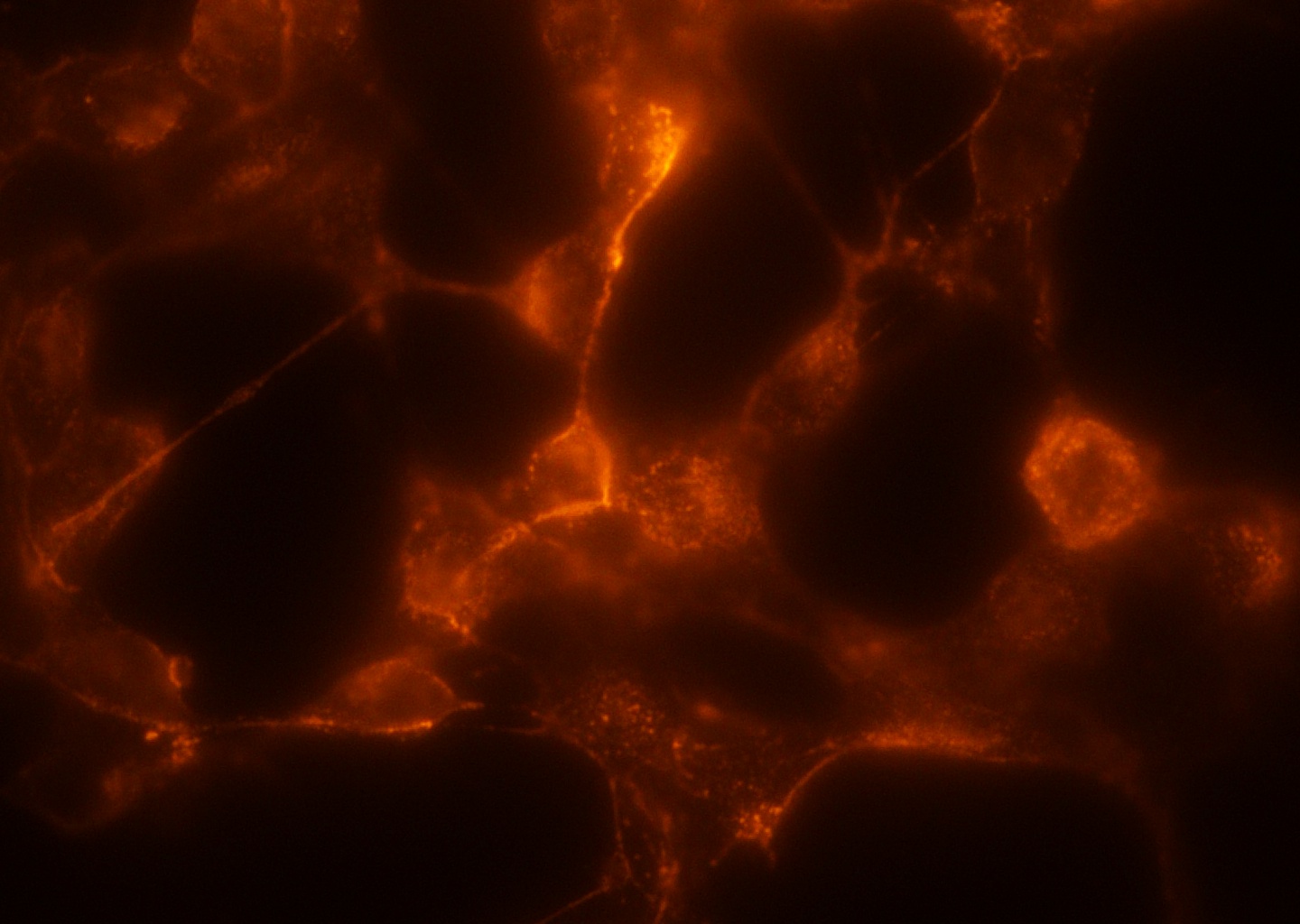

Supplement: Supplementary file 2 — Source Data Fig. 1 [file 44318_2024_35_MOESM2_ESM.zip › EMBOJ-2023-115792R2_SourceData_Fig1/Fig1C/R2/WT SINV 2A-GFP/J2.jpg]

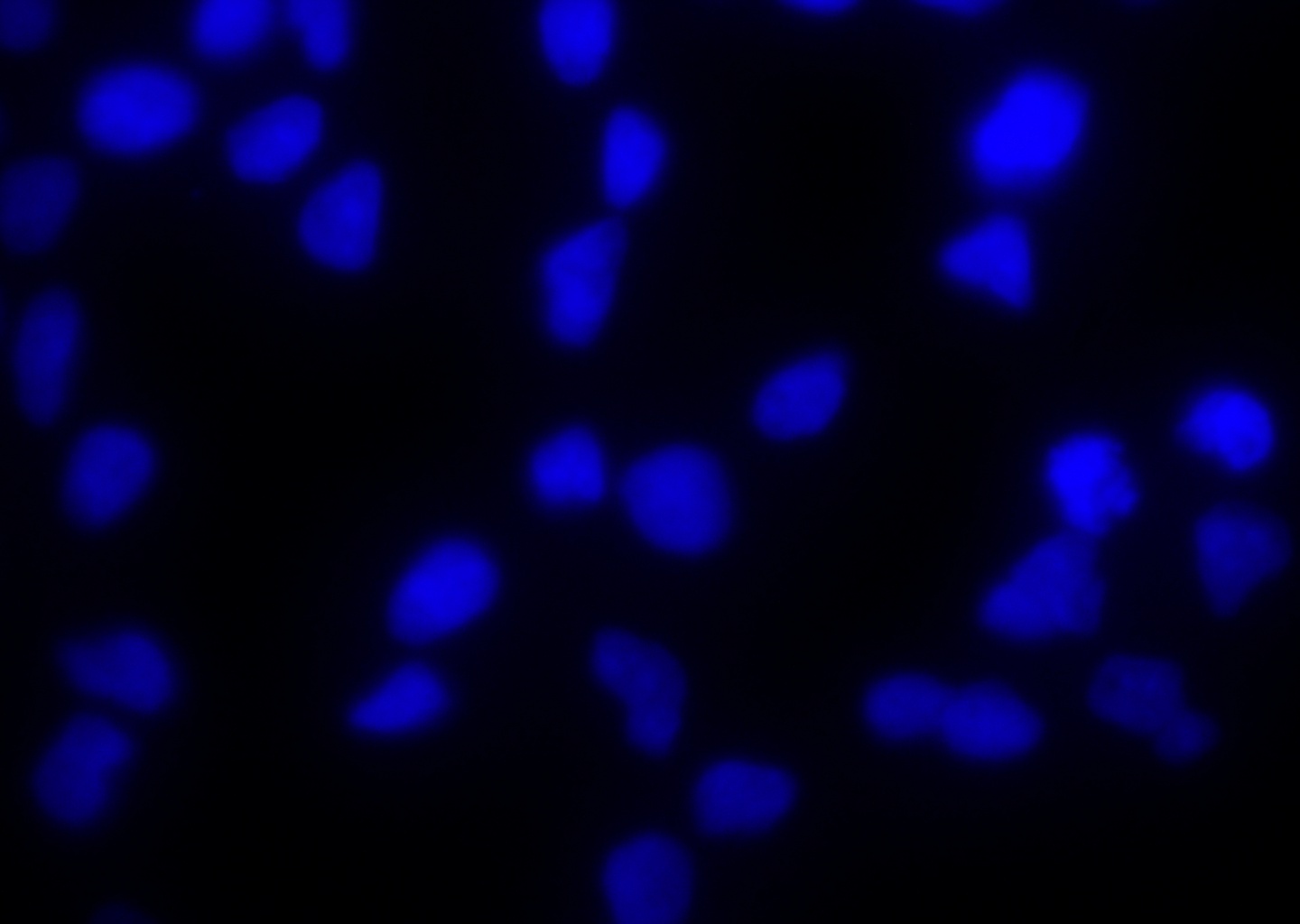

Supplement: Supplementary file 2 — Source Data Fig. 1 [file 44318_2024_35_MOESM2_ESM.zip › EMBOJ-2023-115792R2_SourceData_Fig1/Fig1C/R2/WT SINV 2A-GFP/DAPI.jpg]

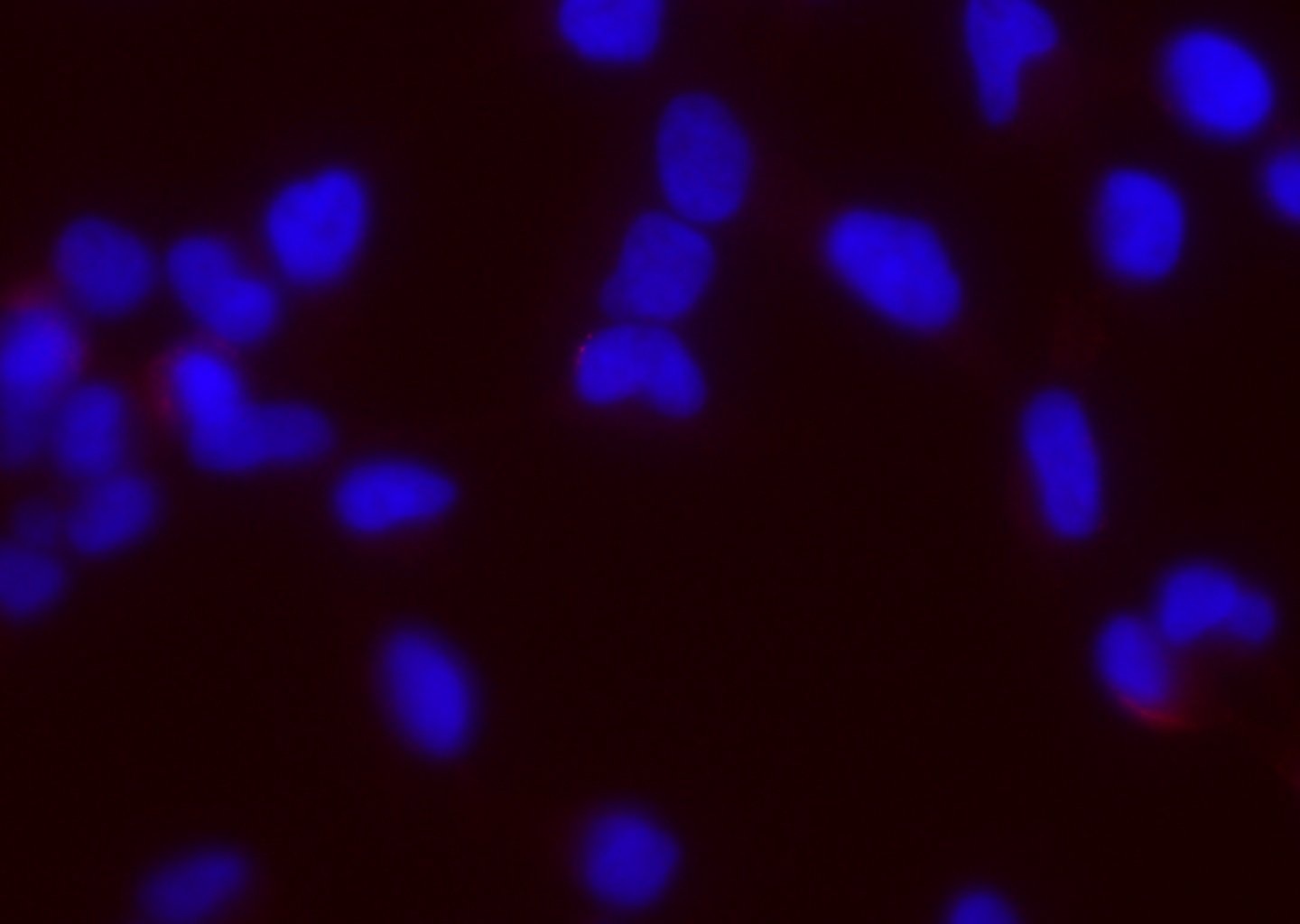

Supplement: Supplementary file 2 — Source Data Fig. 1 [file 44318_2024_35_MOESM2_ESM.zip › EMBOJ-2023-115792R2_SourceData_Fig1/Fig1C/R2/WT MOCK/WT MOCK merge.jpg]

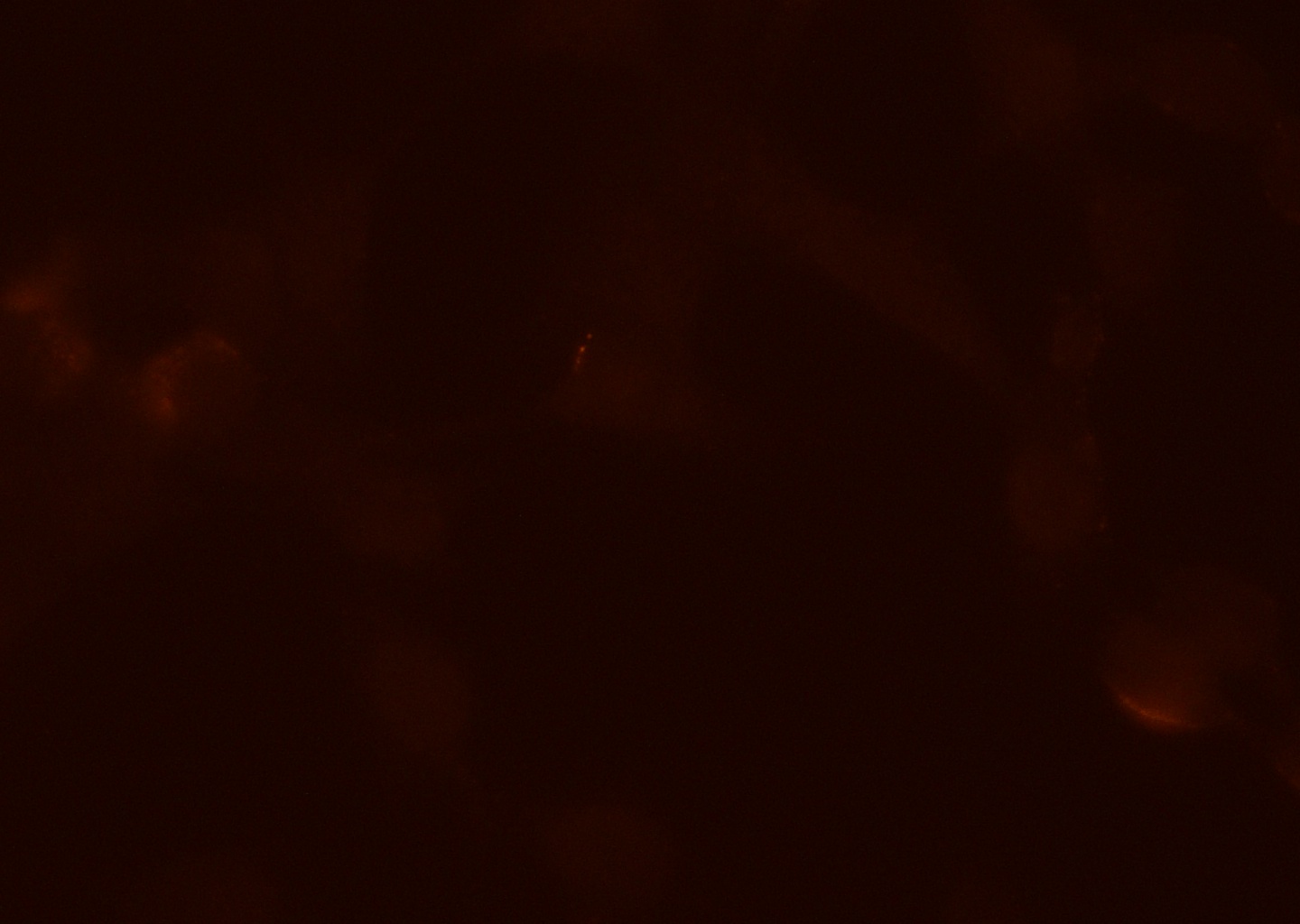

Supplement: Supplementary file 2 — Source Data Fig. 1 [file 44318_2024_35_MOESM2_ESM.zip › EMBOJ-2023-115792R2_SourceData_Fig1/Fig1C/R2/WT MOCK/J2.jpg]

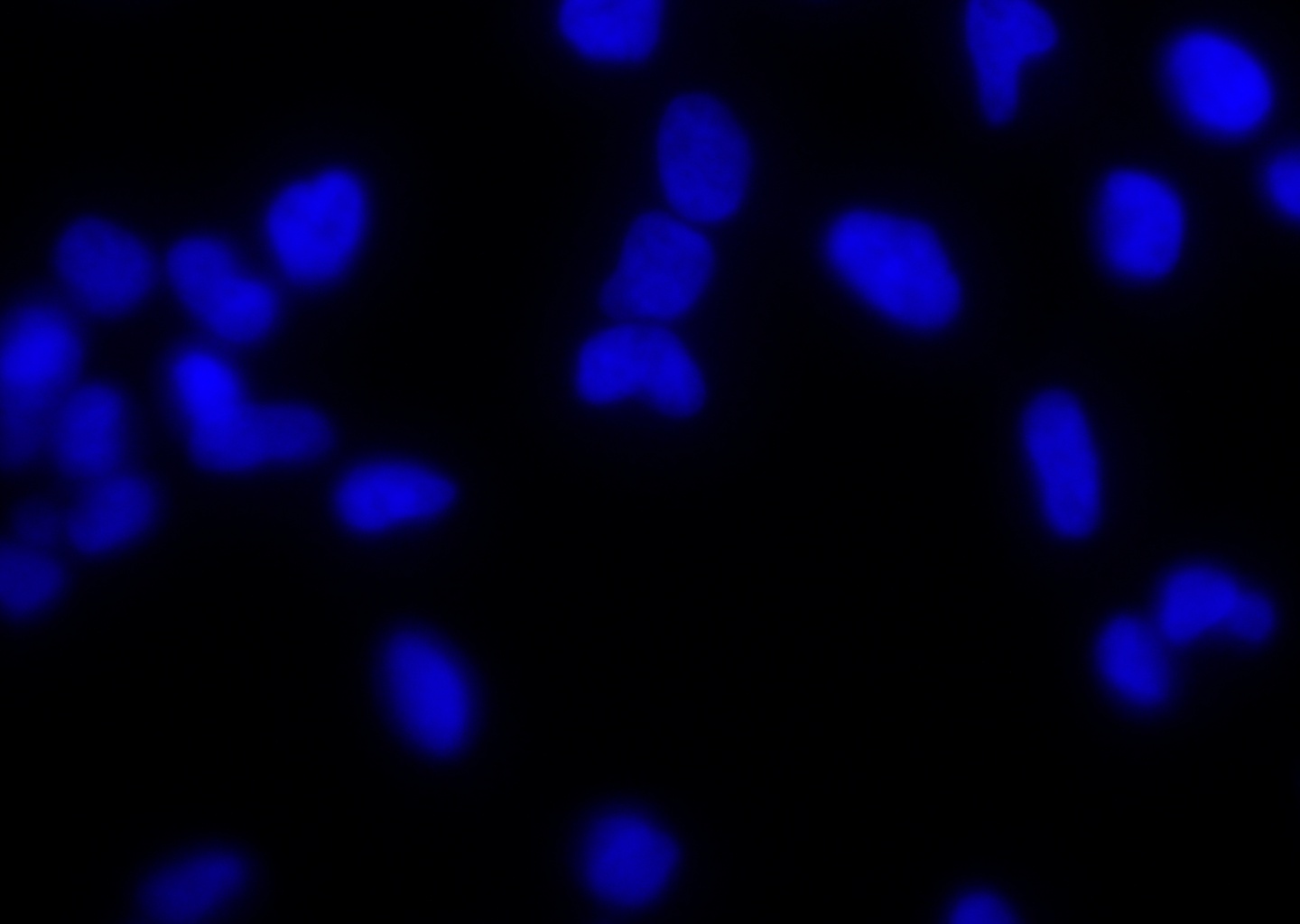

Supplement: Supplementary file 2 — Source Data Fig. 1 [file 44318_2024_35_MOESM2_ESM.zip › EMBOJ-2023-115792R2_SourceData_Fig1/Fig1C/R2/WT MOCK/DAPI.jpg]

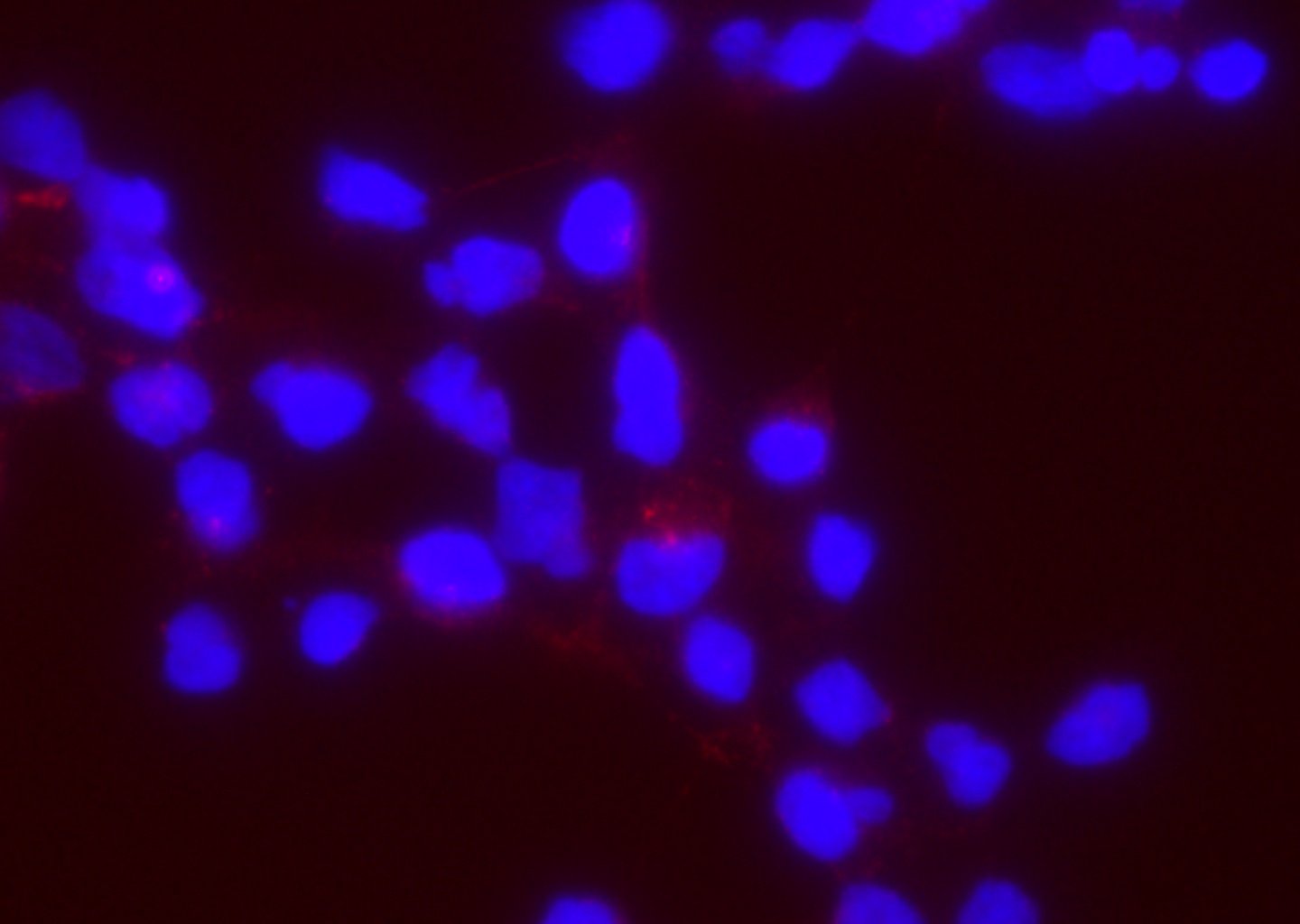

Supplement: Supplementary file 2 — Source Data Fig. 1 [file 44318_2024_35_MOESM2_ESM.zip › EMBOJ-2023-115792R2_SourceData_Fig1/Fig1C/R2/N1 SINV-GFP/N1 SINV-GFP merge.jpg]

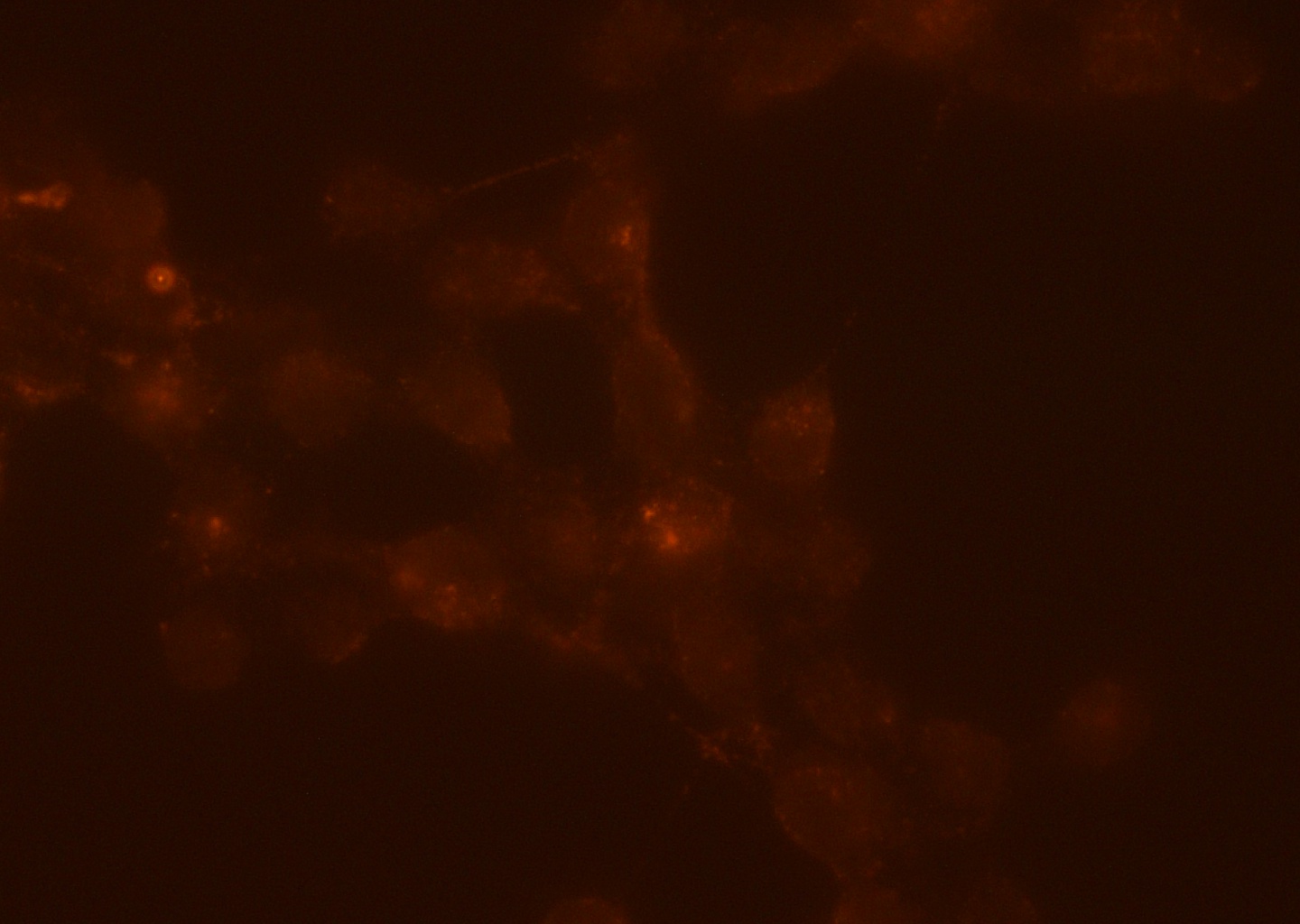

Supplement: Supplementary file 2 — Source Data Fig. 1 [file 44318_2024_35_MOESM2_ESM.zip › EMBOJ-2023-115792R2_SourceData_Fig1/Fig1C/R2/N1 SINV-GFP/J2.jpg]

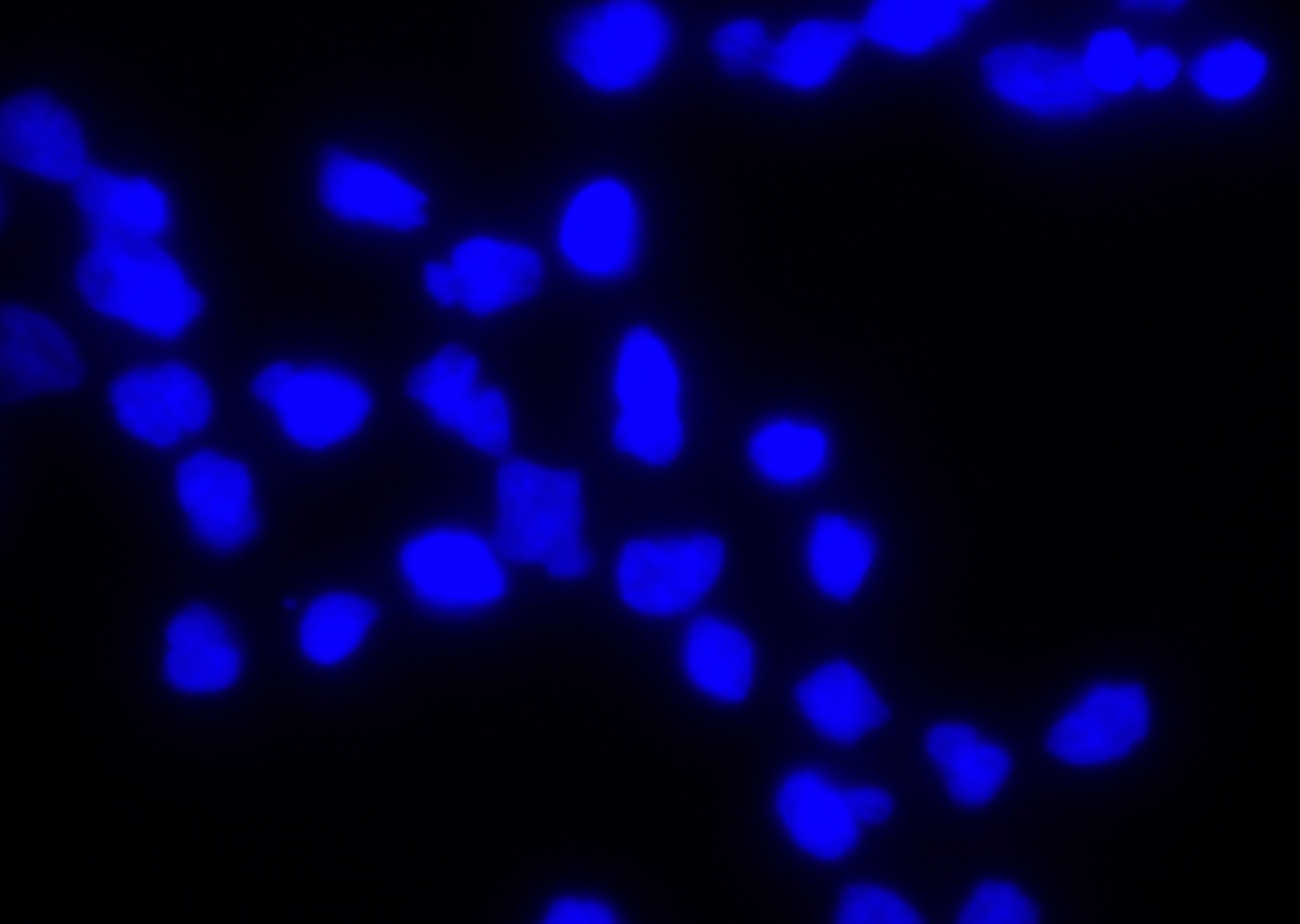

Supplement: Supplementary file 2 — Source Data Fig. 1 [file 44318_2024_35_MOESM2_ESM.zip › EMBOJ-2023-115792R2_SourceData_Fig1/Fig1C/R2/N1 SINV-GFP/DAPI.jpg]

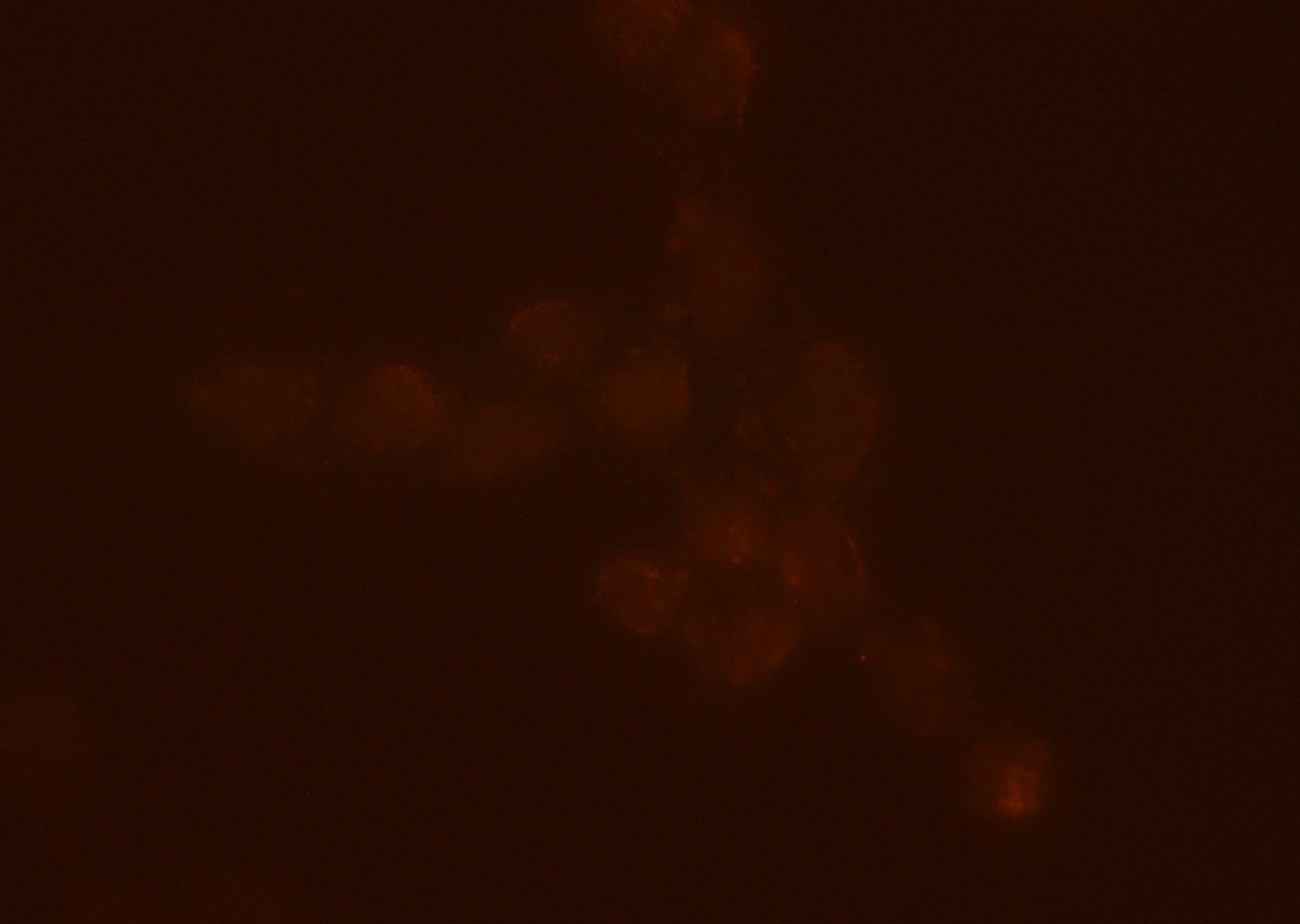

Supplement: Supplementary file 2 — Source Data Fig. 1 [file 44318_2024_35_MOESM2_ESM.zip › EMBOJ-2023-115792R2_SourceData_Fig1/Fig1C/R2/N1 MOCK/J2.jpg]

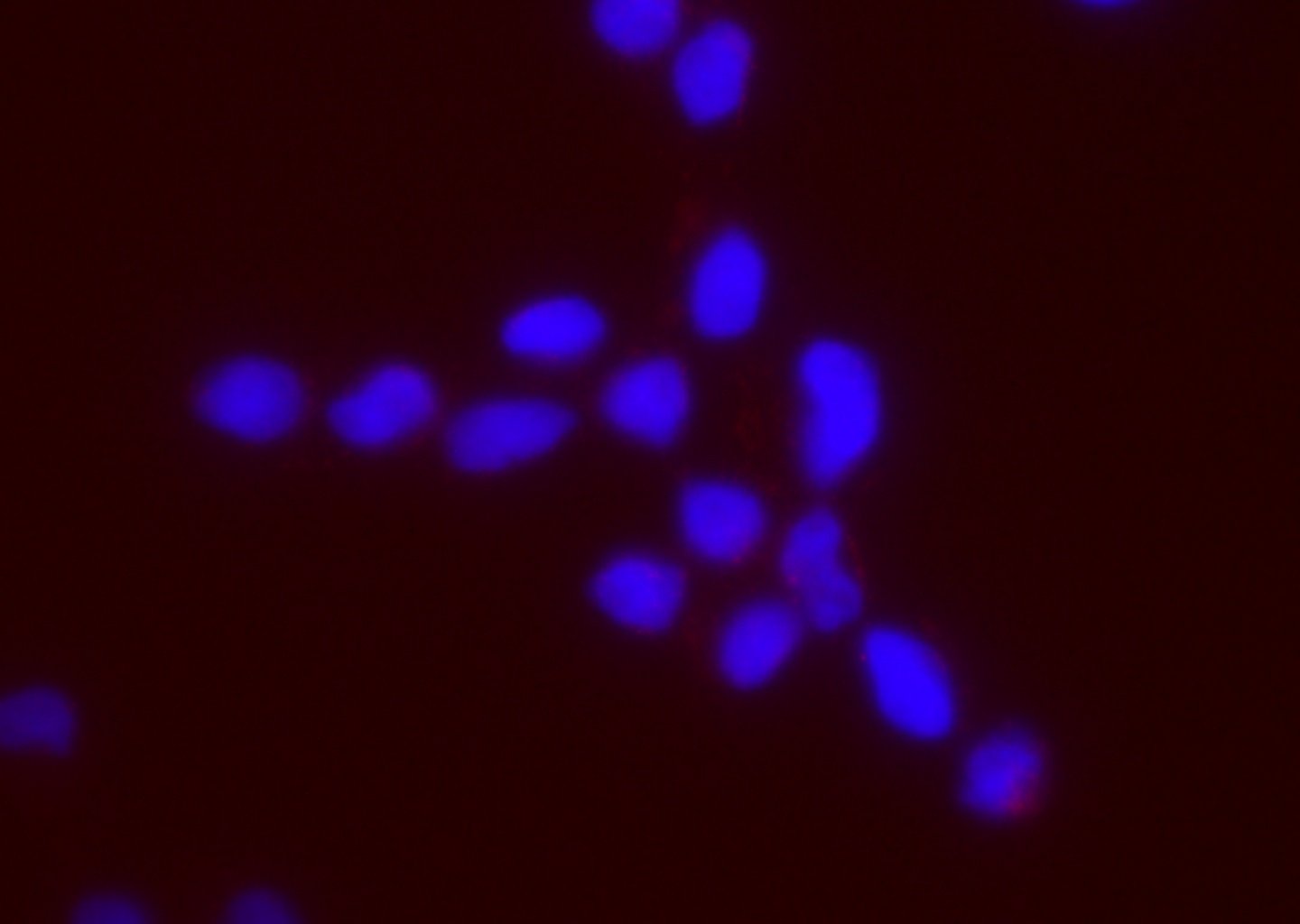

Supplement: Supplementary file 2 — Source Data Fig. 1 [file 44318_2024_35_MOESM2_ESM.zip › EMBOJ-2023-115792R2_SourceData_Fig1/Fig1C/R2/N1 MOCK/N1 MOCK merge.jpg]

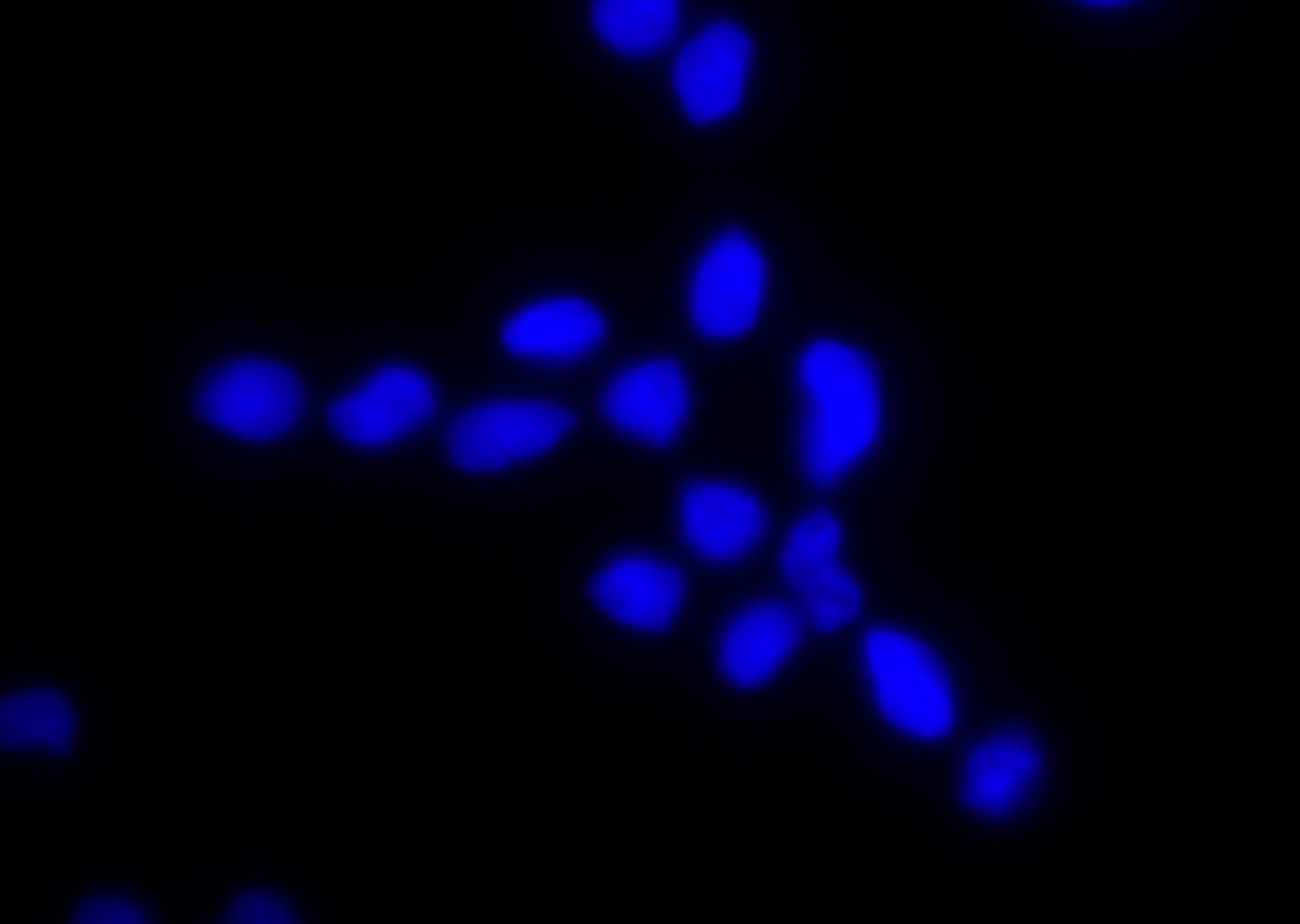

Supplement: Supplementary file 2 — Source Data Fig. 1 [file 44318_2024_35_MOESM2_ESM.zip › EMBOJ-2023-115792R2_SourceData_Fig1/Fig1C/R2/N1 MOCK/DAPI.jpg]

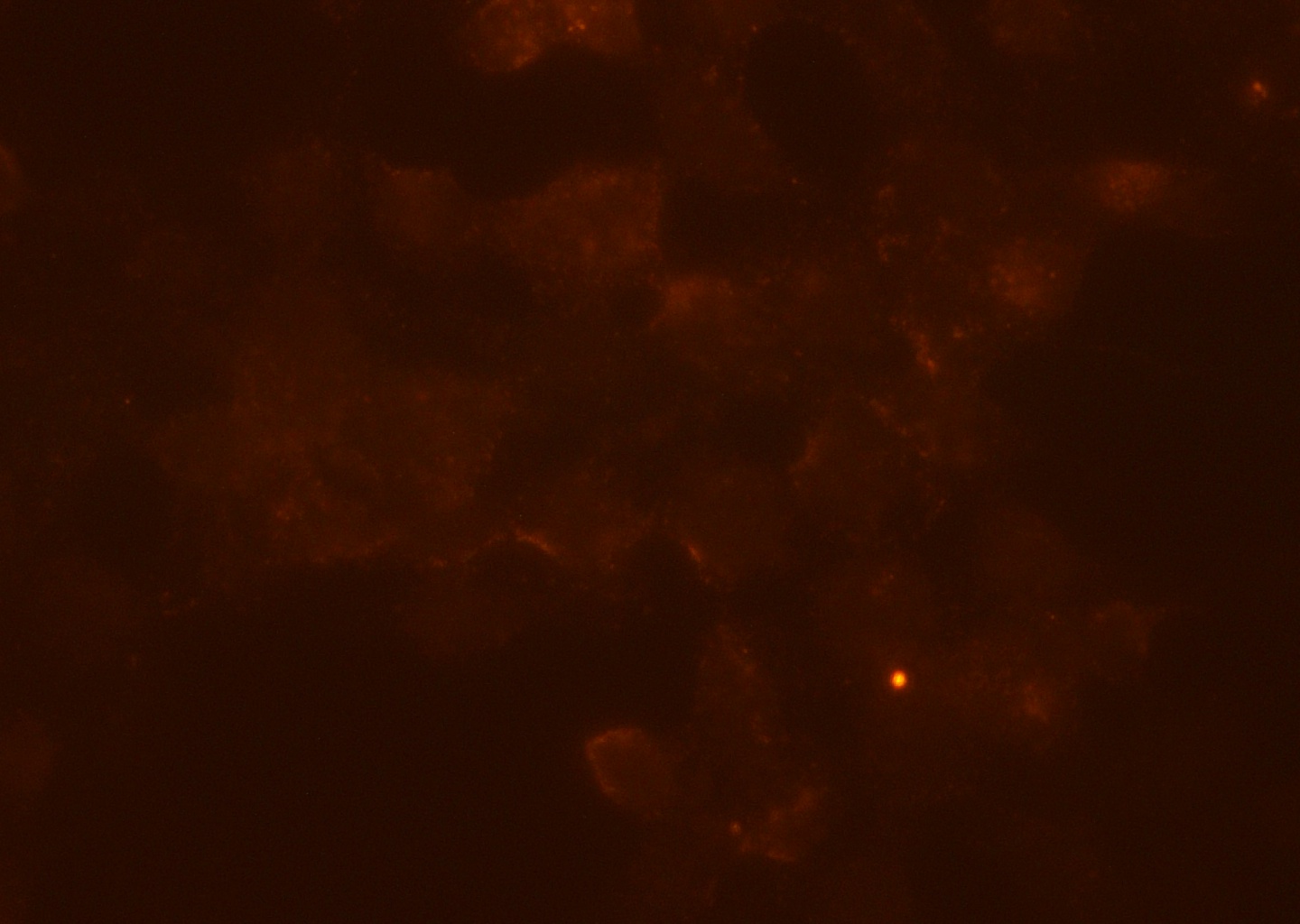

Supplement: Supplementary file 2 — Source Data Fig. 1 [file 44318_2024_35_MOESM2_ESM.zip › EMBOJ-2023-115792R2_SourceData_Fig1/Fig1C/R2/N1 SINV WT /J2.jpg]

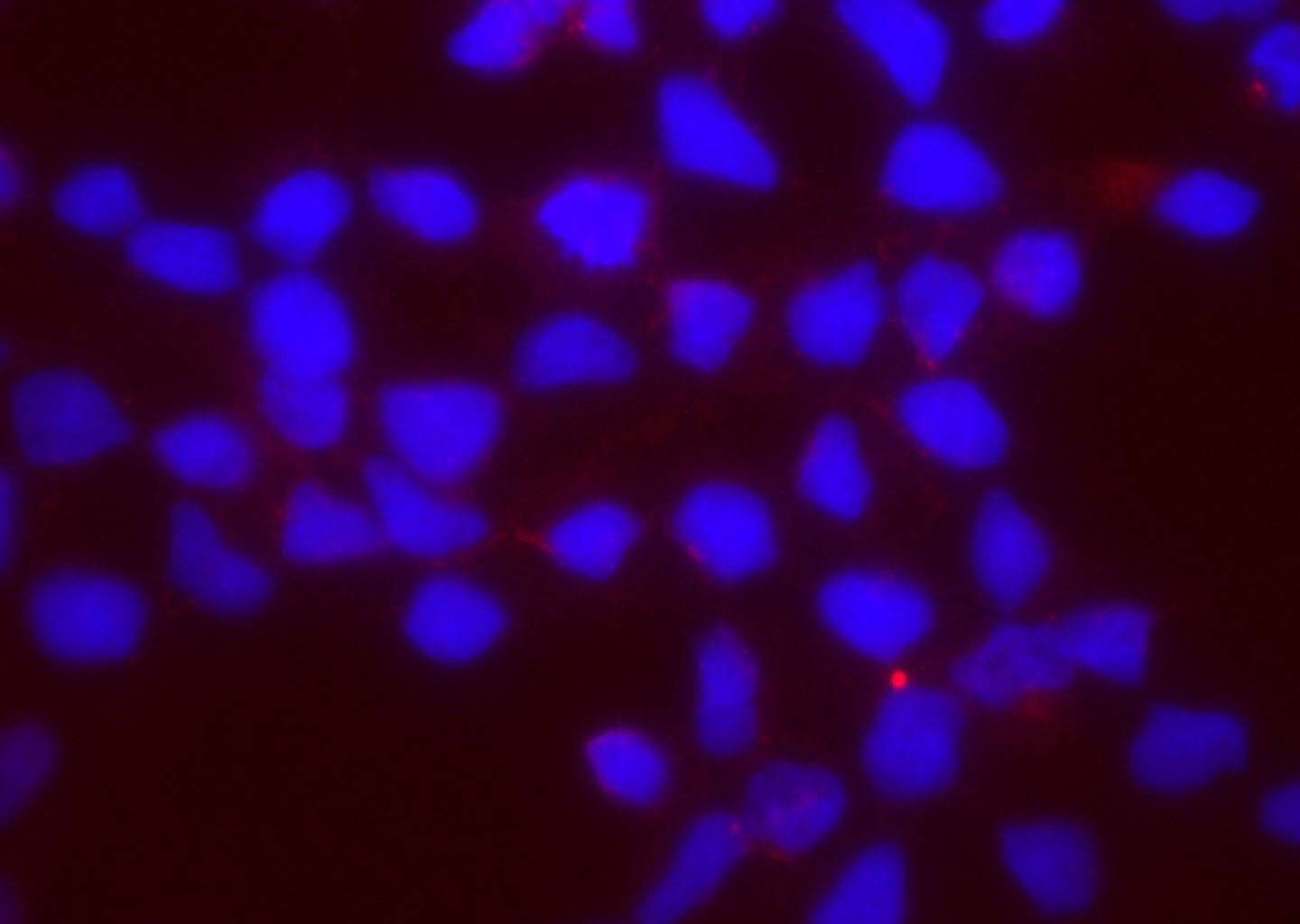

Supplement: Supplementary file 2 — Source Data Fig. 1 [file 44318_2024_35_MOESM2_ESM.zip › EMBOJ-2023-115792R2_SourceData_Fig1/Fig1C/R2/N1 SINV WT /N1 SINV WT merge.jpg]

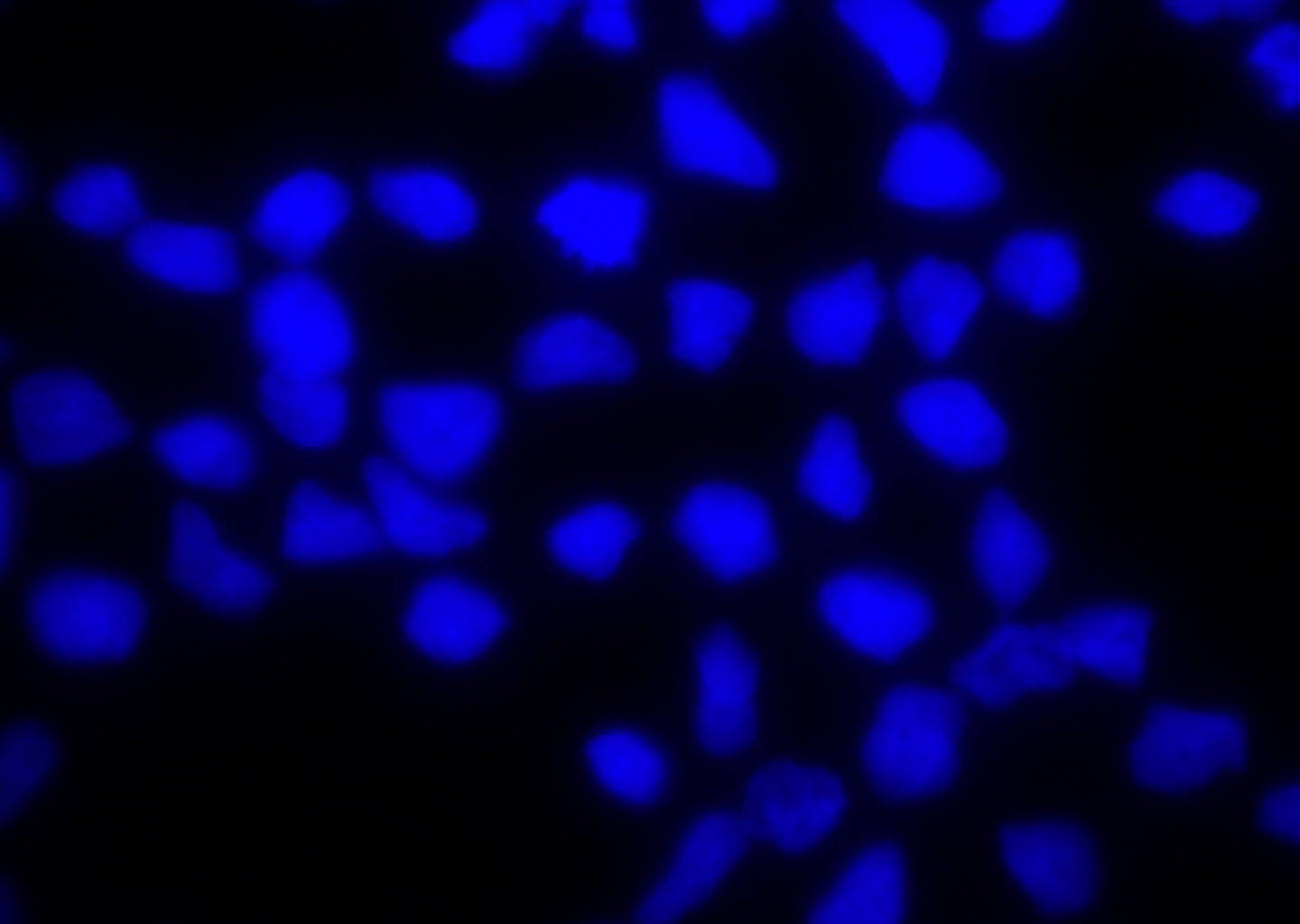

Supplement: Supplementary file 2 — Source Data Fig. 1 [file 44318_2024_35_MOESM2_ESM.zip › EMBOJ-2023-115792R2_SourceData_Fig1/Fig1C/R2/N1 SINV WT /DAPI.jpg]

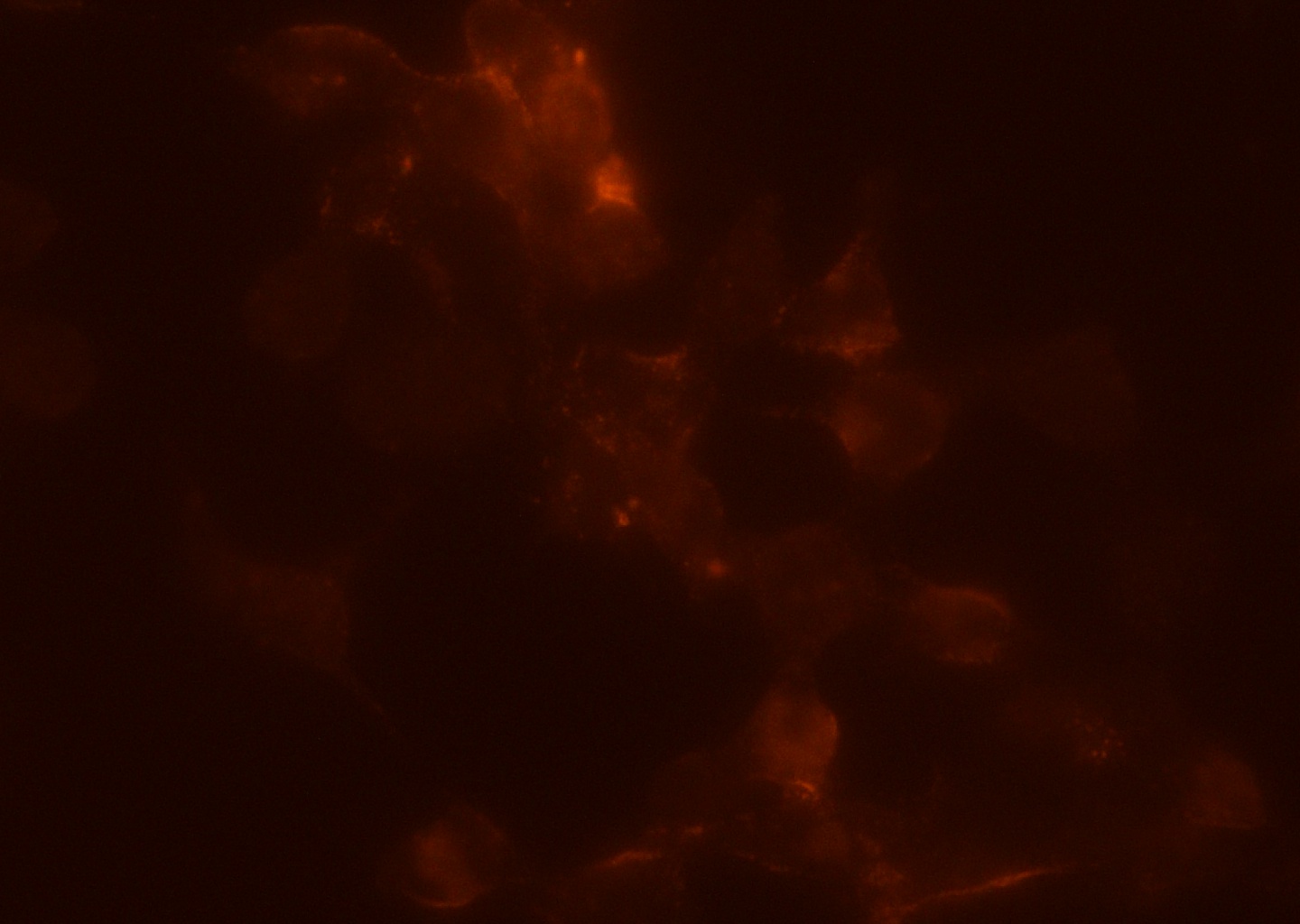

Supplement: Supplementary file 2 — Source Data Fig. 1 [file 44318_2024_35_MOESM2_ESM.zip › EMBOJ-2023-115792R2_SourceData_Fig1/Fig1C/R2/N1 SINV 2A-GFP/J2.jpg]

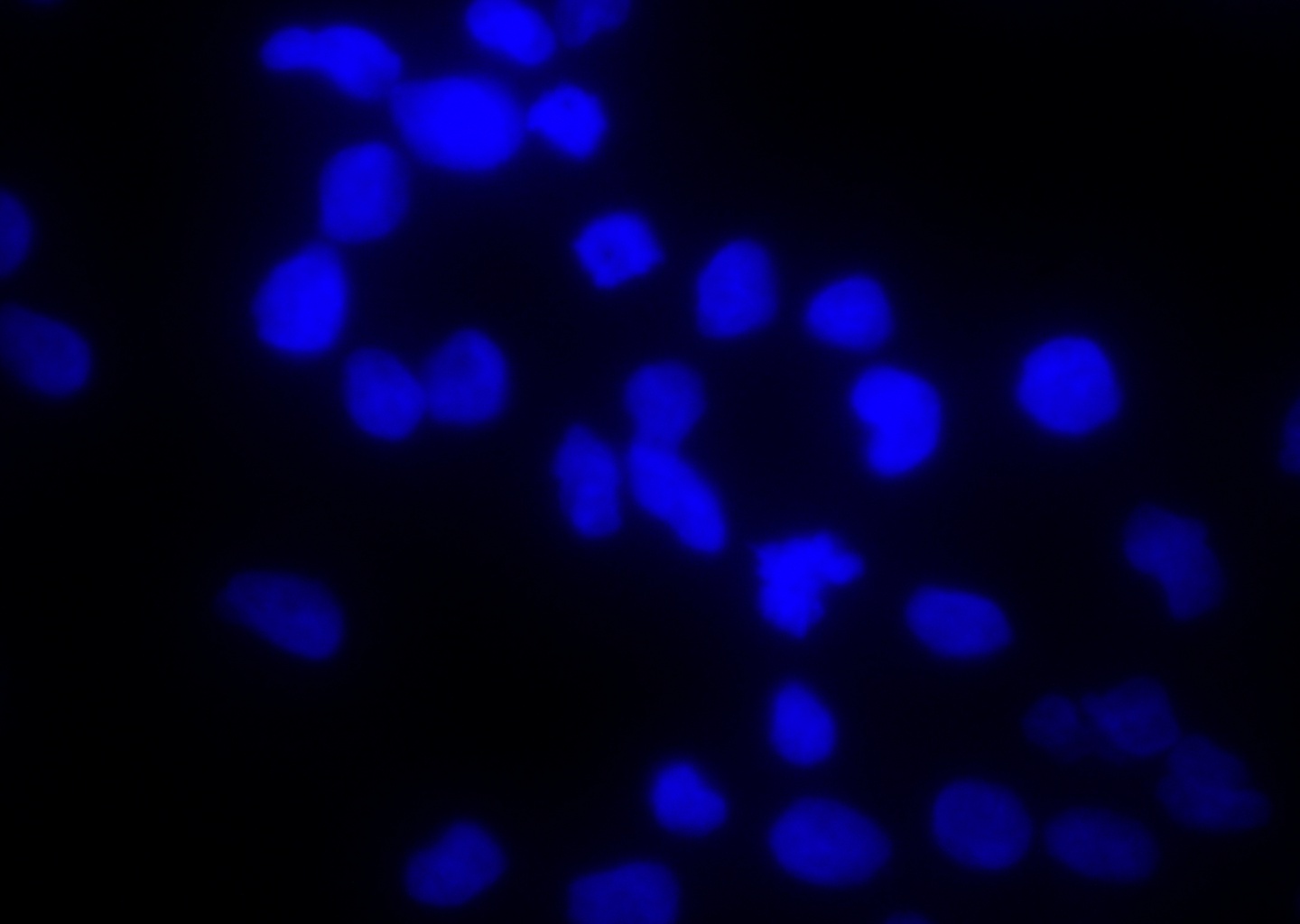

Supplement: Supplementary file 2 — Source Data Fig. 1 [file 44318_2024_35_MOESM2_ESM.zip › EMBOJ-2023-115792R2_SourceData_Fig1/Fig1C/R2/N1 SINV 2A-GFP/DAPI.jpg]

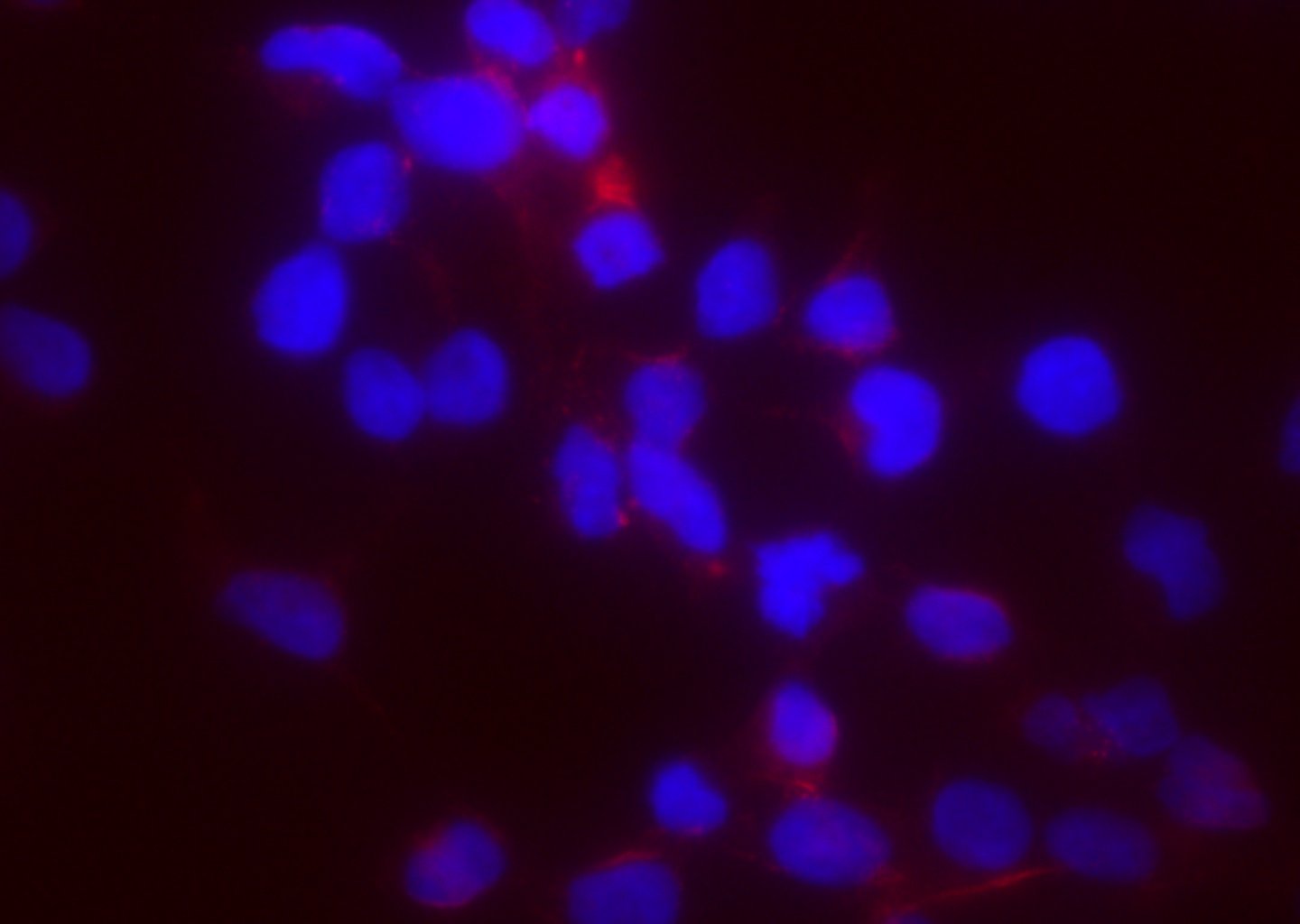

Supplement: Supplementary file 2 — Source Data Fig. 1 [file 44318_2024_35_MOESM2_ESM.zip › EMBOJ-2023-115792R2_SourceData_Fig1/Fig1C/R2/N1 SINV 2A-GFP/N1 2A-GFP merge.jpg]

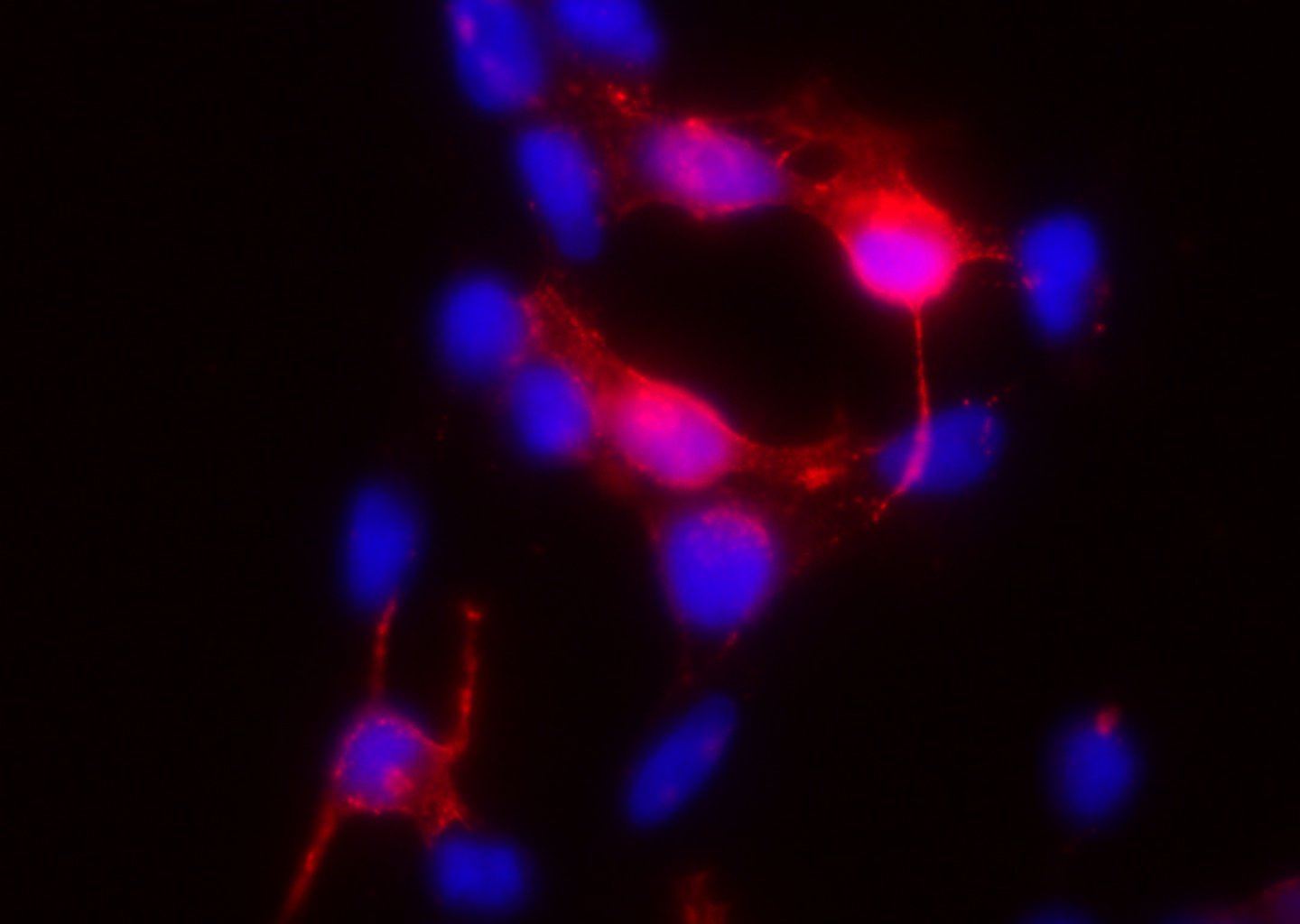

Supplement: Supplementary file 2 — Source Data Fig. 1 [file 44318_2024_35_MOESM2_ESM.zip › EMBOJ-2023-115792R2_SourceData_Fig1/Fig1C/R2/WT SINV-GFP/WT SINV-GFP merge.jpg]

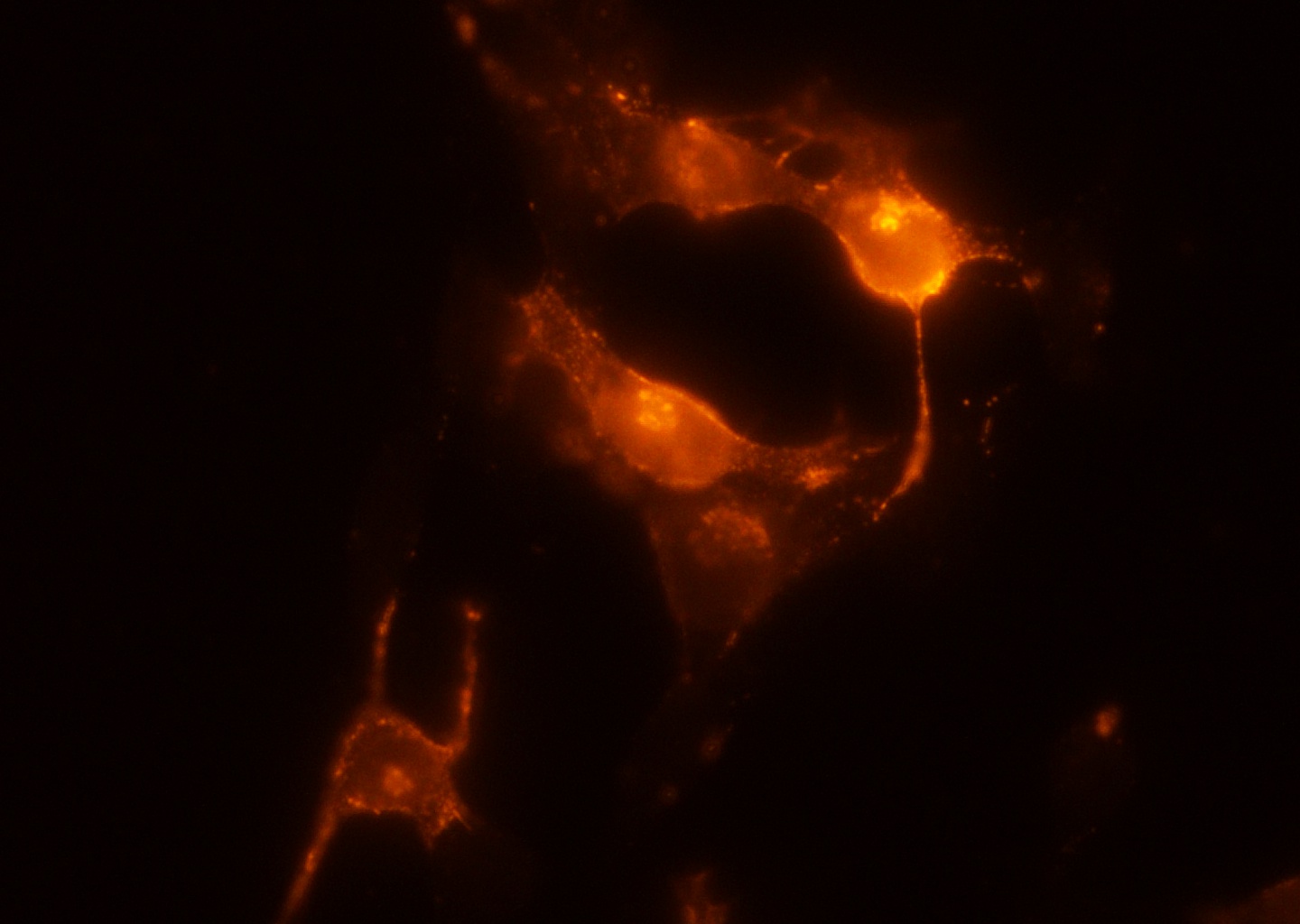

Supplement: Supplementary file 2 — Source Data Fig. 1 [file 44318_2024_35_MOESM2_ESM.zip › EMBOJ-2023-115792R2_SourceData_Fig1/Fig1C/R2/WT SINV-GFP/J2.jpg]

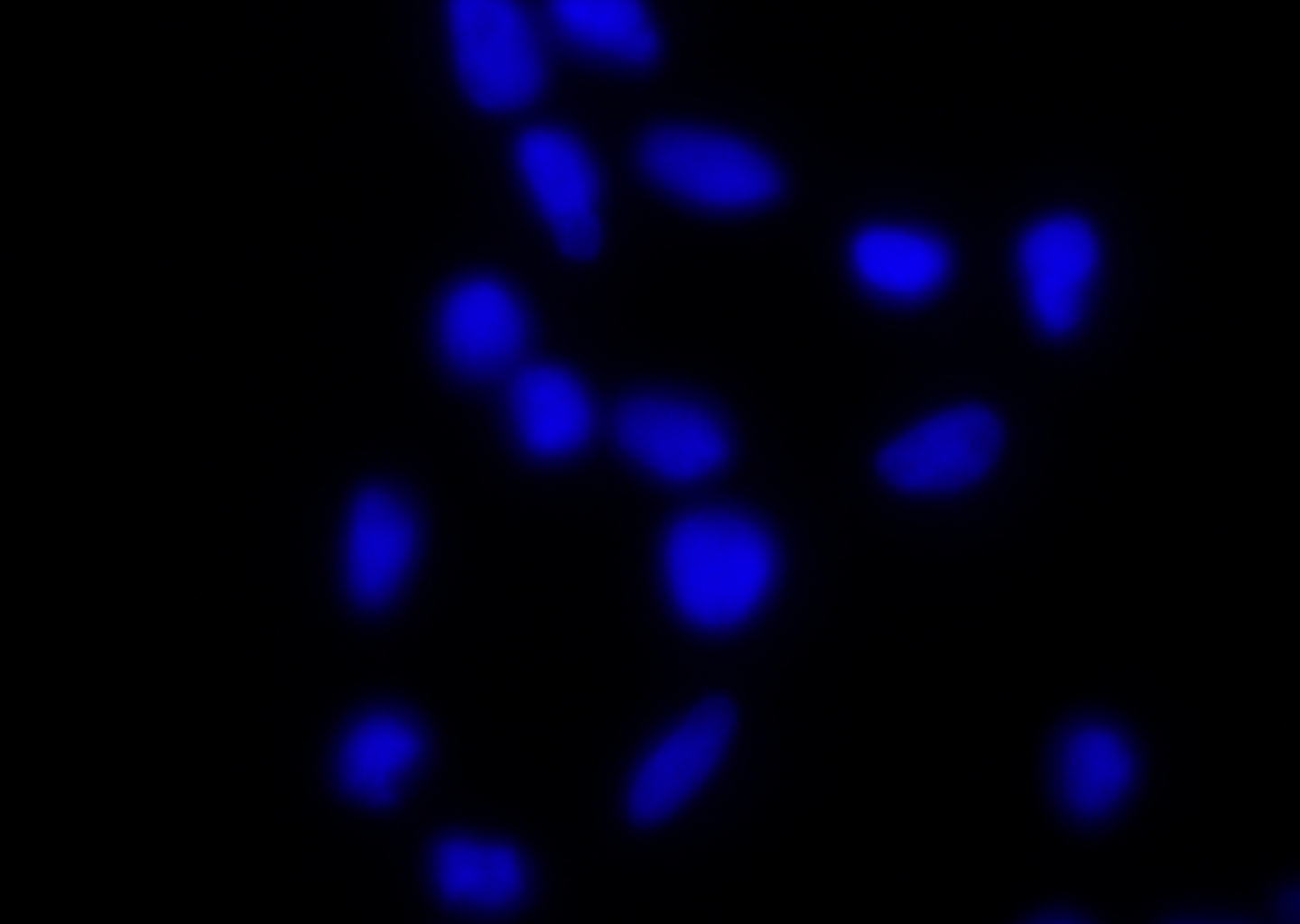

Supplement: Supplementary file 2 — Source Data Fig. 1 [file 44318_2024_35_MOESM2_ESM.zip › EMBOJ-2023-115792R2_SourceData_Fig1/Fig1C/R2/WT SINV-GFP/DAPI.jpg]

D

**Replicate 1**

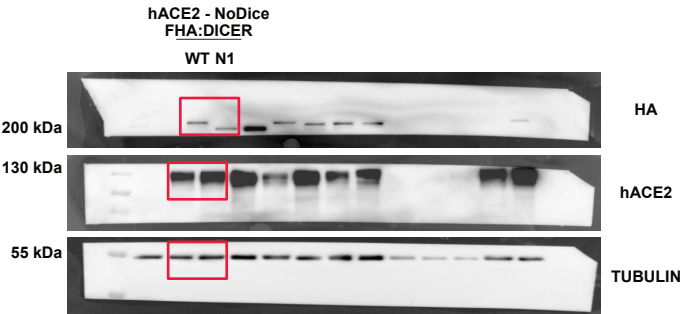

E

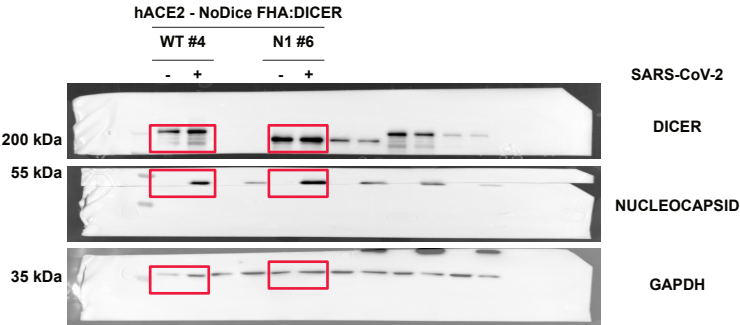

**Replicate 2**

**Replicate 3**

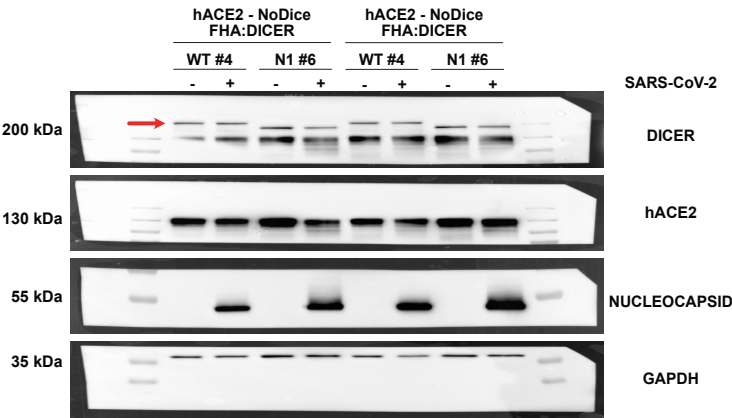

Replicate 1

Replicate 2

Replicate 3

A

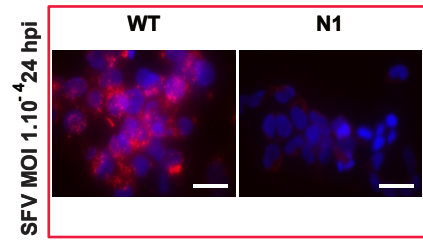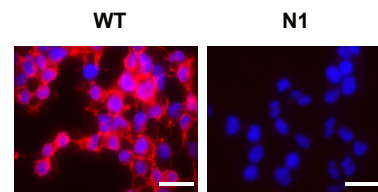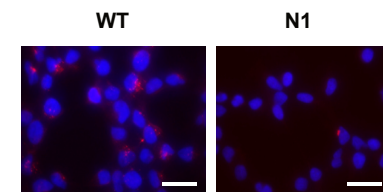

B

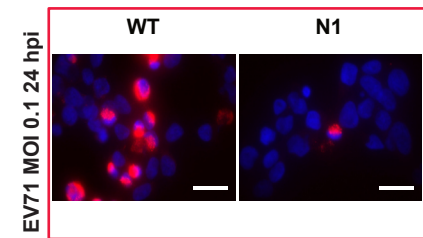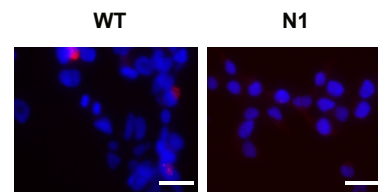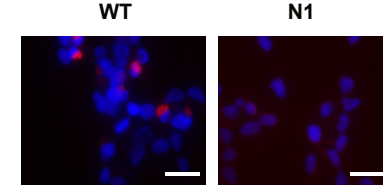

C

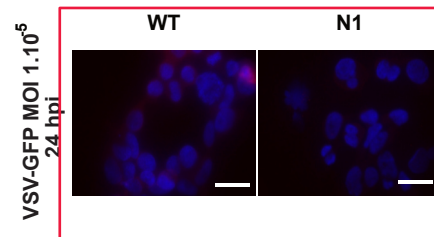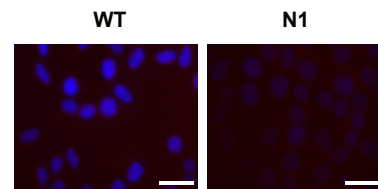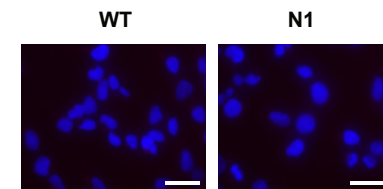

Supplement: Supplementary file 3 — Source Data Fig. 2 [file 44318_2024_35_MOESM3_ESM.zip › EMBOJ-2023-115792R2_SourceData_Fig2/Figure2.pdf]

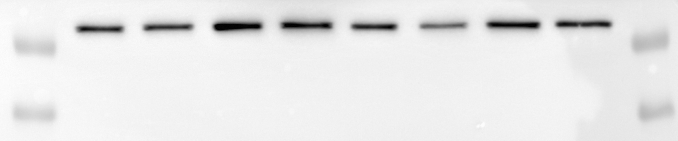

Supplement: Supplementary file 3 — Source Data Fig. 2 [file 44318_2024_35_MOESM3_ESM.zip › EMBOJ-2023-115792R2_SourceData_Fig2/Fig2D-E_western blot/R3/western GAPDH.tiff]

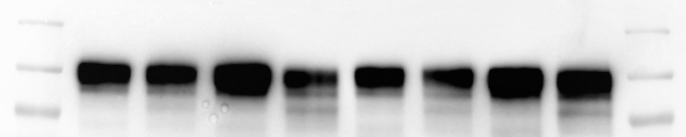

Supplement: Supplementary file 3 — Source Data Fig. 2 [file 44318_2024_35_MOESM3_ESM.zip › EMBOJ-2023-115792R2_SourceData_Fig2/Fig2D-E_western blot/R3/western ace2.tiff]

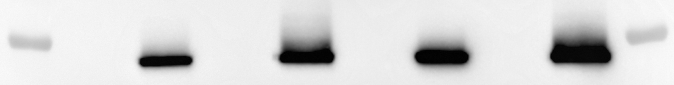

Supplement: Supplementary file 3 — Source Data Fig. 2 [file 44318_2024_35_MOESM3_ESM.zip › EMBOJ-2023-115792R2_SourceData_Fig2/Fig2D-E_western blot/R3/western nucleocapsid.tiff]

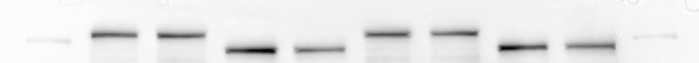

Supplement: Supplementary file 3 — Source Data Fig. 2 [file 44318_2024_35_MOESM3_ESM.zip › EMBOJ-2023-115792R2_SourceData_Fig2/Fig2D-E_western blot/R3/western dicer.tiff]

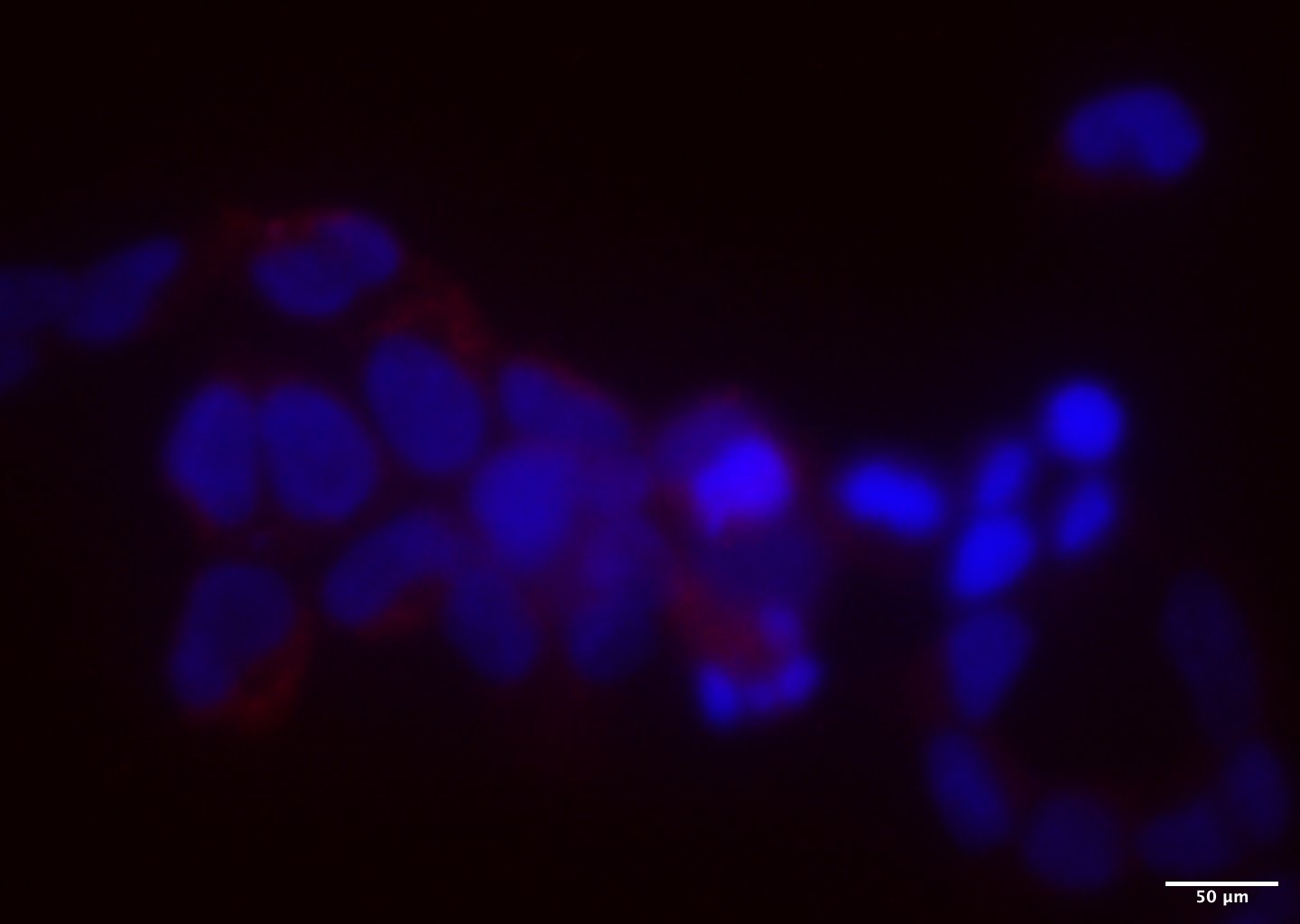

Supplement: Supplementary file 3 — Source Data Fig. 2 [file 44318_2024_35_MOESM3_ESM.zip › EMBOJ-2023-115792R2_SourceData_Fig2/Fig2A-B-C_microscopy/R1/N1 SFV R1/N1 SFV merge.jpg]

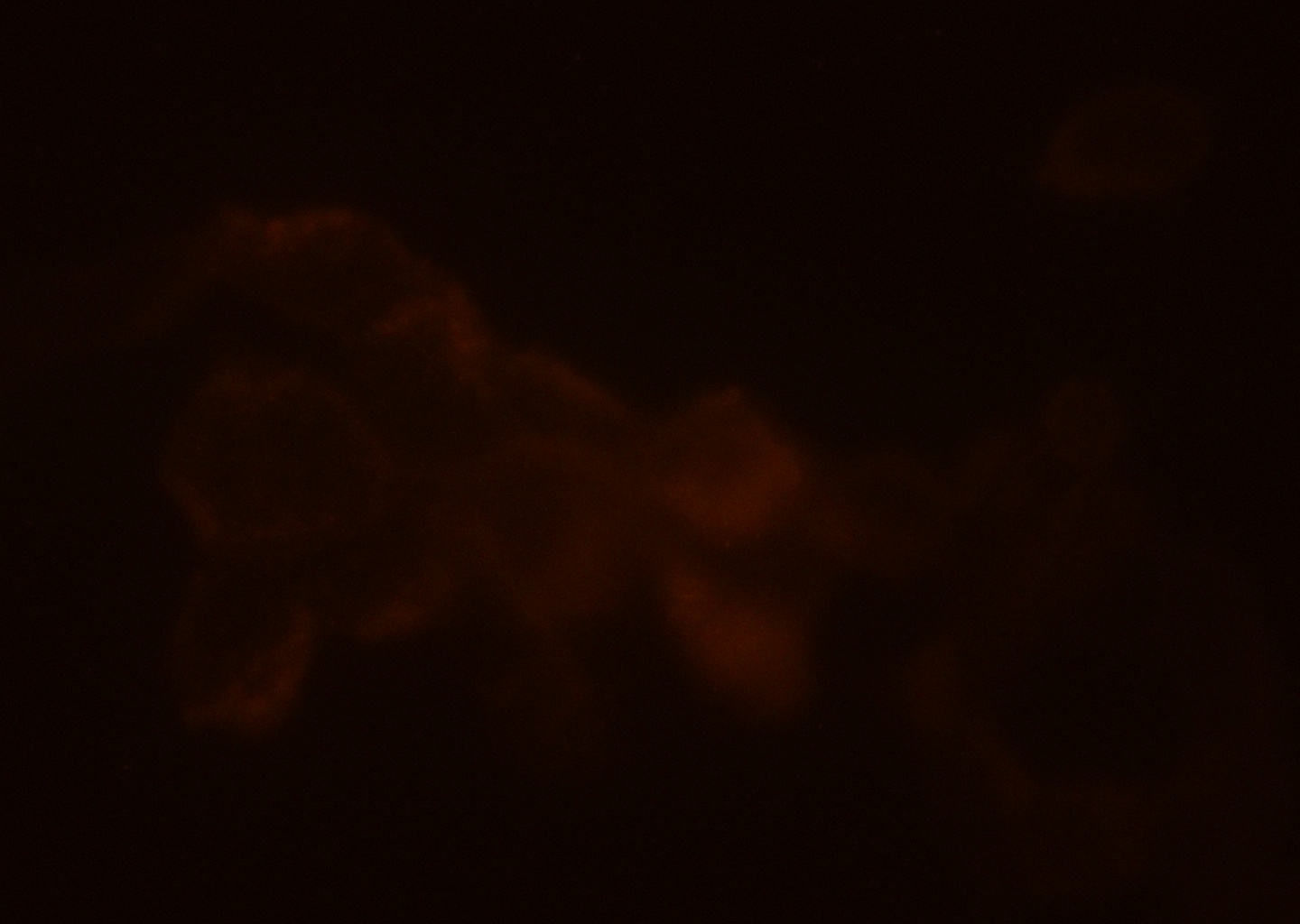

Supplement: Supplementary file 3 — Source Data Fig. 2 [file 44318_2024_35_MOESM3_ESM.zip › EMBOJ-2023-115792R2_SourceData_Fig2/Fig2A-B-C_microscopy/R1/N1 SFV R1/J2.jpg]

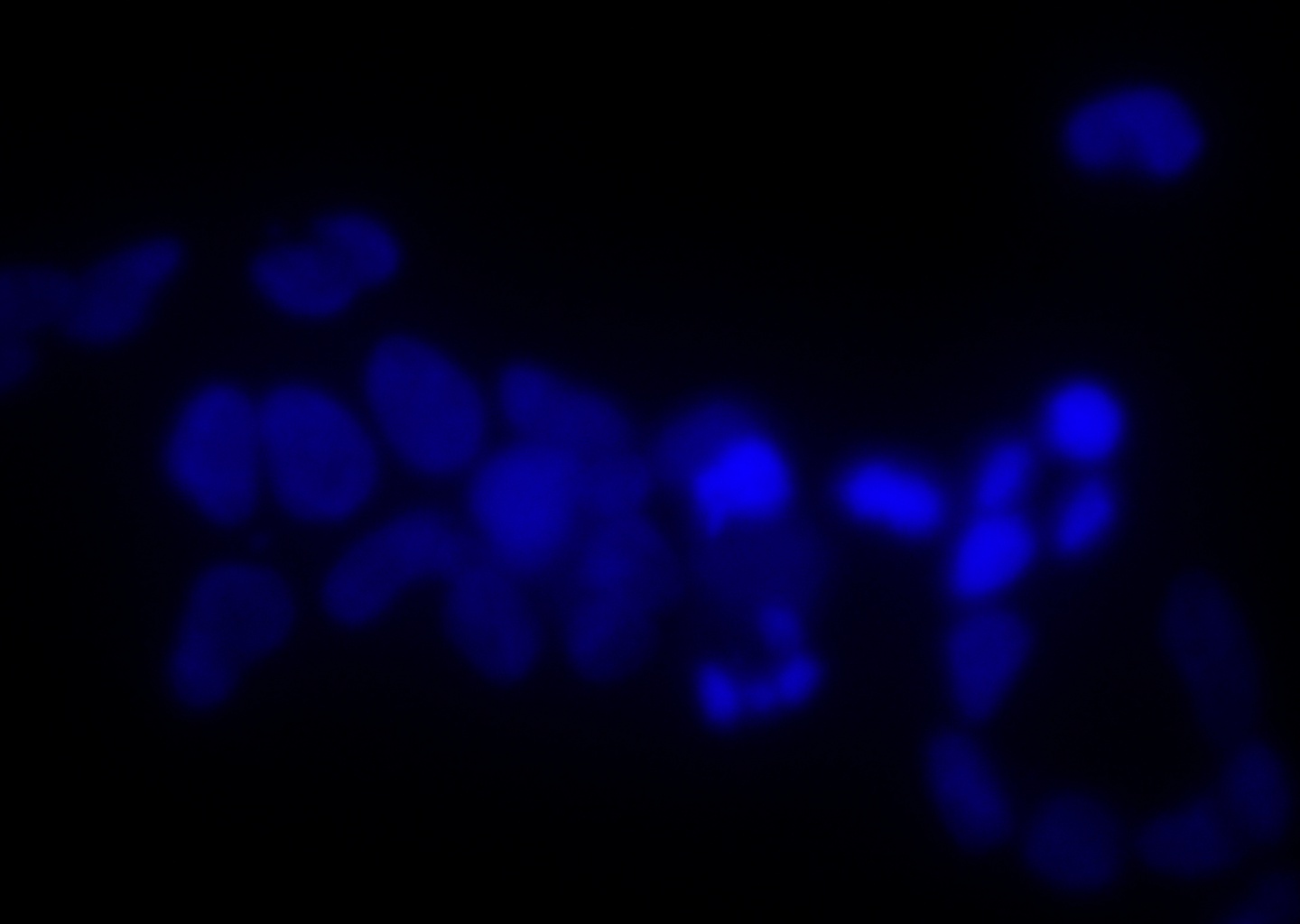

Supplement: Supplementary file 3 — Source Data Fig. 2 [file 44318_2024_35_MOESM3_ESM.zip › EMBOJ-2023-115792R2_SourceData_Fig2/Fig2A-B-C_microscopy/R1/N1 SFV R1/DAPI.jpg]

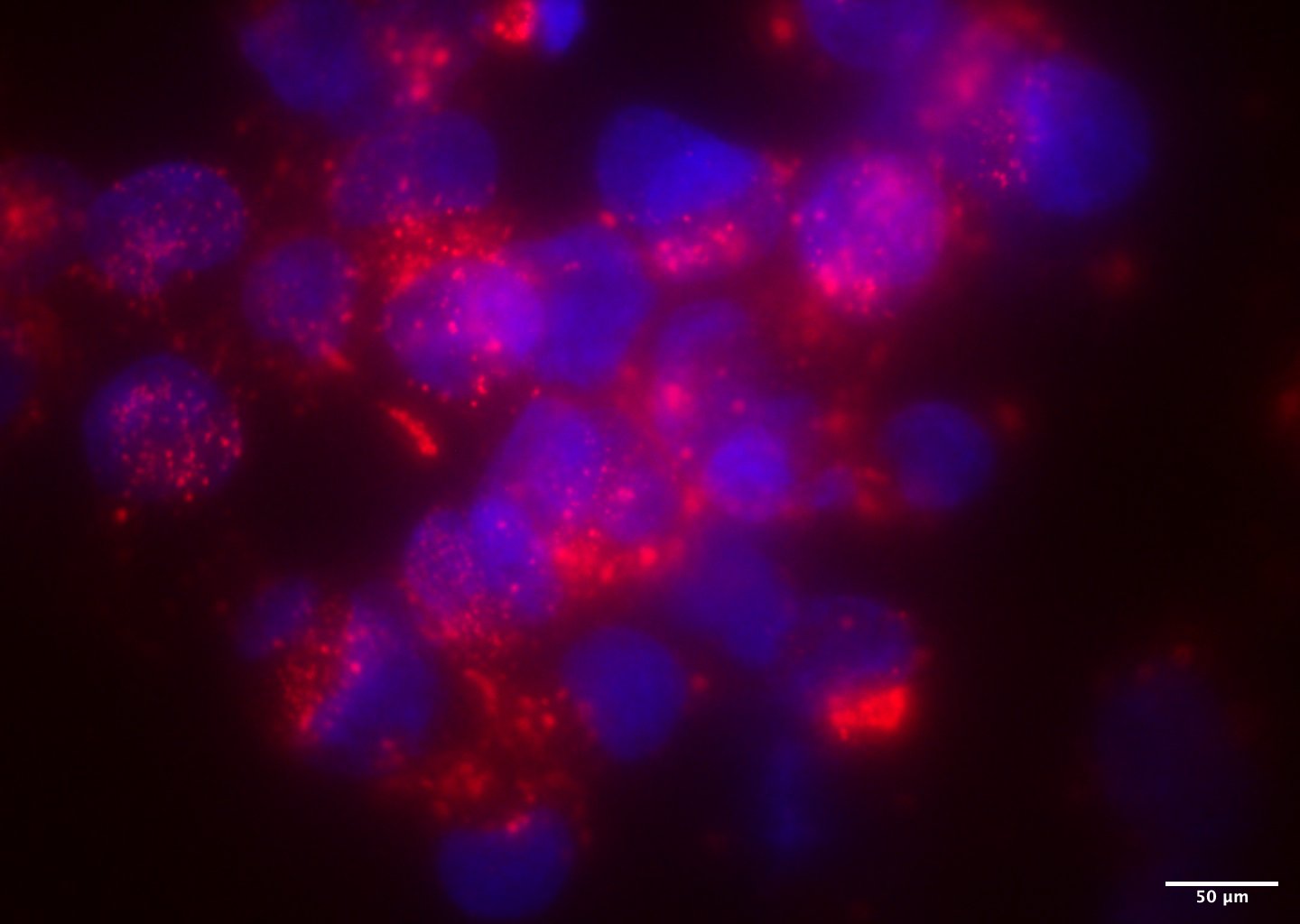

Supplement: Supplementary file 3 — Source Data Fig. 2 [file 44318_2024_35_MOESM3_ESM.zip › EMBOJ-2023-115792R2_SourceData_Fig2/Fig2A-B-C_microscopy/R1/WT SFV R1/WT SFV merge.jpg]

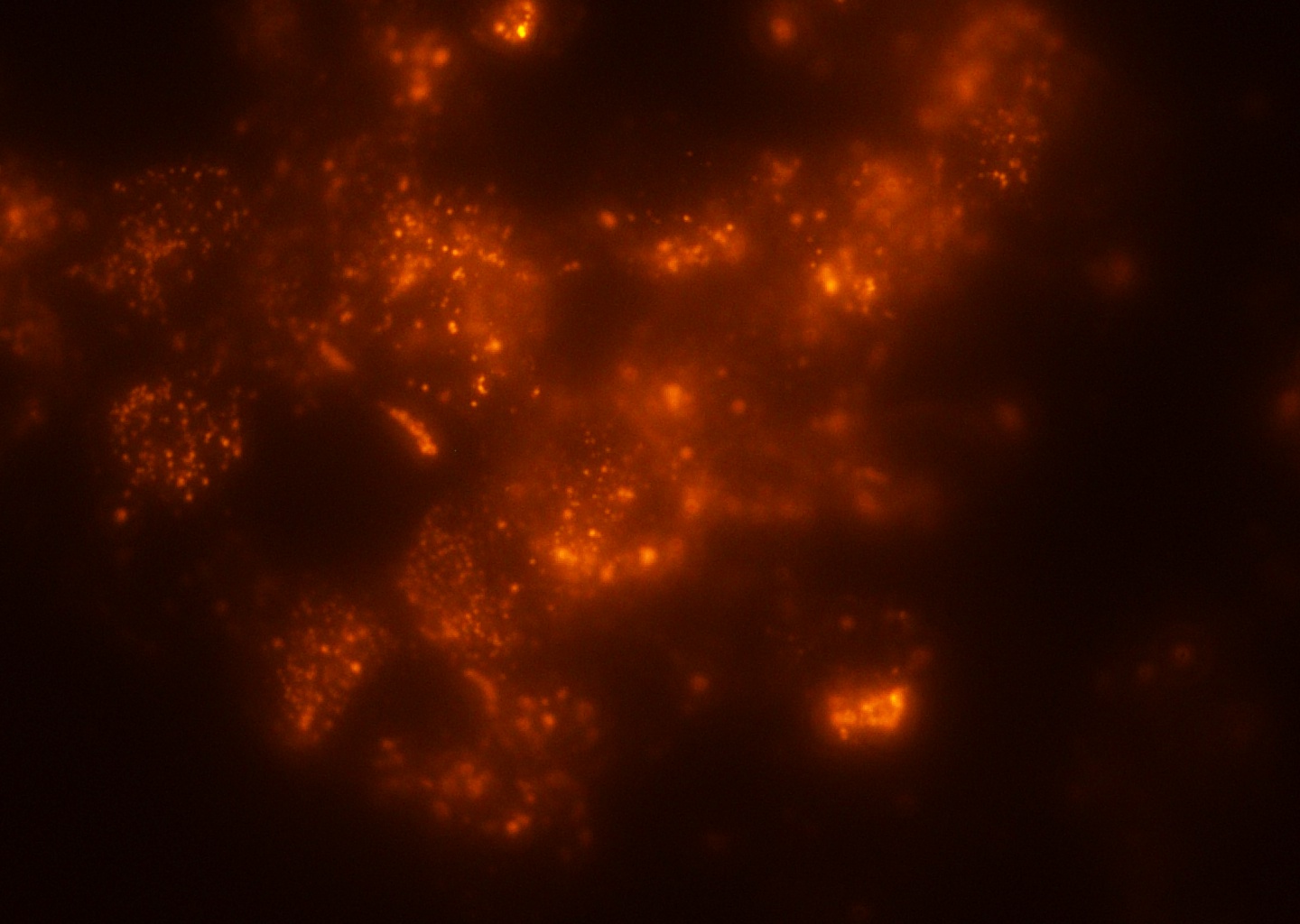

Supplement: Supplementary file 3 — Source Data Fig. 2 [file 44318_2024_35_MOESM3_ESM.zip › EMBOJ-2023-115792R2_SourceData_Fig2/Fig2A-B-C_microscopy/R1/WT SFV R1/J2.jpg]

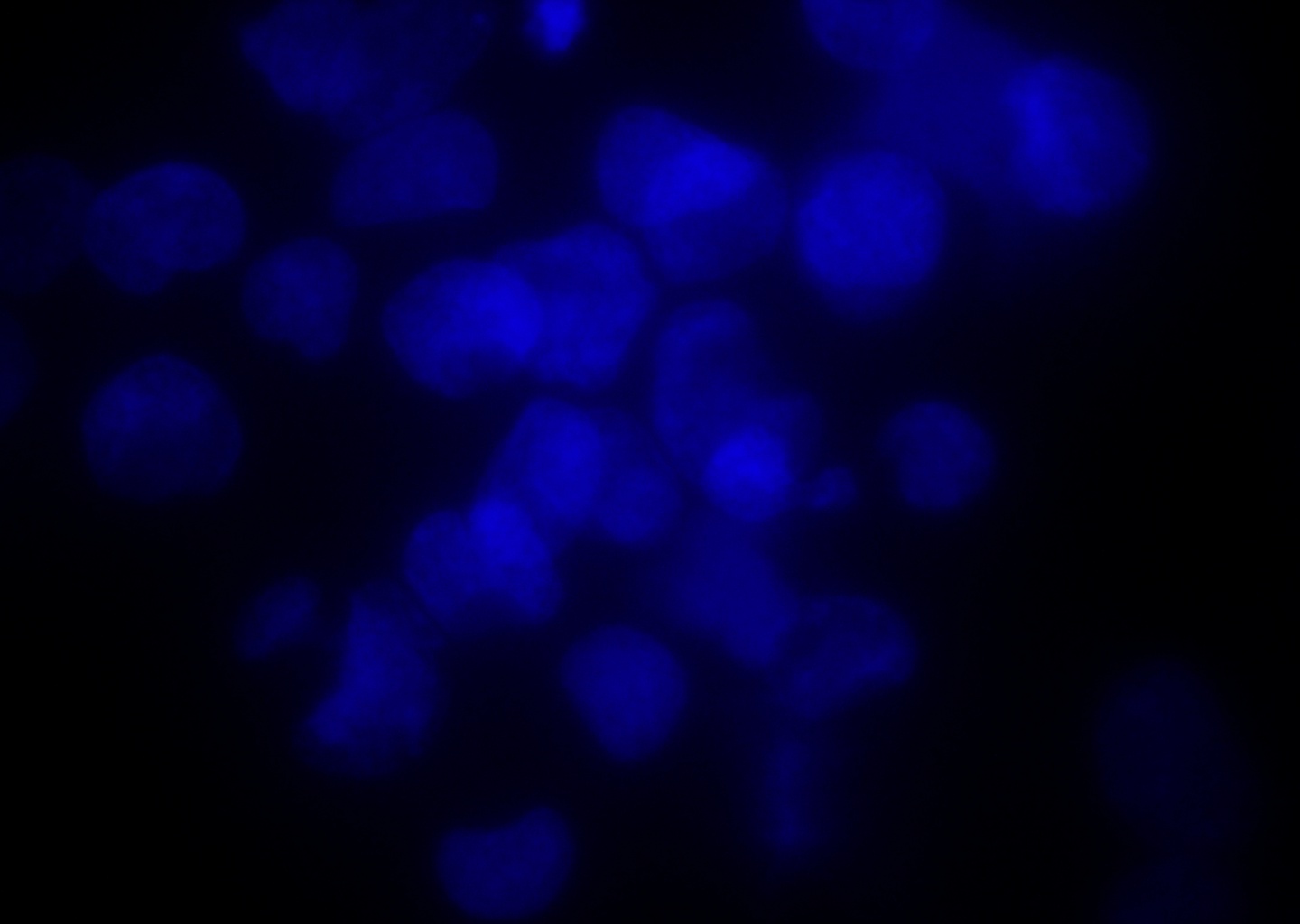

Supplement: Supplementary file 3 — Source Data Fig. 2 [file 44318_2024_35_MOESM3_ESM.zip › EMBOJ-2023-115792R2_SourceData_Fig2/Fig2A-B-C_microscopy/R1/WT SFV R1/DAPi.jpg]

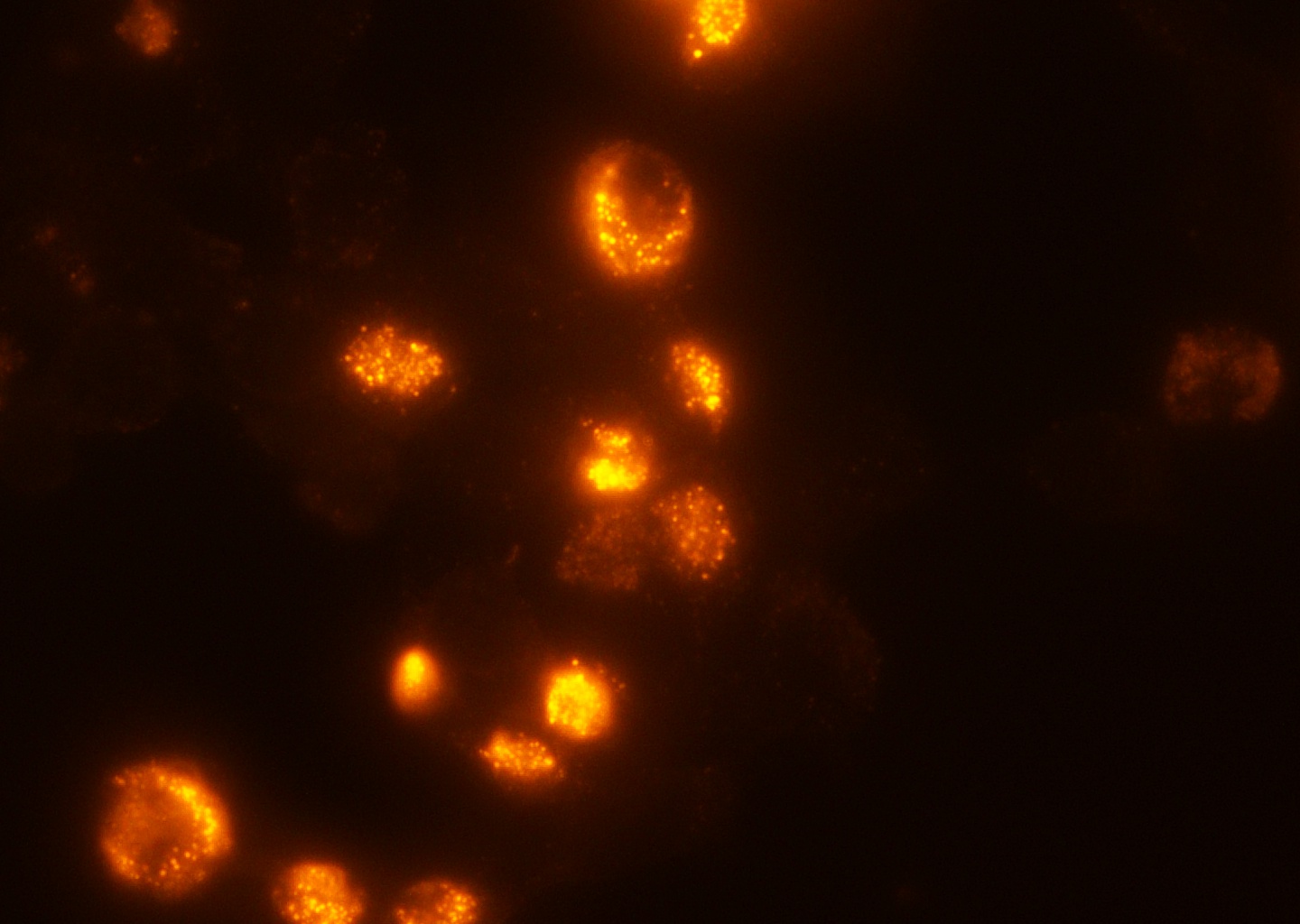

Supplement: Supplementary file 3 — Source Data Fig. 2 [file 44318_2024_35_MOESM3_ESM.zip › EMBOJ-2023-115792R2_SourceData_Fig2/Fig2A-B-C_microscopy/R1/WT EV R1/J2.jpg]

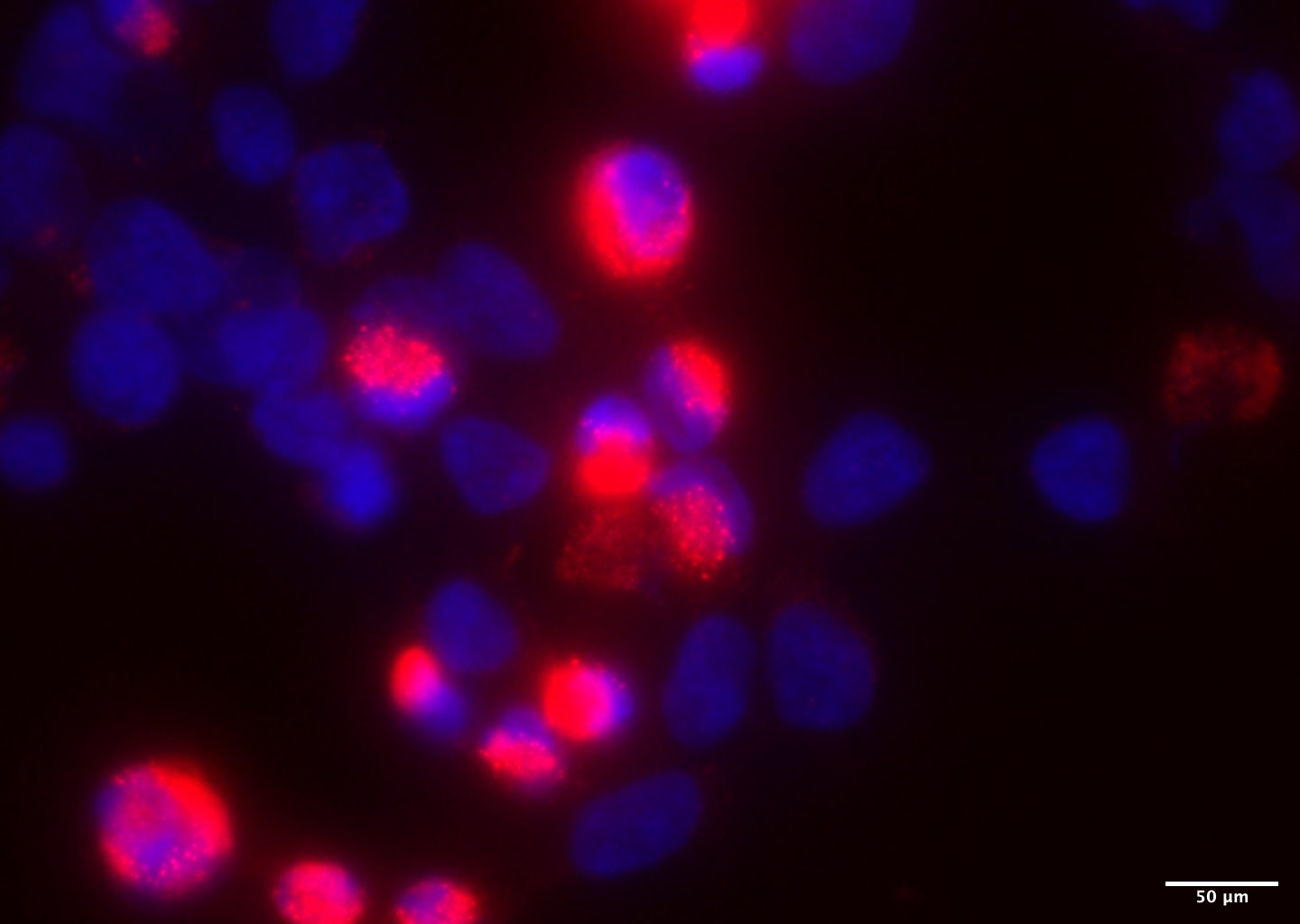

Supplement: Supplementary file 3 — Source Data Fig. 2 [file 44318_2024_35_MOESM3_ESM.zip › EMBOJ-2023-115792R2_SourceData_Fig2/Fig2A-B-C_microscopy/R1/WT EV R1/WT EV merge.jpg]

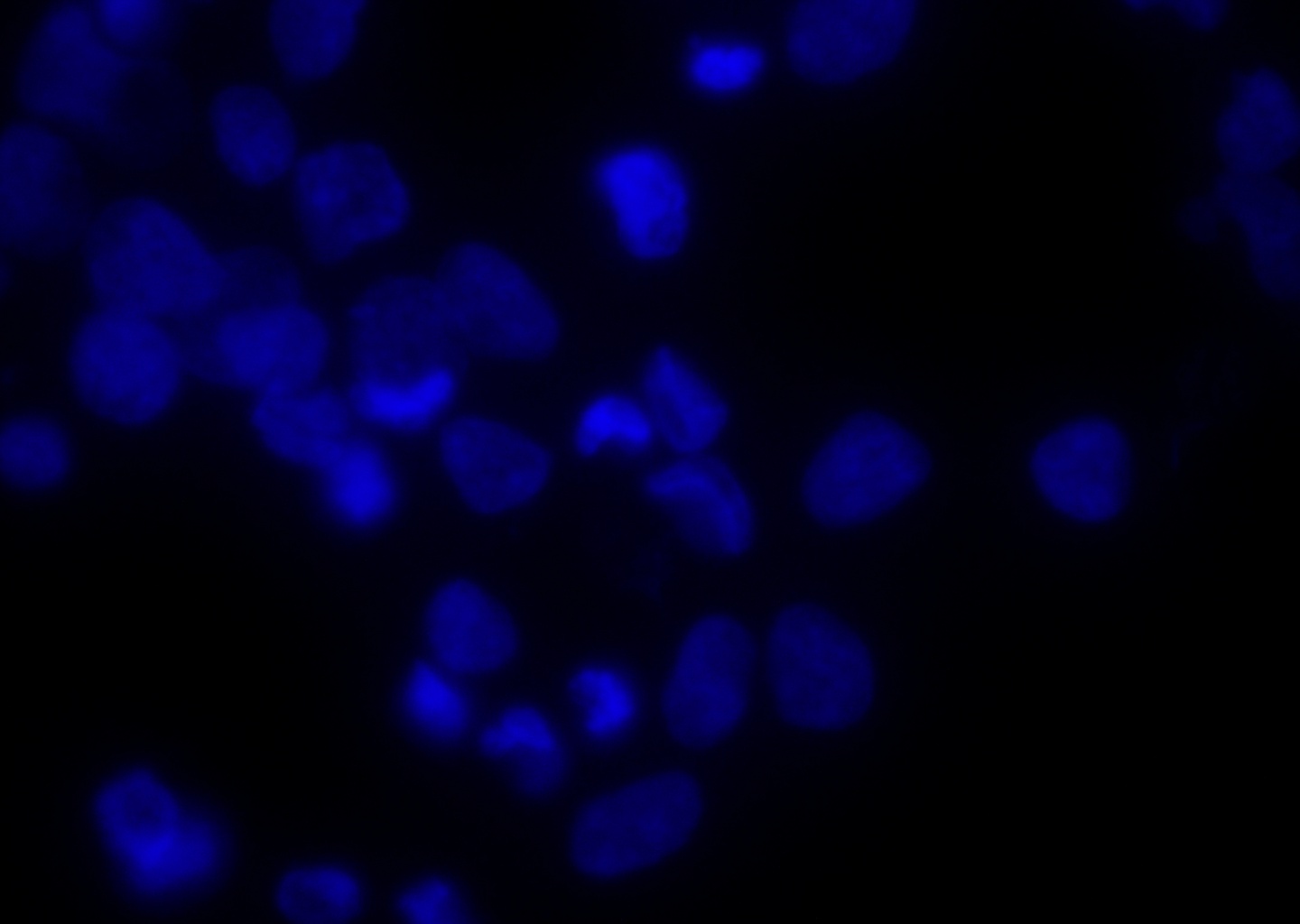

Supplement: Supplementary file 3 — Source Data Fig. 2 [file 44318_2024_35_MOESM3_ESM.zip › EMBOJ-2023-115792R2_SourceData_Fig2/Fig2A-B-C_microscopy/R1/WT EV R1/DAPI.jpg]

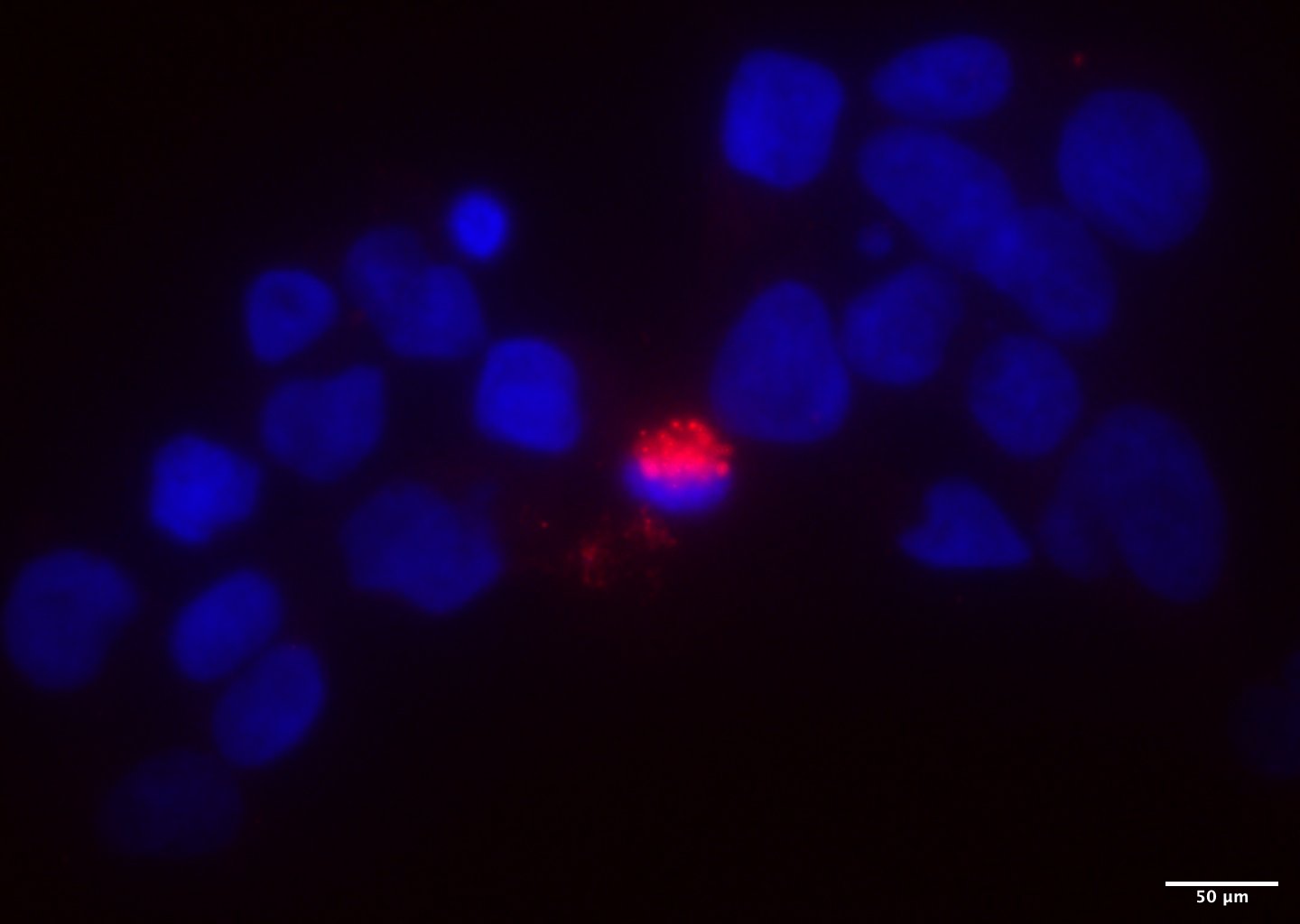

Supplement: Supplementary file 3 — Source Data Fig. 2 [file 44318_2024_35_MOESM3_ESM.zip › EMBOJ-2023-115792R2_SourceData_Fig2/Fig2A-B-C_microscopy/R1/N1 EV R1/N1 EV71 merge.jpg]
