# Supplementary figures and images for: The helicase domain of human Dicer prevents RNAi-independent activation of antiviral and inflammatory pathways (part 2 of 5)
Source: EMBO J. 2024 Jan 29;43(5):7. doi: 10.1038/s44318-024-00035-2 (PMC10907635; doi:10.1038/s44318-024-00035-2)

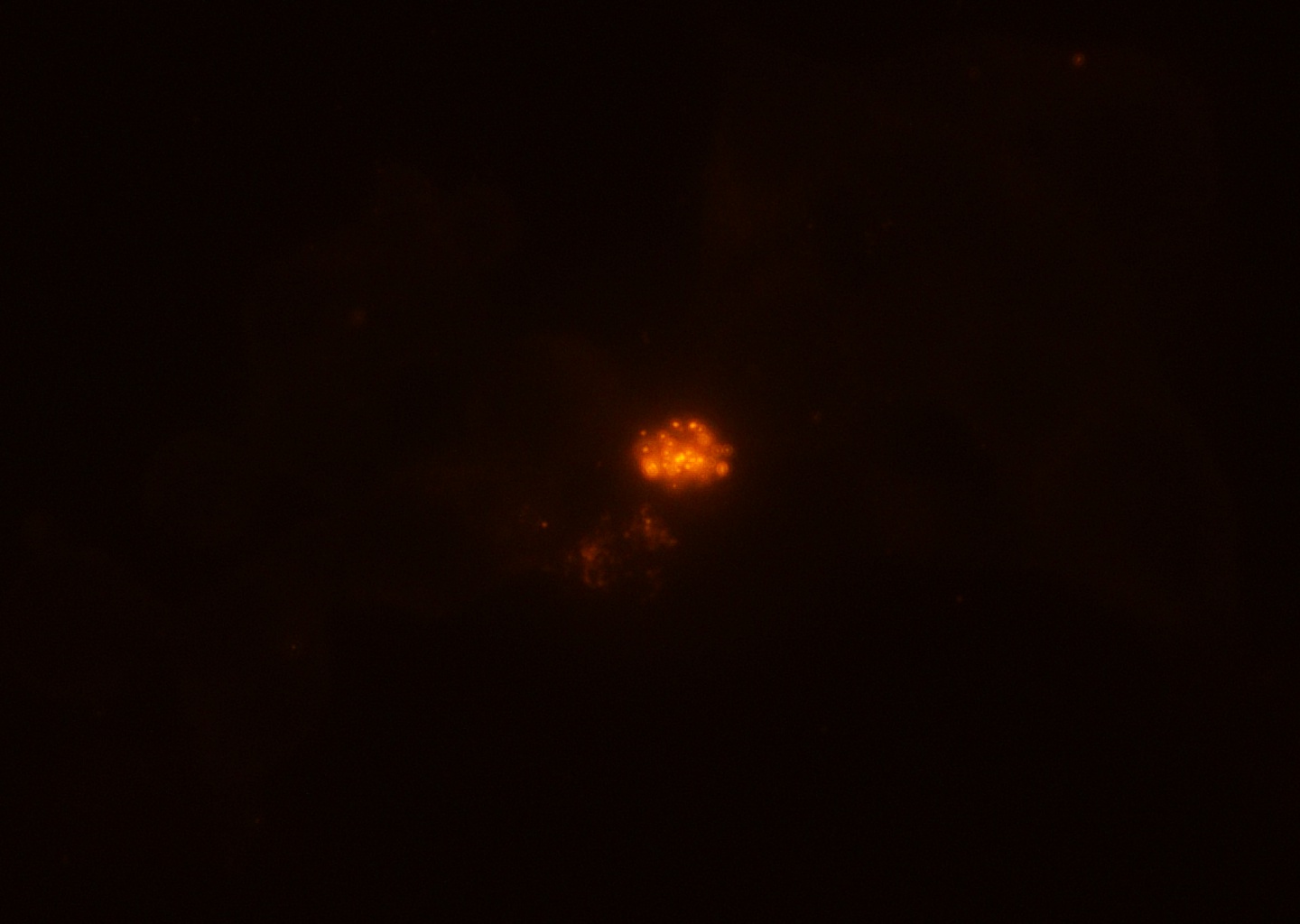

Supplement: Supplementary file 3 — Source Data Fig. 2 [file 44318_2024_35_MOESM3_ESM.zip › EMBOJ-2023-115792R2_SourceData_Fig2/Fig2A-B-C_microscopy/R1/N1 EV R1/J2.jpg]

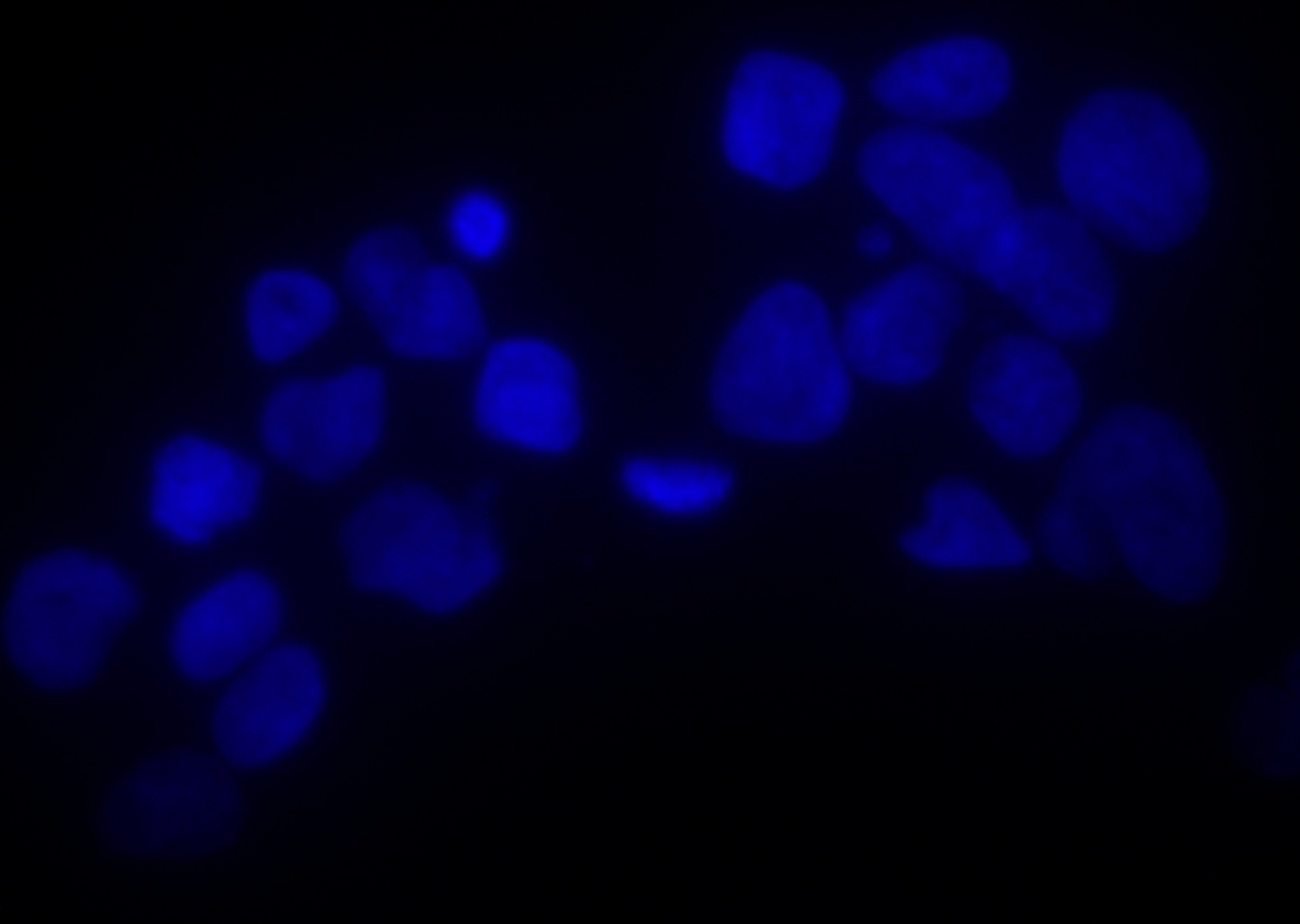

Supplement: Supplementary file 3 — Source Data Fig. 2 [file 44318_2024_35_MOESM3_ESM.zip › EMBOJ-2023-115792R2_SourceData_Fig2/Fig2A-B-C_microscopy/R1/N1 EV R1/DAPI.jpg]

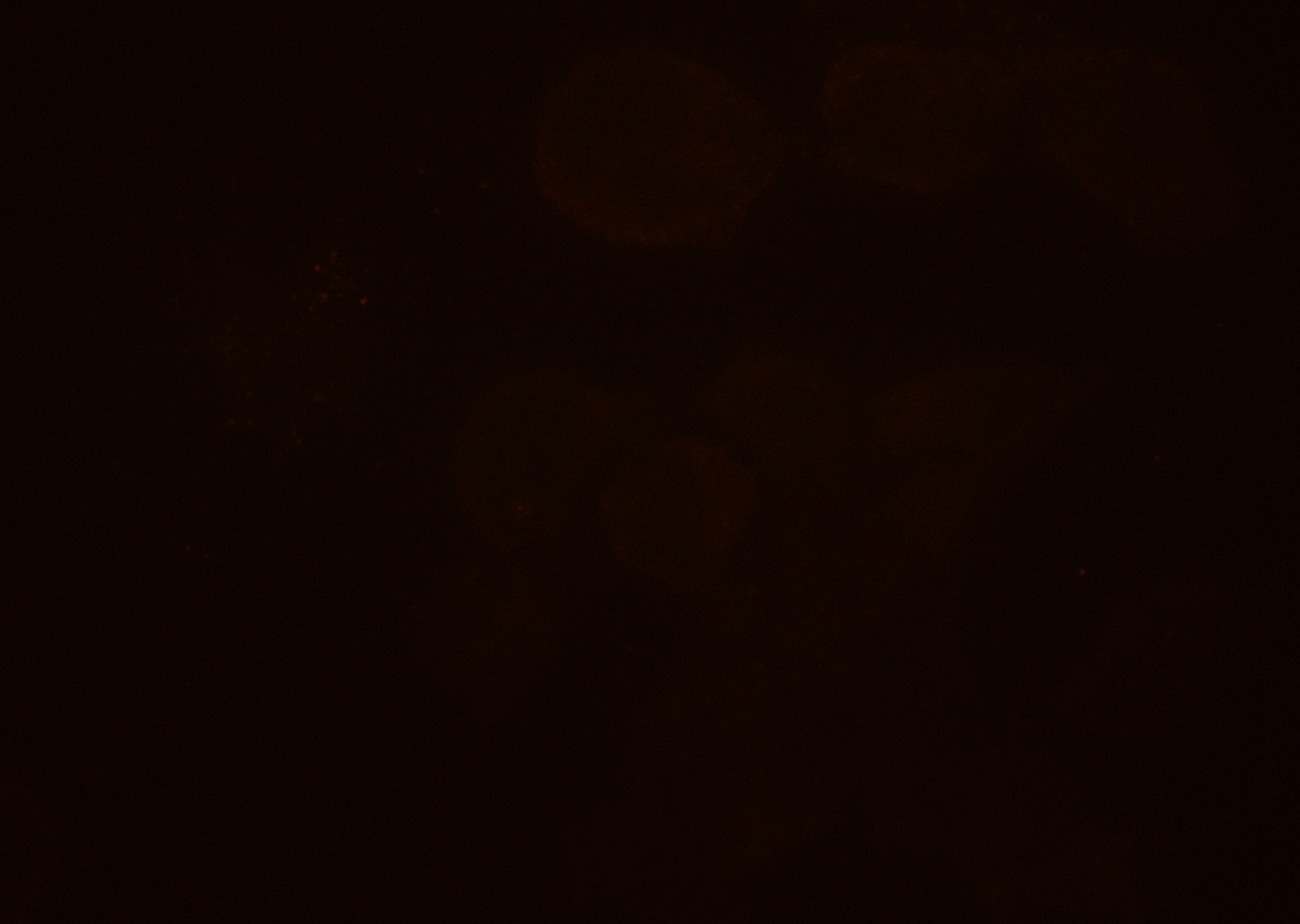

Supplement: Supplementary file 3 — Source Data Fig. 2 [file 44318_2024_35_MOESM3_ESM.zip › EMBOJ-2023-115792R2_SourceData_Fig2/Fig2A-B-C_microscopy/R1/N1 VSV R1/J2.jpg]

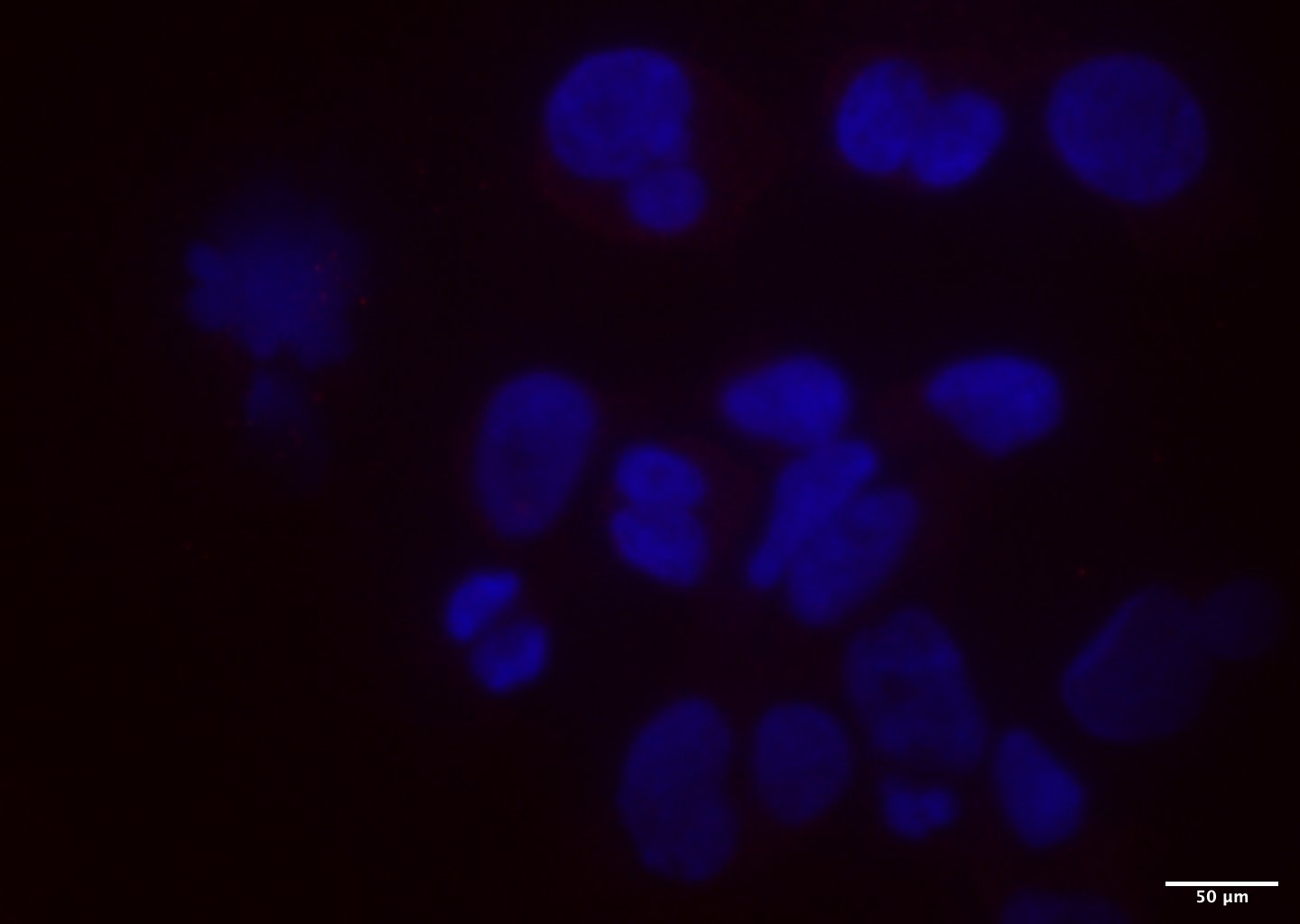

Supplement: Supplementary file 3 — Source Data Fig. 2 [file 44318_2024_35_MOESM3_ESM.zip › EMBOJ-2023-115792R2_SourceData_Fig2/Fig2A-B-C_microscopy/R1/N1 VSV R1/N1 VSV merge.jpg]

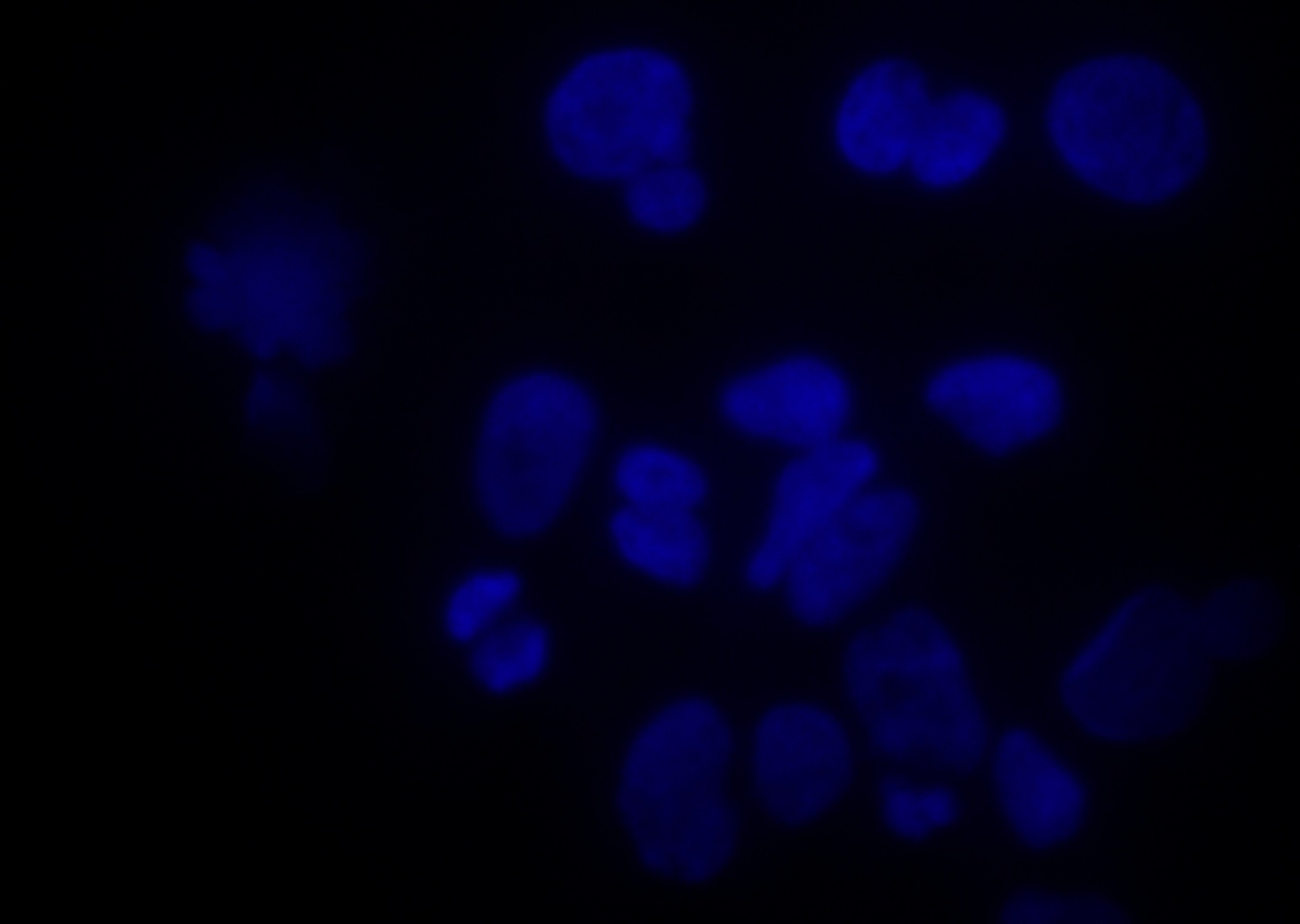

Supplement: Supplementary file 3 — Source Data Fig. 2 [file 44318_2024_35_MOESM3_ESM.zip › EMBOJ-2023-115792R2_SourceData_Fig2/Fig2A-B-C_microscopy/R1/N1 VSV R1/DAPI.jpg]

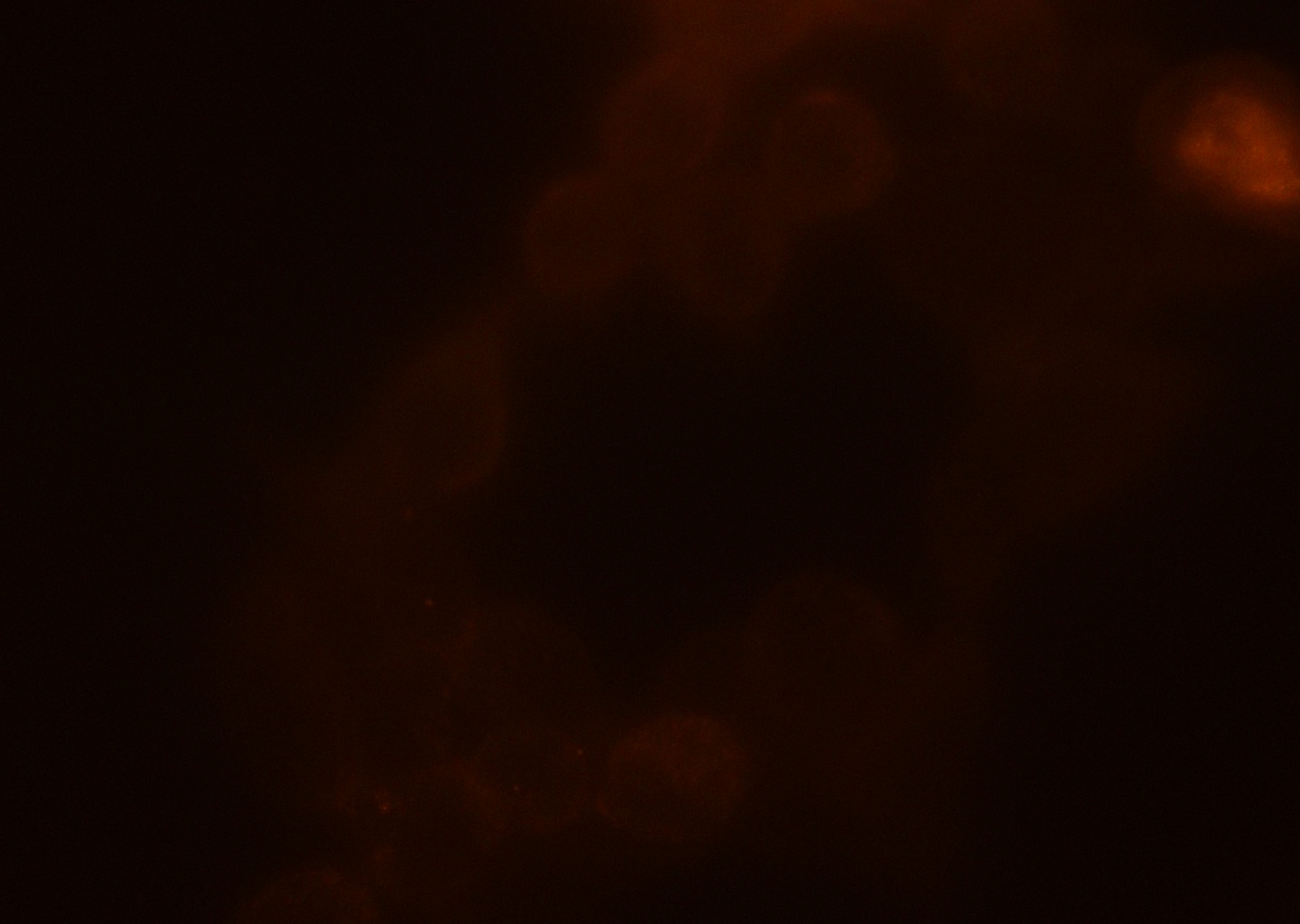

Supplement: Supplementary file 3 — Source Data Fig. 2 [file 44318_2024_35_MOESM3_ESM.zip › EMBOJ-2023-115792R2_SourceData_Fig2/Fig2A-B-C_microscopy/R1/WT VSV R1/J2.jpg]

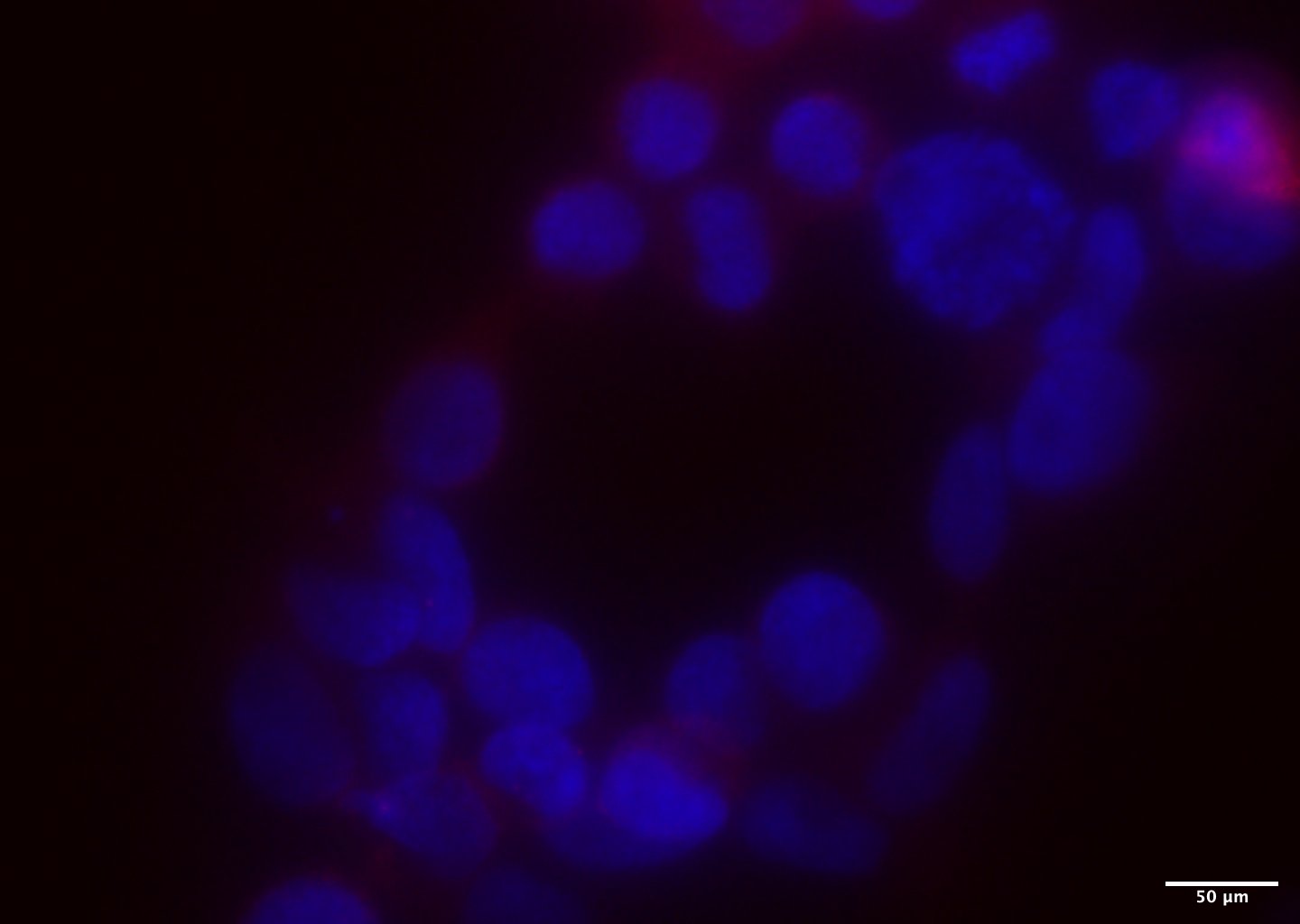

Supplement: Supplementary file 3 — Source Data Fig. 2 [file 44318_2024_35_MOESM3_ESM.zip › EMBOJ-2023-115792R2_SourceData_Fig2/Fig2A-B-C_microscopy/R1/WT VSV R1/WT VSV merge.jpg]

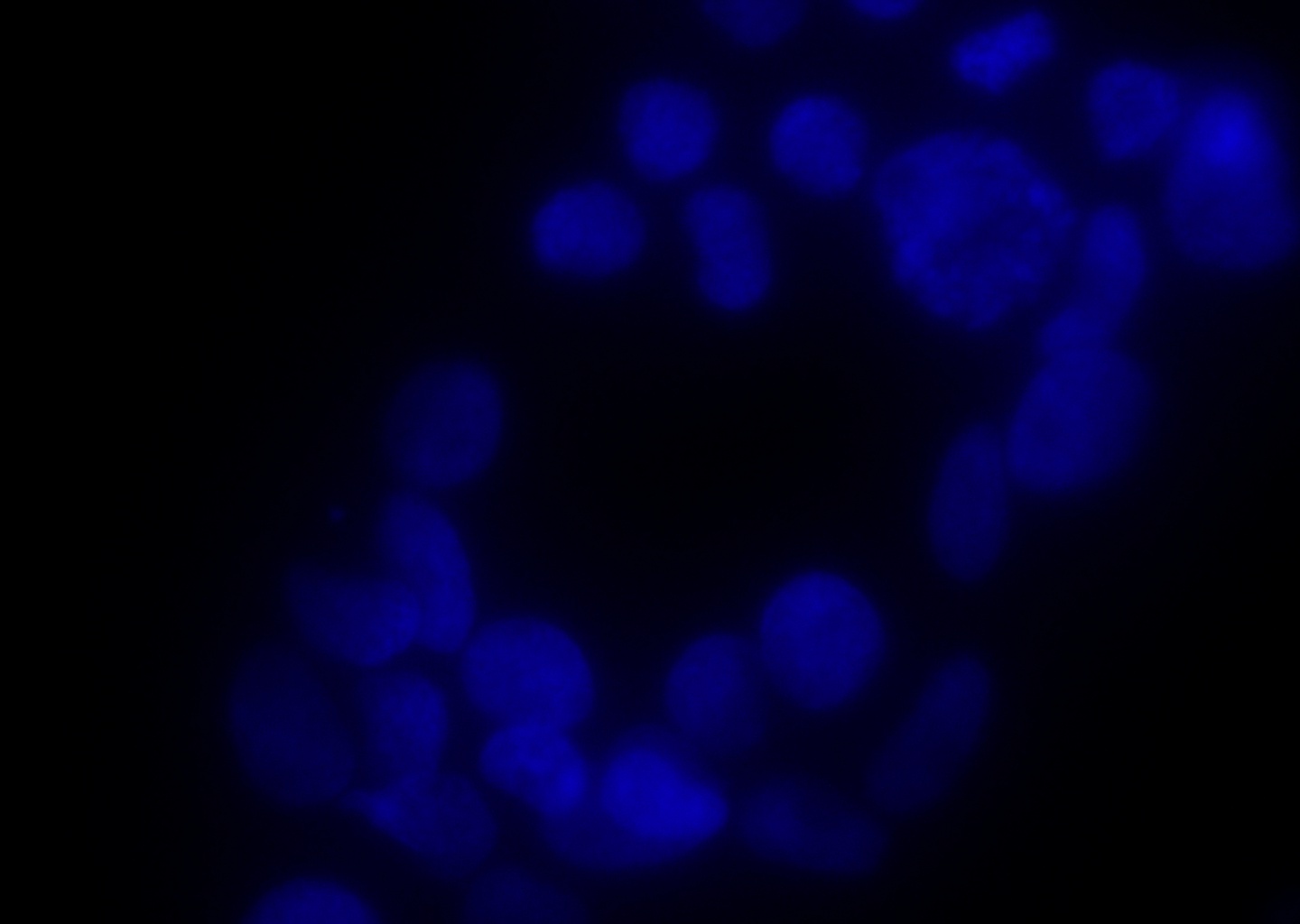

Supplement: Supplementary file 3 — Source Data Fig. 2 [file 44318_2024_35_MOESM3_ESM.zip › EMBOJ-2023-115792R2_SourceData_Fig2/Fig2A-B-C_microscopy/R1/WT VSV R1/DAPI.jpg]

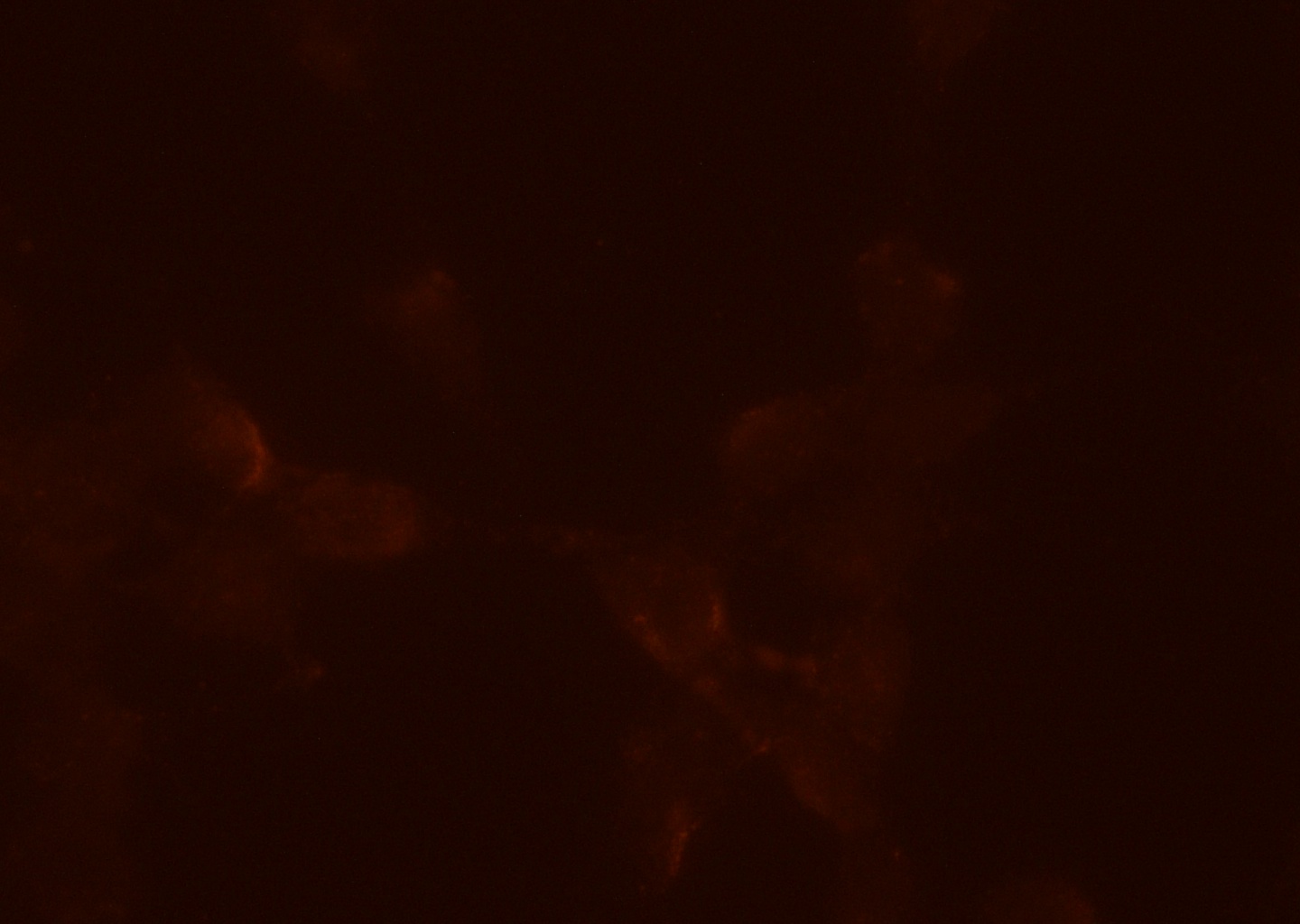

Supplement: Supplementary file 3 — Source Data Fig. 2 [file 44318_2024_35_MOESM3_ESM.zip › EMBOJ-2023-115792R2_SourceData_Fig2/Fig2A-B-C_microscopy/R3/N1 EV R3/J2.jpg]

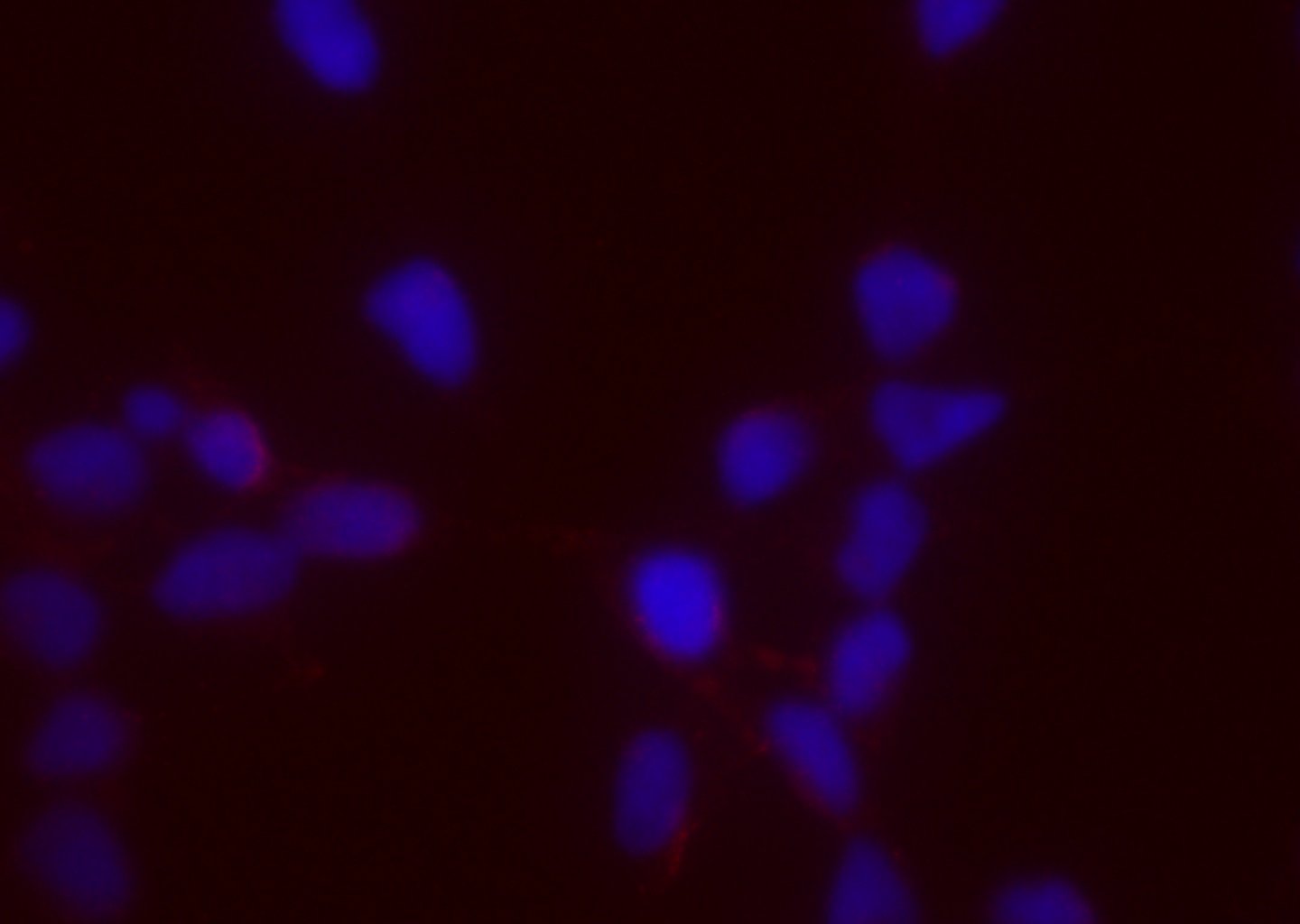

Supplement: Supplementary file 3 — Source Data Fig. 2 [file 44318_2024_35_MOESM3_ESM.zip › EMBOJ-2023-115792R2_SourceData_Fig2/Fig2A-B-C_microscopy/R3/N1 EV R3/N1 EV merge.jpg]

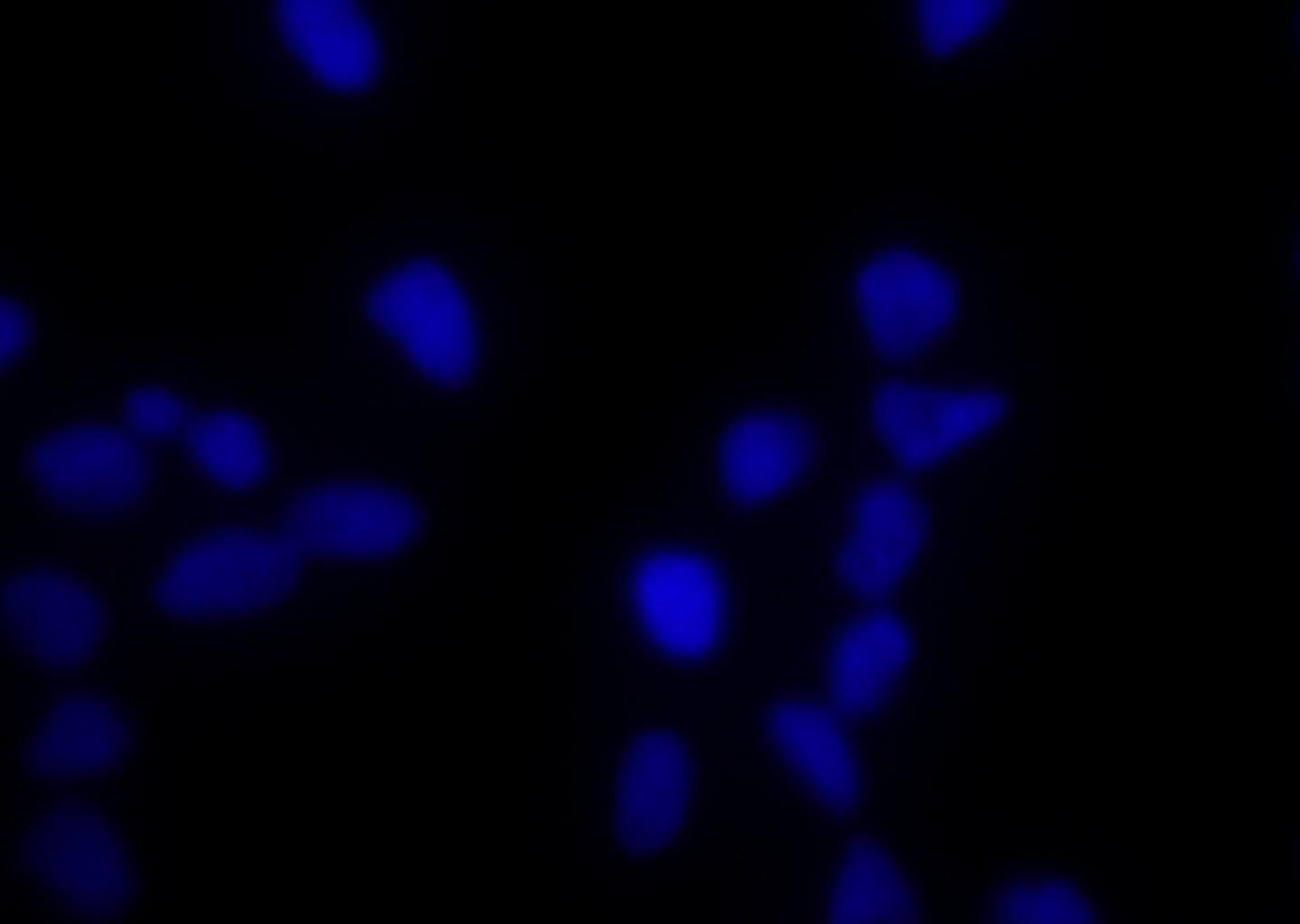

Supplement: Supplementary file 3 — Source Data Fig. 2 [file 44318_2024_35_MOESM3_ESM.zip › EMBOJ-2023-115792R2_SourceData_Fig2/Fig2A-B-C_microscopy/R3/N1 EV R3/DAPI.jpg]

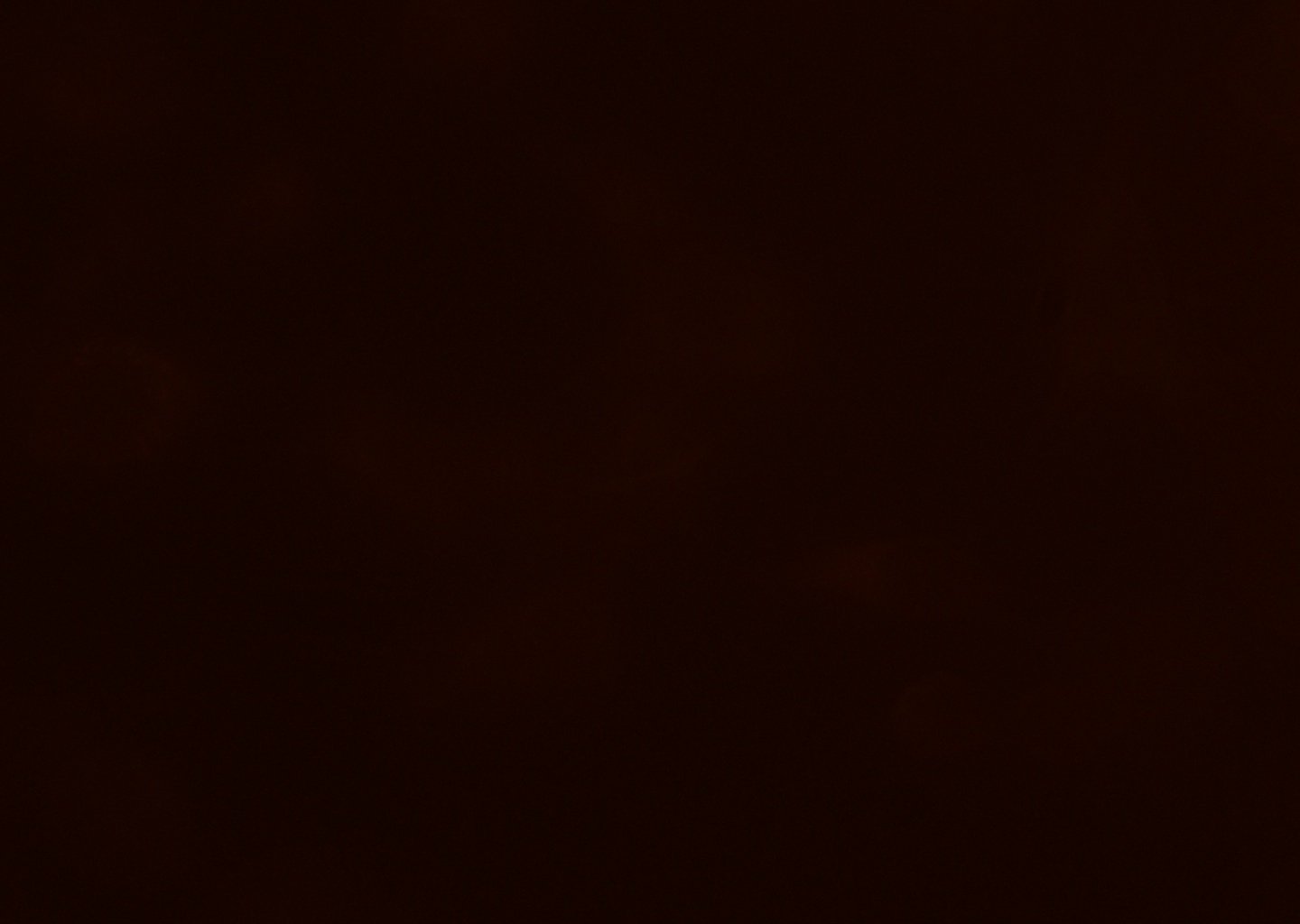

Supplement: Supplementary file 3 — Source Data Fig. 2 [file 44318_2024_35_MOESM3_ESM.zip › EMBOJ-2023-115792R2_SourceData_Fig2/Fig2A-B-C_microscopy/R3/N1 VSV R3/J2.jpg]

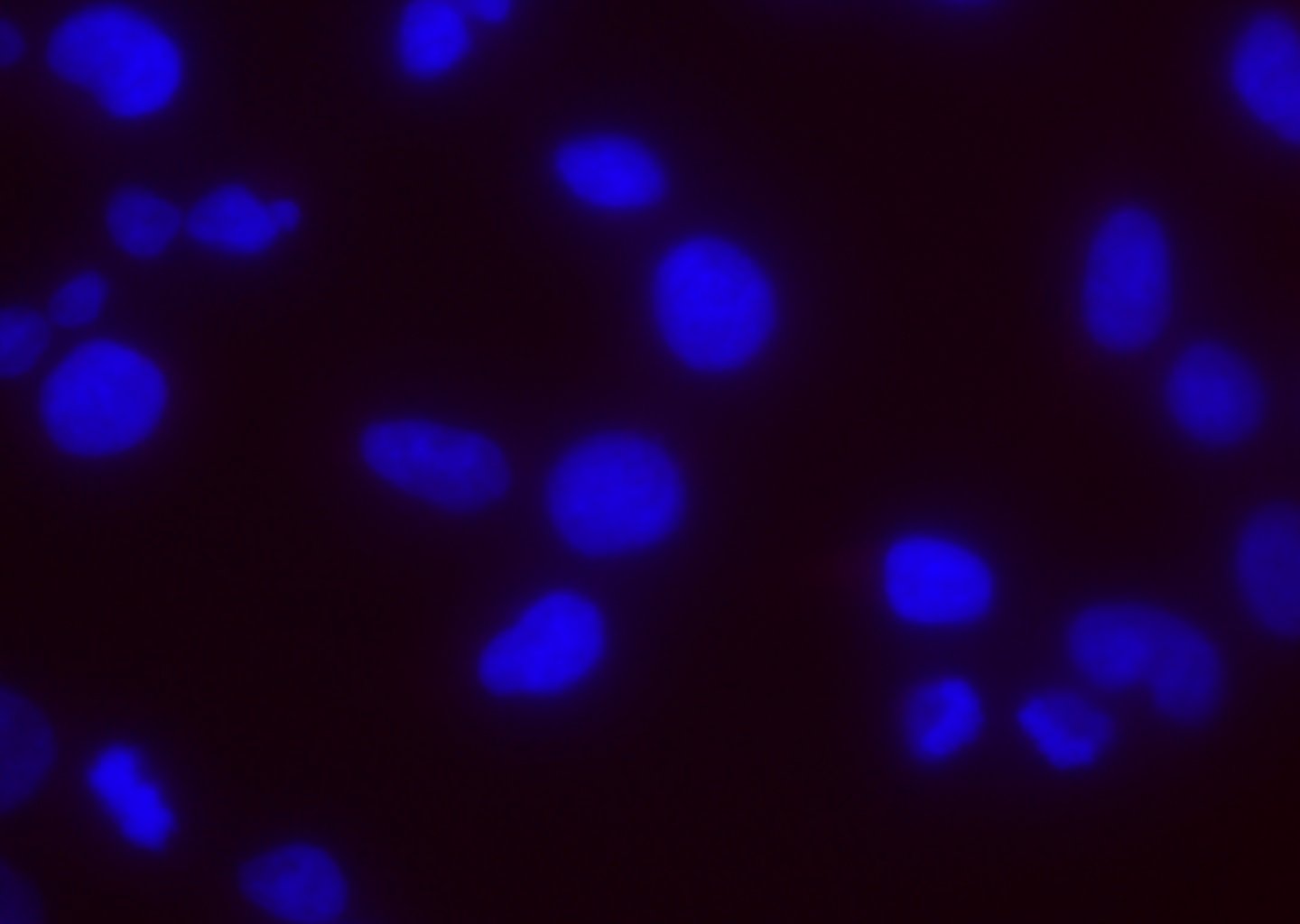

Supplement: Supplementary file 3 — Source Data Fig. 2 [file 44318_2024_35_MOESM3_ESM.zip › EMBOJ-2023-115792R2_SourceData_Fig2/Fig2A-B-C_microscopy/R3/N1 VSV R3/N1 VSV merge.jpg]

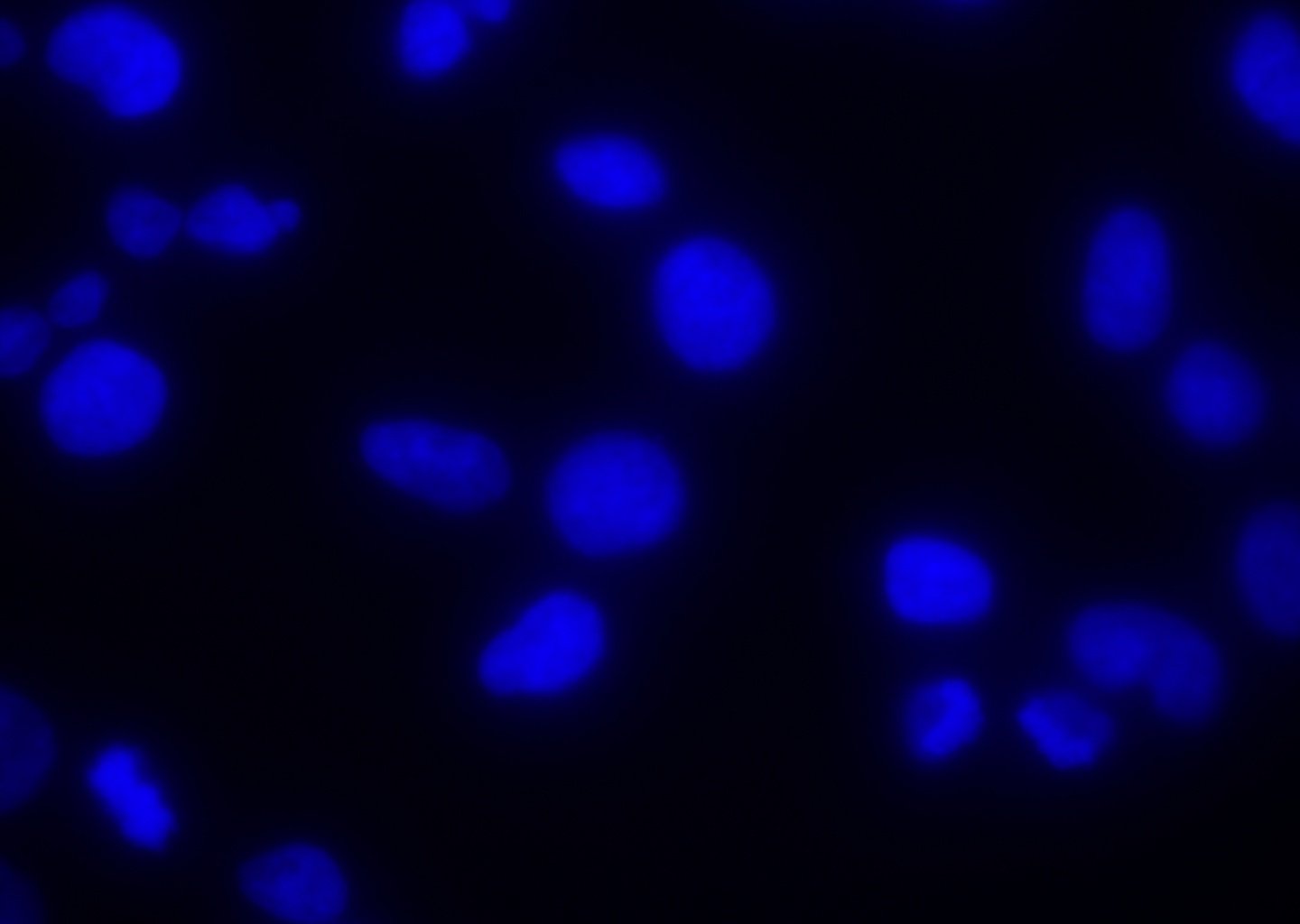

Supplement: Supplementary file 3 — Source Data Fig. 2 [file 44318_2024_35_MOESM3_ESM.zip › EMBOJ-2023-115792R2_SourceData_Fig2/Fig2A-B-C_microscopy/R3/N1 VSV R3/DAPI.jpg]

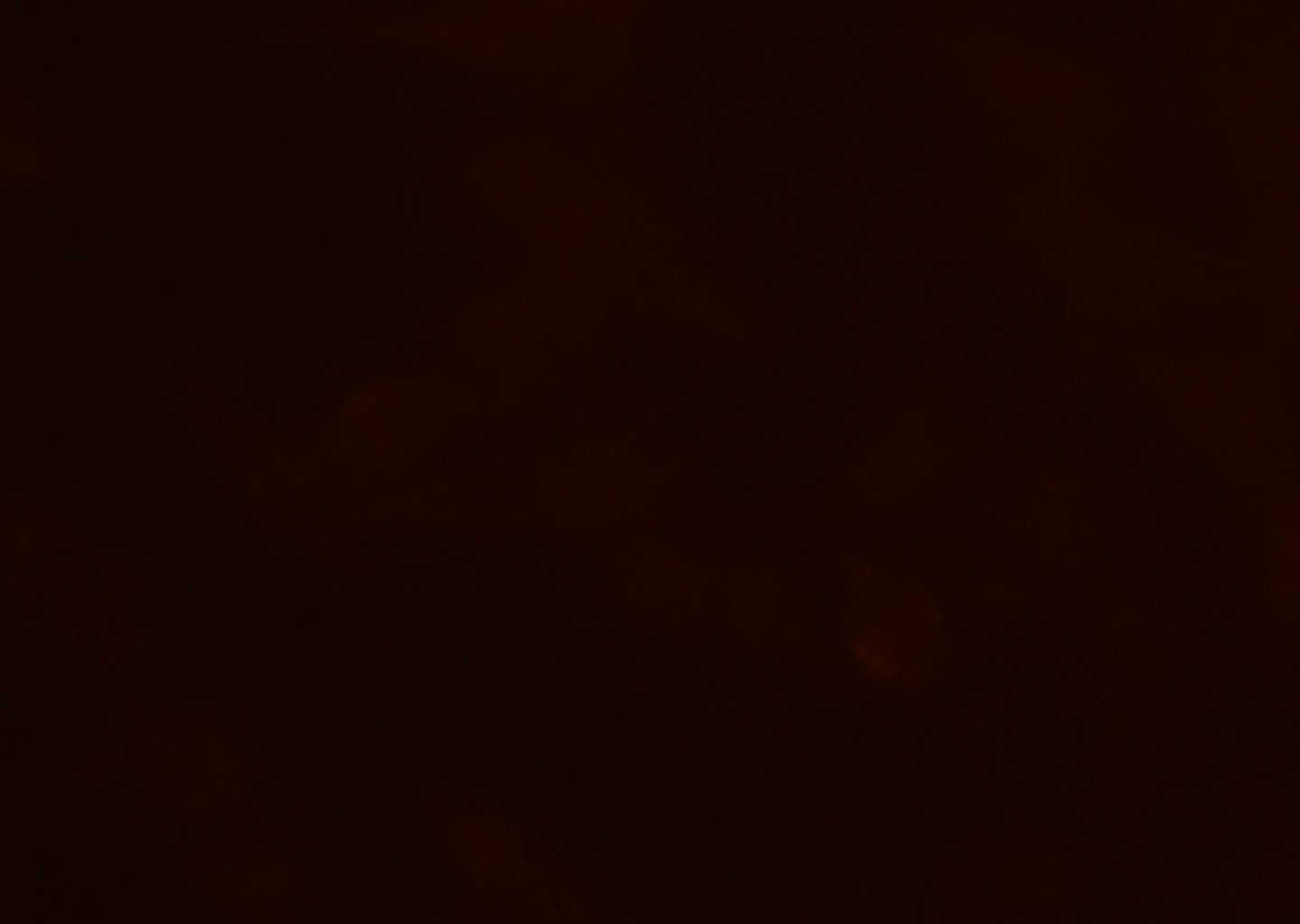

Supplement: Supplementary file 3 — Source Data Fig. 2 [file 44318_2024_35_MOESM3_ESM.zip › EMBOJ-2023-115792R2_SourceData_Fig2/Fig2A-B-C_microscopy/R3/WT VSV R3/J2.jpg]

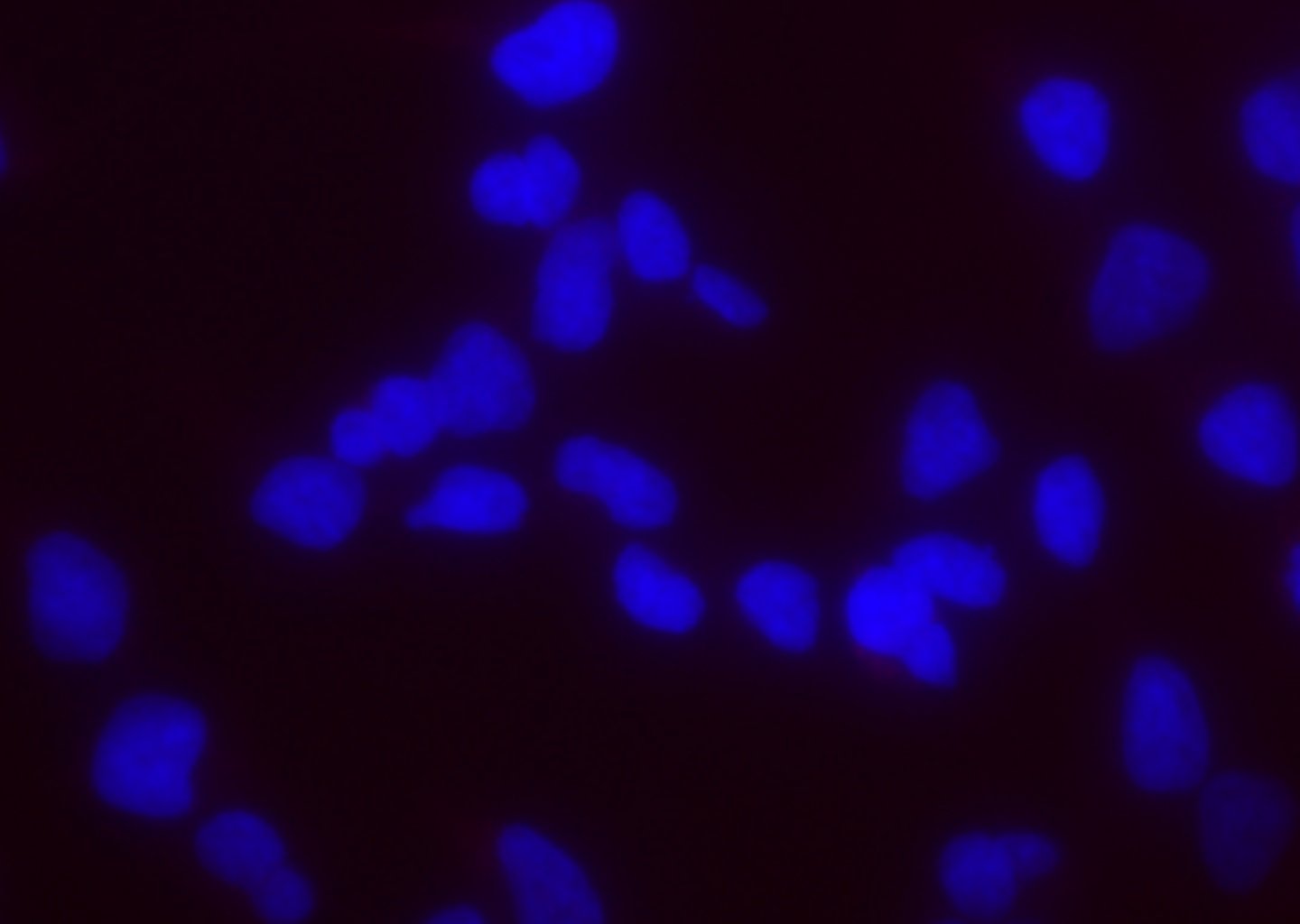

Supplement: Supplementary file 3 — Source Data Fig. 2 [file 44318_2024_35_MOESM3_ESM.zip › EMBOJ-2023-115792R2_SourceData_Fig2/Fig2A-B-C_microscopy/R3/WT VSV R3/WT VSV merge.jpg]

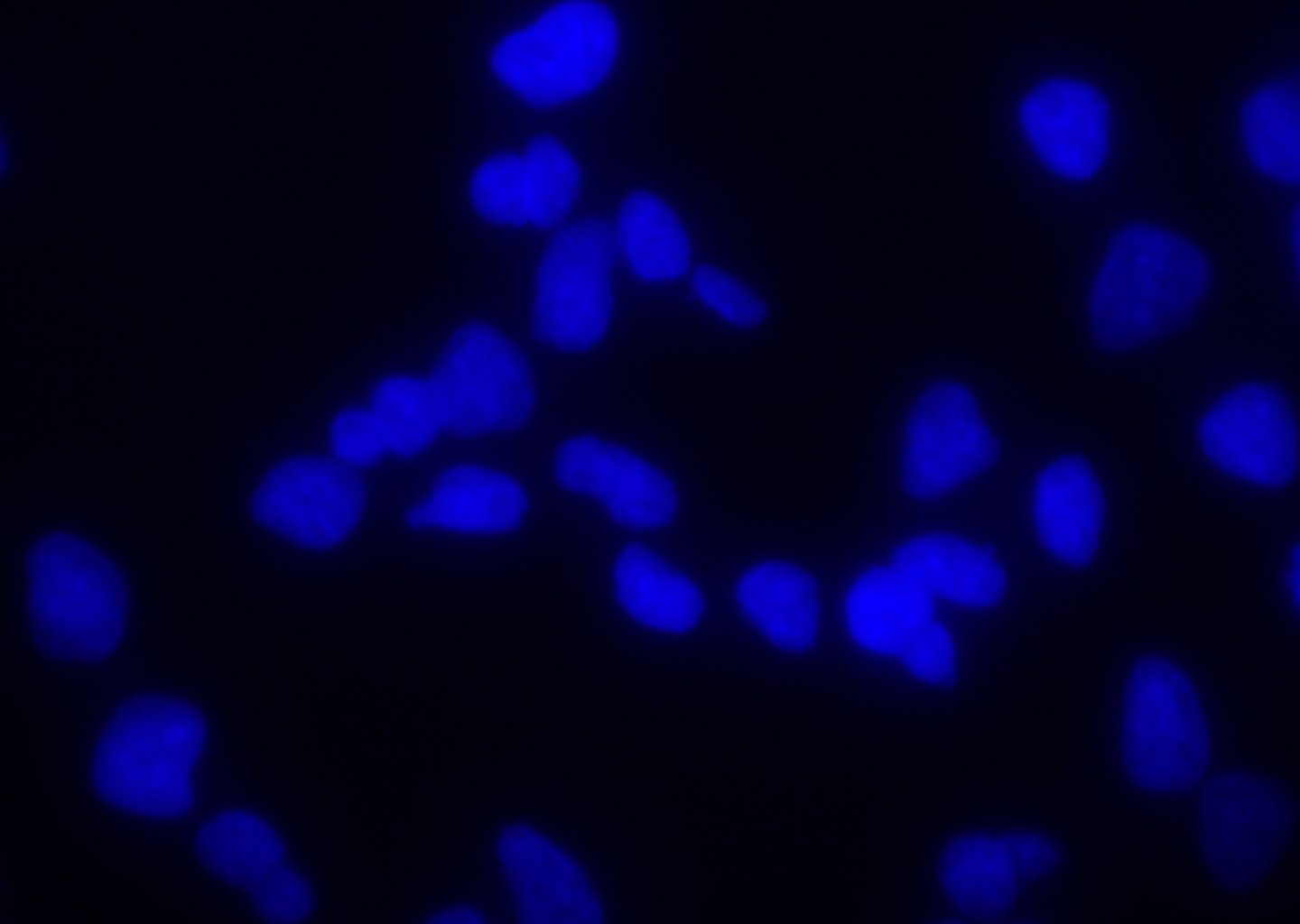

Supplement: Supplementary file 3 — Source Data Fig. 2 [file 44318_2024_35_MOESM3_ESM.zip › EMBOJ-2023-115792R2_SourceData_Fig2/Fig2A-B-C_microscopy/R3/WT VSV R3/DAPI.jpg]

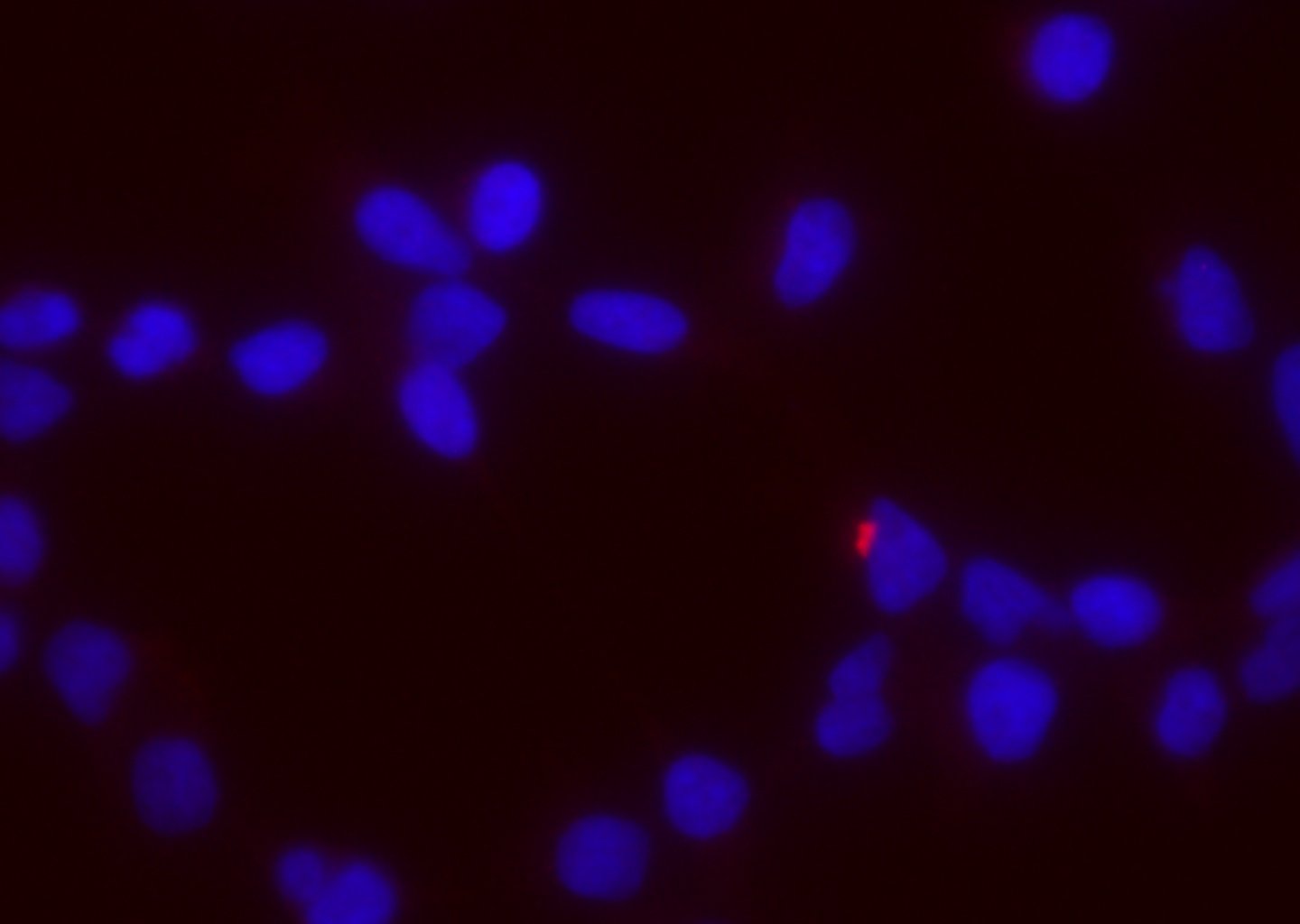

Supplement: Supplementary file 3 — Source Data Fig. 2 [file 44318_2024_35_MOESM3_ESM.zip › EMBOJ-2023-115792R2_SourceData_Fig2/Fig2A-B-C_microscopy/R3/N1 SFV R3/N1 SFV merge.jpg]

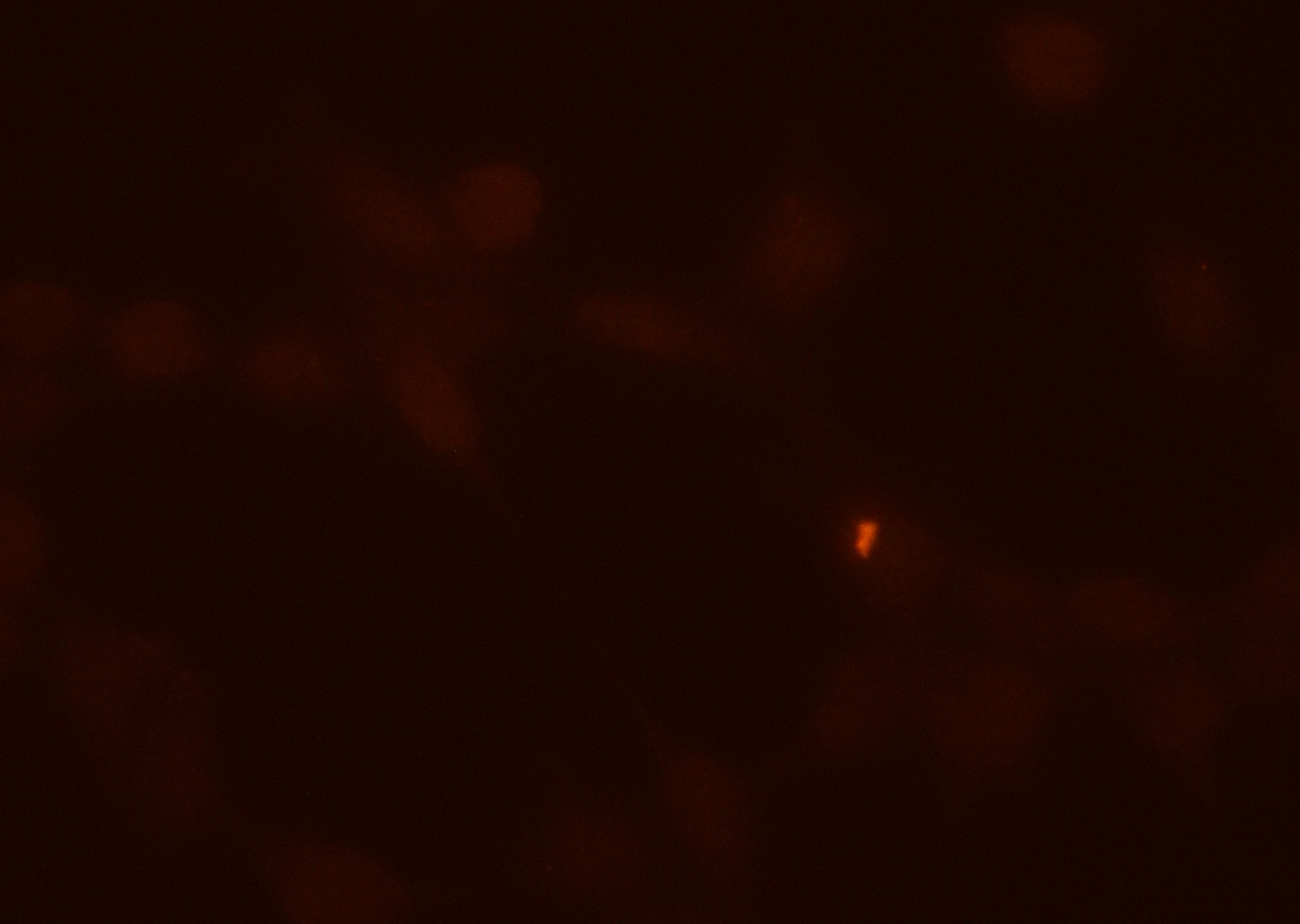

Supplement: Supplementary file 3 — Source Data Fig. 2 [file 44318_2024_35_MOESM3_ESM.zip › EMBOJ-2023-115792R2_SourceData_Fig2/Fig2A-B-C_microscopy/R3/N1 SFV R3/J2.jpg]

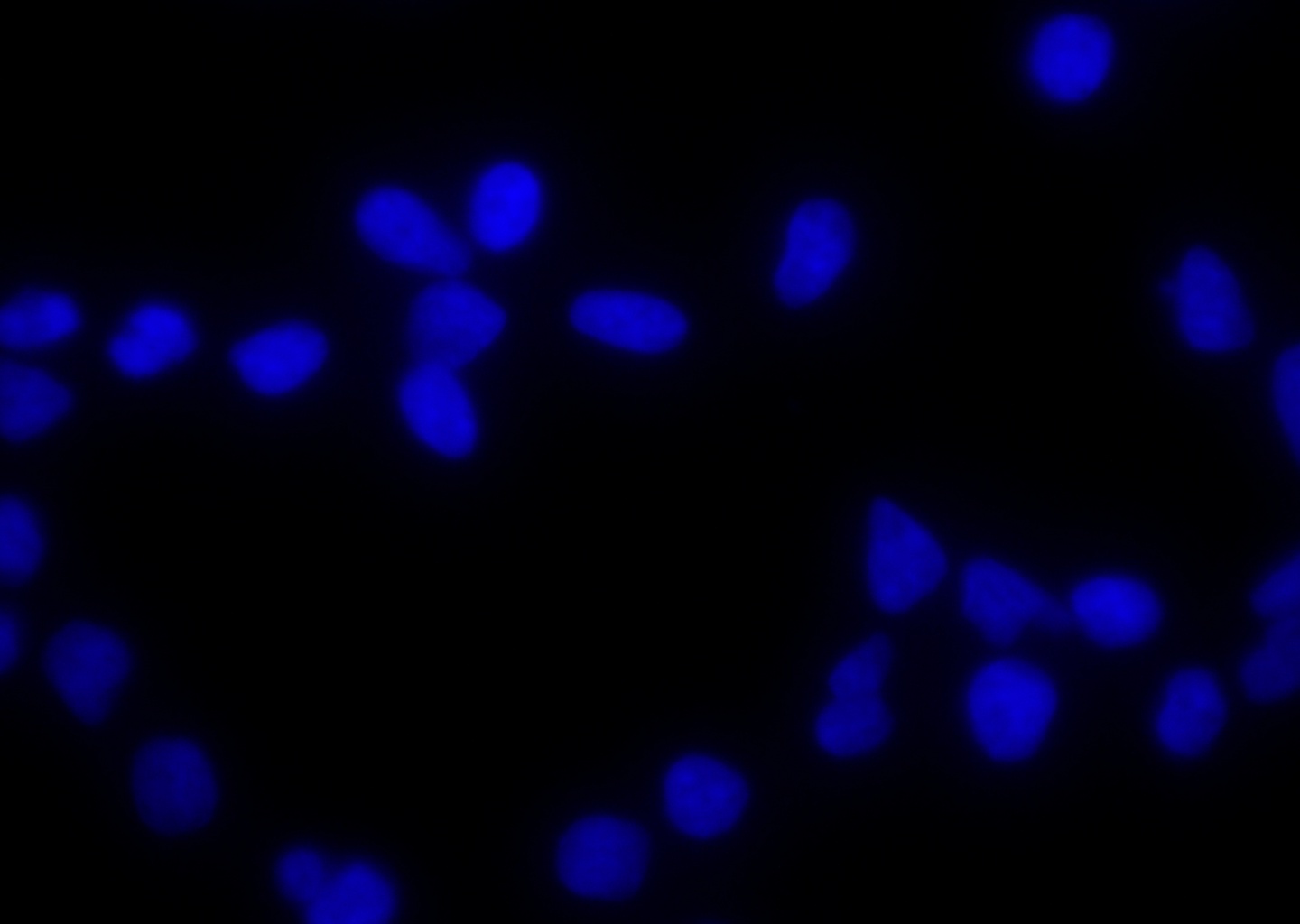

Supplement: Supplementary file 3 — Source Data Fig. 2 [file 44318_2024_35_MOESM3_ESM.zip › EMBOJ-2023-115792R2_SourceData_Fig2/Fig2A-B-C_microscopy/R3/N1 SFV R3/DAPI.jpg]

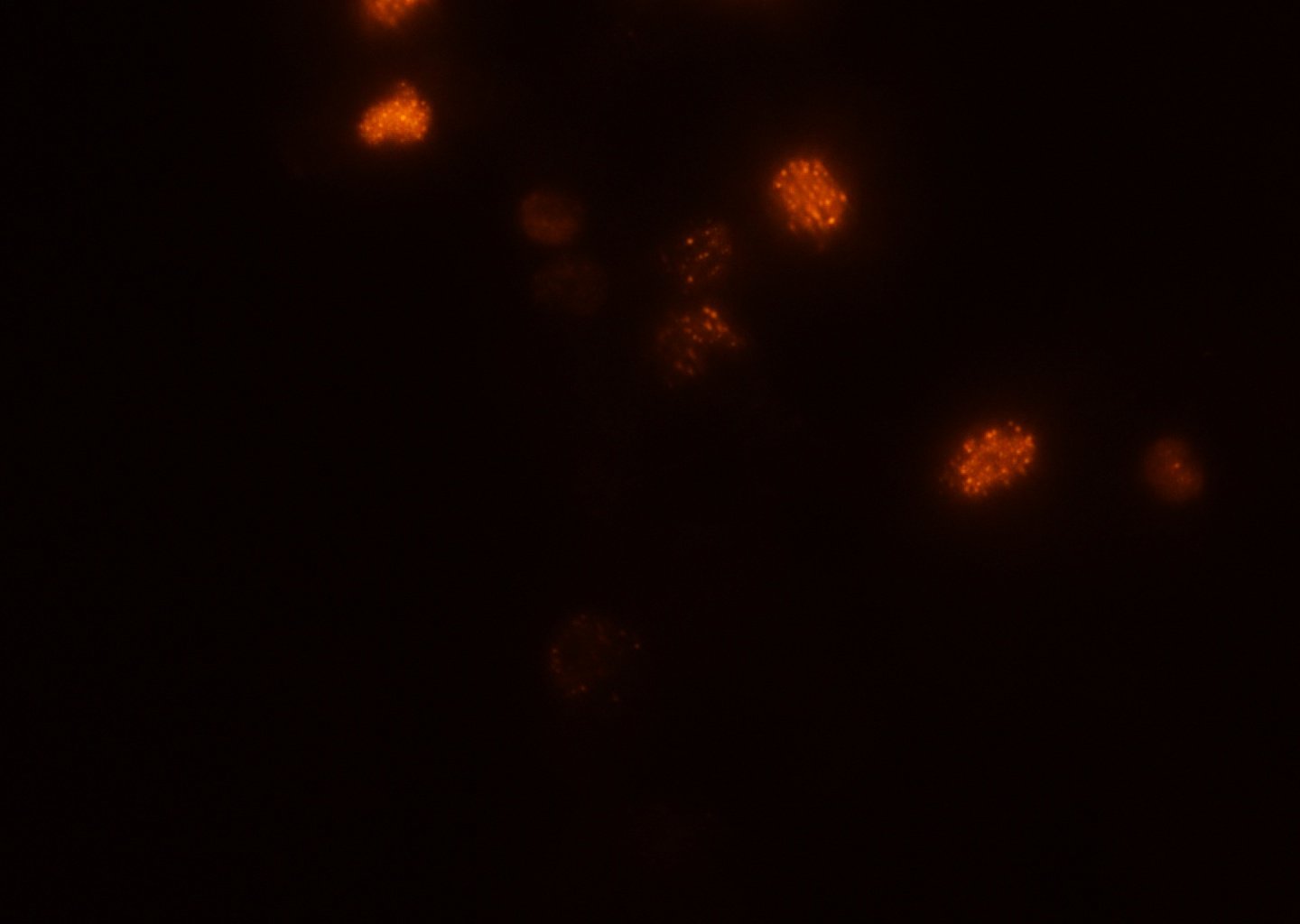

Supplement: Supplementary file 3 — Source Data Fig. 2 [file 44318_2024_35_MOESM3_ESM.zip › EMBOJ-2023-115792R2_SourceData_Fig2/Fig2A-B-C_microscopy/R3/WT EV R3/J2.jpg]

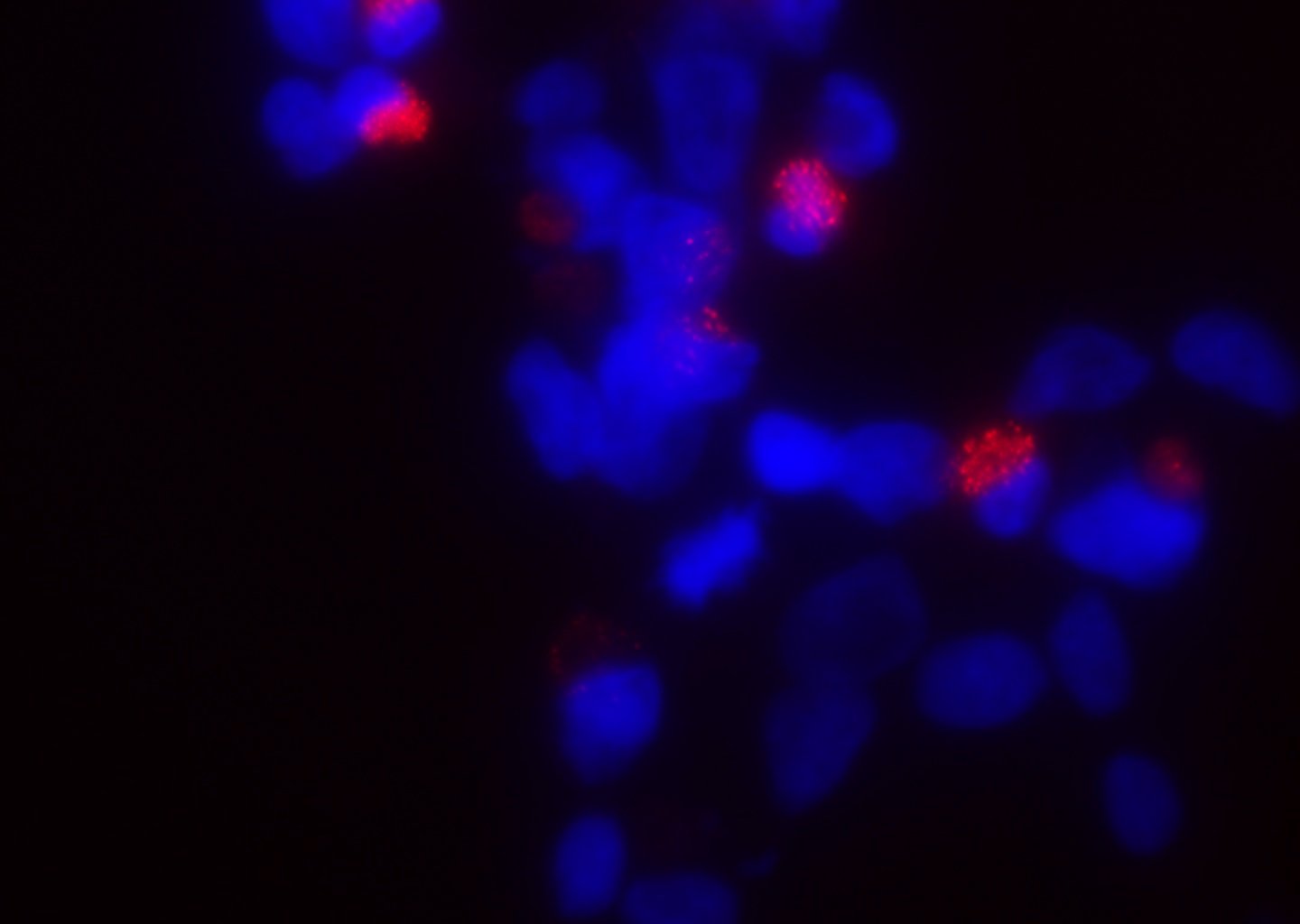

Supplement: Supplementary file 3 — Source Data Fig. 2 [file 44318_2024_35_MOESM3_ESM.zip › EMBOJ-2023-115792R2_SourceData_Fig2/Fig2A-B-C_microscopy/R3/WT EV R3/WT EV merge.jpg]

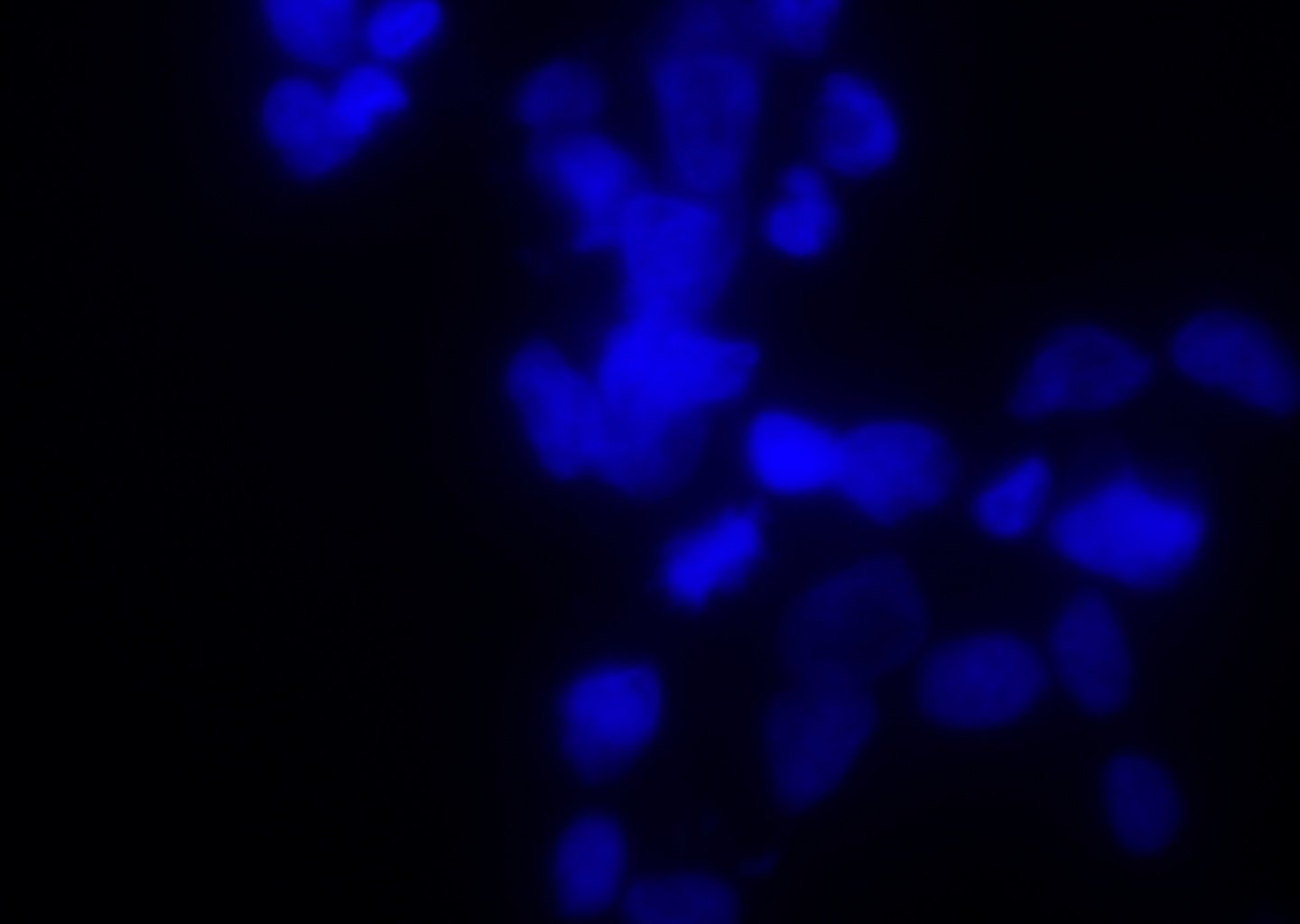

Supplement: Supplementary file 3 — Source Data Fig. 2 [file 44318_2024_35_MOESM3_ESM.zip › EMBOJ-2023-115792R2_SourceData_Fig2/Fig2A-B-C_microscopy/R3/WT EV R3/DAPI.jpg]

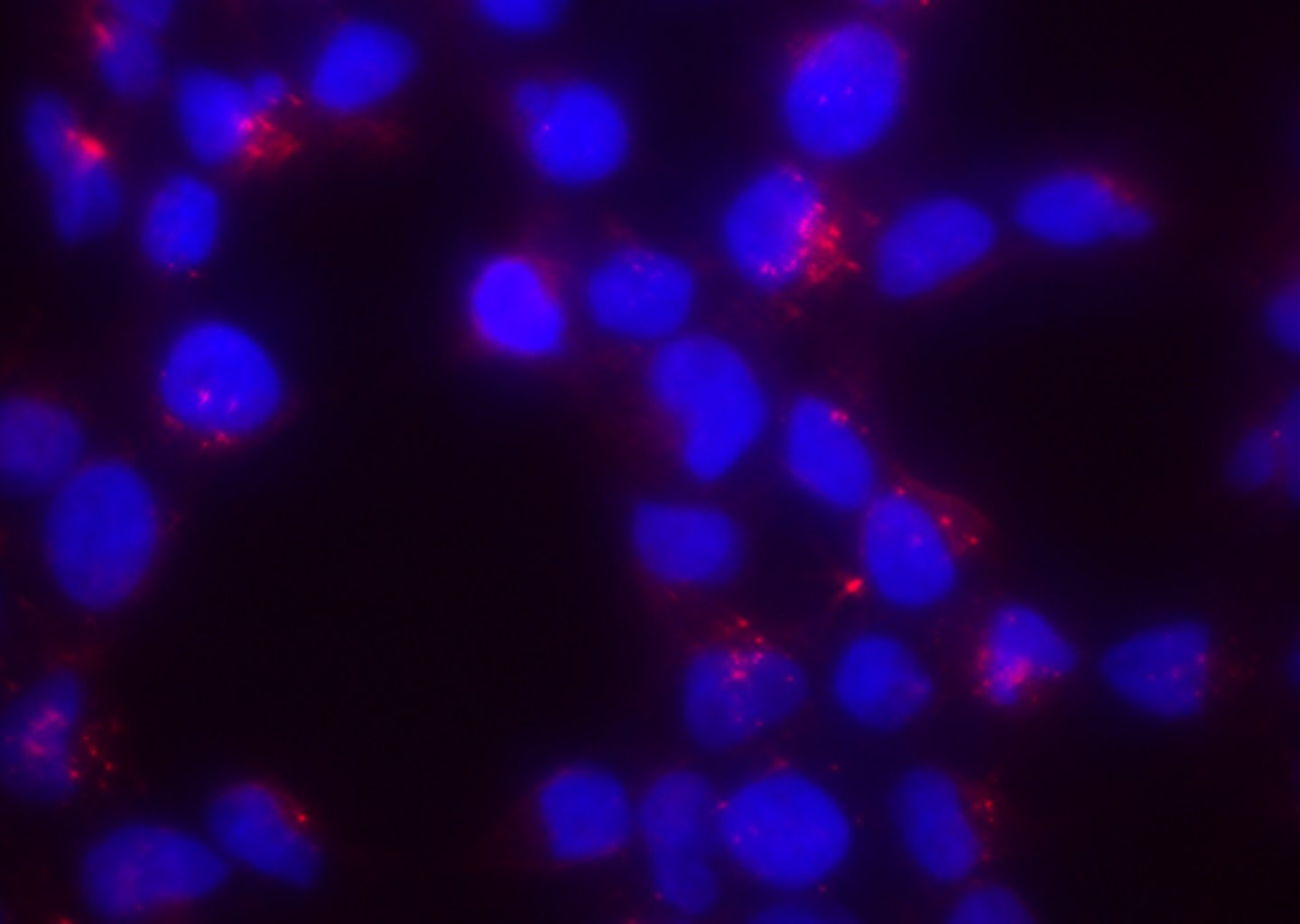

Supplement: Supplementary file 3 — Source Data Fig. 2 [file 44318_2024_35_MOESM3_ESM.zip › EMBOJ-2023-115792R2_SourceData_Fig2/Fig2A-B-C_microscopy/R3/WT SFV R3/WT SFV merge.jpg]

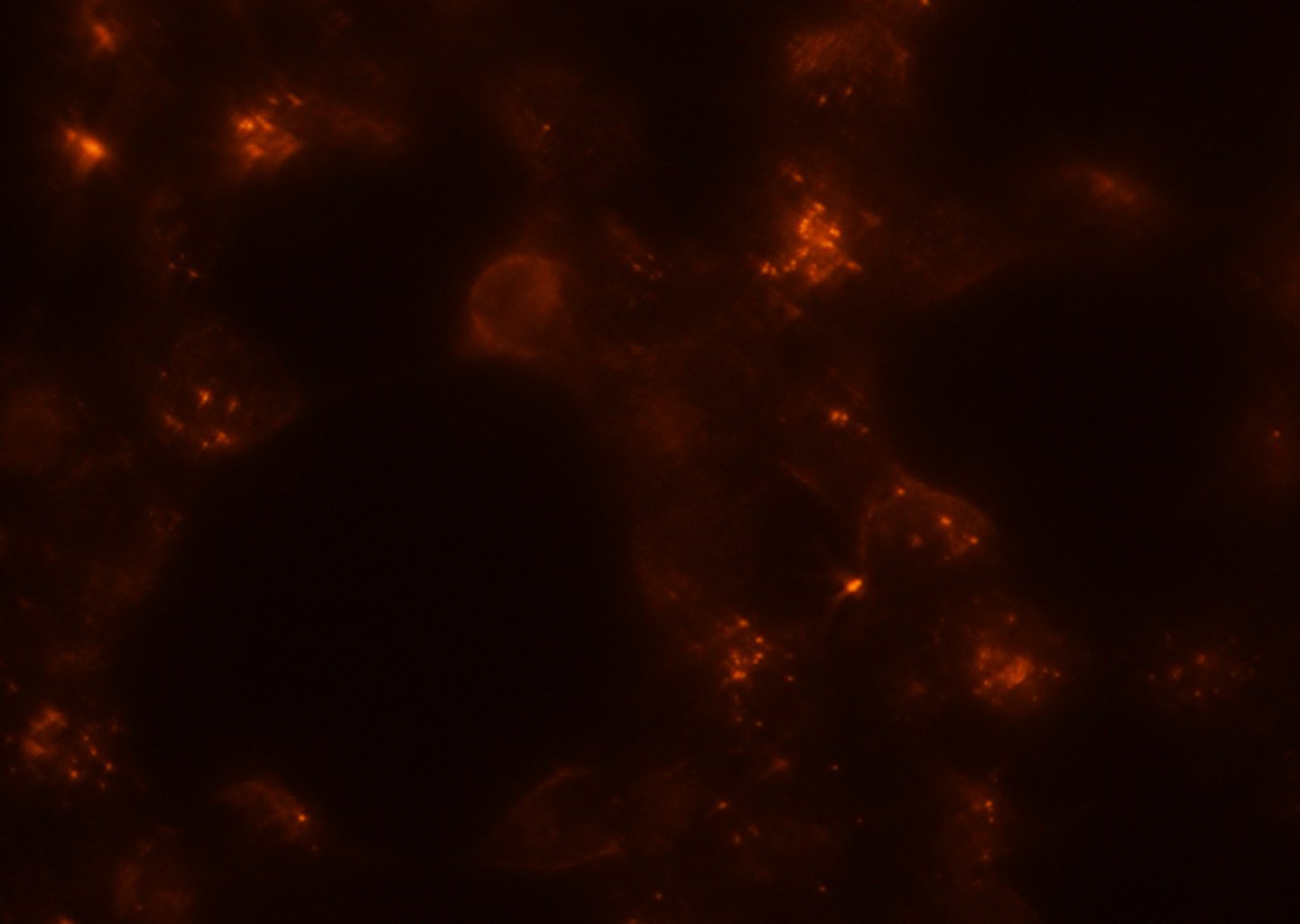

Supplement: Supplementary file 3 — Source Data Fig. 2 [file 44318_2024_35_MOESM3_ESM.zip › EMBOJ-2023-115792R2_SourceData_Fig2/Fig2A-B-C_microscopy/R3/WT SFV R3/J2.jpg]

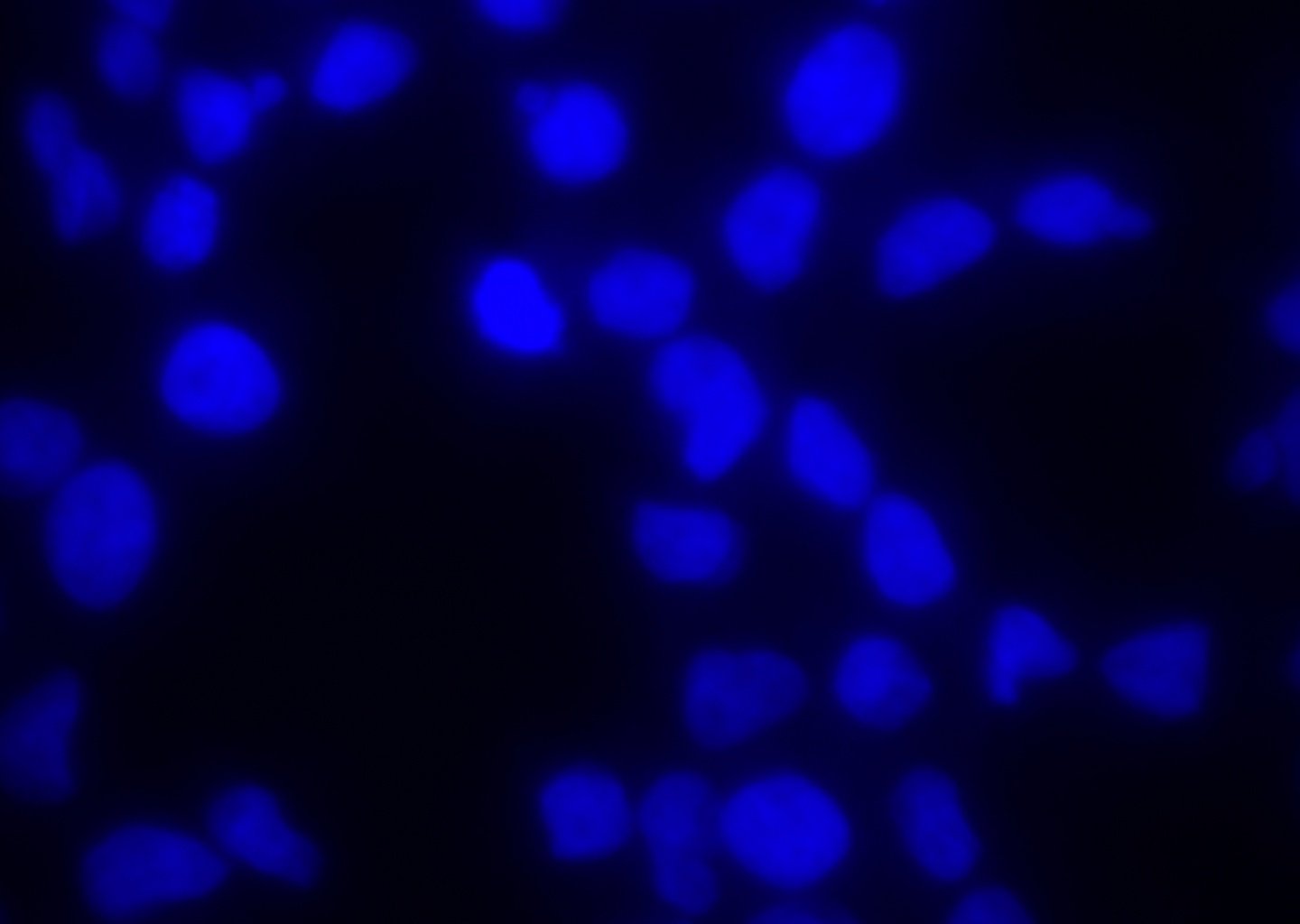

Supplement: Supplementary file 3 — Source Data Fig. 2 [file 44318_2024_35_MOESM3_ESM.zip › EMBOJ-2023-115792R2_SourceData_Fig2/Fig2A-B-C_microscopy/R3/WT SFV R3/DAPI.jpg]

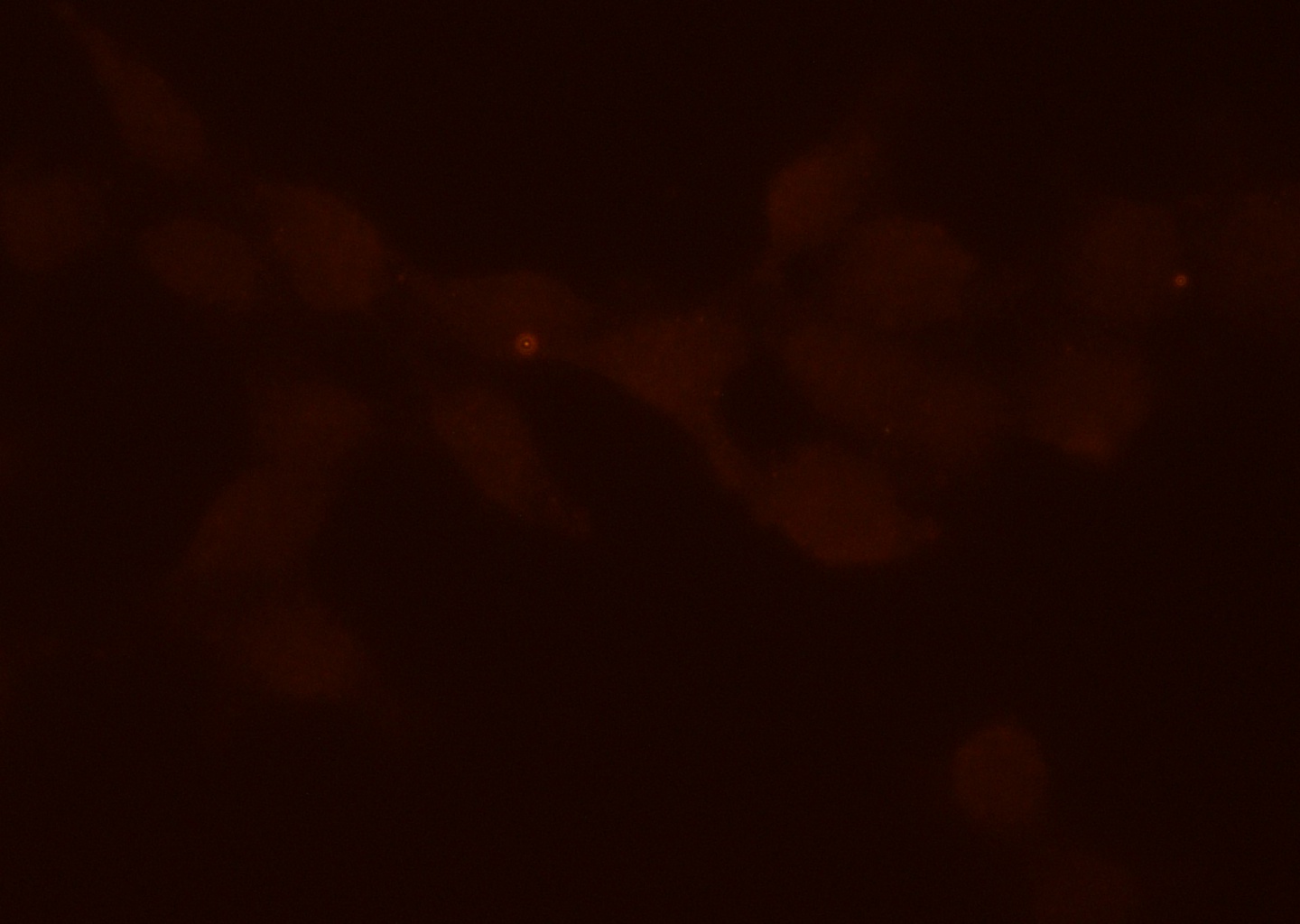

Supplement: Supplementary file 3 — Source Data Fig. 2 [file 44318_2024_35_MOESM3_ESM.zip › EMBOJ-2023-115792R2_SourceData_Fig2/Fig2A-B-C_microscopy/R2/N1 EV R2/J2.jpg]

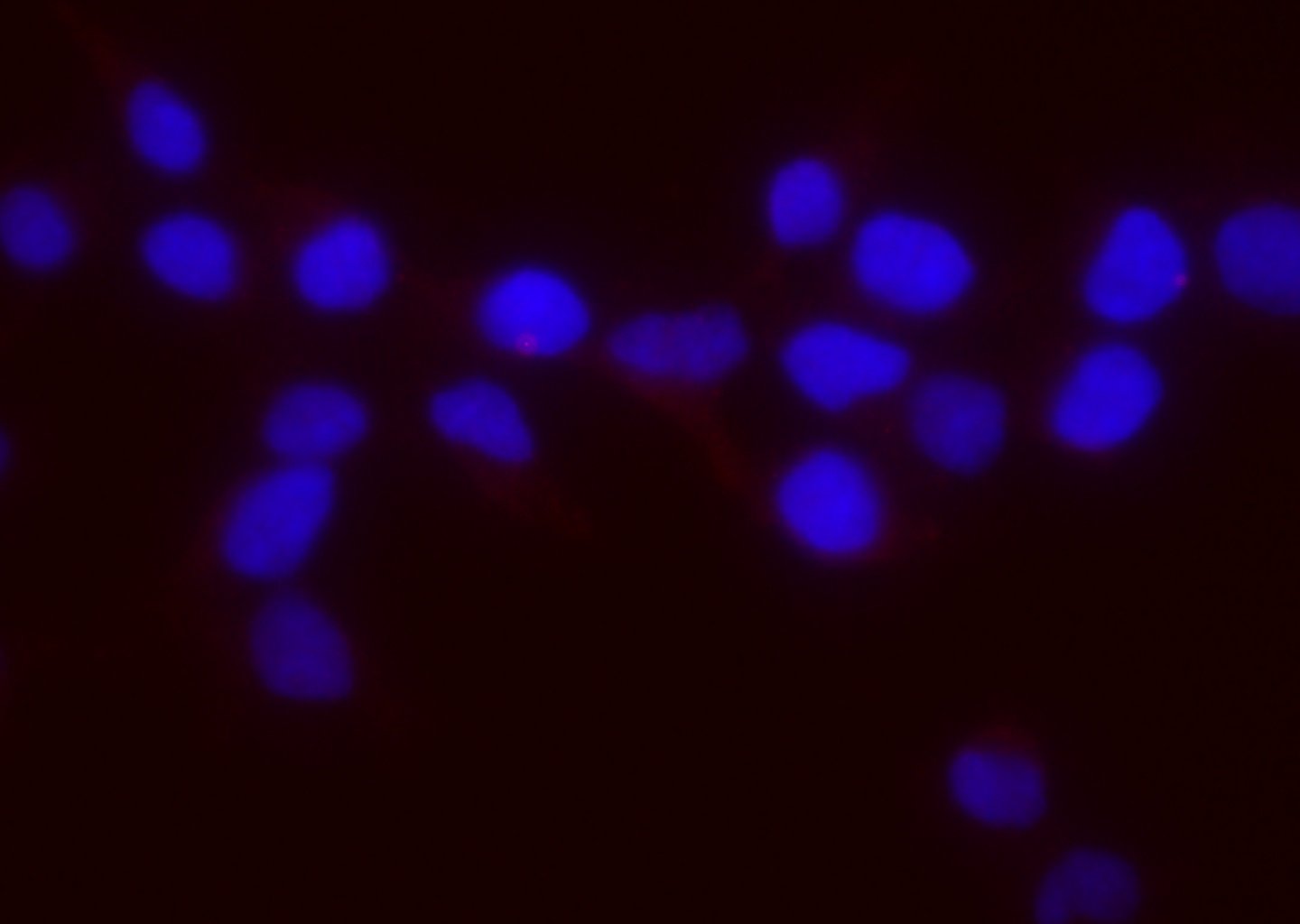

Supplement: Supplementary file 3 — Source Data Fig. 2 [file 44318_2024_35_MOESM3_ESM.zip › EMBOJ-2023-115792R2_SourceData_Fig2/Fig2A-B-C_microscopy/R2/N1 EV R2/N1 EV merge.jpg]

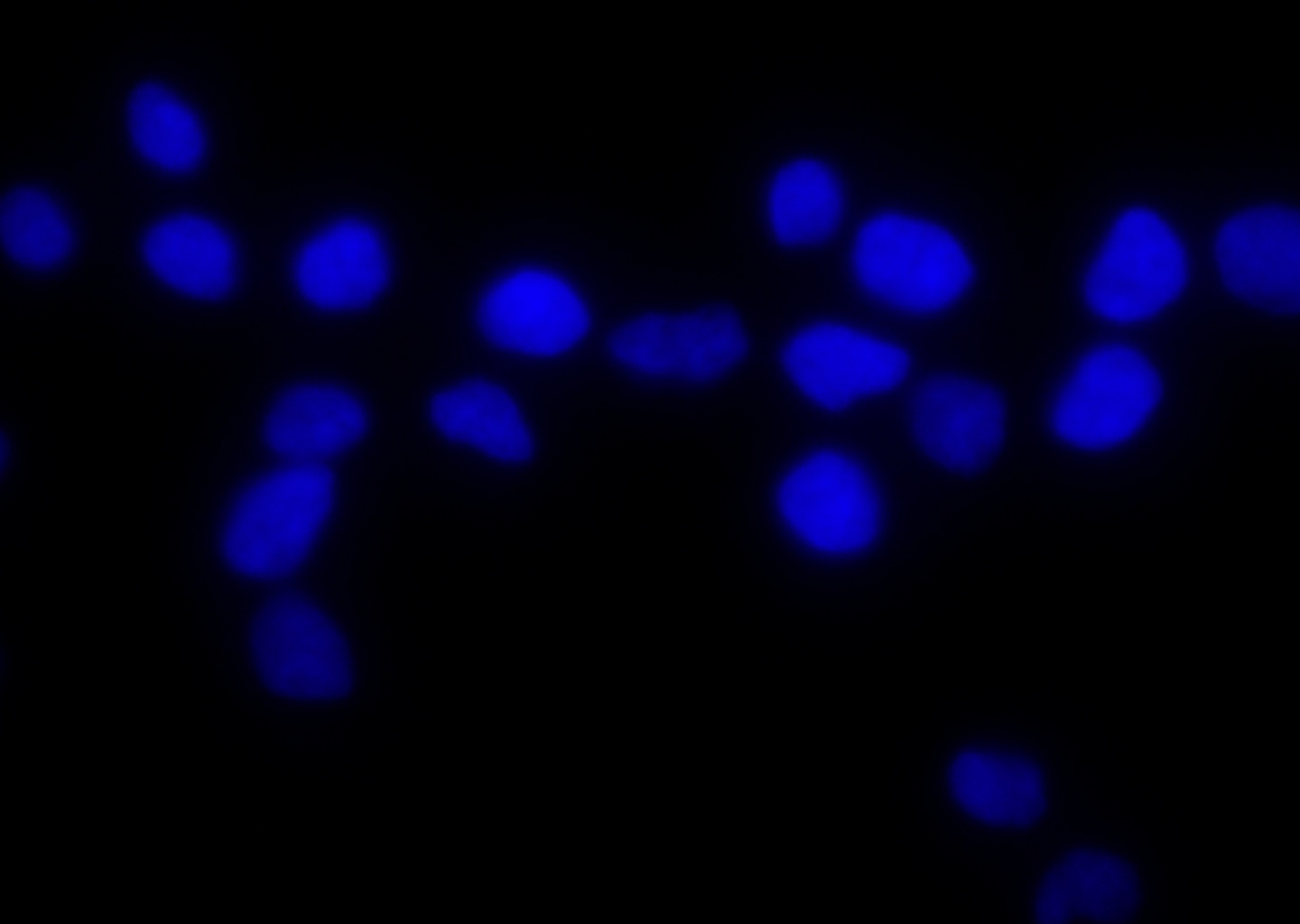

Supplement: Supplementary file 3 — Source Data Fig. 2 [file 44318_2024_35_MOESM3_ESM.zip › EMBOJ-2023-115792R2_SourceData_Fig2/Fig2A-B-C_microscopy/R2/N1 EV R2/DAPI.jpg]

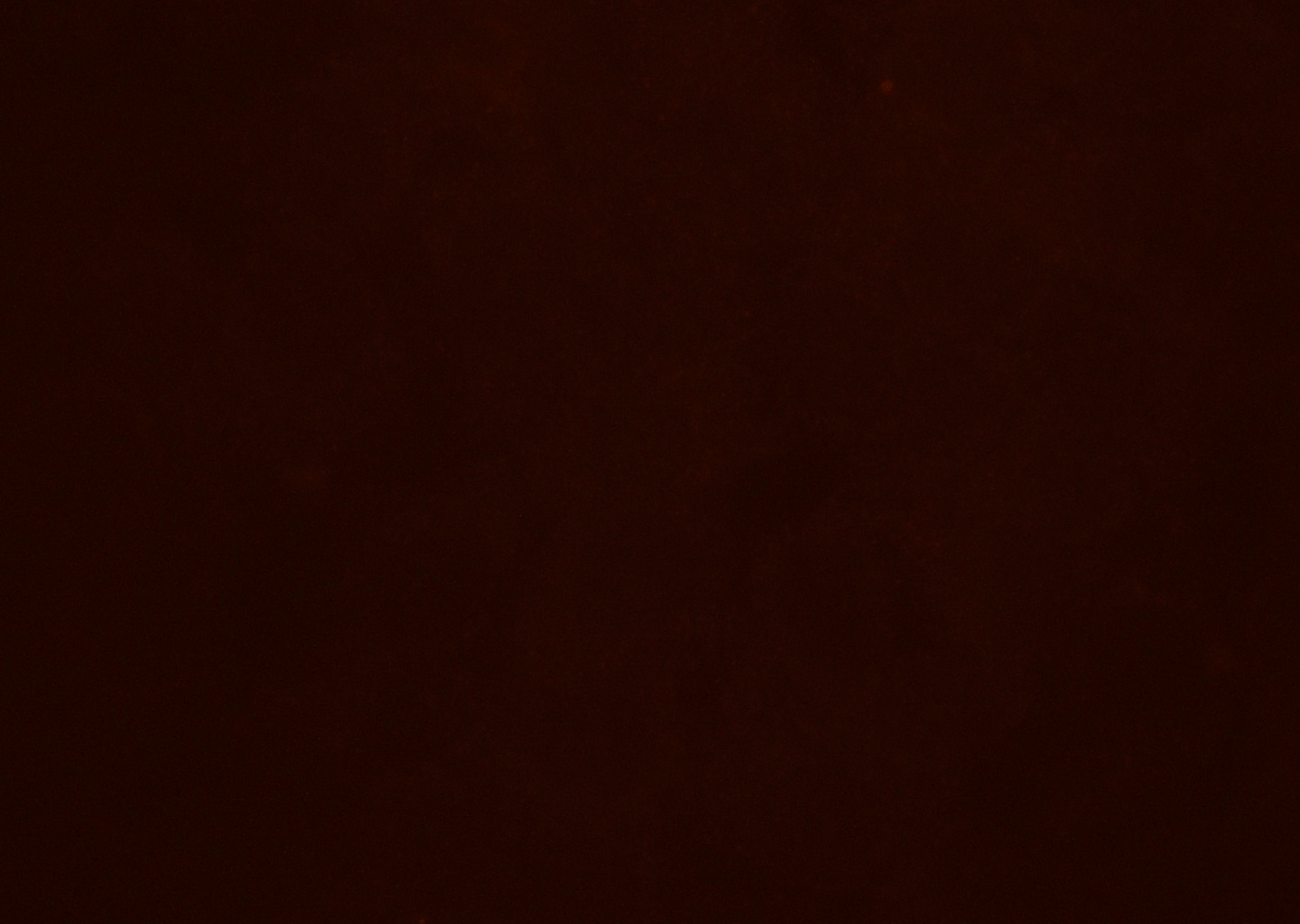

Supplement: Supplementary file 3 — Source Data Fig. 2 [file 44318_2024_35_MOESM3_ESM.zip › EMBOJ-2023-115792R2_SourceData_Fig2/Fig2A-B-C_microscopy/R2/N1 VSV R2/J2.jpg]

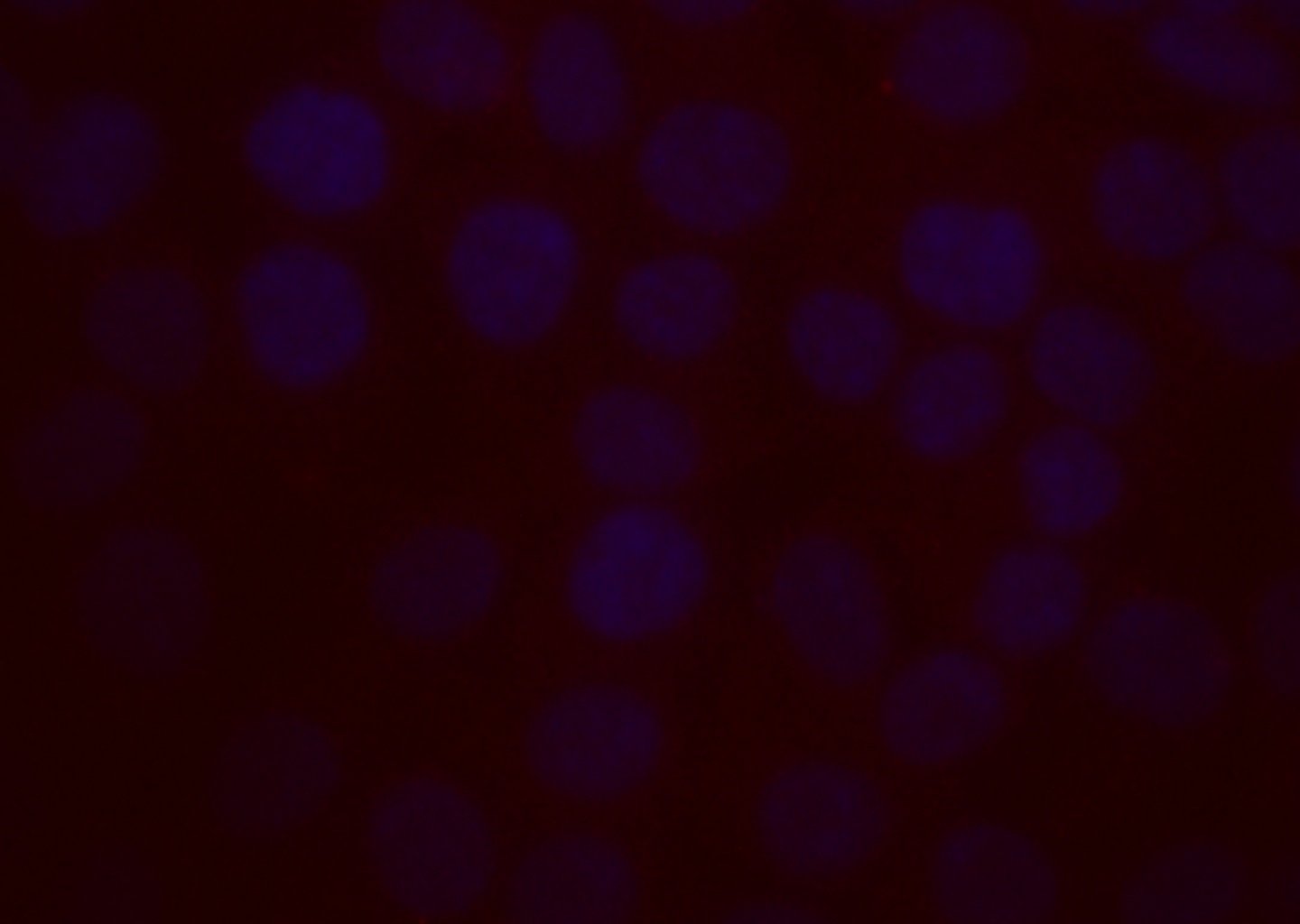

Supplement: Supplementary file 3 — Source Data Fig. 2 [file 44318_2024_35_MOESM3_ESM.zip › EMBOJ-2023-115792R2_SourceData_Fig2/Fig2A-B-C_microscopy/R2/N1 VSV R2/N1 VSV merge.jpg]

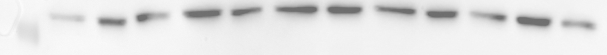

Supplement: Supplementary file 3 — Source Data Fig. 2 [file 44318_2024_35_MOESM3_ESM.zip › EMBOJ-2023-115792R2_SourceData_Fig2/Fig2D-E_western blot/R1/sars/western GAPDH.tiff]

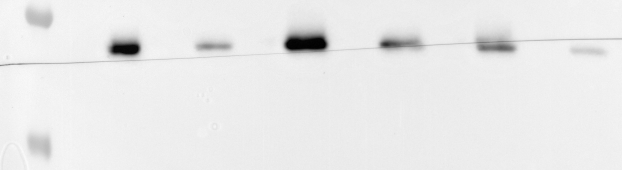

Supplement: Supplementary file 3 — Source Data Fig. 2 [file 44318_2024_35_MOESM3_ESM.zip › EMBOJ-2023-115792R2_SourceData_Fig2/Fig2D-E_western blot/R1/sars/western nucleocapsid.tiff]

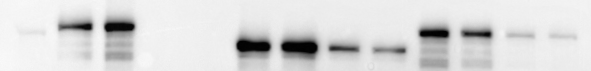

Supplement: Supplementary file 3 — Source Data Fig. 2 [file 44318_2024_35_MOESM3_ESM.zip › EMBOJ-2023-115792R2_SourceData_Fig2/Fig2D-E_western blot/R1/sars/western dicer.tiff]

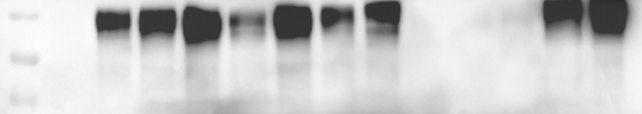

Supplement: Supplementary file 3 — Source Data Fig. 2 [file 44318_2024_35_MOESM3_ESM.zip › EMBOJ-2023-115792R2_SourceData_Fig2/Fig2D-E_western blot/R1/ace2/western ace2.tiff]

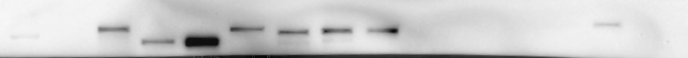

Supplement: Supplementary file 3 — Source Data Fig. 2 [file 44318_2024_35_MOESM3_ESM.zip › EMBOJ-2023-115792R2_SourceData_Fig2/Fig2D-E_western blot/R1/ace2/western HA.tiff]

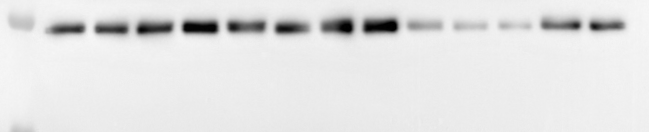

Supplement: Supplementary file 3 — Source Data Fig. 2 [file 44318_2024_35_MOESM3_ESM.zip › EMBOJ-2023-115792R2_SourceData_Fig2/Fig2D-E_western blot/R1/ace2/western tubulin.tiff]

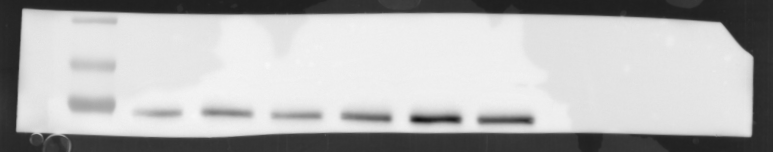

Supplement: Supplementary file 4 — Source Data Fig. 3 [file 44318_2024_35_MOESM4_ESM.zip › EMBOJ-2023-115792R2_SourceData_Fig3/Fig3D western blot/R1/western PKR.tiff]

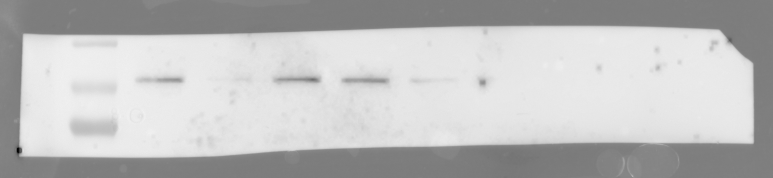

Supplement: Supplementary file 4 — Source Data Fig. 3 [file 44318_2024_35_MOESM4_ESM.zip › EMBOJ-2023-115792R2_SourceData_Fig3/Fig3D western blot/R1/western ago.tiff]

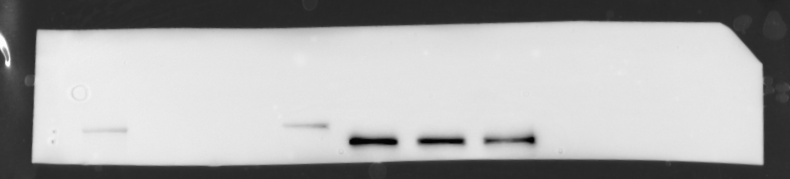

Supplement: Supplementary file 4 — Source Data Fig. 3 [file 44318_2024_35_MOESM4_ESM.zip › EMBOJ-2023-115792R2_SourceData_Fig3/Fig3D western blot/R1/western HA.tiff]

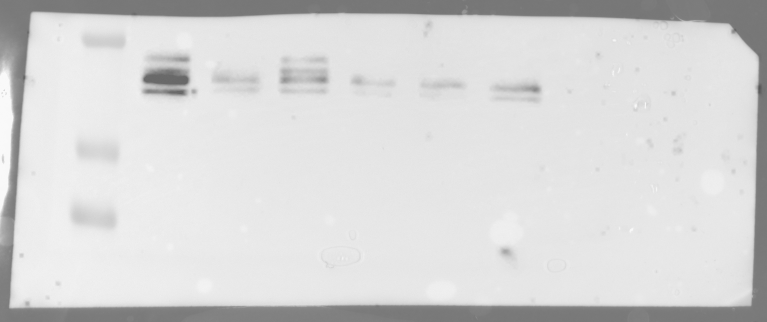

Supplement: Supplementary file 4 — Source Data Fig. 3 [file 44318_2024_35_MOESM4_ESM.zip › EMBOJ-2023-115792R2_SourceData_Fig3/Fig3D western blot/R1/western TRBP.tiff]

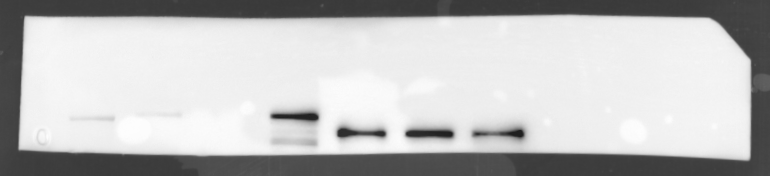

Supplement: Supplementary file 4 — Source Data Fig. 3 [file 44318_2024_35_MOESM4_ESM.zip › EMBOJ-2023-115792R2_SourceData_Fig3/Fig3D western blot/R1/western dicer.tiff]

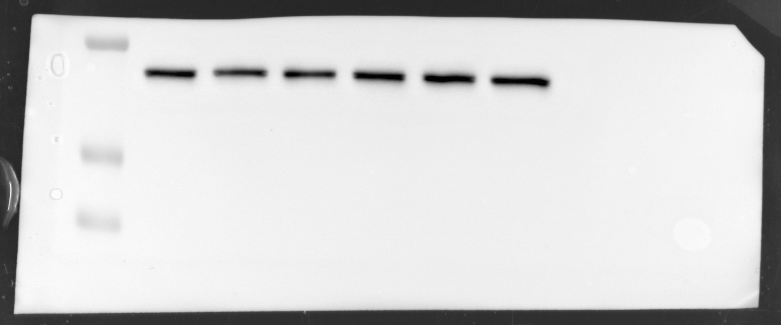

Supplement: Supplementary file 4 — Source Data Fig. 3 [file 44318_2024_35_MOESM4_ESM.zip › EMBOJ-2023-115792R2_SourceData_Fig3/Fig3D western blot/R1/western tubulin.tiff]

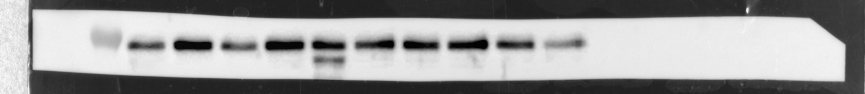

Supplement: Supplementary file 4 — Source Data Fig. 3 [file 44318_2024_35_MOESM4_ESM.zip › EMBOJ-2023-115792R2_SourceData_Fig3/Fig3D western blot/R3/western PKR.tiff]

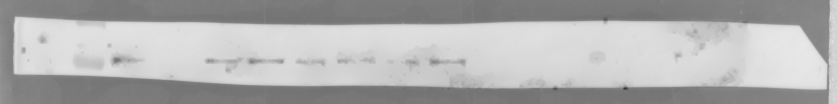

Supplement: Supplementary file 4 — Source Data Fig. 3 [file 44318_2024_35_MOESM4_ESM.zip › EMBOJ-2023-115792R2_SourceData_Fig3/Fig3D western blot/R3/western ago.tiff]

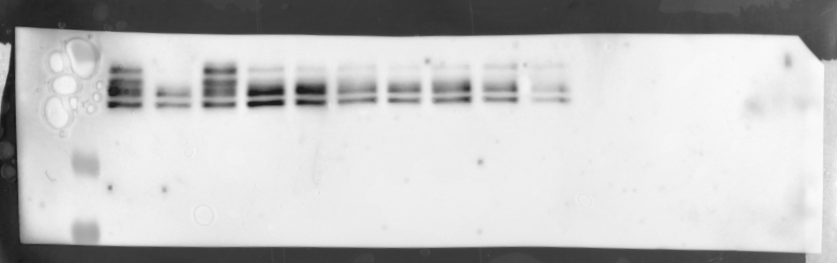

Supplement: Supplementary file 4 — Source Data Fig. 3 [file 44318_2024_35_MOESM4_ESM.zip › EMBOJ-2023-115792R2_SourceData_Fig3/Fig3D western blot/R3/western TRBP.tiff]

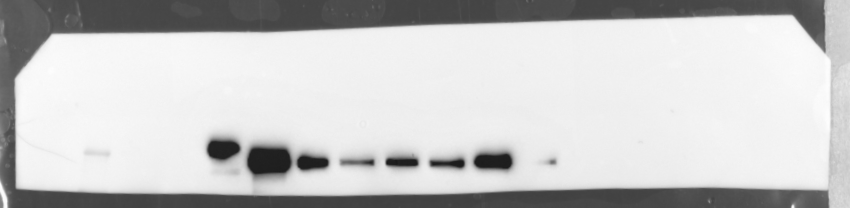

Supplement: Supplementary file 4 — Source Data Fig. 3 [file 44318_2024_35_MOESM4_ESM.zip › EMBOJ-2023-115792R2_SourceData_Fig3/Fig3D western blot/R3/western dicer.tiff]

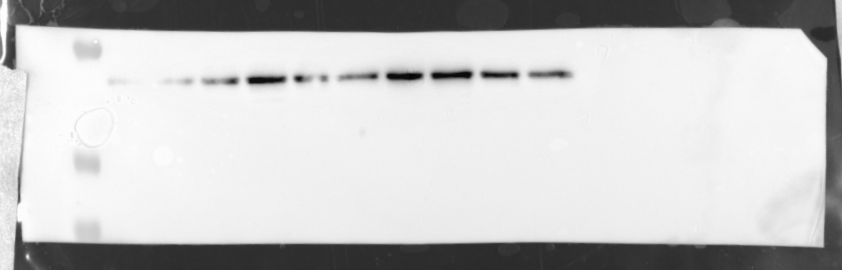

Supplement: Supplementary file 4 — Source Data Fig. 3 [file 44318_2024_35_MOESM4_ESM.zip › EMBOJ-2023-115792R2_SourceData_Fig3/Fig3D western blot/R3/western tubulin.tiff]

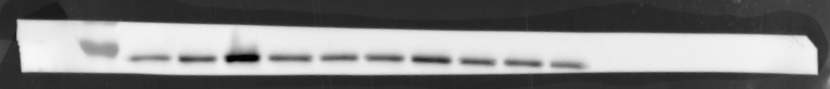

Supplement: Supplementary file 4 — Source Data Fig. 3 [file 44318_2024_35_MOESM4_ESM.zip › EMBOJ-2023-115792R2_SourceData_Fig3/Fig3D western blot/R2/western PKR.tiff]

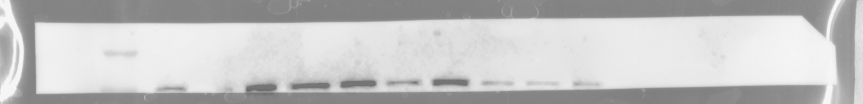

Supplement: Supplementary file 4 — Source Data Fig. 3 [file 44318_2024_35_MOESM4_ESM.zip › EMBOJ-2023-115792R2_SourceData_Fig3/Fig3D western blot/R2/western ago.tiff]

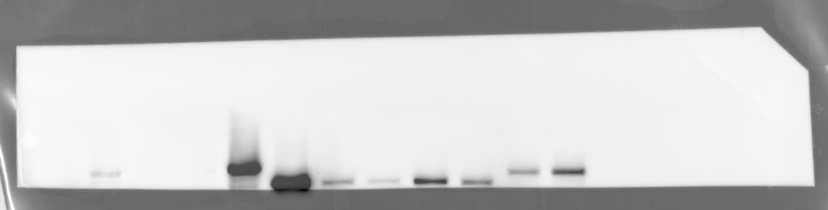

Supplement: Supplementary file 4 — Source Data Fig. 3 [file 44318_2024_35_MOESM4_ESM.zip › EMBOJ-2023-115792R2_SourceData_Fig3/Fig3D western blot/R2/western HA.tiff]

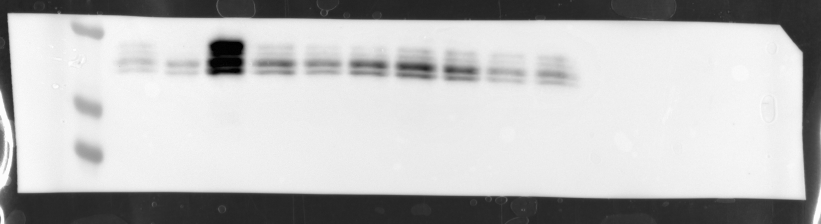

Supplement: Supplementary file 4 — Source Data Fig. 3 [file 44318_2024_35_MOESM4_ESM.zip › EMBOJ-2023-115792R2_SourceData_Fig3/Fig3D western blot/R2/western TRBP.tiff]

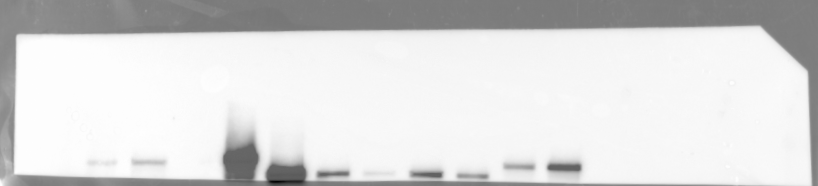

Supplement: Supplementary file 4 — Source Data Fig. 3 [file 44318_2024_35_MOESM4_ESM.zip › EMBOJ-2023-115792R2_SourceData_Fig3/Fig3D western blot/R2/western dicer.tiff]

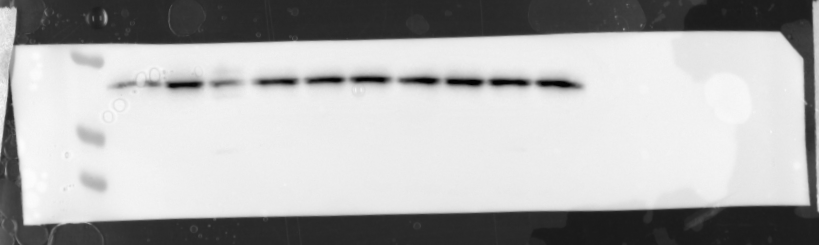

Supplement: Supplementary file 4 — Source Data Fig. 3 [file 44318_2024_35_MOESM4_ESM.zip › EMBOJ-2023-115792R2_SourceData_Fig3/Fig3D western blot/R2/western tubulin.tiff]

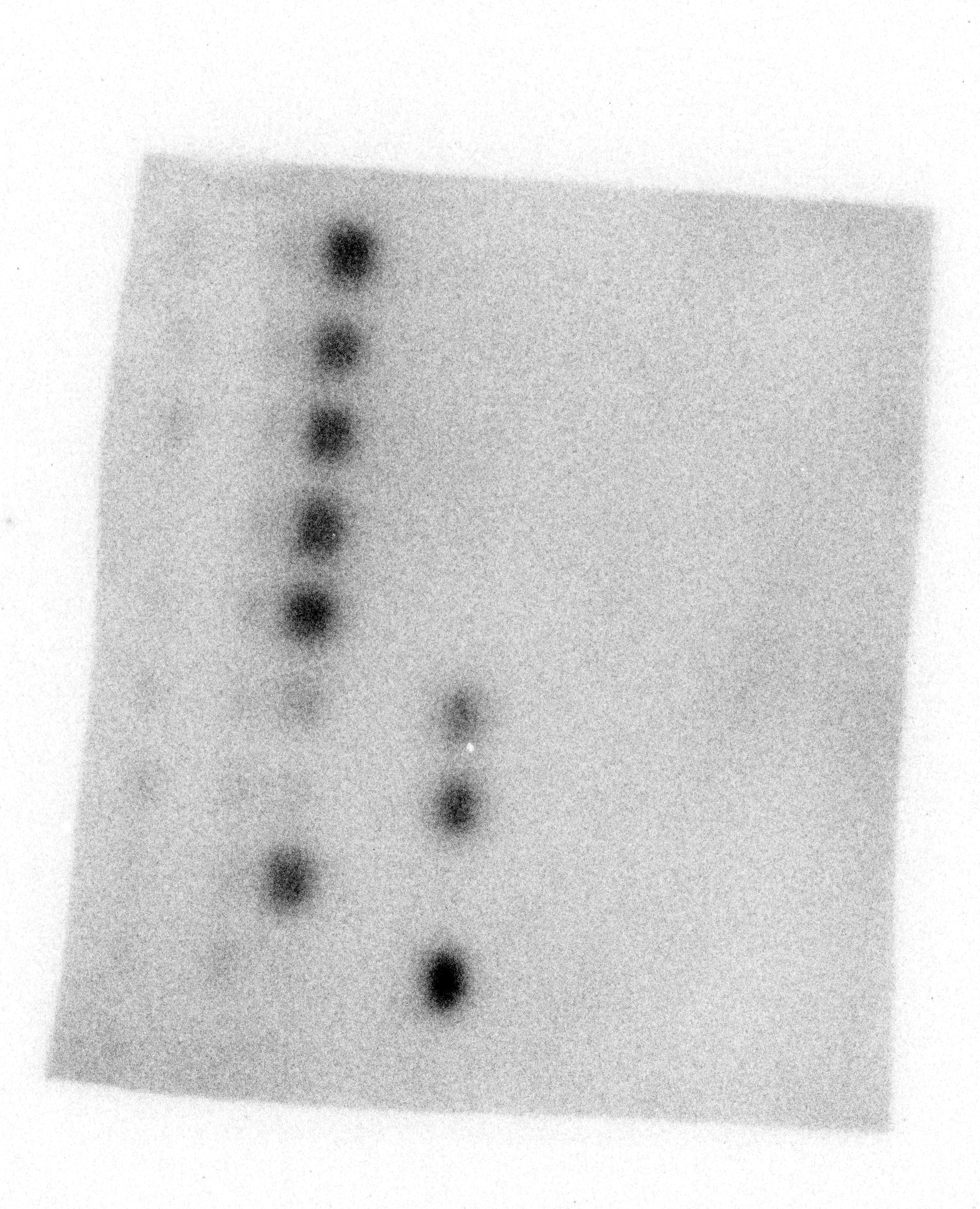

Supplement: Supplementary file 4 — Source Data Fig. 3 [file 44318_2024_35_MOESM4_ESM.zip › EMBOJ-2023-115792R2_SourceData_Fig3/Fig3E northern blot/R1/northern miR16.tiff]

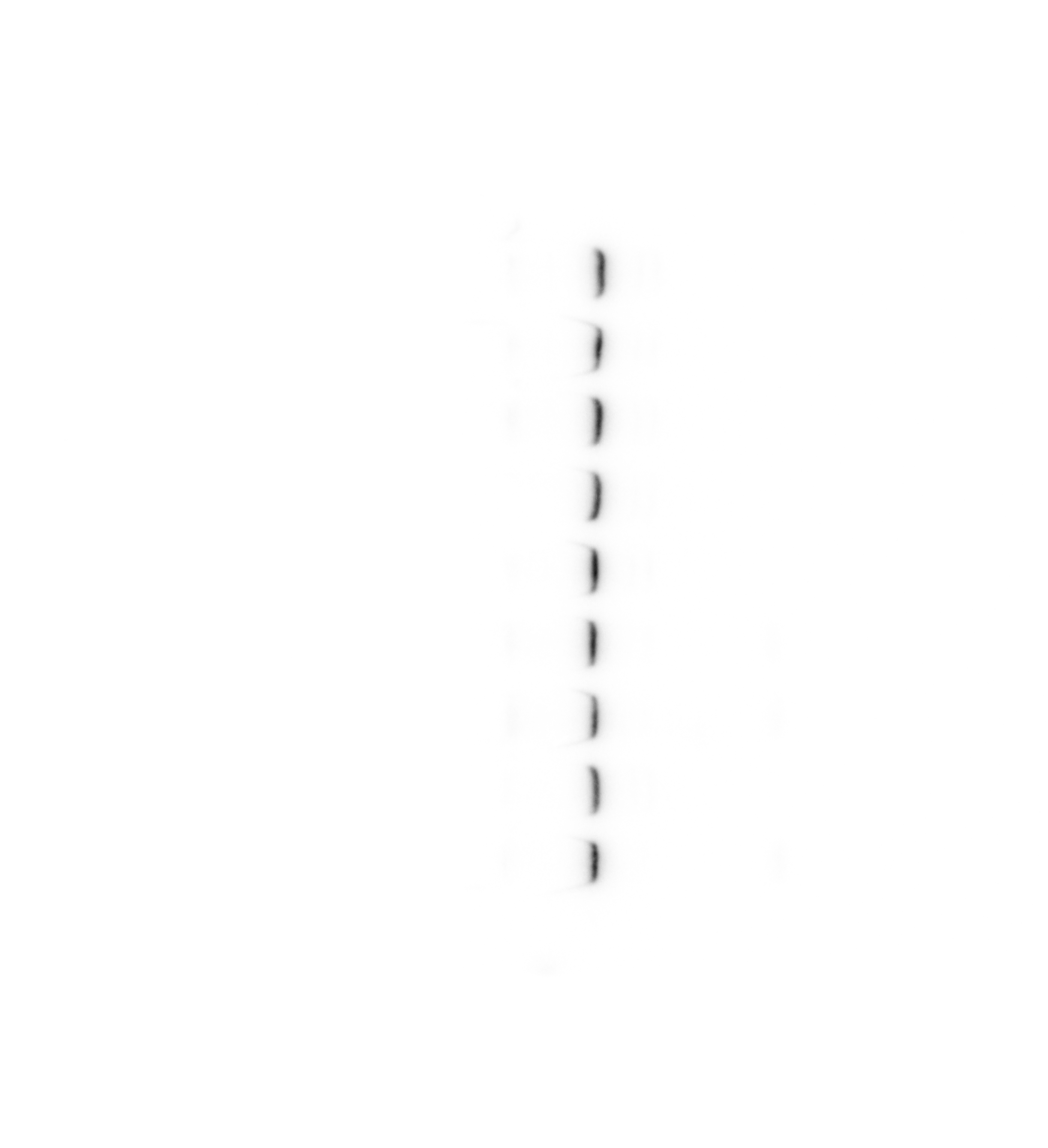

Supplement: Supplementary file 4 — Source Data Fig. 3 [file 44318_2024_35_MOESM4_ESM.zip › EMBOJ-2023-115792R2_SourceData_Fig3/Fig3E northern blot/R1/northern U6.tiff]

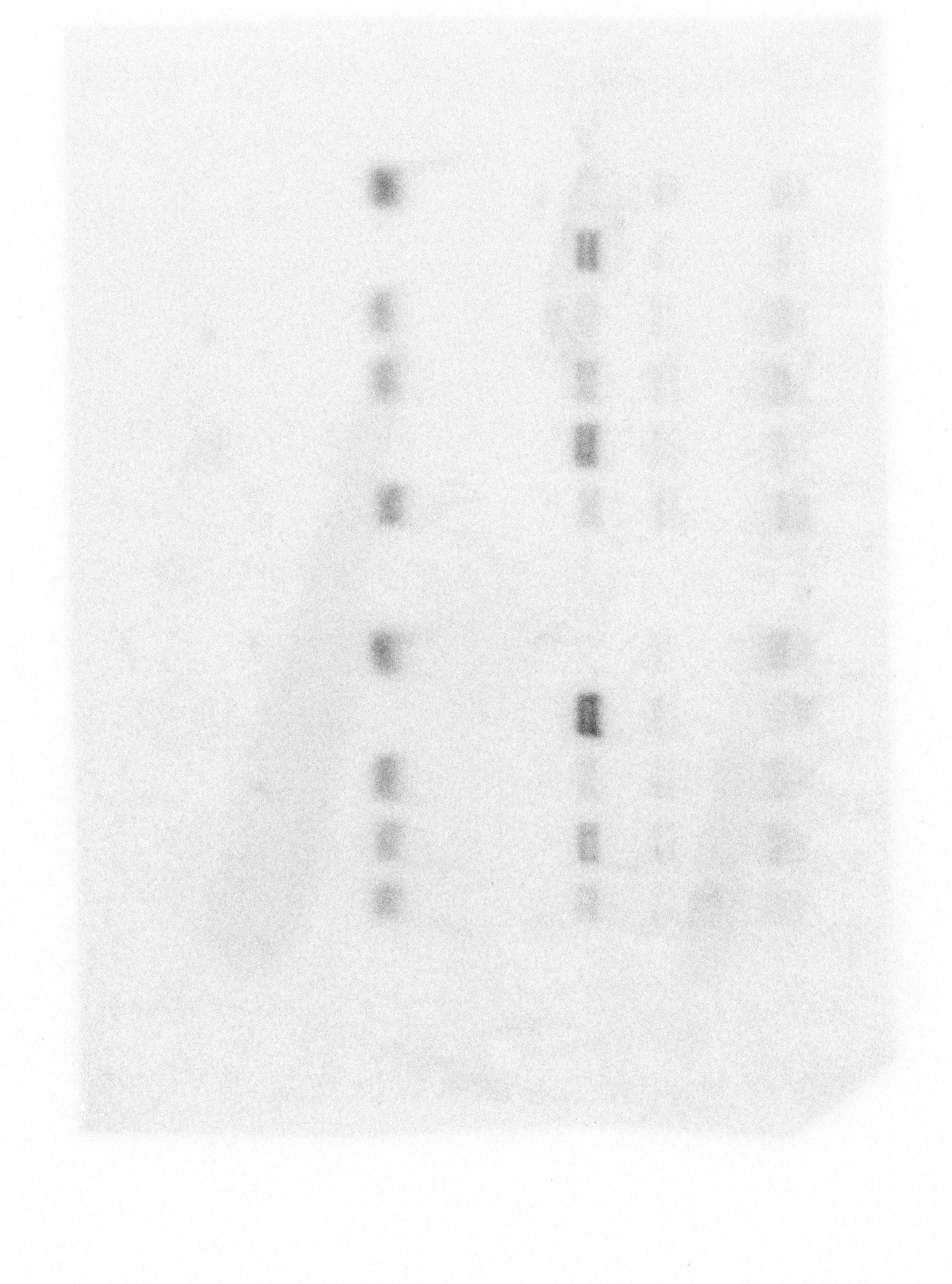

Supplement: Supplementary file 4 — Source Data Fig. 3 [file 44318_2024_35_MOESM4_ESM.zip › EMBOJ-2023-115792R2_SourceData_Fig3/Fig3E northern blot/R3/northern miR16.tiff]

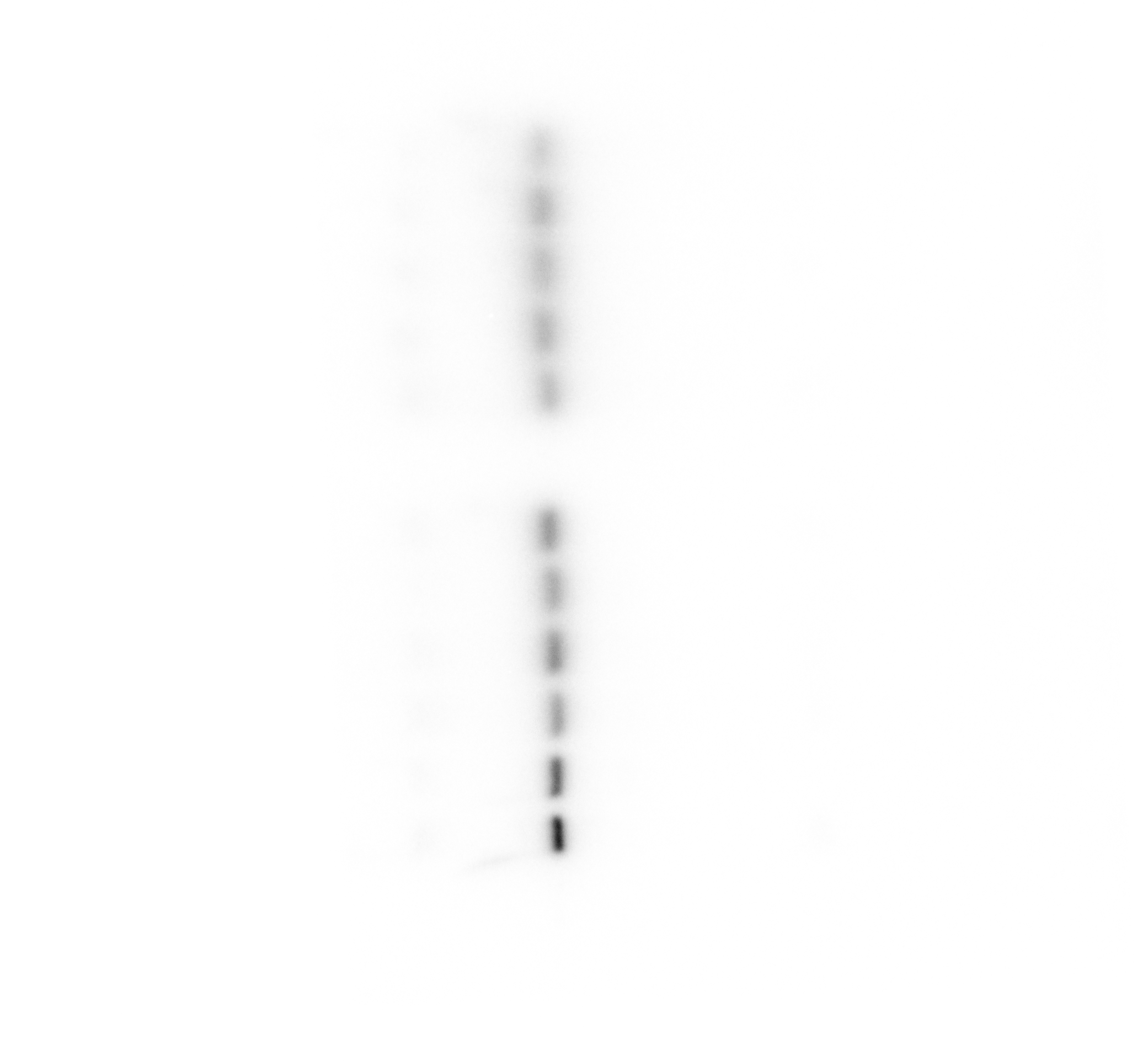

Supplement: Supplementary file 4 — Source Data Fig. 3 [file 44318_2024_35_MOESM4_ESM.zip › EMBOJ-2023-115792R2_SourceData_Fig3/Fig3E northern blot/R3/northern U6.tiff]

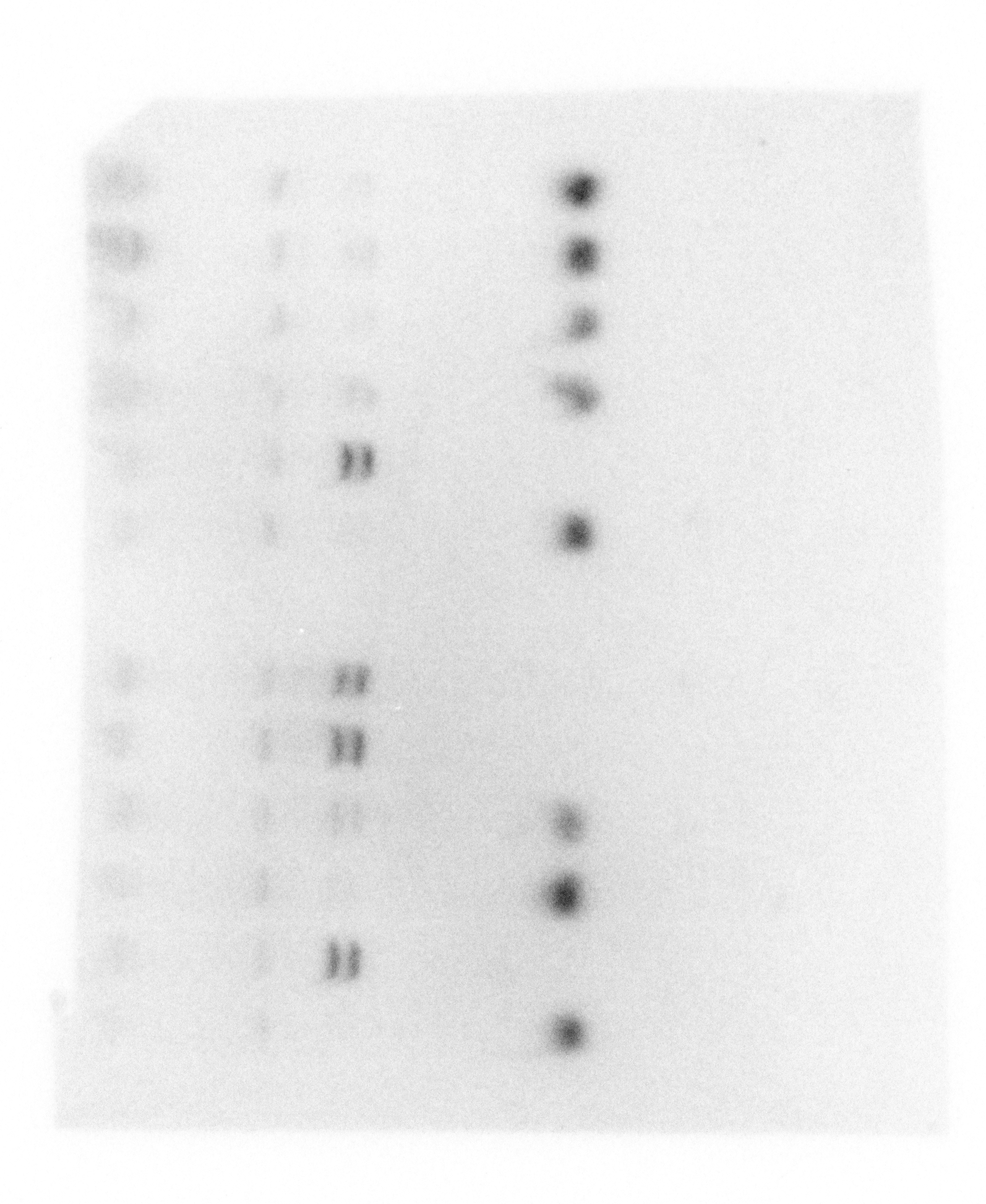

Supplement: Supplementary file 4 — Source Data Fig. 3 [file 44318_2024_35_MOESM4_ESM.zip › EMBOJ-2023-115792R2_SourceData_Fig3/Fig3E northern blot/R2/northern miR16.tiff]

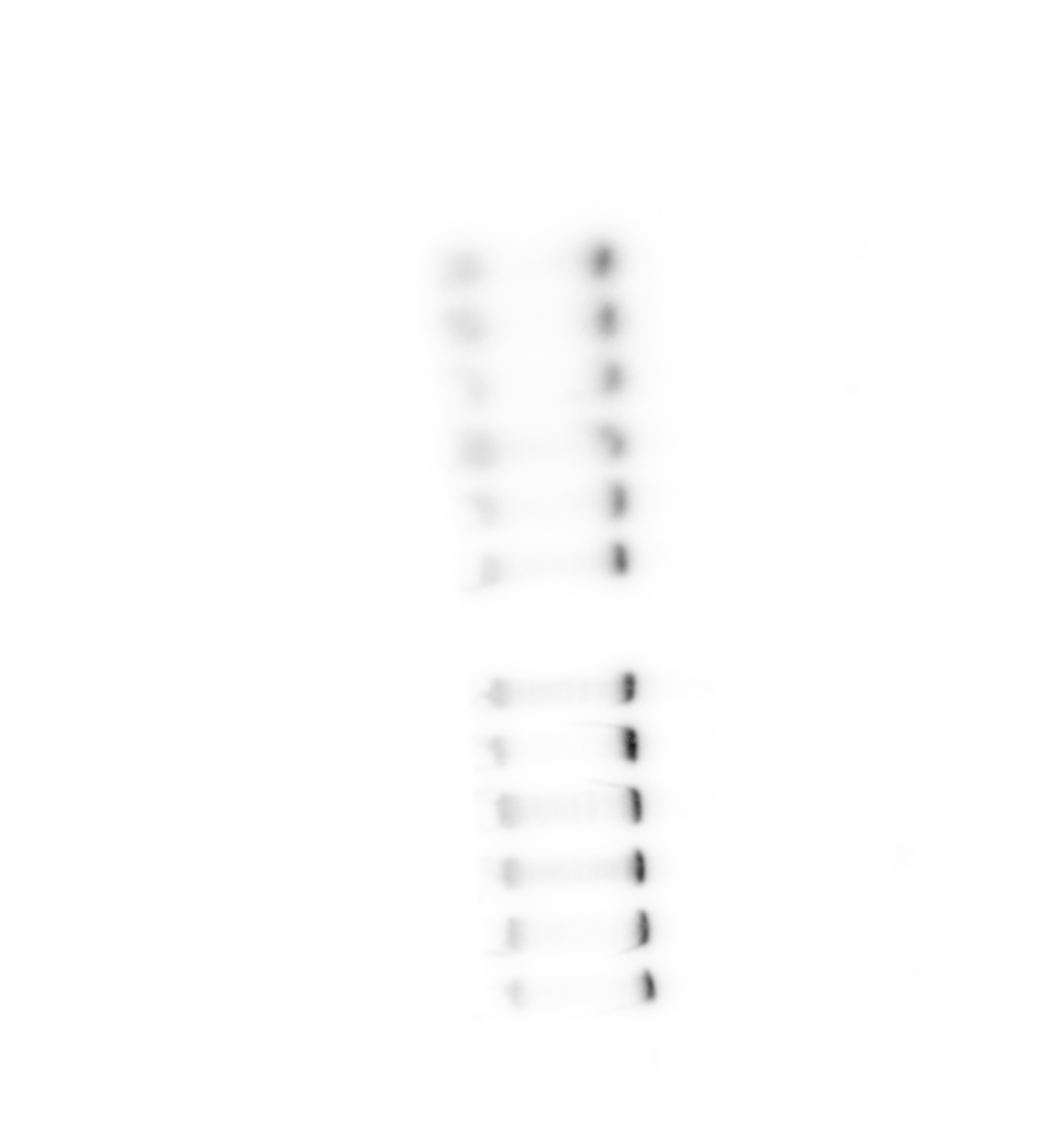

Supplement: Supplementary file 4 — Source Data Fig. 3 [file 44318_2024_35_MOESM4_ESM.zip › EMBOJ-2023-115792R2_SourceData_Fig3/Fig3E northern blot/R2/northern U6.tiff]

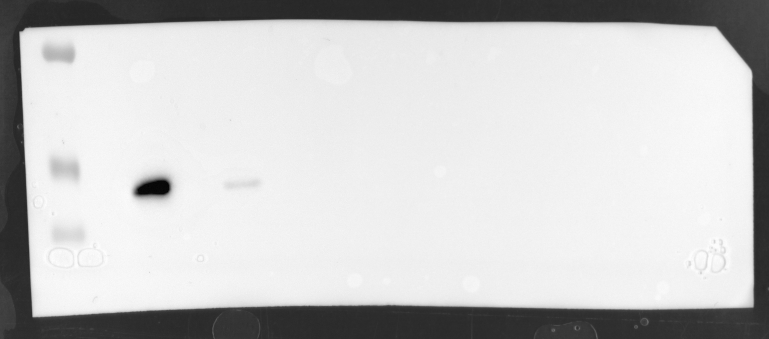

Supplement: Supplementary file 4 — Source Data Fig. 3 [file 44318_2024_35_MOESM4_ESM.zip › EMBOJ-2023-115792R2_SourceData_Fig3/Fig3F western blot/R1/western capsid.tiff]

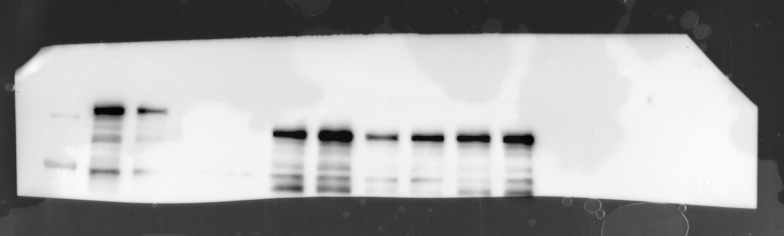

Supplement: Supplementary file 4 — Source Data Fig. 3 [file 44318_2024_35_MOESM4_ESM.zip › EMBOJ-2023-115792R2_SourceData_Fig3/Fig3F western blot/R1/western dicer.tiff]

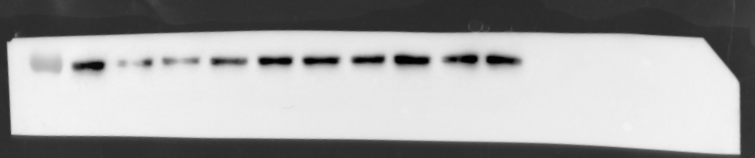

Supplement: Supplementary file 4 — Source Data Fig. 3 [file 44318_2024_35_MOESM4_ESM.zip › EMBOJ-2023-115792R2_SourceData_Fig3/Fig3F western blot/R1/western tubulin.tiff]

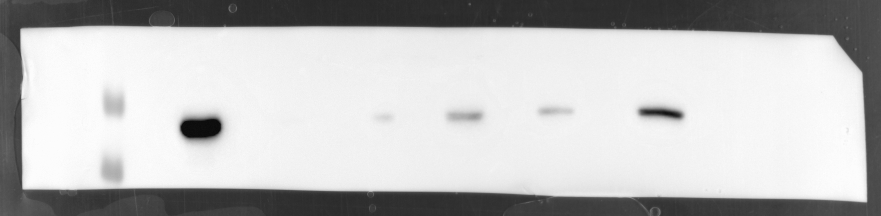

Supplement: Supplementary file 4 — Source Data Fig. 3 [file 44318_2024_35_MOESM4_ESM.zip › EMBOJ-2023-115792R2_SourceData_Fig3/Fig3F western blot/R3/western capsid.tiff]

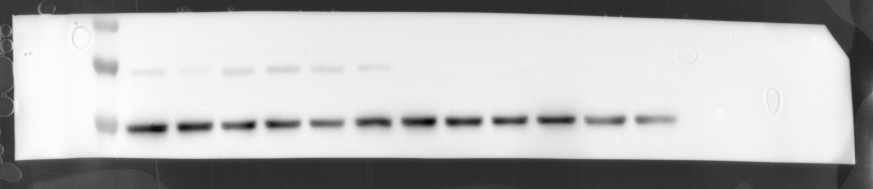

Supplement: Supplementary file 4 — Source Data Fig. 3 [file 44318_2024_35_MOESM4_ESM.zip › EMBOJ-2023-115792R2_SourceData_Fig3/Fig3F western blot/R3/western tubulin.tiff]

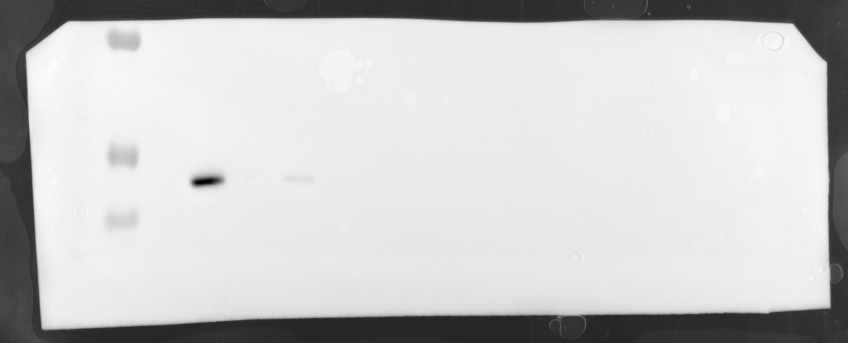

Supplement: Supplementary file 4 — Source Data Fig. 3 [file 44318_2024_35_MOESM4_ESM.zip › EMBOJ-2023-115792R2_SourceData_Fig3/Fig3F western blot/R2/western capsid.tiff]

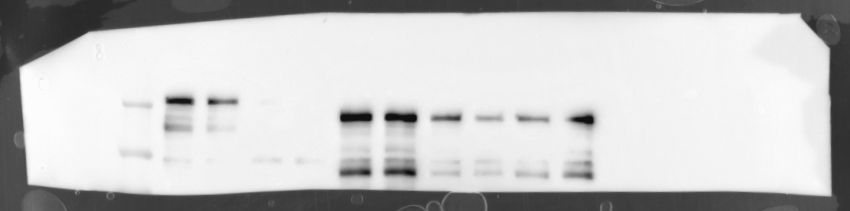

Supplement: Supplementary file 4 — Source Data Fig. 3 [file 44318_2024_35_MOESM4_ESM.zip › EMBOJ-2023-115792R2_SourceData_Fig3/Fig3F western blot/R2/western dicer.tiff]

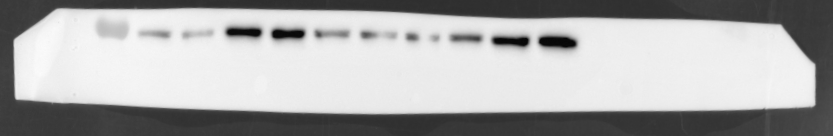

Supplement: Supplementary file 4 — Source Data Fig. 3 [file 44318_2024_35_MOESM4_ESM.zip › EMBOJ-2023-115792R2_SourceData_Fig3/Fig3F western blot/R2/western tubulin.tiff]

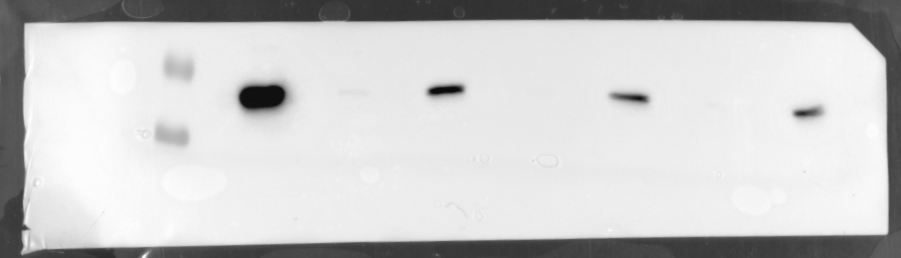

Supplement: Supplementary file 5 — Source Data Fig. 4 [file 44318_2024_35_MOESM5_ESM.zip › EMBOJ-2023-115792R2_SourceData_Fig4/Fig4B_western blot/R1/western capsid.tiff]

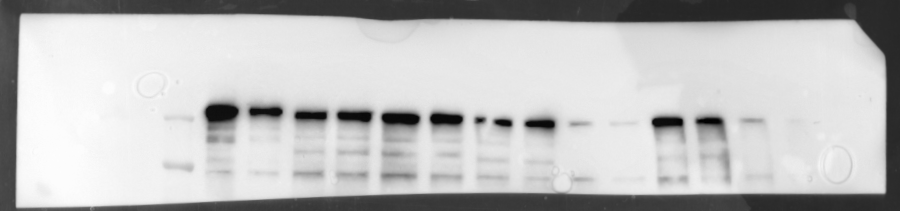

Supplement: Supplementary file 5 — Source Data Fig. 4 [file 44318_2024_35_MOESM5_ESM.zip › EMBOJ-2023-115792R2_SourceData_Fig4/Fig4B_western blot/R1/western dicer.tiff]

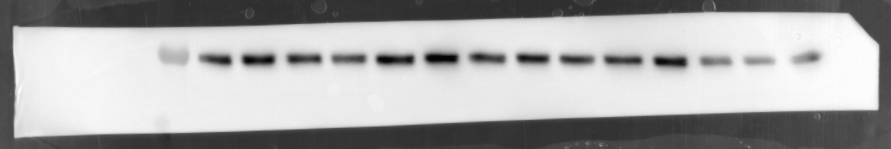

Supplement: Supplementary file 5 — Source Data Fig. 4 [file 44318_2024_35_MOESM5_ESM.zip › EMBOJ-2023-115792R2_SourceData_Fig4/Fig4B_western blot/R1/western tubulin.tiff]

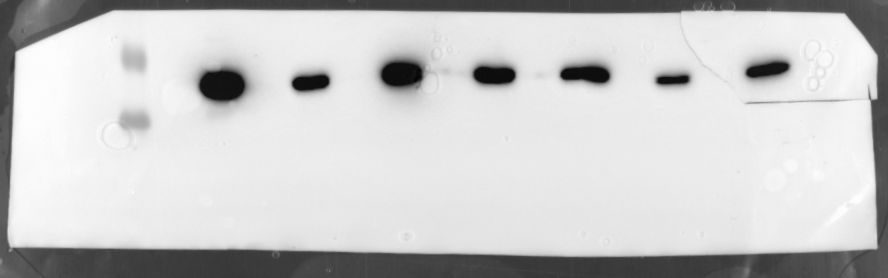

Supplement: Supplementary file 5 — Source Data Fig. 4 [file 44318_2024_35_MOESM5_ESM.zip › EMBOJ-2023-115792R2_SourceData_Fig4/Fig4B_western blot/R3 /western capsid.tiff]

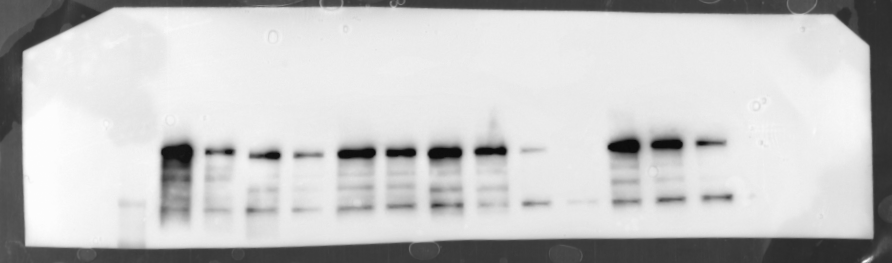

Supplement: Supplementary file 5 — Source Data Fig. 4 [file 44318_2024_35_MOESM5_ESM.zip › EMBOJ-2023-115792R2_SourceData_Fig4/Fig4B_western blot/R3 /western dicer.tiff]

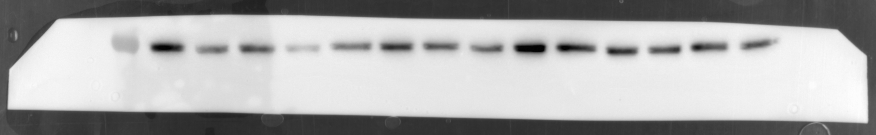

Supplement: Supplementary file 5 — Source Data Fig. 4 [file 44318_2024_35_MOESM5_ESM.zip › EMBOJ-2023-115792R2_SourceData_Fig4/Fig4B_western blot/R3 /western tubulin.tiff]

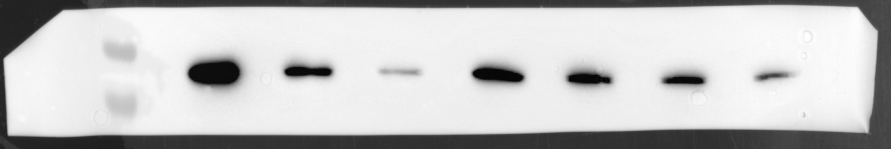

Supplement: Supplementary file 5 — Source Data Fig. 4 [file 44318_2024_35_MOESM5_ESM.zip › EMBOJ-2023-115792R2_SourceData_Fig4/Fig4B_western blot/R2/western capsid.tiff]

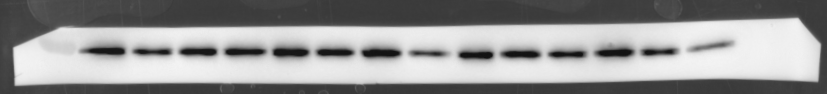

Supplement: Supplementary file 5 — Source Data Fig. 4 [file 44318_2024_35_MOESM5_ESM.zip › EMBOJ-2023-115792R2_SourceData_Fig4/Fig4B_western blot/R2/western tubulin.tiff]

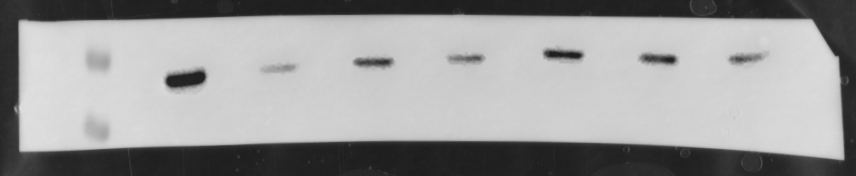

Supplement: Supplementary file 5 — Source Data Fig. 4 [file 44318_2024_35_MOESM5_ESM.zip › EMBOJ-2023-115792R2_SourceData_Fig4/FIG4D_western blot/R1/NoDice/western capsid.tiff]

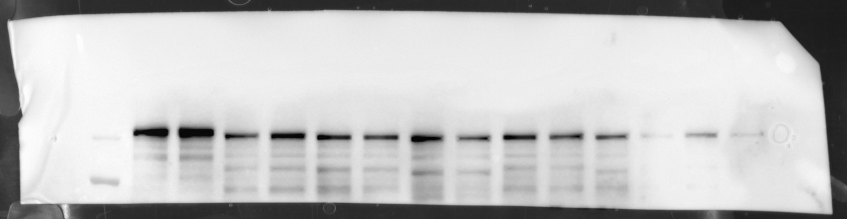

Supplement: Supplementary file 5 — Source Data Fig. 4 [file 44318_2024_35_MOESM5_ESM.zip › EMBOJ-2023-115792R2_SourceData_Fig4/FIG4D_western blot/R1/NoDice/western dicer.tiff]

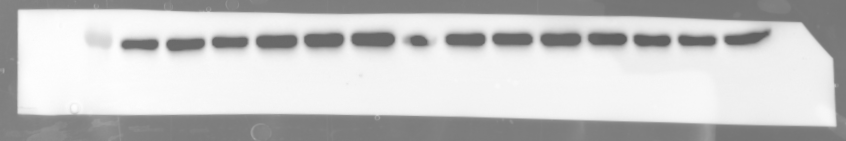

Supplement: Supplementary file 5 — Source Data Fig. 4 [file 44318_2024_35_MOESM5_ESM.zip › EMBOJ-2023-115792R2_SourceData_Fig4/FIG4D_western blot/R1/NoDice/western tubulin.tiff]

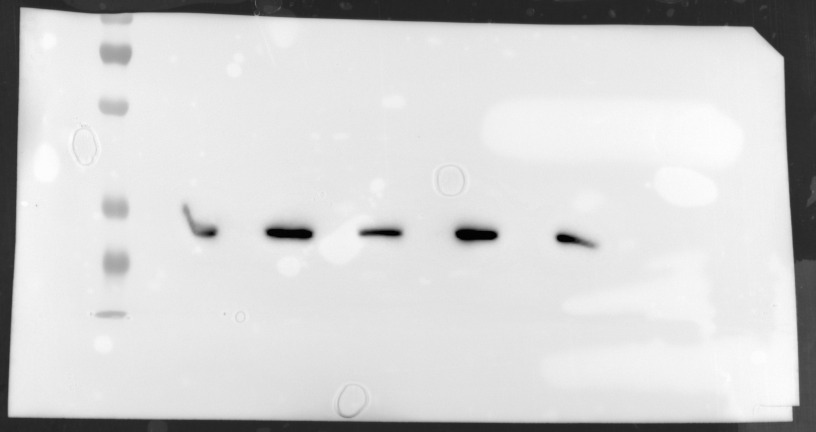

Supplement: Supplementary file 5 — Source Data Fig. 4 [file 44318_2024_35_MOESM5_ESM.zip › EMBOJ-2023-115792R2_SourceData_Fig4/FIG4D_western blot/R1/NoDiceΓêåPKR/western capsid.tiff]

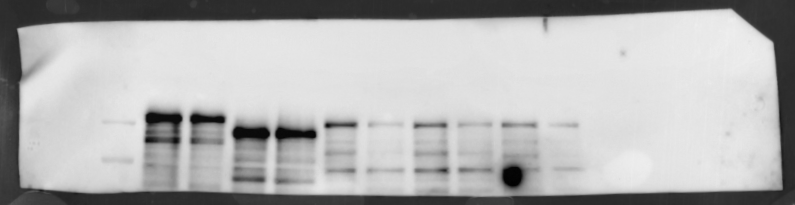

Supplement: Supplementary file 5 — Source Data Fig. 4 [file 44318_2024_35_MOESM5_ESM.zip › EMBOJ-2023-115792R2_SourceData_Fig4/FIG4D_western blot/R1/NoDiceΓêåPKR/western dicer.tiff]

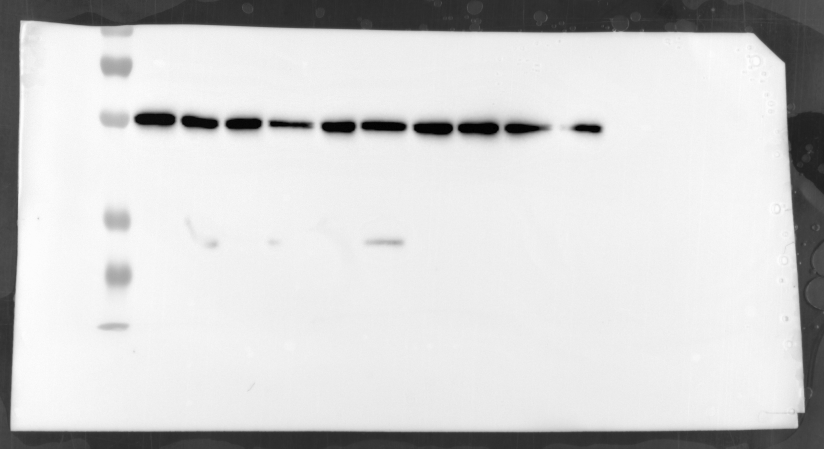

Supplement: Supplementary file 5 — Source Data Fig. 4 [file 44318_2024_35_MOESM5_ESM.zip › EMBOJ-2023-115792R2_SourceData_Fig4/FIG4D_western blot/R1/NoDiceΓêåPKR/western tubulin.tiff]

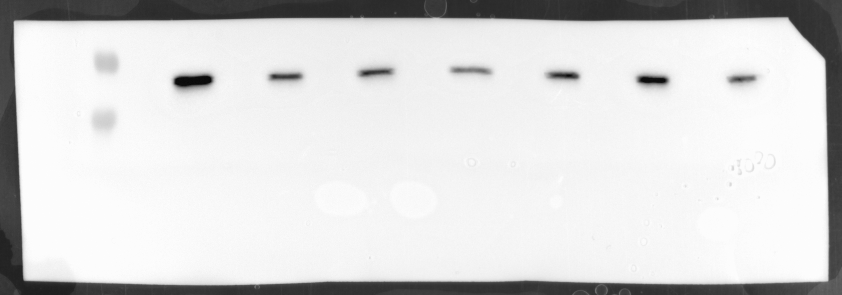

Supplement: Supplementary file 5 — Source Data Fig. 4 [file 44318_2024_35_MOESM5_ESM.zip › EMBOJ-2023-115792R2_SourceData_Fig4/FIG4D_western blot/R3/Nodice/western capsid.tiff]

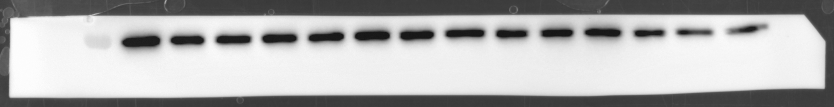

Supplement: Supplementary file 5 — Source Data Fig. 4 [file 44318_2024_35_MOESM5_ESM.zip › EMBOJ-2023-115792R2_SourceData_Fig4/FIG4D_western blot/R3/Nodice/western tubulin.tiff]

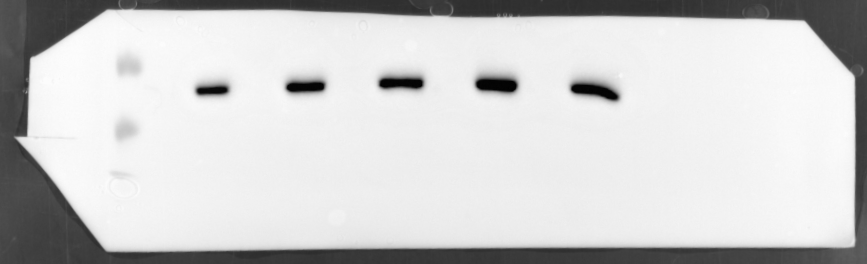

Supplement: Supplementary file 5 — Source Data Fig. 4 [file 44318_2024_35_MOESM5_ESM.zip › EMBOJ-2023-115792R2_SourceData_Fig4/FIG4D_western blot/R3/NoDiceΓêåPKR/western capsid.tiff]

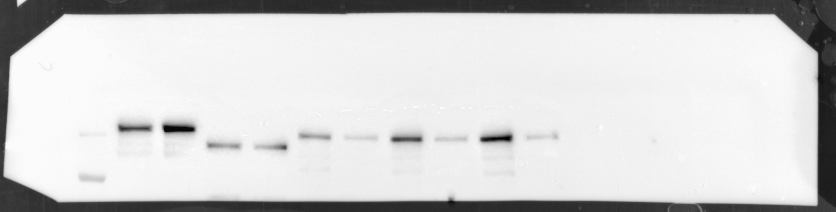

Supplement: Supplementary file 5 — Source Data Fig. 4 [file 44318_2024_35_MOESM5_ESM.zip › EMBOJ-2023-115792R2_SourceData_Fig4/FIG4D_western blot/R3/NoDiceΓêåPKR/western dicer.tiff]

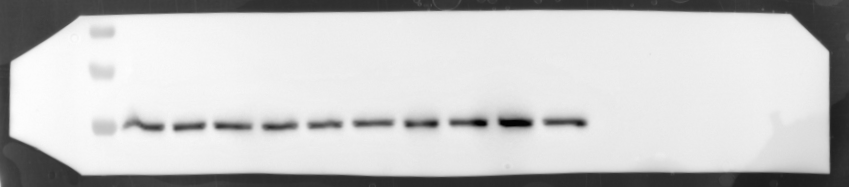

Supplement: Supplementary file 5 — Source Data Fig. 4 [file 44318_2024_35_MOESM5_ESM.zip › EMBOJ-2023-115792R2_SourceData_Fig4/FIG4D_western blot/R3/NoDiceΓêåPKR/western tubulin.tiff]

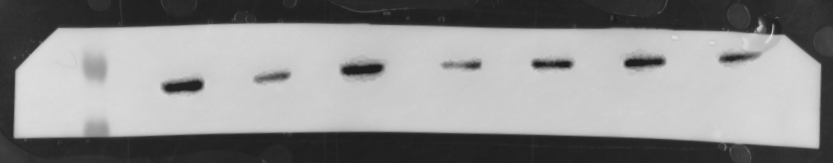

Supplement: Supplementary file 5 — Source Data Fig. 4 [file 44318_2024_35_MOESM5_ESM.zip › EMBOJ-2023-115792R2_SourceData_Fig4/FIG4D_western blot/R2/NoDice/western capsid.tiff]

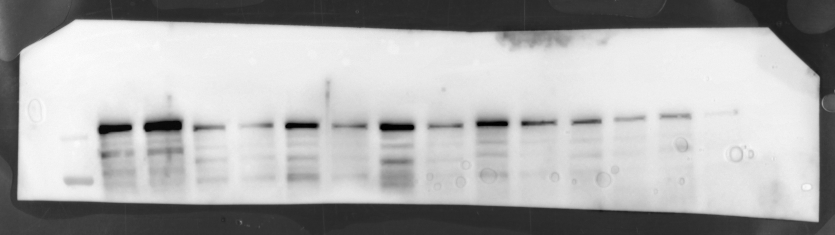

Supplement: Supplementary file 5 — Source Data Fig. 4 [file 44318_2024_35_MOESM5_ESM.zip › EMBOJ-2023-115792R2_SourceData_Fig4/FIG4D_western blot/R2/NoDice/western dicer.tiff]

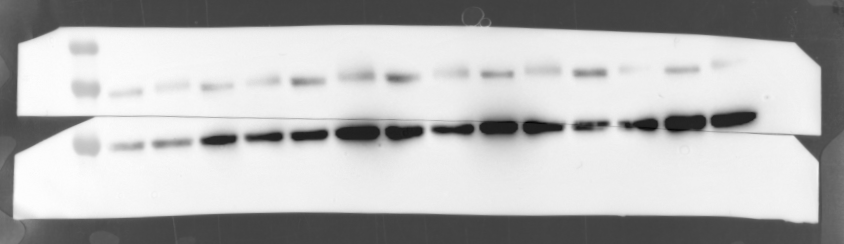

Supplement: Supplementary file 5 — Source Data Fig. 4 [file 44318_2024_35_MOESM5_ESM.zip › EMBOJ-2023-115792R2_SourceData_Fig4/FIG4D_western blot/R2/NoDice/western tubulin.tiff]

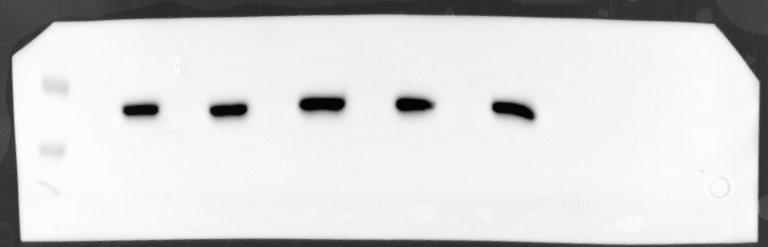

Supplement: Supplementary file 5 — Source Data Fig. 4 [file 44318_2024_35_MOESM5_ESM.zip › EMBOJ-2023-115792R2_SourceData_Fig4/FIG4D_western blot/R2/NoDiceΓêåPKR/western capsid.tiff]

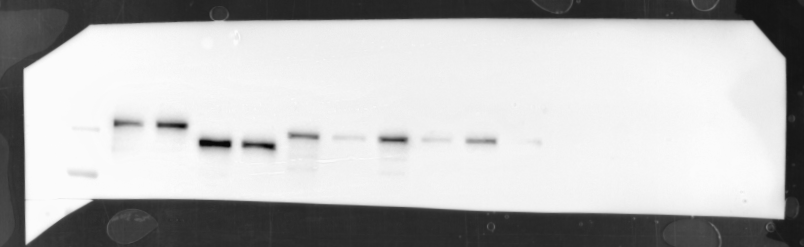

Supplement: Supplementary file 5 — Source Data Fig. 4 [file 44318_2024_35_MOESM5_ESM.zip › EMBOJ-2023-115792R2_SourceData_Fig4/FIG4D_western blot/R2/NoDiceΓêåPKR/western dicer.tiff]

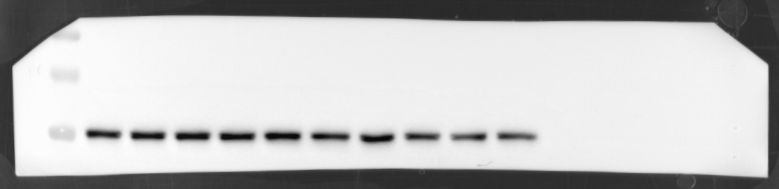

Supplement: Supplementary file 5 — Source Data Fig. 4 [file 44318_2024_35_MOESM5_ESM.zip › EMBOJ-2023-115792R2_SourceData_Fig4/FIG4D_western blot/R2/NoDiceΓêåPKR/western tubulin.tiff]

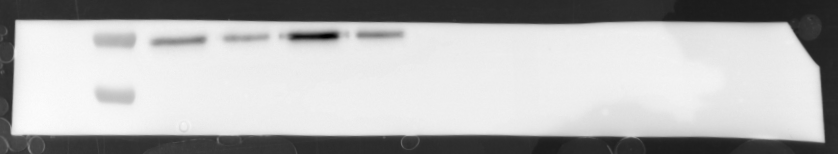

Supplement: Supplementary file 6 — Source Data Fig. 5 [file 44318_2024_35_MOESM6_ESM.zip › EMBOJ-2023-115792R2_SourceData_Fig5/5F western blot/R1/western PKR.tiff]

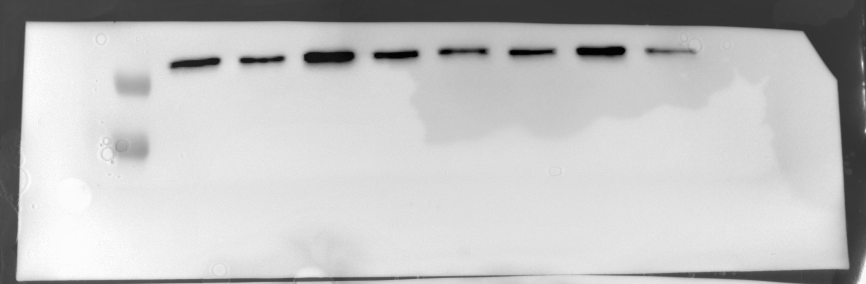

Supplement: Supplementary file 6 — Source Data Fig. 5 [file 44318_2024_35_MOESM6_ESM.zip › EMBOJ-2023-115792R2_SourceData_Fig5/5F western blot/R1/western gapdh.tiff]

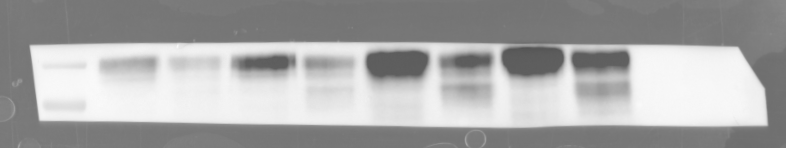

Supplement: Supplementary file 6 — Source Data Fig. 5 [file 44318_2024_35_MOESM6_ESM.zip › EMBOJ-2023-115792R2_SourceData_Fig5/5F western blot/R1/western ace2.tiff]

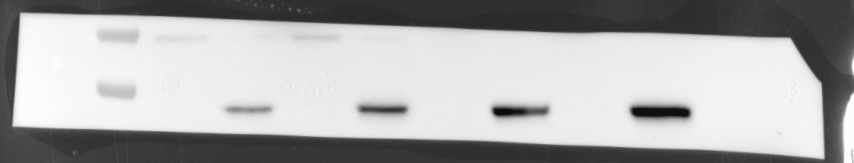

Supplement: Supplementary file 6 — Source Data Fig. 5 [file 44318_2024_35_MOESM6_ESM.zip › EMBOJ-2023-115792R2_SourceData_Fig5/5F western blot/R1/western nucleocapsid.tiff]

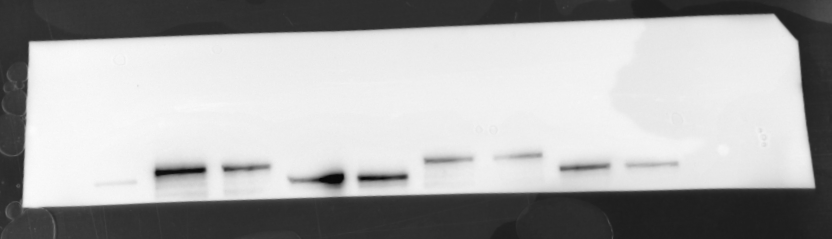

Supplement: Supplementary file 6 — Source Data Fig. 5 [file 44318_2024_35_MOESM6_ESM.zip › EMBOJ-2023-115792R2_SourceData_Fig5/5F western blot/R1/western dicer.tiff]

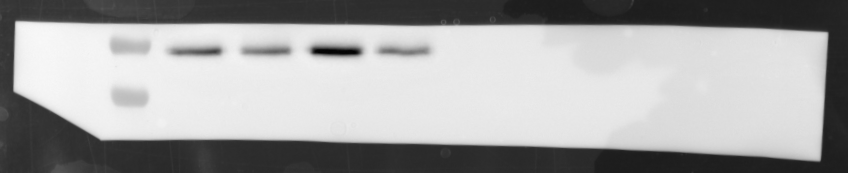

Supplement: Supplementary file 6 — Source Data Fig. 5 [file 44318_2024_35_MOESM6_ESM.zip › EMBOJ-2023-115792R2_SourceData_Fig5/5F western blot/R3/western pkr.tiff]

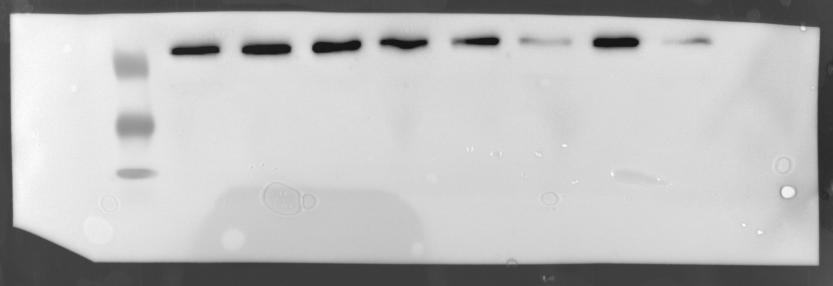

Supplement: Supplementary file 6 — Source Data Fig. 5 [file 44318_2024_35_MOESM6_ESM.zip › EMBOJ-2023-115792R2_SourceData_Fig5/5F western blot/R3/western gapdh.tiff]
