# Supplementary material for: The helicase domain of human Dicer prevents RNAi-independent activation of antiviral and inflammatory pathways
Source: EMBO J. 2024 Jan 29;43(5):7. doi: 10.1038/s44318-024-00035-2 (PMC10907635; doi:10.1038/s44318-024-00035-2)

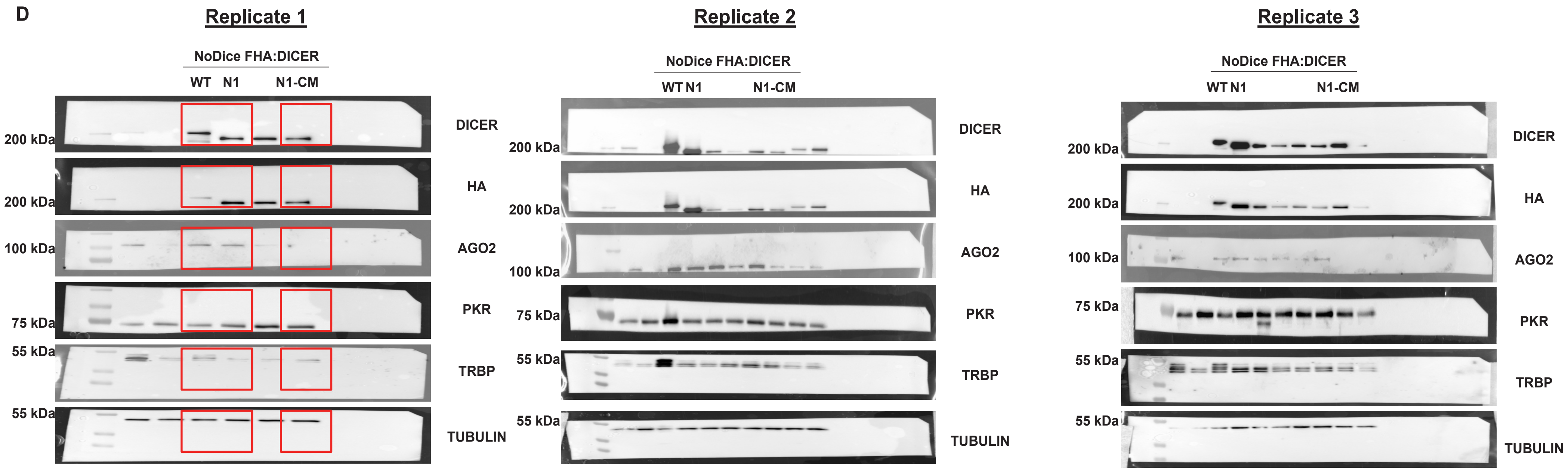

D
Replicate 1
NoDice FHA:DICER
WT N1 N1-CM
200 kDa
200 kDa
100 kDa
75 kDa
55 kDa
55 kDa
DICER
HA
AGO2
PKR
TRBP
TUBULIN
Replicate 2
NoDice FHA:DICER
WT N1 N1-CM
200 kDa
200 kDa
100 kDa
75 kDa
55 kDa
55 kDa
DICER
HA
AGO2
PKR
TRBP
TUBULIN
Replicate 3
NoDice FHA:DICER
WT N1 N1-CM
200 kDa
200 kDa
100 kDa
75 kDa
55 kDa
55 kDa
DICER
HA
AGO2
PKR
TRBP
TUBULIN

E

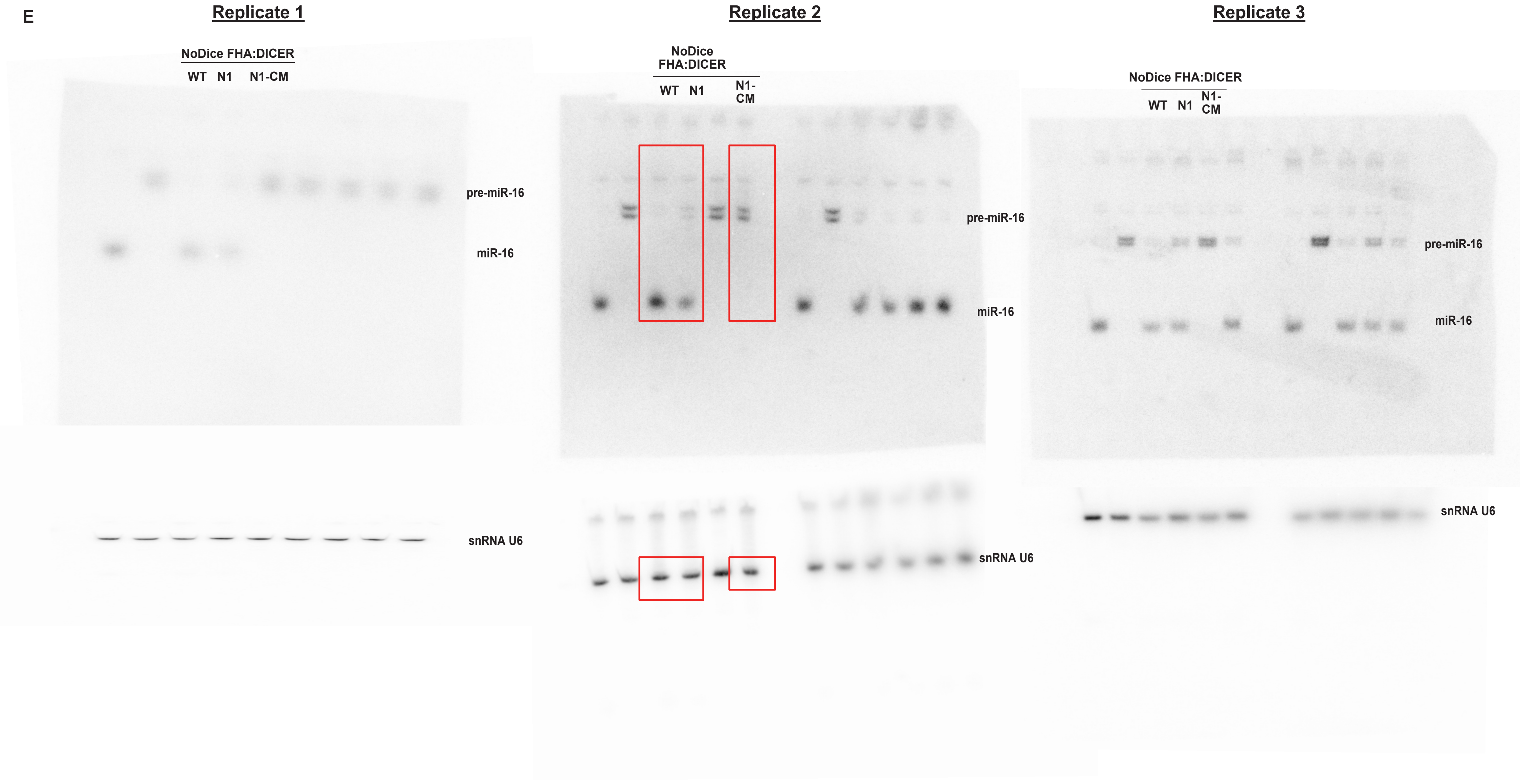
Replicate 1
NoDice FHA:DICER
WT
N1
N1-CM
pre-miR-16
miR-16
snRNA U6
Replicate 2
NoDice FHA:DICER
WT
N1
N1-CM
pre-miR-16
miR-16
snRNA U6
Replicate 3
NoDice FHA:DICER
WT
N1
N1-CM
pre-miR-16
miR-16
snRNA U6

F

Replicate 1

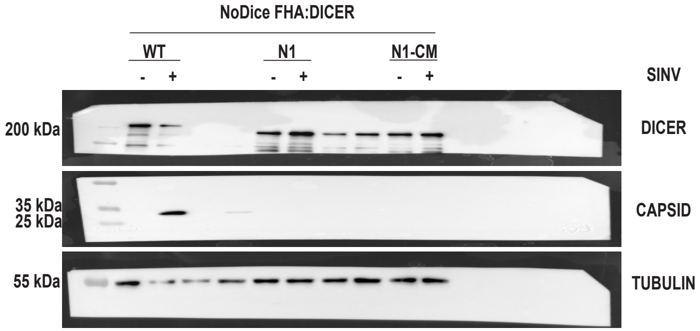

Replicate 2

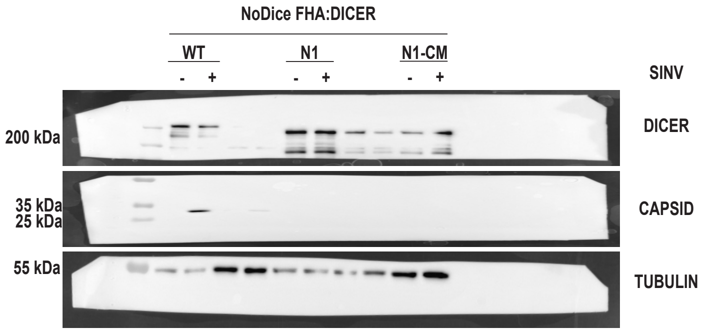

Replicate 3

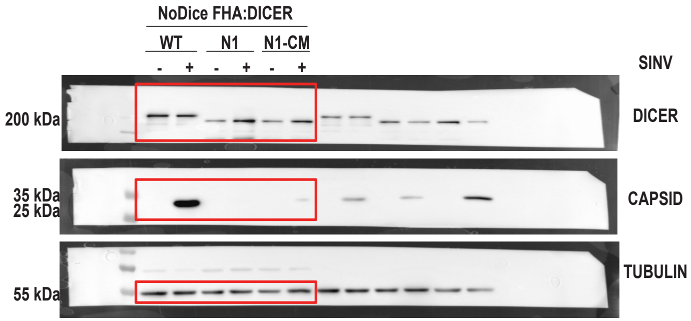

Supplement: Supplementary file 4 — Source Data Fig. 3 [file 44318_2024_35_MOESM4_ESM.zip › EMBOJ-2023-115792R2_SourceData_Fig3/Figure3.pdf]
