# Supplementary material for: The helicase domain of human Dicer prevents RNAi-independent activation of antiviral and inflammatory pathways
Source: EMBO J. 2024 Jan 29;43(5):7. doi: 10.1038/s44318-024-00035-2 (PMC10907635; doi:10.1038/s44318-024-00035-2)
Supplement: Supplementary file 5 — Source Data Fig. 4 [file 44318_2024_35_MOESM5_ESM.zip › EMBOJ-2023-115792R2_SourceData_Fig4/Figure4.pdf]

B

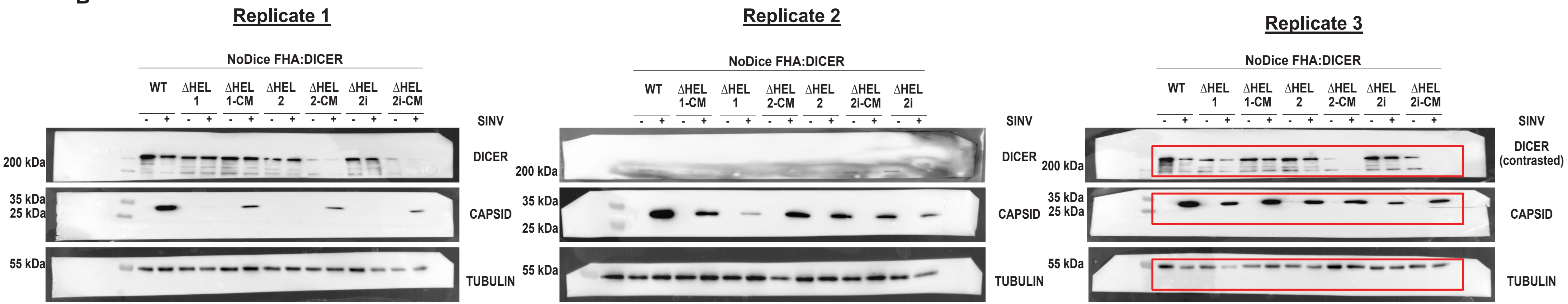
Replicate 1
NoDice FHA:DICER
WT
ΔHEL 1
ΔHEL 1-CM
ΔHEL 2
ΔHEL 2-CM
ΔHEL 2i
ΔHEL 2i-CM
SINV
200 kDa
DICER
35 kDa
25 kDa
CAPSID
55 kDa
TUBULIN
Replicate 2
NoDice FHA:DICER
WT
ΔHEL 1-CM
ΔHEL 1
ΔHEL 2-CM
ΔHEL 2
ΔHEL 2i-CM
ΔHEL 2i
SINV
DICER
200 kDa
35 kDa
25 kDa
CAPSID
55 kDa
TUBULIN
Replicate 3
NoDice FHA:DICER
WT
ΔHEL 1
ΔHEL 1-CM
ΔHEL 2
ΔHEL 2-CM
ΔHEL 2i
ΔHEL 2i-CM
SINV
200 kDa
DICER (contrasted)
35 kDa
25 kDa
CAPSID
55 kDa
TUBULIN

D

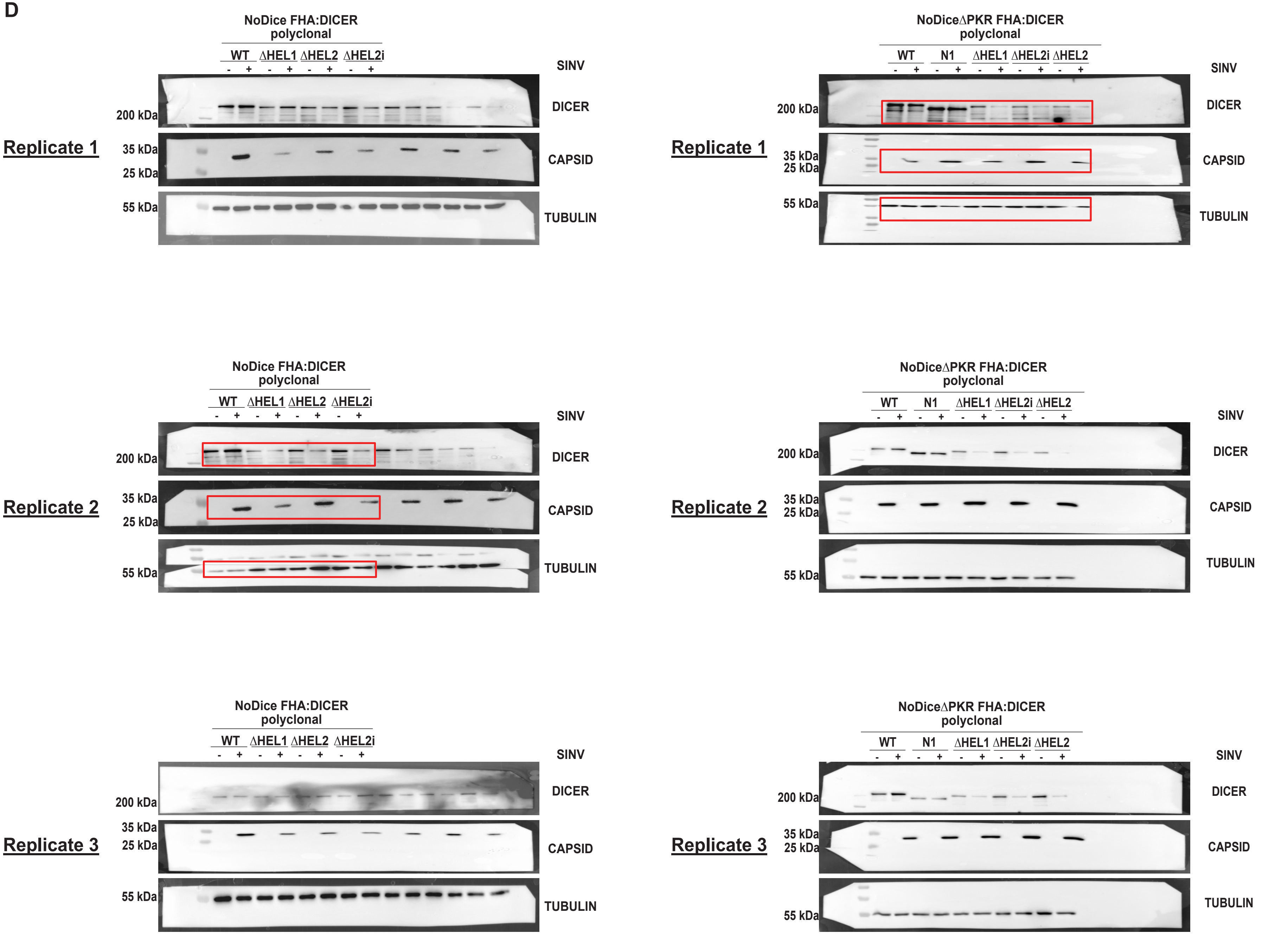

NoDice FHA:DICER
polyclonal
WT ΔHEL1 ΔHEL2 ΔHEL2i
- + - + - + - +
SINV
DICER
CAPSID
TUBULIN
200 kDa
35 kDa
25 kDa
55 kDa
Replicate 1
NoDiceΔPKR FHA:DICER
polyclonal
WT N1 ΔHEL1 ΔHEL2i ΔHEL2
- + - + - + - + - +
SINV
DICER
CAPSID
TUBULIN
200 kDa
35 kDa
25 kDa
55 kDa
Replicate 1
NoDice FHA:DICER
polyclonal
WT ΔHEL1 ΔHEL2 ΔHEL2i
- + - + - + - +
SINV
DICER
CAPSID
TUBULIN
200 kDa
35 kDa
25 kDa
55 kDa
Replicate 2
NoDiceΔPKR FHA:DICER
polyclonal
WT N1 ΔHEL1 ΔHEL2i ΔHEL2
- + - + - + - + - +
SINV
DICER
CAPSID
TUBULIN
200 kDa
35 kDa
25 kDa
55 kDa
Replicate 2
NoDice FHA:DICER
polyclonal
WT ΔHEL1 ΔHEL2 ΔHEL2i
- + - + - + - +
SINV
DICER
CAPSID
TUBULIN
200 kDa
35 kDa
25 kDa
55 kDa
Replicate 3
NoDiceΔPKR FHA:DICER
polyclonal
WT N1 ΔHEL1 ΔHEL2i ΔHEL2
- + - + - + - + - +
SINV
DICER
CAPSID
TUBULIN
200 kDa
35 kDa
25 kDa
55 kDa
Replicate 3
