# Supplementary material for: The helicase domain of human Dicer prevents RNAi-independent activation of antiviral and inflammatory pathways
Source: EMBO J. 2024 Jan 29;43(5):7. doi: 10.1038/s44318-024-00035-2 (PMC10907635; doi:10.1038/s44318-024-00035-2)
Supplement: Supplementary file 6 — Source Data Fig. 5 [file 44318_2024_35_MOESM6_ESM.zip › EMBOJ-2023-115792R2_SourceData_Fig5/Figure5.pdf]

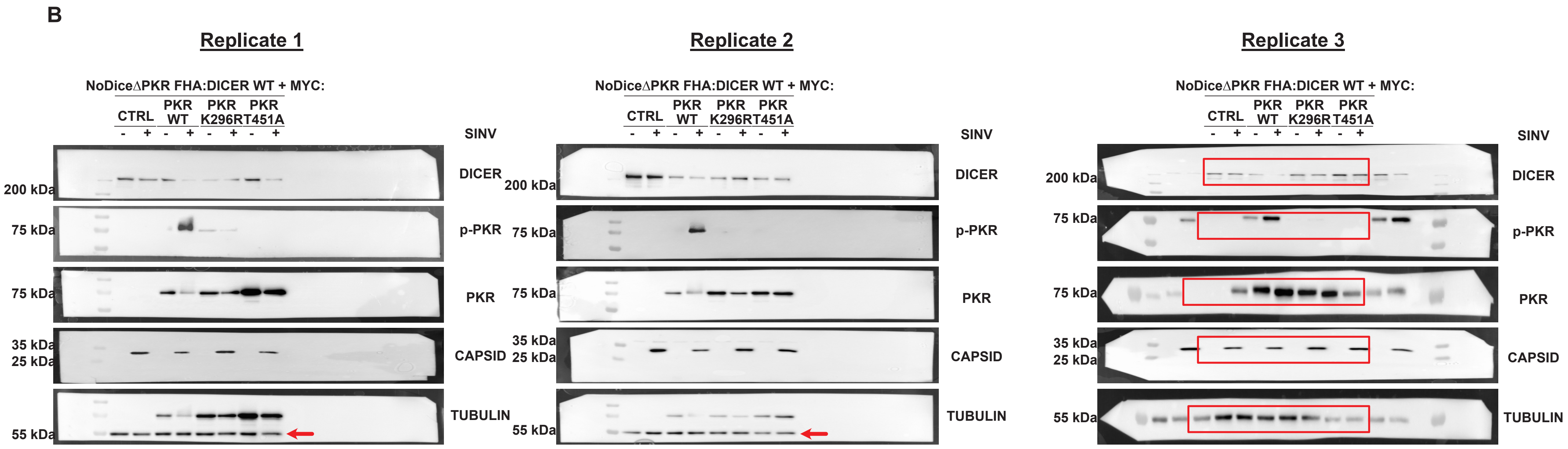
B
Replicate 1
Replicate 2
Replicate 3
NoDiceΔPKR FHA:DICER WT + MYC:
CTRL
PKR WT
PKR K296R
PKR T451A
SINV
- + - + - + - +
200 kDa
75 kDa
35 kDa
25 kDa
55 kDa
DICER
p-PKR
PKR
CAPSID
TUBULIN

D

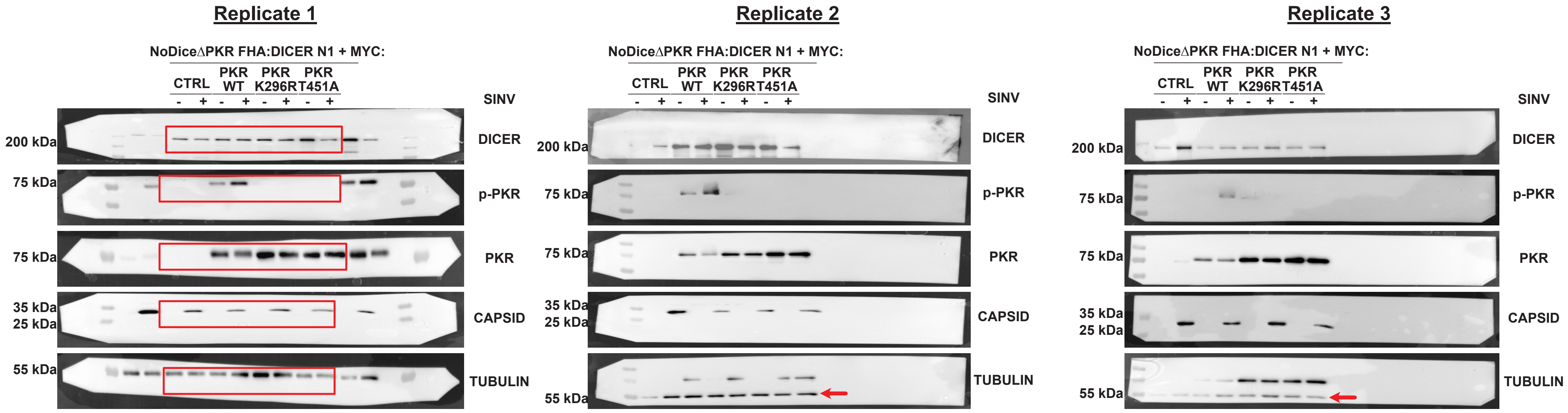
Replicate 1
NoDiceΔPKR FHA:DICER N1 + MYC:
CTRL
PKR WT
PKR K296R
PKR T451A
- + - + - + - +
SINV
200 kDa
DICER
75 kDa
p-PKR
75 kDa
PKR
35 kDa
25 kDa
CAPSID
55 kDa
TUBULIN
Replicate 2
NoDiceΔPKR FHA:DICER N1 + MYC:
CTRL
PKR WT
PKR K296R
PKR T451A
- + - + - + - +
SINV
200 kDa
DICER
75 kDa
p-PKR
75 kDa
PKR
35 kDa
25 kDa
CAPSID
55 kDa
TUBULIN
Replicate 3
NoDiceΔPKR FHA:DICER N1 + MYC:
CTRL
PKR WT
PKR K296R
PKR T451A
- + - + - + - +
SINV
200 kDa
DICER
75 kDa
p-PKR
75 kDa
PKR
35 kDa
25 kDa
CAPSID
55 kDa
TUBULIN

F

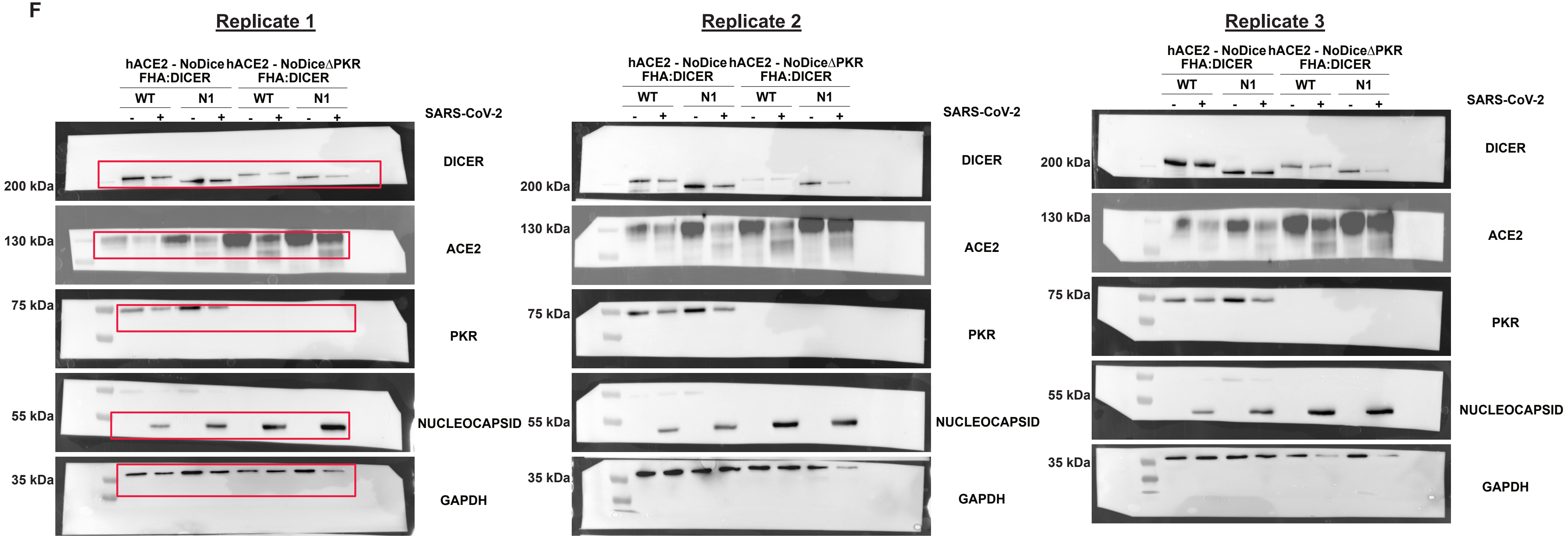
Replicate 1
hACE2 - NoDice FHA:DICER
hACE2 - NoDiceΔPKR FHA:DICER
WT
N1
WT
N1
- + - + - + - +
SARS-CoV-2
200 kDa
DICER
130 kDa
ACE2
75 kDa
PKR
55 kDa
NUCLEOCAPSID
35 kDa
GAPDH
Replicate 2
hACE2 - NoDice FHA:DICER
hACE2 - NoDiceΔPKR FHA:DICER
WT
N1
WT
N1
- + - + - + - +
SARS-CoV-2
200 kDa
DICER
130 kDa
ACE2
75 kDa
PKR
55 kDa
NUCLEOCAPSID
35 kDa
GAPDH
Replicate 3
hACE2 - NoDice FHA:DICER
hACE2 - NoDiceΔPKR FHA:DICER
WT
N1
WT
N1
- + - + - + - +
SARS-CoV-2
200 kDa
DICER
130 kDa
ACE2
75 kDa
PKR
55 kDa
NUCLEOCAPSID
35 kDa
GAPDH
