# Supplementary figures and images for: The helicase domain of human Dicer prevents RNAi-independent activation of antiviral and inflammatory pathways (part 3 of 5)
Source: EMBO J. 2024 Jan 29;43(5):7. doi: 10.1038/s44318-024-00035-2 (PMC10907635; doi:10.1038/s44318-024-00035-2)

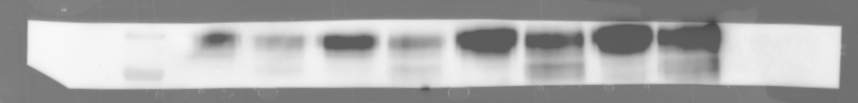

Supplement: Supplementary file 6 — Source Data Fig. 5 [file 44318_2024_35_MOESM6_ESM.zip › EMBOJ-2023-115792R2_SourceData_Fig5/5F western blot/R3/western ace2.tiff]

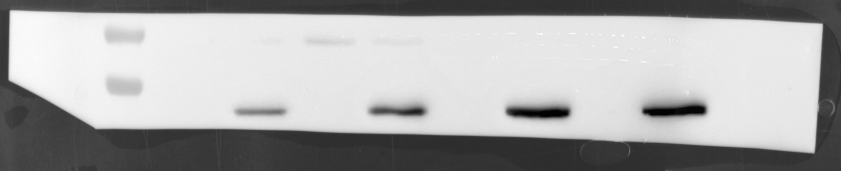

Supplement: Supplementary file 6 — Source Data Fig. 5 [file 44318_2024_35_MOESM6_ESM.zip › EMBOJ-2023-115792R2_SourceData_Fig5/5F western blot/R3/western nucleocapsid.tiff]

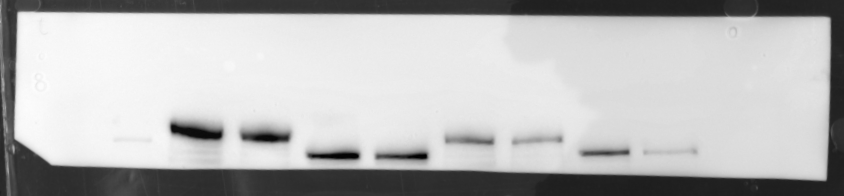

Supplement: Supplementary file 6 — Source Data Fig. 5 [file 44318_2024_35_MOESM6_ESM.zip › EMBOJ-2023-115792R2_SourceData_Fig5/5F western blot/R3/western dicer.tiff]

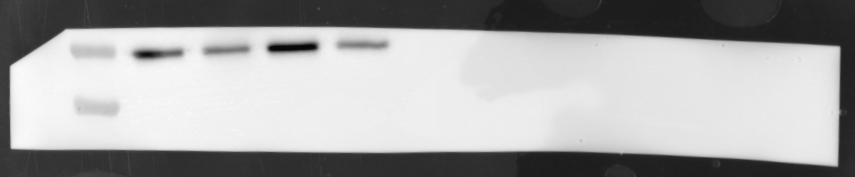

Supplement: Supplementary file 6 — Source Data Fig. 5 [file 44318_2024_35_MOESM6_ESM.zip › EMBOJ-2023-115792R2_SourceData_Fig5/5F western blot/R2/western pkr.tiff]

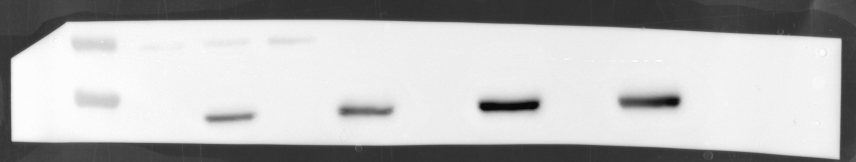

Supplement: Supplementary file 6 — Source Data Fig. 5 [file 44318_2024_35_MOESM6_ESM.zip › EMBOJ-2023-115792R2_SourceData_Fig5/5F western blot/R2/western nucleocaspid.tiff]

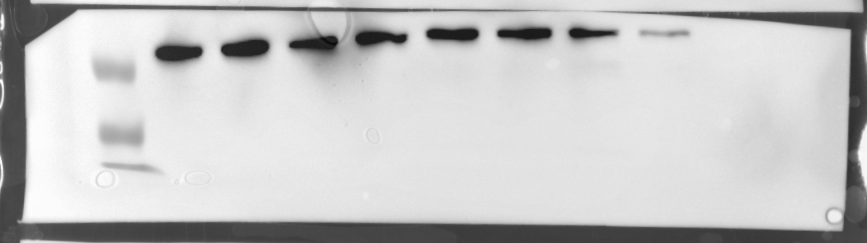

Supplement: Supplementary file 6 — Source Data Fig. 5 [file 44318_2024_35_MOESM6_ESM.zip › EMBOJ-2023-115792R2_SourceData_Fig5/5F western blot/R2/western gapdh.tiff]

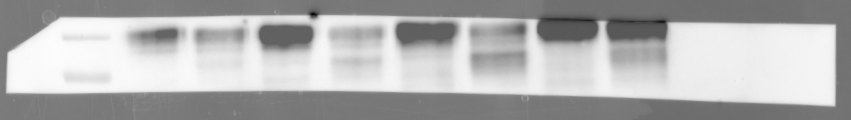

Supplement: Supplementary file 6 — Source Data Fig. 5 [file 44318_2024_35_MOESM6_ESM.zip › EMBOJ-2023-115792R2_SourceData_Fig5/5F western blot/R2/western ace2.tiff]

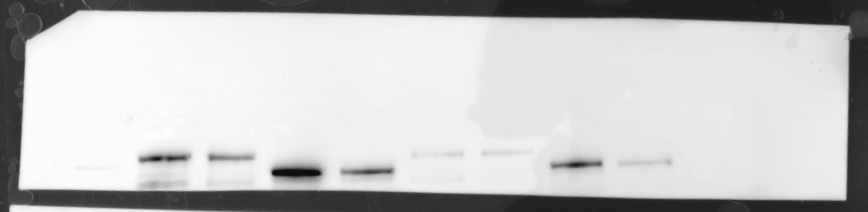

Supplement: Supplementary file 6 — Source Data Fig. 5 [file 44318_2024_35_MOESM6_ESM.zip › EMBOJ-2023-115792R2_SourceData_Fig5/5F western blot/R2/western dicer.tiff]

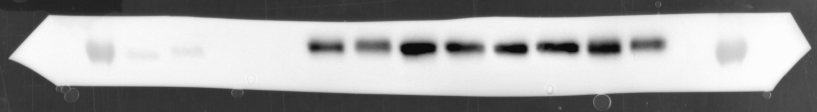

Supplement: Supplementary file 6 — Source Data Fig. 5 [file 44318_2024_35_MOESM6_ESM.zip › EMBOJ-2023-115792R2_SourceData_Fig5/5D western blot/R1/western PKR.tiff]

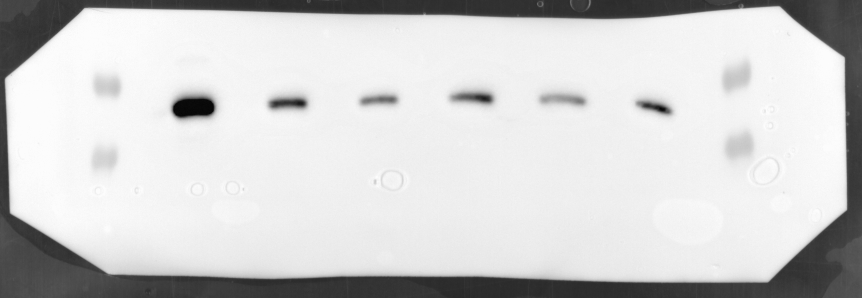

Supplement: Supplementary file 6 — Source Data Fig. 5 [file 44318_2024_35_MOESM6_ESM.zip › EMBOJ-2023-115792R2_SourceData_Fig5/5D western blot/R1/western capsid.tiff]

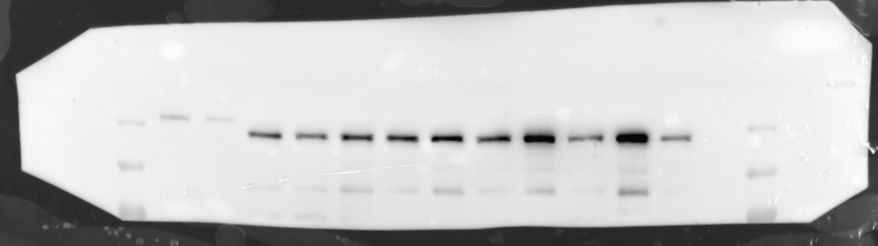

Supplement: Supplementary file 6 — Source Data Fig. 5 [file 44318_2024_35_MOESM6_ESM.zip › EMBOJ-2023-115792R2_SourceData_Fig5/5D western blot/R1/western dicer.tiff]

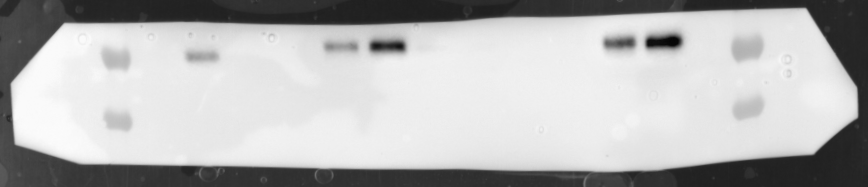

Supplement: Supplementary file 6 — Source Data Fig. 5 [file 44318_2024_35_MOESM6_ESM.zip › EMBOJ-2023-115792R2_SourceData_Fig5/5D western blot/R1/western p-PKR.tiff]

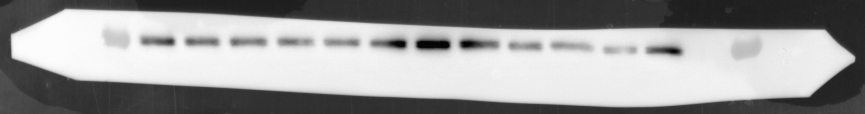

Supplement: Supplementary file 6 — Source Data Fig. 5 [file 44318_2024_35_MOESM6_ESM.zip › EMBOJ-2023-115792R2_SourceData_Fig5/5D western blot/R1/western tubulin.tiff]

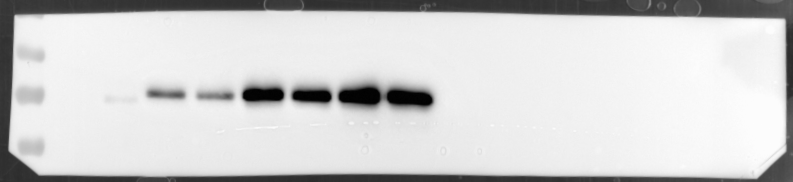

Supplement: Supplementary file 6 — Source Data Fig. 5 [file 44318_2024_35_MOESM6_ESM.zip › EMBOJ-2023-115792R2_SourceData_Fig5/5D western blot/R3/western PKR.tiff]

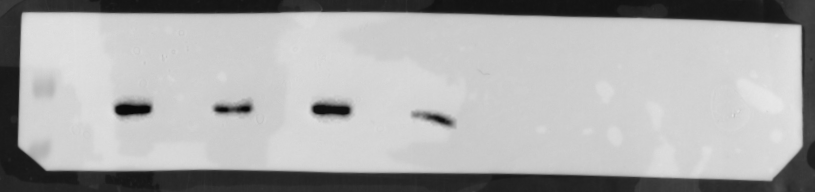

Supplement: Supplementary file 6 — Source Data Fig. 5 [file 44318_2024_35_MOESM6_ESM.zip › EMBOJ-2023-115792R2_SourceData_Fig5/5D western blot/R3/western capsid.tiff]

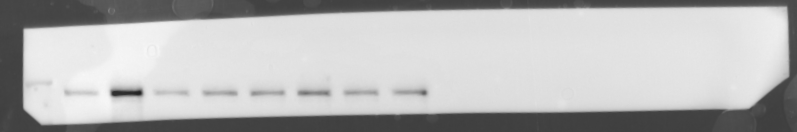

Supplement: Supplementary file 6 — Source Data Fig. 5 [file 44318_2024_35_MOESM6_ESM.zip › EMBOJ-2023-115792R2_SourceData_Fig5/5D western blot/R3/western dicer.tiff]

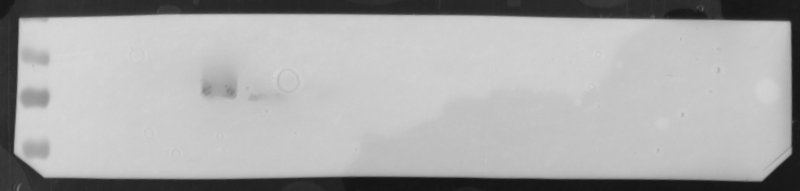

Supplement: Supplementary file 6 — Source Data Fig. 5 [file 44318_2024_35_MOESM6_ESM.zip › EMBOJ-2023-115792R2_SourceData_Fig5/5D western blot/R3/western p-PKR.tiff]

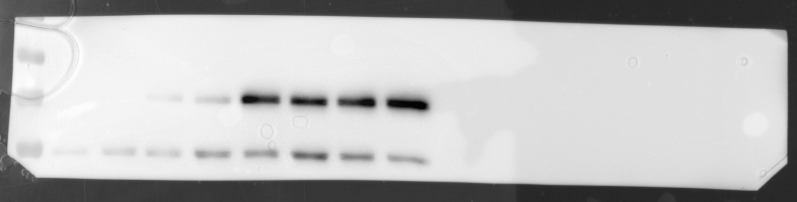

Supplement: Supplementary file 6 — Source Data Fig. 5 [file 44318_2024_35_MOESM6_ESM.zip › EMBOJ-2023-115792R2_SourceData_Fig5/5D western blot/R3/western tubulin.tiff]

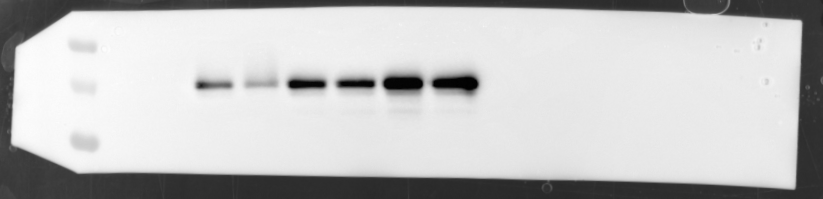

Supplement: Supplementary file 6 — Source Data Fig. 5 [file 44318_2024_35_MOESM6_ESM.zip › EMBOJ-2023-115792R2_SourceData_Fig5/5D western blot/R2/western PKR.tiff]

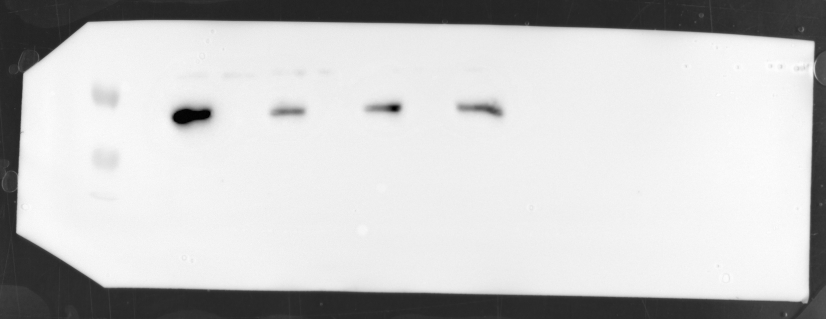

Supplement: Supplementary file 6 — Source Data Fig. 5 [file 44318_2024_35_MOESM6_ESM.zip › EMBOJ-2023-115792R2_SourceData_Fig5/5D western blot/R2/western capsid.tiff]

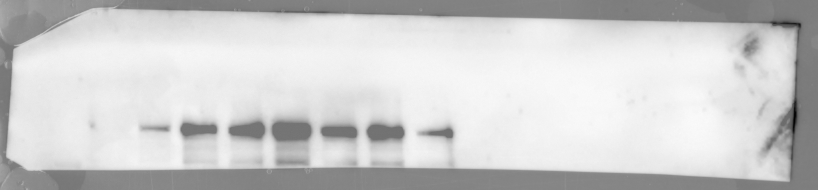

Supplement: Supplementary file 6 — Source Data Fig. 5 [file 44318_2024_35_MOESM6_ESM.zip › EMBOJ-2023-115792R2_SourceData_Fig5/5D western blot/R2/western dicer.tiff]

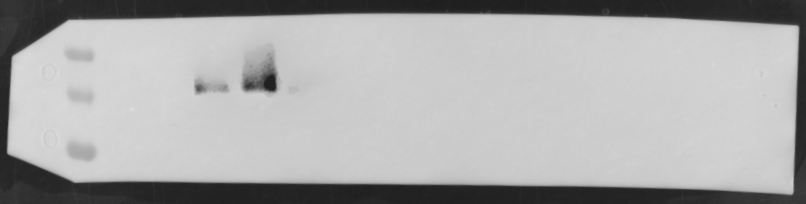

Supplement: Supplementary file 6 — Source Data Fig. 5 [file 44318_2024_35_MOESM6_ESM.zip › EMBOJ-2023-115792R2_SourceData_Fig5/5D western blot/R2/western p-PKR.tiff]

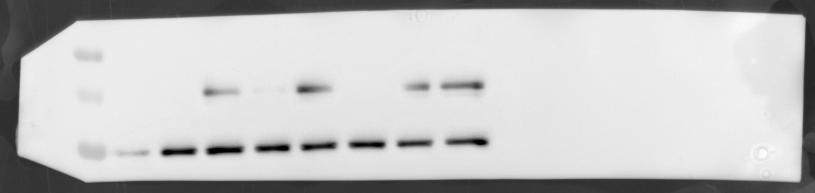

Supplement: Supplementary file 6 — Source Data Fig. 5 [file 44318_2024_35_MOESM6_ESM.zip › EMBOJ-2023-115792R2_SourceData_Fig5/5D western blot/R2/western tubulin.tiff]

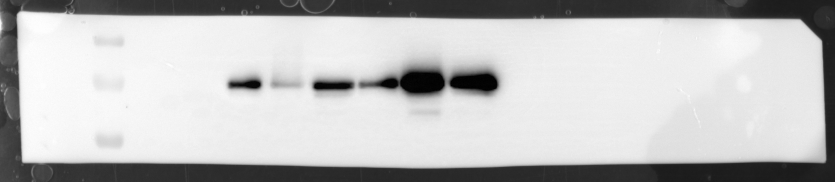

Supplement: Supplementary file 6 — Source Data Fig. 5 [file 44318_2024_35_MOESM6_ESM.zip › EMBOJ-2023-115792R2_SourceData_Fig5/5B western blot/R1/western PKR.tiff]

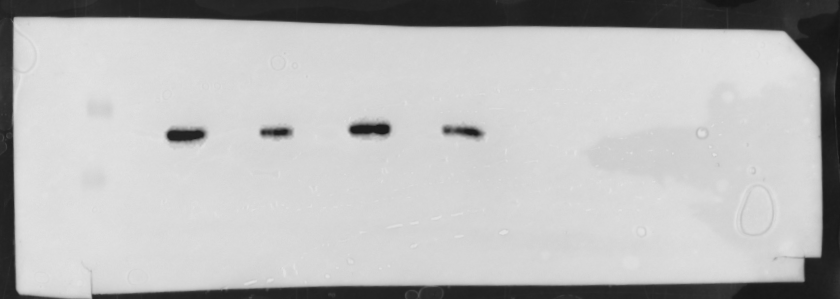

Supplement: Supplementary file 6 — Source Data Fig. 5 [file 44318_2024_35_MOESM6_ESM.zip › EMBOJ-2023-115792R2_SourceData_Fig5/5B western blot/R1/western capsid.tiff]

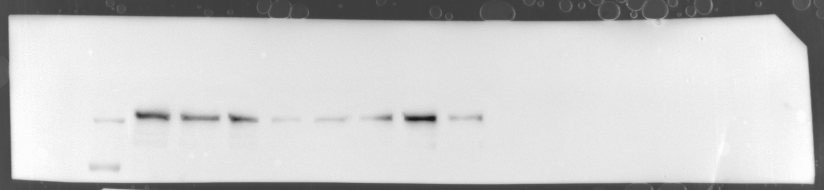

Supplement: Supplementary file 6 — Source Data Fig. 5 [file 44318_2024_35_MOESM6_ESM.zip › EMBOJ-2023-115792R2_SourceData_Fig5/5B western blot/R1/western dicer.tiff]

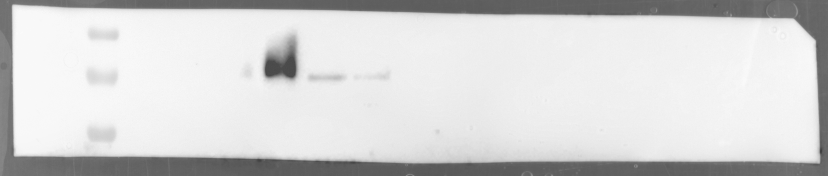

Supplement: Supplementary file 6 — Source Data Fig. 5 [file 44318_2024_35_MOESM6_ESM.zip › EMBOJ-2023-115792R2_SourceData_Fig5/5B western blot/R1/western p-PKR.tiff]

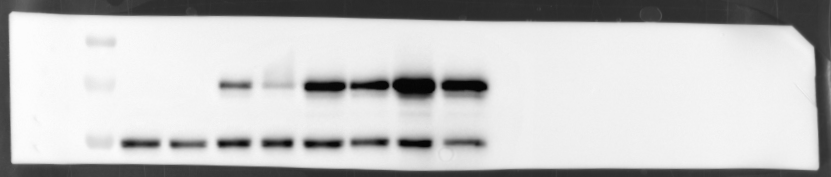

Supplement: Supplementary file 6 — Source Data Fig. 5 [file 44318_2024_35_MOESM6_ESM.zip › EMBOJ-2023-115792R2_SourceData_Fig5/5B western blot/R1/western tubulin.tiff]

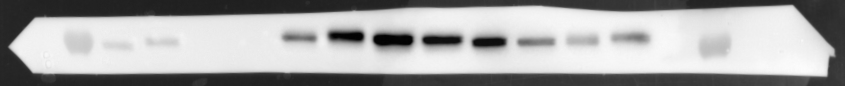

Supplement: Supplementary file 6 — Source Data Fig. 5 [file 44318_2024_35_MOESM6_ESM.zip › EMBOJ-2023-115792R2_SourceData_Fig5/5B western blot/R3/western PKR.tiff]

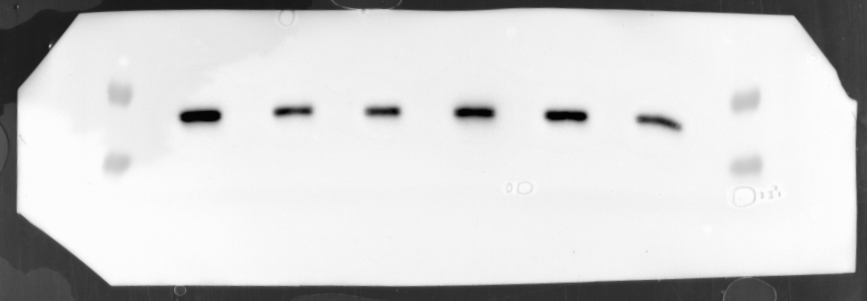

Supplement: Supplementary file 6 — Source Data Fig. 5 [file 44318_2024_35_MOESM6_ESM.zip › EMBOJ-2023-115792R2_SourceData_Fig5/5B western blot/R3/western capsid.tiff]

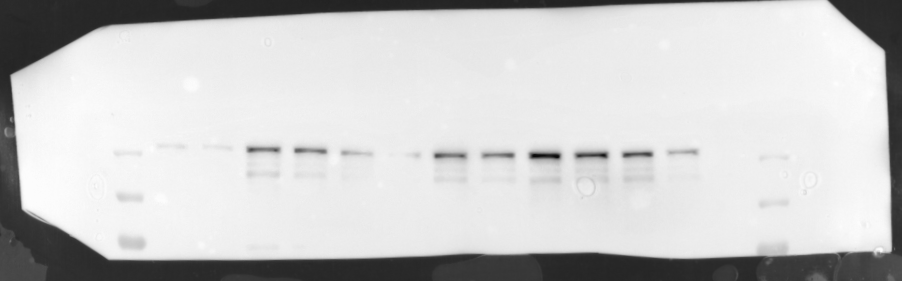

Supplement: Supplementary file 6 — Source Data Fig. 5 [file 44318_2024_35_MOESM6_ESM.zip › EMBOJ-2023-115792R2_SourceData_Fig5/5B western blot/R3/western dicer.tiff]

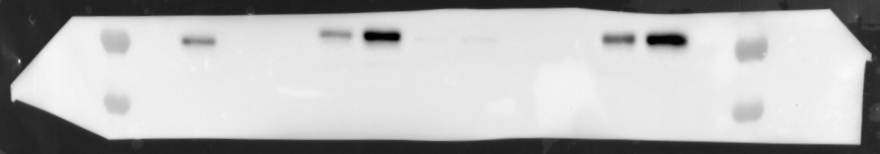

Supplement: Supplementary file 6 — Source Data Fig. 5 [file 44318_2024_35_MOESM6_ESM.zip › EMBOJ-2023-115792R2_SourceData_Fig5/5B western blot/R3/western p-PKR.tiff]

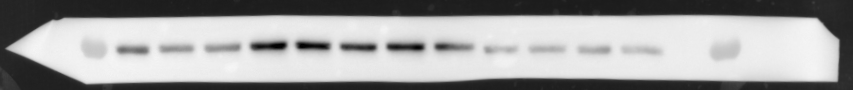

Supplement: Supplementary file 6 — Source Data Fig. 5 [file 44318_2024_35_MOESM6_ESM.zip › EMBOJ-2023-115792R2_SourceData_Fig5/5B western blot/R3/western tubulin.tiff]

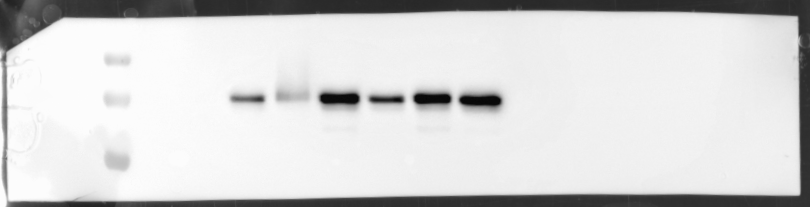

Supplement: Supplementary file 6 — Source Data Fig. 5 [file 44318_2024_35_MOESM6_ESM.zip › EMBOJ-2023-115792R2_SourceData_Fig5/5B western blot/R2/western PKR.tiff]

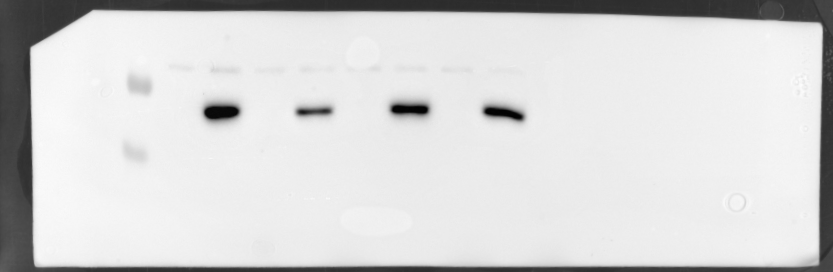

Supplement: Supplementary file 6 — Source Data Fig. 5 [file 44318_2024_35_MOESM6_ESM.zip › EMBOJ-2023-115792R2_SourceData_Fig5/5B western blot/R2/western capsid.tiff]

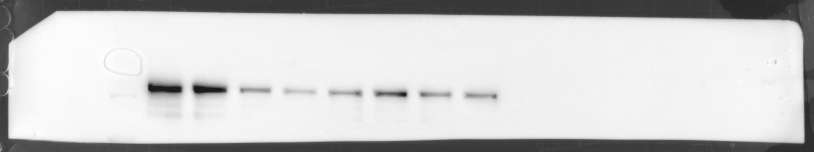

Supplement: Supplementary file 6 — Source Data Fig. 5 [file 44318_2024_35_MOESM6_ESM.zip › EMBOJ-2023-115792R2_SourceData_Fig5/5B western blot/R2/western dicer.tiff]

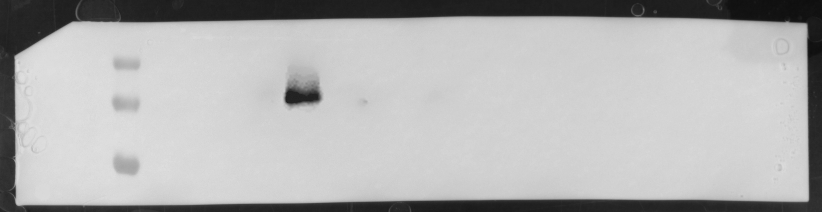

Supplement: Supplementary file 6 — Source Data Fig. 5 [file 44318_2024_35_MOESM6_ESM.zip › EMBOJ-2023-115792R2_SourceData_Fig5/5B western blot/R2/western p-PKR.tiff]

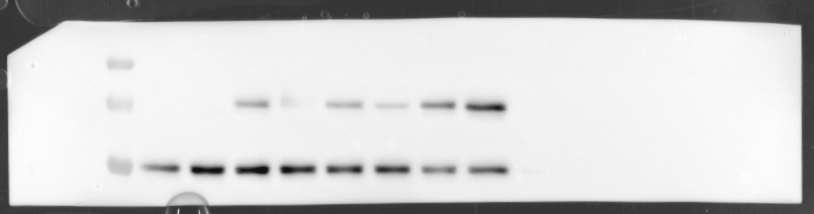

Supplement: Supplementary file 6 — Source Data Fig. 5 [file 44318_2024_35_MOESM6_ESM.zip › EMBOJ-2023-115792R2_SourceData_Fig5/5B western blot/R2/western tubulin.tiff]

A

Replicate 1

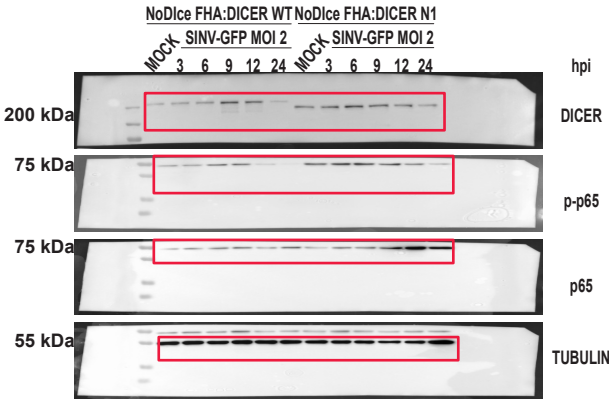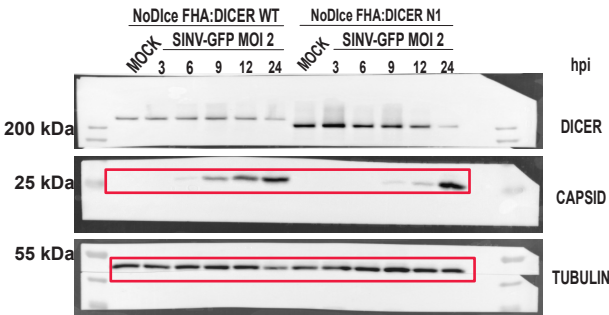

Replicate 2

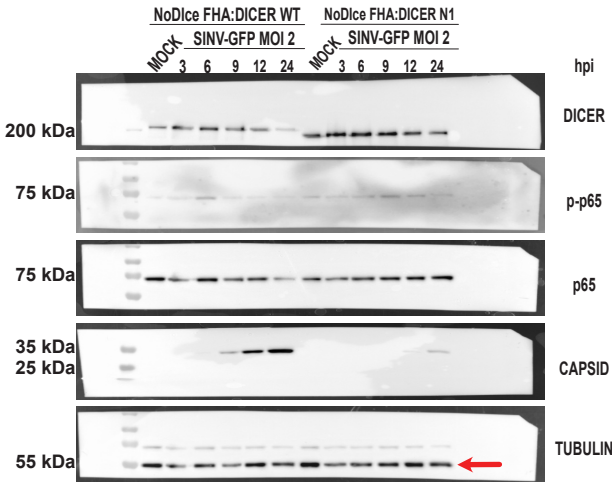

Replicate 3

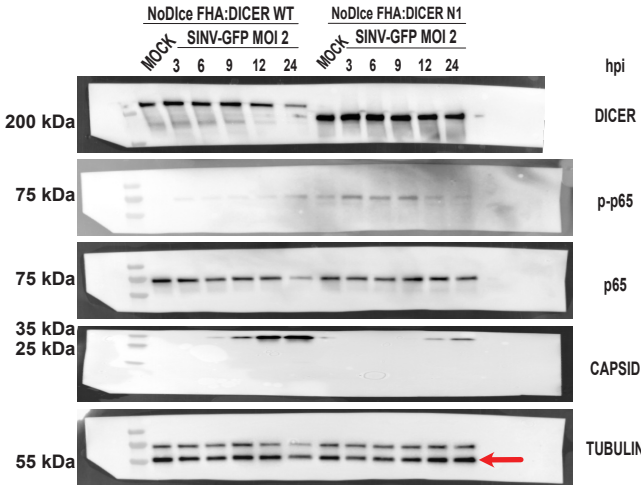

B

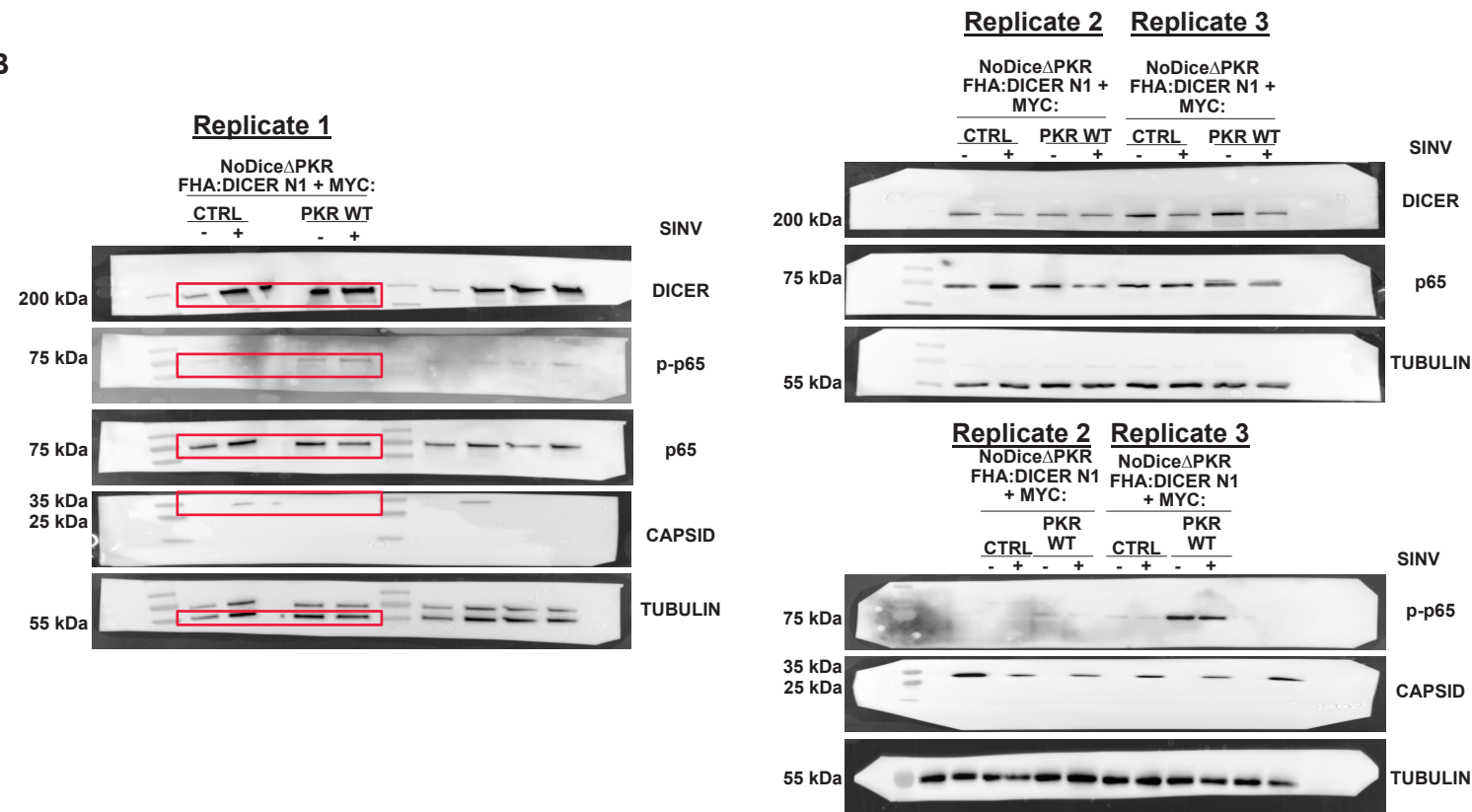

### Replicate 1

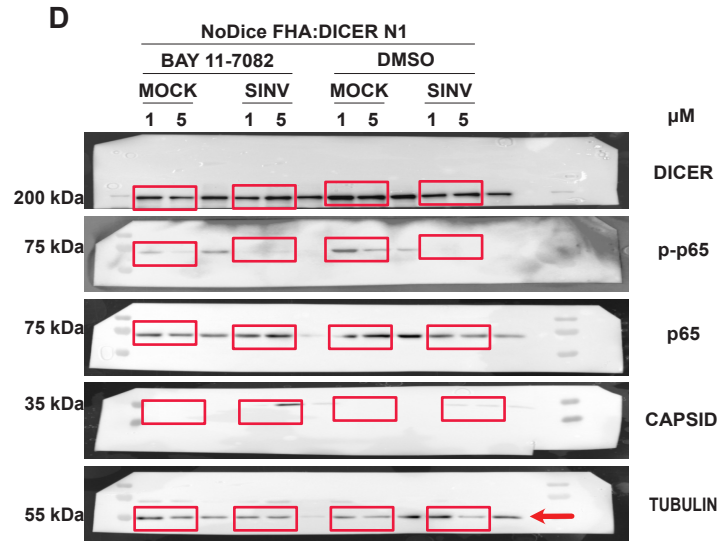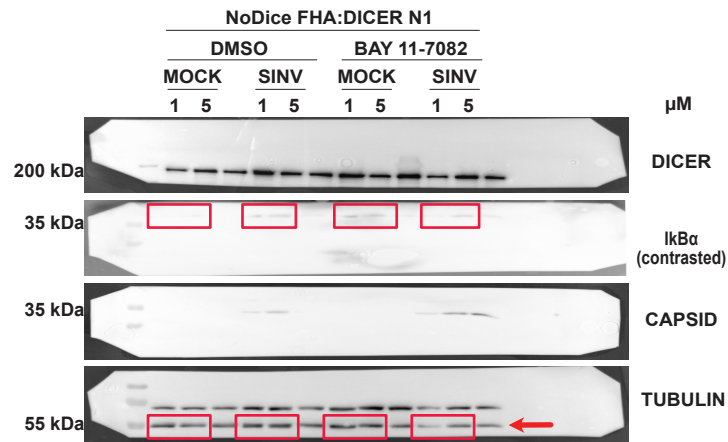

### Replicate 2

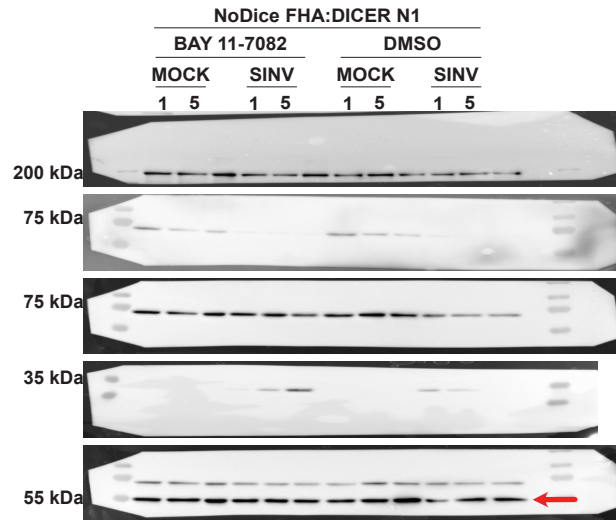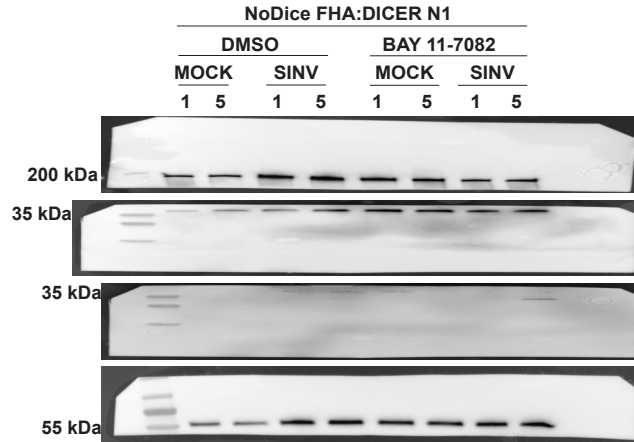

### Replicate 3

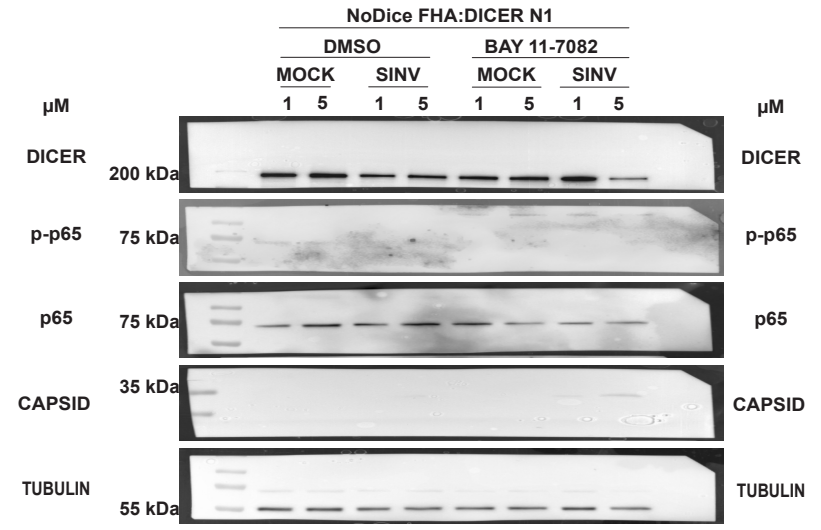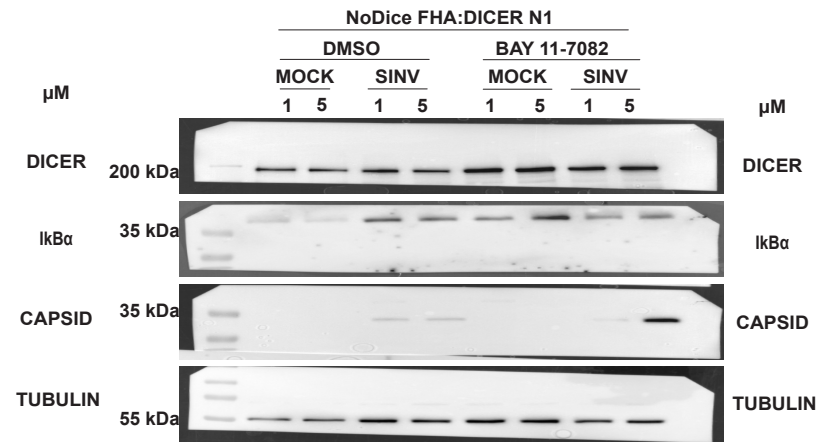

Supplement: Supplementary file 9 — Source Data Fig. 8 [file 44318_2024_35_MOESM9_ESM.zip › EMBOJ-2023-115792R2_SourceData_Fig8/Figure8.pdf]

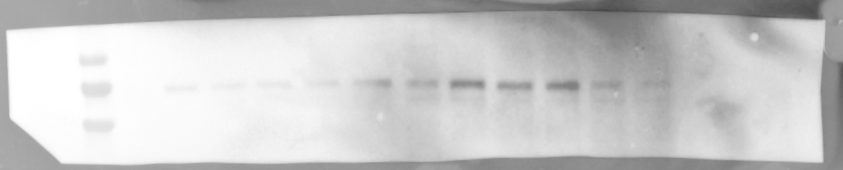

Supplement: Supplementary file 9 — Source Data Fig. 8 [file 44318_2024_35_MOESM9_ESM.zip › EMBOJ-2023-115792R2_SourceData_Fig8/Fig_8A western blot/R3/western p-p65.tiff]

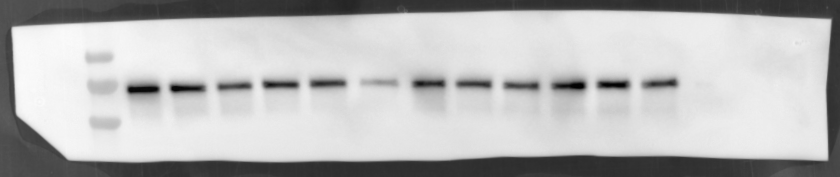

Supplement: Supplementary file 9 — Source Data Fig. 8 [file 44318_2024_35_MOESM9_ESM.zip › EMBOJ-2023-115792R2_SourceData_Fig8/Fig_8A western blot/R3/western p65.tiff]

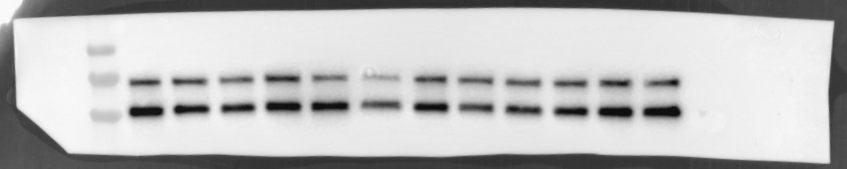

Supplement: Supplementary file 9 — Source Data Fig. 8 [file 44318_2024_35_MOESM9_ESM.zip › EMBOJ-2023-115792R2_SourceData_Fig8/Fig_8A western blot/R3/western tubulin.tiff]

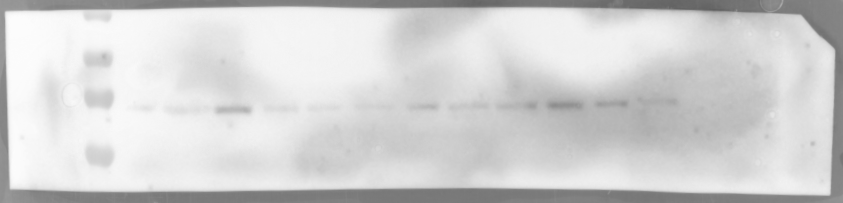

Supplement: Supplementary file 9 — Source Data Fig. 8 [file 44318_2024_35_MOESM9_ESM.zip › EMBOJ-2023-115792R2_SourceData_Fig8/Fig_8A western blot/R2/western p-p65.tiff]

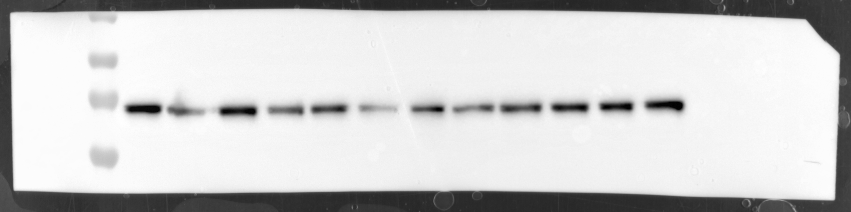

Supplement: Supplementary file 9 — Source Data Fig. 8 [file 44318_2024_35_MOESM9_ESM.zip › EMBOJ-2023-115792R2_SourceData_Fig8/Fig_8A western blot/R2/western p65.tiff]

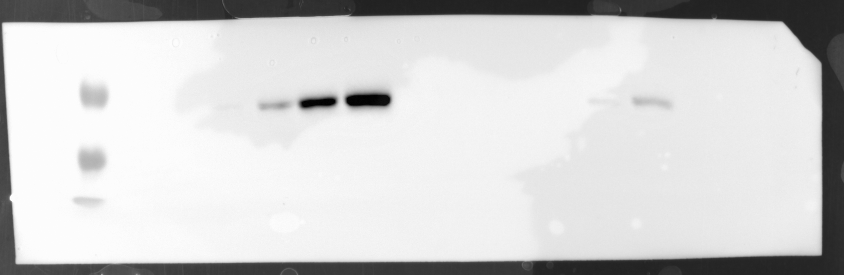

Supplement: Supplementary file 9 — Source Data Fig. 8 [file 44318_2024_35_MOESM9_ESM.zip › EMBOJ-2023-115792R2_SourceData_Fig8/Fig_8A western blot/R2/western capsid.tiff]

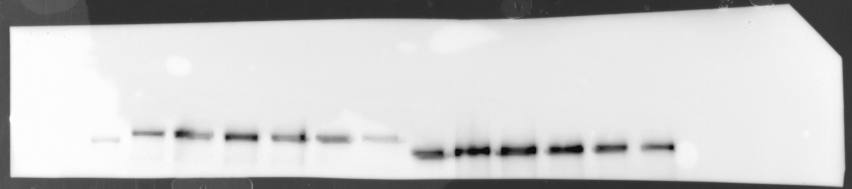

Supplement: Supplementary file 9 — Source Data Fig. 8 [file 44318_2024_35_MOESM9_ESM.zip › EMBOJ-2023-115792R2_SourceData_Fig8/Fig_8A western blot/R2/western dicer.tiff]

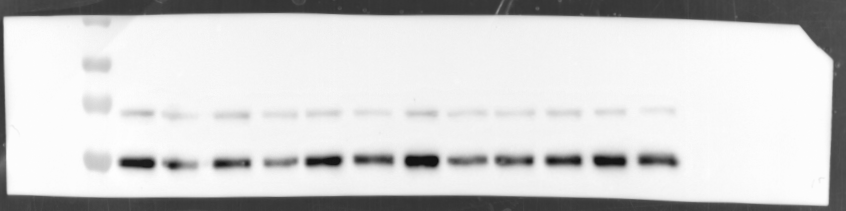

Supplement: Supplementary file 9 — Source Data Fig. 8 [file 44318_2024_35_MOESM9_ESM.zip › EMBOJ-2023-115792R2_SourceData_Fig8/Fig_8A western blot/R2/western tubulin.tiff]

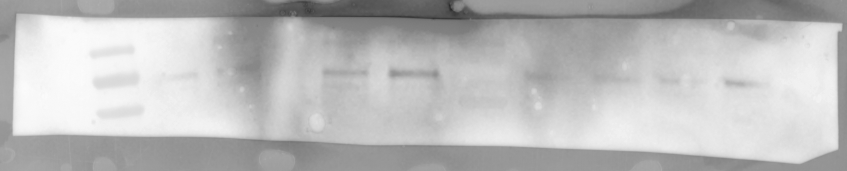

Supplement: Supplementary file 9 — Source Data Fig. 8 [file 44318_2024_35_MOESM9_ESM.zip › EMBOJ-2023-115792R2_SourceData_Fig8/Fig_8B western blot/R1/western p-p65.tiff]

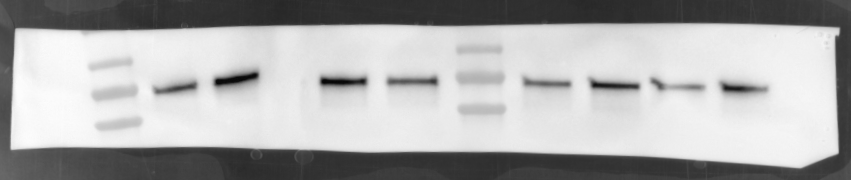

Supplement: Supplementary file 9 — Source Data Fig. 8 [file 44318_2024_35_MOESM9_ESM.zip › EMBOJ-2023-115792R2_SourceData_Fig8/Fig_8B western blot/R1/western p65.tiff]

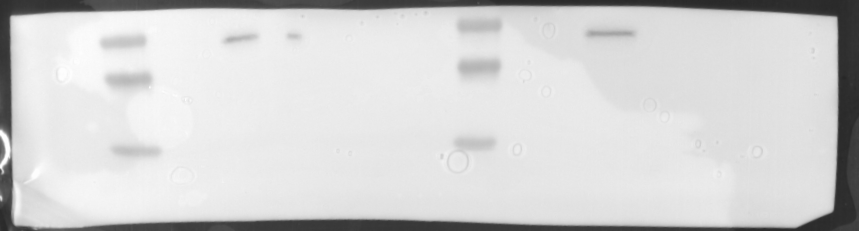

Supplement: Supplementary file 9 — Source Data Fig. 8 [file 44318_2024_35_MOESM9_ESM.zip › EMBOJ-2023-115792R2_SourceData_Fig8/Fig_8B western blot/R1/western capsid.tiff]

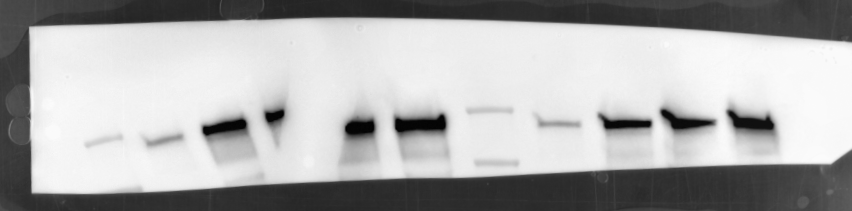

Supplement: Supplementary file 9 — Source Data Fig. 8 [file 44318_2024_35_MOESM9_ESM.zip › EMBOJ-2023-115792R2_SourceData_Fig8/Fig_8B western blot/R1/western dicer.tiff]

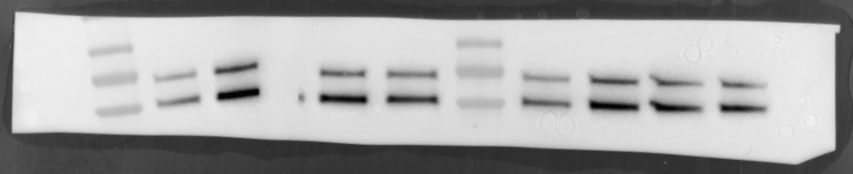

Supplement: Supplementary file 9 — Source Data Fig. 8 [file 44318_2024_35_MOESM9_ESM.zip › EMBOJ-2023-115792R2_SourceData_Fig8/Fig_8B western blot/R1/western tubulin.tiff]

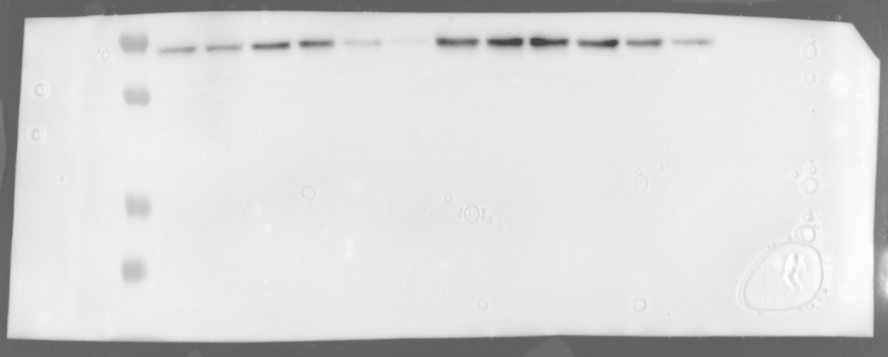

Supplement: Supplementary file 9 — Source Data Fig. 8 [file 44318_2024_35_MOESM9_ESM.zip › EMBOJ-2023-115792R2_SourceData_Fig8/Fig_8A western blot/R1/p65/western p-p65.tiff]

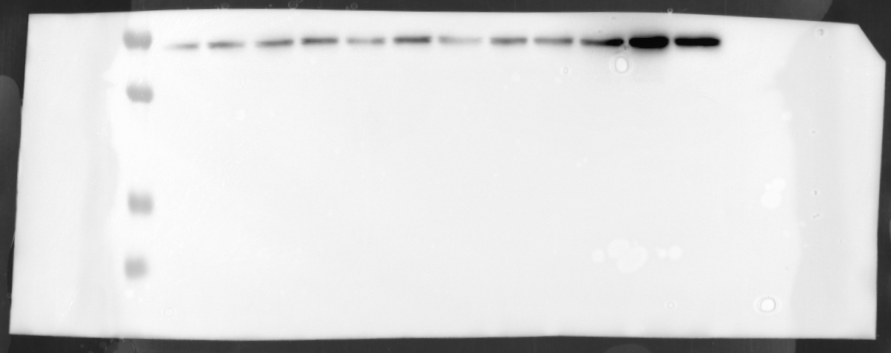

Supplement: Supplementary file 9 — Source Data Fig. 8 [file 44318_2024_35_MOESM9_ESM.zip › EMBOJ-2023-115792R2_SourceData_Fig8/Fig_8A western blot/R1/p65/western p65.tiff]

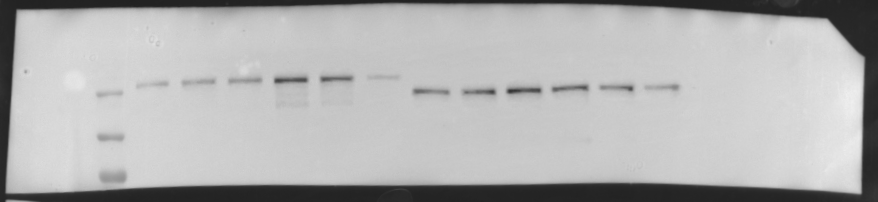

Supplement: Supplementary file 9 — Source Data Fig. 8 [file 44318_2024_35_MOESM9_ESM.zip › EMBOJ-2023-115792R2_SourceData_Fig8/Fig_8A western blot/R1/p65/western dicer.tiff]

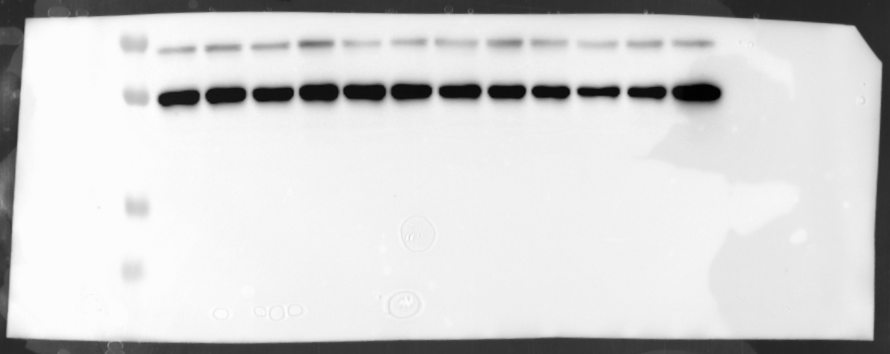

Supplement: Supplementary file 9 — Source Data Fig. 8 [file 44318_2024_35_MOESM9_ESM.zip › EMBOJ-2023-115792R2_SourceData_Fig8/Fig_8A western blot/R1/p65/western tubulin.tiff]

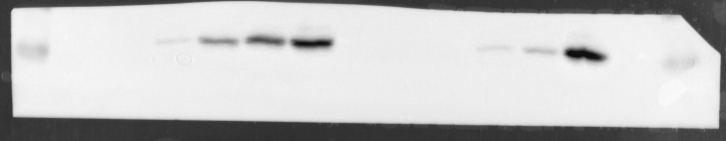

Supplement: Supplementary file 9 — Source Data Fig. 8 [file 44318_2024_35_MOESM9_ESM.zip › EMBOJ-2023-115792R2_SourceData_Fig8/Fig_8A western blot/R1/Capsid/western capsid.tiff]

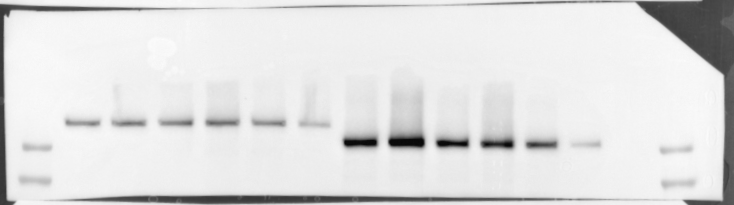

Supplement: Supplementary file 9 — Source Data Fig. 8 [file 44318_2024_35_MOESM9_ESM.zip › EMBOJ-2023-115792R2_SourceData_Fig8/Fig_8A western blot/R1/Capsid/western dicer.tiff]

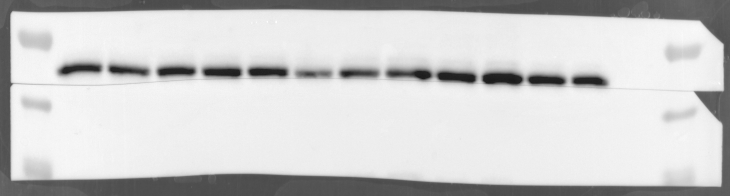

Supplement: Supplementary file 9 — Source Data Fig. 8 [file 44318_2024_35_MOESM9_ESM.zip › EMBOJ-2023-115792R2_SourceData_Fig8/Fig_8A western blot/R1/Capsid/western tubulin.tiff]

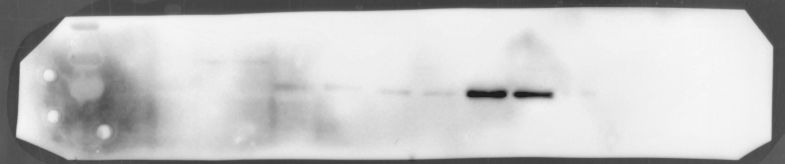

Supplement: Supplementary file 9 — Source Data Fig. 8 [file 44318_2024_35_MOESM9_ESM.zip › EMBOJ-2023-115792R2_SourceData_Fig8/Fig_8B western blot/R3/p-p65/western p-p65.tiff]

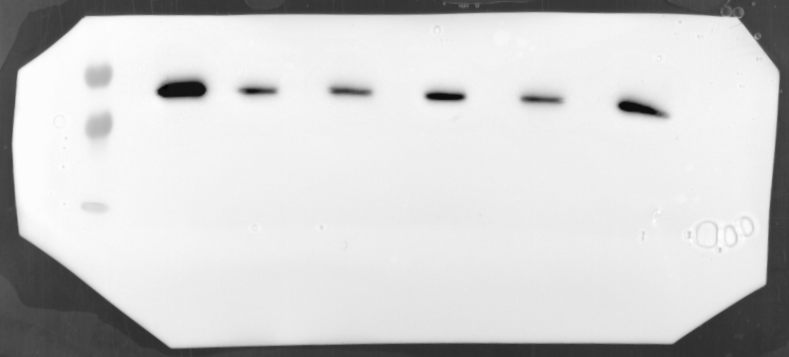

Supplement: Supplementary file 9 — Source Data Fig. 8 [file 44318_2024_35_MOESM9_ESM.zip › EMBOJ-2023-115792R2_SourceData_Fig8/Fig_8B western blot/R3/p-p65/western capsid.tiff]

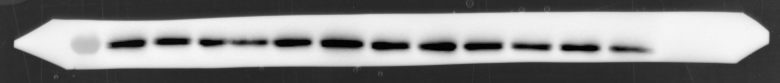

Supplement: Supplementary file 9 — Source Data Fig. 8 [file 44318_2024_35_MOESM9_ESM.zip › EMBOJ-2023-115792R2_SourceData_Fig8/Fig_8B western blot/R3/p-p65/western tubulin.tiff]

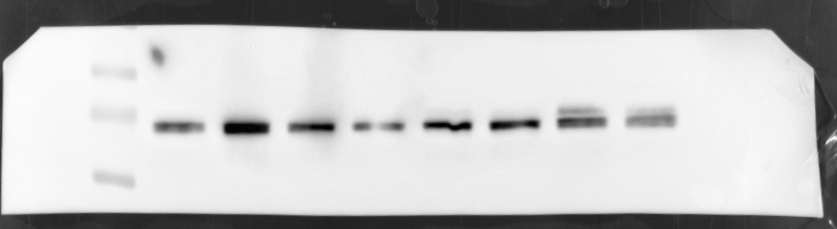

Supplement: Supplementary file 9 — Source Data Fig. 8 [file 44318_2024_35_MOESM9_ESM.zip › EMBOJ-2023-115792R2_SourceData_Fig8/Fig_8B western blot/R3/p65/western p65.tiff]

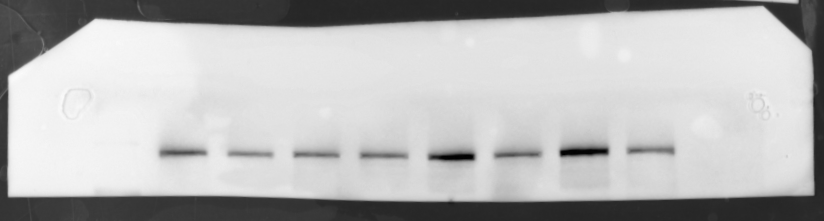

Supplement: Supplementary file 9 — Source Data Fig. 8 [file 44318_2024_35_MOESM9_ESM.zip › EMBOJ-2023-115792R2_SourceData_Fig8/Fig_8B western blot/R3/p65/western dicer.tiff]

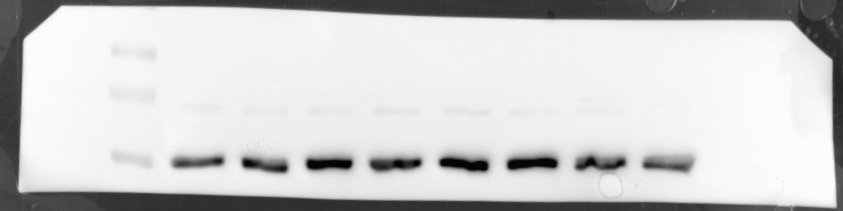

Supplement: Supplementary file 9 — Source Data Fig. 8 [file 44318_2024_35_MOESM9_ESM.zip › EMBOJ-2023-115792R2_SourceData_Fig8/Fig_8B western blot/R3/p65/western tubulin.tiff]

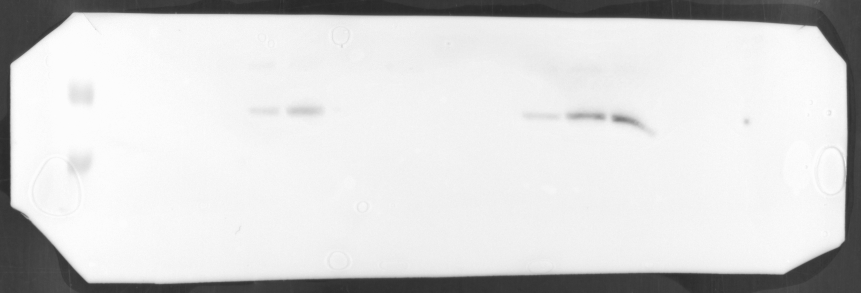

Supplement: Supplementary file 9 — Source Data Fig. 8 [file 44318_2024_35_MOESM9_ESM.zip › EMBOJ-2023-115792R2_SourceData_Fig8/Fig_8D western blot/R1/ikba/western capsid.tiff]

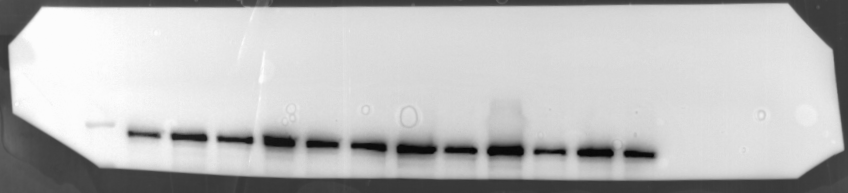

Supplement: Supplementary file 9 — Source Data Fig. 8 [file 44318_2024_35_MOESM9_ESM.zip › EMBOJ-2023-115792R2_SourceData_Fig8/Fig_8D western blot/R1/ikba/western dicer.tiff]

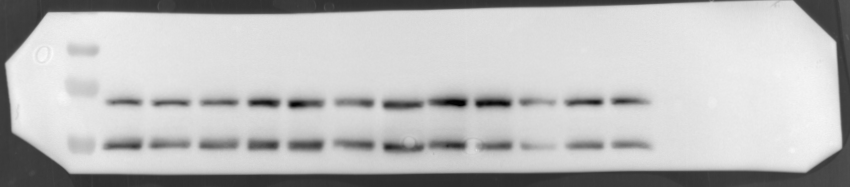

Supplement: Supplementary file 9 — Source Data Fig. 8 [file 44318_2024_35_MOESM9_ESM.zip › EMBOJ-2023-115792R2_SourceData_Fig8/Fig_8D western blot/R1/ikba/western tubulin.tiff]

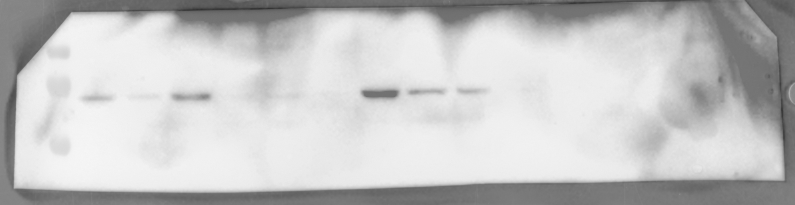

Supplement: Supplementary file 9 — Source Data Fig. 8 [file 44318_2024_35_MOESM9_ESM.zip › EMBOJ-2023-115792R2_SourceData_Fig8/Fig_8D western blot/R1/p65/western p-p65.tiff]

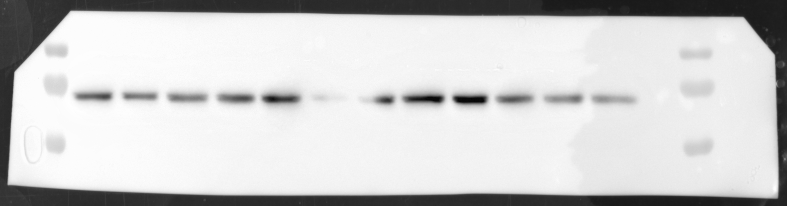

Supplement: Supplementary file 9 — Source Data Fig. 8 [file 44318_2024_35_MOESM9_ESM.zip › EMBOJ-2023-115792R2_SourceData_Fig8/Fig_8D western blot/R1/p65/western p65.tiff]

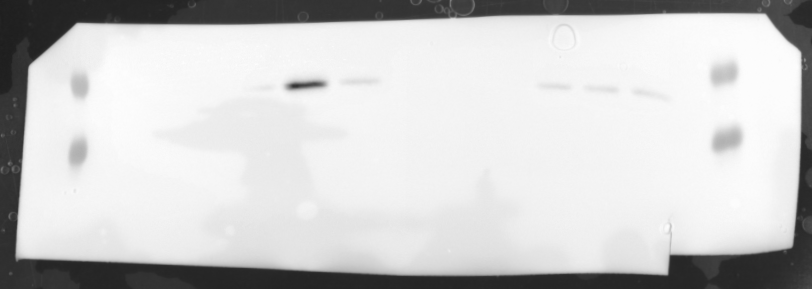

Supplement: Supplementary file 9 — Source Data Fig. 8 [file 44318_2024_35_MOESM9_ESM.zip › EMBOJ-2023-115792R2_SourceData_Fig8/Fig_8D western blot/R1/p65/western capsid.tiff]

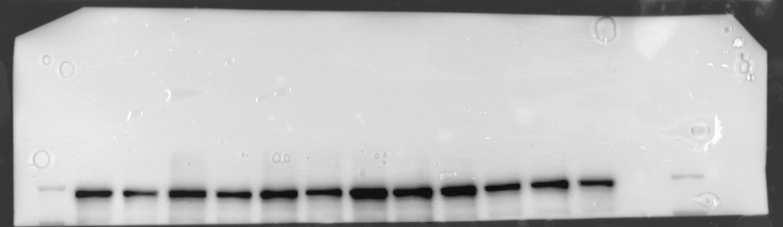

Supplement: Supplementary file 9 — Source Data Fig. 8 [file 44318_2024_35_MOESM9_ESM.zip › EMBOJ-2023-115792R2_SourceData_Fig8/Fig_8D western blot/R1/p65/western dicer.tiff]

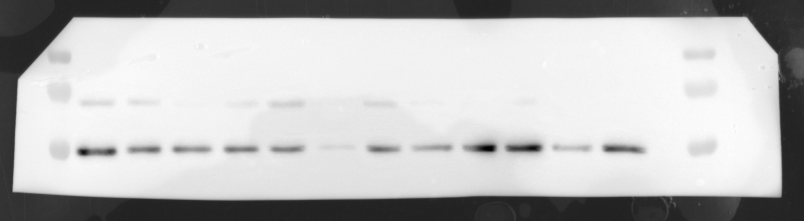

Supplement: Supplementary file 9 — Source Data Fig. 8 [file 44318_2024_35_MOESM9_ESM.zip › EMBOJ-2023-115792R2_SourceData_Fig8/Fig_8D western blot/R1/p65/western tubulin.tiff]

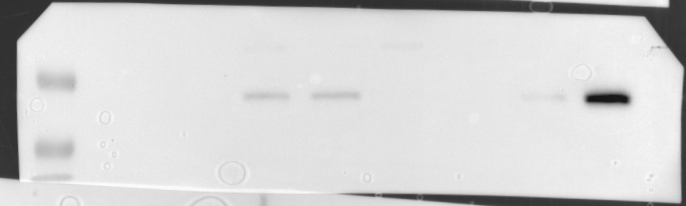

Supplement: Supplementary file 9 — Source Data Fig. 8 [file 44318_2024_35_MOESM9_ESM.zip › EMBOJ-2023-115792R2_SourceData_Fig8/Fig_8D western blot/R3/ikba/western capsid.tiff]

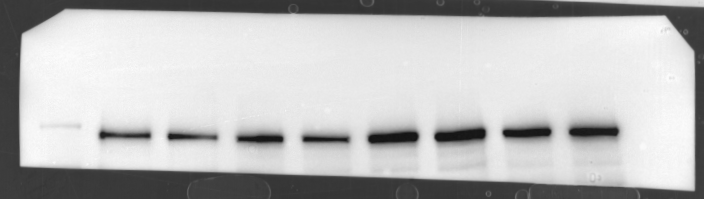

Supplement: Supplementary file 9 — Source Data Fig. 8 [file 44318_2024_35_MOESM9_ESM.zip › EMBOJ-2023-115792R2_SourceData_Fig8/Fig_8D western blot/R3/ikba/western dicer.tiff]

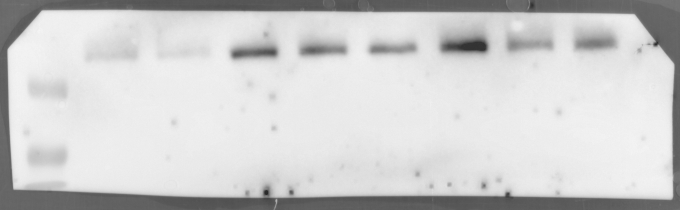

Supplement: Supplementary file 9 — Source Data Fig. 8 [file 44318_2024_35_MOESM9_ESM.zip › EMBOJ-2023-115792R2_SourceData_Fig8/Fig_8D western blot/R3/ikba/western ikba.tiff]

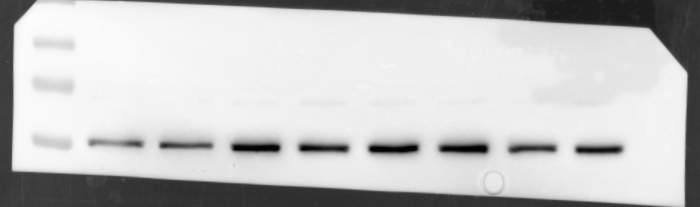

Supplement: Supplementary file 9 — Source Data Fig. 8 [file 44318_2024_35_MOESM9_ESM.zip › EMBOJ-2023-115792R2_SourceData_Fig8/Fig_8D western blot/R3/ikba/western tubulin.tiff]

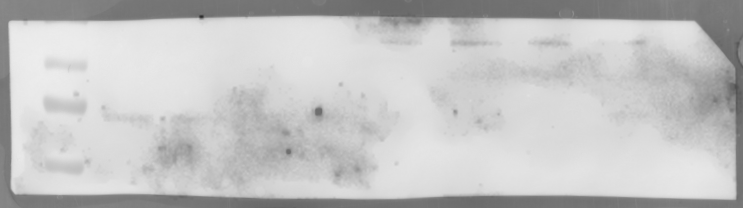

Supplement: Supplementary file 9 — Source Data Fig. 8 [file 44318_2024_35_MOESM9_ESM.zip › EMBOJ-2023-115792R2_SourceData_Fig8/Fig_8D western blot/R3/p65/western p-p65.tiff]

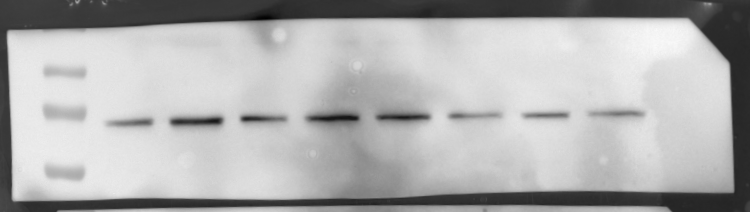

Supplement: Supplementary file 9 — Source Data Fig. 8 [file 44318_2024_35_MOESM9_ESM.zip › EMBOJ-2023-115792R2_SourceData_Fig8/Fig_8D western blot/R3/p65/western p65.tiff]

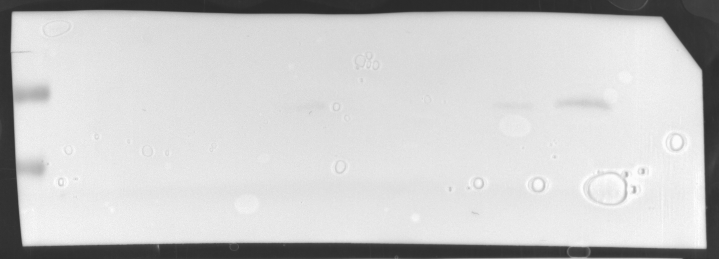

Supplement: Supplementary file 9 — Source Data Fig. 8 [file 44318_2024_35_MOESM9_ESM.zip › EMBOJ-2023-115792R2_SourceData_Fig8/Fig_8D western blot/R3/p65/western capsid.tiff]

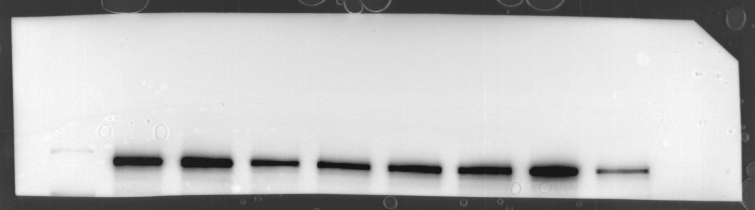

Supplement: Supplementary file 9 — Source Data Fig. 8 [file 44318_2024_35_MOESM9_ESM.zip › EMBOJ-2023-115792R2_SourceData_Fig8/Fig_8D western blot/R3/p65/western dicer.tiff]

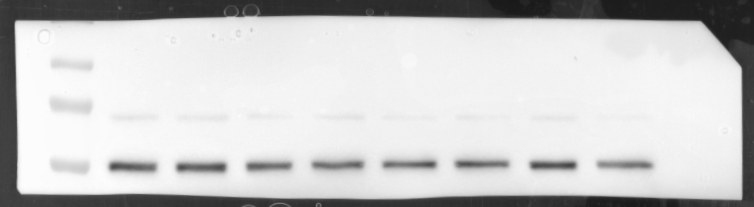

Supplement: Supplementary file 9 — Source Data Fig. 8 [file 44318_2024_35_MOESM9_ESM.zip › EMBOJ-2023-115792R2_SourceData_Fig8/Fig_8D western blot/R3/p65/western tubulin.tiff]

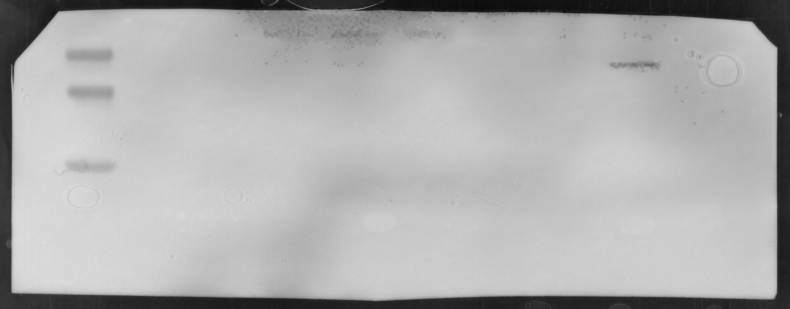

Supplement: Supplementary file 9 — Source Data Fig. 8 [file 44318_2024_35_MOESM9_ESM.zip › EMBOJ-2023-115792R2_SourceData_Fig8/Fig_8D western blot/R2/ikba/western capsid.tiff]

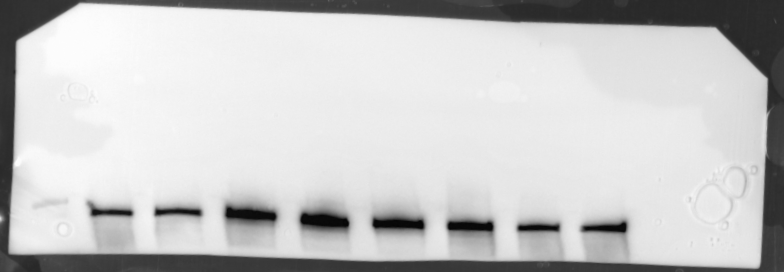

Supplement: Supplementary file 9 — Source Data Fig. 8 [file 44318_2024_35_MOESM9_ESM.zip › EMBOJ-2023-115792R2_SourceData_Fig8/Fig_8D western blot/R2/ikba/western dicer.tiff]

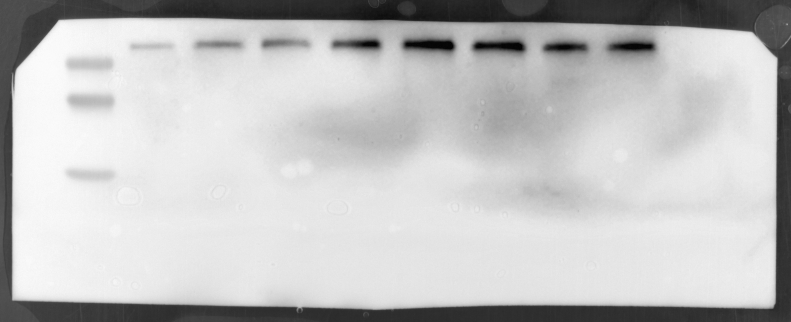

Supplement: Supplementary file 9 — Source Data Fig. 8 [file 44318_2024_35_MOESM9_ESM.zip › EMBOJ-2023-115792R2_SourceData_Fig8/Fig_8D western blot/R2/ikba/western ikba.tiff]

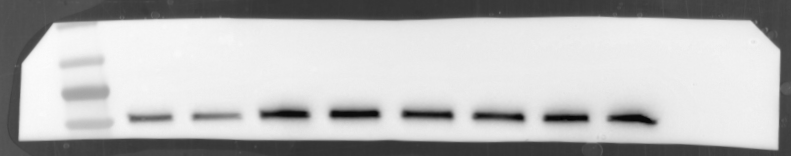

Supplement: Supplementary file 9 — Source Data Fig. 8 [file 44318_2024_35_MOESM9_ESM.zip › EMBOJ-2023-115792R2_SourceData_Fig8/Fig_8D western blot/R2/ikba/western tubulin.tiff]

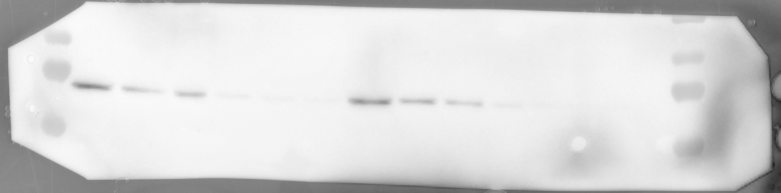

Supplement: Supplementary file 9 — Source Data Fig. 8 [file 44318_2024_35_MOESM9_ESM.zip › EMBOJ-2023-115792R2_SourceData_Fig8/Fig_8D western blot/R2/p65/western p-p65.tiff]

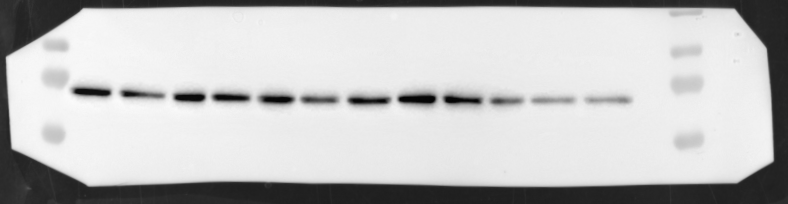

Supplement: Supplementary file 9 — Source Data Fig. 8 [file 44318_2024_35_MOESM9_ESM.zip › EMBOJ-2023-115792R2_SourceData_Fig8/Fig_8D western blot/R2/p65/western p65.tiff]

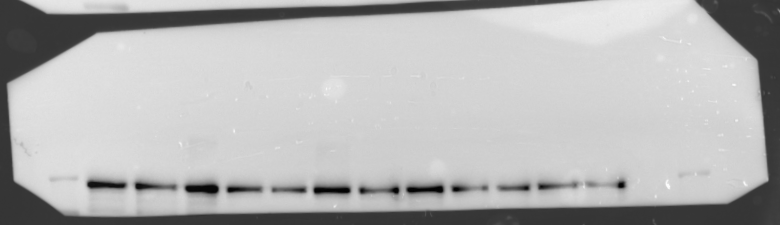

Supplement: Supplementary file 9 — Source Data Fig. 8 [file 44318_2024_35_MOESM9_ESM.zip › EMBOJ-2023-115792R2_SourceData_Fig8/Fig_8D western blot/R2/p65/western dicer.tiff]

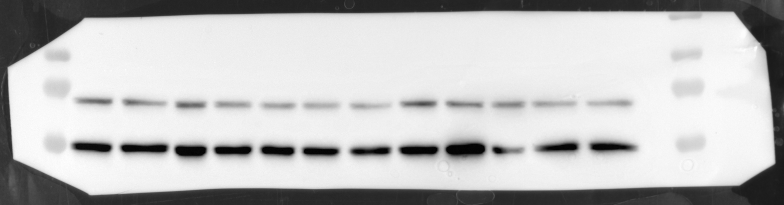

Supplement: Supplementary file 9 — Source Data Fig. 8 [file 44318_2024_35_MOESM9_ESM.zip › EMBOJ-2023-115792R2_SourceData_Fig8/Fig_8D western blot/R2/p65/western tubulin.tiff]

**Replicate 1**

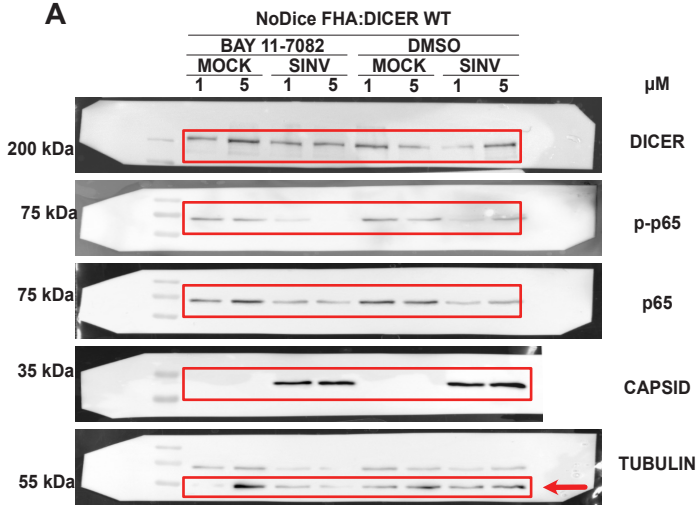

**Replicate 2**

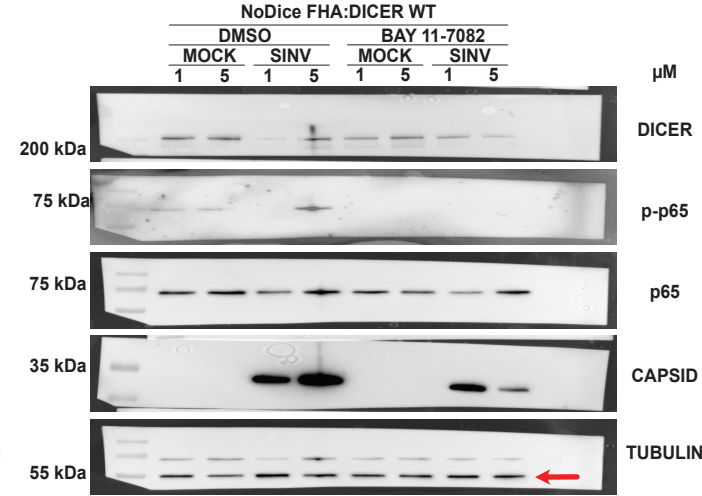

**Replicate 3**

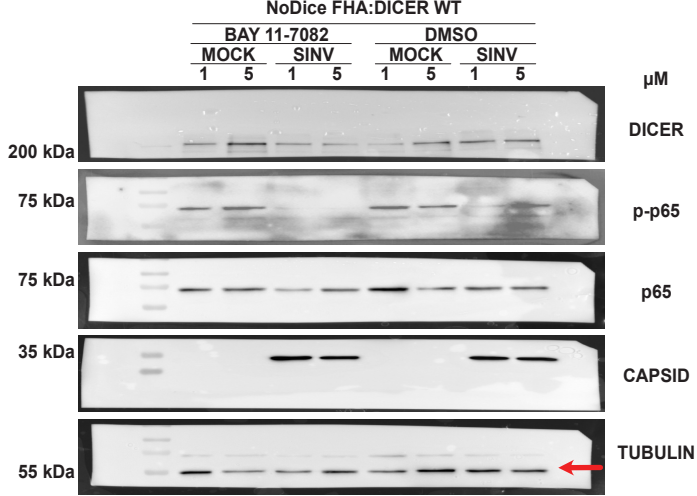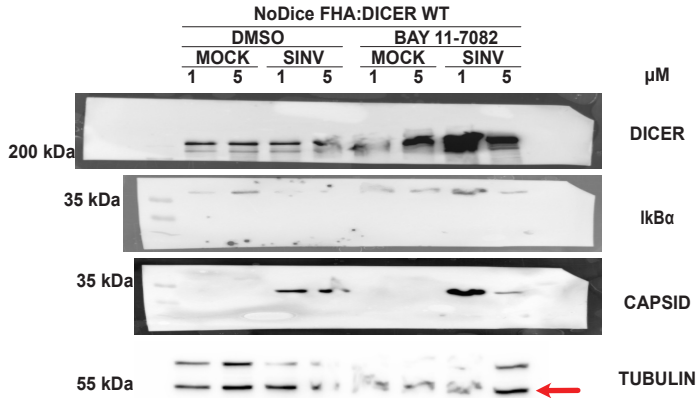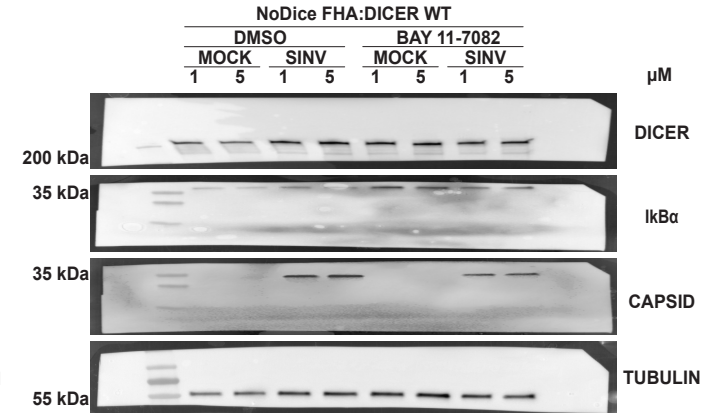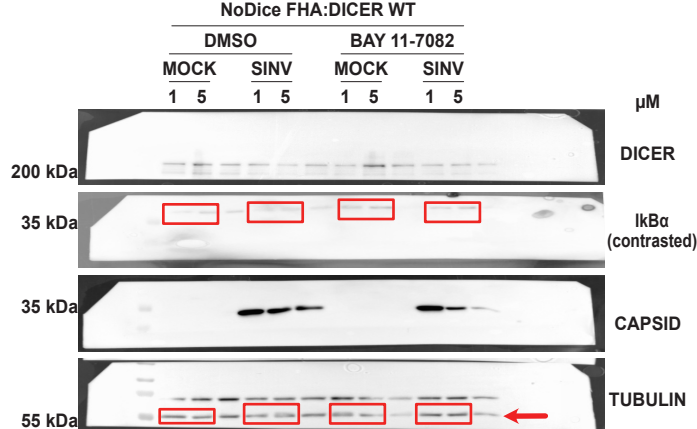

Supplement: Supplementary file 10 — Source Data of EV and Appendix figures [file 44318_2024_35_MOESM10_ESM.zip › EMBOJ-2023-115792R2_SourceData_EV+Appendix/Appendix figure S4/appendix figure S4.pdf]

A  
SARS-CoV-2 MOI  
0.001 48 hpi

Replicate 1

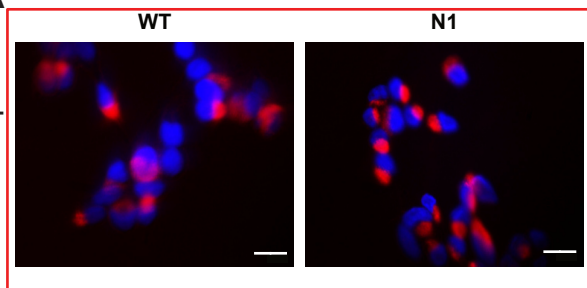

Replicate 2

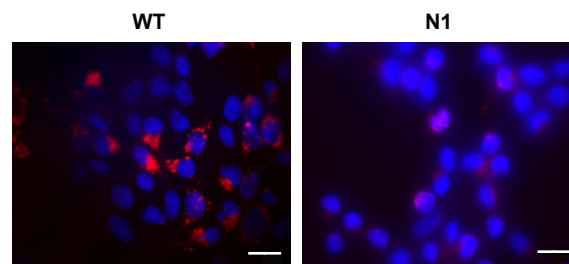

Replicate 3

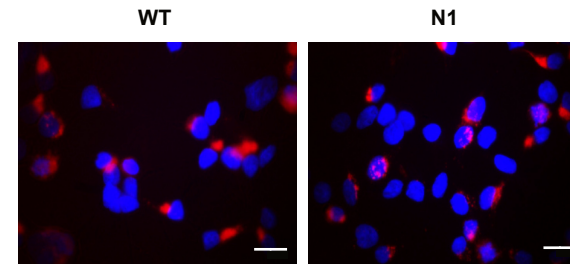

Supplement: Supplementary file 10 — Source Data of EV and Appendix figures [file 44318_2024_35_MOESM10_ESM.zip › EMBOJ-2023-115792R2_SourceData_EV+Appendix/Appendix Figure S2/appendix Figure S2.pdf]

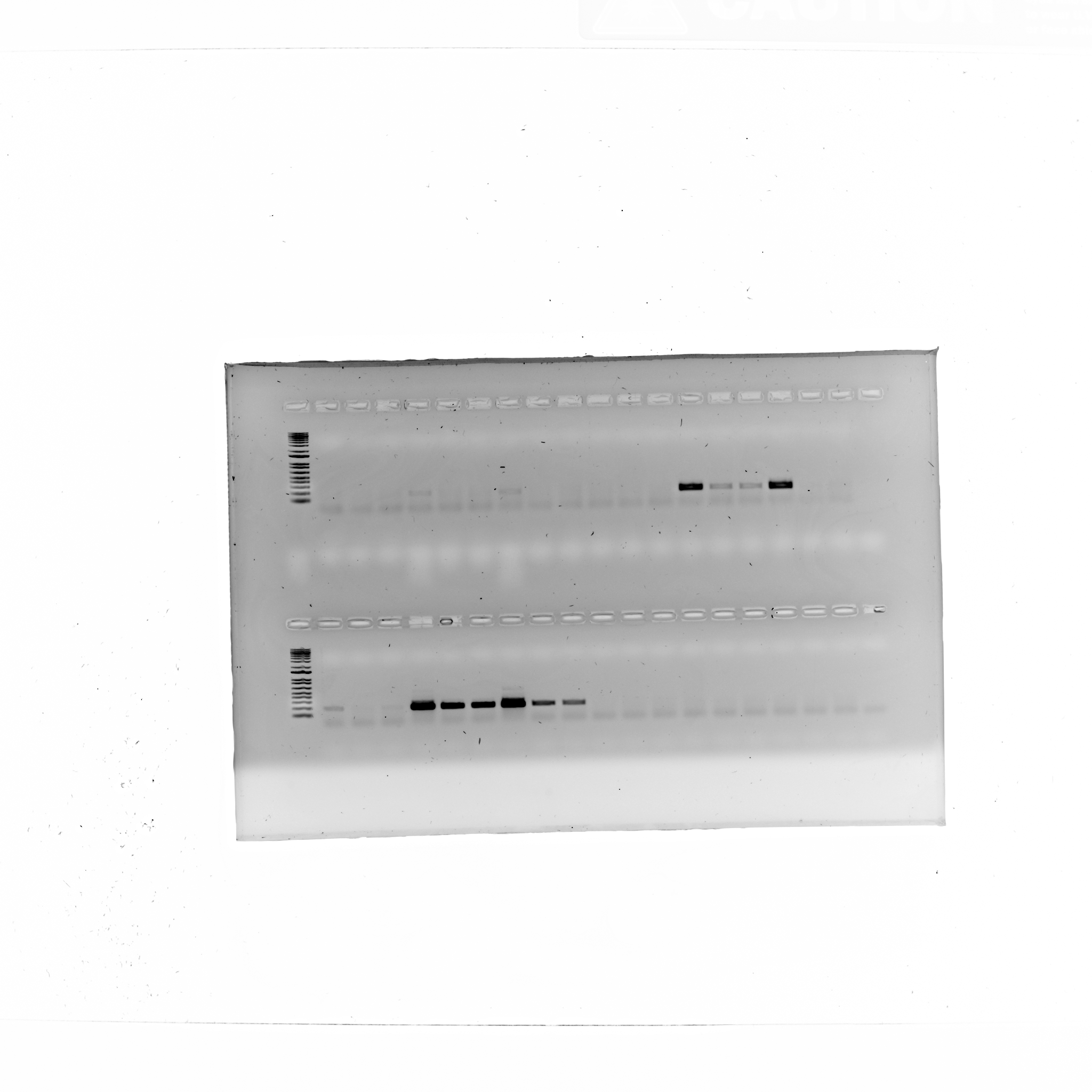

Supplement: Supplementary file 10 — Source Data of EV and Appendix figures [file 44318_2024_35_MOESM10_ESM.zip › EMBOJ-2023-115792R2_SourceData_EV+Appendix/Appendix Figure S1/Appendix_FigS1C PCR gel/R1/R1.tiff]

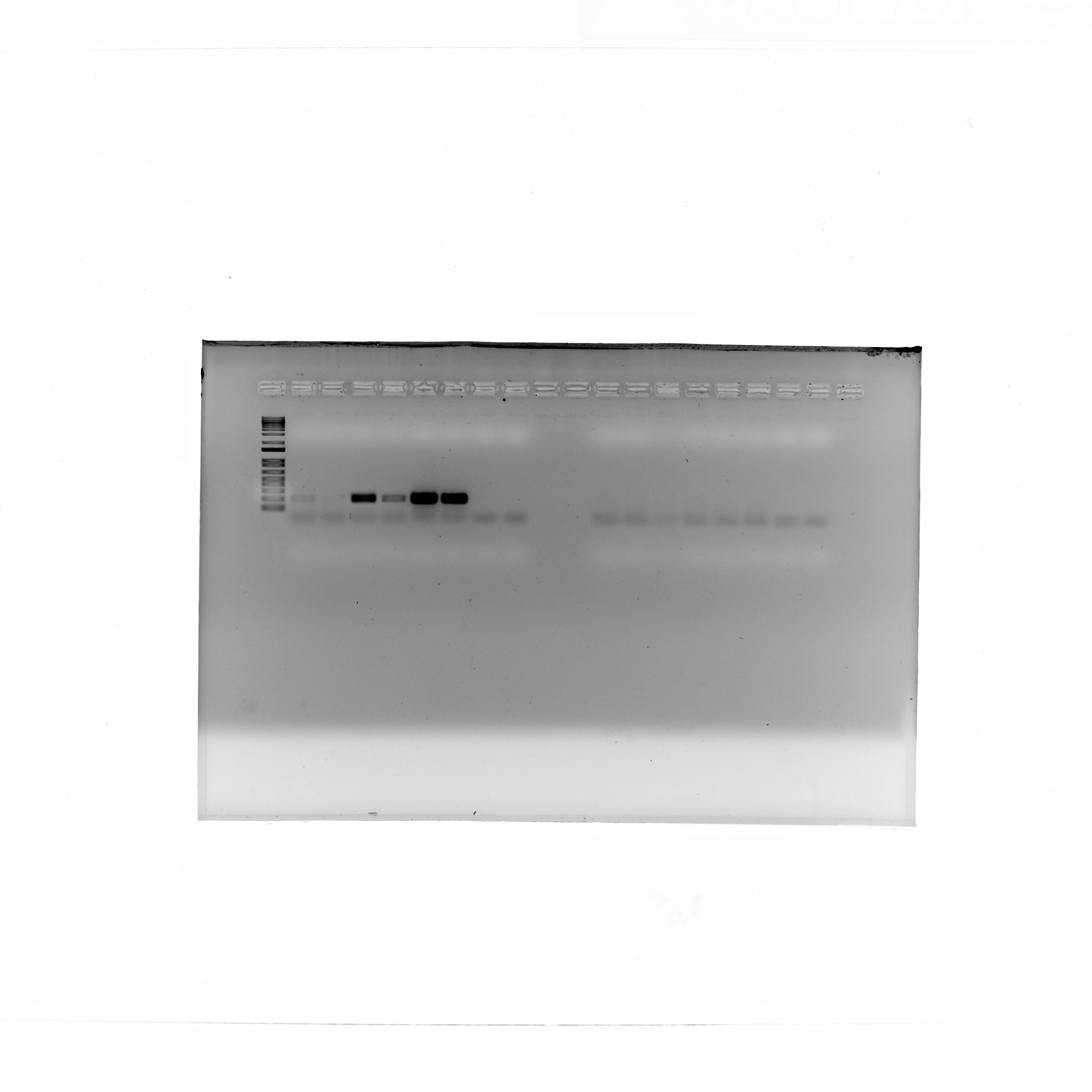

Supplement: Supplementary file 10 — Source Data of EV and Appendix figures [file 44318_2024_35_MOESM10_ESM.zip › EMBOJ-2023-115792R2_SourceData_EV+Appendix/Appendix Figure S1/Appendix_FigS1C PCR gel/R3/r3.tiff]

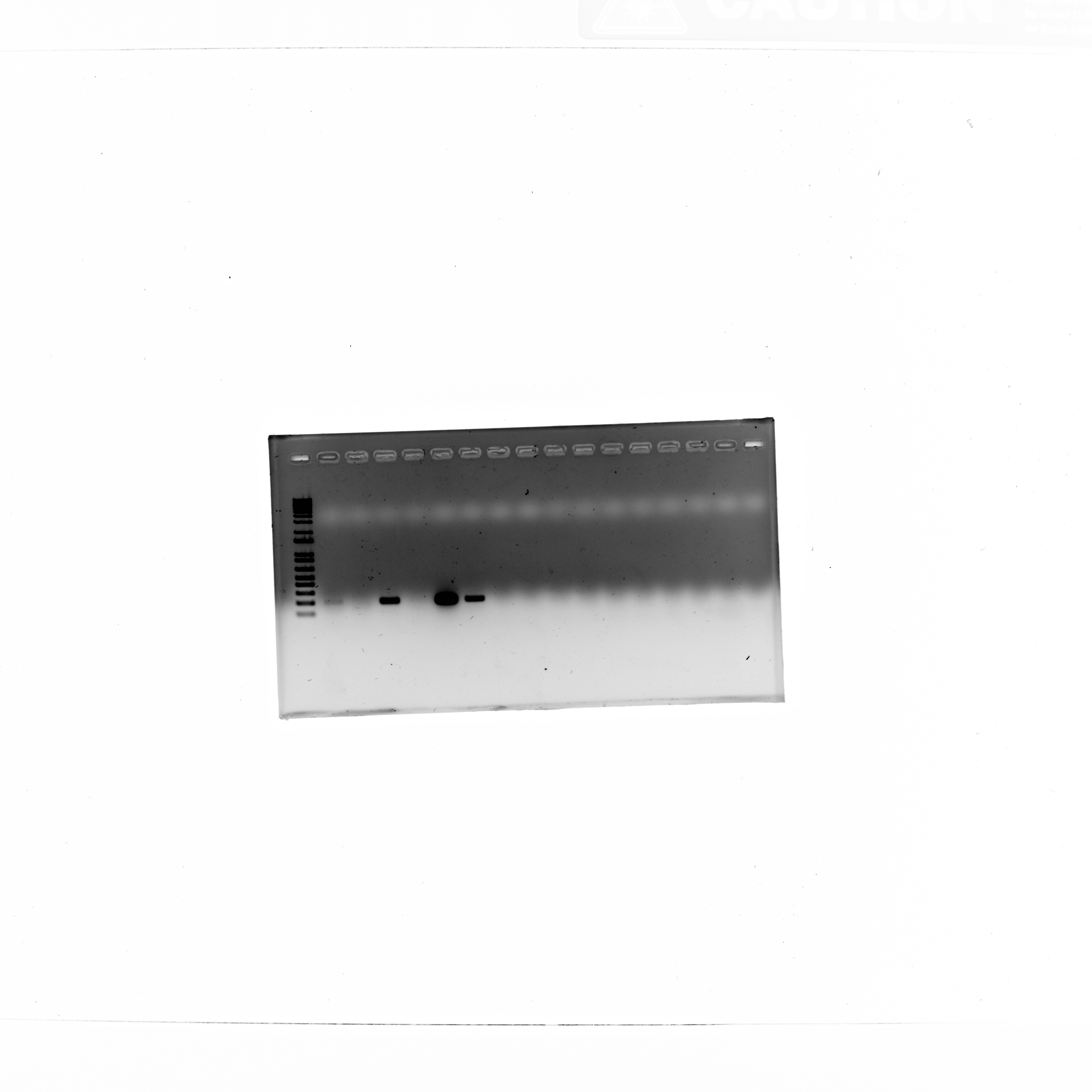

Supplement: Supplementary file 10 — Source Data of EV and Appendix figures [file 44318_2024_35_MOESM10_ESM.zip › EMBOJ-2023-115792R2_SourceData_EV+Appendix/Appendix Figure S1/Appendix_FigS1C PCR gel/R2/R2.tiff]

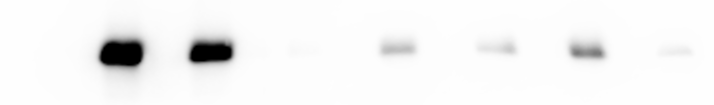

Supplement: Supplementary file 10 — Source Data of EV and Appendix figures [file 44318_2024_35_MOESM10_ESM.zip › EMBOJ-2023-115792R2_SourceData_EV+Appendix/FigEV1/FigEV1D western blot/R1/western capsid.tiff]

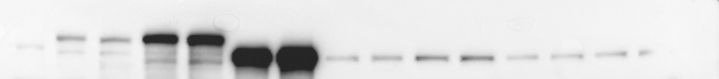

Supplement: Supplementary file 10 — Source Data of EV and Appendix figures [file 44318_2024_35_MOESM10_ESM.zip › EMBOJ-2023-115792R2_SourceData_EV+Appendix/FigEV1/FigEV1D western blot/R1/western dicer.tiff]

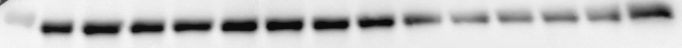

Supplement: Supplementary file 10 — Source Data of EV and Appendix figures [file 44318_2024_35_MOESM10_ESM.zip › EMBOJ-2023-115792R2_SourceData_EV+Appendix/FigEV1/FigEV1D western blot/R1/western tubulin.tiff]

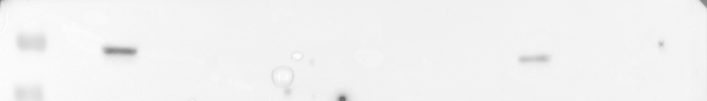

Supplement: Supplementary file 10 — Source Data of EV and Appendix figures [file 44318_2024_35_MOESM10_ESM.zip › EMBOJ-2023-115792R2_SourceData_EV+Appendix/FigEV1/FigEV1D western blot/R3/western capsid.tiff]

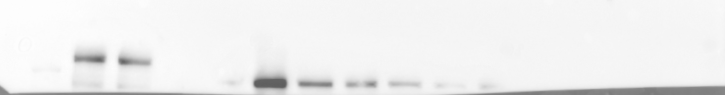

Supplement: Supplementary file 10 — Source Data of EV and Appendix figures [file 44318_2024_35_MOESM10_ESM.zip › EMBOJ-2023-115792R2_SourceData_EV+Appendix/FigEV1/FigEV1D western blot/R3/western dicer.tiff]
