# Supplementary material for: The helicase domain of human Dicer prevents RNAi-independent activation of antiviral and inflammatory pathways
Source: EMBO J. 2024 Jan 29;43(5):7. doi: 10.1038/s44318-024-00035-2 (PMC10907635; doi:10.1038/s44318-024-00035-2)
Supplement: Supplementary file 10 — Source Data of EV and Appendix figures [file 44318_2024_35_MOESM10_ESM.zip › EMBOJ-2023-115792R2_SourceData_EV+Appendix/Appendix Figure S1/Appendix Figure S1.pdf]

Replicate 1

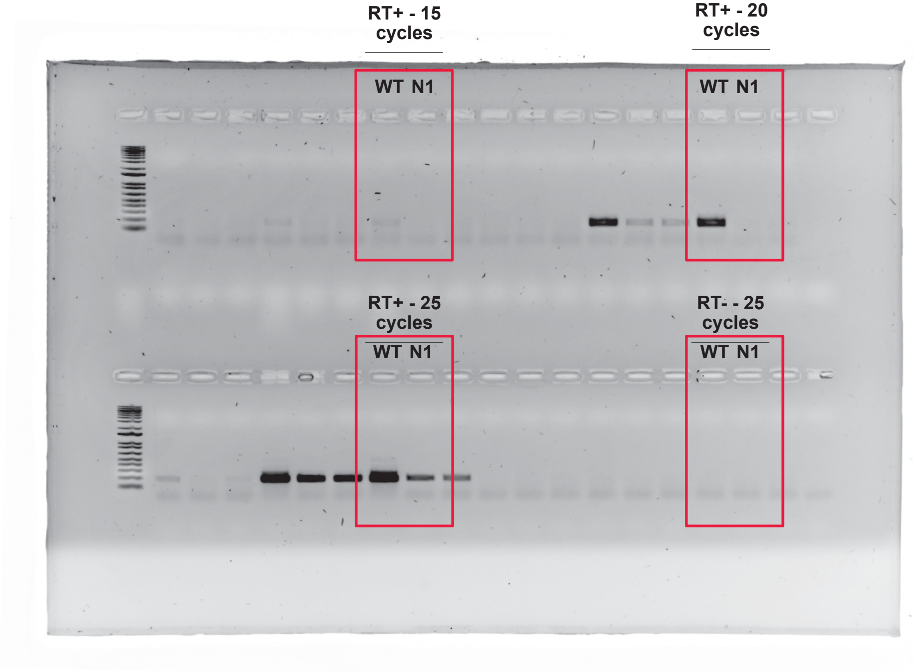

Replicate 2

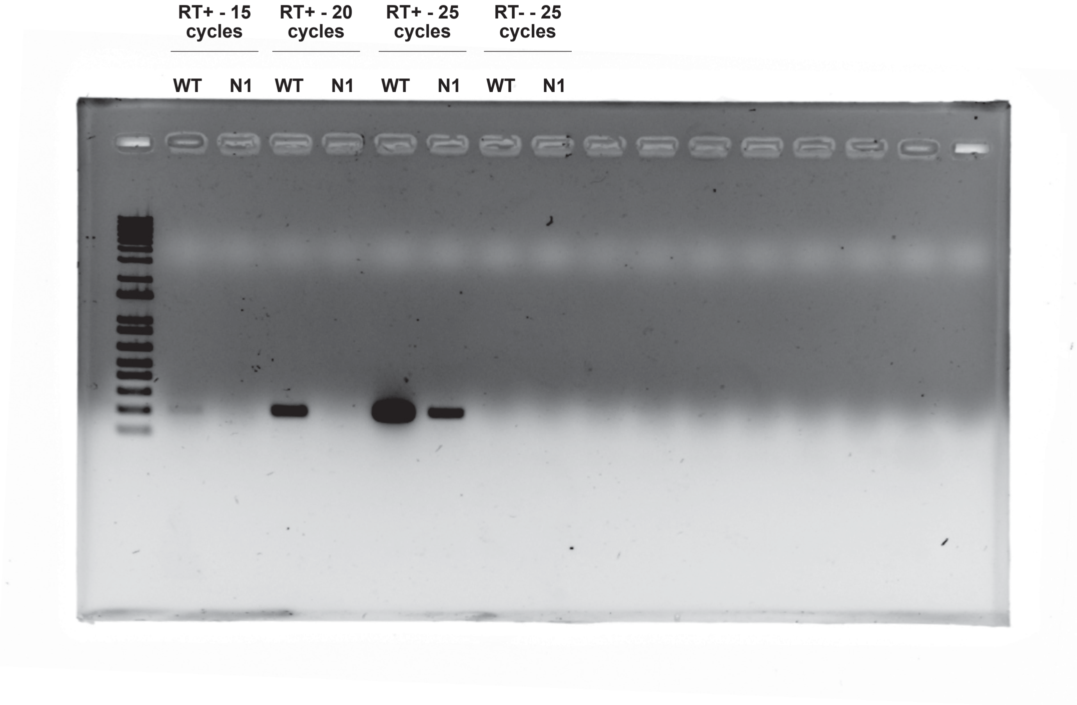

Replicate 3

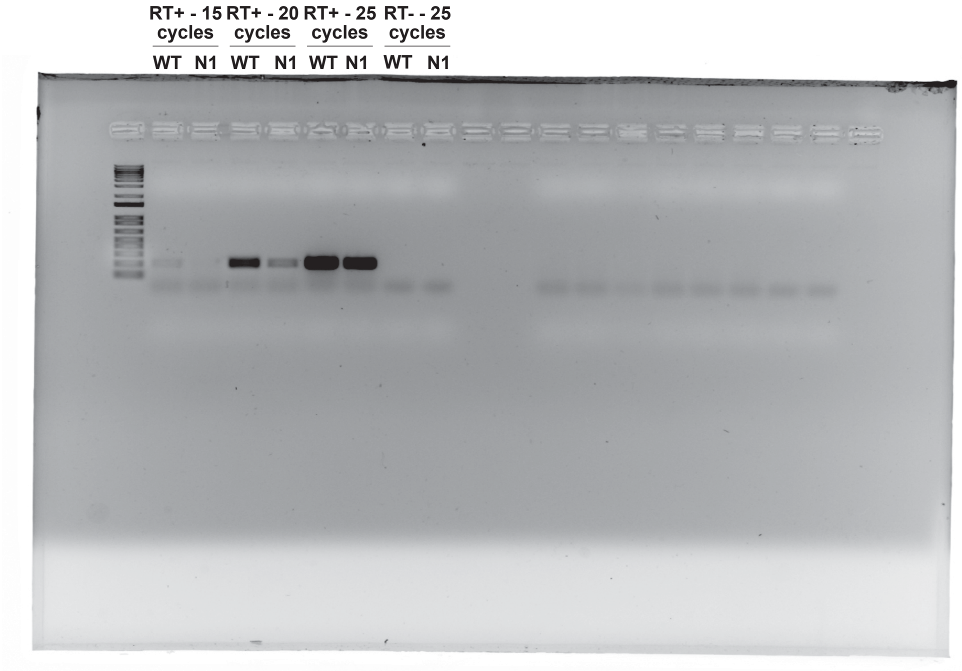

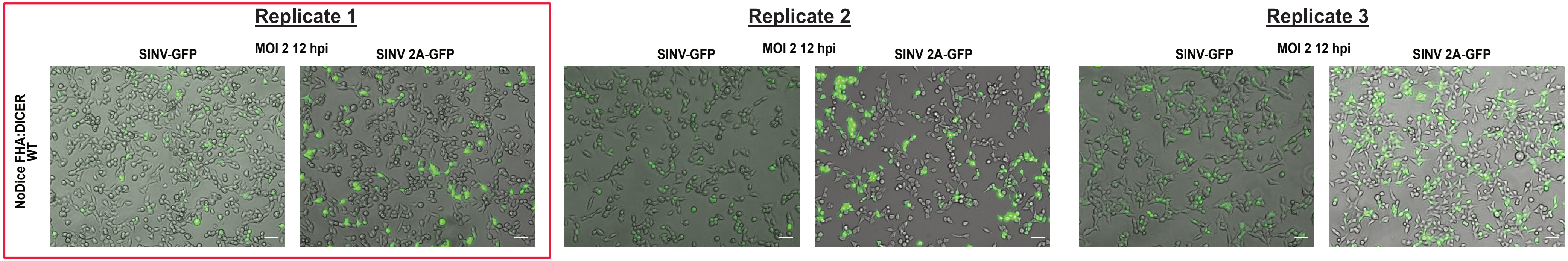

Replicate 1
Replicate 2
Replicate 3
SINV-GFP
MOI 2 12 hpi
SINV 2A-GFP
SINV-GFP
MOI 2 12 hpi
SINV 2A-GFP
SINV-GFP
MOI 2 12 hpi
SINV 2A-GFP
NoDice FHA:DICER WT
