# Supplementary material for: The helicase domain of human Dicer prevents RNAi-independent activation of antiviral and inflammatory pathways
Source: EMBO J. 2024 Jan 29;43(5):7. doi: 10.1038/s44318-024-00035-2 (PMC10907635; doi:10.1038/s44318-024-00035-2)
Supplement: Supplementary file 10 — Source Data of EV and Appendix figures [file 44318_2024_35_MOESM10_ESM.zip › EMBOJ-2023-115792R2_SourceData_EV+Appendix/FigEV1/FigEV1.pdf]

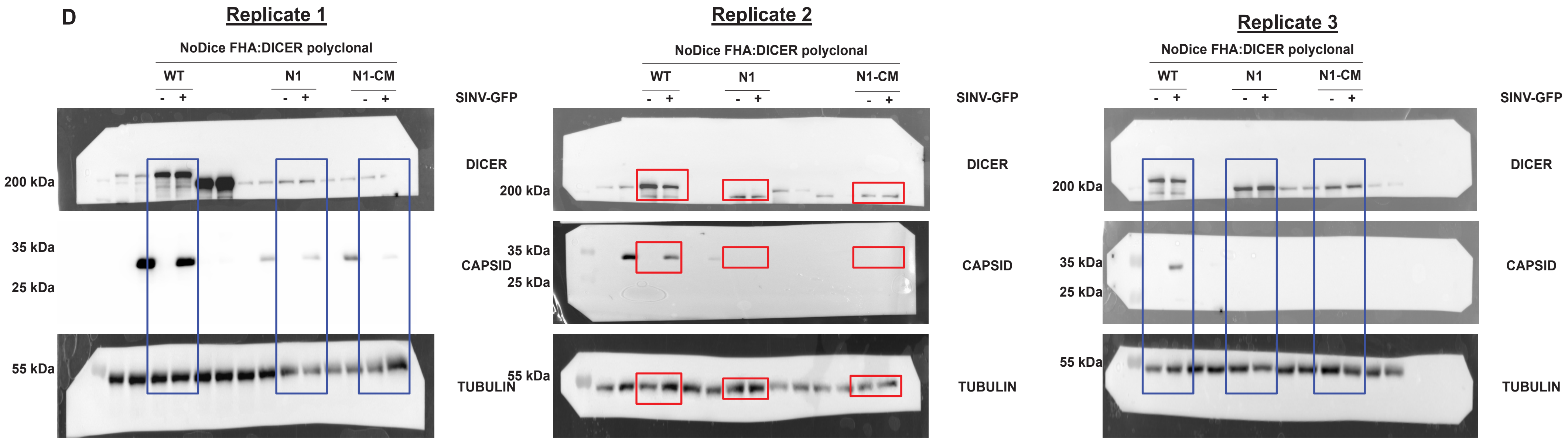
D
Replicate 1
NoDice FHA:DICER polyclonal
WT
N1
N1-CM
- +
- +
- +
SINV-GFP
DICER
200 kDa
35 kDa
CAPSID
25 kDa
55 kDa
TUBULIN
Replicate 2
NoDice FHA:DICER polyclonal
WT
N1
N1-CM
- +
- +
- +
SINV-GFP
DICER
200 kDa
35 kDa
CAPSID
25 kDa
55 kDa
TUBULIN
Replicate 3
NoDice FHA:DICER polyclonal
WT
N1
N1-CM
- +
- +
- +
SINV-GFP
DICER
200 kDa
35 kDa
CAPSID
25 kDa
55 kDa
TUBULIN

F

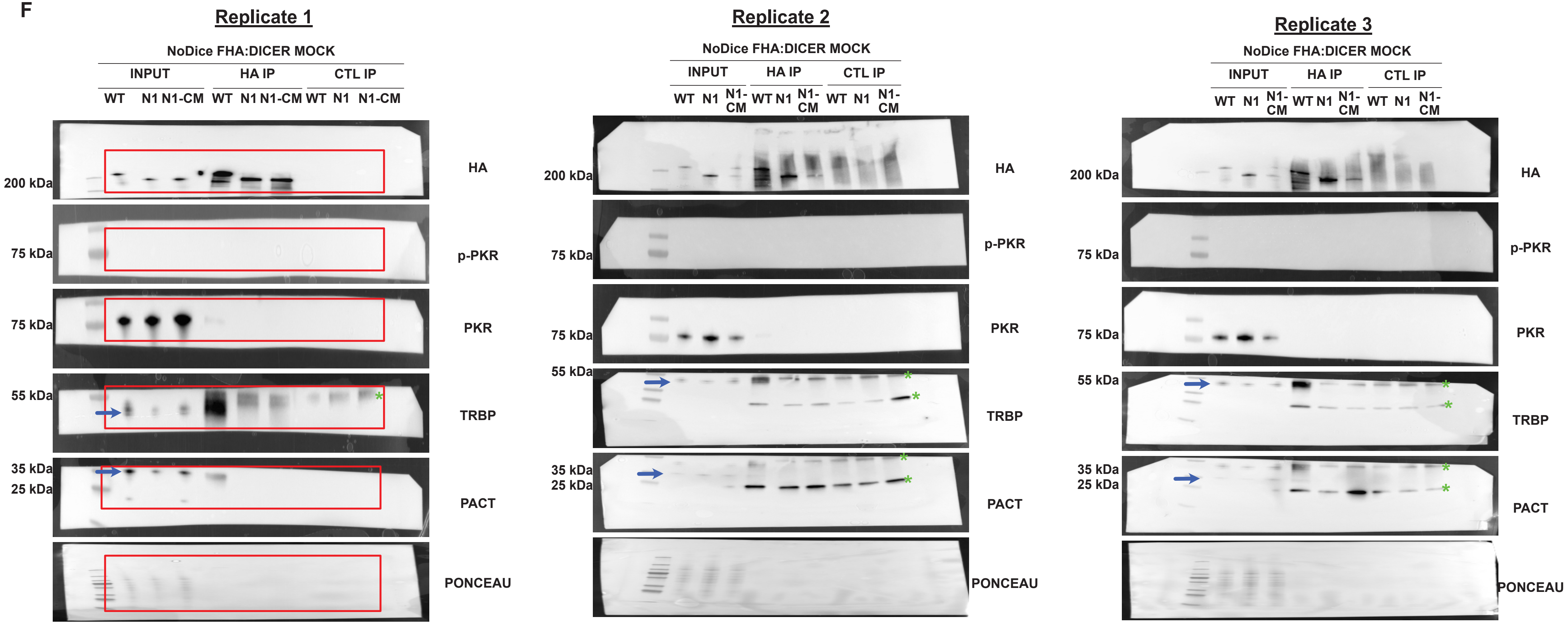

Replicate 1
Replicate 2
Replicate 3
NoDice FHA:DICER MOCK
INPUT
HA IP
CTL IP
WT
N1
N1-CM
200 kDa
75 kDa
55 kDa
35 kDa
25 kDa
HA
p-PKR
PKR
TRBP
PACT
PONCEAU

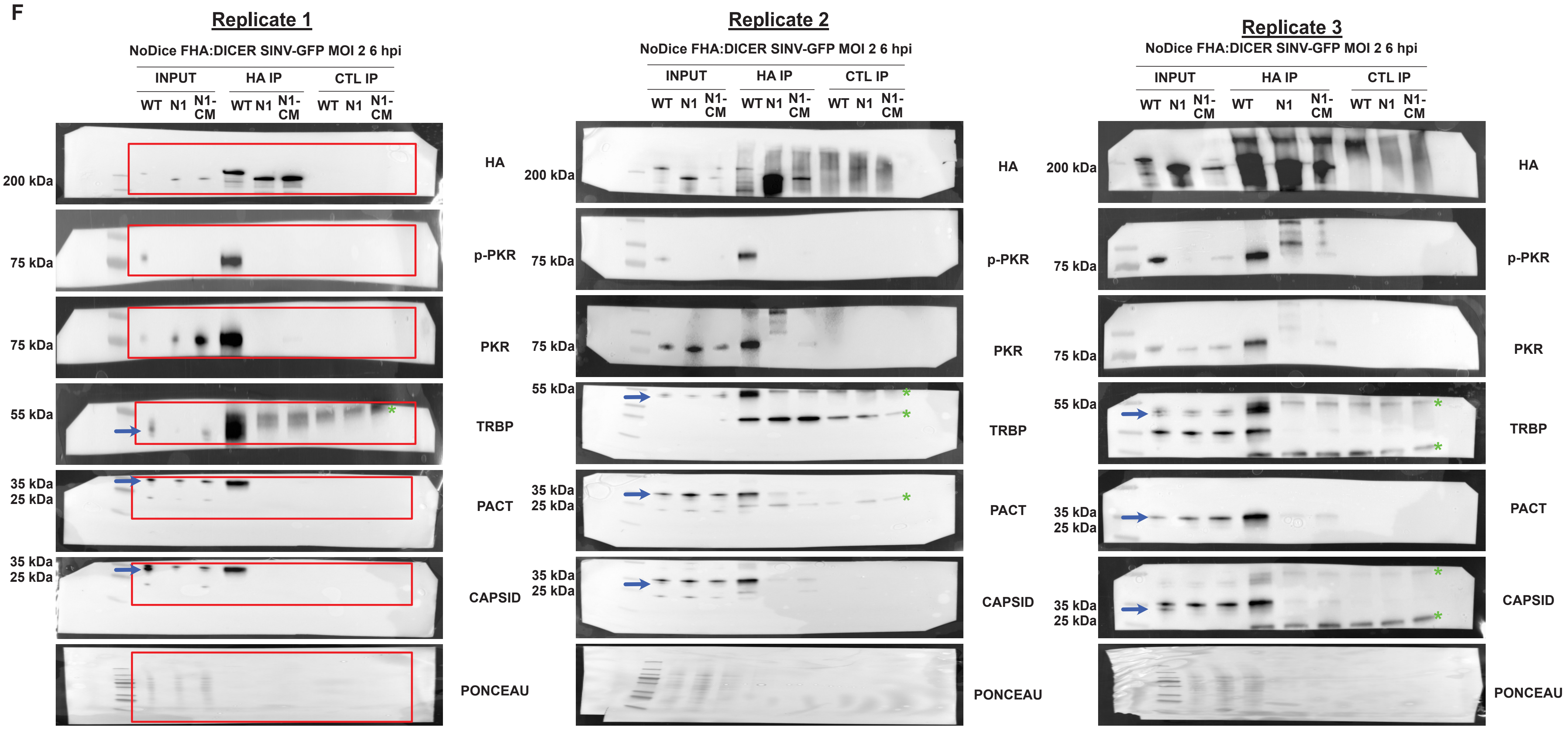
F
Replicate 1
NoDice FHA:DICER SINV-GFP MOI 2 6 hpi
INPUT
HA IP
CTL IP
WT N1 N1-CM WT N1 N1-CM WT N1 N1-CM
200 kDa
75 kDa
75 kDa
55 kDa
35 kDa
25 kDa
35 kDa
25 kDa
HA
p-PKR
PKR
TRBP
PACT
CAPSID
PONCEAU
Replicate 2
NoDice FHA:DICER SINV-GFP MOI 2 6 hpi
INPUT
HA IP
CTL IP
WT N1 N1-CM WT N1 N1-CM WT N1 N1-CM
200 kDa
75 kDa
75 kDa
55 kDa
35 kDa
25 kDa
35 kDa
25 kDa
HA
p-PKR
PKR
TRBP
PACT
CAPSID
PONCEAU
Replicate 3
NoDice FHA:DICER SINV-GFP MOI 2 6 hpi
INPUT
HA IP
CTL IP
WT N1 N1-CM WT N1 N1-CM WT N1 N1-CM
200 kDa
75 kDa
75 kDa
55 kDa
35 kDa
25 kDa
35 kDa
25 kDa
HA
p-PKR
PKR
TRBP
PACT
CAPSID
PONCEAU
