# Supplementary material for: The helicase domain of human Dicer prevents RNAi-independent activation of antiviral and inflammatory pathways
Source: EMBO J. 2024 Jan 29;43(5):7. doi: 10.1038/s44318-024-00035-2 (PMC10907635; doi:10.1038/s44318-024-00035-2)
Supplement: Supplementary file 10 — Source Data of EV and Appendix figures [file 44318_2024_35_MOESM10_ESM.zip › EMBOJ-2023-115792R2_SourceData_EV+Appendix/FigEV3/FigEV3.pdf]

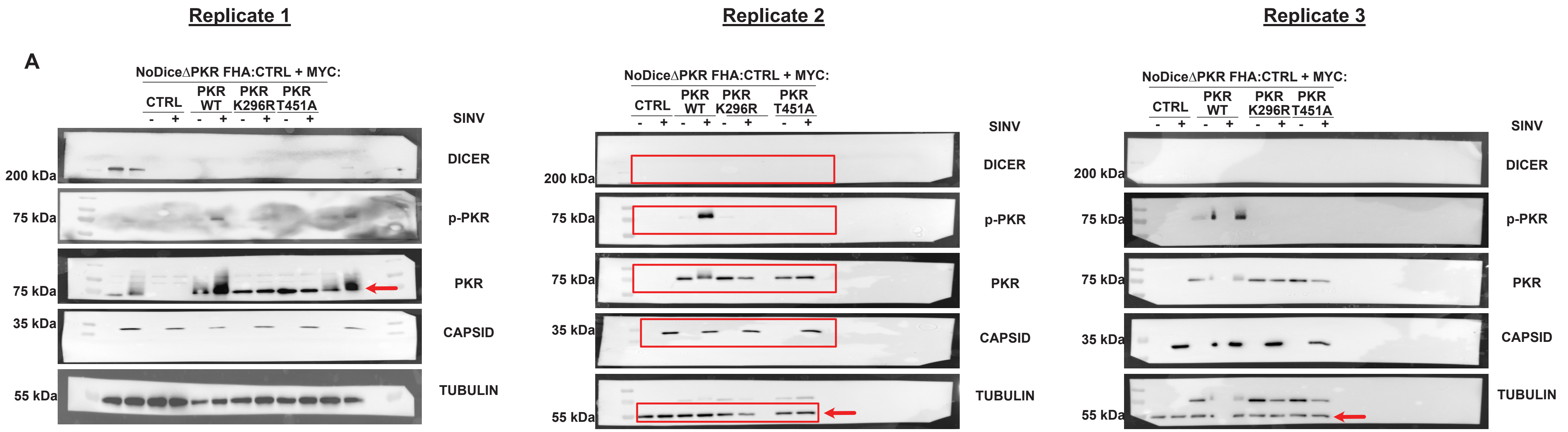

Replicate 1
Replicate 2
Replicate 3
A
NoDiceΔPKR FHA:CTRL + MYC:
CTRL
PKR WT
PKR K296R
PKR T451A
- + - + - + - +
SINV
DICER
p-PKR
PKR
CAPSID
TUBULIN
200 kDa
75 kDa
75 kDa
35 kDa
55 kDa
