# Supplementary material for: The helicase domain of human Dicer prevents RNAi-independent activation of antiviral and inflammatory pathways
Source: EMBO J. 2024 Jan 29;43(5):7. doi: 10.1038/s44318-024-00035-2 (PMC10907635; doi:10.1038/s44318-024-00035-2)
Supplement: Supplementary file 10 — Source Data of EV and Appendix figures [file 44318_2024_35_MOESM10_ESM.zip › EMBOJ-2023-115792R2_SourceData_EV+Appendix/FigEV2/FigEV2.pdf]

A

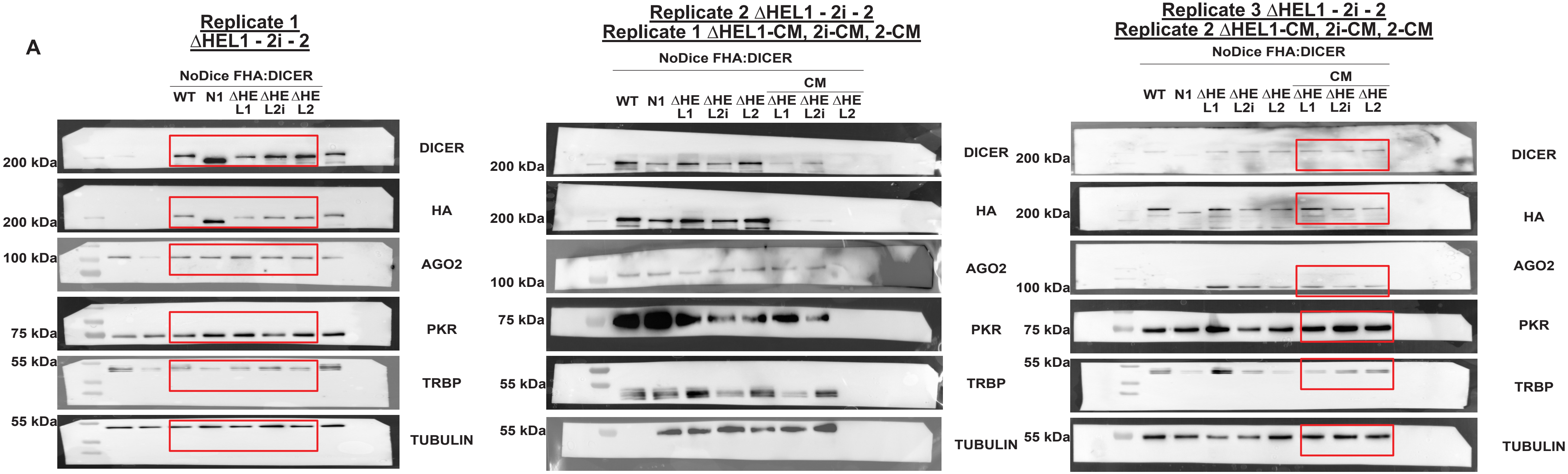
Replicate 1
ΔHEL1 - 2i - 2
NoDice FHA:DICER
WT
N1
ΔHE L1
ΔHE L2i
ΔHE L2
200 kDa
200 kDa
100 kDa
75 kDa
55 kDa
55 kDa
DICER
HA
AGO2
PKR
TRBP
TUBULIN
Replicate 2 ΔHEL1 - 2i - 2
Replicate 1 ΔHEL1-CM, 2i-CM, 2-CM
NoDice FHA:DICER
CM
WT
N1
ΔHE L1
ΔHE L2i
ΔHE L2
ΔHE L1
ΔHE L2i
ΔHE L2
200 kDa
200 kDa
100 kDa
75 kDa
55 kDa
55 kDa
DICER
HA
AGO2
PKR
TRBP
TUBULIN
Replicate 3 ΔHEL1 - 2i - 2
Replicate 2 ΔHEL1-CM, 2i-CM, 2-CM
NoDice FHA:DICER
CM
WT
N1
ΔHE L1
ΔHE L2i
ΔHE L2
ΔHE L1
ΔHE L2i
ΔHE L2
200 kDa
200 kDa
100 kDa
75 kDa
55 kDa
55 kDa
DICER
HA
AGO2
PKR
TRBP
TUBULIN

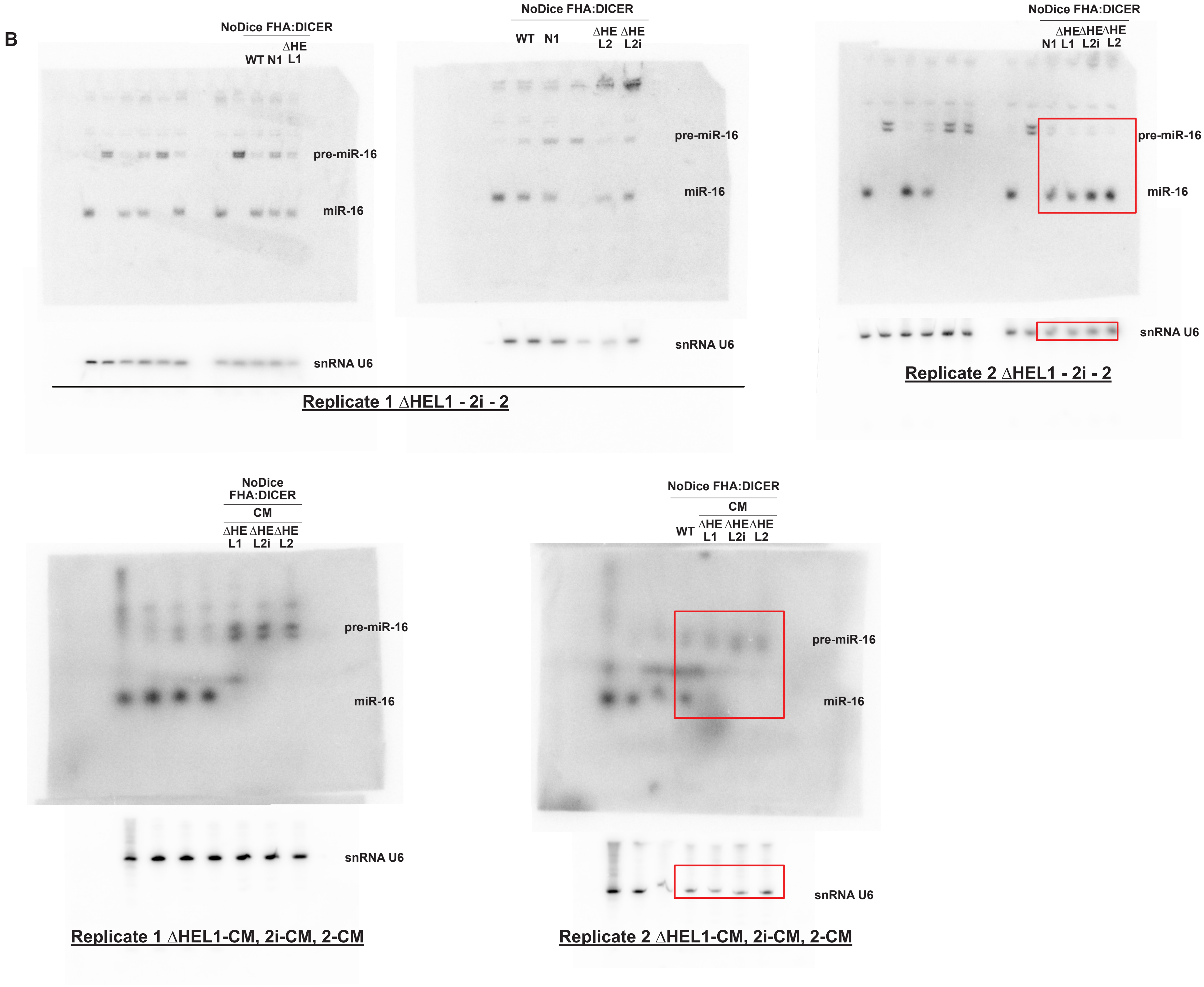
B
NoDice FHA:DICER
WT N1 ΔHE L1
pre-miR-16
miR-16
snRNA U6
Replicate 1 ΔHEL1 - 2i - 2
NoDice FHA:DICER
WT N1 ΔHE L2 ΔHE L2i
pre-miR-16
miR-16
snRNA U6
NoDice FHA:DICER
N1 ΔHE L1 ΔHE L2i ΔHE L2
pre-miR-16
miR-16
snRNA U6
Replicate 2 ΔHEL1 - 2i - 2
NoDice FHA:DICER
CM
ΔHE L1 ΔHE L2i ΔHE L2
pre-miR-16
miR-16
snRNA U6
Replicate 1 ΔHEL1-CM, 2i-CM, 2-CM
NoDice FHA:DICER
CM
WT ΔHE L1 ΔHE L2i ΔHE L2
pre-miR-16
miR-16
snRNA U6
Replicate 2 ΔHEL1-CM, 2i-CM, 2-CM

C

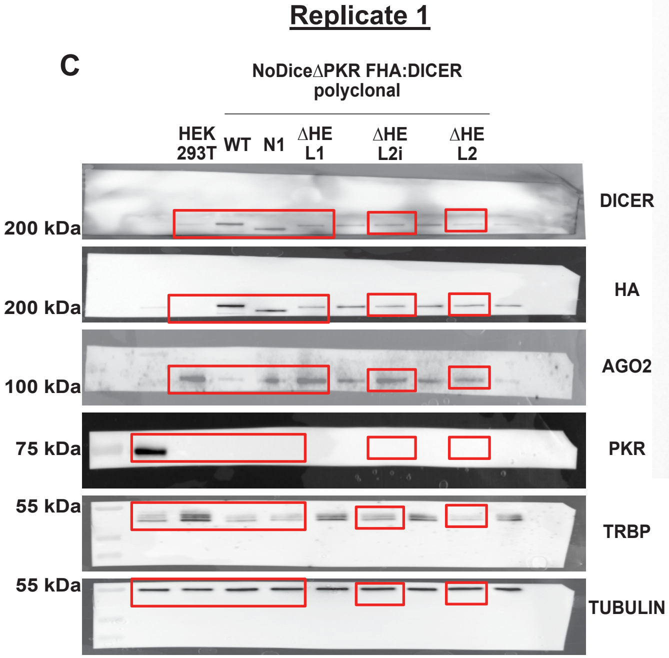

Replicate 1
NoDiceΔPKR FHA:DICER
polyclonal
HEK 293T
WT
N1
ΔHEL1
ΔHEL2i
ΔHEL2
200 kDa
DICER
200 kDa
HA
100 kDa
AGO2
75 kDa
PKR
55 kDa
TRBP
55 kDa
TUBULIN

D

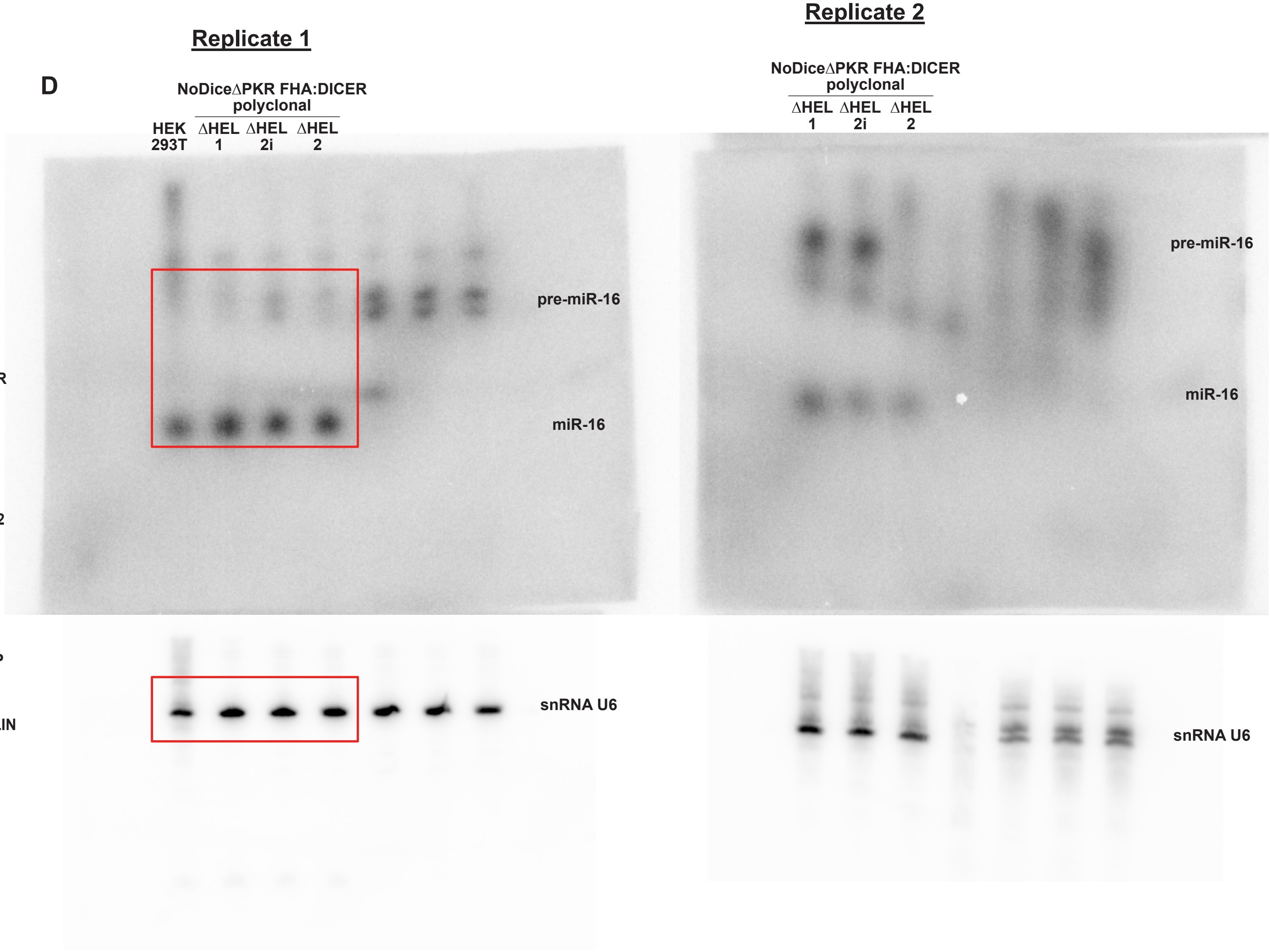

Replicate 1
NoDiceΔPKR FHA:DICER
polyclonal
HEK 293T
ΔHEL 1
ΔHEL 2i
ΔHEL 2
pre-miR-16
miR-16
snRNA U6
Replicate 2
NoDiceΔPKR FHA:DICER
polyclonal
ΔHEL 1
ΔHEL 2i
ΔHEL 2
pre-miR-16
miR-16
snRNA U6
