# Supplementary figures and images for: The helicase domain of human Dicer prevents RNAi-independent activation of antiviral and inflammatory pathways (part 5 of 5)
Source: EMBO J. 2024 Jan 29;43(5):7. doi: 10.1038/s44318-024-00035-2 (PMC10907635; doi:10.1038/s44318-024-00035-2)

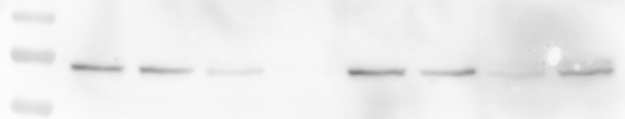

Supplement: Supplementary file 10 — Source Data of EV and Appendix figures [file 44318_2024_35_MOESM10_ESM.zip › EMBOJ-2023-115792R2_SourceData_EV+Appendix/Appendix figure S4/S4A western blot/R1/nfkb/western p-p65.tiff]

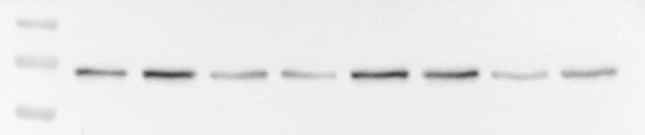

Supplement: Supplementary file 10 — Source Data of EV and Appendix figures [file 44318_2024_35_MOESM10_ESM.zip › EMBOJ-2023-115792R2_SourceData_EV+Appendix/Appendix figure S4/S4A western blot/R1/nfkb/western p65.tiff.tiff]

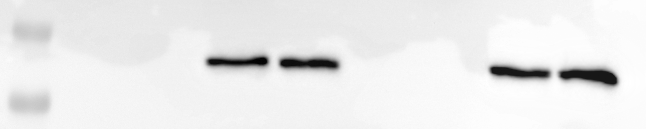

Supplement: Supplementary file 10 — Source Data of EV and Appendix figures [file 44318_2024_35_MOESM10_ESM.zip › EMBOJ-2023-115792R2_SourceData_EV+Appendix/Appendix figure S4/S4A western blot/R1/nfkb/western capsid.tiff]

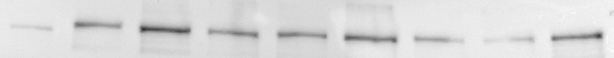

Supplement: Supplementary file 10 — Source Data of EV and Appendix figures [file 44318_2024_35_MOESM10_ESM.zip › EMBOJ-2023-115792R2_SourceData_EV+Appendix/Appendix figure S4/S4A western blot/R1/nfkb/western dicer.tiff]

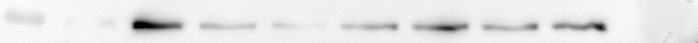

Supplement: Supplementary file 10 — Source Data of EV and Appendix figures [file 44318_2024_35_MOESM10_ESM.zip › EMBOJ-2023-115792R2_SourceData_EV+Appendix/Appendix figure S4/S4A western blot/R1/nfkb/western tubulin.tiff]

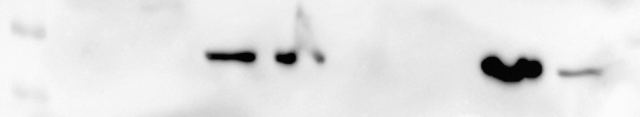

Supplement: Supplementary file 10 — Source Data of EV and Appendix figures [file 44318_2024_35_MOESM10_ESM.zip › EMBOJ-2023-115792R2_SourceData_EV+Appendix/Appendix figure S4/S4A western blot/R1/ikba/western capsid.tiff]

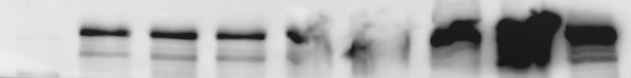

Supplement: Supplementary file 10 — Source Data of EV and Appendix figures [file 44318_2024_35_MOESM10_ESM.zip › EMBOJ-2023-115792R2_SourceData_EV+Appendix/Appendix figure S4/S4A western blot/R1/ikba/western dicer.tiff]

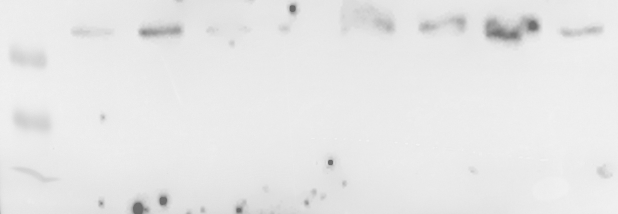

Supplement: Supplementary file 10 — Source Data of EV and Appendix figures [file 44318_2024_35_MOESM10_ESM.zip › EMBOJ-2023-115792R2_SourceData_EV+Appendix/Appendix figure S4/S4A western blot/R1/ikba/western ikba.tiff]

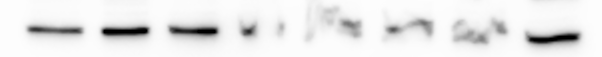

Supplement: Supplementary file 10 — Source Data of EV and Appendix figures [file 44318_2024_35_MOESM10_ESM.zip › EMBOJ-2023-115792R2_SourceData_EV+Appendix/Appendix figure S4/S4A western blot/R1/ikba/western tubulin.tiff]

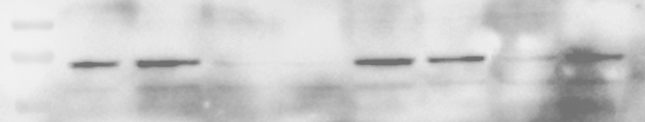

Supplement: Supplementary file 10 — Source Data of EV and Appendix figures [file 44318_2024_35_MOESM10_ESM.zip › EMBOJ-2023-115792R2_SourceData_EV+Appendix/Appendix figure S4/S4A western blot/R3/nfkb/western p-p65.tiff]

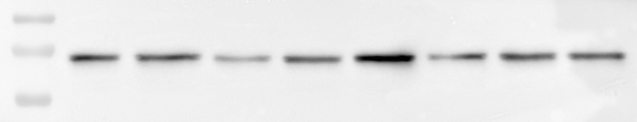

Supplement: Supplementary file 10 — Source Data of EV and Appendix figures [file 44318_2024_35_MOESM10_ESM.zip › EMBOJ-2023-115792R2_SourceData_EV+Appendix/Appendix figure S4/S4A western blot/R3/nfkb/western p65.tiff]

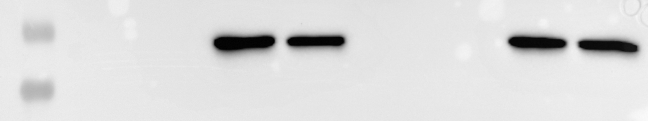

Supplement: Supplementary file 10 — Source Data of EV and Appendix figures [file 44318_2024_35_MOESM10_ESM.zip › EMBOJ-2023-115792R2_SourceData_EV+Appendix/Appendix figure S4/S4A western blot/R3/nfkb/western capsid.tiff]

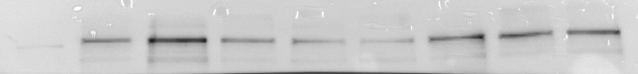

Supplement: Supplementary file 10 — Source Data of EV and Appendix figures [file 44318_2024_35_MOESM10_ESM.zip › EMBOJ-2023-115792R2_SourceData_EV+Appendix/Appendix figure S4/S4A western blot/R3/nfkb/western dicer.tiff]

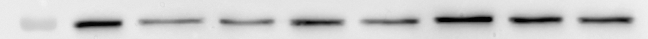

Supplement: Supplementary file 10 — Source Data of EV and Appendix figures [file 44318_2024_35_MOESM10_ESM.zip › EMBOJ-2023-115792R2_SourceData_EV+Appendix/Appendix figure S4/S4A western blot/R3/nfkb/western tubulin.tiff]

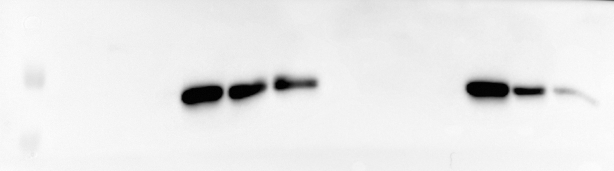

Supplement: Supplementary file 10 — Source Data of EV and Appendix figures [file 44318_2024_35_MOESM10_ESM.zip › EMBOJ-2023-115792R2_SourceData_EV+Appendix/Appendix figure S4/S4A western blot/R3/ikba/western capsid.tiff]

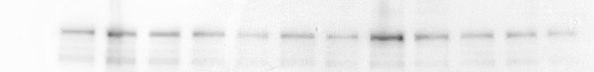

Supplement: Supplementary file 10 — Source Data of EV and Appendix figures [file 44318_2024_35_MOESM10_ESM.zip › EMBOJ-2023-115792R2_SourceData_EV+Appendix/Appendix figure S4/S4A western blot/R3/ikba/western dicer.tiff]

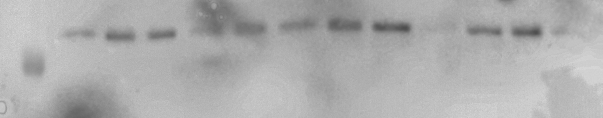

Supplement: Supplementary file 10 — Source Data of EV and Appendix figures [file 44318_2024_35_MOESM10_ESM.zip › EMBOJ-2023-115792R2_SourceData_EV+Appendix/Appendix figure S4/S4A western blot/R3/ikba/western contrasted ikba.tiff]

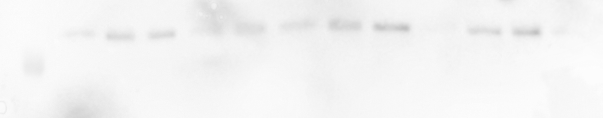

Supplement: Supplementary file 10 — Source Data of EV and Appendix figures [file 44318_2024_35_MOESM10_ESM.zip › EMBOJ-2023-115792R2_SourceData_EV+Appendix/Appendix figure S4/S4A western blot/R3/ikba/western ikba.tiff]

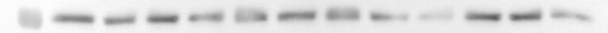

Supplement: Supplementary file 10 — Source Data of EV and Appendix figures [file 44318_2024_35_MOESM10_ESM.zip › EMBOJ-2023-115792R2_SourceData_EV+Appendix/Appendix figure S4/S4A western blot/R3/ikba/western tubulin.tiff]

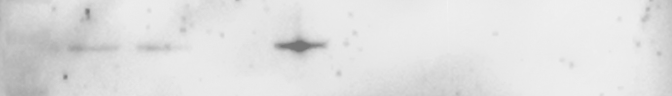

Supplement: Supplementary file 10 — Source Data of EV and Appendix figures [file 44318_2024_35_MOESM10_ESM.zip › EMBOJ-2023-115792R2_SourceData_EV+Appendix/Appendix figure S4/S4A western blot/R2/nfkb/western p-p65.tiff]

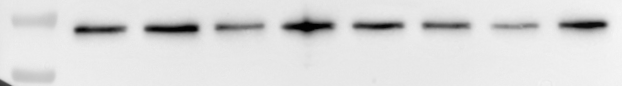

Supplement: Supplementary file 10 — Source Data of EV and Appendix figures [file 44318_2024_35_MOESM10_ESM.zip › EMBOJ-2023-115792R2_SourceData_EV+Appendix/Appendix figure S4/S4A western blot/R2/nfkb/western p65.tiff]

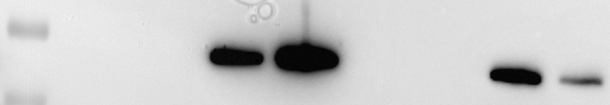

Supplement: Supplementary file 10 — Source Data of EV and Appendix figures [file 44318_2024_35_MOESM10_ESM.zip › EMBOJ-2023-115792R2_SourceData_EV+Appendix/Appendix figure S4/S4A western blot/R2/nfkb/western capsid.tiff]

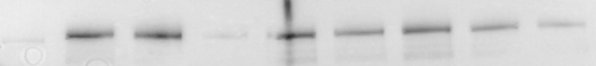

Supplement: Supplementary file 10 — Source Data of EV and Appendix figures [file 44318_2024_35_MOESM10_ESM.zip › EMBOJ-2023-115792R2_SourceData_EV+Appendix/Appendix figure S4/S4A western blot/R2/nfkb/western dicer.tiff]

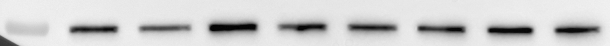

Supplement: Supplementary file 10 — Source Data of EV and Appendix figures [file 44318_2024_35_MOESM10_ESM.zip › EMBOJ-2023-115792R2_SourceData_EV+Appendix/Appendix figure S4/S4A western blot/R2/nfkb/western tubulin.tiff]

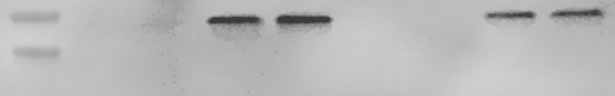

Supplement: Supplementary file 10 — Source Data of EV and Appendix figures [file 44318_2024_35_MOESM10_ESM.zip › EMBOJ-2023-115792R2_SourceData_EV+Appendix/Appendix figure S4/S4A western blot/R2/ikba/western capsid.tiff]

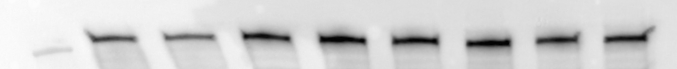

Supplement: Supplementary file 10 — Source Data of EV and Appendix figures [file 44318_2024_35_MOESM10_ESM.zip › EMBOJ-2023-115792R2_SourceData_EV+Appendix/Appendix figure S4/S4A western blot/R2/ikba/western dicer.tiff]

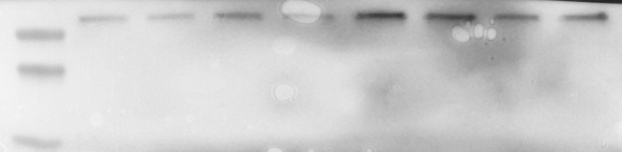

Supplement: Supplementary file 10 — Source Data of EV and Appendix figures [file 44318_2024_35_MOESM10_ESM.zip › EMBOJ-2023-115792R2_SourceData_EV+Appendix/Appendix figure S4/S4A western blot/R2/ikba/western ikba.tiff]

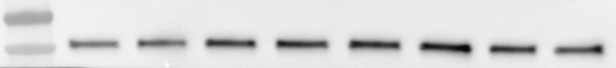

Supplement: Supplementary file 10 — Source Data of EV and Appendix figures [file 44318_2024_35_MOESM10_ESM.zip › EMBOJ-2023-115792R2_SourceData_EV+Appendix/Appendix figure S4/S4A western blot/R2/ikba/western tubulin.tiff]

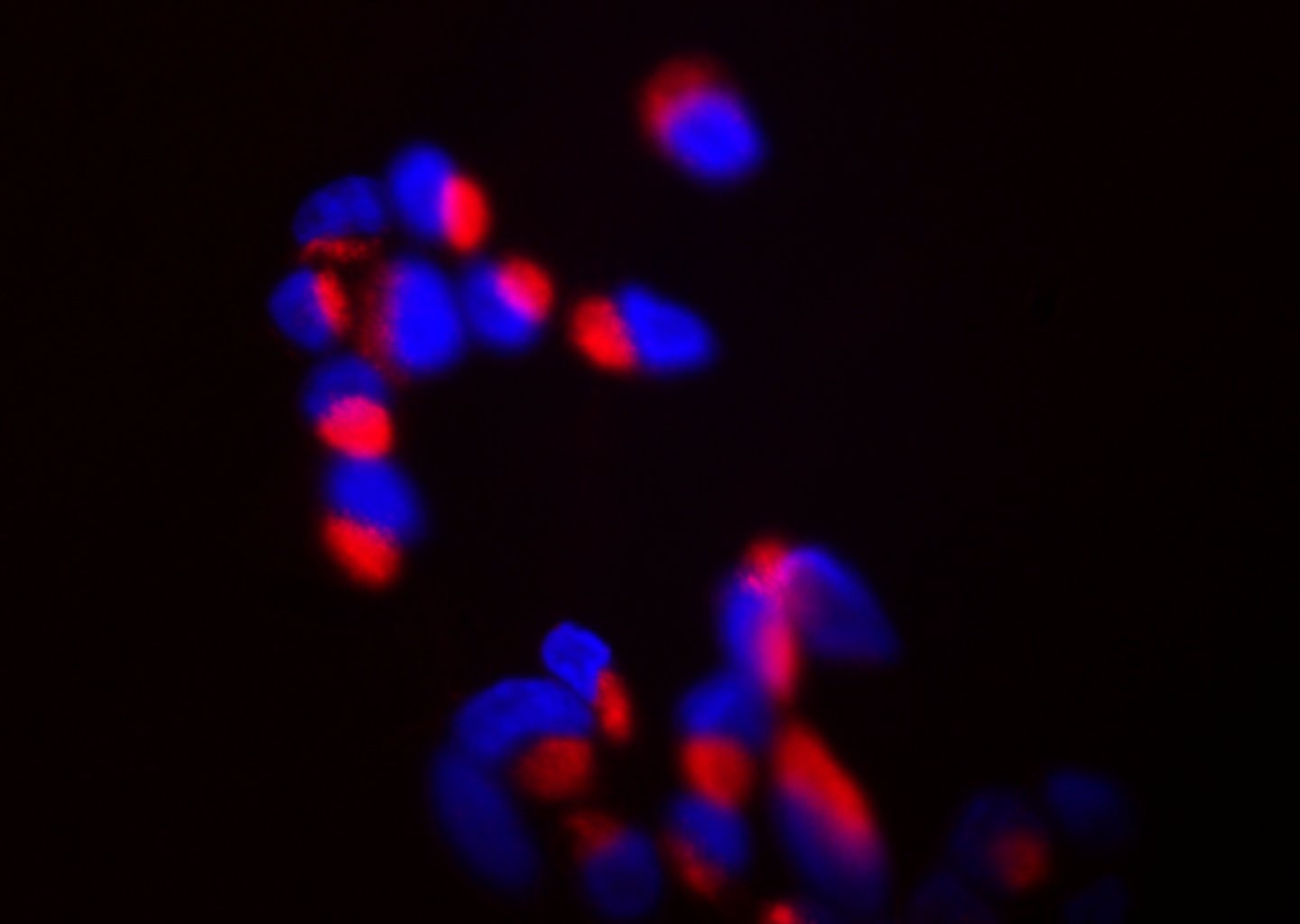

Supplement: Supplementary file 10 — Source Data of EV and Appendix figures [file 44318_2024_35_MOESM10_ESM.zip › EMBOJ-2023-115792R2_SourceData_EV+Appendix/Appendix Figure S2/Appendix_FigS2A microscopy/R1/N1 SARS R1/N1 SARS merge.jpg]

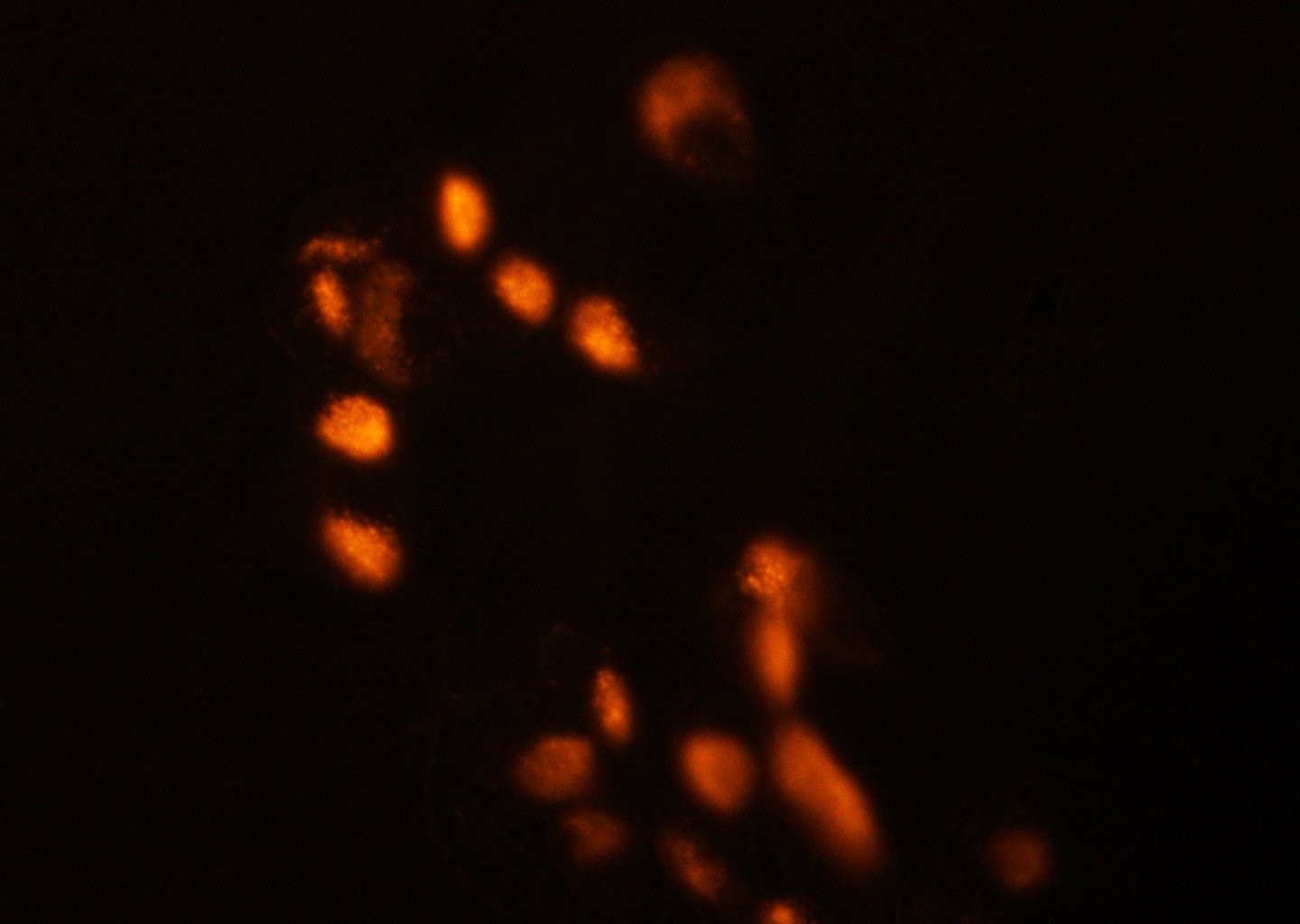

Supplement: Supplementary file 10 — Source Data of EV and Appendix figures [file 44318_2024_35_MOESM10_ESM.zip › EMBOJ-2023-115792R2_SourceData_EV+Appendix/Appendix Figure S2/Appendix_FigS2A microscopy/R1/N1 SARS R1/J2.jpg]

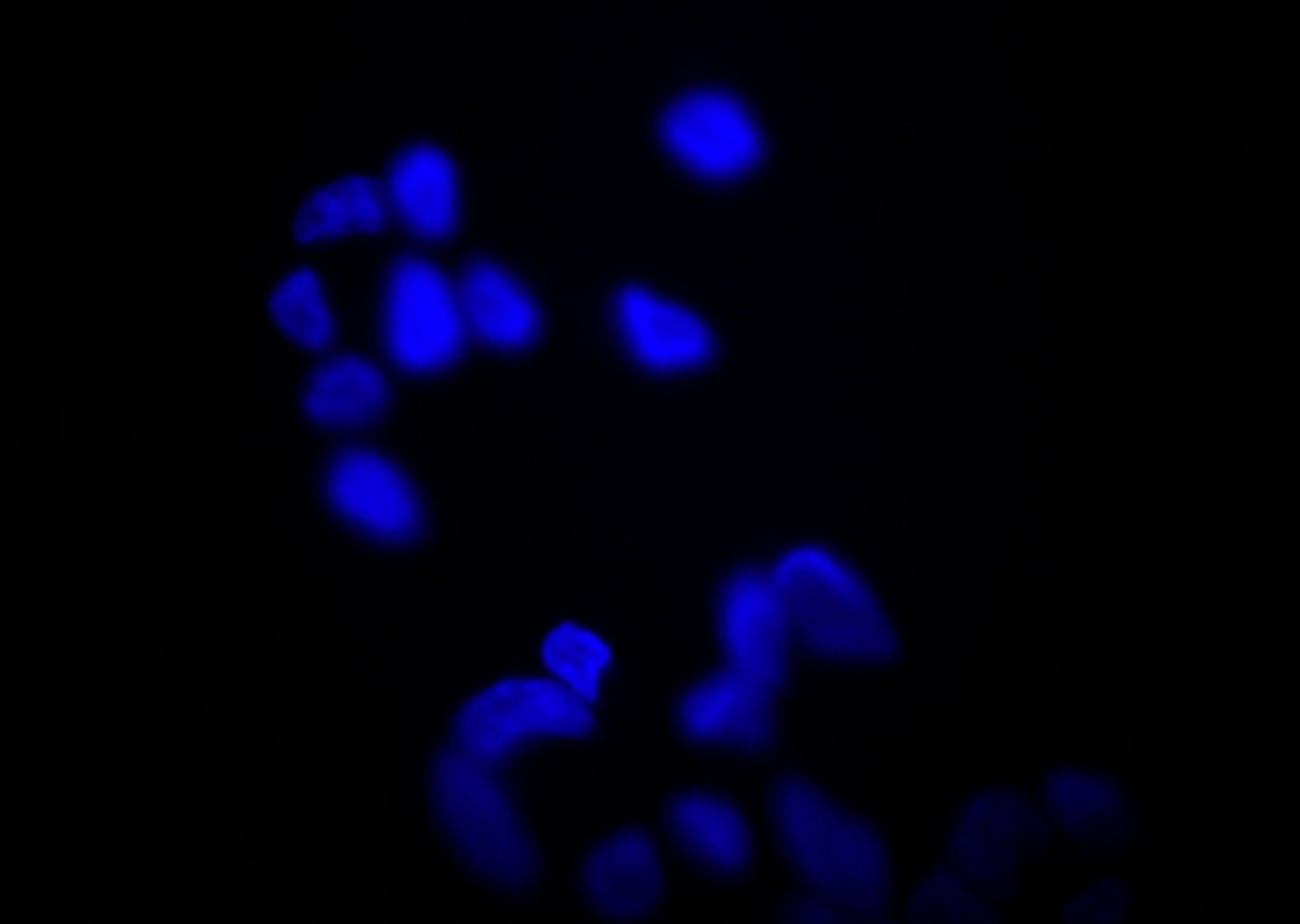

Supplement: Supplementary file 10 — Source Data of EV and Appendix figures [file 44318_2024_35_MOESM10_ESM.zip › EMBOJ-2023-115792R2_SourceData_EV+Appendix/Appendix Figure S2/Appendix_FigS2A microscopy/R1/N1 SARS R1/DAPI.jpg]

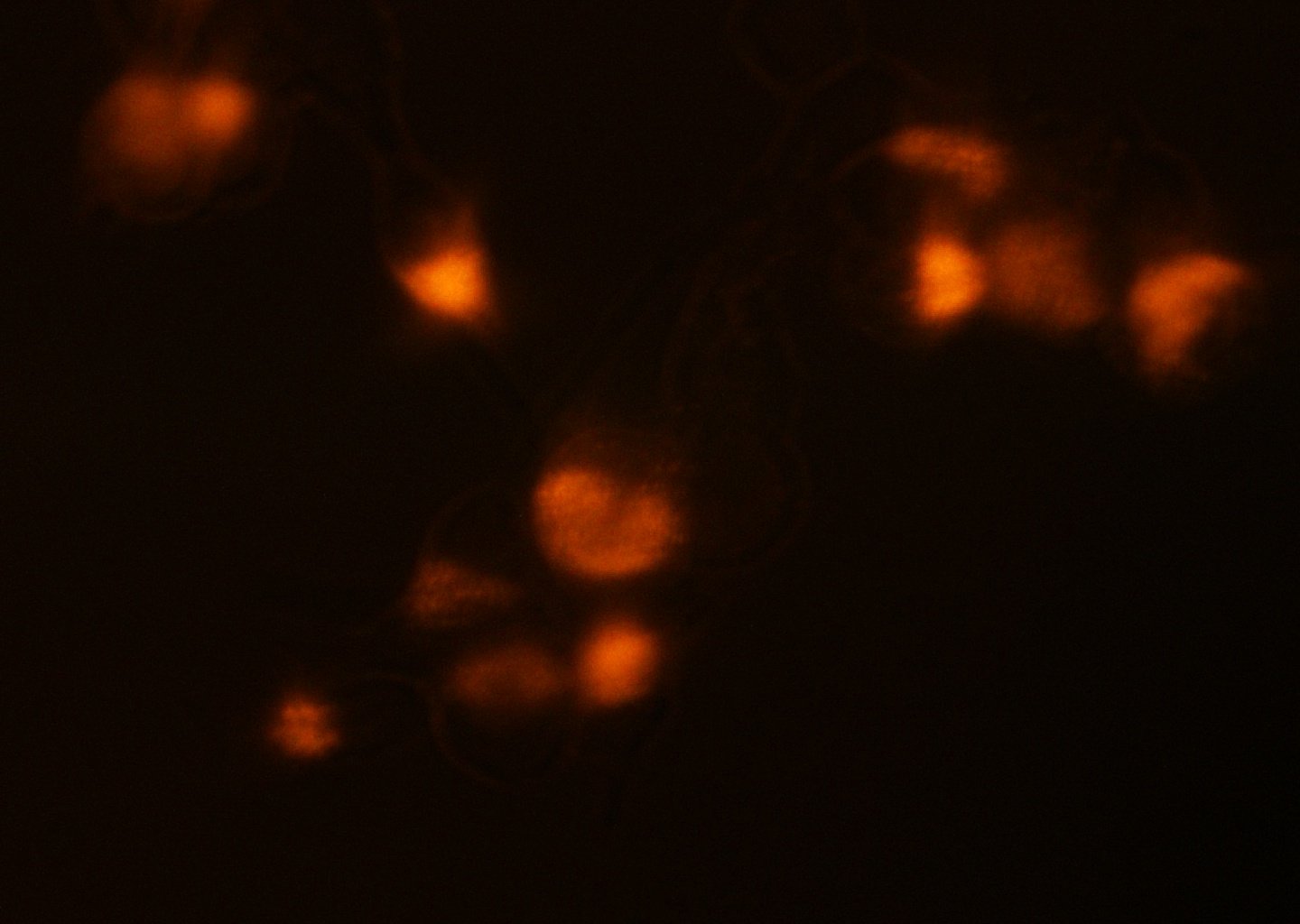

Supplement: Supplementary file 10 — Source Data of EV and Appendix figures [file 44318_2024_35_MOESM10_ESM.zip › EMBOJ-2023-115792R2_SourceData_EV+Appendix/Appendix Figure S2/Appendix_FigS2A microscopy/R1/WT SARS R1/J2.jpg]

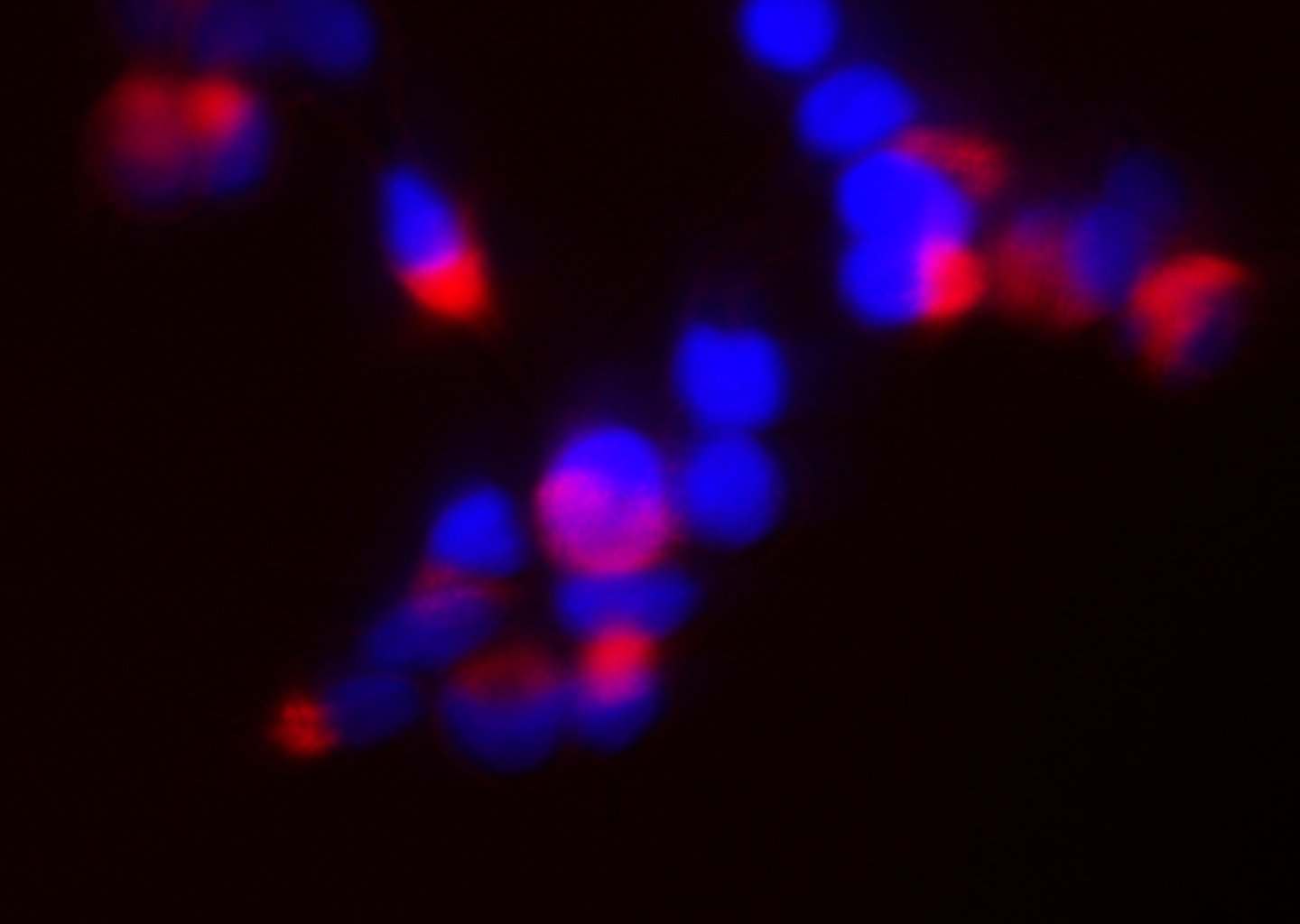

Supplement: Supplementary file 10 — Source Data of EV and Appendix figures [file 44318_2024_35_MOESM10_ESM.zip › EMBOJ-2023-115792R2_SourceData_EV+Appendix/Appendix Figure S2/Appendix_FigS2A microscopy/R1/WT SARS R1/WT SARS merge.jpg]

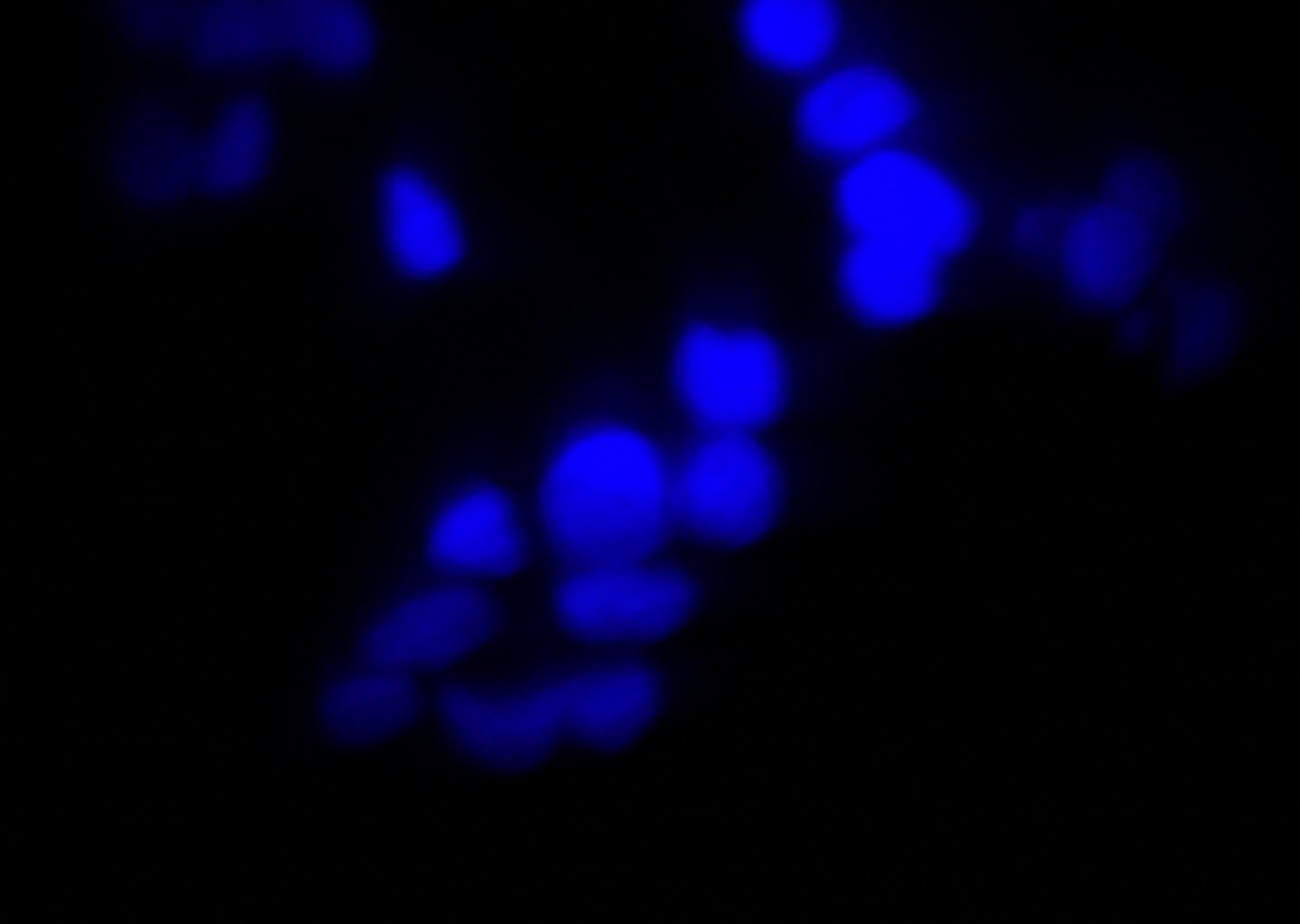

Supplement: Supplementary file 10 — Source Data of EV and Appendix figures [file 44318_2024_35_MOESM10_ESM.zip › EMBOJ-2023-115792R2_SourceData_EV+Appendix/Appendix Figure S2/Appendix_FigS2A microscopy/R1/WT SARS R1/DAPI.jpg]

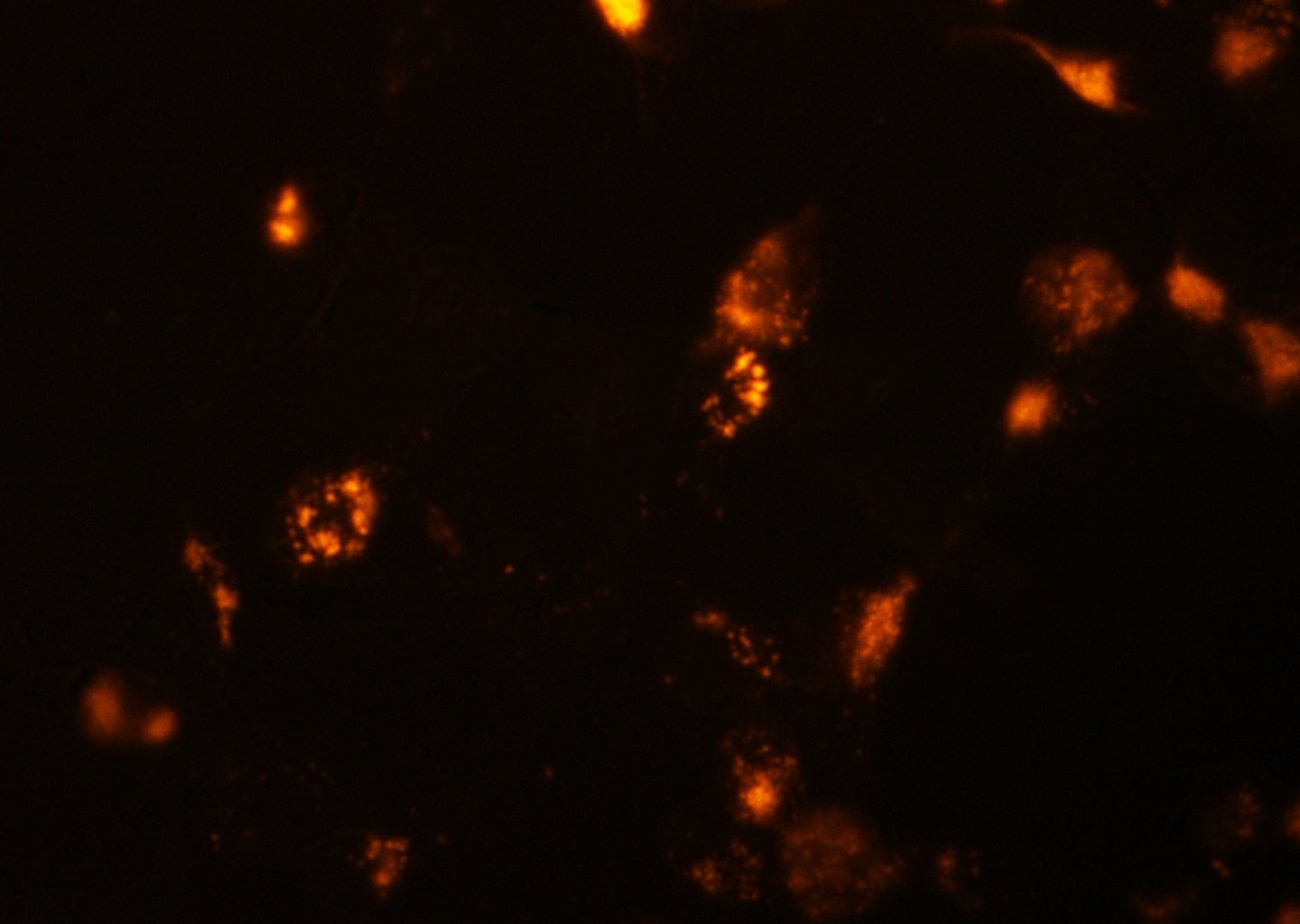

Supplement: Supplementary file 10 — Source Data of EV and Appendix figures [file 44318_2024_35_MOESM10_ESM.zip › EMBOJ-2023-115792R2_SourceData_EV+Appendix/Appendix Figure S2/Appendix_FigS2A microscopy/R3/N1 SARS R3/J2.jpg]

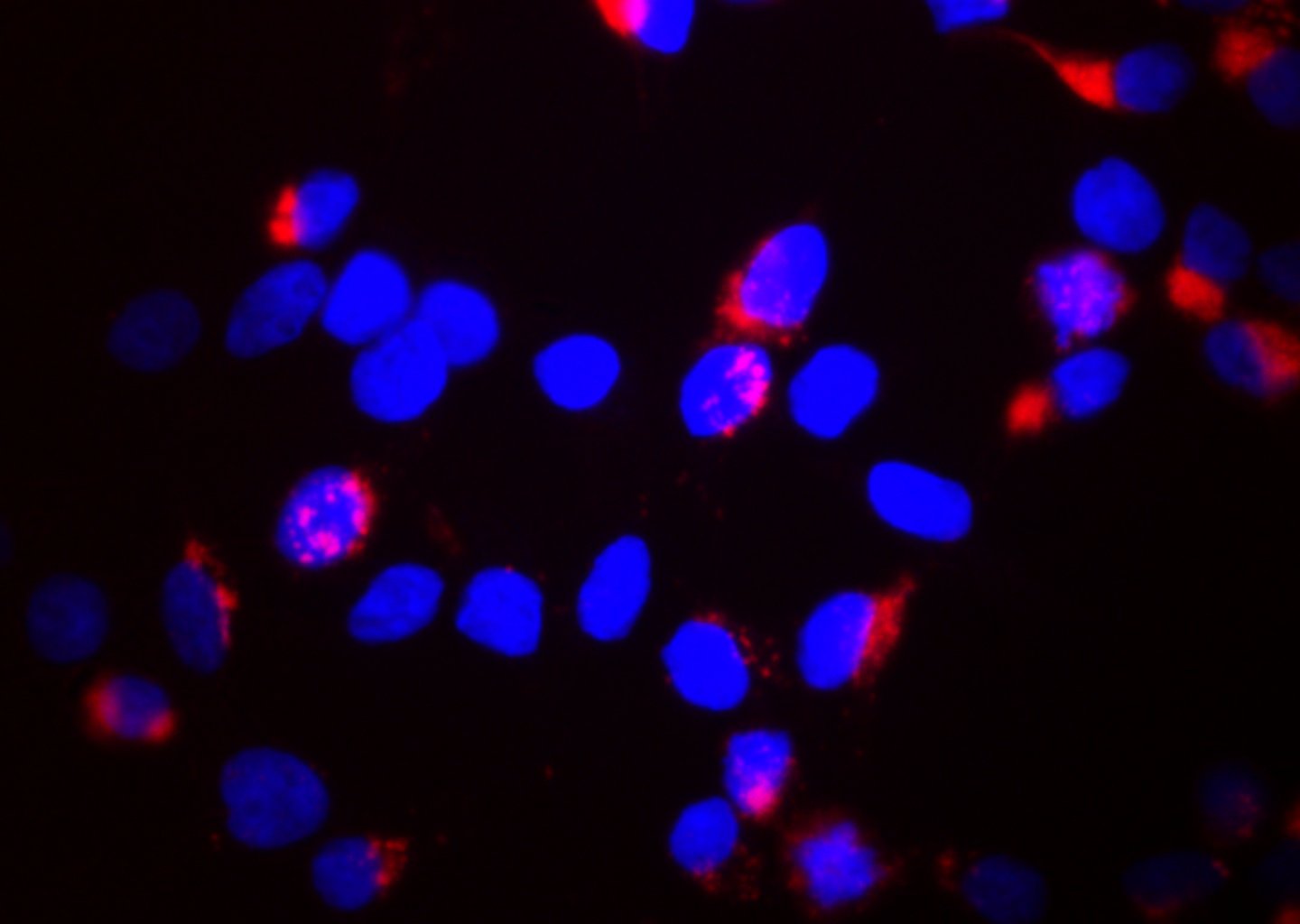

Supplement: Supplementary file 10 — Source Data of EV and Appendix figures [file 44318_2024_35_MOESM10_ESM.zip › EMBOJ-2023-115792R2_SourceData_EV+Appendix/Appendix Figure S2/Appendix_FigS2A microscopy/R3/N1 SARS R3/N1 SARS R3 merge.jpg]

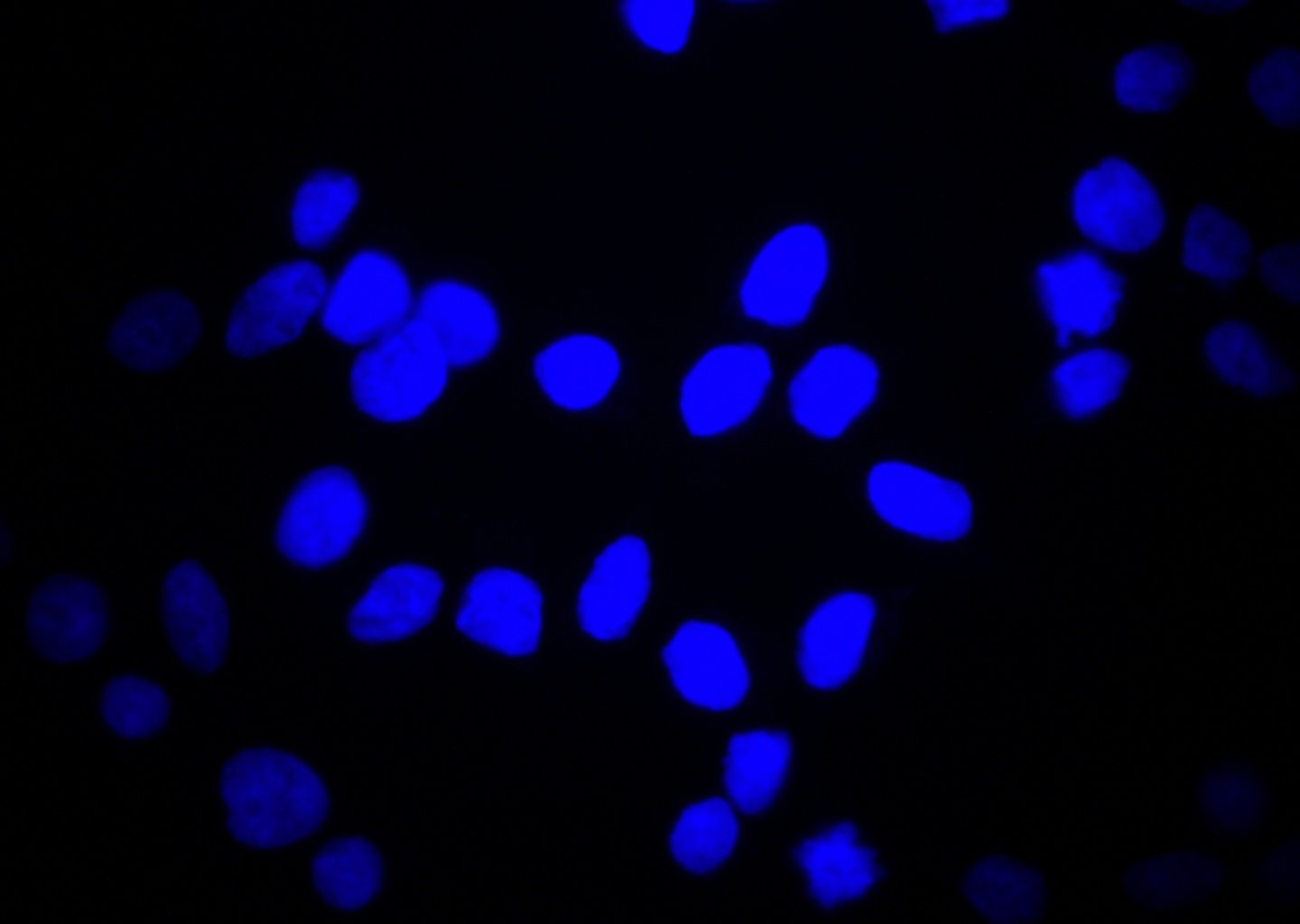

Supplement: Supplementary file 10 — Source Data of EV and Appendix figures [file 44318_2024_35_MOESM10_ESM.zip › EMBOJ-2023-115792R2_SourceData_EV+Appendix/Appendix Figure S2/Appendix_FigS2A microscopy/R3/N1 SARS R3/DAPI.jpg]
