# Supplementary figures and images for: The helicase domain of human Dicer prevents RNAi-independent activation of antiviral and inflammatory pathways (part 4 of 5)
Source: EMBO J. 2024 Jan 29;43(5):7. doi: 10.1038/s44318-024-00035-2 (PMC10907635; doi:10.1038/s44318-024-00035-2)

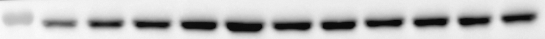

Supplement: Supplementary file 10 — Source Data of EV and Appendix figures [file 44318_2024_35_MOESM10_ESM.zip › EMBOJ-2023-115792R2_SourceData_EV+Appendix/FigEV1/FigEV1D western blot/R3/western tubulin.tiff]

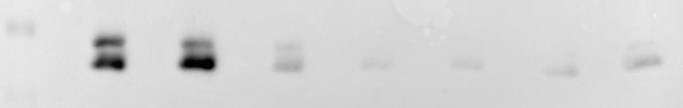

Supplement: Supplementary file 10 — Source Data of EV and Appendix figures [file 44318_2024_35_MOESM10_ESM.zip › EMBOJ-2023-115792R2_SourceData_EV+Appendix/FigEV1/FigEV1D western blot/R2/western gfp.tiff]

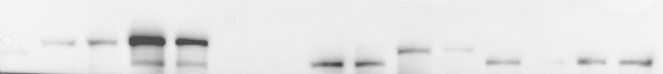

Supplement: Supplementary file 10 — Source Data of EV and Appendix figures [file 44318_2024_35_MOESM10_ESM.zip › EMBOJ-2023-115792R2_SourceData_EV+Appendix/FigEV1/FigEV1D western blot/R2/western dicer.tiff]

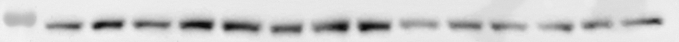

Supplement: Supplementary file 10 — Source Data of EV and Appendix figures [file 44318_2024_35_MOESM10_ESM.zip › EMBOJ-2023-115792R2_SourceData_EV+Appendix/FigEV1/FigEV1D western blot/R2/western tubulin.tiff]

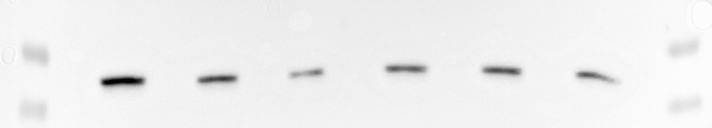

Supplement: Supplementary file 10 — Source Data of EV and Appendix figures [file 44318_2024_35_MOESM10_ESM.zip › EMBOJ-2023-115792R2_SourceData_EV+Appendix/FigEV3/FigEV3A western blot/R1/wester capsid.tiff]

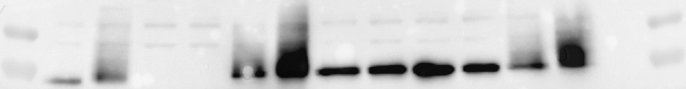

Supplement: Supplementary file 10 — Source Data of EV and Appendix figures [file 44318_2024_35_MOESM10_ESM.zip › EMBOJ-2023-115792R2_SourceData_EV+Appendix/FigEV3/FigEV3A western blot/R1/western PKR.tiff]

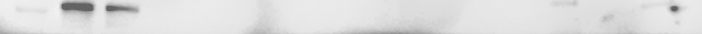

Supplement: Supplementary file 10 — Source Data of EV and Appendix figures [file 44318_2024_35_MOESM10_ESM.zip › EMBOJ-2023-115792R2_SourceData_EV+Appendix/FigEV3/FigEV3A western blot/R1/western dicer.tiff]

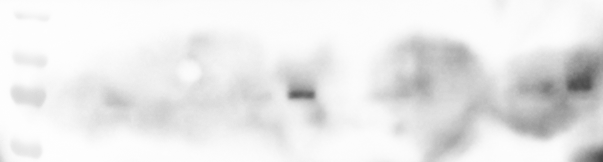

Supplement: Supplementary file 10 — Source Data of EV and Appendix figures [file 44318_2024_35_MOESM10_ESM.zip › EMBOJ-2023-115792R2_SourceData_EV+Appendix/FigEV3/FigEV3A western blot/R1/western p-PKR.tiff]

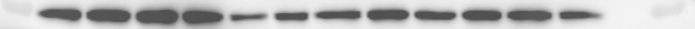

Supplement: Supplementary file 10 — Source Data of EV and Appendix figures [file 44318_2024_35_MOESM10_ESM.zip › EMBOJ-2023-115792R2_SourceData_EV+Appendix/FigEV3/FigEV3A western blot/R1/western tubulin.tiff]

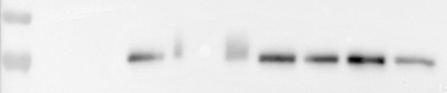

Supplement: Supplementary file 10 — Source Data of EV and Appendix figures [file 44318_2024_35_MOESM10_ESM.zip › EMBOJ-2023-115792R2_SourceData_EV+Appendix/FigEV3/FigEV3A western blot/R3/western PKR.tiff]

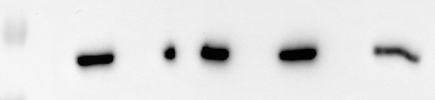

Supplement: Supplementary file 10 — Source Data of EV and Appendix figures [file 44318_2024_35_MOESM10_ESM.zip › EMBOJ-2023-115792R2_SourceData_EV+Appendix/FigEV3/FigEV3A western blot/R3/western capsid.tiff]

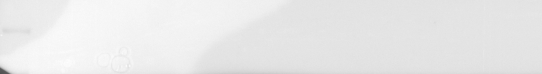

Supplement: Supplementary file 10 — Source Data of EV and Appendix figures [file 44318_2024_35_MOESM10_ESM.zip › EMBOJ-2023-115792R2_SourceData_EV+Appendix/FigEV3/FigEV3A western blot/R3/western dicer.tiff]

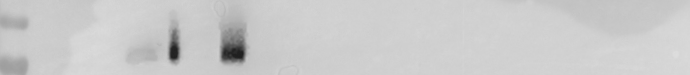

Supplement: Supplementary file 10 — Source Data of EV and Appendix figures [file 44318_2024_35_MOESM10_ESM.zip › EMBOJ-2023-115792R2_SourceData_EV+Appendix/FigEV3/FigEV3A western blot/R3/western p-PKR.tiff]

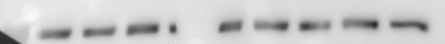

Supplement: Supplementary file 10 — Source Data of EV and Appendix figures [file 44318_2024_35_MOESM10_ESM.zip › EMBOJ-2023-115792R2_SourceData_EV+Appendix/FigEV3/FigEV3A western blot/R3/western tubulin.tiff]

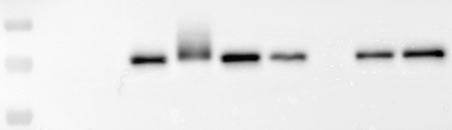

Supplement: Supplementary file 10 — Source Data of EV and Appendix figures [file 44318_2024_35_MOESM10_ESM.zip › EMBOJ-2023-115792R2_SourceData_EV+Appendix/FigEV3/FigEV3A western blot/R2/western PKR.tiff]

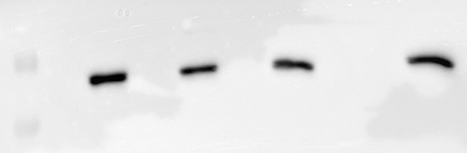

Supplement: Supplementary file 10 — Source Data of EV and Appendix figures [file 44318_2024_35_MOESM10_ESM.zip › EMBOJ-2023-115792R2_SourceData_EV+Appendix/FigEV3/FigEV3A western blot/R2/western capsid.tiff]

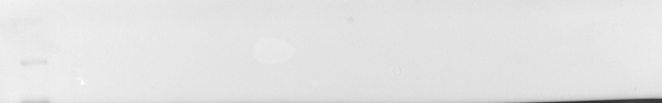

Supplement: Supplementary file 10 — Source Data of EV and Appendix figures [file 44318_2024_35_MOESM10_ESM.zip › EMBOJ-2023-115792R2_SourceData_EV+Appendix/FigEV3/FigEV3A western blot/R2/western dicer.tiff]

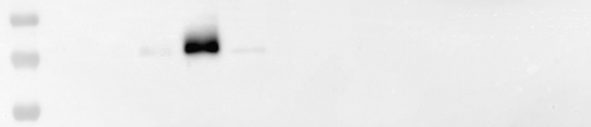

Supplement: Supplementary file 10 — Source Data of EV and Appendix figures [file 44318_2024_35_MOESM10_ESM.zip › EMBOJ-2023-115792R2_SourceData_EV+Appendix/FigEV3/FigEV3A western blot/R2/western p-PKR.tiff]

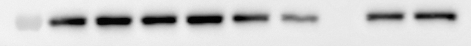

Supplement: Supplementary file 10 — Source Data of EV and Appendix figures [file 44318_2024_35_MOESM10_ESM.zip › EMBOJ-2023-115792R2_SourceData_EV+Appendix/FigEV3/FigEV3A western blot/R2/western tubulin.tiff]

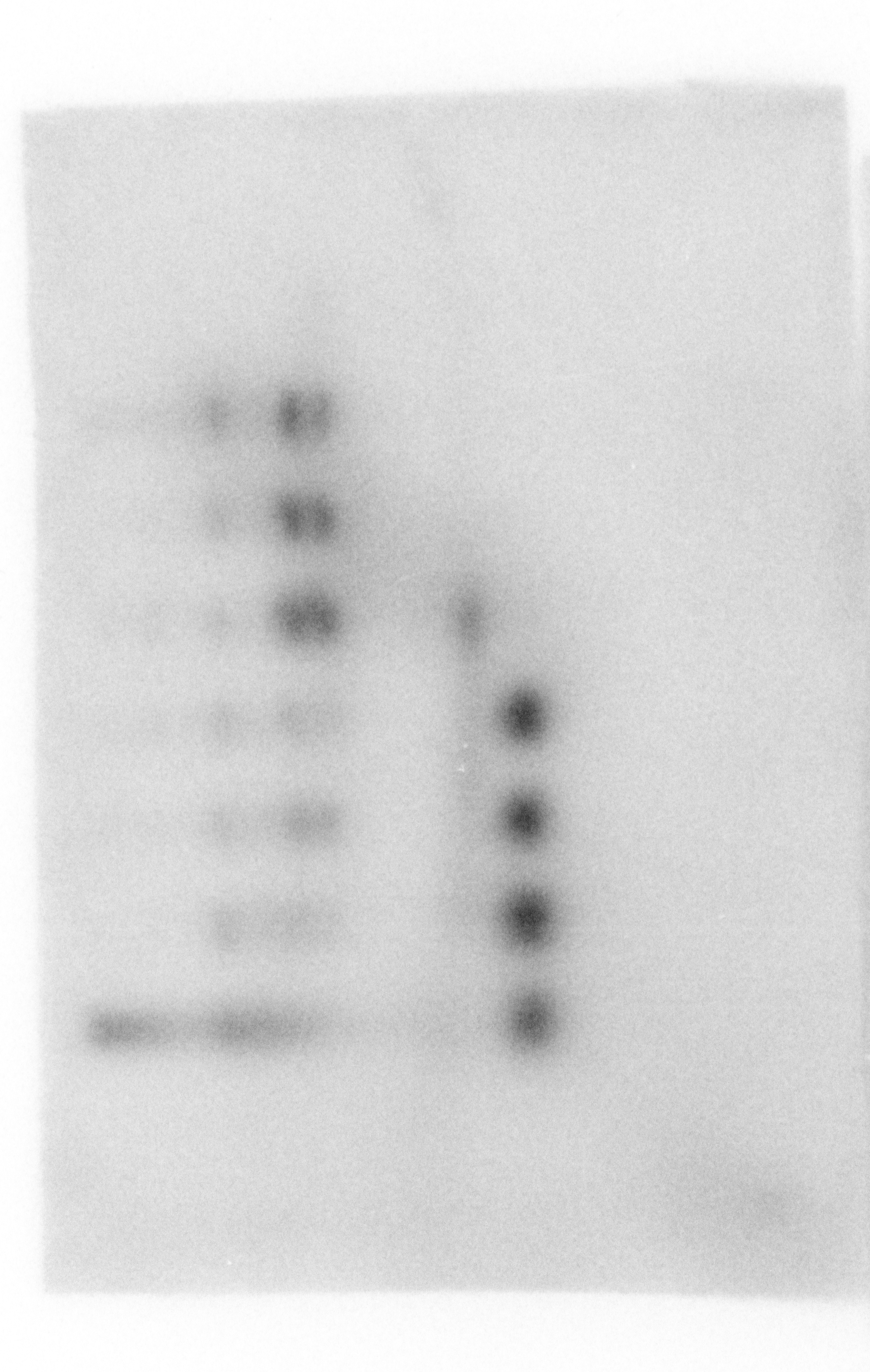

Supplement: Supplementary file 10 — Source Data of EV and Appendix figures [file 44318_2024_35_MOESM10_ESM.zip › EMBOJ-2023-115792R2_SourceData_EV+Appendix/FigEV2/FigEV2D northern blot/R1/northern miR16.tiff]

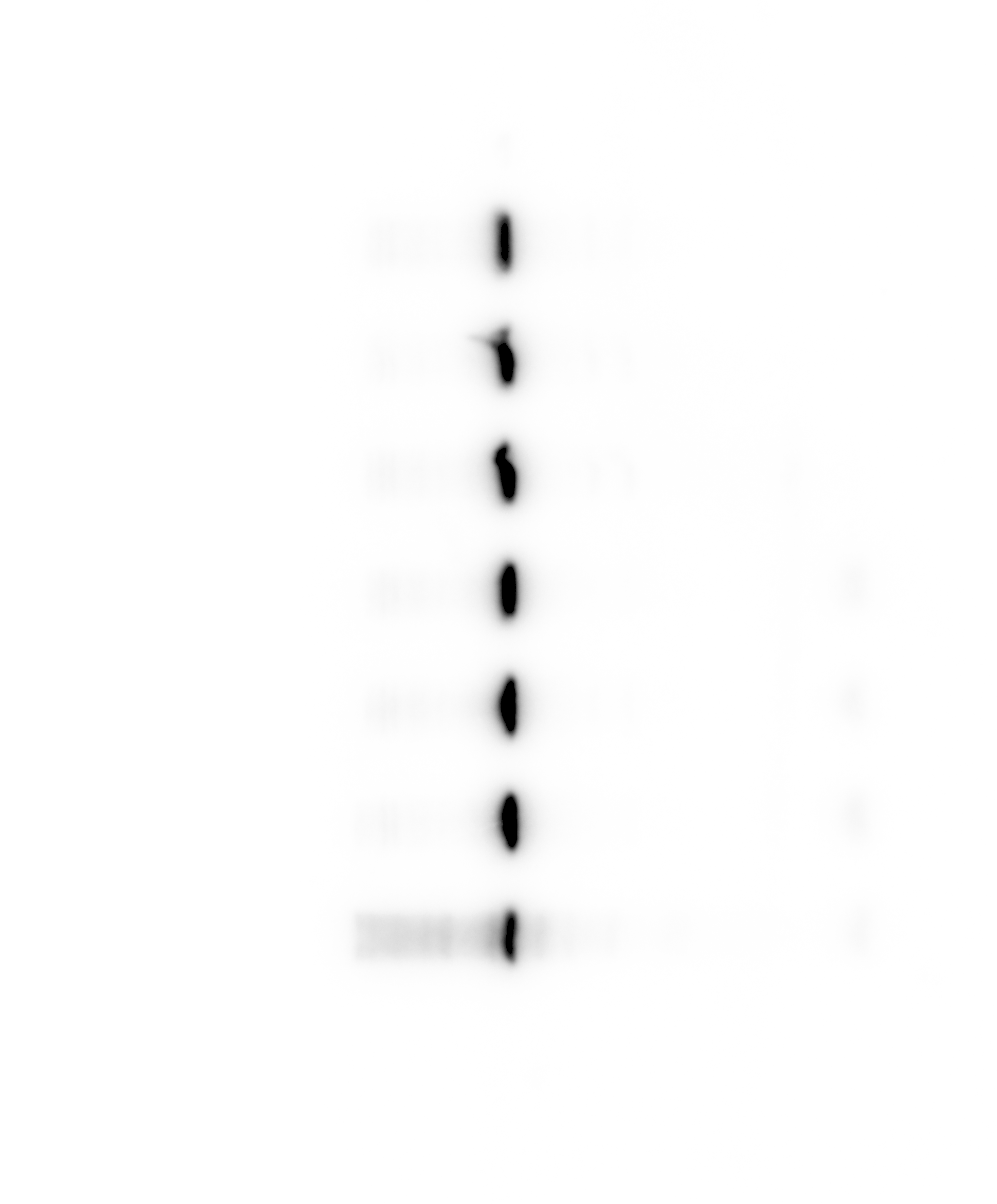

Supplement: Supplementary file 10 — Source Data of EV and Appendix figures [file 44318_2024_35_MOESM10_ESM.zip › EMBOJ-2023-115792R2_SourceData_EV+Appendix/FigEV2/FigEV2D northern blot/R1/northern U6.tiff]

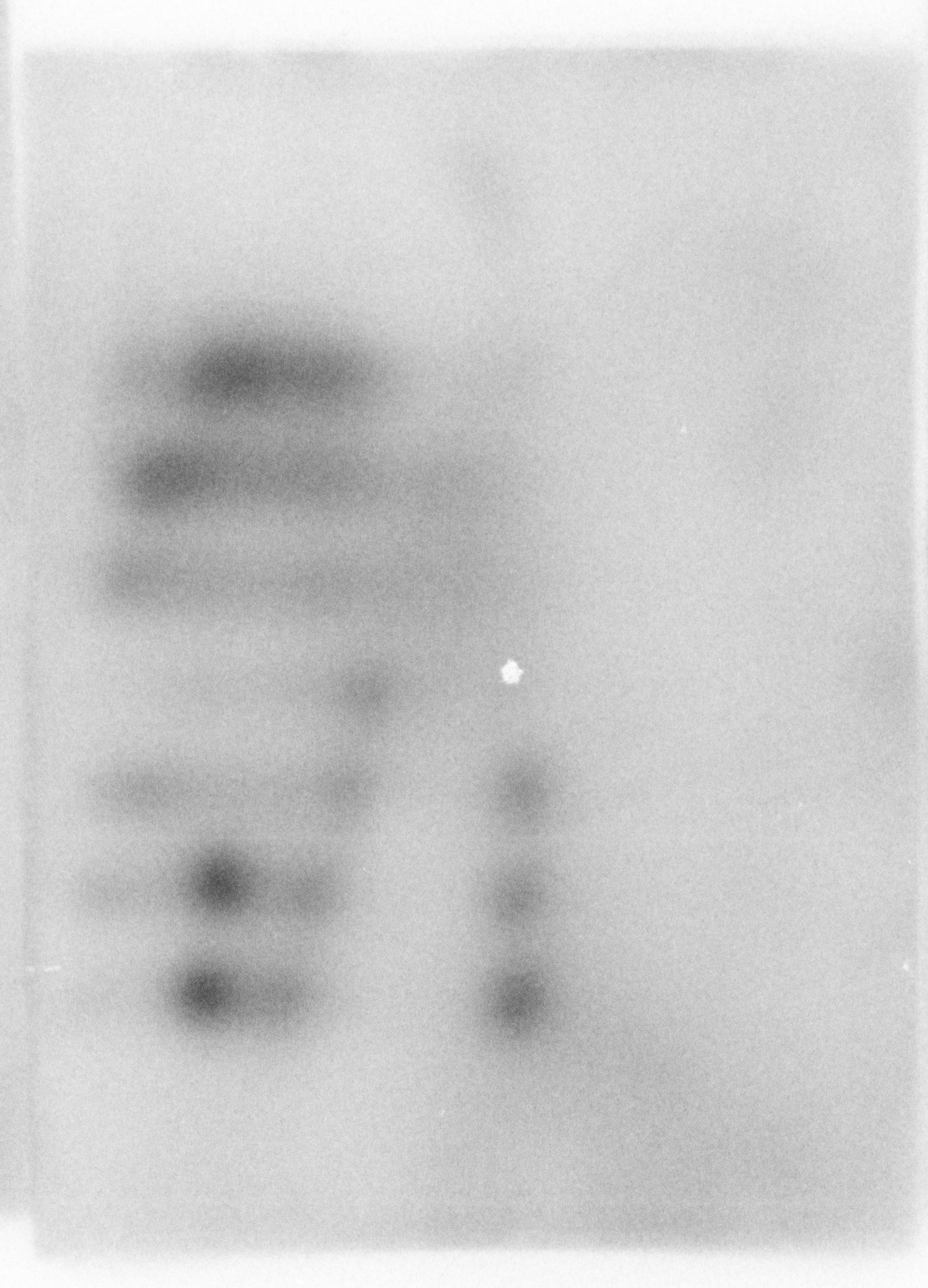

Supplement: Supplementary file 10 — Source Data of EV and Appendix figures [file 44318_2024_35_MOESM10_ESM.zip › EMBOJ-2023-115792R2_SourceData_EV+Appendix/FigEV2/FigEV2D northern blot/R2/northern miR16.tiff]

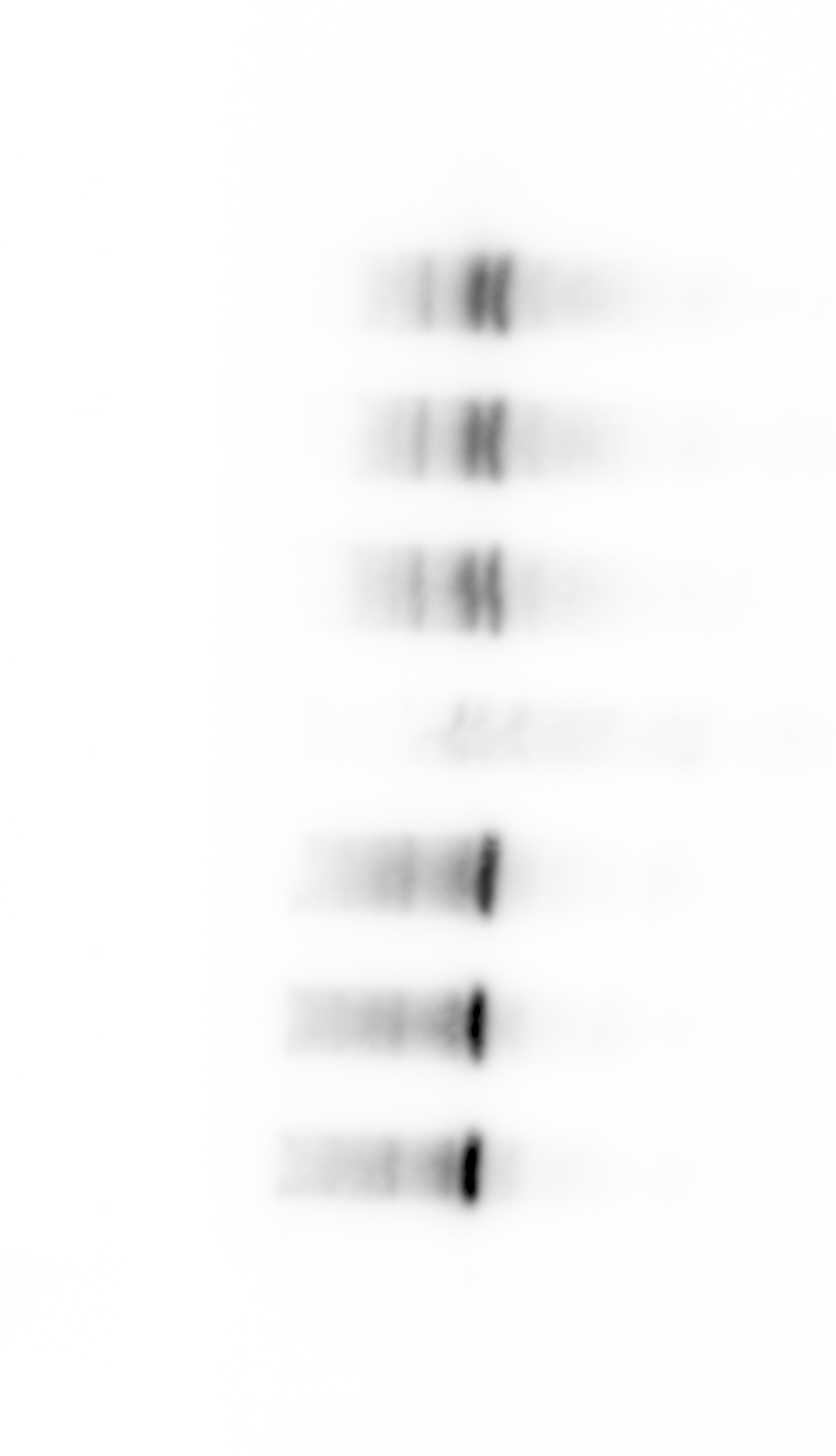

Supplement: Supplementary file 10 — Source Data of EV and Appendix figures [file 44318_2024_35_MOESM10_ESM.zip › EMBOJ-2023-115792R2_SourceData_EV+Appendix/FigEV2/FigEV2D northern blot/R2/northern U6.tiff]

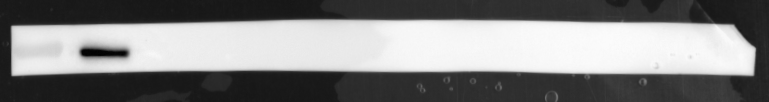

Supplement: Supplementary file 10 — Source Data of EV and Appendix figures [file 44318_2024_35_MOESM10_ESM.zip › EMBOJ-2023-115792R2_SourceData_EV+Appendix/FigEV2/FigEV2C western blot/R1/western PKR .tiff]

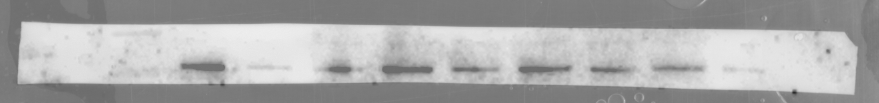

Supplement: Supplementary file 10 — Source Data of EV and Appendix figures [file 44318_2024_35_MOESM10_ESM.zip › EMBOJ-2023-115792R2_SourceData_EV+Appendix/FigEV2/FigEV2C western blot/R1/western ago.tiff]

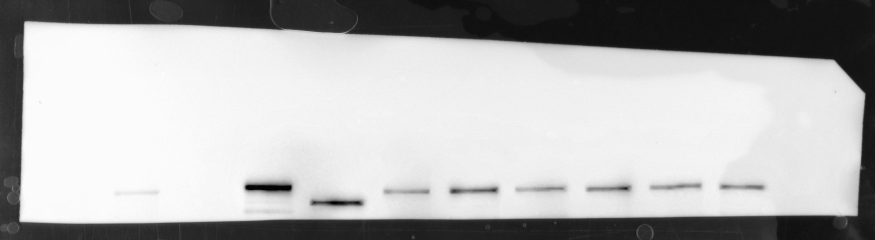

Supplement: Supplementary file 10 — Source Data of EV and Appendix figures [file 44318_2024_35_MOESM10_ESM.zip › EMBOJ-2023-115792R2_SourceData_EV+Appendix/FigEV2/FigEV2C western blot/R1/western HA.tiff]

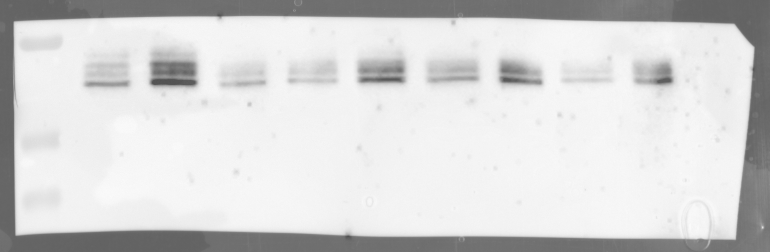

Supplement: Supplementary file 10 — Source Data of EV and Appendix figures [file 44318_2024_35_MOESM10_ESM.zip › EMBOJ-2023-115792R2_SourceData_EV+Appendix/FigEV2/FigEV2C western blot/R1/western TRBP.tiff]

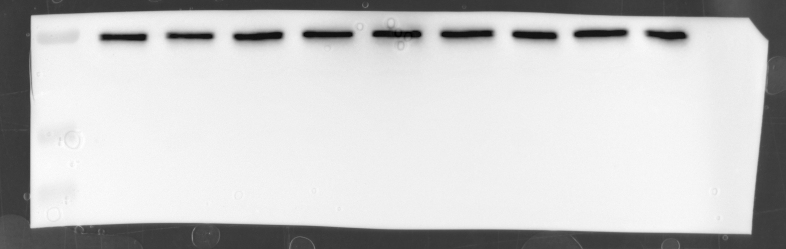

Supplement: Supplementary file 10 — Source Data of EV and Appendix figures [file 44318_2024_35_MOESM10_ESM.zip › EMBOJ-2023-115792R2_SourceData_EV+Appendix/FigEV2/FigEV2C western blot/R1/western tubulin.tiff]

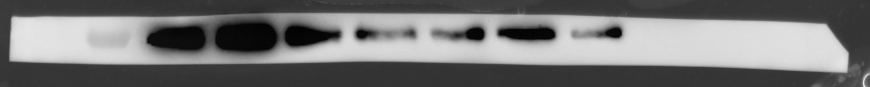

Supplement: Supplementary file 10 — Source Data of EV and Appendix figures [file 44318_2024_35_MOESM10_ESM.zip › EMBOJ-2023-115792R2_SourceData_EV+Appendix/FigEV2/FigEV2A western blot/R2 /western PKR.tiff]

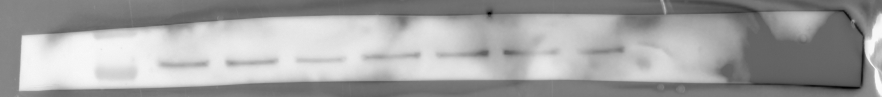

Supplement: Supplementary file 10 — Source Data of EV and Appendix figures [file 44318_2024_35_MOESM10_ESM.zip › EMBOJ-2023-115792R2_SourceData_EV+Appendix/FigEV2/FigEV2A western blot/R2 /western ago.tiff]

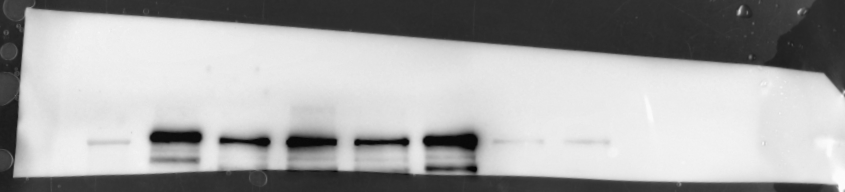

Supplement: Supplementary file 10 — Source Data of EV and Appendix figures [file 44318_2024_35_MOESM10_ESM.zip › EMBOJ-2023-115792R2_SourceData_EV+Appendix/FigEV2/FigEV2A western blot/R2 /western HA.tiff]

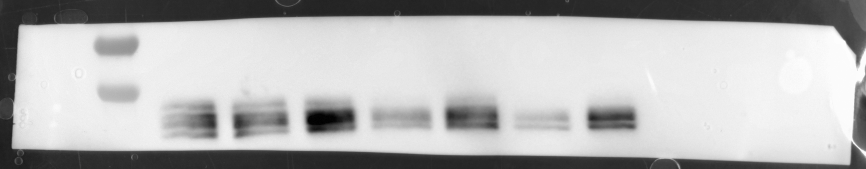

Supplement: Supplementary file 10 — Source Data of EV and Appendix figures [file 44318_2024_35_MOESM10_ESM.zip › EMBOJ-2023-115792R2_SourceData_EV+Appendix/FigEV2/FigEV2A western blot/R2 /western TRBP.tiff]

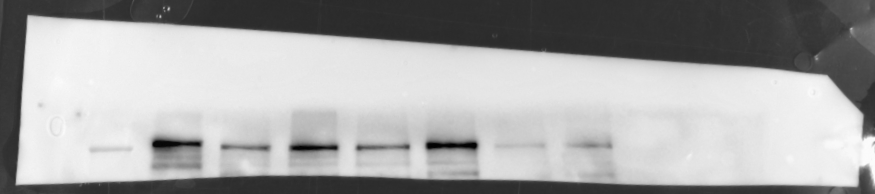

Supplement: Supplementary file 10 — Source Data of EV and Appendix figures [file 44318_2024_35_MOESM10_ESM.zip › EMBOJ-2023-115792R2_SourceData_EV+Appendix/FigEV2/FigEV2A western blot/R2 /western dicer.tiff]

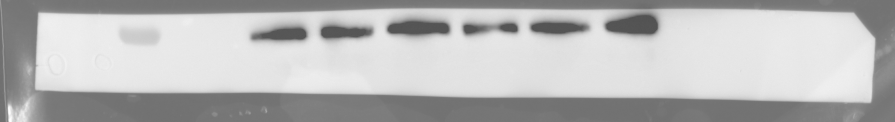

Supplement: Supplementary file 10 — Source Data of EV and Appendix figures [file 44318_2024_35_MOESM10_ESM.zip › EMBOJ-2023-115792R2_SourceData_EV+Appendix/FigEV2/FigEV2A western blot/R2 /western tubulin.tiff]

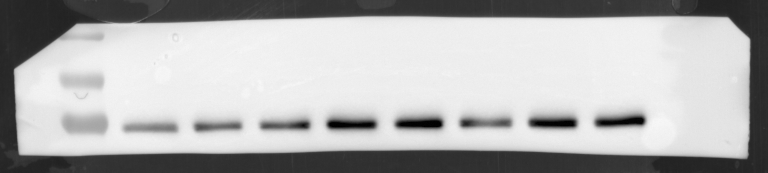

Supplement: Supplementary file 10 — Source Data of EV and Appendix figures [file 44318_2024_35_MOESM10_ESM.zip › EMBOJ-2023-115792R2_SourceData_EV+Appendix/FigEV2/FigEV2A western blot/R1/western PKR.tiff]

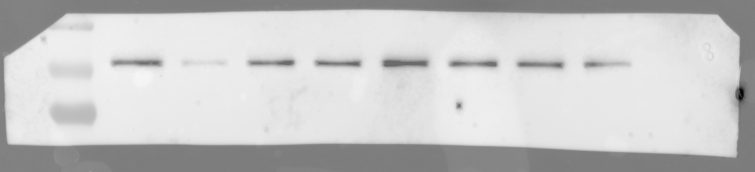

Supplement: Supplementary file 10 — Source Data of EV and Appendix figures [file 44318_2024_35_MOESM10_ESM.zip › EMBOJ-2023-115792R2_SourceData_EV+Appendix/FigEV2/FigEV2A western blot/R1/western AGO.tiff]

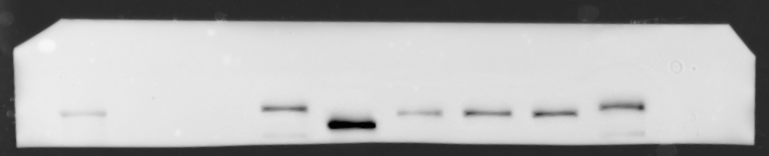

Supplement: Supplementary file 10 — Source Data of EV and Appendix figures [file 44318_2024_35_MOESM10_ESM.zip › EMBOJ-2023-115792R2_SourceData_EV+Appendix/FigEV2/FigEV2A western blot/R1/western HA.tiff]

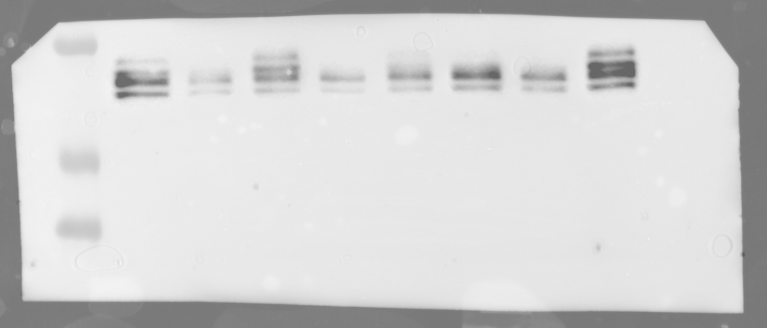

Supplement: Supplementary file 10 — Source Data of EV and Appendix figures [file 44318_2024_35_MOESM10_ESM.zip › EMBOJ-2023-115792R2_SourceData_EV+Appendix/FigEV2/FigEV2A western blot/R1/western TRBP.tiff]

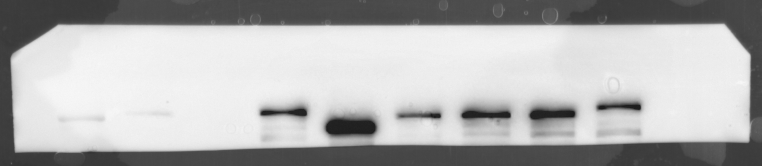

Supplement: Supplementary file 10 — Source Data of EV and Appendix figures [file 44318_2024_35_MOESM10_ESM.zip › EMBOJ-2023-115792R2_SourceData_EV+Appendix/FigEV2/FigEV2A western blot/R1/western dicer.tiff]

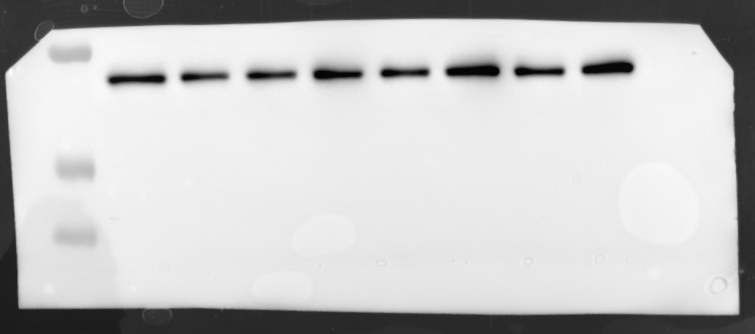

Supplement: Supplementary file 10 — Source Data of EV and Appendix figures [file 44318_2024_35_MOESM10_ESM.zip › EMBOJ-2023-115792R2_SourceData_EV+Appendix/FigEV2/FigEV2A western blot/R1/western tubulin.tiff]

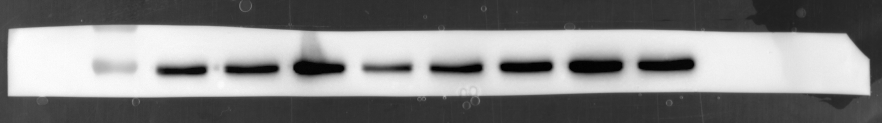

Supplement: Supplementary file 10 — Source Data of EV and Appendix figures [file 44318_2024_35_MOESM10_ESM.zip › EMBOJ-2023-115792R2_SourceData_EV+Appendix/FigEV2/FigEV2A western blot/R3 /western PKR.tiff]

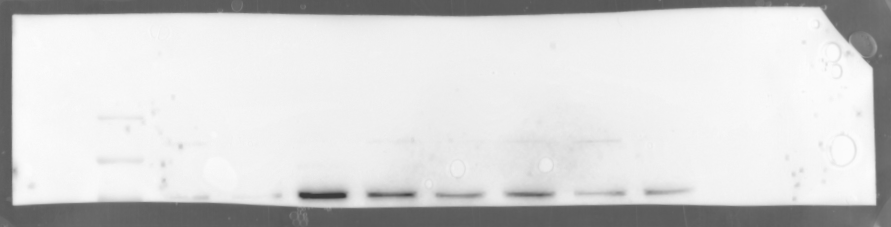

Supplement: Supplementary file 10 — Source Data of EV and Appendix figures [file 44318_2024_35_MOESM10_ESM.zip › EMBOJ-2023-115792R2_SourceData_EV+Appendix/FigEV2/FigEV2A western blot/R3 /western ago.tiff]

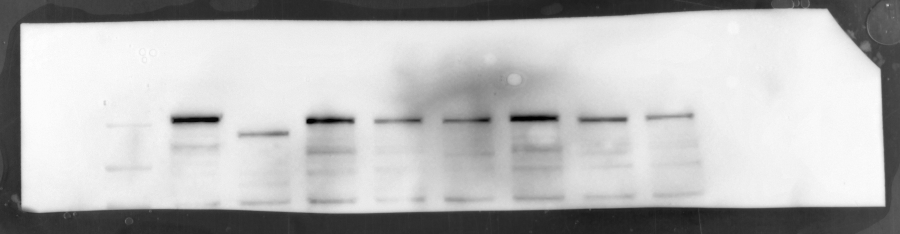

Supplement: Supplementary file 10 — Source Data of EV and Appendix figures [file 44318_2024_35_MOESM10_ESM.zip › EMBOJ-2023-115792R2_SourceData_EV+Appendix/FigEV2/FigEV2A western blot/R3 /western HA.tiff]

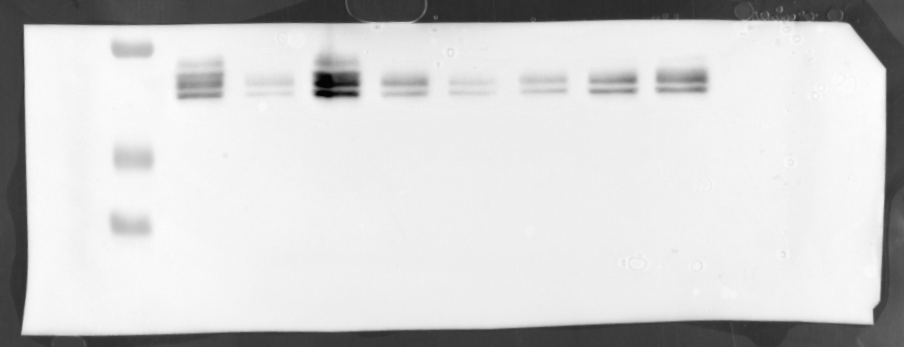

Supplement: Supplementary file 10 — Source Data of EV and Appendix figures [file 44318_2024_35_MOESM10_ESM.zip › EMBOJ-2023-115792R2_SourceData_EV+Appendix/FigEV2/FigEV2A western blot/R3 /western TRBP.tiff]

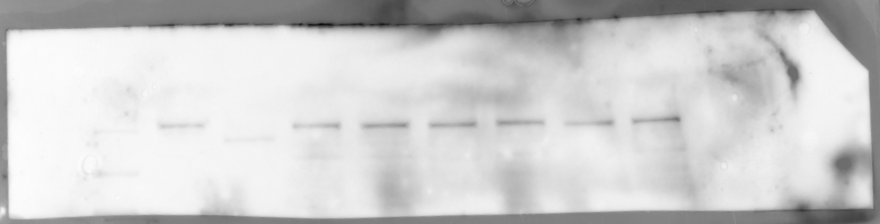

Supplement: Supplementary file 10 — Source Data of EV and Appendix figures [file 44318_2024_35_MOESM10_ESM.zip › EMBOJ-2023-115792R2_SourceData_EV+Appendix/FigEV2/FigEV2A western blot/R3 /western dicer.tiff]

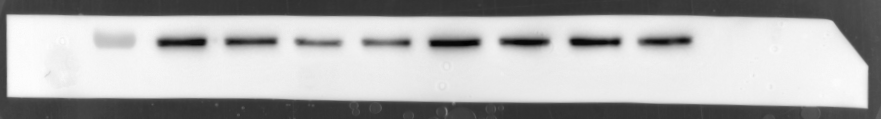

Supplement: Supplementary file 10 — Source Data of EV and Appendix figures [file 44318_2024_35_MOESM10_ESM.zip › EMBOJ-2023-115792R2_SourceData_EV+Appendix/FigEV2/FigEV2A western blot/R3 /western tubulin.tiff]

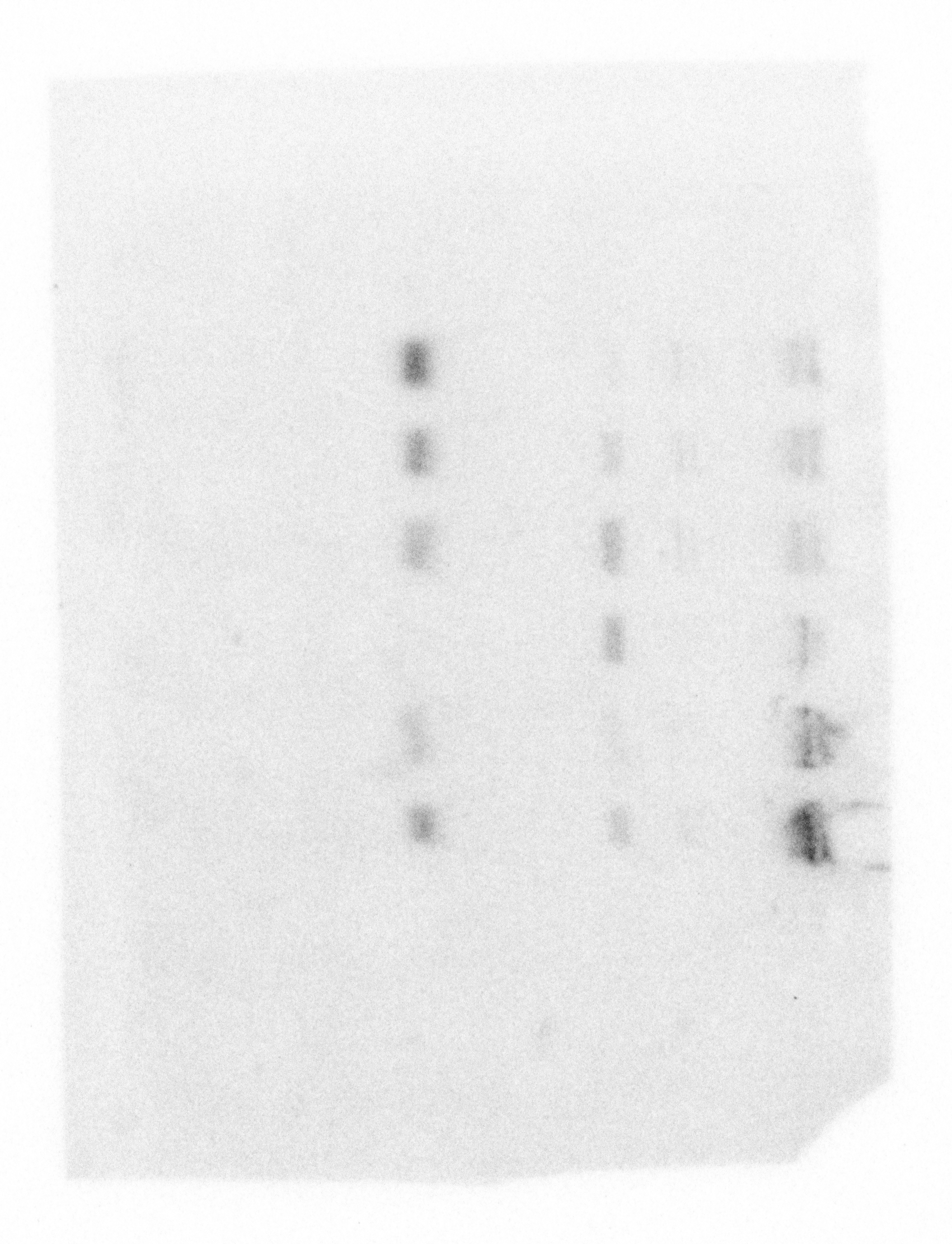

Supplement: Supplementary file 10 — Source Data of EV and Appendix figures [file 44318_2024_35_MOESM10_ESM.zip › EMBOJ-2023-115792R2_SourceData_EV+Appendix/FigEV2/FigEV2B northern blot/R1 dHEL1, 2i and 2/northern miR16 dhel2i 2.tiff]

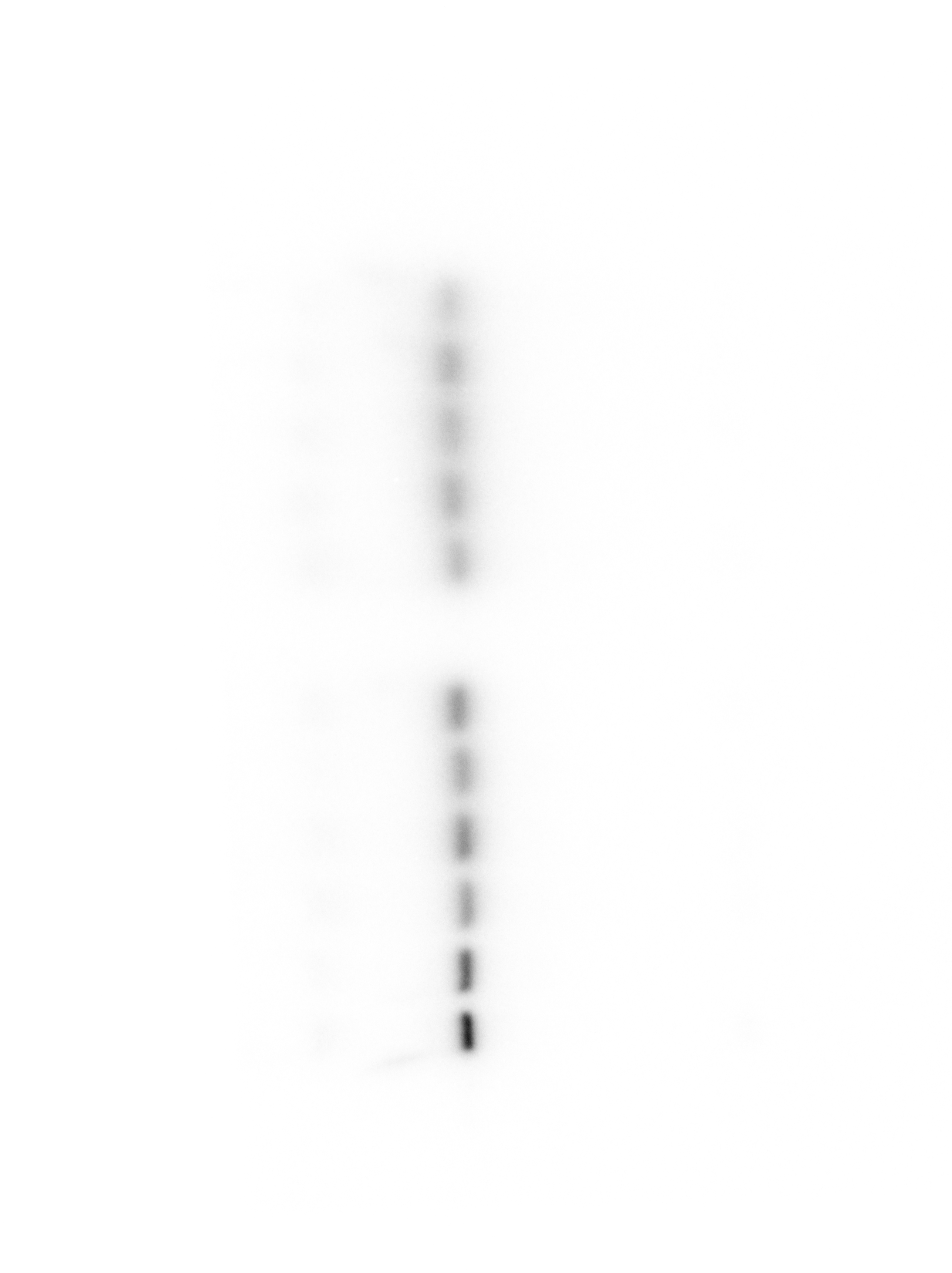

Supplement: Supplementary file 10 — Source Data of EV and Appendix figures [file 44318_2024_35_MOESM10_ESM.zip › EMBOJ-2023-115792R2_SourceData_EV+Appendix/FigEV2/FigEV2B northern blot/R1 dHEL1, 2i and 2/northern u6 dhel1 .tiff]

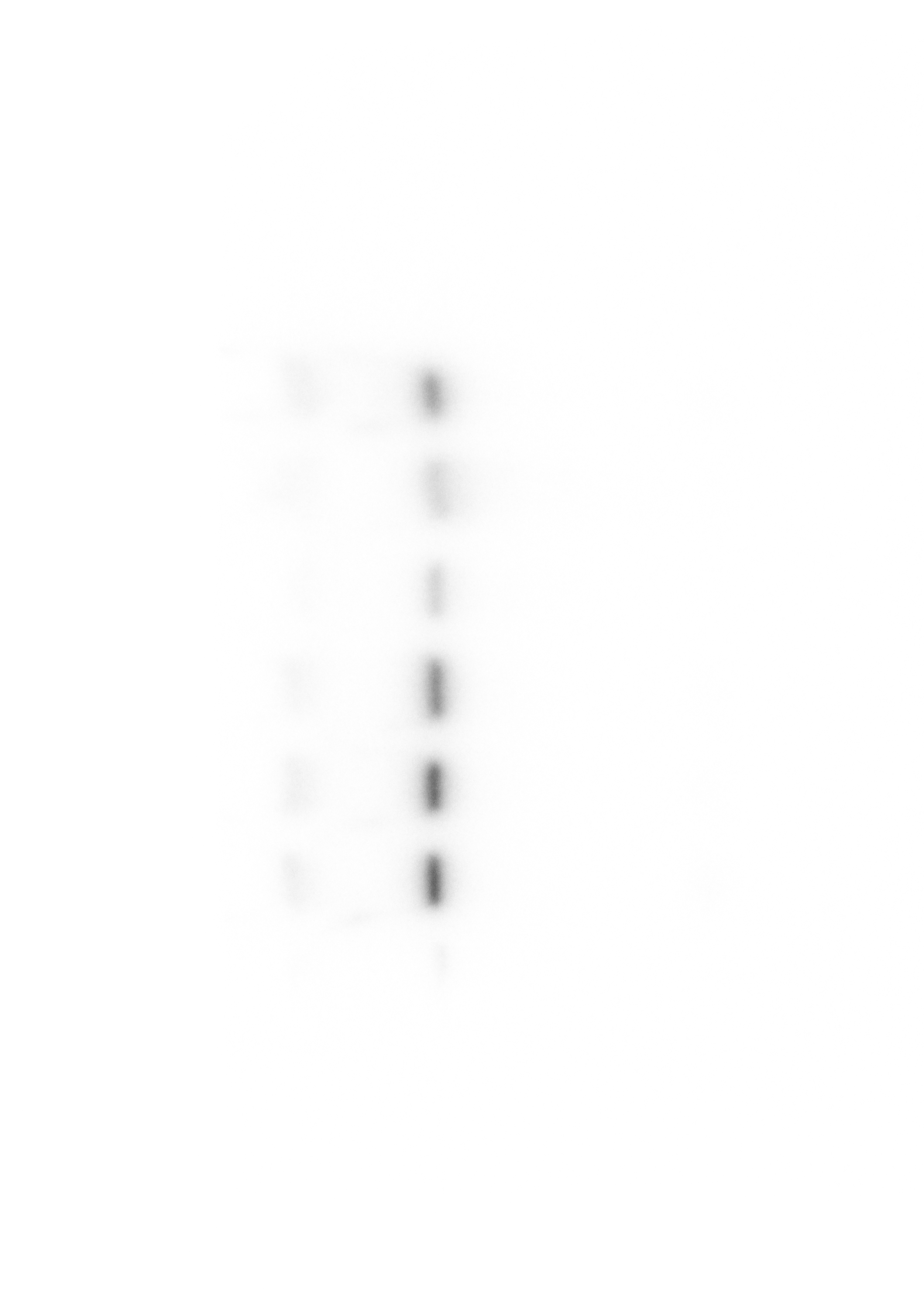

Supplement: Supplementary file 10 — Source Data of EV and Appendix figures [file 44318_2024_35_MOESM10_ESM.zip › EMBOJ-2023-115792R2_SourceData_EV+Appendix/FigEV2/FigEV2B northern blot/R1 dHEL1, 2i and 2/northern u6 dhel2i 2.tiff]

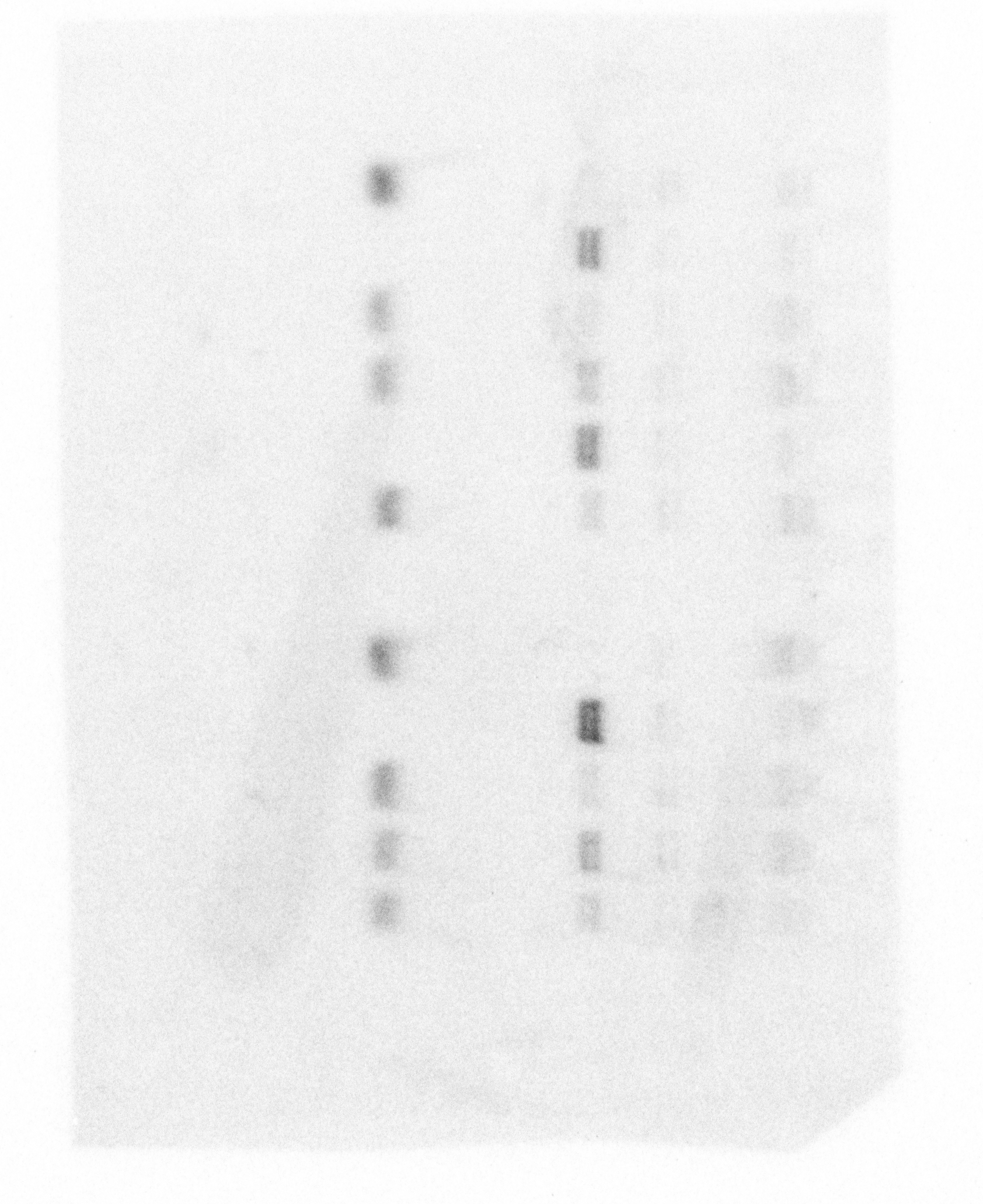

Supplement: Supplementary file 10 — Source Data of EV and Appendix figures [file 44318_2024_35_MOESM10_ESM.zip › EMBOJ-2023-115792R2_SourceData_EV+Appendix/FigEV2/FigEV2B northern blot/R1 dHEL1, 2i and 2/northern mir16 dhel1.tiff]

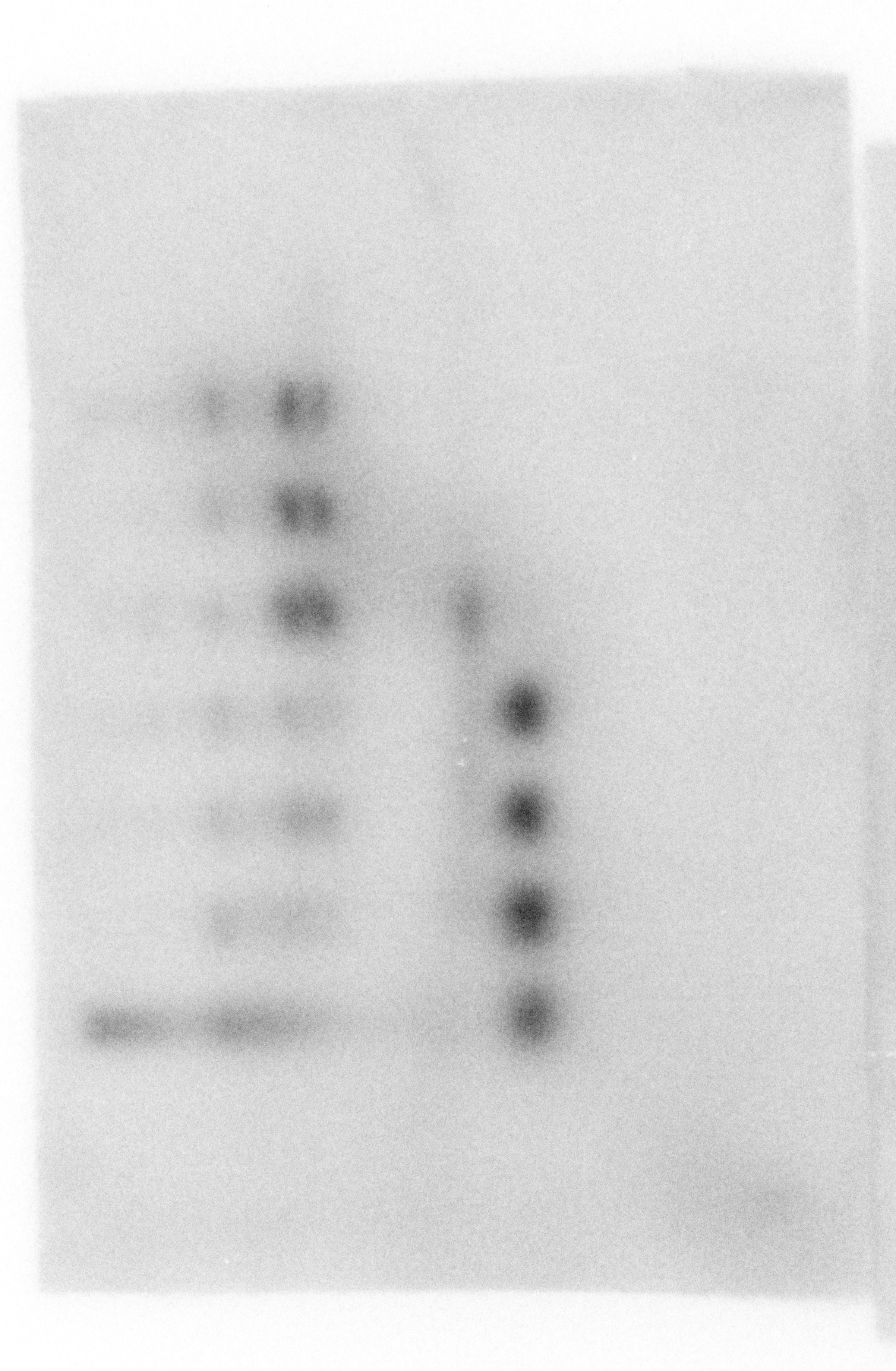

Supplement: Supplementary file 10 — Source Data of EV and Appendix figures [file 44318_2024_35_MOESM10_ESM.zip › EMBOJ-2023-115792R2_SourceData_EV+Appendix/FigEV2/FigEV2B northern blot/R1 dHEL1-CM, 2i-CM and 2-CM/northern mir16.tiff]

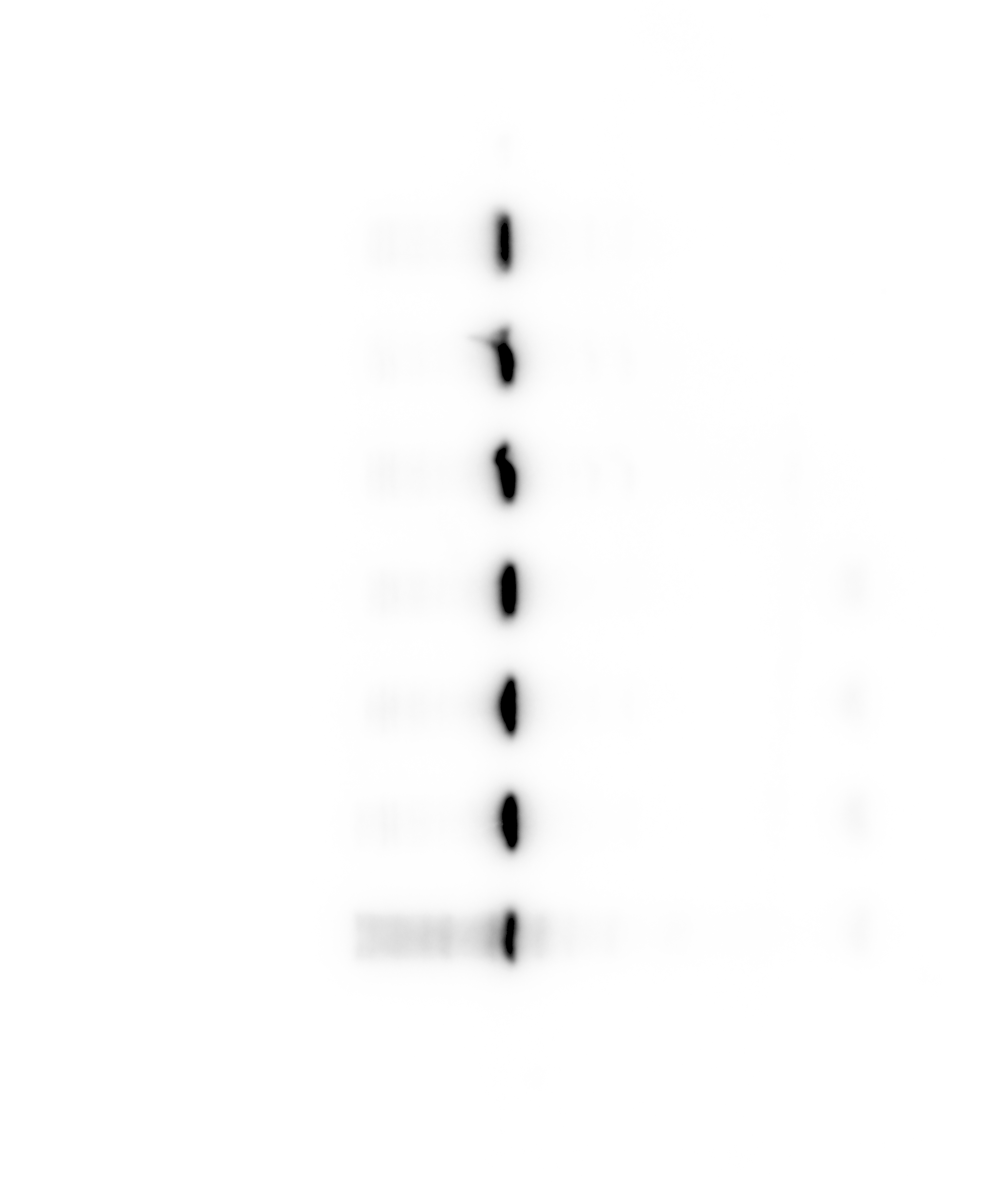

Supplement: Supplementary file 10 — Source Data of EV and Appendix figures [file 44318_2024_35_MOESM10_ESM.zip › EMBOJ-2023-115792R2_SourceData_EV+Appendix/FigEV2/FigEV2B northern blot/R1 dHEL1-CM, 2i-CM and 2-CM/northern U6.tiff]

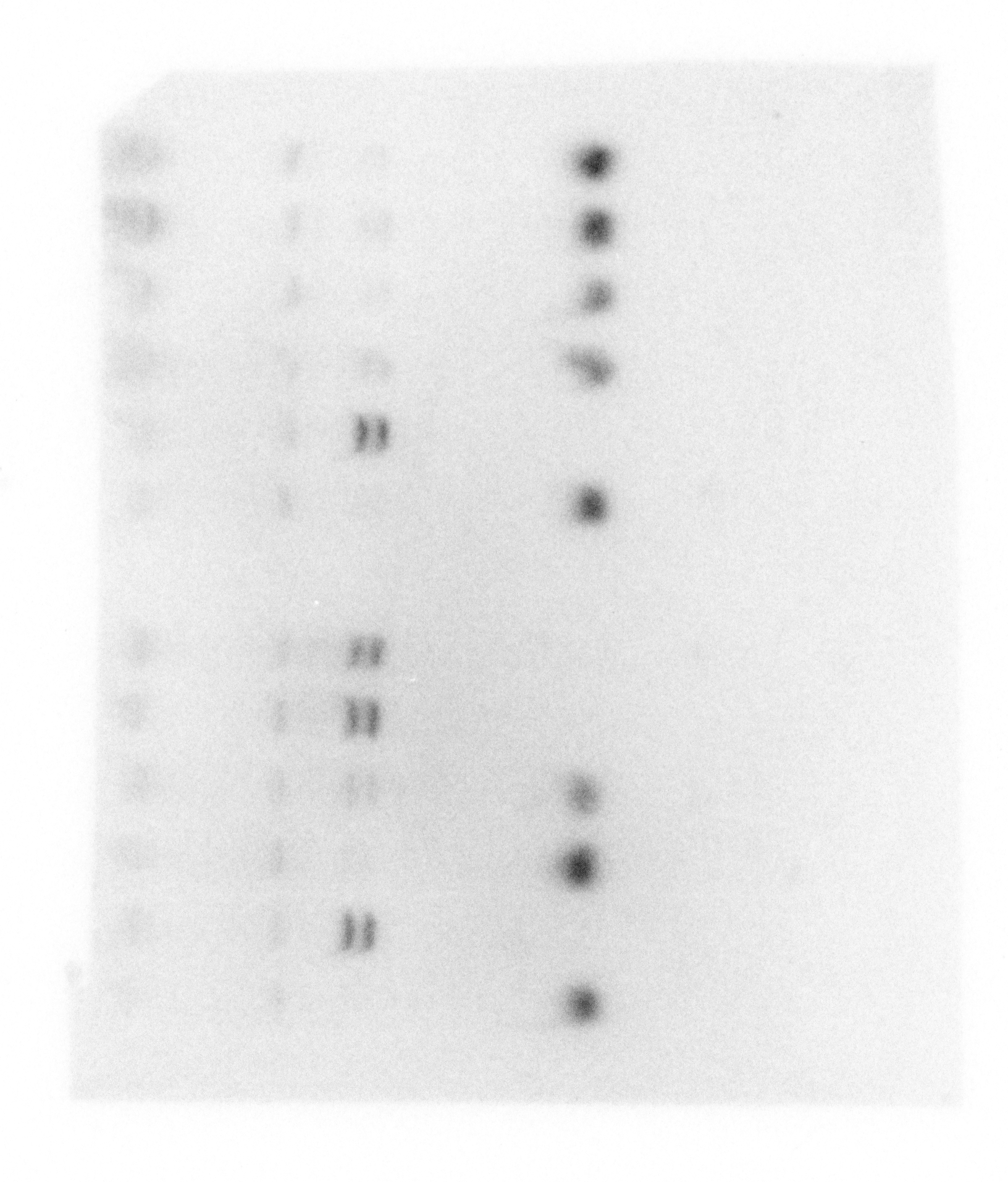

Supplement: Supplementary file 10 — Source Data of EV and Appendix figures [file 44318_2024_35_MOESM10_ESM.zip › EMBOJ-2023-115792R2_SourceData_EV+Appendix/FigEV2/FigEV2B northern blot/R2 dHEL1, 2i and 2/northern miR16.tiff]

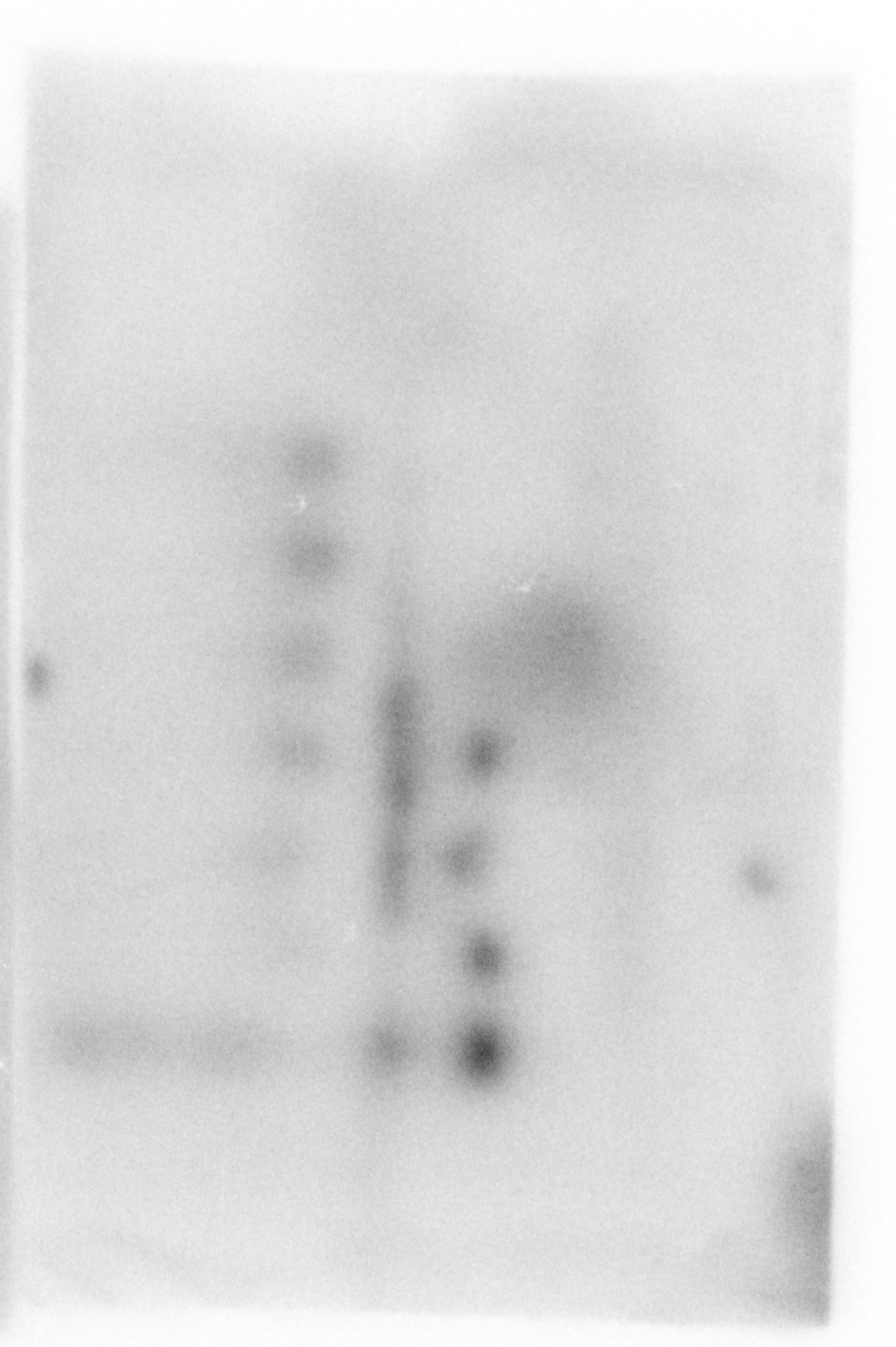

Supplement: Supplementary file 10 — Source Data of EV and Appendix figures [file 44318_2024_35_MOESM10_ESM.zip › EMBOJ-2023-115792R2_SourceData_EV+Appendix/FigEV2/FigEV2B northern blot/R2 dHEL1-CM, 2i-CM and 2-CM/northern miR16.tiff]

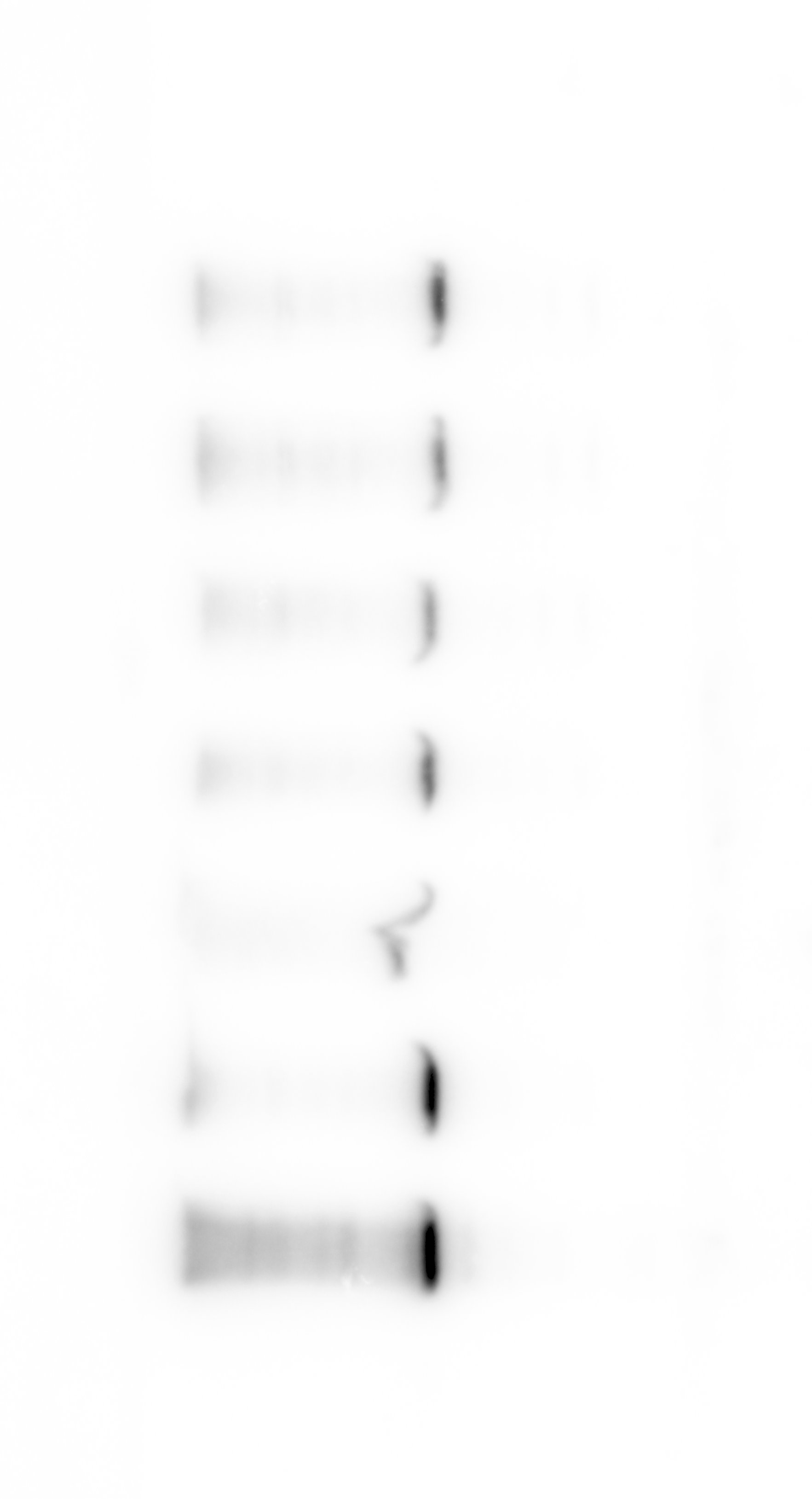

Supplement: Supplementary file 10 — Source Data of EV and Appendix figures [file 44318_2024_35_MOESM10_ESM.zip › EMBOJ-2023-115792R2_SourceData_EV+Appendix/FigEV2/FigEV2B northern blot/R2 dHEL1-CM, 2i-CM and 2-CM/northern U6.tiff]

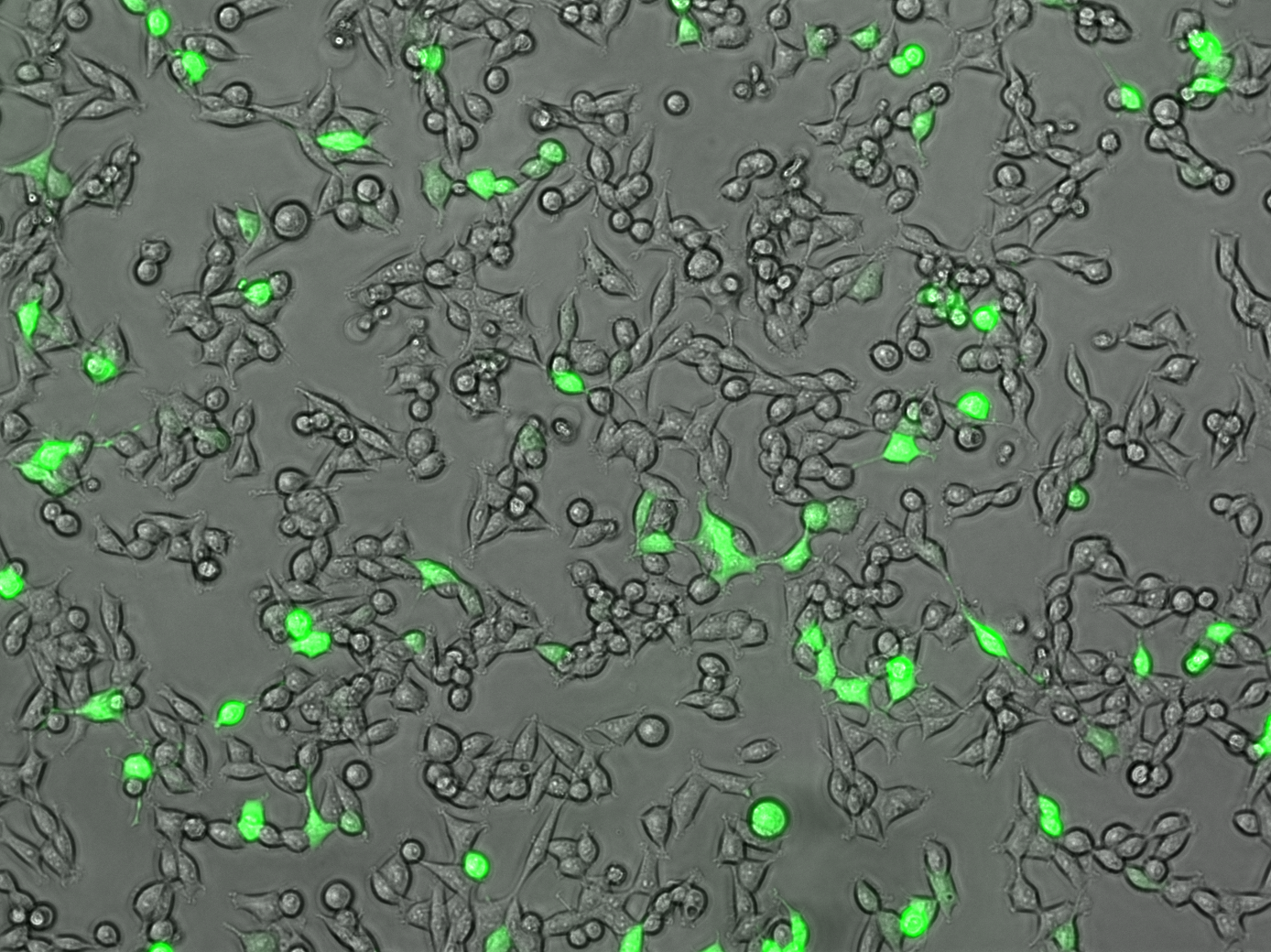

Supplement: Supplementary file 10 — Source Data of EV and Appendix figures [file 44318_2024_35_MOESM10_ESM.zip › EMBOJ-2023-115792R2_SourceData_EV+Appendix/Appendix Figure S1/Appendix_FigS1A microscopy/R1/2A-GFP R1/merge.tif]

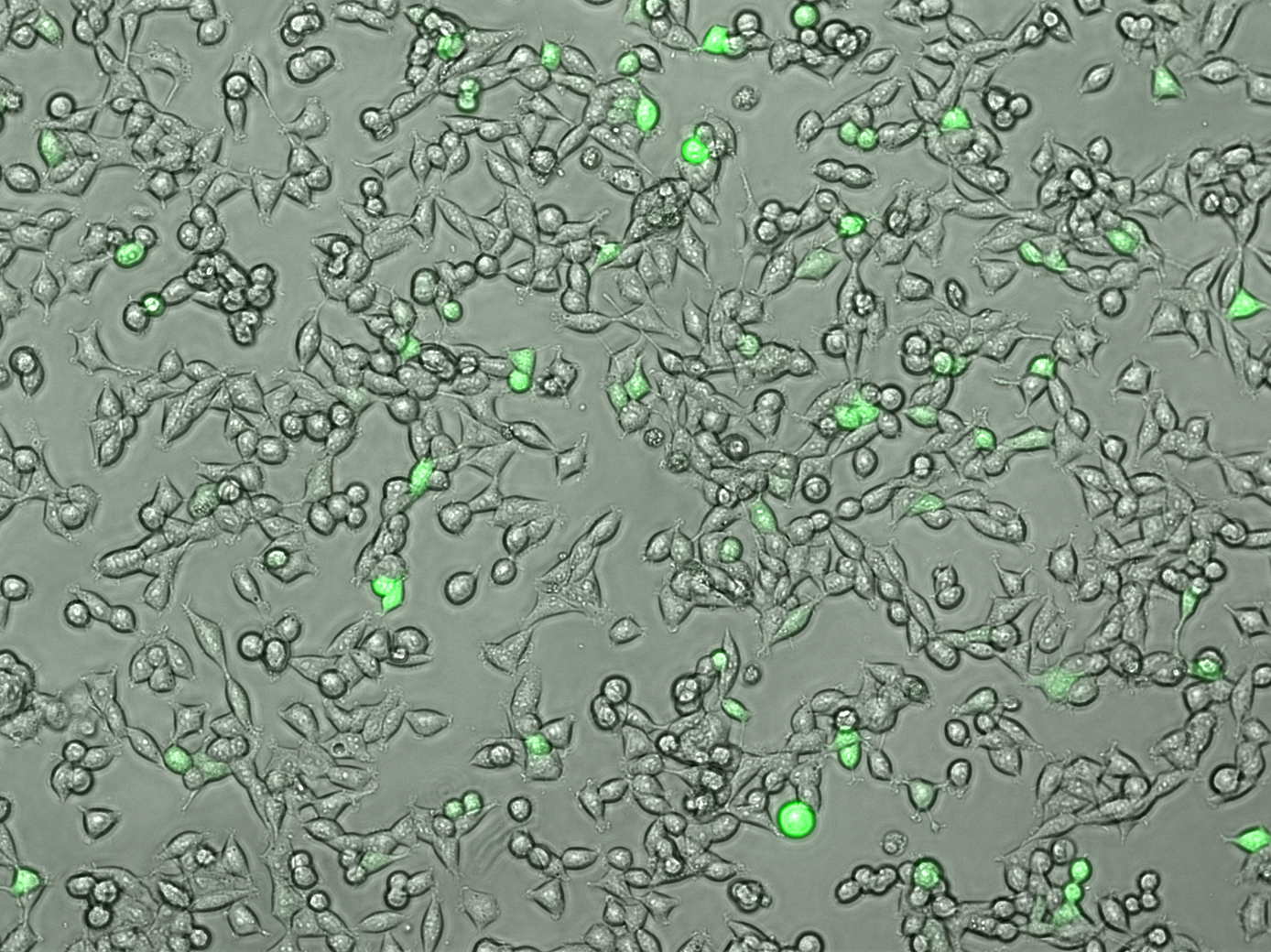

Supplement: Supplementary file 10 — Source Data of EV and Appendix figures [file 44318_2024_35_MOESM10_ESM.zip › EMBOJ-2023-115792R2_SourceData_EV+Appendix/Appendix Figure S1/Appendix_FigS1A microscopy/R1/GFP R1/merge.tif]

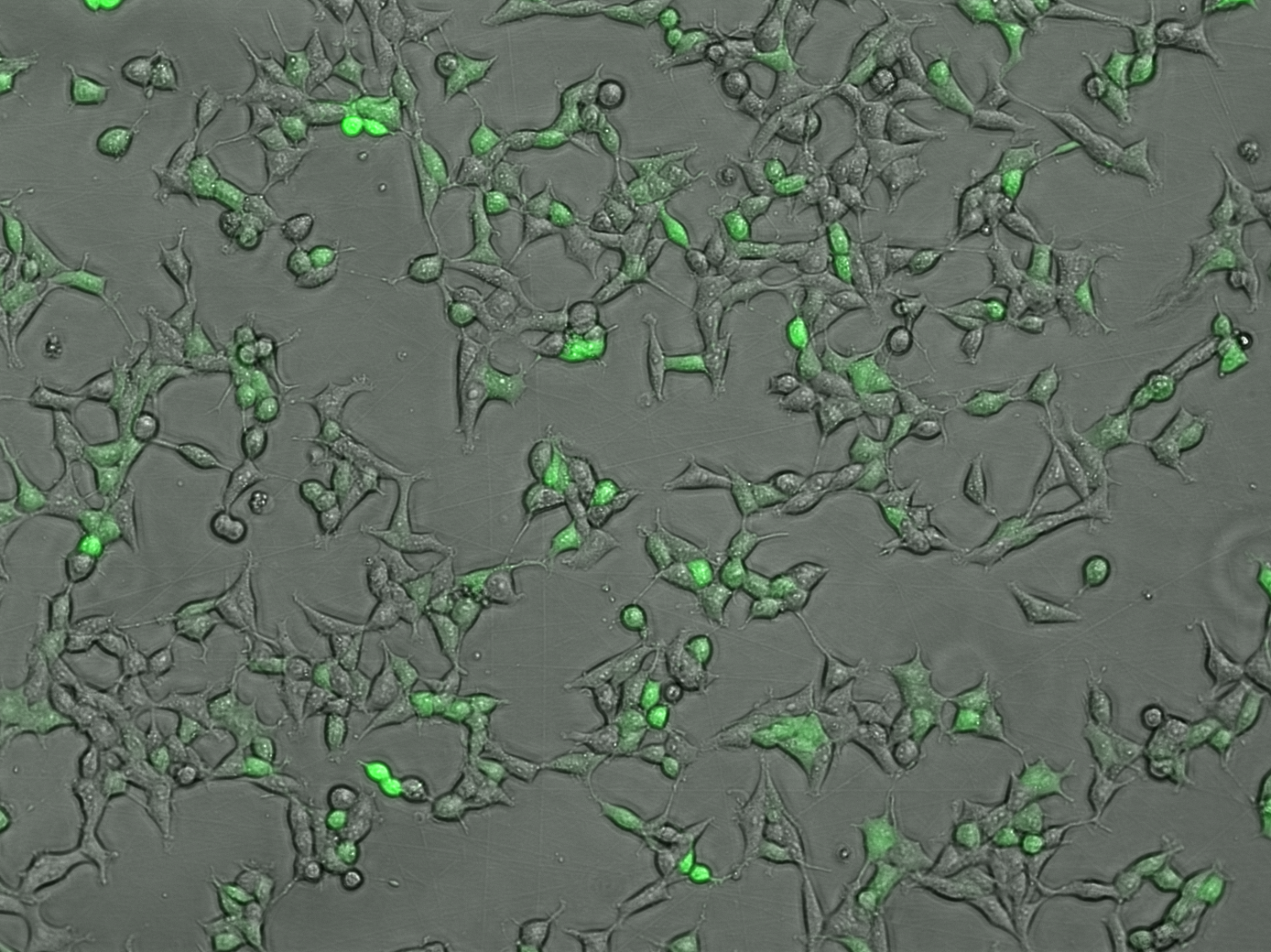

Supplement: Supplementary file 10 — Source Data of EV and Appendix figures [file 44318_2024_35_MOESM10_ESM.zip › EMBOJ-2023-115792R2_SourceData_EV+Appendix/Appendix Figure S1/Appendix_FigS1A microscopy/R3/GFP R3/merge.tif]

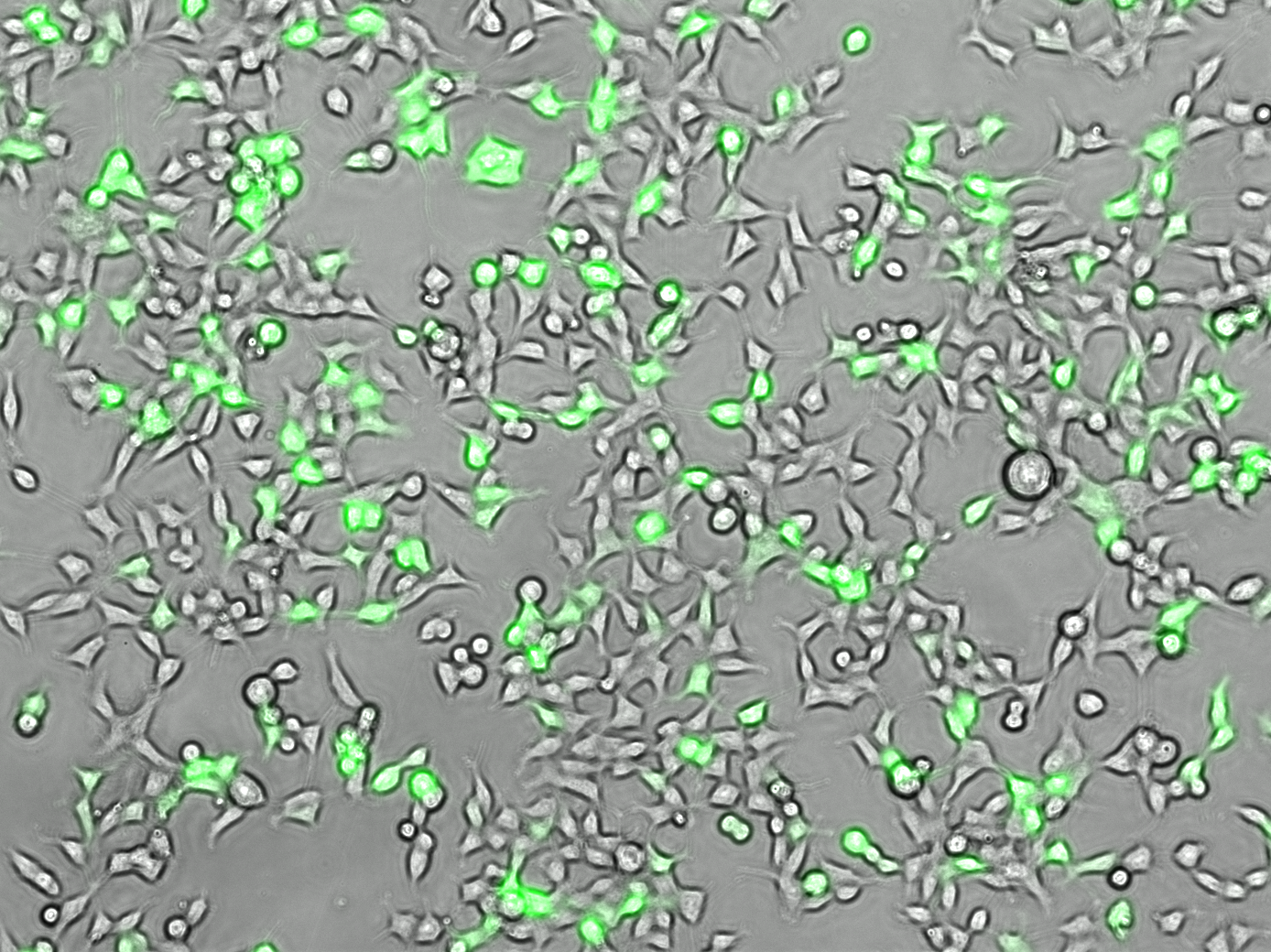

Supplement: Supplementary file 10 — Source Data of EV and Appendix figures [file 44318_2024_35_MOESM10_ESM.zip › EMBOJ-2023-115792R2_SourceData_EV+Appendix/Appendix Figure S1/Appendix_FigS1A microscopy/R3/2A R3/merge.tif]

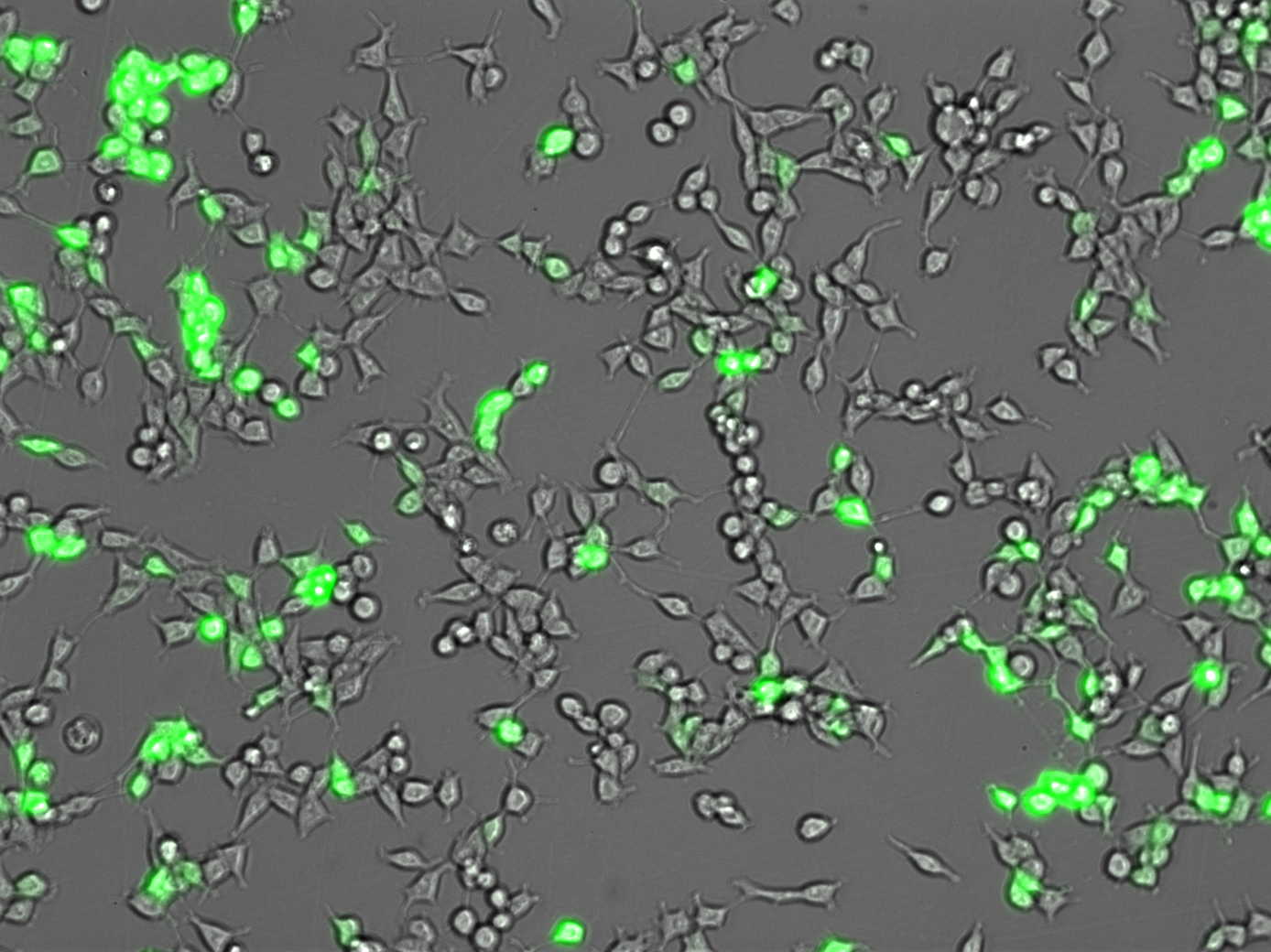

Supplement: Supplementary file 10 — Source Data of EV and Appendix figures [file 44318_2024_35_MOESM10_ESM.zip › EMBOJ-2023-115792R2_SourceData_EV+Appendix/Appendix Figure S1/Appendix_FigS1A microscopy/R2/2A R2/merge.tif]

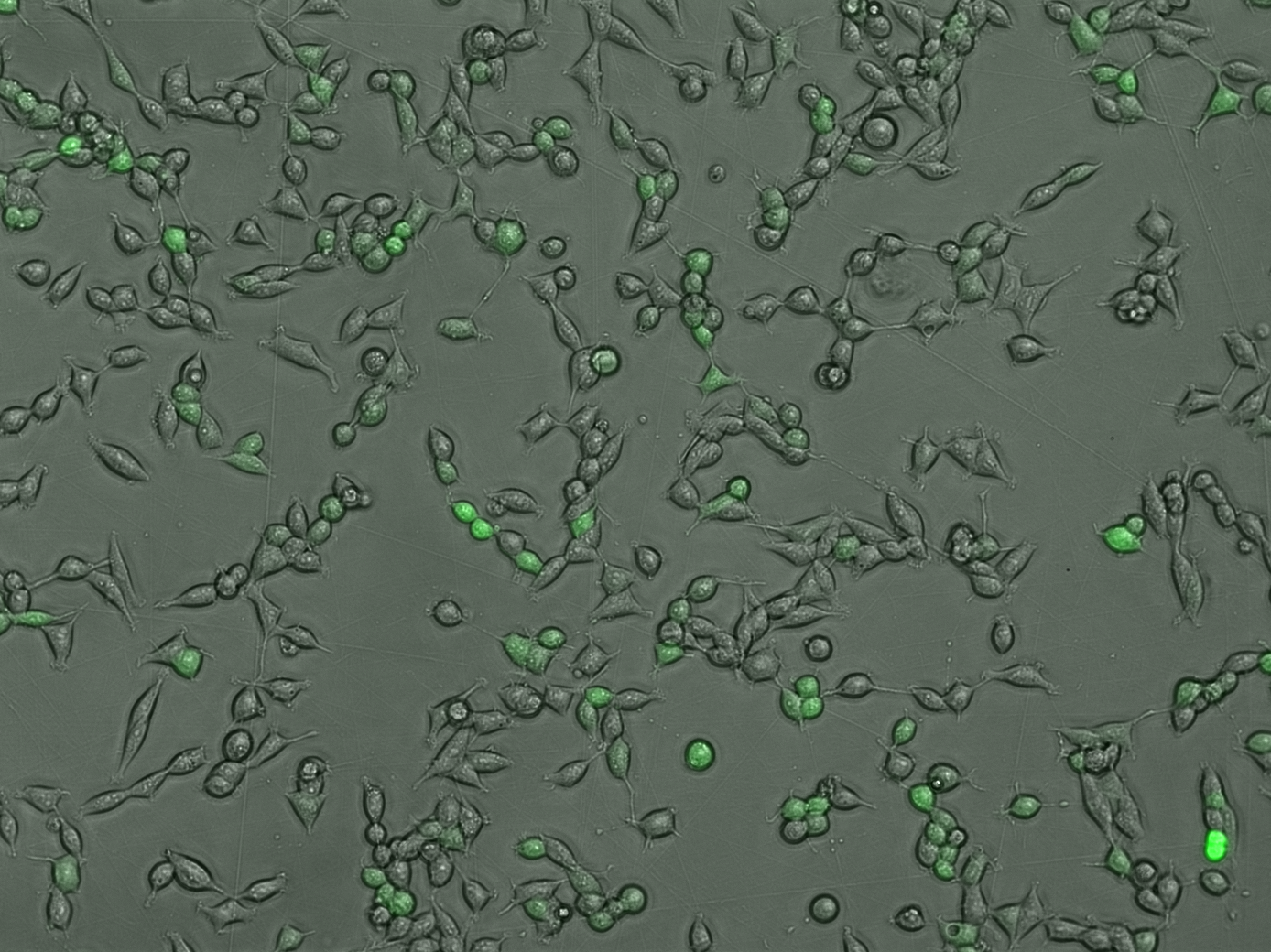

Supplement: Supplementary file 10 — Source Data of EV and Appendix figures [file 44318_2024_35_MOESM10_ESM.zip › EMBOJ-2023-115792R2_SourceData_EV+Appendix/Appendix Figure S1/Appendix_FigS1A microscopy/R2/GFP R2/merge.tif]

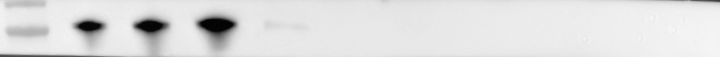

Supplement: Supplementary file 10 — Source Data of EV and Appendix figures [file 44318_2024_35_MOESM10_ESM.zip › EMBOJ-2023-115792R2_SourceData_EV+Appendix/FigEV1/FigEV1F western blot/R1/MOCK/western PKR.tiff]

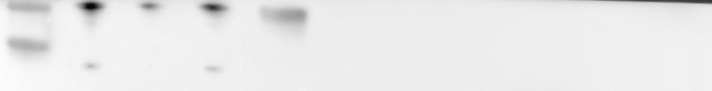

Supplement: Supplementary file 10 — Source Data of EV and Appendix figures [file 44318_2024_35_MOESM10_ESM.zip › EMBOJ-2023-115792R2_SourceData_EV+Appendix/FigEV1/FigEV1F western blot/R1/MOCK/western PACT.tiff]

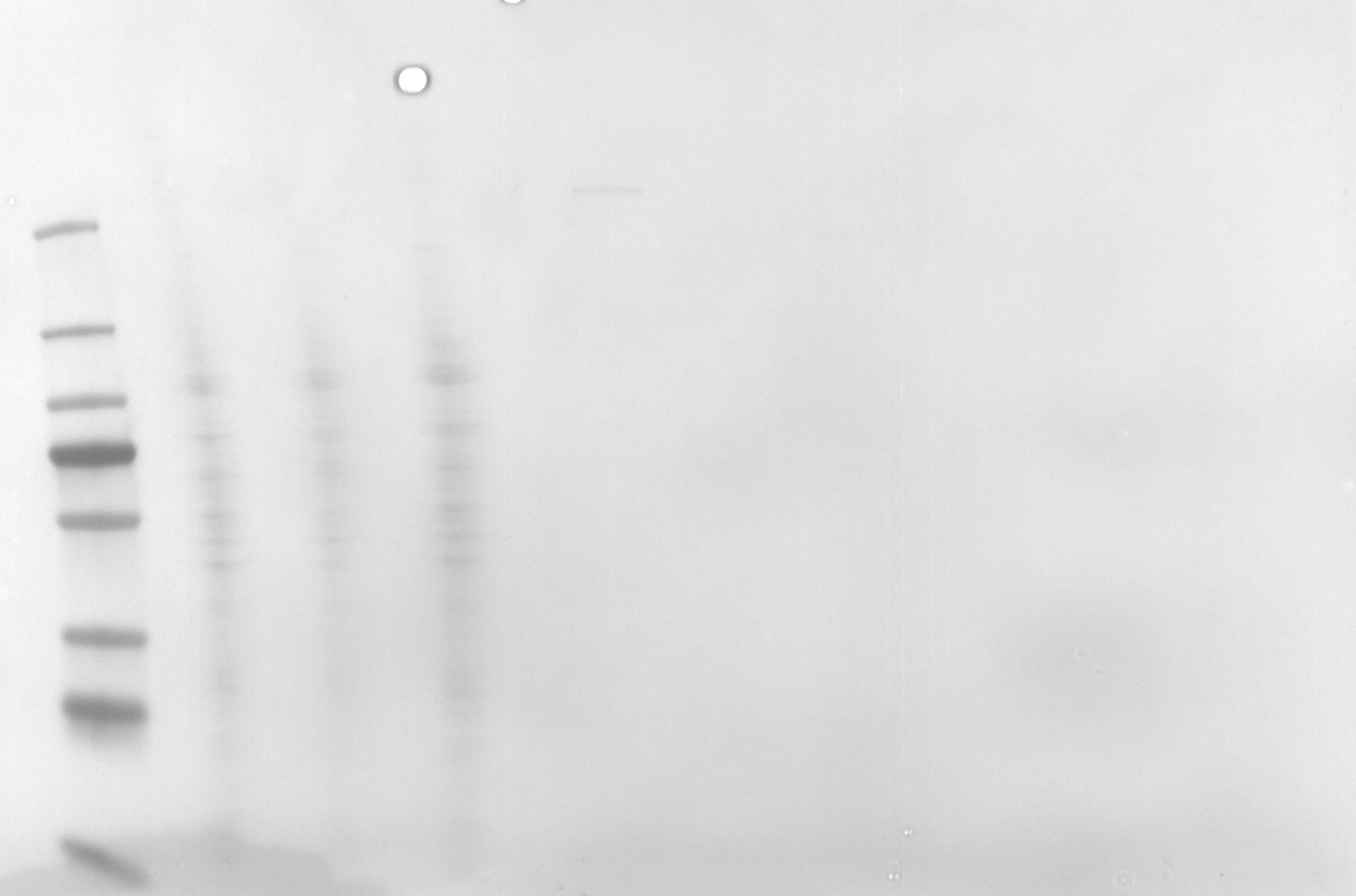

Supplement: Supplementary file 10 — Source Data of EV and Appendix figures [file 44318_2024_35_MOESM10_ESM.zip › EMBOJ-2023-115792R2_SourceData_EV+Appendix/FigEV1/FigEV1F western blot/R1/MOCK/western ponceau.tiff]

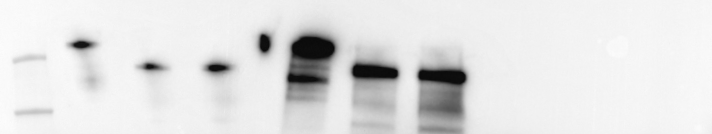

Supplement: Supplementary file 10 — Source Data of EV and Appendix figures [file 44318_2024_35_MOESM10_ESM.zip › EMBOJ-2023-115792R2_SourceData_EV+Appendix/FigEV1/FigEV1F western blot/R1/MOCK/western HA.tiff]

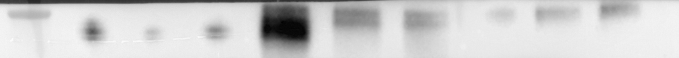

Supplement: Supplementary file 10 — Source Data of EV and Appendix figures [file 44318_2024_35_MOESM10_ESM.zip › EMBOJ-2023-115792R2_SourceData_EV+Appendix/FigEV1/FigEV1F western blot/R1/MOCK/western TRBP.tiff]

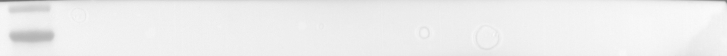

Supplement: Supplementary file 10 — Source Data of EV and Appendix figures [file 44318_2024_35_MOESM10_ESM.zip › EMBOJ-2023-115792R2_SourceData_EV+Appendix/FigEV1/FigEV1F western blot/R1/MOCK/western p-PKR.tiff]

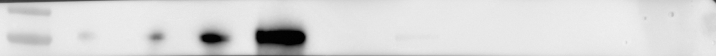

Supplement: Supplementary file 10 — Source Data of EV and Appendix figures [file 44318_2024_35_MOESM10_ESM.zip › EMBOJ-2023-115792R2_SourceData_EV+Appendix/FigEV1/FigEV1F western blot/R1/SINV/western PKR.tiff]

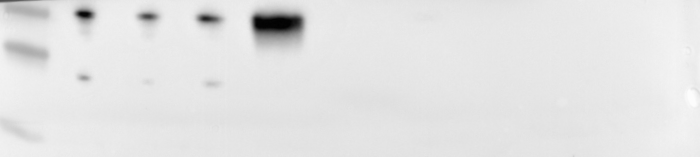

Supplement: Supplementary file 10 — Source Data of EV and Appendix figures [file 44318_2024_35_MOESM10_ESM.zip › EMBOJ-2023-115792R2_SourceData_EV+Appendix/FigEV1/FigEV1F western blot/R1/SINV/western PACT.tiff]

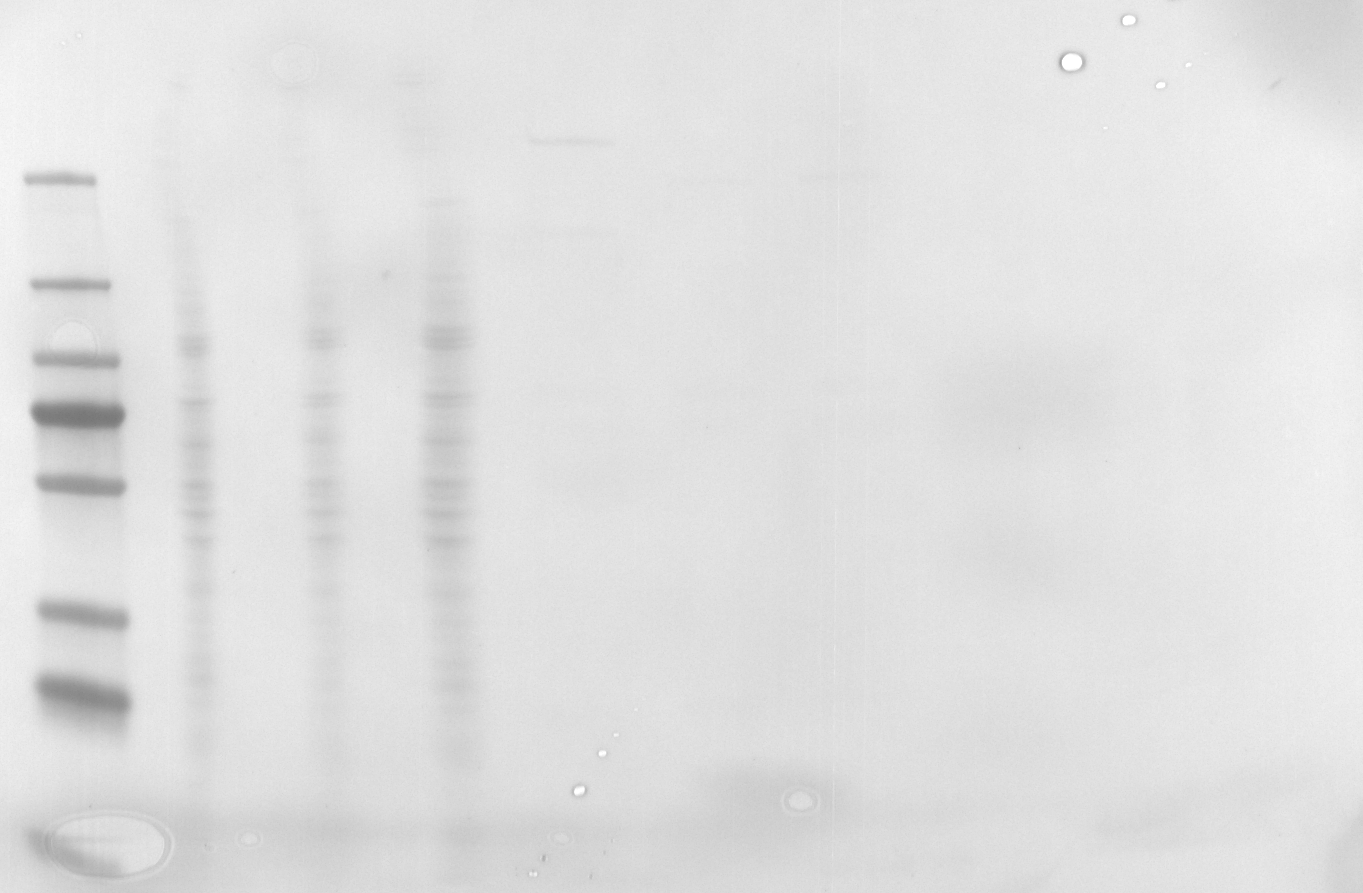

Supplement: Supplementary file 10 — Source Data of EV and Appendix figures [file 44318_2024_35_MOESM10_ESM.zip › EMBOJ-2023-115792R2_SourceData_EV+Appendix/FigEV1/FigEV1F western blot/R1/SINV/western ponceau.tiff]

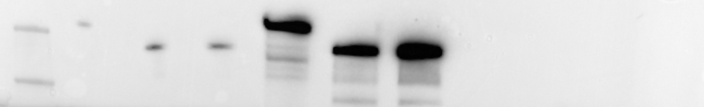

Supplement: Supplementary file 10 — Source Data of EV and Appendix figures [file 44318_2024_35_MOESM10_ESM.zip › EMBOJ-2023-115792R2_SourceData_EV+Appendix/FigEV1/FigEV1F western blot/R1/SINV/western HA.tiff]

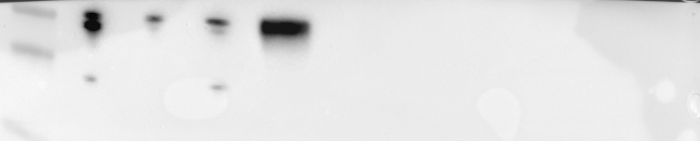

Supplement: Supplementary file 10 — Source Data of EV and Appendix figures [file 44318_2024_35_MOESM10_ESM.zip › EMBOJ-2023-115792R2_SourceData_EV+Appendix/FigEV1/FigEV1F western blot/R1/SINV/western capsid.tiff]

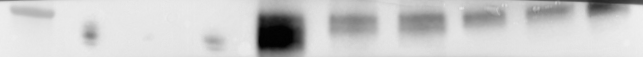

Supplement: Supplementary file 10 — Source Data of EV and Appendix figures [file 44318_2024_35_MOESM10_ESM.zip › EMBOJ-2023-115792R2_SourceData_EV+Appendix/FigEV1/FigEV1F western blot/R1/SINV/western TRBP.tiff]

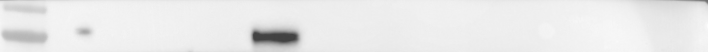

Supplement: Supplementary file 10 — Source Data of EV and Appendix figures [file 44318_2024_35_MOESM10_ESM.zip › EMBOJ-2023-115792R2_SourceData_EV+Appendix/FigEV1/FigEV1F western blot/R1/SINV/western p-PKR.tiff]

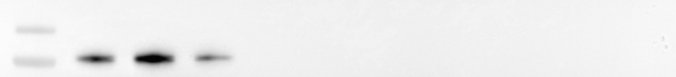

Supplement: Supplementary file 10 — Source Data of EV and Appendix figures [file 44318_2024_35_MOESM10_ESM.zip › EMBOJ-2023-115792R2_SourceData_EV+Appendix/FigEV1/FigEV1F western blot/R3/mock/western PKR.tiff]

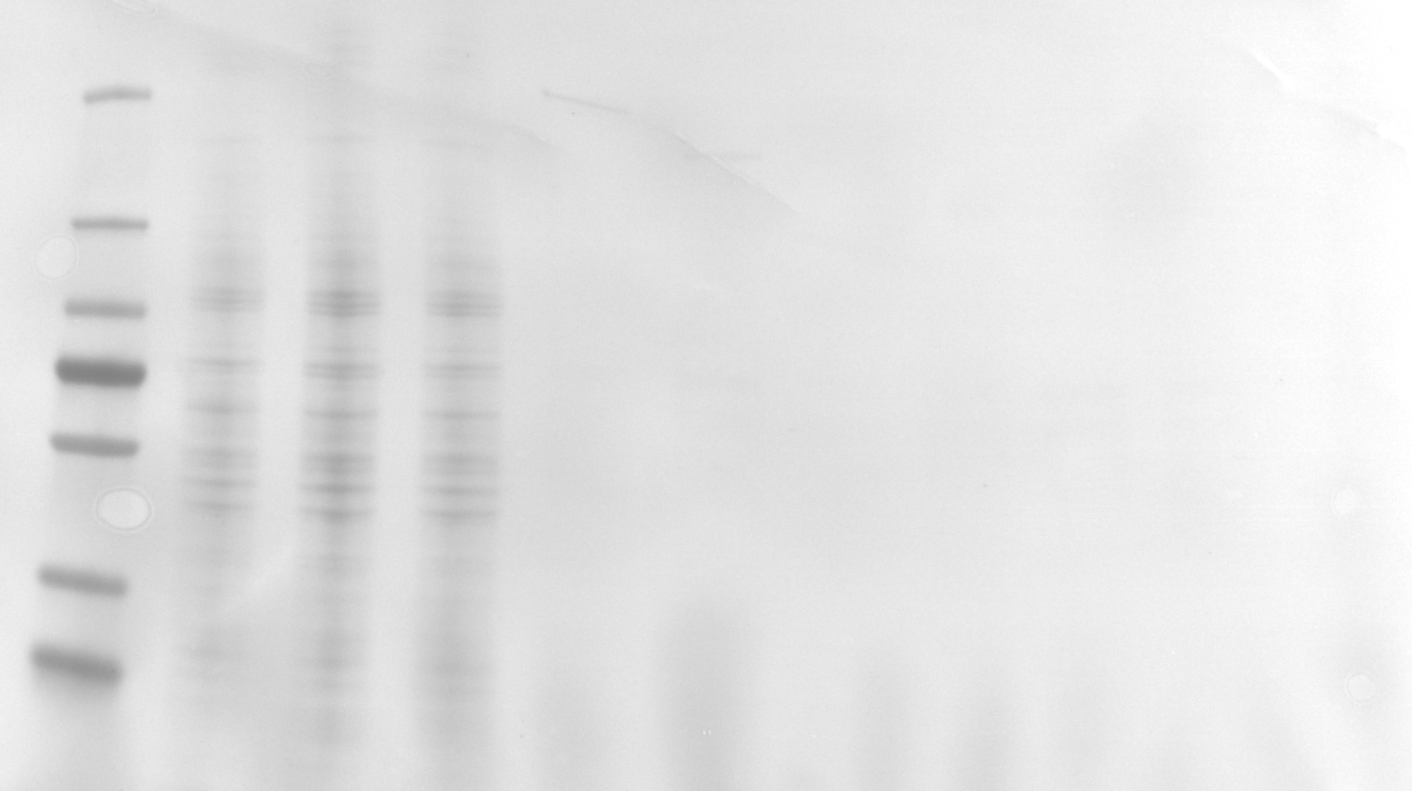

Supplement: Supplementary file 10 — Source Data of EV and Appendix figures [file 44318_2024_35_MOESM10_ESM.zip › EMBOJ-2023-115792R2_SourceData_EV+Appendix/FigEV1/FigEV1F western blot/R3/mock/western ponceau.tiff]

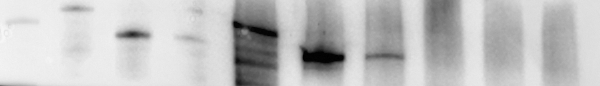

Supplement: Supplementary file 10 — Source Data of EV and Appendix figures [file 44318_2024_35_MOESM10_ESM.zip › EMBOJ-2023-115792R2_SourceData_EV+Appendix/FigEV1/FigEV1F western blot/R3/mock/western HA.tiff]

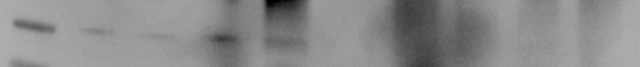

Supplement: Supplementary file 10 — Source Data of EV and Appendix figures [file 44318_2024_35_MOESM10_ESM.zip › EMBOJ-2023-115792R2_SourceData_EV+Appendix/FigEV1/FigEV1F western blot/R3/mock/western PACCT.tiff]

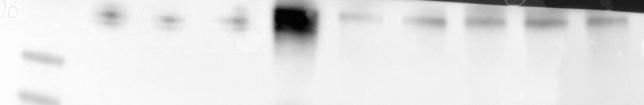

Supplement: Supplementary file 10 — Source Data of EV and Appendix figures [file 44318_2024_35_MOESM10_ESM.zip › EMBOJ-2023-115792R2_SourceData_EV+Appendix/FigEV1/FigEV1F western blot/R3/mock/western TRBP.tiff]

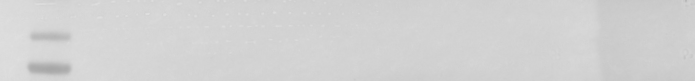

Supplement: Supplementary file 10 — Source Data of EV and Appendix figures [file 44318_2024_35_MOESM10_ESM.zip › EMBOJ-2023-115792R2_SourceData_EV+Appendix/FigEV1/FigEV1F western blot/R3/mock/western p-PKR.tiff]

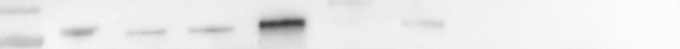

Supplement: Supplementary file 10 — Source Data of EV and Appendix figures [file 44318_2024_35_MOESM10_ESM.zip › EMBOJ-2023-115792R2_SourceData_EV+Appendix/FigEV1/FigEV1F western blot/R3/SINV/western PKR.tiff]

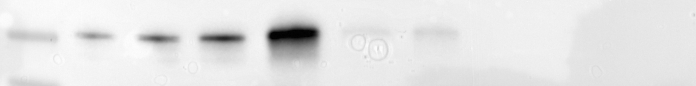

Supplement: Supplementary file 10 — Source Data of EV and Appendix figures [file 44318_2024_35_MOESM10_ESM.zip › EMBOJ-2023-115792R2_SourceData_EV+Appendix/FigEV1/FigEV1F western blot/R3/SINV/western PACT.tiff]

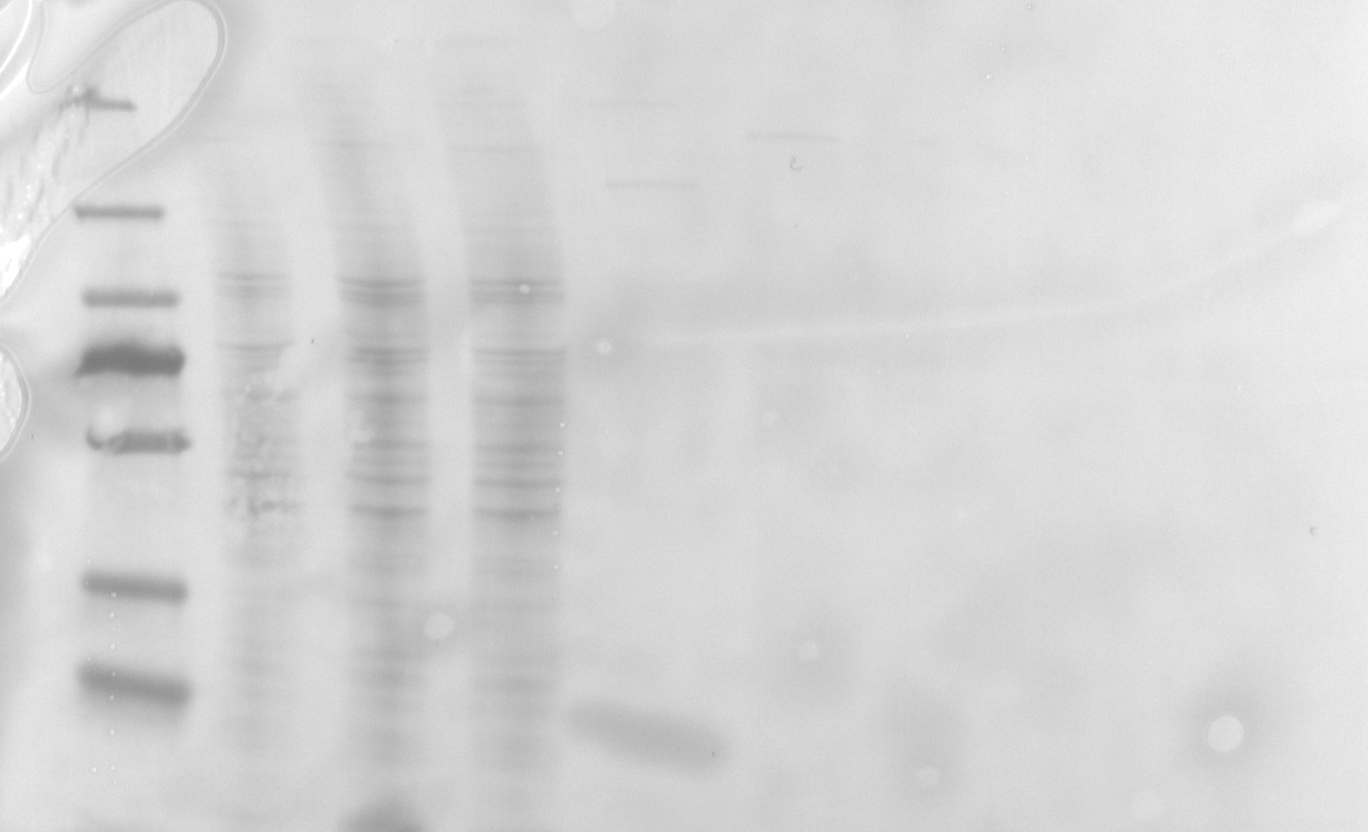

Supplement: Supplementary file 10 — Source Data of EV and Appendix figures [file 44318_2024_35_MOESM10_ESM.zip › EMBOJ-2023-115792R2_SourceData_EV+Appendix/FigEV1/FigEV1F western blot/R3/SINV/western ponceau.tiff]

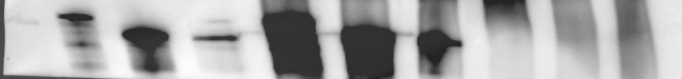

Supplement: Supplementary file 10 — Source Data of EV and Appendix figures [file 44318_2024_35_MOESM10_ESM.zip › EMBOJ-2023-115792R2_SourceData_EV+Appendix/FigEV1/FigEV1F western blot/R3/SINV/western HA.tiff]

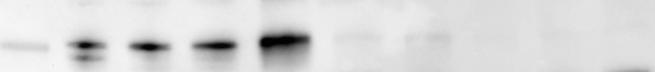

Supplement: Supplementary file 10 — Source Data of EV and Appendix figures [file 44318_2024_35_MOESM10_ESM.zip › EMBOJ-2023-115792R2_SourceData_EV+Appendix/FigEV1/FigEV1F western blot/R3/SINV/western capsid.tiff]

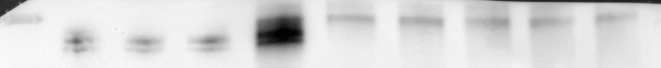

Supplement: Supplementary file 10 — Source Data of EV and Appendix figures [file 44318_2024_35_MOESM10_ESM.zip › EMBOJ-2023-115792R2_SourceData_EV+Appendix/FigEV1/FigEV1F western blot/R3/SINV/western TRBP.tiff]

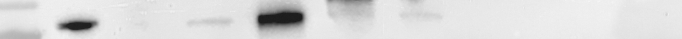

Supplement: Supplementary file 10 — Source Data of EV and Appendix figures [file 44318_2024_35_MOESM10_ESM.zip › EMBOJ-2023-115792R2_SourceData_EV+Appendix/FigEV1/FigEV1F western blot/R3/SINV/western p-PKR.tiff]

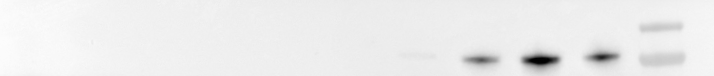

Supplement: Supplementary file 10 — Source Data of EV and Appendix figures [file 44318_2024_35_MOESM10_ESM.zip › EMBOJ-2023-115792R2_SourceData_EV+Appendix/FigEV1/FigEV1F western blot/R2/MOCK/western PKR.tiff]

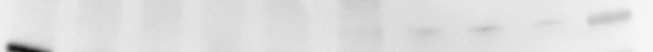

Supplement: Supplementary file 10 — Source Data of EV and Appendix figures [file 44318_2024_35_MOESM10_ESM.zip › EMBOJ-2023-115792R2_SourceData_EV+Appendix/FigEV1/FigEV1F western blot/R2/MOCK/western PACT.tiff]

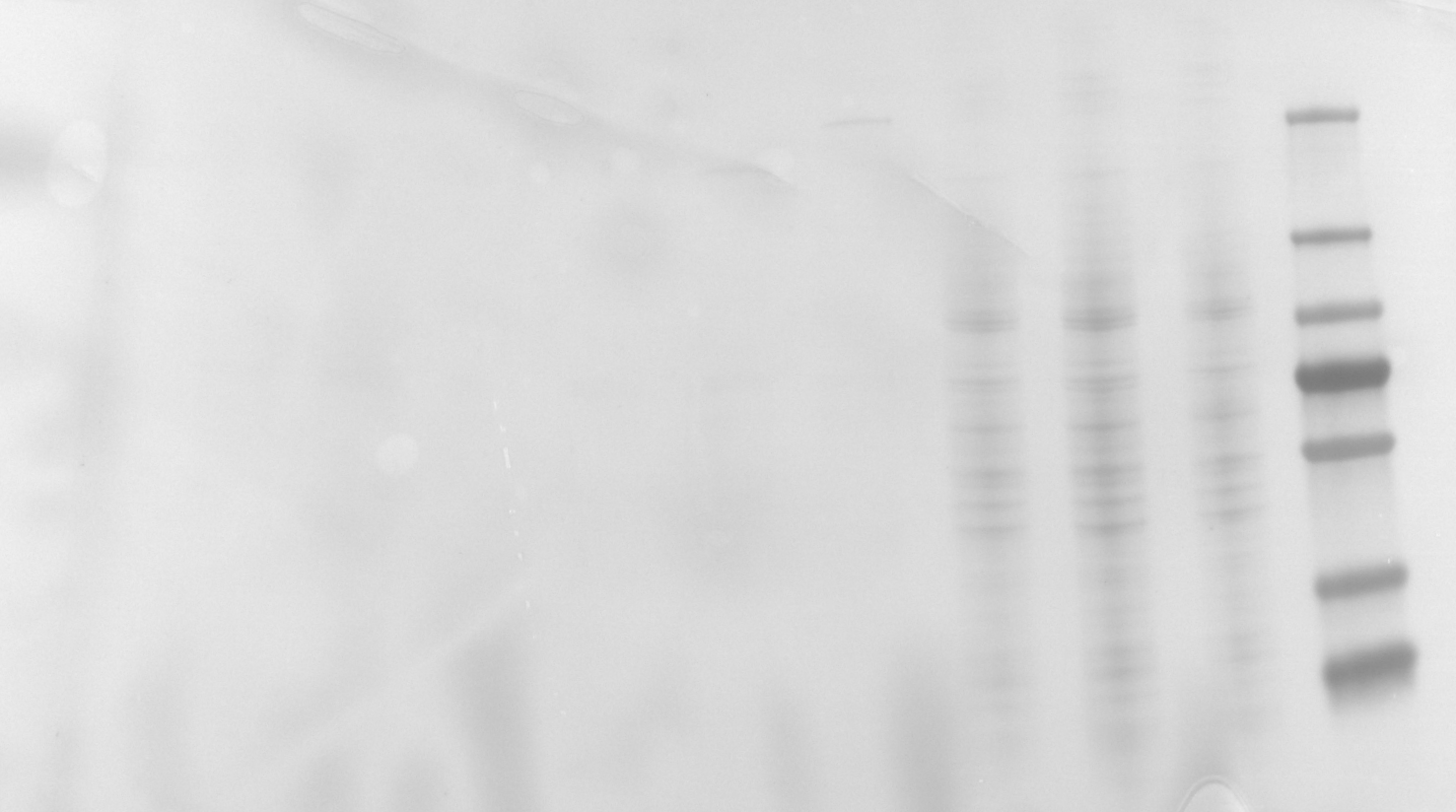

Supplement: Supplementary file 10 — Source Data of EV and Appendix figures [file 44318_2024_35_MOESM10_ESM.zip › EMBOJ-2023-115792R2_SourceData_EV+Appendix/FigEV1/FigEV1F western blot/R2/MOCK/western ponceau.tiff]

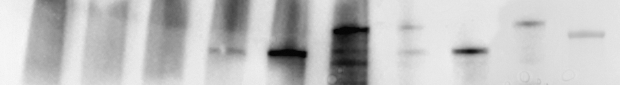

Supplement: Supplementary file 10 — Source Data of EV and Appendix figures [file 44318_2024_35_MOESM10_ESM.zip › EMBOJ-2023-115792R2_SourceData_EV+Appendix/FigEV1/FigEV1F western blot/R2/MOCK/western HA.tiff]

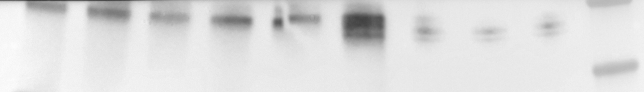

Supplement: Supplementary file 10 — Source Data of EV and Appendix figures [file 44318_2024_35_MOESM10_ESM.zip › EMBOJ-2023-115792R2_SourceData_EV+Appendix/FigEV1/FigEV1F western blot/R2/MOCK/western TRBP.tiff]

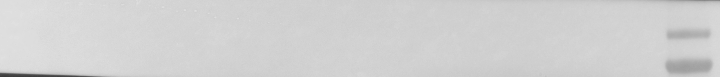

Supplement: Supplementary file 10 — Source Data of EV and Appendix figures [file 44318_2024_35_MOESM10_ESM.zip › EMBOJ-2023-115792R2_SourceData_EV+Appendix/FigEV1/FigEV1F western blot/R2/MOCK/western p-PKR.tiff]

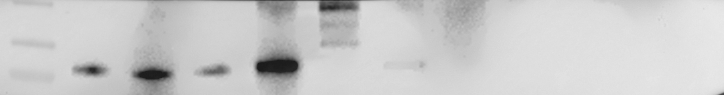

Supplement: Supplementary file 10 — Source Data of EV and Appendix figures [file 44318_2024_35_MOESM10_ESM.zip › EMBOJ-2023-115792R2_SourceData_EV+Appendix/FigEV1/FigEV1F western blot/R2/SINV/western PKR.tiff]

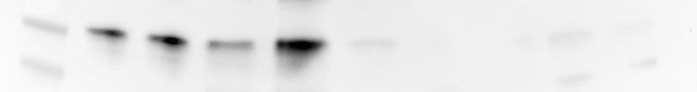

Supplement: Supplementary file 10 — Source Data of EV and Appendix figures [file 44318_2024_35_MOESM10_ESM.zip › EMBOJ-2023-115792R2_SourceData_EV+Appendix/FigEV1/FigEV1F western blot/R2/SINV/western PACT.tiff]

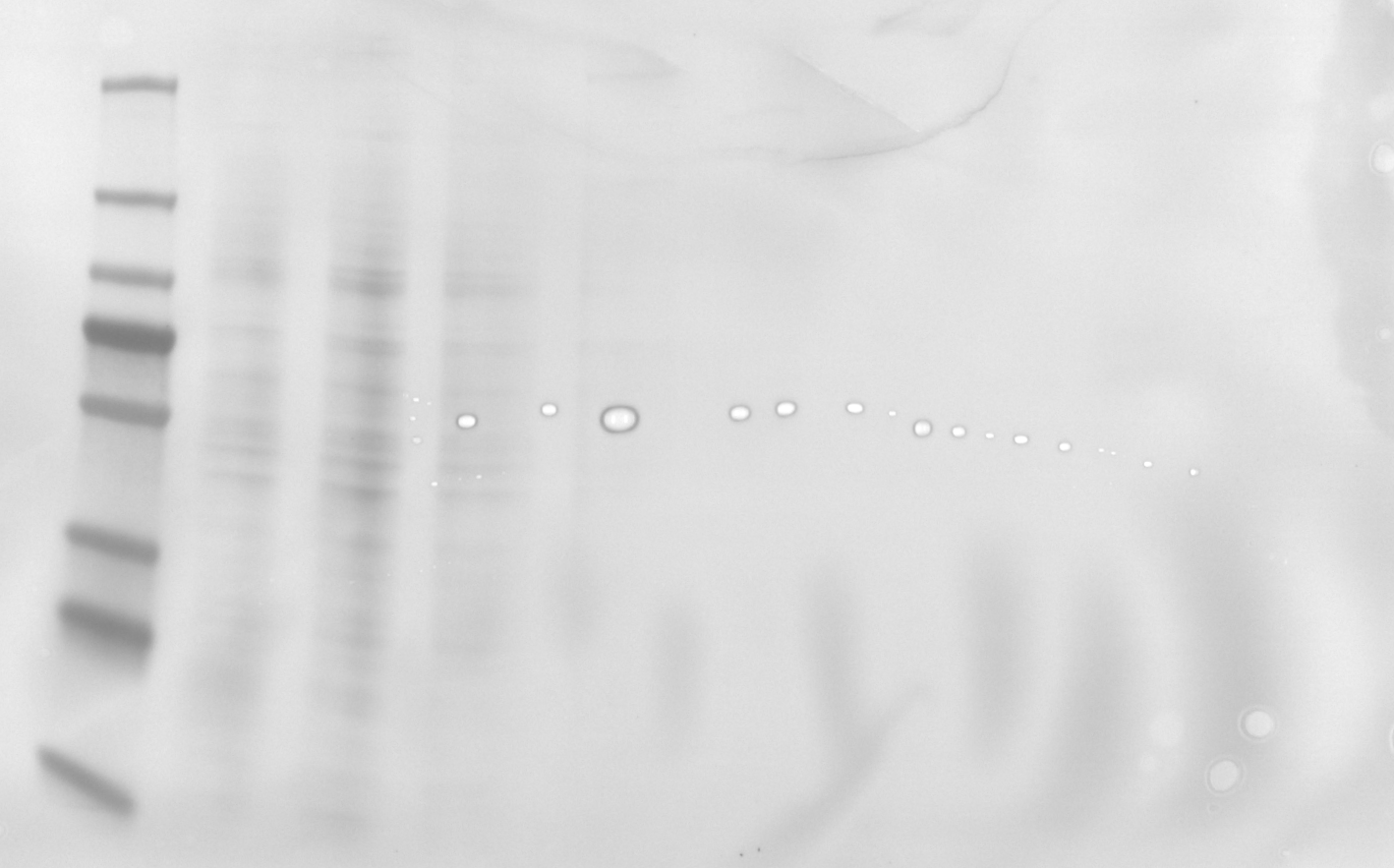

Supplement: Supplementary file 10 — Source Data of EV and Appendix figures [file 44318_2024_35_MOESM10_ESM.zip › EMBOJ-2023-115792R2_SourceData_EV+Appendix/FigEV1/FigEV1F western blot/R2/SINV/western ponceau.tiff]

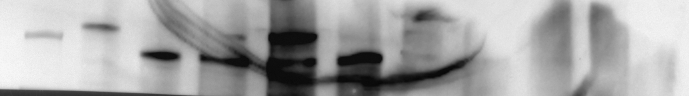

Supplement: Supplementary file 10 — Source Data of EV and Appendix figures [file 44318_2024_35_MOESM10_ESM.zip › EMBOJ-2023-115792R2_SourceData_EV+Appendix/FigEV1/FigEV1F western blot/R2/SINV/western HA.tiff]

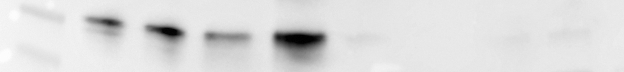

Supplement: Supplementary file 10 — Source Data of EV and Appendix figures [file 44318_2024_35_MOESM10_ESM.zip › EMBOJ-2023-115792R2_SourceData_EV+Appendix/FigEV1/FigEV1F western blot/R2/SINV/western capsid.tiff]

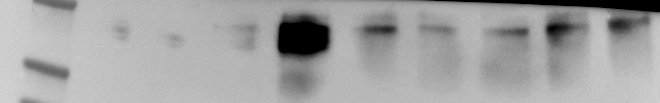

Supplement: Supplementary file 10 — Source Data of EV and Appendix figures [file 44318_2024_35_MOESM10_ESM.zip › EMBOJ-2023-115792R2_SourceData_EV+Appendix/FigEV1/FigEV1F western blot/R2/SINV/western TRBP.tiff]

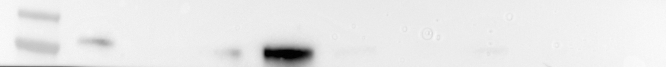

Supplement: Supplementary file 10 — Source Data of EV and Appendix figures [file 44318_2024_35_MOESM10_ESM.zip › EMBOJ-2023-115792R2_SourceData_EV+Appendix/FigEV1/FigEV1F western blot/R2/SINV/western p-PKR.tiff]
