# Supplementary material for: The helicase domain of human Dicer prevents RNAi-independent activation of antiviral and inflammatory pathways
Source: EMBO J. 2024 Jan 29;43(5):7. doi: 10.1038/s44318-024-00035-2 (PMC10907635; doi:10.1038/s44318-024-00035-2)
Supplement: Supplementary file 12 — Expanded View Figures [file 44318_2024_35_MOESM12_ESM.pdf]

Expanded View Figures

Figure EV1. Dicer N1 retains the same miRNA profile and interacting partners upon infection.

(**A**) Representation of small RNA reads distribution (in percent) upon AGO-IP in the three replicates for NoDice FHA:DICER WT and N1. Green: reads mapping to human RNAs only; Red: reads mapping to viral RNAs only; gray: reads mapping to both human and viral RNAs. (**B**) Heatmap representing the relative expression levels of the 50 most abundant human miRNAs in all three replicates upon AGO-IP followed by small RNA sequencing in SINV-GFP infected NoDice FHA:DICER WT and N1 cells at an MOI of 0.02 for 24 h. (**C**) MA plot for miRNA enrichment upon AGO-IP followed by small RNA sequencing in SINV-GFP infected NoDice FHA:DICER N1 vs WT cells at an MOI of 0.02 for 24 h. Each dot represents a miRNA either up- (red), down- (blue) or un-regulated (gray). $n = 3$ biological replicates (Wald test, DESeq2 package). (**D**) Western blot analysis of DICER and CAPSID expression in SINV-GFP infected polyclonal NoDice FHA:DICER WT, N1 and N1-CM cells at an MOI of 0.02 for 24 h. Alpha-Tubulin was used as loading control. Band intensity was quantified and normalized to Tubulin for three independent biological replicates, then represented as mean (+/− SD) under the corresponding lane. (**E**) Mean (+/− SEM) of SINV-GFP viral titers in the same samples as in (**D**), infected at an MOI of 0.02 for 24 h ($n = 3$ biological replicates) from plaque assay quantification. Ordinary one-way ANOVA test with Dunnett's correction. ****$p < 0.0001$. (**F**) Western blot analysis of Dicer interacting partners upon HA-IP in NoDice FHA:DICER WT, N1 and N1-CM cells, in mock (left) or SINV-GFP infected (right) conditions at an MOI of 2 for 6 h ($n = 3$ biological replicates). Anti-HA antibodies were used to validate the immunoprecipitation and Ponceau was used as a loading control. Source data are available online for this figure.

© The Author(s)

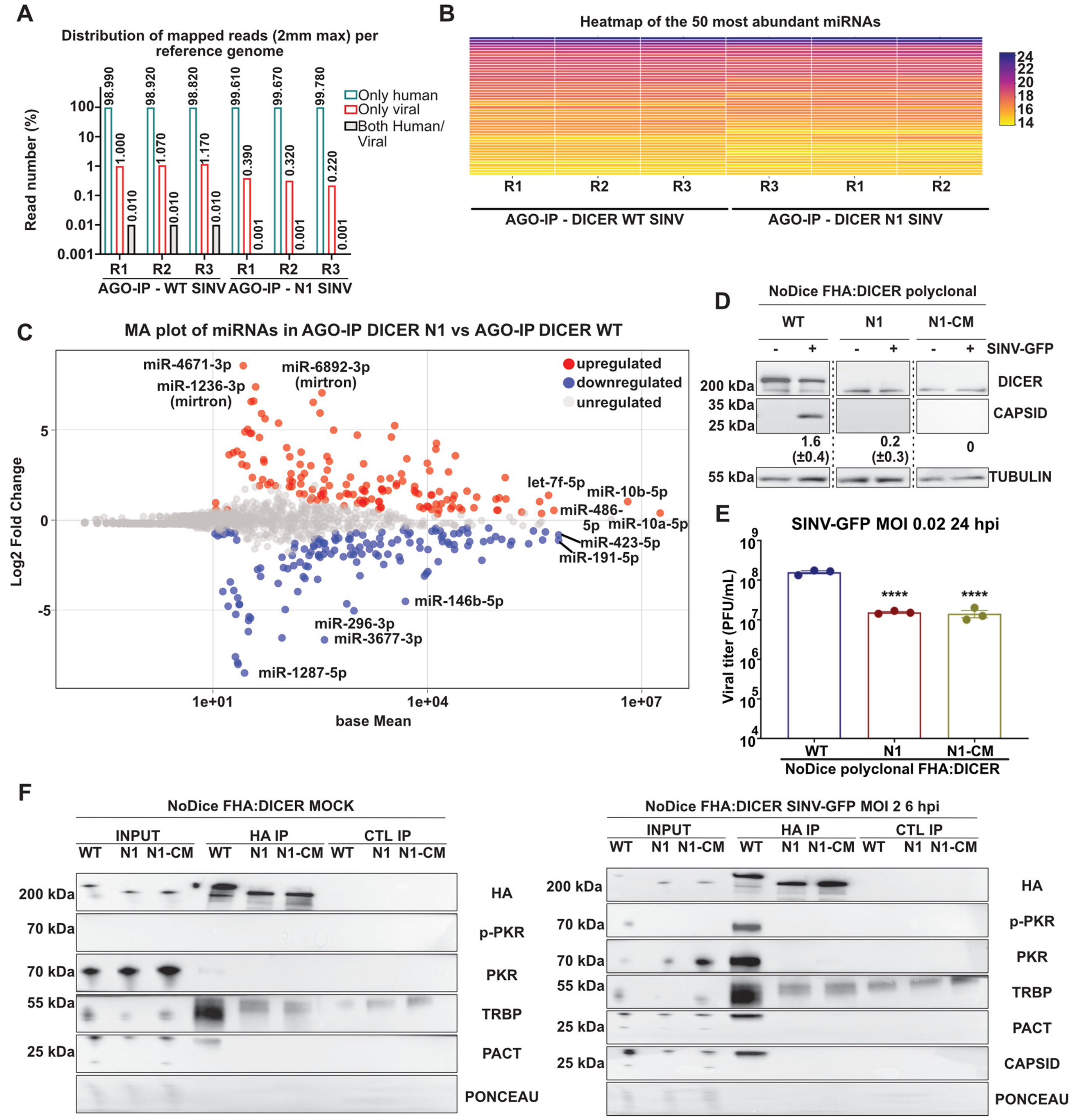
A
Distribution of mapped reads (2mm max) per reference genome
Read number (%)
Only human
Only viral
Both Human/ Viral
AGO-IP - WT SINV
AGO-IP - N1 SINV
B
Heatmap of the 50 most abundant miRNAs
AGO-IP - DICER WT SINV
AGO-IP - DICER N1 SINV
C
MA plot of miRNAs in AGO-IP DICER N1 vs AGO-IP DICER WT
upregulated
downregulated
unregulated
Log2 Fold Change
base Mean
miR-4671-3p
miR-1236-3p (mirtron)
miR-6892-3p (mirtron)
let-7f-5p
miR-10b-5p
miR-486-5p
miR-10a-5p
miR-423-5p
miR-191-5p
miR-146b-5p
miR-296-3p
miR-3677-3p
miR-1287-5p
D
NoDice FHA:DICER polyclonal
SINV-GFP
DICER
CAPSID
TUBULIN
E
SINV-GFP MOI 0.02 24 hpi
Viral titer (PFU/mL)
NoDice polyclonal FHA:DICER
F
NoDice FHA:DICER MOCK
NoDice FHA:DICER SINV-GFP MOI 2 6 hpi
INPUT
HA IP
CTL IP
HA
p-PKR
PKR
TRBP
PACT
CAPSID
PONCEAU

 © The Author(s)

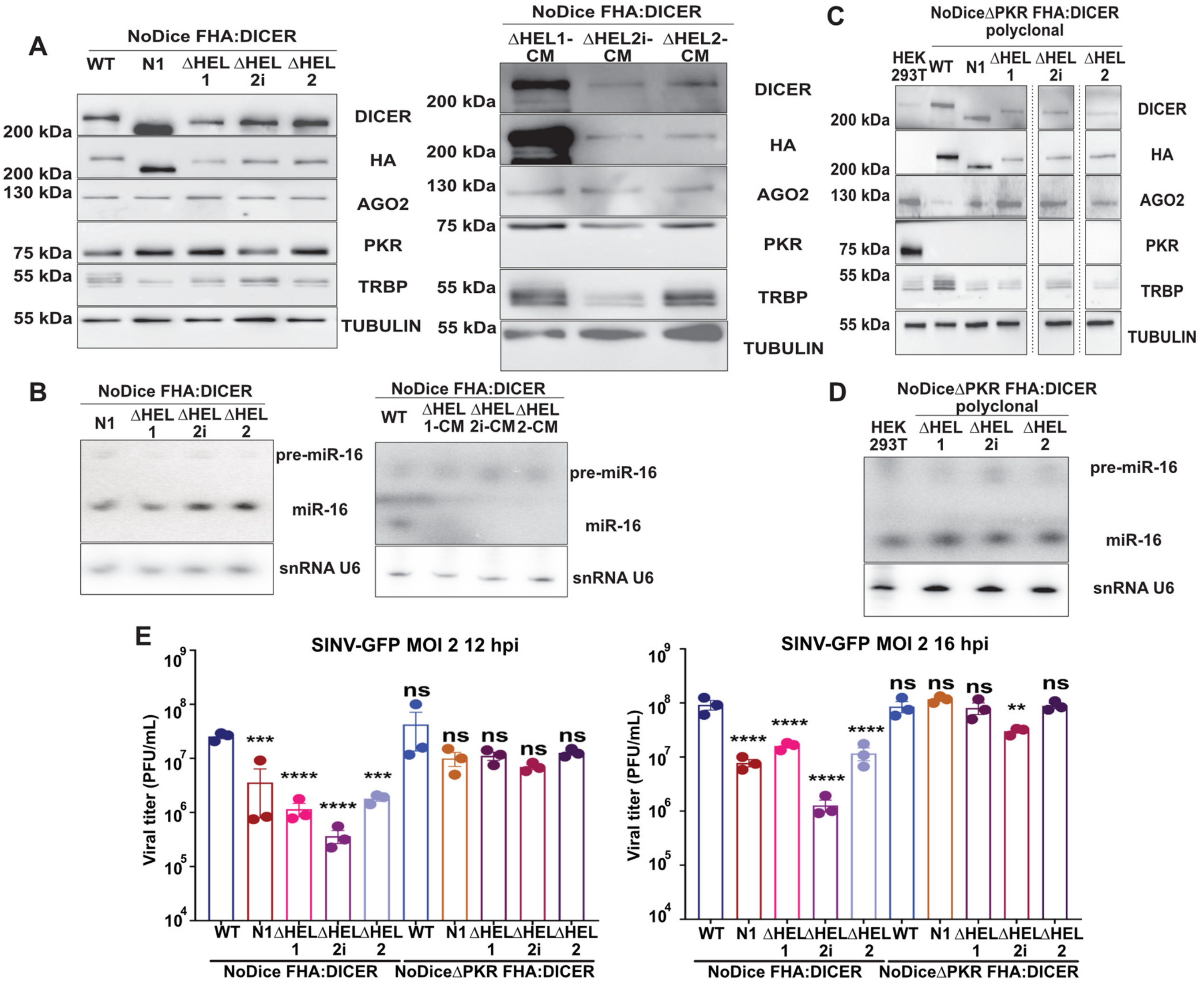

Figure EV2. The helicase sub-domains as well as PKR are important for Dicer antiviral activity.

(**A**) Western blot analysis of DICER, HA, AGO2, PKR and TRBP expression in the monoclonal cell lines NoDice FHA:DICER WT, N1, ΔHEL1, ΔHEL2i and ΔHEL2 (left, $n = 3$ biological replicates) and ΔHEL1-CM, ΔHEL2i-CM and ΔHEL2-CM (right, $n = 2$ biological replicates). Alpha-Tubulin was used as a loading control. (**B**) Northern blot analysis of mirR-16 expression in the same samples as in (A) ($n = 3$ biological replicates for ΔHEL1, 2i and 2; $n = 2$ biological replicates for ΔHEL1-CM, 2i-CM and 2-CM). Expression of snRNA U6 was used as a loading control. (**C**) Western blot analysis of DICER, HA, AGO2, PKR and TRBP expression in the polyclonal cell lines NoDiceΔPKR FHA:DICER WT, N1, ΔHEL1, ΔHEL2i and ΔHEL2 ($n = 1$ biological replicate). Alpha-Tubulin was used as a loading control. (**D**) Northern blot analysis of mirR-16 expression in the same samples as in (**C**) ($n = 2$ biological replicates). Expression of snRNA U6 was used as a loading control. (**E**) Mean (+/− SEM) of SINV-GFP viral titers in the polyclonal cell lines NoDice and NoDiceΔPKR FHA:DICER WT, N1, ΔHEL1, ΔHEL2i and ΔHEL2, infected at an MOI of 2 for 12 h (left) or 16 h (right) from plaque assay quantification ($n = 3$ biological replicates). Ordinary one-way ANOVA test with Dunnett's correction. $^{**}p < 0.01$; $^{***}p < 0.001$; $^{****}p < 0.0001$; ns: non-significant. Source data are available online for this figure.

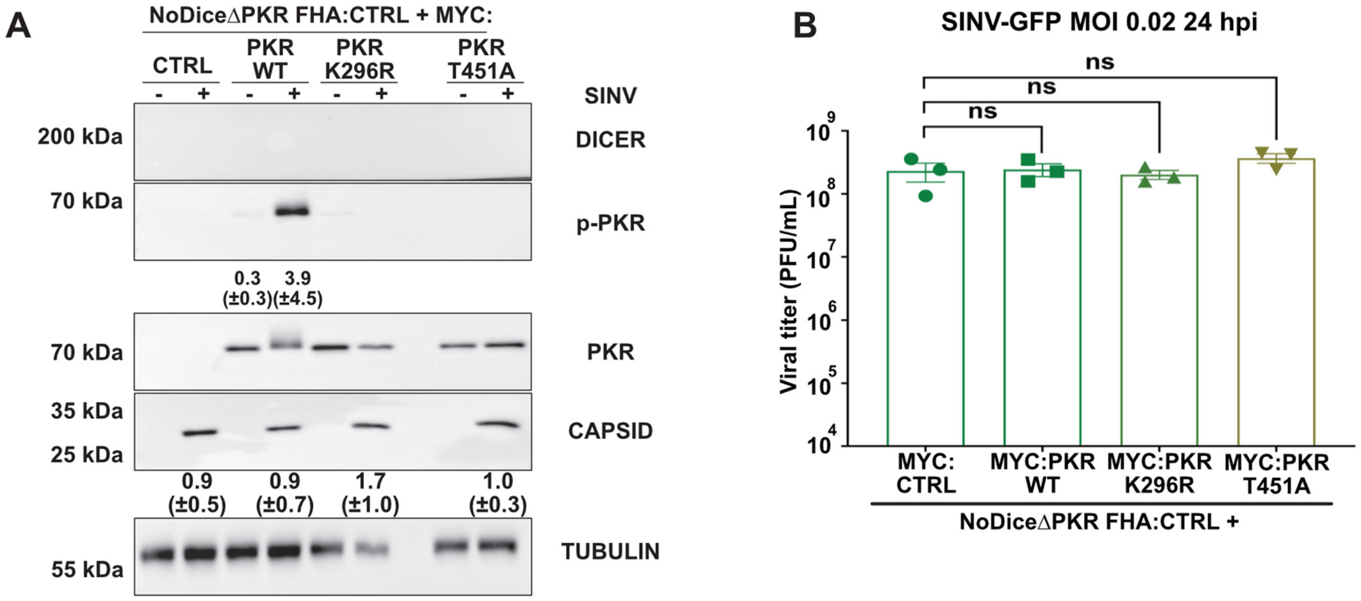

Figure EV3. PKR dimerization and/or catalytic activities do not change the infection outcome in NoDiceΔPKR FHA:CTRL cells.

(**A**) Western blot analysis of DICER, p-PKR, PKR and CAPSID expression in SINV-GFP infected NoDiceΔPKR FHA:CTRL cells expressing MYC:EMPTY CTRL vector, MYC:PKR, MYC K296R or MYC:T451A at an MOI of 0.02 for 24 h. Alpha-Tubulin was used as loading control. Band intensity was quantified and normalized to Tubulin for three independent biological replicates, then represented as mean (+/− SD) under the corresponding lane; p-PKR = p-PKR/PKR quantification. (**B**) Mean (+/− SEM) of SINV-GFP viral titers in the same samples as in (A), infected at an MOI of 0.02 for 24 h ($n = 3$ biological replicates) from plaque assay quantification. Ordinary one-way ANOVA test with Dunnett's correction. ns: non-significant. Source data are available online for this figure.

 © The Author(s)

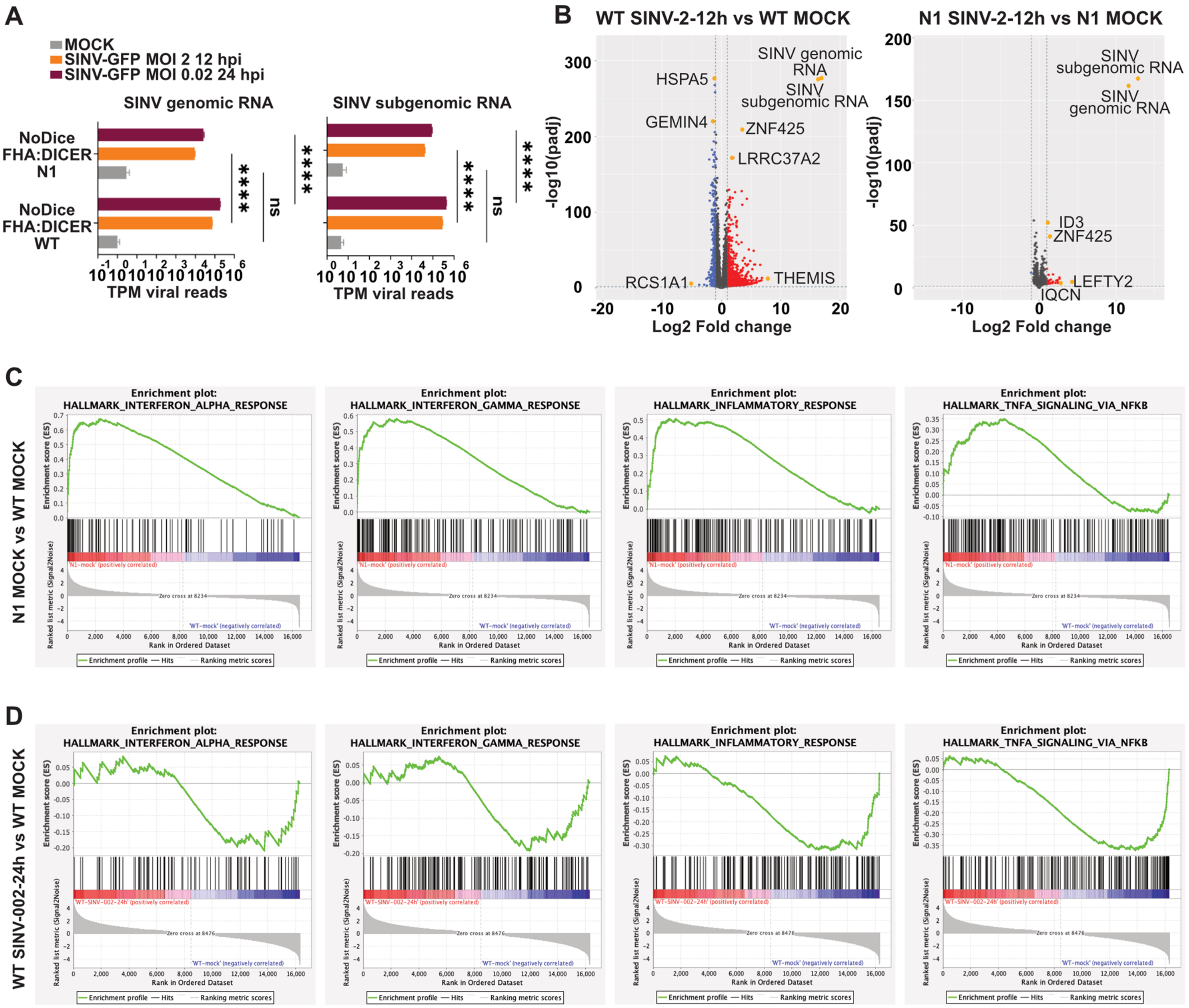

Figure EV4. Dicer N1 cells express a different gene set from Dicer WT cells.

(**A**) SINV-GFP genomic and subgenomic read distribution detected by RNA-sequencing analysis in NoDice FHA:DICER WT or N1 cells uninfected (Mock, gray) or infected at an MOI of 2 for 12 h (orange) or 0.02 for 24 h (purple). The viral reads number (mean +/− SEM) is normalized to the total mapped reads in each condition. TPM: transcripts per million. Ordinary two-way ANOVA test with Sidak's correction. ****$p < 0.0001$; ns: non-significant. $n = 3$ biological replicates, error bars represent SEM. (**B**) Volcano plots showing for each gene the log2 fold change and adjusted p value (Wald test, DESeq2 package) between SINV-infected (MOI of 2 for 12 h) and mock NoDice FHA:DICER WT cells (left), or SINV-infected and mock NoDice FHA:DICER N1 cells (right). Each gene is marked as a dot (red: upregulated, blue: downregulated, gray: unchanged). The horizontal line denotes an adjusted *p*-value of 0.05 and the vertical ones the Log2 fold change cut-offs (−1 and 1) ($n = 3$ biological replicates). (**C**,**D**) GSEA enrichment plots for selected biological states and processes linked to inflammatory and antiviral pathways for mock NoDice FHA:DICER N1 vs mock NoDice FHA:DICER WT (**C**), or SINV-infected (MOI of 0.02 for 24 h) vs. mock NoDice FHA:DICER WT cells (**D**). Source data are available online for this figure.

© The Author(s)

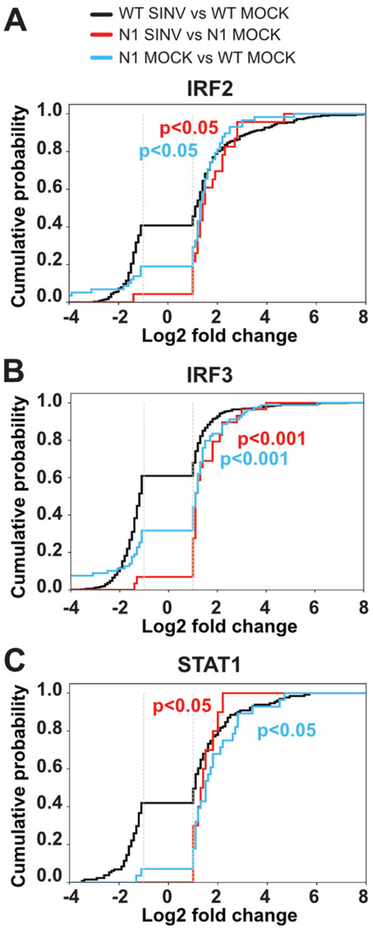

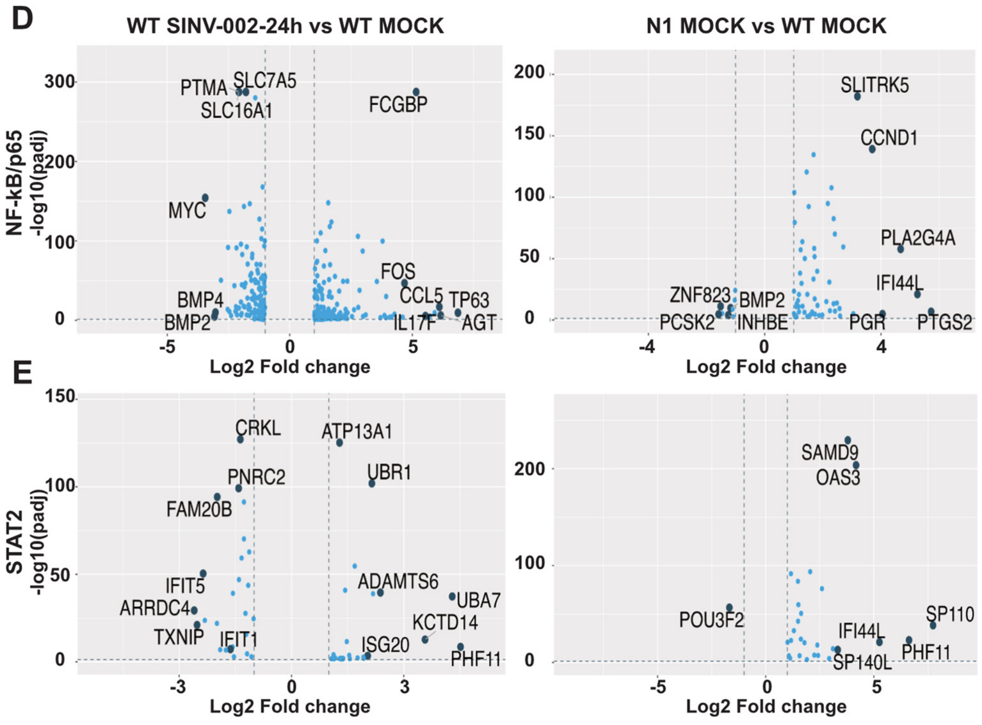

F

Gene	Log2 Fold Change	padj
PTGS2	5.7250	1.48E-07
IFI44L	5.2543	9.44E-22
PLA2G4A	4.6765	1.60E-58
PGR	4.0523	1.26E-05
CCND1	3.6990	6.67E-140
SLITRK5	3.1897	7.75E-183
TLL1	3.0494	6.81E-06
LGALS1	2.7020	2.92E-60
HGF	2.5920	0.0004199
PSMB9	2.5752	3.98E-10
VEGFC	2.4830	1.42E-14
MX1	2.4202	5.55E-08
CR2	2.4149	9.30E-71
ABCB1	2.3721	2.97E-83
APOBEC3B	1.6823	2.37E-135

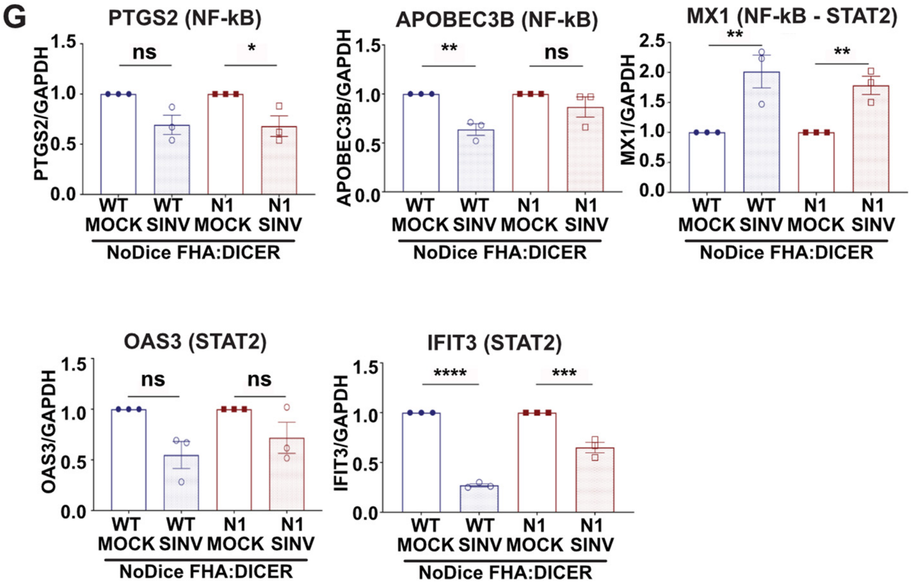

Figure EV5. Immune-related transcription factors activation is involved in the deregulation of Dicer N1 cells mRNAs.

(**A–C**) Histograms representing the cumulative probability of differentially expressed genes controlled by the transcription factors IRF2 (**A**), IRF3 (**B**) or STAT1 (**C**), plotted according to their Log2 fold change. The vertical lines stand for the Log2 fold change cut-offs (−1 and 1). The two-sample Kolmogorov–Smirnov test was used to assess whether each distribution was statistically different from the distribution of NoDice FHA:DICER WT cells infected with SINV vs. mock. *p*-values are indicated on each histogram. Black: WT SINV-002-24h vs WT MOCK; red: N1 SINV-002-24h vs N1 MOCK; blue: N1 MOCK vs WT MOCK. (**D,E**) Volcano plots for differentially expressed genes (DEGs) under the control of NF-kB/p65 (**D**) or STAT2 (**E**). Each gene is marked as a dot and plotted based on its log2 fold change and adjusted p values (Wald test, DESeq2 package) comparing SINV-infected (MOI of 0.02 for 24 h) vs mock NoDice FHA:DICER WT cells (left), or mock NoDice FHA:DICER WT vs mock NoDice FHA:DICER N1 cells (right). The horizontal line denotes an adjusted p-value of 0.05 and the vertical ones the Log2 fold change cut-offs (−1 and 1). $n = 3$ biological replicates. (**F**) Table of 15 representative upregulated NF-kB/p65 targets from the DEGs in the comparison mock NoDice FHA:DICER N1 vs mock NoDice FHA:DICER WT. Classification was made according to their Log2 fold change values. padj: adjusted *p*-value (Wald test, DESeq2 package). (**G**) RT-qPCR on selected DEGs controlled by either NF-kB/p65 or STAT2 in NoDice FHA:DICER WT and N1 cells infected or not with SINV-GFP at an MOI of 0.02 for 24 h. Mean (+/− SEM); $n = 3$ biological replicates. One-way ANOVA with Sidak's correction. *$p < 0.05$; **$p < 0.01$; ***$p < 0.001$; ****$p < 0.0001$; ns: non-significant. Source data are available online for this figure.

 © The Author(s)
